# Supplementary material for: Iron-catalyzed fluoroalkylative alkylsulfonylation of alkenes via radical-anion relay
Source: Nat Commun. 2024 Feb 17;15:1480. doi: 10.1038/s41467-024-45867-y (PMC10874428; doi:10.1038/s41467-024-45867-y)
Supplement: Supplementary file 1 — Supplementary Information [file 41467_2024_45867_MOESM1_ESM.pdf]

Supplementary information

Iron-catalyzed fluoroalkylative alkylsulfonylation of alkenes via radical-anion relay

Xiaoya Hou[1], Hongchi Liu[1], and Hanmin Huang[1,2*]

[1]Key Laboratory of Precision and Intelligent Chemistry, and Department of Chemistry, University of Science and Technology of China, Hefei, 230026, P. R. China.
[2]Key Laboratory of Green and Precise Synthetic Chemistry and Applications, Ministry of Education, Huaibei Normal University; Huaibei, 235000, P. R. China.
*Corresponding author: hanmin@ustc.edu.cn

CONTENTS

Table of contents

1. Supplementary Methods 3
2. Optimization of the Reaction Conditions 5
3. General Procedure for the Catalytic Reaction 9
   3.1 ***General procedure I*** for four-component reactions: 9
   3.2 ***General procedure II*** for tree-component reactions: 9
4. Starting Materials Preparation 10
   4.1 Alkenes 10
   4.2 Radical precursor 15
   4.3 Alkyl bromides 17
5. Experimental Characterization Data for Products 19
6. Synthetic Transformation of Products 54
   6.1 Gram-scale synthesis of **9** 54
   6.2 Post-functionalization of product **9** 54
   6.3 Gram-scale synthesis of **104** 57
   6.4 Synthesis the intermediates of Erysolin and its derivatives 58
7. Mechanistic Experiments 60
   7.1 Radical clock experiments 60

7.2 Reaction with stoichiometric amount of [Fe] 61
7.3 Sparging experiment set up..62
7.4 Reaction profiles...63
8. X-ray Crystal Structure Analysis 64
8.1 X-ray crystal structure of **27** 64
8.2 X-ray crystal structure of **38** 65
8.3 X-ray crystal structure of **40** 66
8.4 X-ray crystal structure of Fe(DPEphos)Cl_2 67
9. Supplementary NMR Spectra of Products 68
10. Supplementary References...250

1. Supplementary Methods

Methods:

All non-aqueous reactions and manipulations were using standard Schlenk techniques. Flash column chromatography was performed using 200-300 mesh silica gels. Thin layer chromatography was used for product detection using silica gel-coated plates, with visualization affected via exposure to UV Light (λ_{ex} = 254 nm).

Materials and Reagents:

$FeCl_2$ (Ferrous chloride, CAS = 7758-94-3), DPEphos (1-(diphenylphosphino)-2-(2-(diphenylphosphino)phenoxy)benzene, CAS = 166330-10-5) were purchased from Energy Chemical and used as received. Unless otherwise noted, other reagents and solvents were purchased from commercial suppliers including Energy Chemical, Bidepharm, Adamas, Leyan, Aladdin, Macklin, J&K Scientific, Sigma-Aldrich, TCI, Alfa Aesar, Acros Organics, Cambridge Isotope Laboratories, or Strem and used as received. Anhydrous solvents (CH_3CN (acetonitrile), ^{i}PrOH (isopropyl alcohol), THF (tetrahydrofuran), DCM (dichloromethane), DMF (dimethylformamide) and DMAc (*N*,*N*-dimethylacetamide)) were purchased from Energy Chemical, stored under N_2 atmosphere and degassed by standard methods before using. Deuterated solvents were purchased from Energy Chemical.

Instrumentation:

Nuclear magnetic resonance spectra (^{1}H NMR, ^{13}C NMR, ^{19}F NMR) were recorded on BRUKER Avance III 400 or 500 MHz NMR spectrometers. Chemical shifts are reported in parts per million (ppm, δ), downfield from tetramethylsilane (TMS, δ = 0.00 ppm) and are referenced to residual solvent ($CDCl_3$, δ = 7.26 ppm (^{1}H) and 77.16 ppm (^{13}C)). Coupling constants (*J*) were reported in Hertz (Hz) and referred to apparent peak multiplications. Data for 1H NMR (400 MHz, CDCl3) spectra were reported as follows: chemical shift (ppm), multiplicity (s = singlet, d = doublet, t = triplet, q = quartet, dd = doublet of doublets, td = triplet of doublets, m = multiplet, brs = broad singlet), coupling constants (Hz) and integration. High resolution mass spectra (HRMS) were recorded on Bruker MicroTOF-QII mass instrument (ESI). Gas chromatography (GC) analyses were performed on Agilent 7890B instrument with Hp-5 column. GC-MS analyses were performed on Thermo Trace 1330/ISQ QD GC-MS system.

Abbreviations:

Me = methyl, Et = ethyl, ^{n}Bu = *normal*-butyl, ^{i}Pr = isopropyl, Bn = benzyl, Ph = phenyl, Bz = Benzoyl, Boc = *tert*-butyloxycarbonyl, TMS = trimethylsilyl, Ts = tosyl.

DMF = dimethylformamide, DMAc = *N*,*N*-dimethylacetamide, EtOAc = ethyl acetate, DCM = dichloromethane, THF = tetrahydrofuran, CH_3CN = acetonitrile, $^{i}PrOH$ = isopropyl alcohol, EA = ethyl acetate, PE = petroleum ether.

DPEphos = 1-(diphenylphosphino)-2-(2-(diphenylphosphino)phenoxy)benzene, TERPY = 2,2':6',2"-terpyridine, PPh_3 = triphenylphosphine, Au_2P^nBu = di(1-adamantyl)-n-butylphosphine, DPPE = 1,2-bis(diphenylphosphanyl)ethane, Xantphos = 4,5-bis(diphenylphosphino)-9,9-dimethylxanthene, BINAP = 1.1'-Binaphthyl-2.2'-diphemyl phosphine.

equiv. = equivalents, g = grams, mg = milligrams, aq. = aqueous, n. d. = not detected, dr = diastereomer ratio, rr = regioisomer ratio.

2. Optimization of the Reaction Conditions

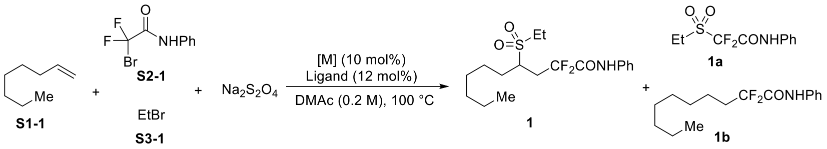

In a N_2 atmosphere-controlled glove box, metal (10 mol%), ligand (12 mol%), 2-bromo-2,2-difluoro-*N*-phenylacetamideand (**S2-1**, 60.0 mg, 0.24 mmol, 1.2 equiv.), $Na_2S_2O_4$ (52.2 mg, 0.30 mmol, 1.5 equiv.) and solvent (1.0 mL) were added to a 25 mL flame-dried Young-type tube. Then 1-octene (**S1-1,** 22.4 mg, 0.20 mmol, 1.0 equiv.) and bromoethane (**S3-1**, 32.7 mg, 0.30 mmol, 1.5 equiv.) were added to the mixture under N_2 atmosphere. The reaction mixture was stirred at 100 ^{o}C. After stirring for 12 hours and monitored by TLC, the reaction was cooled to room temperature. The reaction mixture was diluted with H_2O. Then aqueous phase was extracted with ethyl acetate (3 × 5 mL). The combined organic extracts were washed with brine, dried over Na_2SO_4, filtered, and concentrated in vacuo. The residue was purified by silica gel column chromatography to give the desired product **1** and the by-products **1a** and **1b**.

2-(Ethylsulfonyl)-2,2-difluoro-*N*-phenylacetamide (1a)

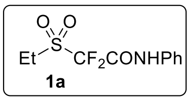

The title compound was prepared according to the above method and purified by column chromatography (EA:PE = 0-10%) as light yellow oil. **^{1}H NMR** (400 MHz, $CDCl_3$) δ 8.16 (s, 1H), 7.64 – 7.50 (m, 2H), 7.40 (t, *J* = 7.9 Hz, 2H), 7.25 (d, *J* = 4.8 Hz, 1H), 3.44 (q, *J* = 7.5 Hz, 2H), 1.53 (t, *J* = 7.5 Hz, 3H). **^{13}C NMR** (101 MHz, $CDCl_3$) δ 155.76, 135.33, 129.51, 126.65, 120.71, 114.00, 45.19, 5.63. **^{19}F NMR** (376 MHz, $CDCl_3$) δ -112.23 (s, 2F). **HRMS** (ESI) calcd for $C_{10}H_{12}F_2NO_3S$ $[M + H]^+$: 264.0500, found: 264.0497.

2,2-Difluoro-*N*-phenyldecanamide (1b)[1]

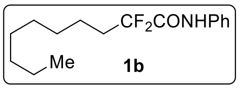

The title compound was prepared according to the above method and purified by column chromatography (EA:PE = 0-1.0%) as a yellow oil. **^{1}H NMR** (400 MHz, $CDCl_3$) δ 7.98 (s, 1H), 7.62 – 7.49 (m, 2H), 7.37 (t, *J* = 8.0 Hz, 2H), 7.20 (t, *J* = 7.4 Hz, 1H), 2.28 – 2.05 (m, 2H), 1.50 (td, *J* = 11.4, 9.8, 5.5 Hz, 2H), 1.44 – 1.21 (m, 10H), 0.88 (t, *J* = 6.7 Hz, 3H). **^{13}C NMR** (101 MHz, $CDCl_3$) δ 162.36 (t, $^2J_{C-F}$ = 27.9 Hz), 136.16, 129.36, 125.68, 120.29, 118.59 (t, $^1J_{C-F}$ = 253.4 Hz), 33.93 (t, $^2J_{C-F}$ = 23.1 Hz), 31.90, 29.35, 29.26, 29.21, 22.76, 21.69 (t, $^3J_{C-F}$ = 4.2 Hz), 14.23. **^{19}F NMR** (376 MHz, $CDCl_3$) δ -105.52 (s, 2F).

Supplementary Table 1. Screening of metal catalysts

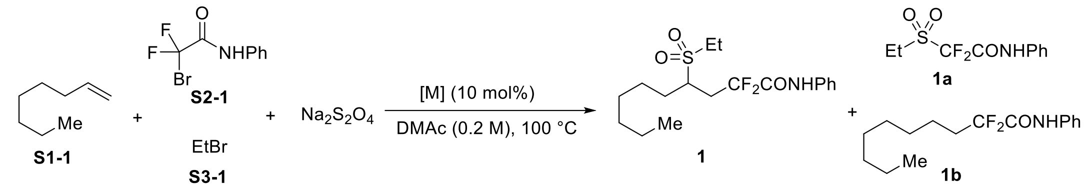

entry	[M]	yield/%[a]		
		**1**	**1a**	**1b**
1	none	26	8.5	22
2	$Ni(OAc)_2 \cdot 4H_2O$	20	5	39
3	$Ni(acac)_2$	25	6	23
4	$Ni(OTf)_2$	26	8	36
5	$NiBr_2 \cdot DME$	26	5	27
6	CuI	25	11	17
7	$Co(acac)_2$	17	9	32
8	$FeCl_2$	34	7.5	0
9	$Fe(OAc)_2$	32	8	0
10	$Fe(acac)_2$	37	8	0
11	$FeCl_3$	35	7	0
12	$Fe(OTf)_3$	34	7.5	0
13	$Fe(acac)_3$	26	8	0

[a]Reaction conditions: **S1-1** (0.20 mmol, 1.0 equiv.), **S2-1** (0.20 mmol, 1.0 equiv.), **S3-1** (0.30 mmol, 1.5 equiv.), [M] (10 mol%), $Na_2S_2O_4$ (0.30 mmol, 1.5 equiv.), DMAc (1.0 mL), 100 ^{o}C, 12 h. Yields were determined by ^{1}H NMR analysis with dibromomethane as an internal standard.

Supplementary Table 2. Screening of ligands

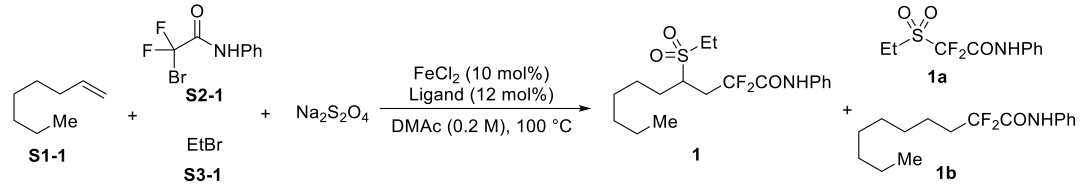

entry	ligand	yield/%[a]		
		**1**	**1a**	**1b**
1	TERPY	32	6.5	0
2	PPh_3	36	6.5	0
3	Au_2P^nBu	29	7	0
4	DPPE	57	7	0
5	Xantphos	60	5	0
6	BINAP	35	6	0
7	DPEphos	67	8.5	0
8[b]	DPEphos	81	trace	0

[a]Reaction conditions: **S1-1** (0.20 mmol, 1.0 equiv.), **S2-1** (0.20 mmol, 1.0 equiv.), **S3-1** (0.30 mmol, 1.5 equiv.), $FeCl_2$ (10 mol%), ligands (12 mol%), $Na_2S_2O_4$ (0.30 mmol, 1.5 equiv.), DMAc (1.0 mL), 100 °C, 12 h. Yields were determined by ^1H NMR analysis with dibromomethane as an internal standard. [b]**S2-1** (0.24 mmol, 1.2 equiv.). Isolated yield.

Supplementary Table 3. Screening of solvents

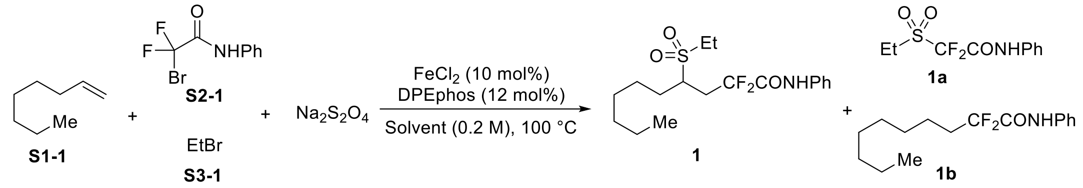

entry	solvent	yield/%[a]		
		**1**	**1a**	**1b**
1	MeCN	0	0	0
2	^{i}PrOH	0	0	0
3	DCM	0	0	trace
4	THF	0	0	0
5	DMF	67	trace	0

[a]Reaction conditions: **S1-1** (0.20 mmol, 1.0 equiv.), **S2-1** (0.24 mmol, 1.2 equiv.), **S3-1** (0.3 mmol,

1.5 equiv.), $FeCl_2$ (10 mol%), DPEPhos (12 mol%), $Na_2S_2O_4$ (0.3 mmol, 1.5 equiv.), solvent (1.0 mL), 100 °C, 12 h. Yields were determined by ^{1}H NMR analysis with dibromomethane as an internal standard.

Supplementary Table 4. Screening of temperature

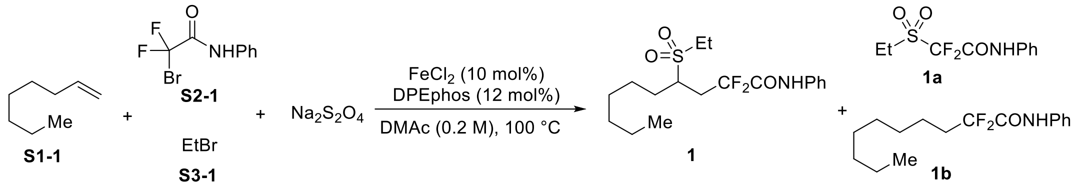

entry	temperature/°C	yield/%[a]		
		**1**	**1a**	**1b**
1	25	trace	0	0
2	40	13	trace	0
3	60	46	12	0
4	80	72	7	0
5	120	82	trace	0

[a]Reaction conditions: **S1-1** (0.20 mmol, 1.0 equiv.), **S2-1** (0.24 mmol, 1.2 equiv.), **S3-1** (0.3 mmol, 1.5 equiv.), $FeCl_2$ (10 mol%), DPEPhos (12 mol%), $Na_2S_2O_4$ (0.3 mmol, 1.5 equiv.), DMAc (1.0 mL), 12 h. Yields were determined by ^{1}H NMR analysis with dibromomethane as an internal standard.

Preparation of ($Fe(DPEphos)Cl_2$)

Anhydrous $FeCl_2$ (1.0 g, 7.9 mmol) and DPEphos (4.7 g, 8.3 mmol) were suspended in THF (70 mL) and heated under reflux. After 15 min, the light pink suspension changed to a brown solution and after 35 min to a very heavy light brown suspension. The mixture was heated for a further 2 h and was filtered hot, giving a beige powder and a brown filtrate. The solid was then washed with THF (40 mL) and THF/hexane (1:3, 45 mL) and dried under vacuum. Yield: 3.8 g (67%). The solid-state structure of $Fe(DPEphos)Cl_2$ was unambiguously determined by single-crystal X-ray crystallographic analysis.

3. General Procedure for the Catalytic Reaction

3.1 *General procedure I* for four-component reactions:

In a N_2 atmosphere-controlled glove box, $FeCl_2$ (5.0 mg, 10 mol%), DPEphos (25.9 mg, 12 mol%), olefins (**S1-1-50**, 0.40 mmol, 1.0 equiv.), radical precursor (**S2-1-13**, 0.48 mmol, 1.2 equiv.), alkyl bromide (**S3-1-18**, 0.60 mmol, 1.5 equiv.), $Na_2S_2O_4$ (104.4 mg, 0.60 mmol, 1.5 equiv.) and DMAc (2.0 mL) were added to a 25 mL flame-dried Young-type tube. The reaction mixture was stirred at 100 ^oC. After stirring for 12 hours and monitored by TLC, the reaction was cooled to room temperature. The reaction mixture was diluted with H_2O. Then aqueous phase was extracted with EtOAc (3 × 10 mL). The combined organic extracts were washed with brine, dried over Na_2SO_4, filtered, and concentrated in vacuo. The residue was purified by silica gel column chromatography to give the corresponding product.

3.2 *General procedure II* for tree-component reactions:

In a N_2 atmosphere-controlled glove box, $FeCl_2$ (5.0 mg, 10 mol%), DPEphos (25.9 mg, 12 mol%), 2-bromo-2,2-difluoro-*N*-phenylacetamide **S2-1** (120.0 mg, 0.48 mmol, 1.2 equiv.), $Na_2S_2O_4$ (104.4 mg, 0.60 mmol, 1.5 equiv.) and DMAc (4.0 mL) were added to a 25 mL flame-dried Young-type tube. Then **S1-51 - 63** (0.4 mmol, 1.0 equiv.) were added to the mixture under N_2 atmosphere. The reaction mixture was stirred at 100 ^oC. After stirring for 12 hours and monitored by TLC, the reaction was cooled to room temperature. The reaction mixture was diluted with H_2O. Then aqueous phase was extracted with ethyl acetate (3 × 20 mL). The combined organic extracts were washed with brine, dried over Na_2SO_4, filtered, and concentrated in vacuo. The residue was purified by silica gel column chromatography to give the corresponding product.

4. Starting Materials Preparation

4.1 Alkenes

Substrates **S1-(1-10, 12-14, 16-29, 31-32, 34-47, 51, 53-58, 60-63, 67)** were purchased from commercial sources and used as received. Other alkene substrates **S1-(11**, **15, 30**,**33**, **48, 49**, **50**, **52, 59, 64-66, 68**) were prepared according to the procedures reported in the literature.[2-10]

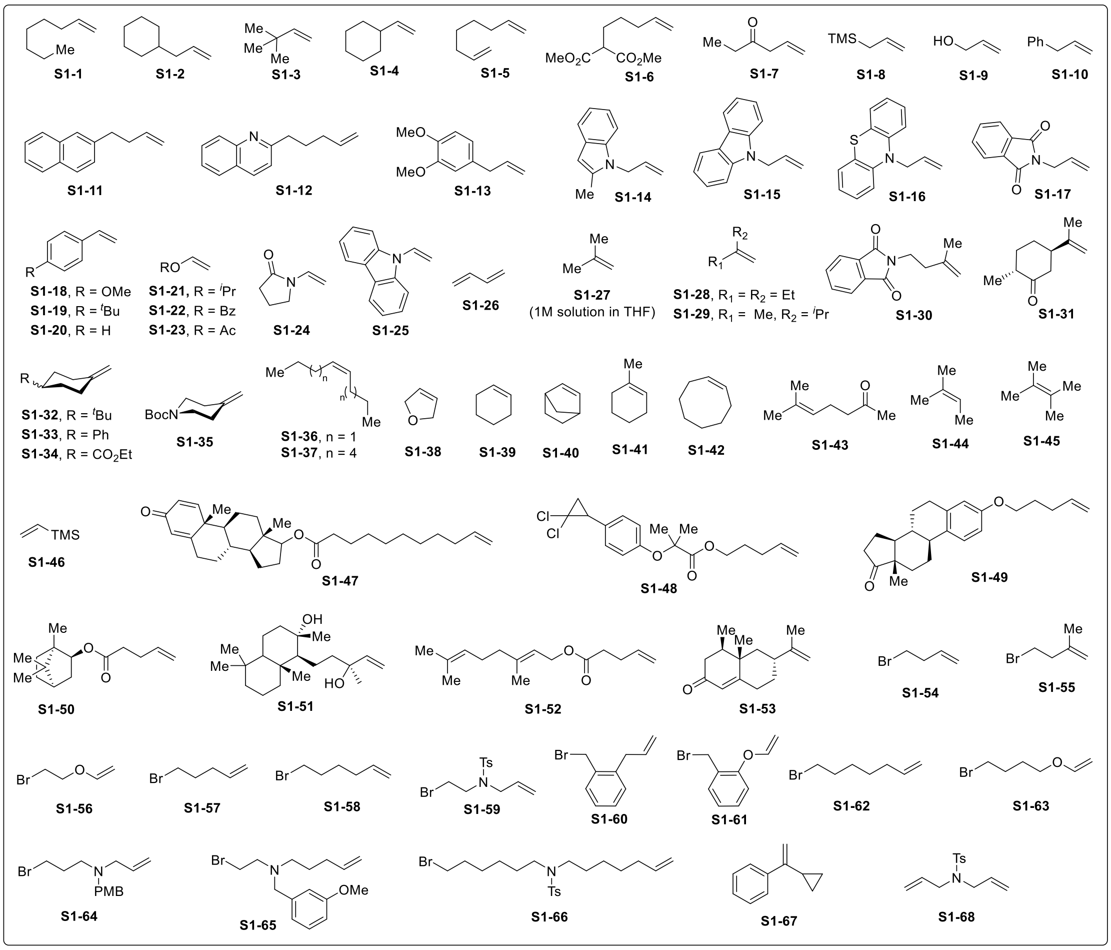

Supplementary Figure 1. Alkene substrates used in the reaction

2-(But-3-en-1-yl)naphthalene (S1-11)

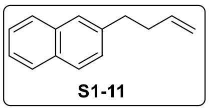

Following a reported procedure[2], 2-(bromomethyl)naphthalene (2.21 g, 10 mmol, 1.0 equiv.) and 20 mL dry THF were added to a dried 100 mL round-bottom flask, then allylmagnesium bromide (1.0 M solution in Et_2O, 1.5 equiv.) was added dropwise at 0 °C. After being stirred at room temperature for 4 h, the reaction mixture was quenched with sat. NH_4Cl solution, extracted with EtOAc (3×30 mL), dried over anhydrous Na_2SO_4, and concentrated under reduced pressure. Purification via column chromatography on silica gel (PE) afforded **S1-11** as a colorless oil (1.47 g, 81% yield). **^{1}H NMR** (400 MHz, $CDCl_3$) δ 7.84 – 7.73 (m, 3H), 7.65 (s, 1H), 7.51 – 7.41 (m, 2H), 7.37 (dd, *J* = 8.4, 1.8 Hz, 1H), 6.04 – 5.81

(m, 1H), 5.20 – 4.95 (m, 2H), 3.05 – 2.79 (m, 2H), 2.68 – 2.40 (m, 2H). Spectral data are in accordance with the literature.[2]

***N*-Allyl-carbazole (S1-15)**

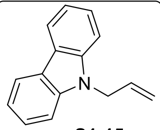
S1-15

Following a reported procedure[3], under air, Cs_2CO_3 (1.95 g, 6.0 mmol, 1.2 equiv.) was added to a solution of carbazole (836 mg, 5.0 mmol, 1.0 equiv.) in DMF (50 mL). Allyl bromide (475 μL, 5.5 mmol, 1.1 equiv.) was added and after stirring overnight, the reaction mixture was diluted with CH_2Cl_2 and quenched with H_2O. The phases were separated and the organics were washed with H_2O, dried over $MgSO_4$, concentrated in vacuo and purified by column chromatography on silica gel (EA:PE = 0-5%) to afford **S1-15** as a white solid (0.97 g, 94%). **^{1}H NMR** (400 MHz, $CDCl_3$) δ 8.18 – 8.07 (m, 2H), 7.54 – 7.42 (m, 2H), 7.42 – 7.36 (m, 2H), 7.31 – 7.21 (m, 2H), 6.07 – 5.91 (m, 1H), 5.23 – 5.11 (m, 1H), 5.08 – 4.99 (m, 1H), 4.96 – 4.86 (m, 2H). Spectral data are in accordance with the literature.[3]

2-(3-Methylbut-3-en-1-yl)isoindoline-1,3-dione (S1-30)

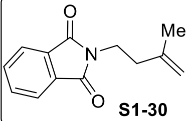
S1-30

Following a reported procedure[4], to a two-necked 100 mL flask were added 3-methylbut-3-en-1-ol (861.3 mg, 10.0 mmol, 1.0 equiv.), phthalimide (1.77 g, 12.0 mmol, 1.2 equiv.), PPh_3 (3.15 g, 12.0 mmol, 1.2 equiv.) and 10 mL anhydrous THF under argon atmosphere, then diisopropyl azodicarboxylate (2.43 g, 12.0 mmol, 1.2 equiv.) in 4 mL THF was added dropwise to the solution. The reaction was stirred at room temperature for 3 hours. The reaction mixture was concentrated under vacuum and further purified by flash column chromatography (EA:PE = 0-5%) to afford **S1-30** as a white solid (2.00 g, 93% yield). **^{1}H NMR** (400 MHz, $CDCl_3$) δ 7.84 (d, J = 3.1 Hz, 1H), 7.83 (d, J = 3.0 Hz, 1H), 7.71 (d, J = 3.0 Hz, 1H), 7.70 (d, J = 3.0 Hz, 1H), 4.79 – 4.52 (m, 2H), 3.94 – 3.62 (m, 2H), 2.39 (t, J = 6.8 Hz, 2H), 1.95 – 1.74 (m, 3H). Spectral data are in accordance with the literature.[4]

(4-Methylenecyclohexyl)benzene (S1-33)

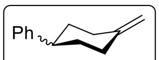
S1-33

Following a reported procedure[5], to a solution of *t*-BuOK (1.68 g, 15.0 mmol, 1.5 equiv.) in dry Et_2O (20 mL) was added Ph_3PCH_2Br (5.36 g, 15.0 mmol, 1.5 equiv.) under Ar at rt. The solution turned to yellow. The reaction mixture was stirred 30 min. 4-phenylcyclohexanone (1.74 g, 10 mmol, 1.0 equiv.) in dry Et_2O (7.0 mL) was then canulated into the reaction mixture. It was then stirred 1.5 h at reflux. The reaction mixture was then diluted into Et_2O and was washed with water. The organic layer was then dried over $MgSO_4$, filtered and concentrated under reduced pressure. The crude mixture was then purified by flash chromatography (EA:PE = 0-5%) to give **S1-33** as a colorless oil (1.72 g, quant. yield). **^{1}H NMR** (400 MHz, $CDCl_3$) δ 7.33 – 7.24 (m, 2H), 7.22 – 7.11 (m, 3H), 4.68 (t, J = 1.8 Hz, 2H), 2.72 – 2.56 (m, 1H), 2.46 –

2.34 (m, 2H), 2.24 – 2.09 (m, 2H), 2.04 – 1.87 (m, 2H), 1.63 – 1.45 (m, 2H). Spectral data are in accordance with the literature.[5]

Pent-4-en-1-yl 2-(4-(2,2-dichlorocyclopropyl)phenoxy)-2-methylpropanoate (S1-48)

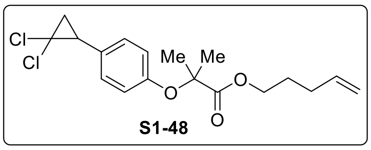

Following a reported procedure[6], ciprofibrate (2.89 g, 10 mmol, 1.0 equiv.) and potassium carbonate (2.07g, 15 mmol, 1.5 equiv.) were combined in acetonitrile (10 mL) under air at room temperature. 5-Bromo-1-pentene (1.94 g, 13 mmol, 1.3 equiv.) was added slowly and the reaction was stirred for 24 h at 70 °C. The reaction mixture was diluted with CH_2Cl_2 and $NaHCO_3$ and extracted with CH_2Cl_2. The organic layer was dried over Na_2SO_4. After removal of solvent, the residue was purified by flash chromatography (EA:PE = 0-5%) to give **S1-48** as colorless oil (2.63 g, 91% yield). **^{1}H NMR** (400 MHz, $CDCl_3$) δ 7.14 – 7.02 (m, 2H), 6.80 (d, *J* = 8.7 Hz, 2H), 5.81 – 5.61 (m, 1H), 5.04 – 4.84 (m, 2H), 4.16 (t, *J* = 6.5 Hz, 2H), 2.96 – 2.77 (m, 1H), 2.03 – 1.89 (m, 3H), 1.81 – 1.64 (m, 3H), 1.61 (s, 6H). **^{13}C NMR** (101 MHz, $CDCl_3$) δ 174.41, 155.07, 137.37, 129.77, 128.12, 118.45, 115.53, 79.21, 64.96, 60.97, 34.89, 29.97, 27.65, 25.91, 25.56, 25.53.

(8*R*,9*S*,13*S*,14*S*)-13-Methyl-3-(pent-4-en-1-yloxy)-6,7,8,9,11,12,13,14,15,16-decahydro-17*H*-cyclopenta[*a*]phenanthren-17-one (S1-49)

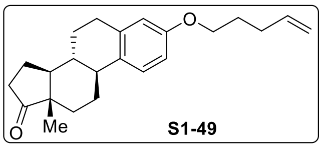

Following a reported procedure[7], a mixture of 5-bromo-1-pentene (1.94 g, 13.0 mmol, 1.3 equiv.), Estrone (2.70 g, 10 mmol, 1.0 equiv.), and potassium carbonate (2.07 g, 15.0 mmol, 1.5 equiv.) in anhydrous acetonitrile was heated to reflux in an oil bath. After refluxing overnight, the reaction mixture was cooled to room temperature, concentrated under reduced pressure, diluted with saturated NH_4Cl and extracted with ethyl acetate. The combined organic layers were dried with Na_2SO_4, filtered, and concentrated under reduced pressure. The residue was purified by silica gel column chromatography (EA:PE = 0-30%) to afford **S1-49** as a white solid (2.90 g, 97% yield). **^{1}H NMR** (400 MHz, $CDCl_3$) δ 7.23 – 7.14 (m, 1H), 6.82 – 6.67 (m, 1H), 6.65 (d, *J* = 2.8 Hz, 1H), 5.94 – 5.74 (m, 1H), 5.19 – 4.91 (m, 2H), 3.94 (t, *J* = 6.4 Hz, 2H), 3.00 – 2.78 (m, 2H), 2.56 – 2.42 (m, 1H), 2.44 – 2.37 (m, 1H), 2.28 – 1.80 (m, 9H), 1.67 – 1.40 (m, 6H), 0.91 (s, 3H). **^{13}C NMR** (101 MHz, $CDCl_3$) δ 157.15, 138.03, 137.85, 132.00, 126.45, 115.28, 114.63, 112.21, 77.36, 67.16, 50.48, 48.16, 44.09, 38.47, 36.03, 31.67, 30.27, 29.79, 28.59, 26.68, 26.04, 21.72, 13.98. Spectral data are in accordance with the literature.[7]

S1-50 and **S1-52** were prepared following a reported procedure.[8]

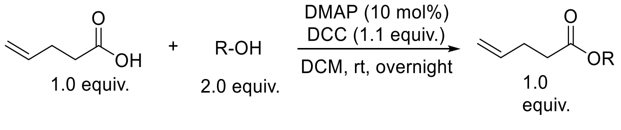

To a stirred solution of pent-4-enoic acid (1.00 g, 10.0 mmol, 1.0 equiv.) in 30 mL anhydrous CH_2Cl_2 is added DMAP (122.2 mg, 1.0 mmol) and alcohol (20.0 mmol, 2.0 equiv.). DCC (2.27 g, 11.0 mmol, 1.1 equiv.) was then added to the reaction mixture at 0 ^oC, and then allowed to stir overnight at room temperature. Precipitated urea was then filtered off. Filtrate was evaporated and the residue was dissolved in CH_2Cl_2 and was washed twice with saturated $NaHCO_3$ solution, and then dried over $MgSO_4$. The solvent was removed by evaporation and the residue was purified by column chromatography (EA:PE = 0-2%) to give the desired product **S1-50** (1.45g, 61% yield) and **S1-52** (1.51g, 64% yield) as colorless oil.

(1*R*,2*S*,4*R*)-1,7,7-Trimethylbicyclo[2.2.1]heptan-2-yl pent-4-enoate (S1-50)

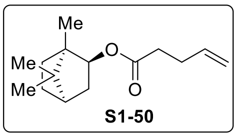

^{1}H NMR (400 MHz, $CDCl_3$) δ 5.90 – 5.75 (m, 1H), 5.12 – 4.96 (m, 2H), 4.92 – 4.83 (m, 1H), 2.49 – 2.25 (m, 5H), 1.99 – 1.86 (m, 1H), 1.79 – 1.68 (m, 1H), 1.66 (t, *J* = 4.6 Hz, 1H), 1.34 – 1.17 (m, 2H), 0.99 – 0.87 (m, 5H), 0.86 (s, 3H), 0.82 (s, 3H). **^{13}C NMR** (101 MHz, $CDCl_3$) δ 173.51, 136.90, 115.55, 79.94, 48.82, 47.89, 44.95, 36.90, 33.98, 29.18, 28.13, 27.20, 19.82, 18.95, 13.63.

(*E*)-3,7-Dimethylocta-2,6-dien-1-yl pent-4-enoate (S1-52)

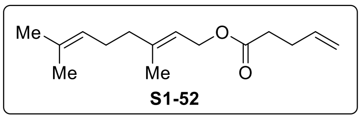

^{1}H NMR (400 MHz, $CDCl_3$) δ 5.94 – 5.75 (m, 1H), 5.44 – 5.30 (m, 1H), 5.13 – 4.91 (m, 3H), 4.57 (dd, *J* = 7.2, 1.0 Hz, 2H), 2.49 – 2.30 (m, 4H), 2.19 – 1.99 (m, *J* = 5.3 Hz, 4H), 1.77 (q, *J* = 1.1 Hz, 3H), 1.68 (d, *J* = 1.3 Hz, 3H), 1.60 (s, 3H). Spectral data are in accordance with the literature.[8]

S1-59, **S1-64**, **S1-65**, **S1-66** and **S1-68** were prepared following a reported procedure.[9]

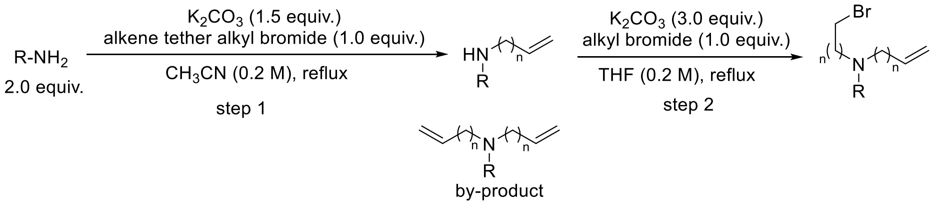

step 1: To a flame-dried round bottom flash with a magnetic stir bar, potassium carbonate (2.07 g, 15.0 mmol, 1.5 equiv.) was added to a solution of primary amine (20 mmol, 2.0 equiv.) and alkene tether alkyl bromide (10 mmol, 1.0 equiv.) in CH_3CN (0.2 M). The reaction was refluxed overnight. Once the reaction was deemed complete via TLC, it was concentrated in vacuo. After removal of solvent, the residue was purified by flash chromatography (EA:PE = 0-50%) to give secondary amine and by-product (EA:PE = 0-5%) as light yellow oil.

step 2: To a high-pressure tube, potassium carbonate (3.0 equiv.) was added slowly to a solution of secondary amine in THF (0.25 M). Alkyl bromide was added and the reaction was heated to

reflux for 48 h. Using ether and water, the organic phase was extracted and concentrated in vacuo. Crude mixture was purified by flash chromatography (EA:PE = 0-10%) to give target product.

***N*-Allyl-*N*-(2-bromoethyl)-4-methylbenzenesulfonamide (S1-59)**

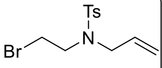
S1-59

Synthesized as a clear oil (1.53 g, 48%). **^{1}H NMR** (400 MHz, $CDCl_3$) δ 7.70 (d, *J* = 8.4 Hz, 2H), 7.32 (d, *J* = 8.0 Hz, 2H), 5.73 – 5.50 (m, 1H), 5.25 – 5.12 (m, 2H), 3.81 (dt, *J* = 6.5, 1.4 Hz, 2H), 3.55 – 3.35 (m, 4H), 2.43 (s, 3H). **^{13}C NMR** (101 MHz, $CDCl_3$) δ 143.68, 136.00, 132.72, 129.82, 127.02, 119.67, 51.91, 48.80, 29.34, 21.47. Spectral data are in accordance with the literature.[9]

***N*-(3-Bromopropyl)-*N*-(4-methoxybenzyl)prop-2-en-1-amine (S1-64)**

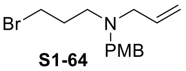
S1-64

Synthesized as a clear oil (1.61 g, 54%). **^{1}H NMR** (400 MHz, $CDCl_3$) δ 7.22 (d, *J* = 8.6 Hz, 2H), 6.85 (d, *J* = 8.6 Hz, 2H), 5.93 – 5.80 (m, 1H), 5.23 – 5.09 (m, 2H), 3.80 (s, 3H), 3.50 (s, 2H), 3.44 (t, *J* = 6.8 Hz, 2H), 3.05 (dt, *J* = 6.4, 1.4 Hz, 2H), 2.55 (t, *J* = 6.7 Hz, 2H), 2.00 (p, *J* = 6.7 Hz, 2H). **^{13}C NMR** (101 MHz, $CDCl_3$) δ 158.65, 135.89, 131.37, 130.04, 117.53, 113.66, 57.66, 56.80, 55.36, 51.39, 32.13, 30.68. **HRMS** (ESI) calcd for $C_{14}H_{21}BrNO$ [M + H]$^+$: 298.0801, found: 298.0803.

***N*-(2-Bromoethyl)-*N*-(3-methoxybenzyl)pent-4-en-1-amine (S1-65)**

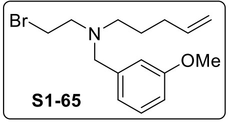
S1-65

Synthesized as a colorless oil (1.16 g, 37%). **^{1}H NMR** (400 MHz, $CDCl_3$) δ 7.27 – 7.16 (m, 1H), 6.92 – 6.70 (m, 3H), 5.77 (dt, *J* = 16.7, 8.4 Hz, 1H), 5.09 – 4.86 (m, 2H), 4.53 – 4.34 (m, 3H), 3.80 (s, 3H), 3.55 (dt, *J* = 20.3, 5.6 Hz, 2H), 3.23 (dt, *J* = 20.7, 7.6 Hz, 2H), 2.08 – 1.94 (m, 2H), 1.64 (p, *J* = 7.5 Hz, 3H). **^{13}C NMR** (126 MHz, $CDCl_3$) δ 160.01, 139.48, 137.93, 129.74, 115.19, 113.51, 112.90, 112.77 64.92, 60.27, 59.88, 55.39, 31.04, 29.94, 25.88. **HRMS** (ESI) calcd for C15H23BrNO [M + H]+: 312.0958, found: 312.0959.

***N*-(6-Bromohexyl)-*N*-(hept-6-en-1-yl)-4-methylbenzenesulfonamide (S1-66)**

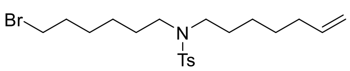
S1-66

Synthesized as a colorless oil (2.03 g, 47%). **^{1}H NMR** (400 MHz, $CDCl_3$) δ 7.66 (d, *J* = 8.3 Hz, 2H), 7.28 (d, *J* = 8.0 Hz, 2H), 5.96 – 5.67 (m, 1H), 5.20 – 4.72 (m, 2H), 3.38 (t, *J* = 6.8 Hz, 2H), 3.14 – 3.00 (m, 4H), 2.41 (s, 3H), 2.08 – 1.94 (m, 2H), 1.81 (p, *J* = 6.9 Hz, 2H), 1.51 (dt, *J* = 15.1, 7.5 Hz, 4H), 1.43 – 1.32 (m, 4H), 1.31 – 1.16 (m, 4H). **^{13}C NMR** (101 MHz, $CDCl_3$) δ 143.07, 138.78, 136.96, 129.67, 127.13, 114.58, 48.30, 48.12, 33.93, 33.69, 32.65, 28.64, 28.61, 28.51, 27.79, 26.24, 25.89, 21.58. **HRMS** (ESI) calcd for $C_{20}H_{33}BrNO_2S$ [M + H]$^+$: 430.1410, found: 430.1415.

N,N-Diallyl-4-methylbenzenesulfonamide (S1-68)

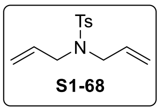

Synthesized as yellow oil (349.3 mg, 14%). **^{1}H NMR** (400 MHz, $CDCl_3$) δ 7.69 (d, *J* = 8.3 Hz, 2H), 7.28 (d, *J* = 7.8 Hz, 2H), 5.74 – 5.39 (m, 2H), 5.29 – 4.91 (m, 4H), 3.78 (dt, *J* = 6.2, 1.3 Hz, 4H), 2.41 (s, 3H). Spectral data are in accordance with the literature.[10]

4.2 Radical precursor

Substrates **S2-(2-3, 5**, **7-11**) were purchased from commercial sources and used as received. Other alkyl bromides **S2-(1, 4, 6, 12, 13, 14**) were prepared according to the procedures reported in the literature.[11-15]

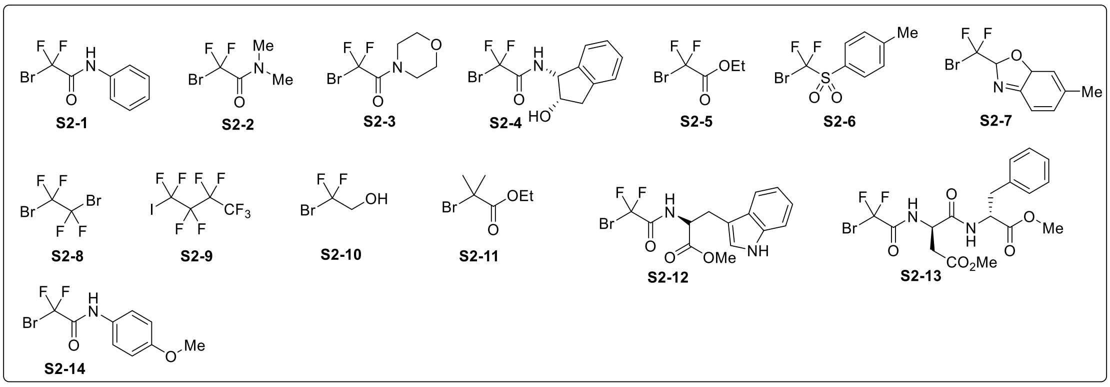

Supplementary Figure 2. Radical precursor substrates used in the reaction

S2-1, **S2-4**, and **S2-14** were prepared according to the literature.[11]

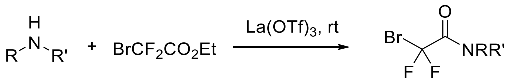

To a round-bottom flask equipped with stir bar was added amine (10.0 mmol, 1.0 equiv.) under argon, then ethyl bromodifluoroacetate (12mmol, 1.2 equiv.) was added with lanthanum trifluoromethanesulfonate (5 mol %). The mixture was stirred at the room temperature and monitored by TLC. After the amine was exhausted, the mixture was extracted with EA, and then the extract was washed with brine and dried over $MgSO_4$. The solvent was removed in vacuo and the residue was purified by column chromatography on silica gel to give the corresponding amide.

2-Bromo-2,2-difluoro-*N*-phenylacetamide (S2-1)

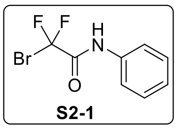

Synthesized as white solid (2.48 g, 99%). **^{1}H NMR** (400 MHz, $CDCl_3$) δ 7.80 (s, 1H), 7.67 – 7.52 (m, 2H), 7.47 – 7.40 (m, 2H), 7.26 – 7.19 (m, 1H). **^{19}F NMR** (376 MHz, $CDCl_3$) δ -60.68 (s, 2F). Spectral data are in accordance with the literature.[12]

2-Bromo-2,2-difluoro-*N*-((1*R*,2*S*)-2-hydroxy-2,3-dihydro-1*H*-inden-1-yl)acetamide (S2-4)

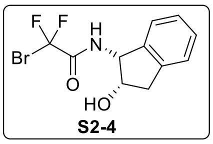

Synthesized as white solid (2.99 g, 98%). **^{1}H NMR** (400 MHz, $CDCl_3$) δ 7.34 – 7.20 (m, 4H), 7.08 (d, *J* = 8.4 Hz, 1H), 5.34 (dd, *J* = 8.5, 5.0 Hz, 1H), 4.66 (qd, *J* = 5.2, 1.8 Hz, 1H), 3.21 (dd, *J* = 16.7, 5.0 Hz, 1H), 2.96 (dd, *J* = 16.7, 1.8 Hz, 1H), 2.23 (d, *J* = 5.4 Hz, 1H). **^{19}F NMR** (376 MHz, $CDCl_3$) δ -60.58 (d, *J* = 2.3 Hz, 2F). Spectral data are in accordance with the literature.[11]

2-Bromo-2,2-difluoro-*N*-(4-methoxyphenyl)acetamide (S2-14)

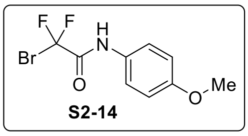

Synthesized as white solid (2.64 g, 94%). **^{1}H NMR** (400 MHz, $CDCl_3$) δ 7.77 (s, 1H), 7.48 (d, *J* = 9.0 Hz, 2H), 6.92 (d, *J* = 9.0 Hz, 2H), 3.82 (s, 3H). **^{19}F NMR** (376 MHz, $CDCl_3$) δ -60.45 (s, 2F). Spectral data are in accordance with the literature.[13]

S2-12 and **S2-13** were prepared according to the literature.[11]

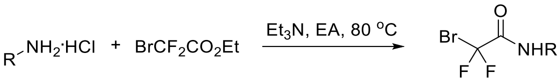

To a solution of amino acid hydrochloride (10.0 mmol, 1.0 equiv.) and triethylamine (20.0 mmol, 2.0 equiv.) in ethyl acetate (25 mL) were added $BrCF_2CO_2Et$ (10.0 mmol) at room temperature. The resulting mixture was stirred at room temperature for 2 h, and then was heated to 80 ^oC for 7 h. The reaction was cooled to room temperature and washed with water, extracted with EtOAc. The combined organic layers were dried over Na_2SO_4 and concentrated. The residue was purified with silica gel chromatography (EA:PE = 0-20%) to give compound.

Methyl (2-bromo-2,2-difluoroacetyl)-*D*-tryptophanate (S2-12)

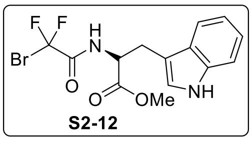

Synthesized as white solid (3.05 g, 81%). **^{1}H NMR** (400 MHz, $CDCl_3$) δ 8.16 (s, 1H), 7.52 (dd, *J* = 7.9, 1.1 Hz, 1H), 7.38 (dt, *J* = 8.2, 1.0 Hz, 1H), 7.24 – 7.19 (m, 1H), 7.17 – 7.11 (m, 1H), 7.01 (d, *J* = 2.4 Hz, 1H), 6.81 (d, *J* = 7.7 Hz, 1H), 4.93 (dt, *J* = 7.9, 5.1 Hz, 1H), 3.74 (s, 3H), 3.63 – 3.10 (m, 2H). ^{19}F NMR (376 MHz, $CDCl_3$) δ -60.71 (s, 2F). Spectral data are in accordance with the literature.[11]

Methyl (*R*)-3-(2-bromo-2,2-difluoroacetamido)-4-(((*R*)-1-methoxy-1-oxo-3-phenylpropan-2-yl)amino)-4-oxobutanoate (S2-13)

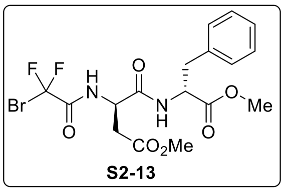

Synthesized as white solid (3.68 g, 79%). **^{1}H NMR** (400 MHz, $CDCl_3$) δ 7.65 (d, *J* = 7.4 Hz, 1H), 7.28 – 7.13 (m, 3H), 7.07 – 7.01 (m, 2H), 6.96 (d, *J* = 7.9 Hz, 1H), 4.72 (p, *J* = 5.9, 5.4 Hz, 2H), 3.64 (s, 3H), 3.62 (s, 3H), 3.16 – 2.97 (m, 2H), 2.94 – 2.81 (m, 1H), 2.73 – 2.48 (m, 1H). **^{19}F NMR** (376 MHz, $CDCl_3$) δ -60.79 (d, *J* = 161.4 Hz, 1F), -61.30 (d, *J* = 161.4 Hz, 1F). Spectral data are in accordance with the literature.[14]

1-((Bromodifluoromethyl)sulfonyl)-4-methylbenzene (S2-6)

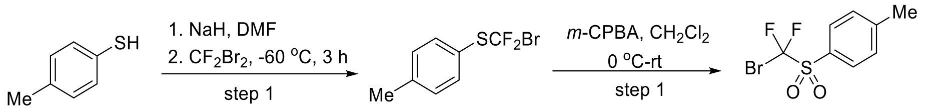

S2-6

step 1: Following a reported procedure[15], to a suspension of sodium hydride (360.0 mg, 15.0 mmol, 1.5 equiv.) in DMF (20 mL) was slowly added 4-methylbenzenethiol (1.24 g, 10.0 mmol, 1.0 equiv.) at 0 °C. After the addition was complete, the reaction mixture was warmed to room temperature for 30 min. The reaction mixture was brought to -60 °C for 15 min before dibromodifluoromethane (CF_2Br_2, 6.29 g, 30.0 mmol, 3.0 equiv.) was bubbled into the reaction mixture. The resulting mixture was maintained at -60 °C for 3 h. The reaction flask was cooled in ice-water bath and excess sodium hydride was destroyed by dropwise addition of water (10 mL). The aqueous phase was extracted with Et_2O (3×10 mL). The combined organic layers were washed with water (3×10 mL), brine (5 mL) and dried ($MgSO_4$). Filtration and solvent removal (aspirator, then vacuo) afforded a crude product. Purification by flash column chromatography (PE) on silica gel to give the (bromodifluoromethyl)(*p*-tolyl)sulfane as colorless liquid.

step 2: An oven-dried round-bottom flask with a magnetic stir-bar was charged with (bromodifluoromethyl)(*p*-tolyl)sulfane (1.27 g, 5.0 mmol, 1.0 equiv.) and dry CH_2Cl_2 (10 mL). After cooling to 0 °C, *m*-CPBA (2.59 g, 15 mmol, 3 equiv.) was added. The reaction mixture was stirred at room temperature for 24 h. The reaction mixture was concentrated, and dissolved with EtOAc (30 mL). The solution was washed with 10% NaOH (3×20 mL), saturated NaCl solution (20 mL), and dried over anhydrous $MgSO_4$. The combined organic material was dried in vacuo, and purified via silica gel column chromatography (EA:PE = 0-10%) to give **S2-6** as white solid. **^{1}H NMR** (400 MHz, $CDCl_3$) δ 7.91 (d, *J* = 8.3 Hz, 2H), 7.45 (d, *J* = 8.2 Hz, 2H), 2.51 (s, 3H). **^{19}F NMR** (376 MHz, $CDCl_3$) δ -57.73 (s, 2F). Spectral data are in accordance with the literature.[15]

4.3 Alkyl bromides

Substrates **S3-(1-19)** were purchased from commercial sources and used as received.

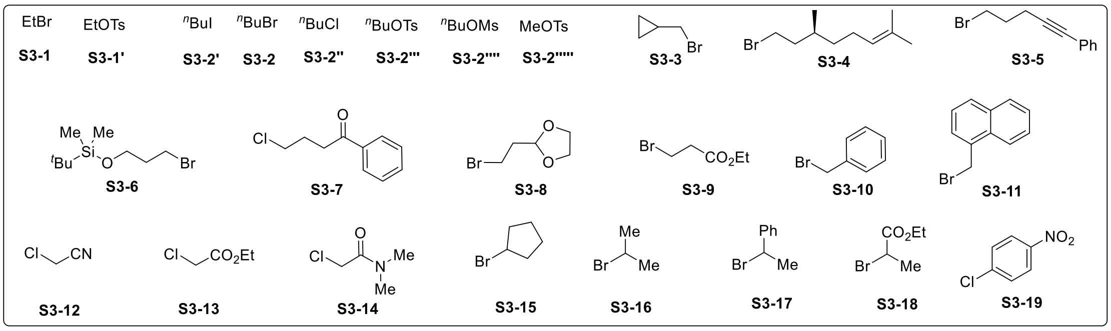

Supplementary Figure 3. Alkyl bromide substrates used in the reaction

5. Experimental Characterization Data for Products

4-(Ethylsulfonyl)-2,2-difluoro-*N*-phenyldecanamide (1)

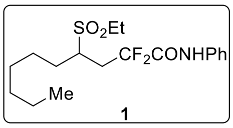

The title compound was prepared according to the ***General procedure I*** and purified by column chromatography (EA:PE = 0-10%) as a yellow oil (122.4 mg, 81% yield). **^{1}H NMR** (500 MHz, $CDCl_3$) δ 8.31 (s, 1H), 7.57 (d, *J* = 8.7 Hz, 2H), 7.35 (t, *J* = 8.0 Hz, 2H), 7.19 (t, *J* = 7.4 Hz, 1H), 3.39 – 3.22 (m, 1H), 3.03 (q, *J* = 7.5 Hz, 2H), 2.98 – 2.81 (m, 1H), 2.59 – 2.42 (m, 1H), 2.09 – 1.97 (m, 1H), 1.83 – 1.69 (m, 1H), 1.59 – 1.42 (m, 2H), 1.38 (t, *J* = 7.4 Hz, 3H), 1.33 – 1.13 (m, 6H), 0.86 (t, *J* = 5.0 Hz, 3H). **^{13}C NMR** (126 MHz, $CDCl_3$) δ 161.21 (t, $^2J_{C-F}$ = 28.3 Hz), 135.85, 129.27, 125.91, 120.54, 116.64 (t, $^1J_{C-F}$ = 255.7 Hz), 58.38 (t, $^3J_{C-F}$ = 2.5 Hz), 45.15, 32.71 (t, $^2J_{C-F}$ = 23.9 Hz), 31.47, 29.20, 28.99, 26.30, 22.59, 14.08, 6.09. **^{19}F NMR** (376 MHz, $CDCl_3$) δ -102.37 (d, *J* = 256.2 Hz, 1F), -104.62 (d, *J* = 256.2 Hz, 1F). **HRMS** (ESI) calcd for $C_{18}H_{27}F_2NO_3SNa$ [M + Na]$^+$: 398.1572, found: 398.1579.

5-Cyclohexyl-4-(ethylsulfonyl)-2,2-difluoro-*N*-phenylpentanamide (2)

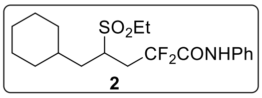

The title compound was prepared according to the ***General procedure I*** and purified by column chromatography (EA:PE = 0-10%) as a light yellow oil (131.4 mg, 85% yield). **^{1}H NMR** (400 MHz, $CDCl_3$) δ 8.29 (s, 1H), 7.56 (d, *J* = 9.5 Hz, 2H), 7.35 (t, *J* = 8.0 Hz, 2H), 7.18 (t, *J* = 8.0 Hz, 1H), 3.42 – 3.34 (m, 1H), 3.02 (q, *J* = 7.4 Hz, 2H), 2.96 – 2.81 (m, 1H), 2.55 – 2.24 (m, 1H), 2.03 – 1.83 (m, 1H), 1.83 – 1.59 (m, 5H), 1.58 – 1.43 (m, 2H), 1.37 (t, *J* = 7.5 Hz, 3H), 1.29 – 1.02 (m, 3H), 1.03 – 0.74 (m, 2H). **^{13}C NMR** (101 MHz, $CDCl_3$) δ 161.26 (t, $^2J_{C-F}$ = 28.4 Hz), 135.91, 129.24, 125.87, 120.60, 116.60 (t, $^1J_{C-F}$ = 255.6 Hz), 53.07, 44.85, 37.02, 34.69, 33.82 (t, $^2J_{C-F}$ = 24.2 Hz), 33.55, 32.62, 26.35, 26.10, 25.89, 6.01. **^{19}F NMR** (376 MHz, $CDCl_3$) δ -101.83 (d, *J* = 256.3 Hz, 1F), -104.14 (d, *J* = 256.5 Hz, 1F). **HRMS** (ESI) calcd for $C_{19}H_{27}F_2NO_3SNa$ [M + Na]$^+$: 410.1572, found: 410.1578.

4-(Ethylsulfonyl)-2,2-difluoro-5,5-dimethyl-*N*-phenylhexanamide (3)

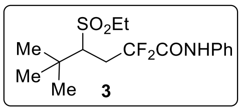

The title compound was prepared according to the ***General procedure I*** and purified by column chromatography (EA:PE = 0-20%) as a colorless oil (83.1 mg, 60% yield). **^{1}H NMR** (500 MHz, $CDCl_3$) δ 8.09 (s, 1H), 7.55 (d, *J* = 7.8 Hz, 2H), 7.36 (t, *J* = 7.9 Hz, 2H), 7.20 (t, *J* = 7.4 Hz, 1H), 3.32 (dd, *J* = 6.0, 2.7 Hz, 1H), 3.12 (q, *J* = 7.4 Hz, 2H), 3.09 – 2.96 (m, 1H), 2.70 – 2.52 (m, 1H), 1.40 (t, *J* = 7.4 Hz, 3H), 1.23 (s, 9H). **^{13}C NMR** (126 MHz, $CDCl_3$) δ 161.72 (t, $^2J_{C-F}$ = 28.5 Hz), 135.95, 129.33, 125.90, 120.61, 116.35 (t, $^1J_{C-F}$ = 254.5 Hz), 64.37, 50.11, 35.66, 30.98 (t, $^2J_{C-F}$ = 23.9 Hz), 28.32, 6.20. **^{19}F NMR** (471 MHz, $CDCl_3$) δ -103.04 (d, *J* = 250.3 Hz, 1F), -104.62 (d, *J* = 250.6 Hz, 1F). **HRMS** (ESI) calcd for $C_{16}H_{23}F_2NO_3SNa$ [M + Na]$^+$: 370.1259, found: 370.1268.

4-Cyclohexyl-4-(ethylsulfonyl)-2,2-difluoro-*N*-phenylbutanamide (4)

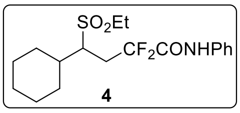

The title compound was prepared according to the ***General procedure I*** and purified by column chromatography (EA:PE = 0-20%) as a light yellow oil (129.6 mg, 87% yield). **^{1}H NMR** (400 MHz, $CDCl_3$) δ 8.31 (s, 1H), 7.56 (d, *J* = 8.7 Hz, 2H), 7.35 (t, *J* = 8.0 Hz, 2H), 7.19 (t, *J* = 7.4 Hz, 1H), 3.27 – 3.19 (m, 1H), 3.12 – 2.98 (m, 2H), 2.98 – 2.79 (m, 1H), 2.75 – 2.52 (m, 1H), 2.36 – 2.18 (m, 1H), 1.92 (s, 1H), 1.83 – 1.70 (m, 2H), 1.70 – 1.54 (m, 2H), 1.36 (t, *J* = 7.4 Hz, 3H), 1.33 – 1.00 (m, 5H). **^{13}C NMR** (126 MHz, $CDCl_3$) δ 161.34 (t, $^2J_{C-F}$ = 28.4 Hz), 135.87, 129.25, 125.89, 120.56, 116.61 (t, $^1J_{C-F}$ = 255.2 Hz), 59.33, 46.99, 37.66, 30.71, 29.76 (t, $^2J_{C-F}$ = 23.9 Hz), 27.95, 26.58, 25.97, 25.81, 6.29. **^{19}F NMR** (471 MHz, $CDCl_3$) δ -102.77 (d, *J* = 253.8 Hz), -103.61 (d, *J* = 253.6 Hz). **HRMS** (ESI) calcd for $C_{18}H_{25}F_2NO_3SNa$ $[M + Na]^+$: 396.1415, found: 396.1419.

4-(Ethylsulfonyl)-2,2-difluoro-*N*-phenyldec-9-enamide (5)

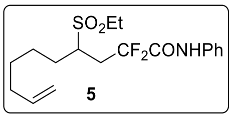

The title compound was prepared according to the ***General procedure I*** and purified by column chromatography (EA:PE = 0-10%) as a light yellow oil (121.1 mg, 81% yield). **^{1}H NMR** (500 MHz, $CDCl_3$) δ 8.25 (s, 1H), 7.56 (d, *J* = 7.8 Hz, 2H), 7.36 (t, *J* = 7.9 Hz, 2H), 7.20 (t, *J* = 7.4 Hz, 1H), 5.89 – 5.70 (m, 1H), 5.21 – 4.46 (m, 2H), 3.44 – 3.16 (m, 1H), 3.03 (q, *J* = 7.5 Hz, 2H), 2.96 – 2.81 (m, 1H), 2.64 – 2.39 (m, 1H), 2.11 – 1.96 (m, 3H), 1.84 – 1.72 (m, 1H), 1.64 – 1.46 (m, 2H), 1.46 – 1.32 (m, 5H). **^{13}C NMR** (126 MHz, $CDCl_3$) δ 161.17 (t, $^2J_{C-F}$ = 28.3 Hz), 138.39, 135.81, 129.31, 125.96, 120.53, 116.66 (t, $^1J_{C-F}$ = 255.7 Hz), 114.90, 55.31 (t, $^3J_{C-F}$ = 2.5 Hz), 45.20, 33.34, 32.74 (t, $^2J_{C-F}$ = 23.9 Hz), 28.81, 28.71, 25.78, 6.13. **^{19}F NMR** (471 MHz, $CDCl_3$) δ -102.23 (d, *J* = 256.7 Hz, 1F), -104.62 (d, *J* = 256.7 Hz, 1F). **HRMS** (ESI) calcd for $C_{18}H_{25}F_2NO_3SNa$ $[M + Na]^+$: 396.1415, found: 396.1422.

Dimethyl 2-(4-(ethylsulfonyl)-6,6-difluoro-7-oxo-7-(phenylamino)heptyl)malonate (6)

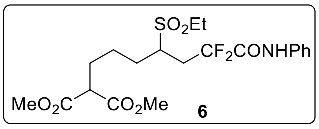

The title compound was prepared according to the ***General procedure I*** and purified by column chromatography (EA:PE = 0-10%) as a light yellow oil (138.7 mg, 75% yield). **^{1}H NMR** (400 MHz, $CDCl_3$) δ 8.41 (s, 1H), 7.57 (d, *J* = 7.9 Hz, 2H), 7.35 (t, *J* = 7.9 Hz, 2H), 7.18 (t, *J* = 7.4 Hz, 1H), 3.71 (t, *J* = 1.5 Hz, 6H), 3.43 – 3.33 (m, 1H), 3.32 – 3.22 (m, 1H), 3.09 – 2.98 (m, 2H), 2.97 – 2.77 (m, 1H), 2.57 – 2.36 (m, 1H), 2.15 – 1.98 (m, 1H), 1.97 – 1.85 (m, 2H), 1.85 – 1.72 (m, 1H), 1.67 – 1.46 (m, 2H), 1.43 – 1.31 (m, 3H). **^{13}C NMR** (101 MHz, $CDCl_3$) δ 169.74, 169.69, 161.10 (t, $^2J_{C-F}$ = 28.3 Hz), 135.88, 129.28, 125.92, 120.56, 116.65 (t, $^1J_{C-F}$ = 255.8 Hz), 54.84, 52.70, 51.16, 45.24, 32.81 (t, $^2J_{C-F}$ = 23.9 Hz), 28.44, 28.27, 23.96, 6.13. **^{19}F NMR** (376 MHz, $CDCl_3$) δ -101.87 (ddq, *J* = 256.9, 11.4, 3.1 Hz, 1F), -104.95 (d, *J* = 257.0 Hz, 1F). **HRMS** (ESI) calcd for

$C_{20}H_{27}F_2NO_7SNa$ [M + Na]$^+$: 486.1369, found: 486.1377.

4-(Ethylsulfonyl)-2,2-difluoro-7-oxo-*N*-phenyloctanamide (7)

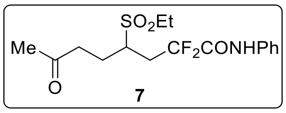

The title compound was prepared according to the ***General procedure I*** and purified by column chromatography (EA:PE = 0-20%) as a light yellow oil (104.9 mg, 73% yield). **^{1}H NMR** (400 MHz, $CDCl_3$) δ 8.63 (s, 1H), 7.56 (d, *J* = 7.7 Hz, 2H), 7.32 (t, *J* = 7.9 Hz, 2H), 7.15 (t, *J* = 7.9 Hz, 1H), 3.41 – 3.29 (m, 1H), 3.12 – 2.97 (m, 2H), 2.94 – 2.61 (m, 3H), 2.50 – 2.30 (m, 1H), 2.26 – 2.17 (m, 1H), 2.07 – 1.97 (m, 4H), 1.33 (t, *J* = 7.5 Hz, 3H). **^{13}C NMR** (101 MHz, $CDCl_3$) δ 207.69, 161.17 (t, $^2J_{C-F}$ = 28.3 Hz), 135.95, 129.15, 125.81, 120.64, 116.60 (t, $^1J_{C-F}$ = 256.5 Hz), 53.75, 45.03, 39.19, 32.39 (t, $^2J_{C-F}$ = 24.1 Hz), 29.92, 22.75, 6.00. **^{19}F NMR** (376 MHz, $CDCl_3$) δ -101.65 (d, *J* = 257.0 Hz, 1F), -104.85 (d, *J* = 256.9 Hz, 1F). **HRMS** (ESI) calcd for $C_{16}H_{21}F_2NO_4SNa$ [M + Na]$^+$: 384.1052, found: 384.1060.

4-(Ethylsulfonyl)-2,2-difluoro-*N*-phenyl-5-(trimethylsilyl)pentanamide (8)

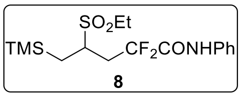

The title compound was prepared according to the ***General procedure I*** and purified by column chromatography (EA:PE = 0-10%) as a yellow oil (102.1 mg, 67% yield). **^{1}H NMR** (400 MHz, $CDCl_3$) δ 8.28 (s, 1H), 7.55 (d, *J* = 8.4 Hz, 2H), 7.34 (t, *J* = 8.0 Hz, 2H), 7.22 – 7.10 (m, 1H), 3.53 – 3.32 (m, 1H), 3.12 – 3.00 (m, 2H), 2.99 – 2.81 (m, 1H), 2.58 – 2.35 (m, 1H), 1.37 (t, *J* = 7.5 Hz, 3H), 1.31 (d, *J* = 4.2 Hz, 1H), 1.04 – 0.94 (m, 1H), 0.12 (s, 9H). **^{13}C NMR** (101 MHz, $CDCl_3$) δ 162.23 (t, $^2J_{C-F}$ = 28.1 Hz), 136.92, 130.25, 126.86, 121.63, 117.51 (t, $^1J_{C-F}$ = 256.5 Hz), 54.41 (t, $^3J_{C-F}$ = 3.0 Hz), 44.81, 36.26 (t, $^2J_{C-F}$ = 23.8 Hz), 18.07, 7.04, 0.06. **^{19}F NMR** (376 MHz, $CDCl_3$) δ -100.44 (d, *J* = 257.1 Hz, 1F), -103.99 (d, *J* = 257.2 Hz, 1F). **HRMS** (ESI) calcd for $C_{16}H_{25}F_2NO_3SSiNa$ [M + Na]$^+$: 400.1185, found: 400.1189.

4-(Ethylsulfonyl)-2,2-difluoro-5-hydroxy-*N*-phenylpentanamide (9).

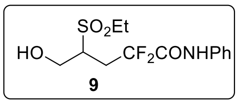

The title compound was prepared according to the ***General procedure I*** and purified by column chromatography (EA:PE = 0-50%) as a light yellow oil (119.7 mg, 93% yield). **^{1}H NMR** (400 MHz, $CDCl_3$) δ 8.30 (s, 1H), 7.55 (d, *J* = 8.0 Hz, 2H), 7.36 (t, *J* = 7.7 Hz, 2H), 7.26 – 7.10 (m, 1H), 4.27 – 4.17 (m, 1H), 4.11 – 3.99 (m, 1H), 3.54 – 3.39 (m, 1H), 3.27 – 3.10 (m, 2H), 3.01 – 2.72 (m, 2H), 2.74 – 2.56 (m, 1H), 1.39 (t, *J* = 7.4 Hz, 3H). **^{13}C NMR** (101 MHz, $CDCl_3$) δ 161.28 (t, $^2J_{C-F}$ = 28.4 Hz), 135.72, 129.38, 126.13, 120.69, 116.74 (t, $^1J_{C-F}$ = 258.2 Hz), 60.01, 57.82, 47.37, 28.83 (t, $^2J_{C-F}$ = 24.1 Hz), 5.97. **^{19}F NMR** (376 MHz, $CDCl_3$) δ -102.85 (d, *J* = 258.6 Hz, 1F), -104.17 (d, *J* = 258.7 Hz, 1F). **HRMS** (ESI) calcd for $C_{13}H_{18}F_2NO_4S$ [M + H]$^+$: 322.0919, found: 322.0913.

4-(Ethylsulfonyl)-2,2-difluoro-*N*,5-diphenylpentanamide (10)

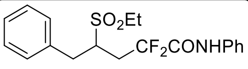

10

The title compound was prepared according to the ***General procedure I*** and purified by column chromatography (EA:PE = 0-20%) as a light yellow oil (131.1 mg, 86% yield). **^{1}H NMR** (400 MHz, $CDCl_3$) δ 8.17 (s, 1H), 7.56 – 7.44 (m, 2H), 7.34 (t, *J* = 7.9 Hz, 2H), 7.32 – 7.26 (m, 4H), 7.27 – 7.22 (m, 1H), 7.21 – 7.15 (m, 1H), 3.77 – 3.61 (m, 1H), 3.43 – 3.14 (m, 2H), 3.12 – 2.94 (m, 1H), 2.78 – 2.57 (m, 2H), 2.58 – 2.35 (m, 1H), 1.22 (t, *J* = 7.4 Hz, 3H). **^{13}C NMR** (126 MHz, $CDCl_3$) δ 161.14 (t, $^{2}J_{C-F}$ = 28.2 Hz), 136.56, 135.82, 129.45, 129.25, 128.99, 127.51, 125.90, 120.58, 116.60 (t, $^{1}J_{C-F}$ = 255.8 Hz), 57.13 (t, $^{3}J_{C-F}$ = 2.5 Hz), 46.94, 35.57, 32.21 (t, $^{2}J_{C-F}$ = 24.2 Hz), 6.12. **^{19}F NMR** (471 MHz, $CDCl_3$) δ -101.67 (d, *J* = 256.3 Hz, 1F), -103.71 (d, *J* = 256.0 Hz, 1F). **HRMS** (ESI) calcd for $C_{19}H_{21}F_2NO_3SNa$ $[M + Na]^+$: 404.1102, found: 404.1104.

4-(Ethylsulfonyl)-2,2-difluoro-6-(naphthalen-1-yl)-*N*-phenylhexanamide (11)

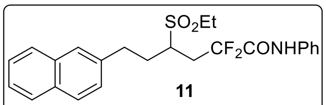

11

The title compound was prepared according to the ***General procedure I*** and purified by column chromatography (EA:PE = 0-20%) as a yellow oil (165.7 mg, 93% yield). **^{1}H NMR** (400 MHz, $CDCl_3$) δ 8.00 (s, 1H), 7.76 (td, *J* = 7.9, 7.3, 2.7 Hz, 3H), 7.65 (s, 1H), 7.50 – 7.39 (m, 4H), 7.35 (td, *J* = 8.7, 8.1, 1.9 Hz, 3H), 7.21 (t, *J* = 7.4 Hz, 1H), 3.41 – 3.30 (m, 1H), 3.26 – 2.83 (m, 5H), 2.69 – 2.41 (m, 2H), 2.29 – 2.15 (m, 1H), 1.34 (t, *J* = 7.5 Hz, 3H). **^{13}C NMR** (101 MHz, $CDCl_3$) δ 161.0 (t, $^{2}J_{C-F}$ = 27.3 Hz), 137.71, 135.63, 133.63, 132.26, 129.39, 128.40, 127.76, 127.59, 127.12, 127.02, 126.28, 126.06, 125.65, 120.47, 108.28 (t, $^{1}J_{C-F}$ = 176.8 Hz), 54.26, 45.35, 33.09 (t, $^{2}J_{C-F}$ = 23.9 Hz), 32.71, 30.68, 6.25. **^{19}F NMR** (376 MHz, $CDCl_3$) δ -100.89 (d, *J* = 258.2 Hz, 1F), -104.69 (d, *J* = 258.2 Hz, 1F). **HRMS** (ESI) calcd for $C_{24}H_{25}F_2NO_3SNa$ $[M + Na]^+$: 468.1415, found: 468.1424.

4-(Ethylsulfonyl)-2,2-difluoro-*N*-phenyl-7-(quinolin-2-yl)heptanamide (12)

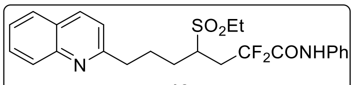

12

The title compound was prepared according to the ***General procedure I*** and purified by column chromatography (EA:PE = 0-20%) as a yellow oil (169.5 mg, 92% yield). **^{1}H NMR** (400 MHz, $CDCl_3$) δ 8.95 (s, 1H), 8.09 (d, *J* = 8.5 Hz, 1H), 8.03 – 7.96 (m, 1H), 7.79 (dd, *J* = 8.2, 1.4 Hz, 1H), 7.73 – 7.65 (m, 1H), 7.64 – 7.57 (m, 2H), 7.56 – 7.47 (m, 1H), 7.38 – 7.27 (m, 3H), 7.18 (td, *J* = 7.4, 1.2 Hz, 1H), 3.39 – 3.27 (m, 1H), 3.19 – 2.95 (m, 4H), 2.97 – 2.82 (m, 1H), 2.60 – 2.39 (m, 1H), 2.26 – 2.12 (m, 2H), 2.12 – 1.97 (m, 1H), 2.00 – 1.81 (m, 1H), 1.36 (t, *J* = 7.4 Hz, 3H). **^{13}C NMR** (101 MHz, $CDCl_3$) δ 161.47, 161.40 (t, $^{2}J_{C-F}$ = 28.2 Hz), 147.74, 136.86, 136.10, 129.78, 129.35, 128.52, 127.77, 126.97, 126.13, 125.91, 121.52, 120.55, 116.82 (t, $^{1}J_{C-F}$ = 258.6 Hz), 54.87, 45.36, 37.89, 33.14 (t, $^{2}J_{C-F}$ = 24.4 Hz), 27.85, 26.13, 6.23. **^{19}F NMR** (376 MHz, $CDCl_3$) δ -100.73

(ddd, J = 255.6, 11.3, 6.5 Hz, 1F), -104.85 (d, J = 255.6 Hz, 1F). **HRMS** (ESI) calcd for $C_{24}H_{26}F_2N_2O_3SNa$ [M + Na]$^+$: 483.1524, found: 483.1533.

5-(3,4-Dimethoxyphenyl)-4-(ethylsulfonyl)-2,2-difluoro-*N*-phenylpentanamide (13)

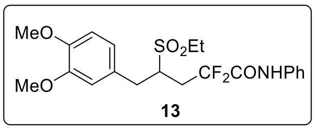

The title compound was prepared according to the ***General procedure I*** and purified by column chromatography (EA:PE = 0-30%) as a yellow oil (139.5 mg, 79% yield). **^{1}H NMR** (400 MHz, $CDCl_3$) δ 8.18 (s, 1H), 7.52 (d, J = 7.9 Hz, 2H), 7.33 (t, J = 7.9 Hz, 2H), 7.17 (t, J = 7.4 Hz, 1H), 6.85 – 6.68 (m, 3H), 3.82 (s, 3H), 3.80 (s, 3H), 3.75 – 3.59 (m, 1H), 3.30 – 3.10 (m, 2H), 3.09 – 2.92 (m, 1H), 2.83 – 2.59 (m, 2H), 2.58 – 2.40 (m, 1H), 1.23 (t, J = 7.4 Hz, 3H). **^{13}C NMR** (101 MHz, $CDCl_3$) δ 161.14 (t, $^2J_{C\text{-}F}$ = 28.3 Hz), 149.23, 148.35, 135.86, 129.23, 128.79, 125.86, 121.69, 120.49, 116.62 (t, $^1J_{C\text{-}F}$ = 255.8 Hz), 112.56, 111.41, 57.21 (t, $^3J_{C\text{-}F}$ = 2.1 Hz), 55.96, 55.85, 46.93, 35.05, 32.22 (t, $^2J_{C\text{-}F}$ = 24.3 Hz), 6.12. **^{19}F NMR** (376 MHz, $CDCl_3$) δ -101.51 (d, J = 255.7 Hz, 1F), -103.35 (d, J = 256.0 Hz, 1F). **HRMS** (ESI) calcd for $C_{21}H_{25}F_2NO_5SNa$ [M + Na]$^+$: 464.1314, found: 464.1318.

4-(Ethylsulfonyl)-2,2-difluoro-5-(2-methyl-1*H*-indol-1-yl)-*N*-phenylpentanamide (14)

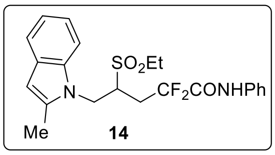

The title compound was prepared according to the ***General procedure I*** and purified by column chromatography (EA:PE = 0-30%) as a yellow oil (146.0 mg, 84% yield). **^{1}H NMR** (400 MHz, $CDCl_3$) δ 8.07 (s, 1H), 7.55 (d, J = 8.0 Hz, 2H), 7.51 (d, J = 7.6 Hz, 1H), 7.43 – 7.33 (m, 3H), 7.27 – 7.20 (m, 1H), 7.16 (t, J = 7.5 Hz, 1H), 7.10 (t, J = 7.4 Hz, 1H), 6.27 (s, 1H), 4.77 – 4.56 (m, 2H), 4.38 – 4.17 (m, 1H), 3.28 – 3.11 (m, 1H), 2.52 (s, 3H), 2.50 – 2.34 (m, 1H), 2.27 – 1.97 (m, 2H), 0.93 (t, J = 7.4 Hz, 3H). **^{13}C NMR** (101 MHz, $CDCl_3$) δ 160.89 (t, $^2J_{C\text{-}F}$ = 28.1 Hz), 136.90, 136.21, 135.66, 129.41, 128.49, 126.13, 121.76, 120.60, 120.49, 120.40, 116.67 (t, $^1J_{C\text{-}F}$ = 256.0 Hz), 109.22, 101.96, 53.09, 47.57, 43.99, 29.82 (t, $^2J_{C\text{-}F}$ = 24.4 Hz), 12.84, 6.21. **^{19}F NMR** (376 MHz, $CDCl_3$) δ -101.91 (d, J = 254.7 Hz, 1F), -104.28 (d, J = 254.8 Hz, 1F). **HRMS** (ESI) calcd for $C_{22}H_{24}F_2N_2O_3SNa$ [M + Na]$^+$: 457.1368, found: 457.1369.

5-(9*H*-Carbazol-9-yl)-4-(ethylsulfonyl)-2,2-difluoro-*N*-phenylpentanamide (15)

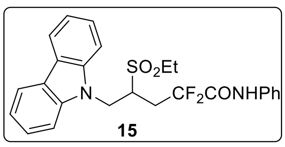

The title compound was prepared according to the ***General procedure I*** and purified by column chromatography (EA:PE = 0-20%) as a yellow oil (129.9 mg, 69% yield). **^{1}H NMR** (400 MHz, DMSO-d_6) δ 10.70 (s, 1H), 8.30 – 8.02 (m, 2H), 7.80 – 7.61 (m, 4H), 7.56 – 7.45 (m, 2H), 7.42 – 7.32 (m, 2H), 7.32 – 7.20 (m, 2H), 7.22 – 7.14 (m, 1H), 5.20 – 4.62 (m, 2H), 4.24 – 4.08 (m, 1H), 3.29 – 3.03 (m, 1H), 2.92

– 2.58 (m, 3H), 0.95 (t, J = 7.4 Hz, 3H). **^{13}C NMR** (126 MHz, DMSO-d_6) δ 161.08 (t, $^2J_{C-F}$ = 29.0 Hz), 139.88, 136.96, 128.75, 126.07, 125.04, 122.53, 121.00, 120.48, 119.62, 116.52 (t, $^1J_{C-F}$ = 254.8 Hz), 109.41, 52.97, 45.69, 43.09, 30.19 (t, $^2J_{C-F}$ = 24.5 Hz), 5.36. **^{19}F NMR** (376 MHz, DMSO-d_6) δ -100.83 (d, J = 253.7 Hz, 1F), -102.68 (d, J = 253.4 Hz, 1F). **HRMS** (ESI) calcd for $C_{25}H_{24}F_2N_2O_3SNa$ [M + Na]$^+$: 493.1368, found: 493.1373.

4-(Ethylsulfonyl)-2,2-difluoro-5-(10*H*-phenothiazin-10-yl)-*N*-phenylpentanamide (16)

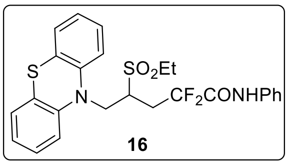

The title compound was prepared according to the ***General procedure I*** and purified by column chromatography (EA:PE = 0-20%) as a yellow oil (134.7 mg, 67% yield). **^{1}H NMR** (500 MHz, $CDCl_3$) δ 8.17 (s, 1H), 7.66 – 7.51 (m, 2H), 7.38 (dd, J = 8.6, 7.3 Hz, 2H), 7.24 – 7.15 (m, 5H), 7.10 – 6.93 (m, 4H), 4.68 – 4.56 (m, 1H), 4.30 (dd, J = 14.3, 4.5 Hz, 1H), 4.03 – 3.92 (m, 1H), 3.47 – 3.27 (m, 2H), 3.23 – 3.04 (m, 1H), 2.81 – 2.47 (m, 1H), 1.16 (t, J = 7.4 Hz, 3H). **^{13}C NMR** (126 MHz, $CDCl_3$) δ 160.94 (t, $^2J_{C-F}$ = 28.3 Hz), 144.31, 135.79, 129.40, 128.26, 128.01, 126.03, 126.01, 123.89, 120.47, 116.84 (t, $^1J_{C-F}$ = 255.7 Hz), 115.86, 51.65, 48.95, 46.84, 30.57 (t, $^2J_{C-F}$ = 24.3 Hz), 6.48. **^{19}F NMR** (471 MHz, $CDCl_3$) δ -102.40 (d, J = 252.5 Hz, 1F), -104.22 (d, J = 252.6 Hz, 1F). **HRMS** (ESI) calcd for $C_{25}H_{24}F_2N_2O_3S_2Na$ [M + Na]$^+$: 525.1089, found: 525.1090.

5-(1,3-Dioxoisoindolin-2-yl)-4-(ethylsulfonyl)-2,2-difluoro-*N*-phenylpentanamide (17)

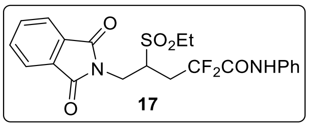

The title compound was prepared according to the ***General procedure I*** and purified by column chromatography (EA:PE = 0-20%) as a colorless oil (133.3 mg, 74% yield). **^{1}H NMR** (400 MHz, $CDCl_3$) δ 8.39 (d, J = 12.5 Hz, 1H), 7.87 – 7.72 (m, 2H), 7.72 – 7.63 (m, 2H), 7.52 – 7.39 (m, 2H), 7.27 (td, J = 7.9, 1.9 Hz, 2H), 7.21 – 7.06 (m, 1H), 4.32 – 4.19 (m, 2H), 3.98 – 3.80 (m, 1H), 3.35 – 2.95 (m, 3H), 2.78 – 2.51 (m, 1H), 1.41 (td, J = 7.4, 1.9 Hz, 3H). **^{13}C NMR** (126 MHz, $CDCl_3$) δ 168.11, 160.85 (t, $^2J_{C-F}$ = 28.2 Hz), 135.86, 134.34, 131.67, 129.10, 125.69, 123.59, 120.44, 116.18 (t, $^1J_{C-F}$ = 256.1 Hz), 52.86 (t, $^3J_{C-F}$ = 3.4 Hz), 45.60, 37.23, 31.55 (t, $^2J_{C-F}$ = 24.8 Hz), 5.87. **^{19}F NMR** (376 MHz, $CDCl_3$) δ -102.85 (d, J = 257.5 Hz, 1F), -103.88 (d, J = 257.3 Hz, 1F). **HRMS** (ESI) calcd for $C_{21}H_{20}F_2N_2O_5SNa$ [M + Na]$^+$: 473.0953, found: 473.0956.

4-(Ethylsulfonyl)-2,2-difluoro-4-(4-methoxyphenyl)-*N*-phenylbutanamide (18)

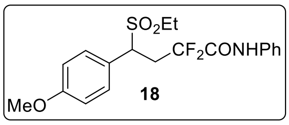

The title compound was prepared according to the ***General procedure I*** and purified by column chromatography (EA:PE = 0-30%) as a white solid (73.4 mg, 46% yield). **^{1}H NMR** (400 MHz, $CDCl_3$) δ 7.75 (s, 1H), 7.47 – 7.27 (m, 6H), 7.17 (t, J = 7.8 Hz, 1H), 6.85 (d, J = 8.8 Hz, 2H), 4.41 (dd, J = 11.2, 3.0

Hz, 1H), 3.71 (s, 3H), 3.31 – 2.99 (m, 2H), 2.84 – 2.62 (m, 2H), 1.27 (t, J = 7.5 Hz, 3H). **^{13}C NMR** (101 MHz, $CDCl_3$) δ 161.02 (t, $^2J_{C-F}$ = 27.8 Hz), 160.59, 135.73, 130.85, 129.20, 125.80, 123.24, 120.30, 116.79 (t, $^1J_{C-F}$ = 256.9 Hz), 114.70, 61.57 (t, $^3J_{C-F}$ = 3.8 Hz), 55.36, 44.77, 32.33 (t, $^2J_{C-F}$ = 24.5 Hz), 6.23. **^{19}F NMR** (376 MHz, $CDCl_3$) δδ -101.09 (d, J = 256.1 Hz, 1F), -104.54 (d, J = 256.1 Hz, 1F). **HRMS** (ESI) calcd for $C_{19}H_{22}F_2NO_4S$ [M + H]$^+$: 398.1232, found: 398.1230.

4-(4-(*tert*-Butyl)phenyl)-4-(ethylsulfonyl)-2,2-difluoro-*N*-phenylbutanamide (19)

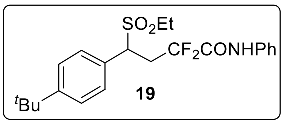

The title compound was prepared according to the ***General procedure I*** and purified by column chromatography as (EA:PE = 0-10%) a white solid (62.8 mg, 37% yield). **^{1}H NMR** (400 MHz, $CDCl_3$) δ 7.73 (s, 1H), 7.41 – 7.27 (m, 8H), 7.20 – 7.09 (m, 1H), 4.42 (dd, J = 11.0, 3.1 Hz, 1H), 3.34 – 3.07 (m, 2H), 2.83 – 2.62 (m, 2H), 1.28 (t, J = 7.5 Hz, 3H), 1.22 (s, 9H). **^{13}C NMR** (101 MHz, $CDCl_3$) δ 160.84 (t, $^2J_{C-F}$ = 27.6 Hz), 152.97, 135.75, 129.29, 129.26, 128.45, 126.24, 125.82, 120.30, 116.80 (t, $^1J_{C-F}$ = 257.6 Hz), 63.01 (t, $^3J_{C-F}$ = 4.0 Hz), 44.86, 34.77, 32.36 (t, $^2J_{C-F}$ = 24.7 Hz), 31.25, 6.22. **^{19}F NMR** (376 MHz, $CDCl_3$) δ -100.57 (d, J = 256.1 Hz, 1F), -105.06 (d, J = 256.1 Hz, 1F). **HRMS** (ESI) calcd for $C_{22}H_{27}F_2NO_3SNa$ [M + Na]$^+$: 446.1572, found: 446.1576.

4-(Ethylsulfonyl)-2,2-difluoro-*N*,4-diphenylbutanamide (20)

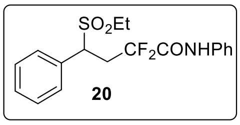

The title compound was prepared according to the ***General procedure I*** and purified by column chromatography (EA:PE = 0-10%) as a white solid (42.5 mg, 29% yield). **^{1}H NMR** (400 MHz, $CDCl_3$) δ 7.81 (s, 1H), 7.51 – 7.44 (m, 2H), 7.43 – 7.29 (m, 7H), 7.21 – 7.15 (m, 1H), 4.48 (dd, J = 11.0, 2.9 Hz, 1H), 3.37 – 3.20 (m, 1H), 3.18 – 3.01 (m, 1H), 2.81 – 2.60 (m, 2H), 1.27 (t, J = 7.5 Hz, 3H). **^{13}C NMR** (101 MHz, $CDCl_3$) δ 160.85 (t, $^2J_{C-F}$ = 27.9 Hz), 135.66, 131.98, 129.71, 129.58, 129.31, 129.26, 125.90, 120.40, 116.72 (t, $^1J_{C-F}$ = 256.5 Hz), 62.17, 44.98, 32.46 (t, $^2J_{C-F}$ = 24.3 Hz), 6.22. **^{19}F NMR** (376 MHz, $CDCl_3$) δ -102.08 (d, J = 256.7 Hz, 1F), -103.49 (d, J = 256.7 Hz, 1F). **HRMS** (ESI) calcd for $C_{18}H_{19}F_2NNaO_3S$ [M + Na]$^+$: 390.0946, found: 390.0950.

4-(Ethylsulfonyl)-2,2-difluoro-4-isopropoxy-*N*-phenylbutanamide (21)

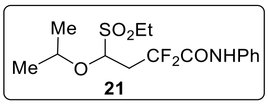

The title compound was prepared according to the ***General procedure I*** and purified by column chromatography (EA:PE = 0-20%) as a light yellow oil (75.4 mg, 54% yield). **^{1}H NMR** (400 MHz, $CDCl_3$) δ 8.09 (s, 1H), 7.72 – 7.49 (m, 2H), 7.42 – 7.32 (m, 2H), 7.25 – 7.12 (m, 1H), 4.74 (dd, J = 10.1, 2.6 Hz, 1H), 4.40 – 4.27 (m, 1H), 3.24 – 3.03 (m, 1H), 2.99 – 2.84 (m, 2H), 2.86 – 2.66 (m, 1H), 1.41 (t, J = 7.5 Hz, 3H), 1.19 (d, J = 6.1 Hz, 3H), 1.17 (d, J = 6.1 Hz, 3H). **^{13}C NMR** (101 MHz, $CDCl_3$) δ 161.01 (t, $^2J_{C-F}$ = 27.9 Hz), 135.87,

129.39, 125.91, 120.38, 116.16 (t, $^{1}J_{C-F}$ = 259.6 Hz), 87.72 (dd, J = 5.5, 2.6 Hz), 75.34, 41.61, 35.34 (t, $^{2}J_{C-F}$ = 24.2 Hz), 22.86, 20.87, 5.21. **^{19}F NMR** (376 MHz, $CDCl_3$) δ -101.96 (d, J = 260.3 Hz, 1F), -105.95 (d, J = 260.3 Hz, 1F). **HRMS** (ESI) calcd for $C_{15}H_{21}F_2NO_4SNa$ $[M + Na]^+$: 372.1052, found: 372.1059.

1-(Ethylsulfonyl)-3,3-difluoro-4-oxo-4-(phenylamino)butyl benzoate (22)

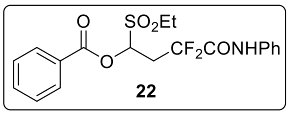

The title compound was prepared according to the ***General procedure I*** and purified by column chromatography (EA:PE = 0-30%) as a light yellow oil (89.1 mg, 54% yield). **^{1}H NMR** (400 MHz, $CDCl_3$) δ 8.06 – 7.81 (m, 3H), 7.64 – 7.52 (m, 1H), 7.44 – 7.34 (m, 4H), 7.25 (td, J = 7.2, 6.8, 1.8 Hz, 2H), 7.19 – 7.07 (m, 1H), 6.45 (dd, J = 8.9, 3.7 Hz, 1H), 3.23 – 3.12 (m, 2H), 3.09 – 3.00 (m, 2H), 1.41 (t, J = 7.5 Hz, 3H). **^{13}C NMR** (101 MHz, $CDCl_3$) δ 164.53, 160.49 (t, $^{2}J_{C-F}$ = 27.5 Hz), 135.62, 134.62, 130.44, 129.19, 128.84, 127.24, 125.81, 120.24, 116.06 (t, $^{1}J_{C-F}$ = 258.3 Hz), 79.12 – 78.01 (m), 45.14, 31.55 (t, $^{2}J_{C-F}$ = 25.1 Hz), 5.88. **^{19}F NMR** (376 MHz, $CDCl_3$) δ -102.34 (d, J = 261.4 Hz, 1F), -105.22 (d, J = 261.2 Hz, 1F). **HRMS** (ESI) calcd for $C_{19}H_{19}F_2NNaO_5S$ $[M + Na]^+$: 434.0844, found: 434.0847.

1-(Ethylsulfonyl)-3,3-difluoro-4-oxo-4-(phenylamino)butyl acetate (23)

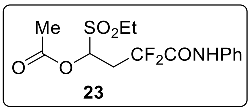

The title compound was prepared according to the ***General procedure I*** and purified by column chromatography (EA:PE = 0-30%) as light yellow oil (79.9 mg, 57% yield). **^{1}H NMR** (400 MHz, $CDCl_3$) δ 8.27 (s, 1H), 7.56 (d, J = 7.9 Hz, 2H), 7.35 (t, J = 7.9 Hz, 2H), 7.19 (t, J = 7.4 Hz, 1H), 6.17 (dd, J = 10.4, 1.8 Hz, 1H), 3.13 – 2.98 (m, 3H), 2.98 – 2.77 (m, 1H), 2.09 (s, 3H), 1.39 (t, J = 7.5 Hz, 3H). **^{13}C NMR** (101 MHz, $CDCl_3$) δ 168.91, 160.60 (t, $^{2}J_{C-F}$ = 27.6 Hz), 135.77, 129.34, 126.00, 120.43, 115.87 (t, $^{1}J_{C-F}$ = 257.0 Hz), 78.13 (t, $^{3}J_{C-F}$ = 4.1 Hz), 44.77, 31.91 (t, $^{2}J_{C-F}$ = 24.7 Hz), 20.49, 5.72. **^{19}F NMR** (376 MHz, $CDCl_3$) δ -103.39 (d, J = 260.5 Hz, 1F), -105.08 (d, J = 260.6 Hz, 1F). **HRMS** (ESI) calcd for $C_{14}H_{17}F_2NO_5SNa$ $[M + Na]^+$: 372.0688, found: 372.0686.

1-(Ethylsulfonyl)-3,3-difluoro-4-oxo-4-(phenylamino)butyl acetate (24)

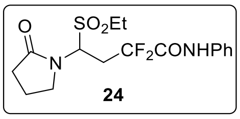

The title compound was prepared according to the ***General procedure I*** and purified by column chromatography (EA:PE = 0-30%) as a yellow solid (100.0 mg, 67% yield). **^{1}H NMR** (400 MHz, $CDCl_3$) δ 8.42 (s, 1H), 7.57 (d, J = 7.8 Hz, 2H), 7.34 (t, J = 7.9 Hz, 2H), 7.18 (t, J = 7.4 Hz, 1H), 5.78 – 5.48 (m, 1H), 3.79 – 3.45 (m, 2H), 3.12 – 2.87 (m, 4H), 2.36 – 2.24 (m, 1H), 2.13 – 2.03 (m, 1H), 2.03 – 1.75 (m, 2H), 1.36 (t, J = 7.5 Hz, 3H). **^{13}C NMR** (101 MHz, $CDCl_3$) δ 176.58, 160.55 (t, $^{2}J_{C-F}$ = 27.6 Hz), 135.78 (d,

$^{3}J_{C-F}$ = 2.9 Hz), 129.31, 126.01, 120.49, 116.14 (t, $^{1}J_{C-F}$ = 256.8 Hz), 63.07, 45.31, 43.31, 30.48, 27.03 (t, $^{2}J_{C-F}$ = 25.3 Hz), 18.38, 6.11. **^{19}F NMR** (376 MHz, $CDCl_3$) δ -102.32 (d, *J* = 258.2 Hz, 1F), -107.80 (d, *J* = 258.0 Hz, 1F). **HRMS** (ESI) calcd for $C_{16}H_{20}F_2N_2O_4SNa$ [M + Na]$^+$: 397.1004, found: 397.1015.

4-(9*H*-Carbazol-9-yl)-4-(ethylsulfonyl)-2,2-difluoro-*N*-phenylbutanamide (25)

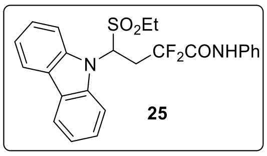

The title compound was prepared according to the ***General procedure I*** and purified by column chromatography (EA:PE = 0-30%) as a yellow solid (122.2 mg, 67% yield). **^{1}H NMR** (500 MHz, $CDCl_3$) δ 8.05 – 7.95 (m, 2H), 7.90 (d, *J* = 8.4 Hz, 1H), 7.60 (s, 1H), 7.51 – 7.38 (m, 3H), 7.35 – 7.19 (m, 4H), 7.18 – 7.07 (m, 3H), 6.18 (dd, *J* = 10.5, 2.7 Hz, 1H), 3.86 – 3.71 (m, 1H), 3.64 – 3.41 (m, 1H), 2.90 – 2.54 (m, 2H), 1.15 (t, *J* = 7.5 Hz, 3H). **^{13}C NMR** (126 MHz, $CDCl_3$) δ 160.15 (t, $^{2}J_{C-F}$ = 27.7 Hz), 140.09, 137.68, 135.33, 129.03, 126.75, 125.77, 124.72, 124.12, 121.50, 121.34, 120.85, 120.50, 120.21, 116.01 (t, $^{1}J_{C-F}$ = 257.5 Hz), 113.72, 108.08, 68.55 (t, $^{3}J_{C-F}$ = 4.0 Hz), 44.84, 28.81 (t, $^{2}J_{C-F}$ = 24.9 Hz), 5.50. **^{19}F NMR** (471 MHz, $CDCl_3$) δ -103.16 (d, *J* = 259.4 Hz, 1F), -105.10 (d, *J* = 259.4 Hz, 1F). **HRMS** (ESI) calcd for $C_{24}H_{22}F_2N_2O_3SNa$ [M + Na]$^+$: 479.1211, found: 479.1217.

(*E*)-6-(Ethylsulfonyl)-2,2-difluoro-*N*-phenylhex-4-enamide (26)

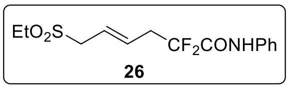

The title compound was prepared according to the ***General procedure I*** and purified by column chromatography (EA:PE = 0-20%) as a yellow solid (54.5 mg, 43% yield). **^{1}H NMR** (400 MHz, $CDCl_3$) δ 8.08 (s, 1H), 7.75 – 7.53 (m, 2H), 7.46 – 7.31 (m, 2H), 7.25 – 7.16 (m, 1H), 5.98 – 5.73 (m, 2H), 3.69 (d, *J* = 6.1 Hz, 2H), 3.02 (td, *J* = 16.3, 5.7 Hz, 2H), 2.91 (q, *J* = 7.5 Hz, 2H), 1.29 (t, *J* = 7.5 Hz, 3H). **^{13}C NMR** (101 MHz, $CDCl_3$) δ 161.40 (t, $^{2}J_{C-F}$ = 28.4 Hz), 135.99, 129.44 (t, $^{3}J_{C-F}$ = 5.5 Hz), 129.36, 125.88, 124.14, 120.37, 116.72 (t, $^{1}J_{C-F}$ = 256.6 Hz), 55.84, 45.91, 37.47 (t, $^{3}J_{C-F}$ = 24.4 Hz), 6.42. **^{19}F NMR** (376 MHz, $CDCl_3$) δ -104.77 (s, 2F). **HRMS** (ESI) calcd for $C_{14}H_{18}F_2NO_3S$ [M + H]$^+$: 318.0970, found: 318.0974.

4-(Ethylsulfonyl)-2,2-difluoro-*N*-phenyldecanamide (27)

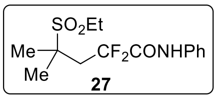

The title compound was prepared according to the ***General procedure I*** and purified by column chromatography (EA:PE = 0-30%) as a white solid (113.7 mg, 89% yield). **^{1}H NMR** (500 MHz, $CDCl_3$) δ 8.27 (s, 1H), 7.56 (d, *J* = 8.7 Hz, 2H), 7.36 (t, *J* = 7.8 Hz, 2H), 7.19 (t, *J* = 7.4 Hz, 1H), 2.99 (q, *J* = 7.4 Hz, 2H), 2.74 (t, *J* = 18.5 Hz, 2H), 1.57 (s, 6H), 1.41 (t, *J* = 7.4 Hz, 3H). **^{13}C NMR** (126 MHz, $CDCl_3$) δ 161.49 (t, $^{2}J_{C-F}$ = 28.0 Hz), 135.86, 129.31, 125.94, 120.54, 117.82 (t, $^{1}J_{C-F}$ = 257.2 Hz), 60.56, 40.13, 37.38 (t, $^{2}J_{C-F}$ = 22.4 Hz), 20.86

(t, $^{3}J_{C-F}$ = 2.3 Hz), 5.14. **^{19}F NMR** (471 MHz, $CDCl_3$) δ -101.79 (s, 2F). **HRMS** (ESI) calcd for $C_{14}H_{19}F_2NO_3SNa$ [M + Na]$^+$: 342.0946, found: 342.0951.

4-Ethyl-4-(ethylsulfonyl)-2,2-difluoro-*N*-phenylhexanamide (28)

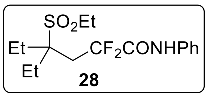

The title compound was prepared according to the ***General procedure I*** and purified by column chromatography (EA:PE = 0-30%) as a yellow oil (85.9 mg, 62% yield). **^{1}H NMR** (400 MHz, $CDCl_3$) δ 8.21 (s, 1H), 7.67 – 7.46 (m, 2H), 7.36 (t, *J* = 7.8 Hz, 2H), 7.27 – 7.10 (m, 1H), 3.04 (q, *J* = 7.4 Hz, 2H), 2.77 (t, *J* = 18.1 Hz, 2H), 2.10 – 1.87 (m, 4H), 1.39 (t, *J* = 7.4 Hz, 3H), 1.10 (t, *J* = 7.5 Hz, 6H). **^{13}C NMR** (101 MHz, $CDCl_3$) δ 161.75 (t, $^{2}J_{C-F}$ = 28.2 Hz), 136.00, 129.28, 125.80, 120.56, 117.49 (t, $^{1}J_{C-F}$ = 256.9 Hz), 67.46, 43.00, 35.09 (t, $^{2}J_{C-F}$ = 23.4 Hz), 25.78, 8.68, 4.95. **^{19}F NMR** (376 MHz, $CDCl_3$) δ -99.51 (s, 2F). **HRMS** (ESI) calcd for $C_{16}H_{24}F_2NO_3S$ [M + H]$^+$: 348.1439, found: 348.1448.

4-(Ethylsulfonyl)-2,2-difluoro-4,5-dimethyl-*N*-phenylhexanamide (29)

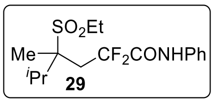

The title compound was prepared according to the ***General procedure I*** and purified by column chromatography (EA:PE = 0-30%) as a light yellow oil (87.1 mg, 63% yield). **^{1}H NMR** (400 MHz, $CDCl_3$) δ 8.22 (s, 1H), 7.56 (d, *J* = 8.3 Hz, 2H), 7.36 (t, *J* = 8.0 Hz, 2H), 7.19 (t, *J* = 7.4 Hz, 1H), 3.14 – 3.02 (m, 2H), 2.97 – 2.60 (m, 2H), 2.41 (p, *J* = 6.8 Hz, 1H), 1.54 (t, *J* = 1.3 Hz, 3H), 1.41 (t, *J* = 7.4 Hz, 3H), 1.16 (d, *J* = 6.8 Hz, 3H), 1.13 (d, *J* = 6.8 Hz, 3H). **^{13}C NMR** (101 MHz, $CDCl_3$) δ 161.76 (t, $^{2}J_{C-F}$ = 28.2 Hz), 136.00, 129.33, 125.87, 120.51, 117.55 (t, $^{1}J_{C-F}$ = 257.0 Hz), 67.52, 43.49, 34.01 (t, $^{2}J_{C-F}$ = 23.2 Hz), 33.73, 18.65, 18.20 (t, $^{3}J_{C-F}$ = 2.2 Hz), 5.26. **^{19}F NMR** (376 MHz, $CDCl_3$) δ -98.92 (d, *J* = 256.5 Hz, 1F), -100.08 (d, *J* = 256.4 Hz, 1F). **HRMS** (ESI) calcd for $C_{16}H_{23}F_2NO_3SNa$ [M + Na]$^+$: 370.1259, found: 370.1274.

6-(1,3-Dioxoisoindolin-2-yl)-4-(ethylsulfonyl)-2,2-difluoro-4-methyl-*N*-phenylhexanamide (30)

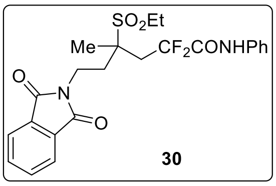

The title compound was prepared according to the ***General procedure I*** and purified by column chromatography (EA:PE = 0-30%) as a colorless oil (115.5 mg, 60% yield). **^{1}H NMR** (400 MHz, $CDCl_3$) δ 8.61 (s, 1H), 7.81 (dd, *J* = 5.5, 3.1 Hz, 2H), 7.71 (dd, *J* = 5.5, 3.0 Hz, 2H), 7.65 – 7.57 (m, 2H), 7.34 (t, *J* = 7.9 Hz, 2H), 7.21 – 7.11 (m, 1H), 4.07 – 3.83 (m, 2H), 3.14 (q, *J* = 7.3 Hz, 2H), 3.00 – 2.70 (m, 2H), 2.46 – 2.16 (m, 2H), 1.63 (s, 3H), 1.45 (t, *J* = 7.4 Hz, 3H). **^{13}C NMR** (101 MHz, $CDCl_3$) δ 168.14, 161.48 (t, $^{2}J_{C-F}$ = 28.2 Hz), 136.16, 134.26, 131.96, 129.23, 125.72, 123.43, 120.45, 117.57 (t, $^{1}J_{C-F}$ = 257.4 Hz), 62.61, 40.89, 35.07 (t, $^{2}J_{C-F}$ = 22.4 Hz), 33.54, 33.47, 19.88 (d, $^{3}J_{C-F}$ = 3.5 Hz), 5.02. **^{19}F NMR** (376 MHz, $CDCl_3$) δ -101.38 (d, *J* = 253.6 Hz, 1F), -103.37 (d, *J* = 253.5 Hz, 1F). **HRMS** (ESI) calcd for $C_{23}H_{25}F_2N_2O_5S$ [M + H]$^+$: 479.1447, found: 479.1456.

(*R*)-4-(Ethylsulfonyl)-2,2-difluoro-4-((1*R*,4*R*)-4-methyl-3-oxocyclohexyl)-*N*-phenylpentanamide (31)

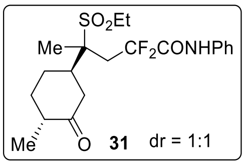

The title compound was prepared according to the ***General procedure I*** and purified by column chromatography (EA:PE = 0-20%) as a light yellow oil (88.1 mg, 53% yield). **^{1}H NMR** (400 MHz, $CDCl_3$) δ 8.47 (s, 0.5H), 8.41 (s, 0.5H), 7.56 (dd, *J* = 8.4, 3.0 Hz, 2H), 7.35 (t, *J* = 7.8 Hz, 2H), 7.19 (t, *J* = 7.4 Hz, 1H), 3.11 – 2.92 (m, 3H), 2.92 – 2.82 (m, 1H), 2.79 – 2.60 (m, 2H), 2.57 – 2.42 (m, 1H), 2.42 – 2.26 (m, 1H), 2.28 – 2.17 (m, 1H), 2.17 – 2.09 (m, 1H), 2.02 – 1.83 (m, 1H), 1.57 (d, *J* = 8.9 Hz, 3H), 1.37 (td, *J* = 7.5, 5.1 Hz, 4H), 1.28 – 1.09 (m, 1H), 0.99 (s, 1H), 0.98 (s, 1H). **^{13}C NMR** (101 MHz, $CDCl_3$) δ 211.45, 211.17, 161.46 (t, $^2J_{C-F}$ = 28.3 Hz), 161.40 (t, $^2J_{C-F}$ = 28.4 Hz), 135.92, 135.87, 129.30, 125.98, 125.94, 120.58, 117.57 (t, $^1J_{C-F}$ = 257.6), 117.51 (t, $^1J_{C-F}$ = 259.6), 66.39, 65.73, 46.23, 45.59, 44.99, 44.94, 44.83, 43.64, 43.54, 43.48, 35.25 (t, $^2J_{C-F}$ = 22.1 Hz), 34.34 (t, $^2J_{C-F}$ = 22.0 Hz), 34.34, 34.23, 27.47, 27.32, 19.19, 18.81, 14.21, 14.19, 5.17, 5.00. **^{19}F NMR** (376 MHz, $CDCl_3$) δ -98.71 – -99.87 (m), -100.65 – -101.98 (m). **HRMS** (ESI) calcd for $C_{20}H_{28}F_2NO_4S$ $[M + H]^+$: 416.1702, found: 416.1714.

3-(4-(*tert*-Butyl)-1-(ethylsulfonyl)cyclohexyl)-2,2-difluoro-*N*-phenylpropanamide (32)

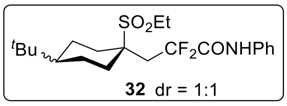

The title compound was prepared according to the ***General procedure I*** and purified by column chromatography (EA:PE = 0-20%) as a light yellow oil (144.4 mg, 87% yield). **^{1}H NMR** (400 MHz, $CDCl_3$) δ 8.28 (s, 0.5H), 8.16 (s, 0.5H), 7.56 – 7.50 (m, 2H), 7.36 (dt, *J* = 8.5, 7.1 Hz, 2H), 7.26 – 7.09 (m, 1H), 3.10 (qd, *J* = 7.5, 1.5 Hz, 2H), 2.82 (t, *J* = 16.6 Hz, 1H), 2.63 (t, *J* = 17.5 Hz, 1H), 2.46 (d, *J* = 10.5 Hz, 1H), 2.04 – 1.91 (m, 2H), 1.87 – 1.75 (m, 2H), 1.60 (d, *J* = 5.3 Hz, 1H), 1.45 – 1.33 (m, 3H), 1.32 – 1.19 (m, 2H), 1.05 (dt, *J* = 12.3, 3.2 Hz, 1H), 0.87 (s, 4.5H), 0.84 (s, 4.5H). **^{13}C NMR** (101 MHz, $CDCl_3$) δ 161.81 (t, $^2J_{C-F}$ = 27.7 Hz), 161.64 (t, $^2J_{C-F}$ = 28.1 Hz), 136.14, 135.99, 129.36, 129.26, 125.91, 125.70, 120.76, 120.58, 117.53, 117.29, 64.30, 62.56, 47.04, 46.56, 41.84, 40.91, 40.98, 33.86, 32.59, 32.45, 31.34, 29.42, 27.56, 27.47, 22.62, 22.13, 5.01, 4.81. **^{19}F NMR** (376 MHz, $CDCl_3$) **Isomer 1** δ -96.37; **Isomer 2** δ -98.69. **HRMS** (ESI) calcd for $C_{21}H_{31}F_2NO_3SNa$ $[M + Na]^+$: 438.1885, found: 438.1898.

3-(1-(Ethylsulfonyl)-4-phenylcyclohexyl)-2,2-difluoro-*N*-phenylpropanamide (33)

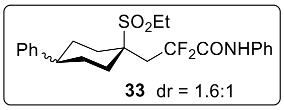

The title compound was prepared according to the ***General procedure I*** and purified by column chromatography (EA:PE = 0-20%) as a light yellow oil (155.0 mg, 89% yield). **^{1}H NMR** (400 MHz, $CDCl_3$) δ 8.32 (s, 0.38H), 8.26 (s, 0.62H), 7.65 – 7.49 (m, 2H), 7.41 – 7.08 (m, 8H), 3.14 (dq, *J* = 9.4, 7.4 Hz, 2H),

2.96 (t, J = 16.6 Hz, 0.8H), 2.72 (t, J = 17.6 Hz, 1.2H), 2.62 – 2.45 (m, 2H), 2.34 (qd, J = 12.7, 3.7 Hz, 1H), 2.19 – 1.91 (m, 4H), 1.81 – 1.69 (m, 2H), 1.41 (td, J = 7.4, 5.1 Hz, 3H). **^{13}C NMR** (101 MHz, $CDCl_3$) **Minor isomer** δ 161.72 (t, $^2J_{C-F}$ = 27.7 Hz), 145.16, 136.05, 129.24, 128.66, 126.78, 126.67, 125.74, 120.73, 117.27 (t, $^1J_{C-F}$ = 255.6 Hz), 63.89, 42.93, 41.03, 33.78 (t, $^2J_{C-F}$ = 25.1 Hz), 29.18, 28.52, 4.83; **Major isomer** δ 161.58 (t, $^2J_{C-F}$ = 27.8 Hz), 146.09, 135.92, 129.33, 128.51, 127.08, 126.33, 125.92, 120.60, 117.54 (t, $^1J_{C-F}$ = 257.1 Hz), 62.36, 42.45, 41.77, 40.21 (t, $^2J_{C-F}$ = 22.8 Hz), 30.60, 28.90, 5.01. **^{19}F NMR** (376 MHz, $CDCl_3$) **Minor isomer** δ -96.56, **Major isomer** -98.75. **HRMS** (ESI) calcd for $C_{23}H_{27}F_2NO_3SNa$ $[M + Na]^+$: 458.1572, found: 458.1586.

Ethyl 4-(2,2-difluoro-3-oxo-3-(phenylamino)propyl)-4-(ethylsulfonyl)cyclohexane-1-carboxylate (34)

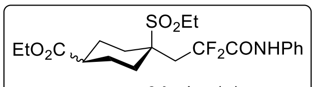

34 dr =1:1

The title compound was prepared according to the ***General procedure I*** and purified by column chromatography (EA:PE = 0-30%) as a light yellow oil (156.9 mg, 91% yield). **^{1}H NMR** (400 MHz, $CDCl_3$) δ 8.34 (s, 0.5H), 8.28 (s, 0.5H), 7.61 – 7.45 (m, 2H), 7.40 – 7.29 (m, 2H), 7.21 – 7.07 (m, 1H), 4.12 (q, J = 7.1 Hz, 2H), 3.08 (dq, J = 15.0, 7.4 Hz, 2H), 2.81 (dt, J = 27.0, 16.9 Hz, 2H), 2.61 – 2.25 (m, 1H), 2.28 – 2.11 (m, 2H), 2.12 – 1.96 (m, 3H), 1.90 – 1.53 (m, 3H), 1.36 (td, J = 7.4, 4.0 Hz, 3H), 1.23 (td, J = 7.1, 5.0 Hz, 3H). **^{13}C NMR** (101 MHz, $CDCl_3$) δ 174.44, 174.21, 161.62, 161.67 (t, $^2J_{C-F}$ = 27.8 Hz), 136.10, 136.07, 129.21, 129.17, 125.74, 125.68, 120.79, 120.69, 117.21 (t, $^1J_{C-F}$ = 256.1 Hz), 63.58, 63.26, 60.73, 60.70, 46.24, 41.66, 38.91, 41.39, 41.07, 35.85 (t, $^2J_{C-F}$ = 24.3 Hz), 33.76 (t, $^2J_{C-F}$ = 24.9 Hz), 29.74, 28.01, 27.09, 23.51, 22.90, 14.27, 14.25, 4.99, 4.77. **^{19}F NMR** (376 MHz, $CDCl_3$) **Isomer 1** δ -96.79; **Isomer 2** δ -97.79. **HRMS** (ESI) calcd for $C_{20}H_{28}F_2NO_5S$ $[M + H]^+$: 432.1651, found: 432.1653.

***tert*-Butyl 4-(2,2-difluoro-3-oxo-3-(phenylamino)propyl)-4-(ethylsulfonyl)piperidine-1-carboxylate (35)**

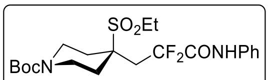

35

The title compound was prepared according to the ***General procedure I*** and purified by column chromatography (EA:PE = 0-30%) as a light yellow oil (110.1 mg, 60% yield). **^{1}H NMR** (400 MHz, $CDCl_3$) δ 8.37 (s, 1H), 7.54 (d, J = 7.7 Hz, 2H), 7.34 (t, J = 7.8 Hz, 2H), 7.17 (t, J = 7.4 Hz, 1H), 4.00 (s, 2H), 3.08 (q, J = 7.4 Hz, 4H), 3.00 – 2.72 (m, 2H), 2.24 – 2.11 (m, 2H), 2.04 – 1.83 (m, 2H), 1.43 (s, 9H), 1.37 (t, J = 7.4 Hz, 3H). **^{13}C NMR** (101 MHz, $CDCl_3$) δ 161.50 (t, $^2J_{C-F}$ = 27.8 Hz), 154.50, 135.98, 129.23, 125.83, 120.77, 117.13 (t, $^1J_{C-F}$ = 256.5 Hz), 80.31, 62.42 (t, $^3J_{C-F}$ = 2.4 Hz), 46.22, 41.32, 38.96 (d, 108.6 Hz), 34.48 (t, $^2J_{C-F}$ = 24.3 Hz), 28.42, 4.87. **^{19}F NMR** (376 MHz, $CDCl_3$) δ -97.14 (s, 1F), -97.21 (s, 1F). **HRMS** (ESI) calcd for $C_{21}H_{30}F_2N_2O_5SNa$ $[M + Na]^+$: 483.1736, found: 483.1736.

4-(Ethylsulfonyl)-2,2-difluoro-*N*-phenyl-3-propylheptanamide (36)

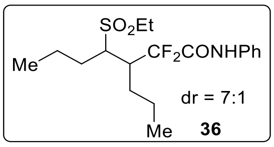

The title compound was prepared according to the ***General procedure I*** and purified by column chromatography (EA:PE = 0-10%) as a light yellow oil (79.6 mg, 53% yield). **^{1}H NMR** (500 MHz, $CDCl_3$) δ 8.12 (s, 1H), 7.56 (d, *J* = 7.7 Hz, 2H), 7.39 (t, *J* = 8.0 Hz, 2H), 7.22 (t, *J* = 7.5 Hz, 1H), 3.33 – 3.24 (m, 1H), 3.23 – 3.11 (m, 1H), 3.06 (q, *J* = 7.4 Hz, 2H), 2.12 – 1.99 (m, 1H), 1.80 – 1.65 (m, 3H), 1.66 – 1.57 (m, 2H), 1.55 – 1.45 (m, 2H), 1.39 (t, *J* = 7.4 Hz, 3H), 0.96 (t, *J* = 7.2 Hz, 3H), 0.91 (t, *J* = 7.3 Hz, 3H). **^{13}C NMR** (126 MHz, $CDCl_3$) δ 161.48 (t, $^{2}J_{C-F}$ = 28.7 Hz), 135.80, 129.47, 126.11, 120.49, 118.86 (t, $^{1}J_{C-F}$ = 258.7 Hz), 59.03 (t, $^{3}J_{C-F}$ = 3.2 Hz), 47.14, 40.84 (t, $^{2}J_{C-F}$ = 21.5 Hz), 26.46, 22.59, 22.11, 22.09, 14.36, 14.15, 6.49. **^{19}F NMR** (471 MHz, $CDCl_3$) **Major isomer** δ -107.00 (d, *J* = 255.8 Hz), -109.08 (d, *J* = 255.8 Hz); **Minor isomer** δ -101.22 (d, *J* = 254.5 Hz), -106.61 (d, *J* = 255.5 Hz). **HRMS** (ESI) calcd for $C_{18}H_{27}F_2NO_3SNa$ $[M + Na]^+$: 398.1572, found: 398.1579.

4-(Ethylsulfonyl)-2,2-difluoro-3-hexyl-*N*-phenyldecanamide (37)

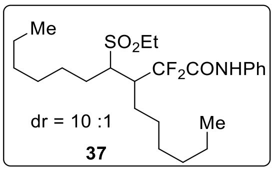

The title compound was prepared according to the ***General procedure I*** and purified by column chromatography (EA:PE = 0-10%) as a light yellow oil (93.8 mg, 51% yield). **^{1}H NMR** (400 MHz, $CDCl_3$) δ 8.13 (s, 1H), 7.57 (d, *J* = 7.7 Hz, 2H), 7.39 (t, *J* = 7.8 Hz, 2H), 7.22 (t, *J* = 7.4 Hz, 1H), 3.29 – 3.22 (m, 1H), 3.21 – 3.10 (m, 1H), 3.06 (q, *J* = 7.4 Hz, 2H), 2.09 – 1.94 (m, 1H), 1.83 – 1.68 (m, 2H), 1.66 – 1.53 (m, 2H), 1.50 – 1.36 (m, 6H), 1.35 – 1.17 (m, 12H), 0.84 (dt, *J* = 18.5, 6.6 Hz, 6H). **^{13}C NMR** (101 MHz, $CDCl_3$) δ 161.46 (t, $^{2}J_{C-F}$ = 28.6 Hz), 135.78, 129.44, 126.07, 120.42, 118.82 (t, $^{1}J_{C-F}$ = 259.2 Hz), 59.28 (t, $^{3}J_{C-F}$ = 3.3 Hz), 47.06, 41.14 (t, $^{2}J_{C-F}$ = 21.5 Hz), 31.54, 31.52, 29.54, 29.40, 28.85, 24.40, 24.35, 24.31, 22.68, 22.64, 14.16, 14.14, 6.48. **^{19}F NM**R (376 MHz, $CDCl_3$) **Major isomer** δ -106.87 (d, *J* = 254.9 Hz), -109.42 (d, *J* = 254.9 Hz); **Minor isomer** δ -105.28 (d, *J* = 529.3 Hz), -109.25 (d, *J* = 255.2 Hz). **HRMS** (ESI) calcd for $C_{24}H_{39}F_2NO_3SNa$ $[M + Na]^+$: 482.2511, found: 482.2522.

2-((3*S*,4*S*)-4-(Ethylsulfonyl)tetrahydrofuran-3-yl)-2,2-difluoro-*N*-phenylacetamide (38)

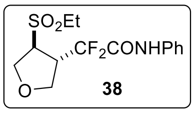

The title compound was prepared according to the ***General procedure I*** and purified by column chromatography (EA:PE = 0-30%) as a white solid (121.3 mg, 91% yield). **^{1}H NMR** (400 MHz, $CDCl_3$) δ 8.29 (s, 1H), 7.54 (d, *J* = 7.8 Hz, 2H), 7.37 (t, *J* = 7.9 Hz, 2H), 7.21 (t, *J* = 7.4 Hz, 1H), 4.33 (dd, *J* = 10.2, 4.7 Hz, 1H), 4.22 – 3.96 (m, 4H), 3.70 – 3.45 (m, 1H), 3.07 (q, *J* = 7.5 Hz, 2H), 1.40 (t, *J* = 7.4 Hz, 3H). **^{13}C NMR** (101 MHz, $CDCl_3$) δ 160.63 (t, $^{2}J_{C-F}$ = 28.3 Hz), 135.61, 129.38, 126.23, 120.76, 116.39 (t, $^{1}J_{C-F}$ = 259.3 Hz), 68.47 (t, $^{3}J_{C-F}$ = 4.2 Hz), 67.71, 60.40 (t, $^{3}J_{C-F}$ = 2.7 Hz), 46.58, 45.89 (t, $^{2}J_{C-F}$ = 22.9 Hz), 6.06.

^{19}**F NMR** (376 MHz, $CDCl_3$) δ -104.66 (d, *J* = 261.3 Hz, 1F), -110.34 (d, *J* = 261.0 Hz, 1F). **HRMS** (ESI) calcd for $C_{14}H_{17}F_2NO_4SNa$ [M + Na]$^+$: 356.0739, found: 356.0755.

2-((1*S*,2*R*)-2-(Ethylsulfonyl)cyclohexyl)-2,2-difluoro-*N*-phenylacetamide (39)

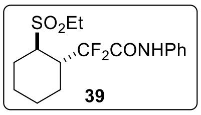

The title compound was prepared according to the ***General procedure I*** and purified by column chromatography (EA:PE = 0-20%) as a light yellow oil (88.4 mg, 64% yield). ^{1}**H NMR** (400 MHz, $CDCl_3$) δ 8.34 (s, 1H), 7.57 (d, *J* = 9.6 Hz, 2H), 7.36 (t, *J* = 8.0 Hz, 2H), 7.20 (t, *J* = 7.4 Hz, 1H), 3.39 – 3.27 (m, 1H), 3.19 – 2.95 (m, 3H), 2.25 (dd, *J* = 14.5, 4.4 Hz, 1H), 2.20 – 2.06 (m, 1H), 2.06 – 1.90 (m, 1H), 1.88 – 1.68 (m, 2H), 1.57 (q, *J* = 4.5 Hz, 3H), 1.36 (t, *J* = 7.4 Hz, 3H). ^{13}**C NMR** (101 MHz, $CDCl_3$) δ 161.37 (t, $^2J_{C\text{-}F}$ = 28.5 Hz), 135.96, 129.34, 125.93, 120.55, 118.91 (t, $^1J_{C\text{-}F}$ = 259.3 Hz), 55.20 (t, $^3J_{C\text{-}F}$ = 2.6 Hz), 45.60, 36.79 (t, $^2J_{C\text{-}F}$ = 21.6 Hz), 22.63, 21.72 (t, $^3J_{C\text{-}F}$ = 3.6 Hz), 21.58, 21.52, 6.34. ^{19}**F NMR** (376 MHz, $CDCl_3$) δ -104.09 (d, *J* = 257.5 Hz, 1F), -105.07 (d, *J* = 257.6 Hz, 1F). **HRMS** (ESI) calcd for $C_{16}H_{21}F_2NO_3SNa$ [M + Na]$^+$: 368.1102, found: 368.1110.

2-((2*S*,3*R*)-3-(Ethylsulfonyl)bicyclo[2.2.1]heptan-2-yl)-2,2-difluoro-*N*-phenylacetamide (40)

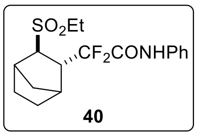

The title compound was prepared according to the ***General procedure I*** and purified by column chromatography (EA:PE = 0-20%) as a white solid (137.1 mg, 87% yield). ^{1}**H NMR** (400 MHz, $CDCl_3$) δ 8.33 (s, 1H), 7.59 (d, *J* = 8.3 Hz, 2H), 7.36 (t, *J* = 7.9 Hz, 2H), 7.19 (t, *J* = 7.4 Hz, 1H), 3.78 – 3.65 (m, 1H), 3.13 – 2.91 (m, 3H), 2.87 (d, *J* = 4.1 Hz, 1H), 2.54 (d, *J* = 4.5 Hz, 1H), 2.28 – 2.15 (m, 1H), 2.07 – 1.59 (m, 3H), 1.57 – 1.43 (m, 2H), 1.37 (t, *J* = 7.4 Hz, 3H). ^{13}**C NMR** (101 MHz, $CDCl_3$) δ 161.30 (t, $^2J_{C\text{-}F}$ = 28.7 Hz), 135.91, 129.28, 125.92, 120.59, 117.20 (t, $^1J_{C\text{-}F}$ = 259.6 Hz), 61.54, 48.53 (d, *J* = 4.9 Hz), 48.08 (t, $^2J_{C\text{-}F}$ = 21.7 Hz), 39.65, 39.58, 38.18 (d, *J* = 2.9 Hz), 28.74, 23.74, 6.26. ^{19}**F NMR** (376 MHz, $CDCl_3$) δ -100.68 (d, *J* = 255.8 Hz, 1F), -111.77 (d, *J* = 256.4 Hz, 1F). **HRMS** (ESI) calcd for $C_{17}H_{21}F_2NO_3SNa$ [M + Na]$^+$: 380.1102, found: 380.1113.

2-((1*S*,2*R*)-2-(Ethylsulfonyl)-2-methylcyclohexyl)-2,2-difluoro-*N*-phenylacetamide (41)

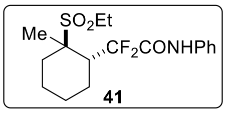

The title compound was prepared according to the ***General procedure I*** and purified by column chromatography (EA:PE = 0-20%) as a yellow oil (66.7 mg, 46% yield). ^{1}**H NMR** (400 MHz, $CDCl_3$) δ 8.50 (s, 1H), 7.57 (d, *J* = 7.7 Hz, 2H), 7.35 (t, *J* = 7.9 Hz, 2H), 7.17 (t, *J* = 7.4 Hz, 1H), 3.25 – 2.89 (m, 3H), 2.17 (dd, *J* = 14.3, 3.9 Hz, 1H), 2.03 – 1.92 (m, 1H), 1.82 (td, *J* = 10.7, 9.0, 4.6 Hz, 3H), 1.71 (dt, *J* = 11.7, 3.3 Hz, 1H), 1.50 (s, 3H), 1.41 (t, *J* = 7.4 Hz, 4H), 1.37 – 1.26 (m, 1H). ^{13}**C NMR** (101 MHz, $CDCl_3$) δ 160.83 (t, $^2J_{C\text{-}F}$ = 28.4 Hz), 135.36, 128.24, 124.55, 119.45, 116.55 (t, $^1J_{C\text{-}F}$ = 258.1 Hz), 64.85, 43.83 (t,

$^{2}J_{C-F}$ = 21.7 Hz), 40.80 (t, $^{3}J_{C-F}$ = 2.7 Hz), 34.09, 23.61, 21.86 (t, $^{3}J_{C-F}$ = 6.4 Hz), 20.45, 13.25, 4.28. **^{19}F NMR** (376 MHz, $CDCl_3$) δ -99.94 (d, *J* = 260.1 Hz, 1F), -100.72 (d, *J* = 259.4 Hz, 1F). **HRMS** (ESI) calcd for $C_{17}H_{23}F_2NO_3SNa$ [M + Na]$^+$: 382.1259, found: 382.1269.

2-(2-(Ethylsulfonyl)cycloheptyl)-2,2-difluoro-*N*-phenylacetamide (42)

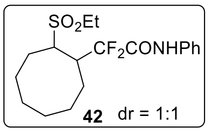

The title compound was prepared according to the ***General procedure I*** and purified by column chromatography (EA:PE = 0-20%) as a yellow oil (102.9 mg, 69% yield). **^{1}H NMR** (400 MHz, $CDCl_3$) δ 8.24 (s, 1H), 7.58 (d, *J* = 7.7 Hz, 2H), 7.35 (t, *J* = 7.8 Hz, 2H), 7.18 (t, *J* = 7.4 Hz, 1H), 3.13 – 2.86 (m, 3H), 2.64 – 2.34 (m, 1H), 2.20 – 2.07 (m, 2H), 2.03 – 1.47 (m, 10H), 1.36 (td, *J* = 7.5, 1.6 Hz, 3H). **^{13}C NMR** (101 MHz, $CDCl_3$) δ 162.11 (t, $^{2}J_{C-F}$ = 29.3), 162.01 (t, $^{2}J_{C-F}$ = 29.3), 136.06, 136.03, 129.24, 125.75, 125.73, 120.45, 119.64 (t, $^{1}J_{C-F}$ = 259.6), 119.58 (t, $^{1}J_{C-F}$ = 257.6), 61.48, 60.10, 44.12, 43.56, 40.85 (t, $^{2}J_{C-F}$ = 20.7 Hz), 40.00 (t, $^{2}J_{C-F}$ = 20.5 Hz), 26.02, 25.62, 25.58, 25.03, 25.19, 25.09, 24.99, 24.83, 24.71 (t, $^{3}J_{C-F}$ = 4.0 Hz), 23.12 (t, $^{3}J_{C-F}$ = 3.7 Hz), 24.62, 23.26, 6.18, 5.98. **^{19}F NMR** (376 MHz, $CDCl_3$) **Isomer 1** δ -111.11 (d, *J* = 255.7 Hz), -112.32 (d, *J* = 251.9 Hz); **Isomer 2** δ -110.52 (d, *J* = 255.7 Hz), -113.12 (d, *J* = 251.9 Hz). **HRMS** (ESI) calcd for $C_{18}H_{25}F_2NO_3SNa$ [M + Na]$^+$: 396.1415, found: 396.1434.

3-(2-(Ethylsulfonyl)propan-2-yl)-2,2-difluoro-6-oxo-*N*-phenylheptanamide (43)

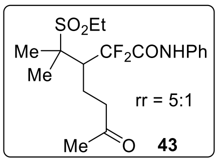

The title compound was prepared according to the ***General procedure I*** and purified by column chromatography (EA:PE = 0-20%) as a light yellow oil (70.0 mg, 45% yield). **^{1}H NMR** (400 MHz, $CDCl_3$) δ 8.40 (s, 1H), 7.58 (d, *J* = 8.6 Hz, 2H), 7.37 (t, *J* = 7.9 Hz, 2H), 7.21 (t, *J* = 7.4 Hz, 1H), 3.26 – 2.94 (m, 4H), 2.83 – 2.61 (m, 2H), 2.47 – 2.34 (m, 1H), 2.10 (s, 3H), 2.05 – 1.92 (m, 1H), 1.58 – 1.49 (m, 5H), 1.42 (t, *J* = 7.4 Hz, 3H). **^{13}C NMR** (101 MHz, $CDCl_3$) δ 208.57, 161.81 (t, $^{2}J_{C-F}$ = 28.3 Hz), 135.94, 129.41, 126.02, 120.50, 119.60 (t, $^{1}J_{C-F}$ = 258.2 Hz), 63.93, 63.90, 43.96, 43.92, 43.05 (t, $^{2}J_{C-F}$ = 20.2 Hz), 41.21, 30.01, 22.03, 21.99, 19.41 (d, $^{3}J_{C-F}$ = 4.7 Hz), 19.36 (d, $^{3}J_{C-F}$ = 4.7 Hz), 18.59, 5.35. **^{19}F NMR** (376 MHz, $CDCl_3$) **Major isomer** δ -100.37 (d, *J* = 266.9 Hz), -106.07 (d, *J* = 266.4 Hz); **Minor isomer** -108.11 (d, *J* = 255.6 Hz), -109.89 (d, *J* = 255.5 Hz). **HRMS** (ESI) calcd for $C_{18}H_{25}F_2NO_4SNa$ [M + Na]$^+$: 412.1365, found: 412.1376.

4-(Ethylsulfonyl)-2,2-difluoro-3,4-dimethyl-*N*-phenylpentanamide (44)

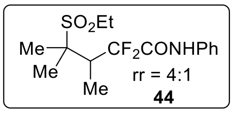

The title compound was prepared according to the ***General procedure I*** and purified by column chromatography (EA:PE = 0-20%) as a light yellow oil (117.2 mg, 88% yield). **^{1}H NMR** (500 MHz, $CDCl_3$) δ 8.21 (s, 1H), 7.62 –

7.50 (m, 2H), 7.37 (t, J = 7.8 Hz, 2H), 7.20 (t, J = 7.4 Hz, 1H), 3.33 – 3.22 (m, 0.8H), 3.14 – 2.94 (m, 1.6H), 2.86 – 2.69 (m, 0.2H), 2.08 – 1.94 (m, 0.4H), 1.59 (d, J = 3.3 Hz, 3H), 1.51 (d, J = 5.0 Hz, 3H), 1.42 (t, J = 7.5 Hz, 2.4H), 1.38 (dd, J = 7.1, 1.6 Hz, 3H), 1.11 (t, J = 7.5 Hz, 0.6H). **^{13}C NMR** (126 MHz, $CDCl_3$) δ 161.87 (t, $^2J_{C\text{-}F}$ = 28.6 Hz), 135.91, 135.97, 129.37, 129.32, 125.97, 125.90, 120.54, 120.58, 119.78 (t, $^1J_{C\text{-}F}$ = 262.1 Hz), 120.03 (t, $^1J_{C\text{-}F}$ = 260.9 Hz), 63.94, 63.88, 41.28, 41.50, 38.88 (t, $^2J_{C\text{-}F}$ = 20.4 Hz), 35.43 (t, $^2J_{C\text{-}F}$ = 23.2 Hz), 22.27 (d, $^3J_{C\text{-}F}$ = 6.5 Hz), 28.05, 18.10, 19.65, 11.04 (t, $^3J_{C\text{-}F}$ = 5.1 Hz), 8.91, 5.41, 5.09. **^{19}F NMR** (471 MHz, $CDCl_3$) **Minor isomer** δ -100.50; **Major isomer** δ -102.71 (d, J = 257.6 Hz), -108.63 (dd, J = 258.3, 5.6 Hz). **HRMS** (ESI) calcd for $C_{15}H_{21}F_2NO_3SNa$ [M + Na]$^+$: 356.1102, found: 356.1117.

4-(Ethylsulfonyl)-2,2-difluoro-3,3,4-trimethyl-*N*-phenylpentanamide (45)

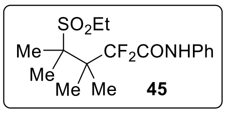

The title compound was prepared according to the ***General procedure I*** and purified by column chromatography (EA:PE = 0-20%) as a yellow oil (45.9 mg, 33% yield). **^{1}H NMR** (500 MHz, $CDCl_3$) δ 8.54 (s, 1H), 7.57 (d, J = 7.8 Hz, 2H), 7.37 (t, J = 8.0 Hz, 2H), 7.19 (t, J = 7.5 Hz, 1H), 3.09 (q, J = 7.4 Hz, 2H), 1.58 (d, J = 3.8 Hz, 12H), 1.43 (t, J = 7.4 Hz, 3H). **^{13}C NMR** (126 MHz, $CDCl_3$) δ 161.76 (t, $^2J_{C\text{-}F}$ = 29.0 Hz), 136.28, 129.34, 125.73, 120.53, 68.68, 48.60 (t, $^2J_{C\text{-}F}$ = 20.5 Hz), 44.25, 21.40 (t, $^3J_{C\text{-}F}$ = 2.6 Hz), 20.21 (t, $^3J_{C\text{-}F}$ = 4.6 Hz), 5.44. **^{19}F NMR** (471 MHz, $CDCl_3$) δ -103.87 (s, 2F). **HRMS** (ESI) calcd for $C_{16}H_{23}F_2NO_3SNa$ [M + Na]$^+$: 370.1259, found: 370.1261.

2,2-Difluoro-4-(methylsulfonyl)-*N*-phenylbutanamide (46)

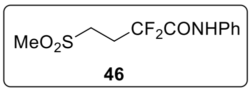

The title compound was prepared according to the ***General procedure I*** and purified by column chromatography (EA:PE = 0-30%) as a white solid (63.3 mg, 57% yield). **^{1}H NMR** (400 MHz, DMSO-d_6) δ 10.68 (s, 1H), 7.69 (d, J = 8.8 Hz, 2H), 7.38 (t, J = 7.9 Hz, 2H), 7.17 (t, J = 7.5 Hz, 1H), 3.53 – 3.28 (m, 2H), 3.09 (s, 3H), 2.74 – 2.56 (m, 2H). **^{13}C NMR** (101 MHz, DMSO-d_6) δ 161.17 (t, $^2J_{C\text{-}F}$ = 28.8 Hz), 137.11, 128.84, 125.06, 120.97, 114.07, 46.49, 34.78, 27.05 (t, $^2J_{C\text{-}F}$ = 24.6 Hz). **^{19}F NMR** (376 MHz, DMSO-d_6) δ -104.13 (s, 2F). **HRMS** (ESI) calcd for $C_{11}H_{14}F_2NO_3S$ [M + H]$^+$: 278.0657, found: 278.0659.

(8*R*,9*S*,10*R*,13*S*,14*S*)-10,13-Dimethyl-3-oxo-6,7,8,9,10,11,12,13,14,15,16,17-dodecahydro-3*H*-cyclopenta[*a*]phenanthren-17-yl 10-(ethylsulfonyl)-12,12-difluoro-13-oxo-13-(phenylamino) tridecanoate (47)

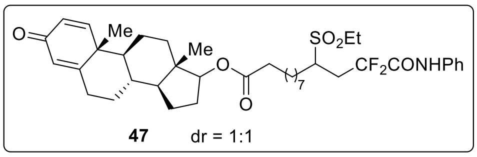

The title compound was prepared according to the ***General procedure I*** and purified by column chromatography (EA:PE = 0-30%) as a yellow oil

(203.6 mg, 71% yield). **^{1}H NMR** (400 MHz, $CDCl_3$) δ 8.88 (s, 1H), 7.57 (d, *J* = 7.9 Hz, 2H), 7.29 (q, *J* = 6.7, 5.7 Hz, 2H), 7.13 (t, *J* = 7.5 Hz, 1H), 7.02 (d, *J* = 10.2 Hz, 1H), 6.17 (dd, *J* = 10.1, 1.9 Hz, 1H), 6.01 (s, 1H), 4.53 (t, *J* = 8.4 Hz, 1H), 3.32 – 3.20 (m, 1H), 2.99 (q, *J* = 7.4 Hz, 2H), 2.89 – 2.75 (m, 1H), 2.57 – 2.35 (m, 2H), 2.34 – 2.17 (m, 4H), 2.15 – 1.81 (m, 4H), 1.80 – 1.39 (m, 11H), 1.33 (t, *J* = 7.3 Hz, 4H), 1.20 (d, *J* = 17.7 Hz, 8H), 1.14 – 1.07 (m, 1H), 1.05 – 0.92 (m, 3H), 0.82 (d, *J* = 5.4 Hz, 4H). **^{13}C NMR** (101 MHz, $CDCl_3$) δ 186.38, 173.80, 169.36, 161.25 (t, $^2J_{C-F}$ = 28.4 Hz), 156.09, 136.09, 129.04, 127.32, 125.60, 123.72, 120.55, 116.53 (t, $^1J_{C-F}$ = 255.5 Hz), 81.99, 55.09, 52.14, 49.76, 45.06, 43.55, 42.68, 36.43, 35.17, 34.41, 33.00, 32.67 (d, $^2J_{C-F}$ = 23.9 Hz), 32.66, 29.33, 28.99, 28.94, 28.91, 28.59, 27.38, 26.10, 24.93, 23.59, 22.28, 18.64, 12.12, 6.01. **^{19}F NMR** (376 MHz, $CDCl_3$) **Isomer 1** δ -102.47 (d, *J* = 256.3 Hz), -104.55 (d, *J* = 256.2 Hz); **Isomer 2** δ -102.50 (d, *J* = 256.3 Hz), -104.55 (d, *J* = 256.2 Hz). **HRMS** (ESI) calcd for $C_{40}H_{56}F_2NO_6S$ [M + H]$^+$: 716.3796, found: 716.3800.

4-(Ethylsulfonyl)-6,6-difluoro-7-oxo-7-(phenylamino)heptyl 2-(4-(2,2-dichlorocyclopropyl) phenoxy)-2-methylpropanoate (48)

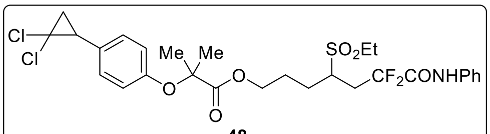

48

The title compound was prepared according to the ***General procedure I*** and purified by column chromatography (EA:PE = 0-20%) as a yellow oil (160.8 mg, 65% yield). **^{1}H NMR** (400 MHz, $CDCl_3$) δ 8.29 (s, 1H), 7.56 (d, *J* = 7.9 Hz, 2H), 7.35 (t, *J* = 7.9 Hz, 2H), 7.19 (t, *J* = 7.4 Hz, 1H), 7.10 (d, *J* = 8.5 Hz, 2H), 6.78 (d, *J* = 8.6 Hz, 2H), 4.17 (q, *J* = 5.6 Hz, 2H), 3.28 (dq, *J* = 7.9, 2.4 Hz, 1H), 3.01 – 2.93 (m, 2H), 2.82 – 2.70 (m, 2H), 2.44 – 2.24 (m, 1H), 2.00 – 1.66 (m, 6H), 1.59 (s, 6H), 1.42 – 1.24 (m, 3H). **^{13}C NMR** (101 MHz, $CDCl_3$) δ 174.26, 160.96 (t, $^2J_{C-F}$ = 28.2 Hz), 154.86, 135.74, 129.75, 129.27, 128.23, 125.96, 120.55 (d, *J* = 2.2 Hz), 118.53, 116.59 (t, $^1J_{C-F}$ = 255.9 Hz), 79.17, 64.54, 60.97, 54.61, 45.11, 34.76, 32.49 (t, $^2J_{C-F}$ = 23.4 Hz), 25.81, 25.41 (t, $^3J_{C-F}$ = 3.6 Hz), 25.30, 25.23, 6.04. **^{19}F NMR** (376 MHz, $CDCl_3$) δ -101.58 (d, *J* = 257.6 Hz, 1F), -104.97 (d, *J* = 255.4 Hz, 1F). **HRMS** (ESI) calcd for $C_{28}H_{34}Cl_2F_2NO_6S$ [M + H]$^+$: 620.1446, found: 620.1445.

4-(Ethylsulfonyl)-2,2-difluoro-7-(((8*R*,9*S*,13*S*,14*S*)-13-methyl-17-oxo-7,8,9,11,12,13,14,15,16, 17-decahydro-6*H*-cyclopenta[a]phenanthren-3-yl)oxy)-*N*-phenylheptanamide (49)

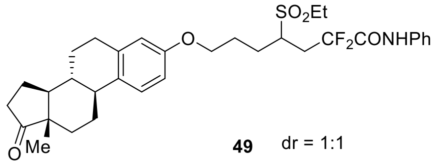

49

The title compound was prepared according to the ***General procedure I*** and purified by column chromatography (EA:PE = 0-30%) as a light yellow oil (161.3 mg, 67% yield). **^{1}H NMR** (400 MHz, $CDCl_3$) δ 8.43 (s, 1H), 7.56 (d, *J* = 8.0 Hz, 2H), 7.35 (t, *J* = 7.9 Hz, 2H), 7.23 – 7.11 (m, 2H), 6.67 (dd, *J* =

8.6, 2.7 Hz, 1H), 6.60 (d, *J* = 2.7 Hz, 1H), 3.95 (t, *J* = 5.3 Hz, 2H), 3.49 – 3.38 (m, 1H), 3.07 (q, *J* = 7.4 Hz, 2H), 3.01 – 2.92 (m, 1H), 2.87 (dt, *J* = 10.2, 3.8 Hz, 2H), 2.51 (td, *J* = 18.4, 9.8 Hz, 2H), 2.36 (dd, *J* = 10.0, 4.0 Hz, 1H), 2.31 – 2.17 (m, 2H), 2.17 – 2.10 (m, 1H), 2.11 – 1.89 (m, 7H), 1.69 – 1.52 (m, 2H), 1.52 – 1.43 (m, 3H), 1.39 (t, *J* = 7.4 Hz, 3H), 0.89 (s, 3H). **^{13}C NMR** (101 MHz, $CDCl_3$) δ 161.16 (t, $^2J_{C\text{-}F}$ = 28.2 Hz), 156.76, 137.83, 135.88, 132.26, 129.23, 126.39, 125.86, 120.57, 116.67 (t, $^1J_{C\text{-}F}$ = 256.1 Hz), 114.51, 112.14, 66.98, 54.85, 50.39, 48.06, 45.24, 43.96, 38.34, 35.91, 32.74 (t, $^3J_{C\text{-}F}$ = 23.9 Hz), 31.59, 29.65, 26.55, 26.07, 25.92, 25.84, 21.61, 13.88, 6.15. **^{19}F NMR** (376 MHz, $CDCl_3$) **Isomer 1** δ -101.79 (d, *J* = 255.7 Hz), -104.56 (d, *J* = 255.7 Hz); **Isomer 2** δ -101.82 (d, *J* = 255.7 Hz), -104.57 (d, *J* = 255.7 Hz). **HRMS** (ESI) calcd for $C_{33}H_{42}F_2NO_5S$ [M + H]$^+$: 602.2746, found: 602.2755.

(1*R*,2*S*,4*R*)-1,7,7-Trimethylbicyclo[2.2.1]heptan-2-yl 4-(ethylsulfonyl)-6,6-difluoro-7-oxo-7-(phenylamino)heptanoate (50)

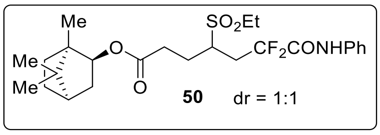

The title compound was prepared according to the ***General procedure I*** and purified by column chromatography (EA:PE = 0-20%) as a yellow oil (151.8 mg, 76% yield). **^{1}H NMR** (400 MHz, $CDCl_3$) δ 8.44 (s, 1H), 7.57 (d, *J* = 7.9 Hz, 2H), 7.34 (t, *J* = 7.9 Hz, 2H), 7.18 (t, *J* = 7.4 Hz, 1H), 4.93 – 4.78 (m, 1H), 3.44 (qd, *J* = 6.4, 2.7 Hz, 1H), 3.07 (qt, *J* = 7.3, 1.5 Hz, 2H), 3.00 – 2.81 (m, 1H), 2.80 – 2.54 (m, 2H), 2.50 – 2.19 (m, 3H), 2.18 – 2.03 (m, 1H), 1.98 – 1.84 (m, 1H), 1.78 – 1.60 (m, 2H), 1.37 (td, *J* = 7.4, 1.3 Hz, 1H, 3H), 1.33 – 1.15 (m, 2H), 1.00 – 0.89 (m, 1H), 0.90 – 0.81 (m, 6H), 0.79 (d, *J* = 9.9 Hz, 3H). **^{13}C NMR** (101 MHz, $CDCl_3$) δ 173.13, 173.11, 161.10 (t, $^2J_{C\text{-}F}$ = 28.1 Hz), 135.93, 129.23, 125.86, 120.55, 116.64 (t, $^1J_{C\text{-}F}$ = 255.9 Hz), 80.61, 80.55, 53.82, 53.79, 48.82, 48.76, 47.87, 47.84, 45.28, 44.87, 36.72, 36.69, 32.69 (t, $^2J_{C\text{-}F}$ = 22.4 Hz), 30.64, 30.61, 28.01, 27.08, 24.13, 19.73, 18.84, 13.52, 6.17. **^{19}F NMR** (376 MHz, $CDCl_3$) **Isomer 1** δ -101.03 (d, *J* = 255.7 Hz), -105.21 (d, *J* = 255.7 Hz); **Isomer 2** δ -100.98 (d, *J* = 255.7 Hz), -105.14 (d, *J* = 255.7 Hz). **HRMS** (ESI) calcd for $C_{25}H_{35}F_2NO_5SNa$ [M + Na]$^+$: 522.2096, found: 522.2099.

(5*S*)-4-(Ethylsulfonyl)-2,2-difluoro-5-hydroxy-7-((1*R*,2*S*,8a*R*)-2-hydroxy-2,5,5,8a-tetramethyldecahydronaphthalen-1-yl)-5-methyl-*N*-phenylheptanamide (51)

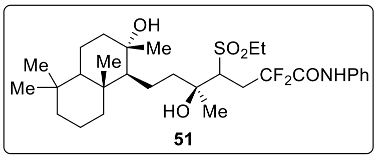

The title compound was prepared according to the ***General procedure I*** and purified by column chromatography (EA:PE = 0-30%) as a light yellow oil (160.6 mg, 70% yield). **^{1}H NMR** (400 MHz, $CDCl_3$) δ 8.29 (s, 1H), 7.59 (d, *J* = 7.9 Hz, 2H), 7.34 (t, *J* = 7.9 Hz, 2H), 7.17 (t, *J* = 7.4 Hz, 1H), 2.98 (q, *J* = 7.5 Hz, 2H), 2.38 – 2.19 (m, 2H), 1.86 – 1.77 (m, 1H), 1.70 – 1.50 (m, 8H), 1.48 – 1.33 (m, 6H), 1.31 – 1.28 (m, 1H), 1.27 – 1.19 (m, 2H),

1.18 – 1.12 (m, 6H), 1.11 – 1.00 (m, 1H), 0.96 – 0.81 (m, 6H), 0.79 – 0.71 (m, 6H). **^{13}C NMR** (101 MHz, $CDCl_3$) δ 162.52 (t, $^{2}J_{C\text{-}F}$ = 29.0 Hz), 136.25, 129.22, 125.61, 120.47, 118.69 (t, $^{1}J_{C\text{-}F}$ = 253.2 Hz), 75.00, 72.06, 61.87, 56.06, 46.25, 44.21, 44.08, 42.03, 39.64, 39.24, 34.80, 33.46, 33.29, 29.03 (t, $^{2}J_{C\text{-}F}$ = 23.4 Hz), 25.84, 24.22, 21.56, 20.52, 18.76, 18.47, 15.51, 6.68. **^{19}F NMR** (376 MHz, $CDCl_3$) δ -104.76 (d, *J* = 251.5 Hz, 1F), -106.04 (d, *J* = 251.4 Hz, 1F). **HRMS** (ESI) calcd for $C_{30}H_{47}F_2NO_5SNa$ [M + Na]$^+$: 594.3035, found: 594.3036.

(*E*)-3,7-Dimethylocta-2,6-dien-1-yl 4-(ethylsulfonyl)-6,6-difluoro-7-oxo-7-(phenylamino) heptanoate (52)

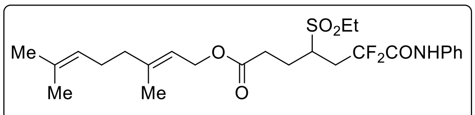

52

The title compound was prepared according to the ***General procedure I*** and purified by column chromatography (EA:PE = 0-20%) as a light yellow oil (107.6 mg, 54% yield). **^{1}H NMR** (400 MHz, $CDCl_3$) δ 8.33 (s, 1H), 7.58 (d, *J* = 8.0 Hz, 2H), 7.36 (t, *J* = 7.9 Hz, 2H), 7.26 – 7.10 (m, 1H), 5.31 (t, *J* = 7.3 Hz, 1H), 5.16 – 4.89 (m, 1H), 4.55 (d, *J* = 7.3 Hz, 2H), 3.51 – 3.37 (m, 1H), 3.18 – 3.02 (m, 2H), 2.01 – 2.82 (m, 1H), 2.81 – 2.67 (m, 1H), 2.63 – 2.52 (m, 1H), 2.49 – 2.26 (m, 2H), 2.20 – 1.97 (m, 5H), 1.74 (s, 3H), 1.67 (s, 3H), 1.59 (s, 3H), 1.38 (t, *J* = 7.5 Hz, 3H). **^{13}C NMR** (101 MHz, $CDCl_3$) δ 172.82, 161.07 (t, $^{2}J_{C\text{-}F}$ = 27.7 Hz), 142.99, 135.86, 132.36, 129.32, 125.96, 123.57, 120.50, 118.89, 116.66 (t, $^{1}J_{C\text{-}F}$ = 27.7 Hz), 61.57, 53.74, 45.30, 32.79 (t, $^{2}J_{C\text{-}F}$ = 255.9 Hz), 32.24, 30.56, 26.70, 25.81, 24.14, 23.62, 17.78, 6.20. **^{19}F NMR** (376 MHz, $CDCl_3$) δ -101.01 (d, *J* = 256.7 Hz, 1F), -105.27 (d, *J* = 256.9 Hz, 1F). **HRMS** (ESI) calcd for $C_{25}H_{35}F_2NNaO_5S$ [M + Na]$^+$: 522.2096, found: 522.2101.

4-((2*R*,8*R*,8a*S*)-8,8a-Dimethyl-6-oxo-1,2,3,4,6,7,8,8a-octahydronaphthalen-2-yl)-4-(ethylsulfonyl)-2,2-difluoro-*N*-phenylpentanamide (53)

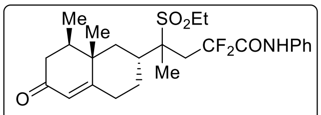

53 dr = 1:1

The title compound was prepared according to the ***General procedure I*** and purified by column chromatography (EA:PE = 0-30%) as a light yellow oil (121.7 mg, 63% yield). **^{1}H NMR** (400 MHz, $CDCl_3$) δ 8.52 (s, 1H), 7.57 (d, *J* = 8.2 Hz, 2H), 7.35 (t, *J* = 7.9 Hz, 2H), 7.18 (t, *J* = 7.4 Hz, 1H), 5.70 (s, 1H), 3.18 – 2.99 (m, 2H), 2.97 – 2.72 (m, 2H), 2.65 – 2.31 (m, 3H), 2.29 – 2.05 (m, 4H), 2.04 – 1.91 (m, 1H), 1.63 – 1.52 (m, 3H), 1.51 – 1.45 (m, 1H), 1.44 – 1.34 (m, 3H), 1.31 – 1.18 (m, 1H), 1.05 (d, *J* = 17.1 Hz, 3H), 0.94 (d, *J* = 6.7 Hz, 3H). **^{13}C NMR** (101 MHz, $CDCl_3$) δ 199.60, 199.53, 169.36, 161.49 (t, $^{2}J_{C\text{-}F}$ = 28.7 Hz), 135.96, 129.27, 125.89, 124.61, 124.59, 120.48, 117.46 (t, $^{1}J_{C\text{-}F}$ = 258.4), 117.38 (t, $^{1}J_{C\text{-}F}$ = 257.6), 66.87, 66.81, 43.71, 43.64, 41.96, 41.94, 40.42, 40.36, 39.68, 39.64, 39.58, 38.73, 35.18 (t, $^{2}J_{C\text{-}F}$ = 22.9 Hz), 34.55 (t, $^{2}J_{C\text{-}F}$ = 22.7 Hz), 32.97, 32.92, 28.24, 28.16, 18.65, 18.43, 16.60, 16.42, 15.05, 15.03, 5.22, 5.17. **^{19}F NMR** (376 MHz, $CDCl_3$) **Isomer 1**

δ -98.22 (d, *J* = 257.5 Hz), -100.38 (d, *J* = 257.5 Hz); **Isomer 2** δ -99.33 (d, *J* = 256.9 Hz), -100.04 (d, *J* = 256.9 Hz). **HRMS** (ESI) calcd for $C_{25}H_{34}F_2NO_4S$ $[M + H]^+$: 482.2171, found: 482.2169.

4-(Ethylsulfonyl)-2,2-difluoro-*N*,*N*-dimethyl-6-(naphthalen-2-yl)hexanamide (54)

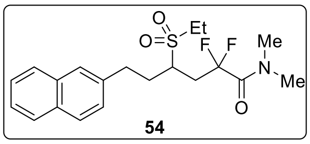

54

The title compound was prepared according to the ***General procedure I*** and purified by column chromatography (EA:PE = 0-20%) as a yellow oil (101.8 mg, 64% yield). **^{1}H NMR** (400 MHz, $CDCl_3$) δ 7.85 – 7.73 (m, 3H), 7.65 (s, 1H), 7.54 – 7.38 (m, 2H), 7.35 (d, *J* = 8.4 Hz, 1H), 3.45 – 3.33 (m, 1H), 3.20 – 2.98 (m, 7H), 2.97 – 2.89 (m, 1H), 2.86 (s, 3H), 2.58 – 2.38 (m, 2H), 2.32 – 2.10 (m, 1H), 1.39 (t, *J* = 7.4 Hz, 3H). **^{13}C NMR** (101 MHz, $CDCl_3$) δ 162.20 (t, $^2J_{C-F}$ = 28.6 Hz), 138.15, 133.59, 132.17, 128.13, 127.64, 127.56, 127.34, 126.89, 126.07, 125.45, 118.46 (t, $^1J_{C-F}$ = 257.4 Hz), 54.49, 54.45, 45.44, 36.88 (t, $^3J_{C-F}$ = 6.4 Hz), 34.26 (dd, *J* = 24.8, 22.5 Hz), 32.99, 30.28, 6.25. **^{19}F NMR** (376 MHz, $CDCl_3$) δ -94.72 (d, *J* = 284.9 Hz, 1F), -99.27 (d, *J* = 285.3 Hz, 1F). **HRMS** (ESI) calcd for $C_{20}H_{26}F_2NO_3S$ $[M + H]^+$: 398.1596, found: 398.1604.

4-(Ethylsulfonyl)-2,2-difluoro-1-morpholino-6-(naphthalen-2-yl)hexan-1-one (55)

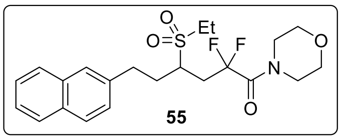

55

The title compound was prepared according to the ***General procedure I*** and purified by column chromatography (EA:PE = 0-30%) as a yellow oil (91.9 mg, 52% yield). **^{1}H NMR** (400 MHz, $CDCl_3$) δ 7.85 – 7.74 (m, 3H), 7.65 (s, 1H), 7.51 – 7.40 m, 2H), 7.34 (dd, *J* = 8.4, 1.8 Hz, 1H), 3.71 – 3.53 (m, 7H), 3.54 – 3.29 (m, 2H), 3.14 – 2.79 (m, 5H), 2.60 – 2.37 (m, 2H), 2.32 – 2.16 (m, 1H), 1.38 (t, *J* = 7.4 Hz, 3H). **^{13}C NMR** (101 MHz, $CDCl_3$) δ 160.87 (t, $^2J_{C-F}$ = 28.7 Hz), 138.03, 133.59, 132.18, 128.18, 127.66, 127.53, 127.28, 126.90, 126.13, 125.51, 118.50 (t, $^1J_{C-F}$ = 257.5 Hz), 66.64, 66.55, 54.32 (d, $^3J_{C-F}$ = 4.3 Hz), 46.43 (t, $^3J_{C-F}$ = 6.1 Hz), 45.41, 43.37, 34.15 (dd, $^2J_{C-F}$ = 24.5, 22.3 Hz), 32.95, 30.48, 6.26. **^{19}F NMR** (376 MHz, $CDCl_3$) δ -94.26 (d, *J* = 285.2 Hz, 1F), -99.06 (d, *J* = 285.1 Hz, 1F). **HRMS** (ESI) calcd for $C_{22}H_{28}F_2NO_4S$ $[M + H]^+$: 440.1702, found: 440.1703.

4-(Ethylsulfonyl)-2,2-difluoro-*N*-((1*S*,2*R*)-2-hydroxy-2,3-dihydro-1*H*-inden-1-yl)-6-(naphthalen-2-yl)hexanamide (56)

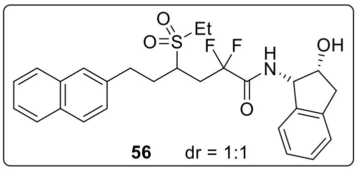

56

The title compound was prepared according to the ***General procedure I*** and purified by column chromatography (EA:PE = 0-30%) as a yellow oil (120.4mg, 60% yield). **^{1}H NMR** (400 MHz, $CDCl_3$) δ 7.83 – 7.69 (m, 3H), 7.64 (d, *J* = 4.5 Hz, 1H), 7.48 – 7.38 (m, 2H), 7.36 – 7.29 (m, 1H), 7.27 – 7.09 (m, 5H), 5.35 – 5.23 (m, 1H), 4.63 – 4.48 (m, 1H), 3.58 – 3.29 (m, 1H), 3.17 – 2.75 (m, 8H), 2.64 – 2.27 (m, 2H), 2.22 – 2.07 (m, 1H), 1.26 (td, *J* =

7.4, 2.4 Hz, 3H). **^{13}C NMR** (101 MHz, $CDCl_3$) δ 163.83 (t, $^2J_{C\text{-}F}$ = 39.6 Hz), 163.80 (t, $^2J_{C\text{-}F}$ = 28.6 Hz), 140.18, 140.09, 139.36, 137.70, 137.66, 133.59, 132.21, 128.63, 128.37, 127.69, 127.56, 127.54, 127.37, 127.36, 127.01, 126.84, 126.21, 125.59, 125.58, 125.46, 124.52, 124.42, 116.71 (t, $^1J_{C\text{-}F}$ = 253.1 Hz), 116.56 (t, $^1J_{C\text{-}F}$ = 255.3 Hz), 72.75, 57.66, 57.59, 54.91, 54.45, 45.21, 44.76, 39.79, 39.74, 33.17 (t, $^2J_{C\text{-}F}$ = 25.2 Hz), 32.88 (t, $^2J_{C\text{-}F}$ = 23.6 Hz), 32.57, 32.47, 31.14, 30.78, 6.05, 5.88. **^{19}F NMR** (376 MHz, $CDCl_3$) **Isomer 1** δ -98.55 (d, *J* = 258.2 Hz), -107.35 (d, *J* = 258.6 Hz); **Isomer 2** δ -102.59 (d, *J* = 255.0 Hz), -103.55 (d, *J* = 255.1 Hz). **HRMS** (ESI) calcd for $C_{27}H_{30}F_2NO_4S$ $[M + H]^+$: 502.1858, found: 502.1862.

Ethyl 4-(ethylsulfonyl)-2,2-difluoro-6-(naphthalen-2-yl)hexanoate (57)

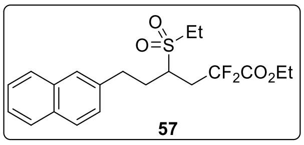

57

The title compound was prepared according to the ***General procedure I*** and purified by column chromatography (EA:PE = 0-20%) as a yellow oil (81.4 mg, 51% yield). **^{1}H NMR** (400 MHz, $CDCl_3$) δ 7.85 – 7.76 (m, 3H), 7.71 – 7.61 (m, 1H), 7.51 – 7.42 (m, 2H), 7.35 (dd, *J* = 8.4, 1.8 Hz, 1H), 4.32 (q, *J* = 7.1, 2H), 3.40 – 3.26 (m, 1H), 3.18 – 3.04 (m, 1H), 2.98 (q, *J* = 7.4, 3H), 2.94 – 2.79 (m, 1H), 2.62 – 2.35 (m, 2H), 2.24 – 2.13 (m, 1H), 1.40 – 1.28 (m, 6H). **^{13}C NMR** (101 MHz, $CDCl_3$) δ 163.31 (t, $^2J_{C\text{-}F}$ = 32.1 Hz), 137.68, 133.69, 132.33, 128.47, 127.76, 127.63, 127.06, 126.93, 126.28, 125.66, 114.88 (t, $^1J_{C\text{-}F}$ = 252.4 Hz), 63.69, 54.22, 45.40, 33.42 (t, $^2J_{C\text{-}F}$ = 23.7 Hz), 32.59, 30.85, 14.00, 6.24. **^{19}F NMR** (376 MHz, $CDCl_3$) δ -102.60 (d, *J* = 262.3 Hz, 1F), -105.56 (d, *J* = 262.2 Hz, 1F). **HRMS** (ESI) calcd for $C_{20}H_{25}F_2O_4S$ $[M + H]^+$: 399.1436, found: 399.1435.

2-(3-(Ethylsulfonyl)-4,4-difluoro-4-tosylbutyl)naphthalene (58)

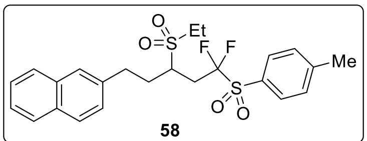

58

The title compound was prepared according to the ***General procedure I*** and purified by column chromatography (EA:PE = 0-30%) as a light yellow oil (124.8 mg, 65% yield). **^{1}H NMR** (400 MHz, $CDCl_3$) δ 7.92 – 7.71 (m, 5H), 7.66 (d, *J* = 1.8 Hz, 1H), 7.53 – 7.39 (m, 4H), 7.34 (dd, *J* = 8.4, 1.8 Hz, 1H), 3.56 – 3.44 (m, 1H), 3.31 – 2.90 (m, 5H), 2.85 – 2.65 (m, 1H), 2.60 – 2.44 (m, 4H), 2.33 – 2.18 (m, 1H), 1.38 (t, *J* = 7.4 Hz, 3H). **^{13}C NMR** (101 MHz, $CDCl_3$) δ 147.59, 137.69, 133.65, 132.29, 130.98, 130.34, 128.39, 128.33, 127.72, 127.60, 127.07, 126.88, 126.19, 125.58, 123.09 (t, $^1J_{C\text{-}F}$ = 287.5 Hz), 54.02, 45.65, 32.61, 30.46, 29.19 (t, $^2J_{C\text{-}F}$ = 20.4 Hz), 21.99, 6.22. **^{19}F NMR** (376 MHz, $CDCl_3$) δ -99.73 (d, *J* = 229.8 Hz, 1F), -102.91 (d, *J* = 230.1 Hz, 1F). **HRMS** (ESI) calcd for $C_{24}H_{27}F_2O_4S_2$ $[M + H]^+$: 481.1313, found: 481.1325.

2-(3-(Ethylsulfonyl)-1,1-difluoro-5-(naphthalen-2-yl)pentyl)-6-methylbenzo[*d*]oxazole (59)

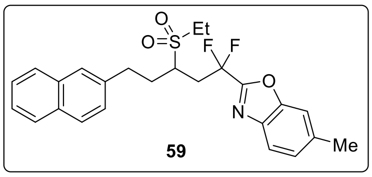

The title compound was prepared according to the ***General procedure I*** and purified by column chromatography (EA:PE = 0-20%) as a yellow oil (84.4 mg, 46% yield). **^{1}H NMR** (400 MHz, $CDCl_3$) δ 7.80 – 7.68 (m, 3H), 7.63 (d, *J* = 8.2 Hz, 1H), 7.61 – 7.57 (m, 1H), 7.47 – 7.38 (m, 2H), 7.37 (dt, *J* = 1.6, 0.7 Hz, 1H), 7.32 (d, *J* = 1.8 Hz, 1H), 7.28 – 7.20 (m, 1H), 3.58 – 3.46 (m, 1H), 3.36 – 3.19 (m, 1H), 3.14 – 2.96 (m, 4H), 2.93 – 2.77 (m, 1H), 2.58 – 2.46 (m, 4H), 2.34 – 2.21 (m, 1H), 1.37 (t, *J* = 7.4 Hz, 3H). **^{13}C NMR** (101 MHz, $CDCl_3$) δ 156.14 (t, $^2J_{C\text{-F}}$ = 32.9 Hz), 150.97, 138.17, 137.76, 137.50, 133.57, 132.19, 128.27, 127.66, 127.51, 127.04, 127.03, 126.85, 126.10, 125.45, 120.60, 115.71 (t, $^1J_{C\text{-F}}$ = 243.1 Hz), 111.54, 54.27, 45.51, 35.09 (t, $^2J_{C\text{-F}}$ = 24.0 Hz), 32.69, 30.30, 21.96, 6.34. **^{19}F NMR** (376 MHz, $CDCl_3$) δ -94.05 (d, *J* = 277.4 Hz, 1F), -97.25 (d, *J* = 277.6 Hz, 1F). **HRMS** (ESI) calcd for $C_{25}H_{25}F_2NNaO_3S$ $[M + Na]^+$: 480.1415, found: 480.1420.

2-(6-Bromo-3-(ethylsulfonyl)-5,5,6,6-tetrafluorohexyl)naphthalene (60)

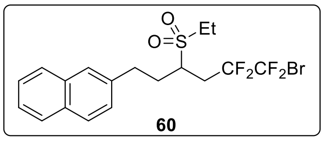

The title compound was prepared according to the ***General procedure I*** and purified by column chromatography (EA:PE = 0-20%) as a light yellow oil (110.9mg, 61% yield). **^{1}H NMR** (400 MHz, $CDCl_3$) δ 7.87 – 7.75 (m, 3H), 7.72 – 7.63 (m, 1H), 7.52 – 7.42 (m, 2H), 7.35 (dd, *J* = 8.4, 1.8 Hz, 1H), 3.42 – 3.24 (m, 1H), 3.17 – 3.06 (m, 1H), 2.99 (q, *J* = 7.5 Hz, 2H), 3.00 – 2.83 (m, 2H), 2.65 – 2.41 (m, 2H), 2.30 – 2.11 (m, 1H), 1.35 (t, *J* = 7.4 Hz, 3H). **^{13}C NMR** (101 MHz, $CDCl_3$) δ 137.44, 133.68, 132.35, 128.55, 127.77, 127.61, 126.95, 126.90, 126.34, 125.72, 120.67 – 113.49 (m), 53.84 (d, *J* = 1.8 Hz), 45.60, 32.48 (d, *J* = 1.6 Hz), 30.67 (d, *J* = 1.5 Hz), 29.47 (t, *J* = 22.0 Hz), 6.25. **^{19}F NMR** (376 MHz, $CDCl_3$) δ -66.25 (q, *J* = 4.5, 3.9 Hz, 2F), -103.45 – -119.21 (m, 2F). **HRMS** (ESI) calcd for $C_{18}H_{20}BrF_4O_2S$ $[M + H]^+$: 455.0298, found: 455.0309.

2-(3-(Ethylsulfonyl)-5,5,6,6,7,7,8,8,8-nonafluorooctyl)naphthalene (61)

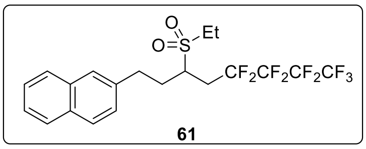

The title compound was prepared according to the ***General procedure I*** and purified by column chromatography (EA:PE = 0-10%) as a yellow oil (83.1mg, 42% yield). **^{1}H NMR** (500 MHz, $CDCl_3$) δ 7.88 – 7.75 (m, 3H), 7.67 (d, *J* = 1.7 Hz, 1H), 7.52 – 7.42 (m, 2H), 7.35 (dd, *J* = 8.4, 1.8 Hz, 1H), 3.46 – 3.31 (m, 1H), 3.16 – 3.06 (m, 1H), 3.05 – 2.84 (m, 4H), 2.62 – 2.43 (m, 2H), 2.37 – 2.12 (m, 1H), 1.35 (t, *J* = 7.5 Hz, 3H). **^{13}C NMR** (126 MHz, $CDCl_3$) δ 137.34, 133.68, 132.37, 128.57, 127.77, 127.59, 126.89, 126.36, 125.74, 120.61 – 107.58 (m), 53.45 (d, *J* = 2.2 Hz), 45.58, 32.47, 30.85, 29.72 (t, $^2J_{C\text{-}F}$ = 21.4 Hz), 6.21. **^{19}F NMR** (471 MHz, $CDCl_3$) δ -80.96 (t, *J* = 9.4 Hz),

-109.48 – -116.15 (m), -120.27 – -128.58 (m). **HRMS** (ESI) calcd for $C_{20}H_{20}F_9O_2S$ $[M + H]^+$: 495.1035, found: 495.1045.

4-(Ethylsulfonyl)-2,2-difluoro-6-(naphthalen-2-yl)hexan-1-ol (62)

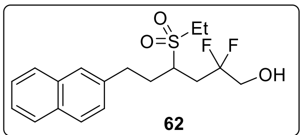

The title compound was prepared according to the ***General procedure I*** and purified by column chromatography (EA:PE = 0-30%) as a light yellow oil (70.1 mg, 49% yield). **^{1}H NMR** (400 MHz, $CDCl_3$) δ 7.87 – 7.73 (m, 3H), 7.66 (s, 1H), 7.54 – 7.41 (m, 2H), 7.34 (dd, J = 8.5, 1.8 Hz, 1H), 3.87 – 3.69 (m, 2H), 3.36 – 3.23 (m, 1H), 3.17 – 3.06 (m, 1H), 3.04 – 2.89 (m, 3H), 2.88 – 2.78 (m, 1H), 2.77 – 2.65 (m, 1H), 2.49 – 2.24 (m, 2H), 2.20 – 2.07 (m, 1H), 1.31 (t, J = 7.4 Hz, 3H). **^{13}C NMR** (101 MHz, $CDCl_3$) δ 137.71, 133.68, 132.31, 128.47, 127.77, 127.61, 127.03, 126.94, 126.31, 125.68, 122.27 (t, $^1J_{C\text{-}F}$ = 245.2 Hz), 63.91 (t, $^2J_{C\text{-}F}$ = 32.2 Hz), 54.78 (t, $^3J_{C\text{-}F}$ = 3.2 Hz), 45.00, 32.49, 32.01 (t, $^2J_{C\text{-}F}$ = 24.5 Hz), 31.49, 6.16. **^{19}F NMR** (376 MHz, $CDCl_3$) δ -103.71 (d, J = 253.1 Hz, 1F), -107.82 (d, J = 252.9 Hz, 1F). **HRMS** (ESI) calcd for $C_{18}H_{22}F_2NaO_3S$ $[M + Na]^+$: 379.1150, found: 379.1147.

Ethyl 4-(ethylsulfonyl)-2,2-dimethyl-6-(naphthalen-2-yl)hexanoate (63)

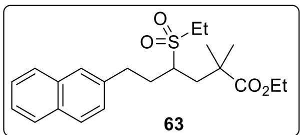

The title compound was prepared according to the ***General procedure I*** and purified by column chromatography (EA:PE = 0-20%) as a light yellow oil (73.3mg, 47% yield). **^{1}H NMR** (400 MHz, $CDCl_3$) δ 7.85 – 7.75 (m, 3H), 7.67 – 7.62 (m, 1H), 7.50 – 7.38 (m, 2H), 7.33 (dd, J = 8.4, 1.8 Hz, 1H), 4.20 – 3.97 (m, 2H), 3.15 – 2.88 (m, 5H), 2.37 – 2.18 (m, 2H), 2.12 – 1.90 (m, 1H), 1.93 – 1.80 (m, 1H), 1.40 (t, J = 7.5 Hz, 3H), 1.24 (s, 3H), 1.22 (s, 3H), 1.19 (t, J = 7.1 Hz, 3H). **^{13}C NMR** (101 MHz, $CDCl_3$) δ 177.02, 138.45, 133.70, 132.26, 128.34, 127.76, 127.58, 127.23, 126.76, 126.22, 125.55, 61.10, 58.24, 44.53, 41.93, 38.76, 33.57, 31.70, 26.00, 25.85, 14.18, 6.10. **HRMS** (ESI) calcd for $C_{22}H_{30}NaO_4S$ $[M + Na]^+$: 413.1757, found: 413.1760.

Methyl (4-(ethylsulfonyl)-2,2-difluoro-6-(naphthalen-2-yl)hexanoyl)-*D*-tryptophanate (64)

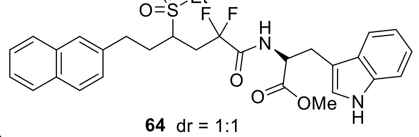

The title compound was prepared according to the ***General procedure I*** and purified by column chromatography (EA:PE = 0-30%) as a light yellow oil (153.2mg, 67% yield). **^{1}H NMR** (400 MHz, $CDCl_3$) δ 8.25 (d, J = 12.3 Hz, 1H), 7.83 – 7.73 (m, 3H), 7.63 (d, J = 2.4 Hz, 1H), 7.56 – 7.41 (m, 3H), 7.38 – 7.27 (m, 2H), 7.24 – 7.09 (m, 2H), 7.02 – 6.89 (m, 2H), 5.02 – 4.82 (m, 1H), 3.73 (s, 3H), 3.44 – 3.12 (m, 3H), 3.09 – 2.66 (m, 5H), 2.57 – 2.30 (m, 2H), 2.24 – 2.07 (m, 1H), 1.36 – 1.25 (m, 3H). **^{13}C NMR** (101 MHz, $CDCl_3$) δ 171.18, 171.09, 162.94 (t, $^2J_{C\text{-}F}$ = 30.0 Hz), 162.86 (t, $^2J_{C\text{-}F}$ = 29.0 Hz), 137.98, 137.95,

136.21, 136.19, 133.62, 132.21, 128.28, 128.25, 127.70, 127.58, 127.36, 127.25, 127.12, 126.81, 126.19, 126.17, 125.55, 123.11, 123.06, 122.52, 122.49, 119.92, 118.34, 118.28, 116.54 (t, $^{1}J_{C\text{-}F}$ = 255.6 Hz), 111.60, 109.15, 109.06, 54.42, 54.30, 53.13, 53.02, 52.83, 45.31, 45.17, 32.99 (t, $^{2}J_{C\text{-}F}$ = 23.5 Hz), 32.82 (t, $^{2}J_{C\text{-}F}$ = 22.9 Hz), 32.50, 32.38, 30.36, 30.31, 27.46, 27.17, 6.04, 5.94. **^{19}F NMR** (376 MHz, $CDCl_3$) **Isomer 1** δ -101.86 (d, *J* = 120.4 Hz), -106.27 (d, *J* = 166.2 Hz); **Isomer 2** δ -102.55 (d, *J* = 120.6 Hz), -105.59 (d, *J* = 166.9 Hz). **HRMS** (ESI) calcd for $C_{30}H_{32}F_2N_2NaO_5S$ [M + Na]$^+$: 593.1892, found: 593.1909.

Methyl (3*R*)-3-(4-(ethylsulfonyl)-2,2-difluoro-6-(naphthalen-2-yl)hexanamido)-4-(((*R*)-1-methoxy-1-oxo-3-phenylpropan-2-yl)amino)-4-oxobutanoate (65)

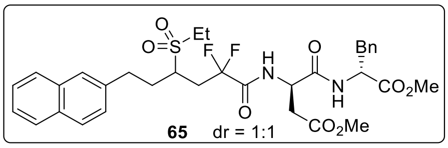

The title compound was prepared according to the ***General procedure I*** and purified by column chromatography (EA:PE = 0-30%) as a yellow foam (174.2mg, 66% yield). **^{1}H NMR** (500 MHz, $CDCl_3$) δ 7.80 (dp, *J* = 11.7, 4.2 Hz, 3H), 7.70 – 7.60 (m, 2H), 7.50 – 7.40 (m, 2H), 7.35 (dd, *J* = 8.4, 1.8 Hz, 1H), 7.32 – 7.24 (m, 2H), 7.25 – 7.18 (m, 1H), 7.17 – 7.10 (m, 2H), 7.04 (dd, *J* = 7.9, 4.1 Hz, 1H), 4.81 – 4.72 (m, 2H), 3.70 (s, 3H), 3.68 (s, 3H), 3.44 – 3.29 (m, 1H), 3.23 – 2.78 (m, 8H), 2.69 – 2.38 (m, 3H), 2.25 – 2.05 (m, 1H), 1.31 (t, *J* = 7.4 Hz, 3H). **^{13}C NMR** (126 MHz, $CDCl_3$) δ 172.34, 172.29, 171.52, 171.46, 168.92, 168.87, 163.19 (t, $^{2}J_{C\text{-}F}$ = 30.8 Hz), 137.66, 135.79, 135.66, 133.66, 132.30, 129.26, 128.78, 128.76, 128.45, 127.75, 127.61, 127.33, 127.30, 127.04, 126.92, 126.90, 126.28, 125.65, 116.32 (t, $^{1}J_{C\text{-}F}$ = 250.1 Hz), 54.80, 54.42, 53.87, 53.83, 52.54, 52.53, 52.48, 52.45, 49.12, 49.06, 45.31, 44.96, 37.54, 37.47, 35.38, 35.27, 32.99 (t, $^{2}J_{C\text{-}F}$ = 24.8 Hz), 32.75 (t, $^{2}J_{C\text{-}F}$ = 21.3 Hz), 32.61, 31.04, 30.85, 6.06, 5.98. **^{19}F NMR** (471 MHz, $CDCl_3$) **Isomer 1** δ -100.69 (d, *J* = 919.9 Hz), -105.68 (d, *J* = 979.7 Hz); **Isomer 2** δ -101.24 (d, *J* = 918.1 Hz), -105.12 (d, *J* = 977.9 Hz). **HRMS** (ESI) calcd for $C_{33}H_{39}F_2N_2O_8S$ [M + H]$^+$: 661.2390, found: 661.2385.

4-(Butylsulfonyl)-2,2-difluoro-4-methyl-*N*-phenylpentanamide (66)

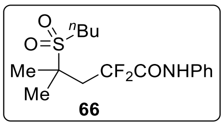

The title compound was prepared according to the ***General procedure I*** and purified by column chromatography (EA:PE = 0-20%) as a light yellow oil (125.7 mg, 91% yield). **^{1}H NMR** (400 MHz, $CDCl_3$) δ 8.29 (s, 1H), 7.56 (d, *J* = 8.4 Hz, 2H), 7.34 (t, *J* = 7.4 Hz, 2H), 7.18 (t, *J* = 7.4 Hz, 1H), 2.98 – 2.89 (m, 2H), 2.85 – 2.62 (m, 2H), 1.91 – 1.74 (m, 2H), 1.55 (d, *J* = 1.7 Hz, 6H), 1.51 – 1.42 (m, 2H), 0.94 (td, *J* = 7.3, 1.4 Hz, 3H). **^{13}C NMR** (101 MHz, $CDCl_3$) δ 161.54 (t, $^{2}J_{C\text{-}F}$ = 28.0 Hz), 135.91, 129.25, 125.90, 120.60, 117.83 (t, $^{1}J_{C\text{-}F}$ = 257.3 Hz), 60.71, 45.39, 37.40 (t, $^{2}J_{C\text{-}F}$ = 22.4 Hz), 22.58, 22.05, 20.78 (t, $^{3}J_{C\text{-}F}$ = 2.4 Hz), 13.67. **^{19}F NMR** (376 MHz, $CDCl_3$) δ -101.64 (s, 2F). **HRMS** (ESI) calcd for

$C_{16}H_{24}F_2NO_3S$ [M + H]$^+$: 348.1439, found: 348.1447.

2,2-Difluoro-4-methyl-4-(methylsulfonyl)-*N*-phenylpentanamide (67)

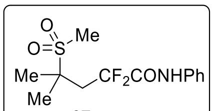
67

The title compound was prepared according to the ***General procedure I*** and purified by column chromatography (EA:PE = 0-30%) as a light yellow oil (108.7 mg, 89% yield). **^{1}H NMR** (400 MHz, $CDCl_3$) δ 8.16 (s, 1H), 7.56 (d, *J* = 7.7 Hz, 2H), 7.37 (t, *J* = 7.9 Hz, 2H), 7.20 (t, *J* = 7.4 Hz, 1H), 2.85 (s, 3H), 2.75 (t, *J* = 18.5 Hz, 2H), 1.58 (s, 6H). **^{13}C NMR** (101 MHz, $CDCl_3$) δ 161.41 (t, $^2J_{C-F}$ = 28.3 Hz), 135.83, 129.39, 126.02, 120.54, 117.83 (t, $^1J_{C-F}$ = 257.0 Hz), 60.52, 37.37 (t, $^2J_{C-F}$ = 22.5 Hz), 34.27, 20.93 (t, $^3J_{C-F}$ = 2.3 Hz). **^{19}F NMR** (376 MHz, $CDCl_3$) δ -101.80 (s, 2F). **HRMS** (ESI) calcd for $C_{13}H_{18}F_2NO_3S$ [M + H]$^+$: 306.0970, found: 306.0979.

4-((Cyclopropylmethyl)sulfonyl)-2,2-difluoro-4-methyl-*N*-phenylpentanamide (68)

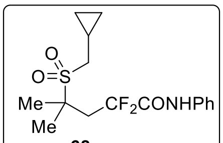
68

The title compound was prepared according to the ***General procedure I*** and purified by column chromatography (EA:PE = 0-20%) as a yellow oil (95.3 mg, 69% yield). **^{1}H NMR** (400 MHz, $CDCl_3$) δ 8.24 (s, 1H), 7.56 (d, *J* = 9.2 Hz, 2H), 7.36 (t, *J* = 7.9 Hz, 2H), 7.19 (t, *J* = 7.4 Hz, 1H), 2.92 (d, *J* = 7.1 Hz, 2H), 2.75 (t, *J* = 18.5 Hz, 2H), 1.56 (s, 6H), 1.25 – 1.14 (m, 1H), 0.83 – 0.65 (m, 2H), 0.42 (dt, *J* = 6.6, 5.0 Hz, 2H). **^{13}C NMR** (101 MHz, $CDCl_3$) δ 161.53 (t, $^2J_{C-F}$ = 28.1 Hz), 135.88, 129.32, 125.95, 120.56, 117.86 (t, $^1J_{C-F}$ = 257.3 Hz), 60.73, 51.75, 37.47 (t, $^2J_{C-F}$ = 22.4 Hz), 20.84 (t, $^3J_{C-F}$ = 2.3 Hz), 4.91, 3.46. **^{19}F NMR** (376 MHz, $CDCl_3$) δ -101.68 (s, 2F). **HRMS** (ESI) calcd for $C_{16}H_{22}F_2NO_3S$ [M + H]$^+$: 346.1283, found: 346.1282.

(*S*)-4-((3,7-Dimethyloct-6-en-1-yl)sulfonyl)-2,2-difluoro-4-methyl-*N*-phenylpentanamide (69)

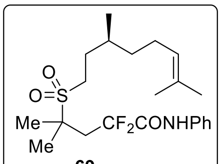
69

The title compound was prepared according to the ***General procedure I*** and purified by column chromatography (EA:PE = 0-10%) as a light yellow oil (70.3 mg, 41% yield). **^{1}H NMR** (400 MHz, $CDCl_3$) δ 8.08 (s, 1H), 7.57 (d, *J* = 8.0 Hz, 2H), 7.38 (t, *J* = 7.9 Hz, 2H), 7.21 (t, *J* = 7.4 Hz, 1H), 5.43 – 4.77 (m, 1H), 3.07 – 2.82 (m, 2H), 2.76 (t, *J* = 18.6 Hz, 2H), 2.12 – 1.82 (m, 3H), 1.87 – 1.64 (m, 4H), 1.63 – 1.50 (m, 10H), 1.42 – 1.26 (m, 1H), 1.26 – 1.03 (m, 1H), 0.94 (d, *J* = 6.6 Hz, 3H). **^{13}C NMR** (126 MHz, $CDCl_3$) δ 161.45 (t, $^2J_{C-F}$ = 27.9 Hz), 135.82, 131.87, 129.41, 126.02, 124.26, 120.48, 117.92 (t, $^1J_{C-F}$ = 257.4 Hz), 60.83, 43.81, 37.51 (t, $^2J_{C-F}$ = 22.3 Hz), 36.68, 32.01, 27.07, 25.85, 25.41, 20.99, 19.23, 17.81. **^{19}F NMR** (376 MHz, $CDCl_3$) δ -101.75 (s, 2F). **HRMS** (ESI) calcd for $C_{22}H_{34}F_2NO_3S$ [M + H]$^+$: 430.2222, found: 430.2276.

2,2-Difluoro-4-methyl-*N*-phenyl-4-((5-phenylpent-4-yn-1-yl)sulfonyl)pentanamide (70)

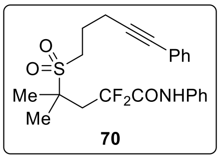

The title compound was prepared according to the ***General procedure I*** and purified by column chromatography (EA:PE = 0-10%) as a brown oil (107.1 mg, 62% yield). **^{1}H NMR** (400 MHz, $CDCl_3$) δ 8.05 (s, 1H), 7.47 (d, *J* = 8.0 Hz, 2H), 7.35 – 7.22 (m, 4H), 7.24 – 7.12 (m, 3H), 7.11 (t, *J* = 7.4 Hz, 1H), 3.08 (dd, *J* = 9.1, 6.4 Hz, 2H), 2.69 (t, *J* = 18.5 Hz, 2H), 2.56 (t, *J* = 6.7 Hz, 2H), 2.11 (p, *J* = 6.9 Hz, 2H), 1.51 (s, 6H). **^{13}C NMR** (101 MHz, $CDCl_3$) δ 161.38 (t, $^2J_{C-F}$ = 28.0 Hz), 135.81, 131.70, 129.38, 128.40, 128.09, 125.99, 123.30, 120.47, 117.85 (t, $^2J_{C-F}$ = 258.2 Hz), 87.41, 82.55, 61.01, 44.72, 37.35 (t, $^2J_{C-F}$ = 22.2 Hz), 20.93, 20.21, 18.76. **^{19}F NMR** (376 MHz, $CDCl_3$) δ -101.79 (s, 2F). **HRMS** (ESI) calcd for $C_{23}H_{25}F_2NNaO_3S$ [M + Na]$^+$: 456.1415, found: 456.1419.

4-((2-((*tert*-Butyldimethylsilyl)oxy)ethyl)sulfonyl)-2,2-difluoro-4-methyl-*N*-phenylpentanamide (71)

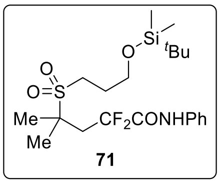

The title compound was prepared according to the ***General procedure I*** and purified by column chromatography (EA:PE = 0-10%) as a light yellow oil (142.1 mg, 79% yield). **^{1}H NMR** (400 MHz, $CDCl_3$) δ 8.14 (s, 1H), 7.56 (d, *J* = 8.0 Hz, 2H), 7.37 (t, *J* = 7.9 Hz, 2H), 7.20 (t, *J* = 7.4 Hz, 1H), 3.75 (t, *J* = 5.8 Hz, 2H), 3.19 – 2.98 (m, 2H), 2.76 (t, *J* = 18.5 Hz, 2H), 2.13 – 2.03 (m, 2H), 1.58 (s, 6H), 0.88 (s, 9H), 0.06 (s, 6H). **^{13}C NMR** (101 MHz, $CDCl_3$) δ 161.44 (t, $^2J_{C-F}$ = 28.0 Hz), 135.91, 129.36, 125.95, 120.51, 117.91 (t, $^1J_{C-F}$ = 257.3 Hz), 61.14, 60.87, 42.78, 37.42 (t, $^2J_{C-F}$ = 22.4 Hz), 25.97, 24.26, 20.97 (t, $^3J_{C-F}$ = 2.2 Hz), 18.33, -5.27. **^{19}F NMR** (376 MHz, $CDCl_3$) δ -101.68 (s, 2F). **HRMS** (ESI) calcd for $C_{21}H_{36}F_2NO_4SSi$ [M + H]$^+$: 464.2097, found: 464.2094.

2,2-Difluoro-4-methyl-4-((4-oxo-4-phenylbutyl)sulfonyl)-*N*-phenylpentanamide (72)

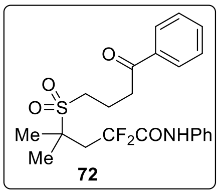

The title compound was prepared according to the ***General procedure I*** and purified by column chromatography (EA:PE = 0-20%) as a light yellow oil (120.8 mg, 69% yield). **^{1}H NMR** (400 MHz, $CDCl_3$) δ 8.14 (s, 1H), 8.00 – 7.87 (m, 2H), 7.56 (d, *J* = 7.4 Hz, 3H), 7.46 (dd, *J* = 8.4, 7.0 Hz, 2H), 7.37 (t, *J* = 7.9 Hz, 2H), 7.23 – 7.16 (m, 1H), 3.27 (t, *J* = 6.6 Hz, 2H), 3.15 (t, *J* = 7.2 Hz, 2H), 2.77 (t, *J* = 18.5 Hz, 2H), 2.37 (p, *J* = 6.9 Hz, 2H), 1.59 (s, 6H). **^{13}C NMR** (101 MHz, $CDCl_3$) δ 198.83, 161.42 (t, $^2J_{C-F}$ = 28.0 Hz), 136.58, 135.88, 133.53, 129.39, 128.82, 128.14, 125.98, 120.49, 117.87 (t, $^1J_{C-F}$ = 257.8 Hz), 61.07, 44.82, 37.43 (t, $^2J_{C-F}$ = 22.3 Hz), 36.64, 20.94 (t, $^3J_{C-F}$ = 2.3 Hz), 15.96. **^{19}F NMR** (376 MHz, $CDCl_3$) δ -101.74 (s, 2F). **HRMS** (ESI) calcd for $C_{22}H_{26}F_2NO_4S$ [M + H]$^+$: 438.1545, found: 438.1551.

4-((2-(1,3-Dioxolan-2-yl)ethyl)sulfonyl)-2,2-difluoro-4-methyl-*N*-phenylpentanamide (73)

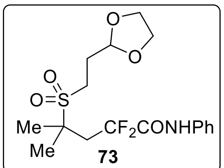

73

The title compound was prepared according to the ***General procedure I*** and purified by column chromatography (EA:PE = 0-20%) as a light yellow oil (136.2 mg, 87% yield). **^{1}H NMR** (400 MHz, $CDCl_3$) δ 8.24 (s, 1H), 7.55 (d, *J* = 7.8 Hz, 2H), 7.36 (t, *J* = 7.9 Hz, 2H), 7.19 (t, *J* = 7.4 Hz, 1H), 5.03 (t, *J* = 3.9 Hz, 1H), 4.07 – 3.67 (m, 4H), 3.17 – 2.85 (m, 2H), 2.74 (t, *J* = 18.6 Hz, 2H), 2.23 (dt, *J* = 11.9, 4.1 Hz, 2H), 1.57 (s, 6H). **^{13}C NMR** (101 MHz, $CDCl_3$) δ 161.45 (t, $^2J_{C-F}$ = 28.0 Hz), 135.85, 129.31, 125.94, 120.54, 117.83 (t, $^1J_{C-F}$ = 257.4 Hz), 101.99, 65.21, 60.99, 40.31, 37.36 (t, $^2J_{C-F}$ = 22.4 Hz), 24.88, 20.82 (t, $^3J_{C-F}$ = 2.2 Hz). **^{19}F NMR** (376 MHz, $CDCl_3$) δ -101.87 (s, 2F). **HRMS** (ESI) calcd for $C_{17}H_{24}F_2NO_5S$ [M + H]$^+$: 392.1338, found: 392.1338.

Ethyl 3-((4,4-difluoro-2-methyl-5-oxo-5-(phenylamino)pentan-2-yl)sulfonyl)propanoate (74)

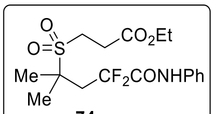

74

The title compound was prepared according to the ***General procedure I*** and purified by column chromatography (EA:PE = 0-20%) as a light yellow oil (97.1 mg, 62% yield). **^{1}H NMR** (400 MHz, $CDCl_3$) δ 8.18 (s, 1H), 7.56 (d, *J* = 7.6 Hz, 2H), 7.36 (t, *J* = 8.0 Hz, 2H), 7.19 (t, *J* = 7.4 Hz, 1H), 4.18 (q, *J* = 7.1 Hz, 2H), 3.30 (dd, *J* = 8.2, 7.1 Hz, 2H), 2.87 (dd, *J* = 8.2, 7.1 Hz, 2H), 2.76 (t, *J* = 18.5 Hz, 2H), 1.59 (d, *J* = 1.3 Hz, 6H), 1.26 (t, *J* = 7.1 Hz, 3H). **^{13}C NMR** (101 MHz, $CDCl_3$) δ 170.64, 161.35 (t, $^2J_{C-F}$ = 28.1 Hz), 135.88, 129.36, 125.97, 120.52, 117.80 (t, $^1J_{C-F}$ = 257.4 Hz), 61.63, 61.10, 41.53, 37.29 (t, $^2J_{C-F}$ = 22.5 Hz), 25.84, 20.85 (t, $^3J_{C-F}$ = 2.4 Hz), 14.21. **^{19}F NMR** (376 MHz, $CDCl_3$) δ -101.73 (s, 2F). **HRMS** (ESI) calcd for $C_{17}H_{24}F_2NO_5S$ [M + H]$^+$: 392.1338, found: 392.1343.

4-(Benzylsulfonyl)-2,2-difluoro-4-methyl-*N*-phenylpentanamide (75)

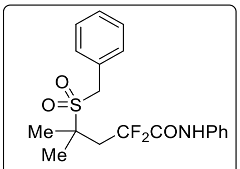

75

The title compound was prepared according to the ***General procedure I*** and purified by column chromatography (EA:PE = 0-20%) as a light yellow oil (100.7 mg, 66% yield). **^{1}H NMR** (400 MHz, $CDCl_3$) δ 8.14 (s, 1H), 7.55 (d, *J* = 8.2 Hz, 2H), 7.46 – 7.32 (m, 7H), 7.21 (t, *J* = 7.4 Hz, 1H), 4.24 (s, 2H), 2.78 (t, *J* = 18.4 Hz, 2H), 1.61 (s, 6H). **^{13}C NMR** (101 MHz, $CDCl_3$) δ 161.49 (t, $^2J_{C-F}$ = 28.1 Hz), 135.87, 131.47, 129.34, 129.10, 128.93, 126.48, 125.97, 120.60, 117.79 (t, $^1J_{C-F}$ = 257.2 Hz), 61.79, 52.82, 37.80 (t, $^2J_{C-F}$ = 22.5 Hz), 21.33 (t, $^3J_{C-F}$ = 2.4 Hz). **^{19}F NMR** (376 MHz, $CDCl_3$) δ -101.44 (s, 2F). **HRMS** (ESI) calcd for $C_{19}H_{22}F_2NO_3S$ [M + H]$^+$: 382.1283, found: 382.1282.

2,2-Difluoro-4-methyl-4-((naphthalen-1-ylmethyl)sulfonyl)-*N*-phenylpentanamide (76)

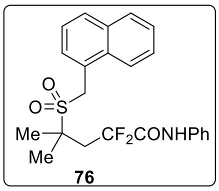

The title compound was prepared according to the ***General procedure I*** and purified by column chromatography (EA:PE = 0-20%) as a yellow foam (122.5 mg, 71% yield). **^{1}H NMR** (400 MHz, $CDCl_3$) δ 7.90 (s, 1H), 7.80 (s, 1H), 7.78 – 7.68 (m, 3H), 7.48 – 7.37 (m, 5H), 7.28 (t, *J* = 7.9 Hz, 2H), 7.12 (t, *J* = 7.4 Hz, 1H), 4.33 (s, 2H), 2.74 (t, *J* = 18.3 Hz, 2H), 1.55 (s, 6H). **^{13}C NMR** (101 MHz, $CDCl_3$) δ 161.47, 135.83, 133.44, 133.36, 131.33, 129.43, 128.73, 128.38, 128.17, 127.87, 126.88, 126.62, 126.06, 123.98, 120.59, 117.86, 61.90, 53.09, 37.96 (t, $^2J_{C-F}$ = 22.8 Hz), 21.54. **^{19}F NMR** (376 MHz, $CDCl_3$) δ -101.37 (s, 2F). **HRMS** (ESI) calcd for $C_{23}H_{24}F_2NO_3S$ [M + H]$^+$: 432.1439, found: 432.1443.

4-((Cyanomethyl)sulfonyl)-2,2-difluoro-4-methyl-*N*-phenylpentanamide (77)

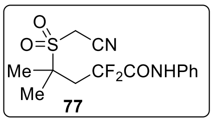

The title compound was prepared according to the ***General procedure I*** and purified by column chromatography (EA:PE = 0-20%) as a yellow oil (78.0 mg, 59% yield). **^{1}H NMR** (500 MHz, $CDCl_3$) δ 8.19 (s, 1H), 7.55 (d, *J* = 7.7 Hz, 2H), 7.37 (t, *J* = 8.0 Hz, 2H), 7.21 (t, *J* = 7.0 Hz, 1H), 4.08 (s, 2H), 2.82 (t, *J* = 18.0 Hz, 2H), 1.67 (s, 6H). **^{13}C NMR** (126 MHz, $CDCl_3$) δ 161.14 (t, $^2J_{C-F}$ = 27.9 Hz), 135.66, 129.42, 126.19, 120.67, 117.35 (t, $^1J_{C-F}$ = 257.8 Hz), 110.70 (d, J_{C-F} = 2.8 Hz), 64.97, 37.66, 37.51 (t, $^2J_{C-F}$ = 22.7 Hz), 21.48 (t, $^3J_{C-F}$ = 2.4 Hz). **^{19}F NMR** (471 MHz, $CDCl_3$) δ -101.51 (s, 2F). **HRMS** (ESI) calcd for $C_{14}H_{16}F_2N_2O_3SNa$ [M + Na]$^+$: 353.0742, found: 353.0744.

Ethyl 2-((4,4-difluoro-2-methyl-5-oxo-5-(phenylamino)pentan-2-yl)sulfonyl)acetate (78)

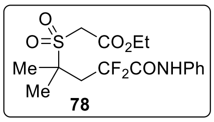

The title compound was prepared according to the ***General procedure I*** and purified by column chromatography (EA:PE = 0-20%) as a light yellow oil (110.2 mg, 73% yield). **^{1}H NMR** (500 MHz, $CDCl_3$) δ 8.27 (s, 1H), 7.56 (d, *J* = 7.7 Hz, 2H), 7.35 (t, *J* = 8.0 Hz, 2H), 7.19 (t, *J* = 7.4 Hz, 1H), 4.25 (q, *J* = 7.2 Hz, 2H), 4.01 (s, 2H), 2.79 (t, *J* = 18.4 Hz, 2H), 1.60 (s, 6H), 1.29 (t, *J* = 7.1 Hz, 3H). **^{13}C NMR** (126 MHz, $CDCl_3$) δ 162.35, 161.31 (t, $^2J_{C-F}$ = 28.1 Hz), 135.86, 129.30, 125.94, 120.54, 117.62 (t, $^1J_{C-F}$ = 257.4 Hz), 63.48, 62.98, 52.97, 37.30 (t, $^2J_{C-F}$ = 22.4 Hz), 20.92 (t, $^3J_{C-F}$ = 2.4 Hz), 13.99. **^{19}F NMR** (471 MHz, $CDCl_3$) δ -101.77 (s, 2F). **HRMS** (ESI) calcd for $C_{16}H_{22}F_2NO_5S$ [M + H]$^+$: 378.1181, found: 378.1190.

4-((2-(Dimethylamino)-2-oxoethyl)sulfonyl)-2,2-difluoro-4-methyl-*N*-phenylpentanamide (79)

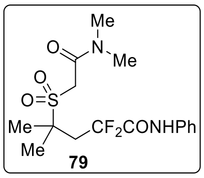

The title compound was prepared according to the ***General procedure I*** and purified by column chromatography (EA:PE = 0-20%) as a yellow oil (109.9 mg, 73% yield). **^{1}H NMR** (400 MHz, $CDCl_3$) δ 8.58 (s, 1H), 7.58 (d, *J* = 8.4 Hz, 2H), 7.32 (t, *J* = 7.9 Hz, 2H), 7.16 (t, *J* = 7.4 Hz, 1H), 4.14 (s, 2H), 3.14 (s, 3H), 2.96 (s, 3H), 2.83 (t, *J* = 18.5 Hz, 2H), 1.60 (s, 6H). **^{13}C NMR** (101 MHz, $CDCl_3$) δ 161.48, 161.48 (t, $^2J_{C-F}$ = 27.7 Hz), 136.14, 129.18, 125.71, 120.57, 117.54 (t, $^1J_{C-F}$ = 256.9 Hz), 63.81, 52.29, 38.99, 37.30 (t, $^2J_{C-F}$ = 22.4 Hz), 36.28, 20.85 (t, $^3J_{C-F}$ = 2.3 Hz). **^{19}F NMR** (376 MHz, $CDCl_3$) δ -101.62 (s, 2F). **HRMS** (ESI) calcd for $C_{16}H_{23}F_2N_2O_4S$ [M + H]$^+$: 377.1341, found: 377.1351.

4-(Cyclopentylsulfonyl)-2,2-difluoro-4-methyl-*N*-phenylpentanamide (80)

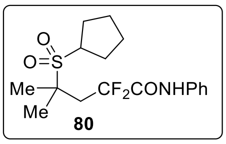

The title compound was prepared according to the ***General procedure I*** and purified by column chromatography (EA:PE = 0-20%) as a light yellow oil (81.9 mg, 57% yield). **^{1}H NMR** (400 MHz, $CDCl_3$) δ 8.05 (s, 1H), 7.56 (d, *J* = 7.7 Hz, 2H), 7.38 (t, *J* = 8.0 Hz, 2H), 7.21 (t, *J* = 7.4 Hz, 1H), 3.59 (p, *J* = 8.4 Hz, 1H), 2.77 (t, *J* = 18.7 Hz, 2H), 2.29 – 1.93 (m, 4H), 1.87 – 1.76 (m, 2H), 1.71 – 1.60 (m, 2H), 1.58 (s, 6H). **^{13}C NMR** (101 MHz, $CDCl_3$) δ 161.54 (t, $^2J_{C-F}$ = 28.0 Hz), 135.85, 129.44, 126.03, 120.48, 118.02 (t, $^1J_{C-F}$ = 258.6 Hz), 61.76, 57.36, 37.87 (t, $^2J_{C-F}$ = 22.3 Hz), 28.68, 25.98, 21.45 (t, $^3J_{C-F}$ = 2.4 Hz). **^{19}F NMR** (376 MHz, $CDCl_3$) δ -101.71 (s, 2F). **HRMS** (ESI) calcd for $C_{17}H_{24}F_2NO_3S$ [M + H]$^+$: 360.1439, found: 360.1445.

2,2-Difluoro-4-(isopropylsulfonyl)-4-methyl-*N*-phenylpentanamide (81)

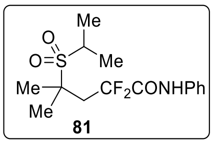

The title compound was prepared according to the ***General procedure I*** and purified by column chromatography (EA:PE = 0-20%) as a light yellow oil (56.2 mg, 42% yield). **^{1}H NMR** (400 MHz, $CDCl_3$) δ 8.19 (s, 1H), 7.56 (d, *J* = 8.3 Hz, 2H), 7.37 (t, *J* = 8.0 Hz, 2H), 7.20 (t, *J* = 7.4 Hz, 1H), 3.48 (p, *J* = 6.9 Hz, 1H), 2.78 (t, *J* = 18.5 Hz, 2H), 1.59 (t, *J* = 1.1 Hz, 6H), 1.42 (d, *J* = 6.9 Hz, 6H). **^{13}C NMR** (101 MHz, $CDCl_3$) δ 161.56 (t, $^2J_{C-F}$ = 28.2 Hz), 135.89, 129.34, 125.97, 120.56, 117.90 (t, $^1J_{C-F}$ = 258.3 Hz), 62.59, 49.82, 37.88 (t, $^2J_{C-F}$ = 22.3 Hz), 21.62 (t, $^3J_{C-F}$ = 2.3 Hz), 17.74. **^{19}F NMR** (376 MHz, $CDCl_3$) δ -101.54 (s, 2F). **HRMS** (ESI) calcd for $C_{15}H_{22}F_2NO_3S$ [M + H]$^+$: 334.1283, found: 334.1286.

2,2-Difluoro-4-methyl-*N*-phenyl-4-((1-phenylethyl)sulfonyl)pentanamide (82)

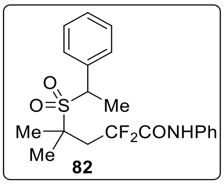

The title compound was prepared according to the ***General procedure I*** and purified by column chromatography (EA:PE = 0-20%) as a light yellow oil (99.5 mg, 63% yield). **^{1}H NMR** (400 MHz, $CDCl_3$) δ 7.91 (s, 1H), 7.57 – 7.47 (m, 4H), 7.43 – 7.30 (m, 5H), 7.21 (t, *J* = 7.4 Hz, 1H), 4.46 (q, *J* = 7.1

Hz, 1H), 2.74 – 2.53 (m, 1H), 2.46 – 2.23 (m, 1H), 1.81 (d, J = 7.1 Hz, 3H), 1.47 (s, 3H), 1.35 (s, 3H). ^{13}**C NMR** (101 MHz, $CDCl_3$) δ 161.34 (t, $^2J_{C-F}$ = 28.2 Hz), 135.85, 135.76, 129.38, 129.25, 129.08, 125.94, 120.46, 117.71 (t, $^1J_{C-F}$ = 258.5 Hz), 63.81, 60.56, 37.90 (t, $^2J_{C-F}$ = 22.2 Hz), 21.72 (t, $^3J_{C-F}$ = 1.6 Hz), 21.64 (t, $^3J_{C-F}$ = 2.4 Hz), 16.73. ^{19}**F NMR** (376 MHz, $CDCl_3$) δ -102.04 (s, 1F), -102.09 (s, 1F). **HRMS** (ESI) calcd for $C_{20}H_{24}F_2NO_3S$ $[M + H]^+$: 396.1439, found: 396.1443.

Ethyl 2-((4,4-difluoro-2-methyl-5-oxo-5-(phenylamino)pentan-2-yl)sulfonyl)propanoate (83)

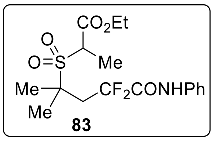

The title compound was prepared according to the ***General procedure I*** and purified by column chromatography (EA:PE = 0-30%) as a yellow oil (144.0 mg, 92% yield). ^{1}**H NMR** (400 MHz, $CDCl_3$) δ 8.28 (s, 1H), 7.56 (d, J = 8.2 Hz, 2H), 7.35 (t, J = 7.9 Hz, 2H), 7.18 (t, J = 7.4 Hz, 1H), 4.34 – 4.10 (m, 3H), 2.01 – 2.66 (m, 2H), 1.68 – 1.49 (m, 9H), 1.28 (t, J = 7.2 Hz, 3H). ^{13}**C NMR** (101 MHz, $CDCl_3$) δ 166.45, 161.38 (t, $^2J_{C-F}$ = 27.8 Hz), 135.88, 129.26, 125.90, 120.58, 117.72 (t, $^1J_{C-F}$ = 257.4 Hz), 64.38, 62.67, 58.09, 37.24 (t, $^2J_{C-F}$ = 22.3 Hz), 22.97 (t, $^3J_{C-F}$ = 2.9 Hz), 13.85, 12.72. ^{19}**F NMR** (376 MHz, $CDCl_3$) δ -101.31 (d, J = 256.7 Hz, 1F), -102.10 (d, J = 256.7 Hz, 1F). **HRMS** (ESI) calcd for $C_{17}H_{24}F_2NO_5S$ $[M + H]^+$: 392.1338, found: 392.1349.

2,2-Difluoro-4-methyl-4-((4-nitrophenyl)sulfonyl)-*N*-phenylpentanamide (84)

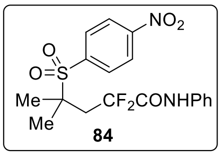

The title compound was prepared according to the ***General procedure I*** and purified by column chromatography (EA:PE = 0-30%) as a yellow oil (71.3 mg, 43% yield). ^{1}**H NMR** (400 MHz, $CDCl_3$) δ 8.07 (s, 1H), 7.54 (d, J = 7.9 Hz, 2H), 7.38 (t, J = 7.9 Hz, 3H), 7.22 (t, J = 7.4 Hz, 3H), 7.13 (s, 1H), 2.79 (t, J = 17.4 Hz, 2H), 1.50 (s, 6H). ^{13}**C NMR** (101 MHz, $CDCl_3$) δ161.99 (t, $^2J_{C-F}$ = 27.6 Hz), 136.83, 135.58, 130.64, 129.66, 129.44, 126.28, 122.34, 121.01, 117.65 (t, $^1J_{C-F}$ = 257.8 Hz), 63.64, 38.98 (t, $^2J_{C-F}$ = 23.3 Hz), 23.33. ^{19}**F NMR** (376 MHz, $CDCl_3$) δ -99.49 (s, 2F). **HRMS** (ESI) calcd for $C_{18}H_{18}F_2N_2NaO_5S$ $[M + Na]^+$: 435.0797, found: 435.0804.

3-(1,1-Dioxidothietan-2-yl)-2,2-difluoro-*N*-phenylpropanamide (85) and 2,2-Difluoro-3-(2-oxido-1,2-oxathiolan-5-yl)-*N*-phenylpropanamide (85')

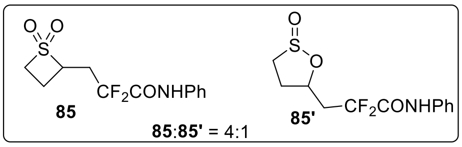

The title compound was prepared according to the ***General procedure II*** and purified by column chromatography (EA:PE = 0-40%) as a white solid (103.0 mg, 89% yield). ^{1}**H NMR** (500 MHz, DMSO-d_6) δ 10.70 (s, 1H), 7.70 (d, J = 7.6 Hz, 2H), 7.37 (t, J = 8.0 Hz, 2H), 7.17 (t, J = 7.4 Hz, 1H), 4.78 – 4.62 (m, 1H), 4.58 – 4.52 (m, 0.18H), 4.37 – 4.29 (m, 0.83H), 3.54 – 3.40 (m, 1H), 2.84 – 2.53 (m, 2H), 2.48 – 2.39 (m, 1H), 2.15 – 2.00 (m,

1H). ^{13}**C NMR** (126 MHz, DMSO-d_6) **85** δ 161.43 (t, $^2J_{C-F}$ = 29.1 Hz), 137.12, 128.86, 125.10, 121.03, 116.90 (t, $^1J_{C-F}$ = 253.2 Hz), 75.10, 60.99, 32.16 (t, $^2J_{C-F}$ = 23.7 Hz), 27.77; **85'** δ 161.41 (t, $^2J_{C-F}$ = 29.1 Hz), 137.08, 128.83, 125.07, 120.99, 116.84 (t, $^1J_{C-F}$ = 253.2 Hz), 75.01, 66.56, 31.25 (t, $^2J_{C-F}$ = 23.9 Hz), 28.97. ^{19}**F NMR** (471 MHz, DMSO-d_6) **85** δ -102.29 (d, *J* = 252.3 Hz), -103.30 (d, *J* = 252.2 Hz); **85'** δ -102.41 (d, *J* = 249.6 Hz), -103.15 (d, *J* = 249.6 Hz). **HRMS** (ESI) calcd for $C_{12}H_{14}F_2NO_3S$ [M + H]$^+$: 290.0657, found: 290.0665.

2,2-Difluoro-3-(2-methyl-1,1-dioxidothietan-2-yl)-*N*-phenylpropanamide (86) and 2,2-Difluoro-3-(5-methyl-2-oxido-1,2-oxathiolan-5-yl)-*N*-phenylpropanamide (86')

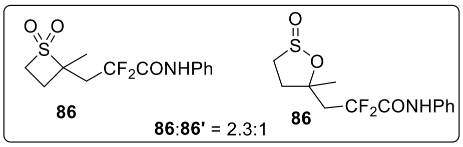

The title compound was prepared according to the ***General procedure II*** and purified by column chromatography (EA:PE = 0-40%) as a white solid (100.9 mg, 83% yield). ^{1}**H NMR** (400 MHz, $CDCl_3$) δ 8.56 (s, 0.3H), 8.51 (s, 0.7H), 7.61 – 7.53 (m, 2H), 7.42 – 7.26 (m, 2H), 7.21 – 7.14 (m, 1H), 4.83 – 4.57 (m, 1H), 4.50 – 4.27 (m, 1H), 2.82 – 2.61 (m, 1.5H), 2.50 – 2.43 (m, 0.5H), 2.41 – 2.29 (m, 1H), 2.29 – 2.20 (m, 0.5H), 2.08 – 2.00 (m, 0.5H), 1.52 (d, *J* = 1.3 Hz, 0.9H), 1.35 (s, 2.1H). ^{13}**C NMR** (126 MHz, $CDCl_3$) **86** δ 161.56 (t, $^2J_{C-F}$ = 28.4 Hz), 136.00, 129.15, 125.73, 120.58, 117.35 (t, $^1J_{C-F}$ = 256.0 Hz), 75.04, 69.56, 37.08 (t, $^2J_{C-F}$ = 22.8 Hz), 34.51, 17.09; **86'** δ 161.47 (t, $^2J_{C-F}$ = 28.4 Hz), 135.92, 129.20, 125.85, 120.60, 117.51 (t, $^1J_{C-F}$ = 257.3 Hz), 75.58, 70.28, 35.86 (t, $^2J_{C-F}$ = 22.8 Hz), 34.39, 17.77 (t, $^3J_{C-F}$ = 2.3 Hz). ^{19}**F NMR** (376 MHz, $CDCl_3$) **86** δ -101.63 (d, *J* = 259.6 Hz), -102.70 (d, *J* = 257.0 Hz); **86'** δ -100.79 (d, *J* = 262.5 Hz), -101.48 (d, *J* = 260.2 Hz). **HRMS** (ESI) calcd for $C_{13}H_{16}F_2NO_3S$ [M + H]$^+$: 304.0813, found: 304.0820.

3-(3,3-Dioxido-1,3-oxathiolan-2-yl)-2,2-difluoro-*N*-phenylpropanamide (87)

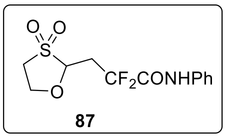

The title compound was prepared according to the ***General procedure II*** and purified by column chromatography (EA:PE = 0-40%) as a light yellow oil (96.4 mg, 79% yield). ^{1}**H NMR** (500 MHz, DMSO-d_6) δ 10.64 (s, 1H), 7.70 – 7.57 (m, 2H), 7.51 – 7.30 (m, 2H), 7.24 – 6.99 (m, 1H), 4.57 (dd, *J* = 7.9, 3.7 Hz, 1H), 4.54 – 4.47 (m, 1H), 4.32 – 4.20 (m, 1H), 3.50 –3.41(m, 1H), 3.34 – 3.24 (m, 1H), 2.94 – 2.73 (m, 1H), 2.72 – 2.55 (m, 1H). ^{13}**C NMR** (126 MHz, DMSO-d_6) δ 160.92 (t, $^2J_{C-F}$ = 28.8 Hz), 137.15, 128.83, 125.03, 120.97, 116.94 (t, $^1J_{C-F}$ = 255.2 Hz), 84.50, 64.81, 47.50, 32.38 (t, $^2J_{C-F}$ = 24.1 Hz). ^{19}**F NMR** (471 MHz, DMSO-d_6) δ -101.11 (d, *J* = 256.5 Hz, 1F), -102.95 (d, *J* = 256.5 Hz, 1F). **HRMS** (ESI) calcd for $C_{12}H_{13}F_2NO_4SNa$ [M + Na]$^+$: 328.0426, found: 328.0431.

3-(1,1-Dioxidotetrahydrothiophen-2-yl)-2,2-difluoro-*N*-phenylpropanamide (88)

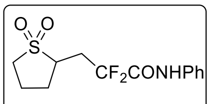

88

The title compound was prepared according to the ***General procedure II*** and purified by column chromatography (EA:PE = 0-30%) as a white solid (73.4 mg, 60% yield). **^{1}H NMR** (400 MHz, $CDCl_3$) δ 8.31 (s, 1H), 7.58 (d, *J* = 8.7 Hz, 2H), 7.36 (t, *J* = 8.0 Hz, 2H), 7.20 (t, *J* = 7.4 Hz, 1H), 3.34 – 3.13 (m, 2H), 3.07 – 2.93 (m, 1H), 2.87 – 2.71 (m, 1H), 2.61 – 2.38 (m, 2H), 2.31 – 2.02 (m, 2H), 1.99 – 1.81 (m, 1H). **^{13}C NMR** (126 MHz, $CDCl_3$) δ 161.19 (t, $^2J_{C\text{-}F}$ = 28.2 Hz), 135.90, 129.30, 125.89, 120.52, 117.63 (d, $^1J_{C\text{-}F}$ = 255.6 Hz), 55.27 (t, $^3J_{C\text{-}F}$ = 2.9 Hz), 50.87, 32.44 (t, $^2J_{C\text{-}F}$ = 24.2 Hz), 29.95, 20.40. **^{19}F NMR** (376 MHz, $CDCl_3$) δ -103.82 (s, 2F). **HRMS** (ESI) calcd for $C_{13}H_{15}F_2NO_3SNa$ [M + Na]$^+$: 326.0633, found: 326.0648.

3-(1,1-Dioxidotetrahydro-2*H*-thiopyran-2-yl)-2,2-difluoro-*N*-phenylpropanamide (89)

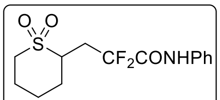

89

The title compound was prepared according to the ***General procedure II*** and purified by column chromatography (EA:PE = 0-30%) as a yellow foam (117.1 mg, 92% yield). **^{1}H NMR** (500 MHz, DMSO-*d_6*) δ 10.67 (s, 1H), 7.67 (d, *J* = 7.7 Hz, 2H), 7.37 (t, *J* = 7.9 Hz, 2H), 7.18 (t, *J* = 7.4 Hz, 1H), 3.46 – 3.37 (m, 1H), 3.32 – 3.21 (m, 1H), 3.20 – 3.13 (m, 1H), 2.85 – 2.64 (m, 1H), 2.41 – 2.24 (m, 1H), 2.22 – 2.13 (m, 1H), 2.05 – 1.94 (m, 1H), 1.89 – 1.65 (m, 3H), 1.62 – 1.44 (m, 1H). **^{13}C NMR** (126 MHz, DMSO-*d_6*) δ 161.31 (t, $^2J_{C\text{-}F}$ = 28.9 Hz), 137.08, 128.84, 125.08, 121.05, 117.03 (t, $^1J_{C\text{-}F}$ = 254.1 Hz), 54.71, 50.51, 30.54, 29.64 (t, $^2J_{C\text{-}F}$ = 24.2 Hz), 24.02, 22.42. **^{19}F NMR** (471 MHz, DMSO-*d_6*) δ -101.80 (d, *J* = 250.6 Hz, 1F), -102.66 (d, *J* = 251.0 Hz, 1F). **HRMS** (ESI) calcd for $C_{14}H_{18}F_2NO_3S$ [M + H]$^+$: 318.0970, found: 318.0988.

3-(1,1-Dioxido-4-tosylthiomorpholin-2-yl)-2,2-difluoro-*N*-phenylpropanamide (90)

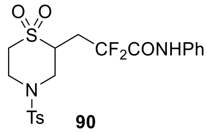

90

The title compound was prepared according to the ***General procedure II*** and purified by column chromatography (EA:PE = 0-30%) as a colorless foam (172.2 mg, 91% yield). **^{1}H NMR** (400 MHz, DMSO-*d_6*) δ 10.74 (s, 1H), 7.69 (td, *J* = 7.5, 6.7, 1.5 Hz, 4H), 7.47 (d, *J* = 8.1 Hz, 2H), 7.43 – 7.31 (m, 2H), 7.24 – 7.07 (m, 1H), 3.89 (d, *J* = 13.3 Hz, 1H), 3.77 (d, *J* = 13.3 Hz, 1H), 3.66 – 3.57 (m, 1H), 3.48 (q, *J* = 4.7 Hz, 2H), 3.25 – 3.01 (m, 2H), 2.85 – 2.62 (m, 1H), 2.60 – 2.45 (m, 1H), 2.41 (s, 3H). **^{13}C NMR** (101 MHz, DMSO-*d_6*) δ 161.00 (t, $^2J_{C\text{-}F}$ = 28.7 Hz), 144.45, 137.07, 133.03, 130.31, 128.87, 127.29, 125.14, 121.05, 116.67 (t, $^1J_{C\text{-}F}$ = 252.5 Hz), 53.60, 49.42, 49.00, 45.08, 27.52 (t, $^2J_{C\text{-}F}$ = 21.2 Hz), 21.11. **^{19}F NMR** (376 MHz, DMSO-*d_6*) δ -102.53 (s, 1F), -102.57 (s, 1F). **HRMS** (ESI) calcd for $C_{20}H_{22}F_2N_2O_5S_2Na$ [M + Na]$^+$: 495.0830, found: 495.0842.

3-(2,2-Dioxidoisothiochroman-3-yl)-2,2-difluoro-*N*-phenylpropanamide (91)

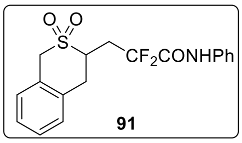

91

The title compound was prepared according to the ***General procedure II*** and purified by column chromatography (EA:PE = 0-30%) as the light yellow oil (77.8 mg, 67% yield). **^{1}H NMR** (400 MHz, DMSO-d_6) δ 10.71 (s, 1H), 7.87 – 7.57 (m, 2H), 7.41 – 7.34 (m, 2H), 7.32 – 7.25 (m, 3H), 7.25 – 7.13 (m, 2H), 4.80 – 4.44 (m, 2H), 3.71 (td, *J* = 9.0, 4.1 Hz, 1H), 3.53 (dd, *J* = 16.4, 4.9 Hz, 1H), 3.27 (dd, *J* = 16.5, 10.1 Hz, 1H), 3.00 – 2.80 (m, 1H), 2.55 – 2.33 (m, 1H). **^{13}C NMR** (101 MHz, DMSO-d_6) δ 161.26 (t, $^{2}J_{C-F}$ = 29.0 Hz), 137.05, 132.85, 129.54, 129.29, 129.00, 128.81, 128.13, 127.18, 125.07, 121.04, 116.84 (t, $^{1}J_{C-F}$ = 254.4 Hz), 52.99, 52.34, 35.08, 30.89 (t, $^{2}J_{C-F}$ = 23.9 Hz). **^{19}F NMR** (376 MHz, DMSO-d_6) δ -101.23 (d, *J* = 252.1 Hz, 1F), -102.90 (d, *J* = 252.2 Hz, 1F). **HRMS** (ESI) calcd for $C_{18}H_{18}F_2NO_3S$ [M + H]$^+$: 366.0970, found: 366.0978.

3-(3,3-Dioxido-2*H*,4*H*-benzo[*e*][1,3]oxathiin-2-yl)-2,2-difluoro-*N*-phenylpropanamide (92)

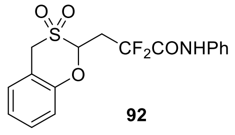

92

The title compound was prepared according to the ***General procedure II*** and purified by column chromatography (EA:PE = 0-30%) as the light yellow oil (86.9 mg, 59% yield). **^{1}H NMR** (400 MHz, $CDCl_3$) δ 8.07 (s, 1H), 7.75 – 7.60 (m, 2H), 7.51 – 7.33 (m, 2H), 7.30 – 7.20 (m, 2H), 7.18 – 7.07 (m, 2H), 6.95 (dd, *J* = 8.2, 1.2 Hz, 1H), 5.14 (dd, *J* = 8.6, 3.6 Hz, 1H), 4.68 – 4.08 (m, 2H), 3.19 – 2.75 (m, 2H). **^{13}C NMR** (101 MHz, $CDCl_3$) δ 160.78 (t, $^{2}J_{C-F}$ = 27.6 Hz), 152.44, 135.87, 130.40, 129.49, 126.05, 125.14, 120.38, 119.51, 117.48, 115.86, 85.63, 53.08, 31.60 (t, $^{2}J_{C-F}$ = 25.2 Hz). **^{19}F NMR** (376 MHz, $CDCl_3$) δ -101.59 (d, *J* = 259.6 Hz, 1F), -104.66 (d, *J* = 259.7 Hz, 1F). **HRMS** (ESI) calcd for $C_{17}H_{16}F_2NO_4S$ [M + H]$^+$: 368.0763, found: 368.0766.

3-(1,1-Dioxidothiepan-2-yl)-2,2-difluoro-*N*-phenylpropanamide (93)

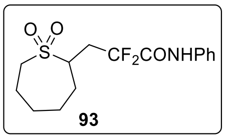

93

The title compound was prepared according to the ***General procedure II*** and purified by column chromatography (EA:PE = 0-30%) as a light yellow oil (116.9 mg, 88% yield). **^{1}H NMR** (500 MHz, DMSO-d_6) δ 10.67 (s, 1H), 7.84 – 7.56 (m, 2H), 7.47 – 7.27 (m, 2H), 7.27 – 7.01 (m, 1H), 3.50 – 3.38 (m, 2H), 3.33 – 3.25 (m, 1H), 2.91 – 2.72 (m, 1H), 2.44 – 2.28 (m, 1H), 2.15 – 1.99 (m, 1H), 1.88 – 1.51 (m, 7H). **^{13}C NMR** (126 MHz, DMSO-d_6) δ 161.42 (t, $^{2}J_{C-F}$ = 28.8 Hz), 137.06, 128.85, 125.09, 121.05, 117.02 (t, $^{1}J_{C-F}$ = 253.8 Hz), 58.26, 52.80, 31.76 (t, $^{2}J_{C-F}$ = 24.0 Hz), 28.43, 25.89, 23.88, 20.24. **^{19}F NMR** (471 MHz, DMSO-d_6) δ -102.35 (d, *J* = 249.6 Hz, 1F), -102.92 (d, *J* = 249.6 Hz, 1F). **HRMS** (ESI) calcd for $C_{15}H_{19}F_2NO_3SNa$ [M + Na]$^+$: 354.0946, found: 354.0959.

3-(3,3-Dioxido-1,3-oxathiepan-2-yl)-2,2-difluoro-*N*-phenylpropanamide (94)

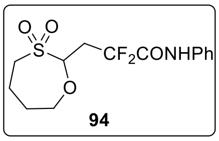

The title compound was prepared according to the ***General procedure II*** and purified by column chromatography (EA:PE = 0-30%) as a white foam (80.1 mg, 60% yield). **^{1}H NMR** (400 MHz, $CDCl_3$) δ 8.01 (s, 1H), 7.57 (d, *J* = 7.6 Hz, 2H), 7.38 (t, *J* = 7.9 Hz, 2H), 7.21 (t, *J* = 7.4 Hz, 1H), 4.89 – 4.78 (m, 1H), 4.34 – 4.19 (m, 1H), 3.77 – 3.66 (m, 1H), 3.38 – 2.28 (m, 1H), 3.25 – 3.17 (m, 1H), 2.85 – 2.70 (m, 2H), 2.23 – 1.95 (m, 2H), 1.93 – 1.75 (m, 2H). **^{13}C NMR** (101 MHz, $CDCl_3$) δ 161.18 (t, $^2J_{C\text{-}F}$ = 28.3 Hz), 135.95, 129.44, 125.92, 120.38, 116.15(t, $^1J_{C\text{-}F}$ = 256.5 Hz), 92.65, 74.45, 54.08, 33.99 (t, $^2J_{C\text{-}F}$ = 25.3 Hz), 29.95, 20.29. **^{19}F NMR** (376 MHz, $CDCl_3$) δ -101.20 (d, *J* = 257.8 Hz, 1F), -105.31 (d, *J* = 257.8 Hz, 1F). **HRMS** (ESI) calcd for $C_{14}H_{17}F_2NO_4SNa$ $[M + Na]^+$: 356.0739, found: 356.0744.

2,2-Difluoro-3-(4-(4-methoxybenzyl)-1,1-dioxido-1,4-thiazepan-2-yl)-*N*-phenylpropanamide (95)

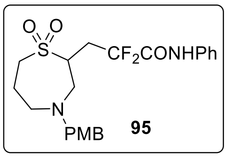

The title compound was prepared according to the ***General procedure II*** and purified by column chromatography (EA:PE = 0-40%) as a yellow oil (107 mg, 59% yield). **^{1}H NMR** (400 MHz, $CDCl_3$) δ 8.13 (s, 1H), 7.56 (d, *J* = 8.1 Hz, 2H), 7.36 (t, *J* = 7.9 Hz, 2H), 7.24 – 7.07 (m, 3H), 6.90 – 6.66 (m, 2H), 3.76 (s, 3H), 3.80 – 3.60 (m, 2H), 3.41 – 3.25 (m, 2H), 3.19 – 3.05 (m, 2H), 2.97 – 2.84 (m, 1H), 2.83 – 2.70 (m, 3H), 2.64 – 2.43 (m, 1H), 2.15 – 2.08 (m, 2H). **^{13}C NMR** (101 MHz, $CDCl_3$) δ 161.10 (t, $^2J_{C\text{-}F}$ = 28.5 Hz), 159.02, 135.91 (d, *J* = 1.9 Hz), 130.36, 130.21, 129.27, 125.82, 120.51 (d, *J* = 1.8 Hz), 116.75 (t, $^1J_{C\text{-}F}$ = 255.9 Hz), 113.94, 62.33, 60.98, 55.35, 52.27, 52.04, 51.71, 29.44 (t, $^2J_{C\text{-}F}$ = 24.0 Hz), 21.40. **^{19}F NMR** (376 MHz, $CDCl_3$) δ -103.16 (d, *J* = 254.5 Hz, 1F), -104.13 – -105.80 (m, 1F). **HRMS** (ESI) calcd for $C_{22}H_{27}F_2N_2O_4S$ $[M + H]^+$: 453.1654, found: 453.1657.

2,2-Difluoro-3-(4-(3-methoxybenzyl)-1,1-dioxido-1,4-thiazocan-8-yl)-*N*-phenylpropanamide (96)

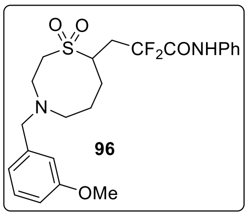

The title compound was prepared according to the ***General procedure II*** and purified by column chromatography (EA:PE = 0-30%) as a yellow oil (128.9 mg, 69% yield). **^{1}H NMR** (400 MHz, $CDCl_3$) δ 8.18 – 8.07 (m, 1H), 7.62 – 7.50 (m, 2H), 7.41 – 7.29 (m, 2H), 7.29 – 7.23 (m, 1H), 7.23 – 7.15 (m, 1H), 6.89 – 6.85 (m, 1H), 6.83 – 6.78 (m, 2H), 3.80 (s, 3H), 3.57 (q, *J* = 12.9 Hz, 2H), 3.18 – 2.90 (m, 2H), 2.83 – 2.72 (m, 1H), 2.64 – 2.28 (m, 5H), 2.08 – 1.86 (m, 3H), 1.84 – 1.56 (m, 2H). **^{13}C NMR** (101 MHz, $CDCl_3$) δ 161.37 (t, $^2J_{C\text{-}F}$ = 28.2 Hz), 159.85, 140.41, 135.91, 129.70, 129.35, 125.89, 121.70, 120.52, 117.27 (t, $^1J_{C\text{-}F}$ = 255.5 Hz), 115.05, 112.77, 61.51, 55.33, 53.56, 53.24, 51.44, 33.32 (t, $^2J_{C\text{-}F}$ = 23.5 Hz), 28.48, 24.85, 21.74. **^{19}F NMR** (376 MHz,

$CDCl_3$) δ -102.48 (d, J = 253.3 Hz, 1F), -104.23 (d, J = 253.0 Hz, 1F). **HRMS** (ESI) calcd for $C_{23}H_{28}F_2N_2O_4SNa$ [M + Na]$^+$: 489.1630, found: 489.1626.

3-(1,1-Dioxido-8-tosyl-1-thia-8-azacyclotetradecan-2-yl)-2,2-difluoro-*N*-phenylpropanamide (97)

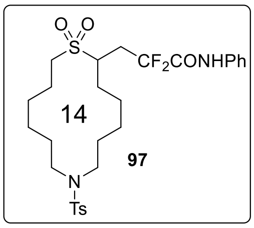

The title compound was prepared according to the ***General procedure II*** and purified by column chromatography (EA:PE = 0-30%) as a yellow oil (70.1 mg, 30% yield). **^{1}H NMR** (400 MHz, $CDCl_3$) δ 8.16 (s, 1H), 7.66 (d, J = 8.2 Hz, 2H), 7.59 – 7.52 (m, 2H), 7.36 (t, J = 7.8 Hz, 2H), 7.31 (d, J = 8.0 Hz, 2H), 7.19 (t, J = 7.4 Hz, 1H), 3.46 – 3.36 (m, 1H), 3.29 – 2.75 (m, 7H), 2.56 – 2.30 (m, 4H), 2.03 – 1.87 (m, 2H), 1.84 – 1.38 (m, 14H). **^{13}C NMR** (101 MHz, $CDCl_3$) δ 161.34 (t, $^2J_{C-F}$ = 28.6 Hz), 143.46, 135.92, 135.61, 129.84, 129.34, 127.37, 125.91, 120.60, 116.38, 52.96, 50.00, 49.50, 48.85, 31.73 (t, $^2J_{C-F}$ = 24.9 Hz), 28.93, 28.05, 26.53, 25.53, 24.11, 23.87, 23.73, 21.63, 20.88. **^{19}F NMR** (376 MHz, $CDCl_3$) δ -102.06 (d, J = 252.4, 1F), -104.29 (d, J = 252.0 Hz, 1F). **HRMS** (ESI) calcd for $C_{28}H_{39}F_2N_2O_5S_2$ [M + H]$^+$: 585.2263, found: 585.2267.

6. Synthetic Transformation of Products

6.1 Gram-scale synthesis of 9

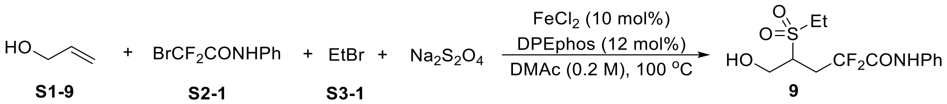

In a N_2 atmosphere-controlled glove box, $FeCl_2$ (50.7 mg, 10 mol%), DPEphos (258.5 mg, 12 mol%), allyl alcohol (**S1-9**, 232.3 mg, 4.0 mmol, 1.0 equiv.), 2-bromo-2,2-difluoro-*N*-phenylacetamide (**S2-1**, 1.20 g, 4.8 mmol, 1.2 equiv.), bromoethane (**S3-1**, 653.8 mg, 6.0 mmol, 1.5 equiv.), $Na_2S_2O_4$ (1.04 g, 6.0 mmol, 1.5 equiv.) and DMAc (20.0 mL) were added to a 50 mL flame-dried Young-type tube. The reaction mixture was stirred at 100 °C. After stirring for 12 hours and monitored by TLC, the reaction was cooled to room temperature. The reaction mixture was diluted with H_2O. Then aqueous phase was extracted with EtOAc (3 × 40 mL). The combined organic extracts were washed with brine, dried over Na_2SO_4, filtered, and concentrated in vacuo. The residue was purified by silica gel column chromatography (EA:PE = 0-30%) to give the desired product **9** (1.21 g, 94% yield).

6.2 Post-functionalization of product 9

5-((*tert*-Butyldimethylsilyl)oxy)-4-(ethylsulfonyl)-2,2-difluoro-*N*-phenylpentanamide (98)

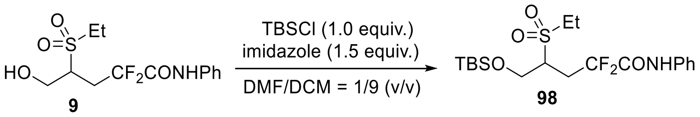

Product **9** (64.2 mg, 0.20 mmol, 1.0 equiv.) in a mixed solvent of DMF/DCM (0.9 mL/0.1 mL) at 0 °C. Then imidazole (20.4 mg, 0.30 mmol, 1.5 equiv.) and TBSCl (30.1 mg, 0.20 mmol, 1.0 equiv.) were added, and the reaction mixture was allowed to stir at room temperature for 16 hours. After completion, the mixture was extracted with DCM. The organic layers were combined and evaporated under reduced pressure. The residue was purified by silica gel column chromatography (EA:PE = 0-10%) to give the product **98** as a colorless oil (77.6 mg, 89% yield). **^{1}H NMR** (400 MHz, $CDCl_3$) δ 8.24 (s, 1H), 7.57 (d, *J* = 7.6 Hz, 2H), 7.35 (t, *J* = 8.0 Hz, 2H), 7.19 (t, *J* = 7.4 Hz, 1H), 4.20 (dd, *J* = 11.4, 2.7 Hz, 1H), 4.01 (dd, *J* = 11.5, 6.8 Hz, 1H), 3.47 – 3.38 (m, 1H), 3.28 – 3.02 (m, 2H), 2.95 – 2.75 (m, 1H), 2.70 – 2.43 (m, 1H), 1.38 (t, *J* = 7.5 Hz, 3H), 0.89 (s, 9H), 0.09 (s, 3H), 0.09 (s, 3H). **^{13}C NMR** (101 MHz, $CDCl_3$) δ 161.08 (t, $^2J_{C\text{-}F}$ = 28.2 Hz), 135.87, 129.28, 125.89, 120.54, 116.68 (t, $^1J_{C\text{-}F}$ = 256.5 Hz), 61.25, 58.79, 48.15, 28.41 (t, $^2J_{C\text{-}F}$ = 24.1 Hz), 25.85, 18.28, 5.94, -5.60, -5.66. **^{19}F NMR** (376 MHz, $CDCl_3$) δ -103.12 (d, *J* = 254.1 Hz, 1F), -105.79 (d, *J* = 254.1 Hz, 1F). **HRMS** (ESI) calcd for $C_{19}H_{31}F_2NNaO_4SSi$ $[M + Na]^+$: 458.1603, found: 458.1606.

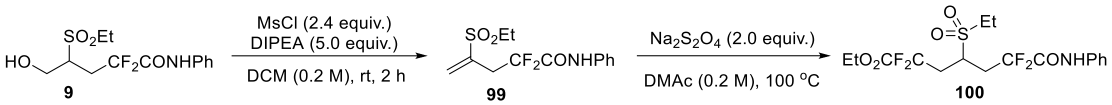

4-(Ethylsulfonyl)-2,2-difluoro-*N*-phenylpent-4-enamide (99)

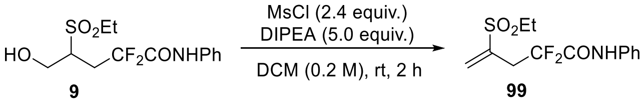

Product **9** (128.5 mg, 0.40 mmol, 1.0 equiv.) was dissolved in DCM (2.0 mL), and the mixture was cooled to 0 °C. DIPEA (258.4 mg, 2.0 mmol, 5.0 equiv.) was added to this solution and methylsulfonyl chloride (110.0 mg, 0.96 mmol, 2.4 equiv.) sequentially. After two hours the reaction was monitored by TLC for completion. Once completed the reaction was quenched with saturated aqueous NH_4Cl. The aqueous layer was extracted with DCM and the combined organic layers were washed with brine, dried over Na_2SO_4, filtered, and concentrated under reduced pressure. The residue was purified by flash chromatography (EA:PE = 0-20%) to give the desired product **99** as light yellow oil (117.8 mg, 97% yield). **^{1}H NMR** (400 MHz, $CDCl_3$) δ 8.36 (s, 1H), 7.70 – 7.47 (m, 2H), 7.35 (t, *J* = 7.9 Hz, 2H), 7.19 (td, *J* = 7.3, 1.2 Hz, 1H), 6.49 (s, 1H), 6.23 (d, *J* = 1.5 Hz, 1H), 3.47 – 3.28 (m, 2H), 3.06 (q, *J* = 7.4 Hz, 2H), 1.28 (t, *J* = 7.4 Hz, 3H). **^{13}C NMR** (101 MHz, $CDCl_3$) δ 160.81 (t, $^2J_{C\text{-}F}$ = 28.1 Hz), 139.43, 135.76, 132.41, 129.31, 126.00, 120.55, 116.11 (t, $^2J_{C\text{-}F}$ = 257.8 Hz), 47.12, 33.67 (t, $^2J_{C\text{-}F}$ = 24.9 Hz), 6.93. **^{19}F NMR** (376 MHz, $CDCl_3$) δ -103.35 (s, 2F). **HRMS** (ESI) calcd for $C_{13}H_{15}F_2NNaO_3S$ $[M + Na]^+$: 326.0633, found: 326.0635.

Ethyl 4-(ethylsulfonyl)-2,2,6,6-tetrafluoro-7-oxo-7-(phenylamino)heptanoate (100)

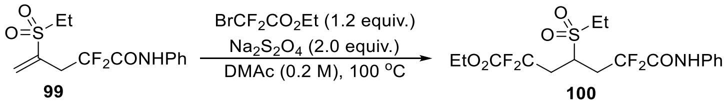

In a N_2 atmosphere-controlled glove box, alkene **99** (64.3 mg, 0.2 mmol, 1.0 equiv.), ethyl bromodifluoroacetate (48.7 mg, 0.24 mmol, 1.2 equiv.), $Na_2S_2O_4$ (69.6 mg, 0.40 mmol, 2.0 equiv.) and DMAc (1.0 mL) were added to a 25 mL flame-dried Young-type tube. The reaction mixture was stirred at 100 °C. After stirring for 12 hours and monitored by TLC, the reaction was cooled to room temperature. The reaction mixture was diluted with H_2O. Then aqueous phase was extracted with EtOAc (3 × 5 mL). The combined organic extracts were washed with brine, dried over Na_2SO_4, filtered, and concentrated in vacuo. The residue was purified by flash chromatography (EA:PE = 0-20%) to give the desired product **100** as yellow oil (48.8 mg, 57% yield). **^{1}H NMR** (400 MHz, $CDCl_3$) δ 7.99 (s, 1H), 7.53 – 7.40 (m, 2H), 7.28 (t, *J* = 8.0 Hz, 2H), 7.15 – 7.06 (m, 1H), 4.23 (q, *J* = 7.1 Hz, 2H), 3.65 – 3.58 (m, 1H), 3.04 (q, *J* = 7.5 Hz, 2H), 3.01 – 2.81 (m, 2H), 2.58 – 2.34 (m, 2H), 1.32 (t, *J* = 7.4 Hz, 3H), 1.25 (t, *J* = 7.2 Hz, 3H). **^{13}C NMR** (101 MHz, $CDCl_3$) δ 163.11 (t, $^2J_{C\text{-}F}$ = 31.9 Hz), 160.84 (t, $^2J_{C\text{-}F}$ = 27.9 Hz), 135.67, 129.40, 126.12, 120.66, 116.51 (t, $^1J_{C\text{-}F}$ = 257.3

Hz), 114.49 (t, $^{1}J_{C\text{-}F}$ = 253.6 Hz), 63.75, 49.37, 45.73, 33.69 (t, $^{2}J_{C\text{-}F}$ = 24.6 Hz), 33.64 (t, $^{2}J_{C\text{-}F}$ = 24.6 Hz), 13.96, 6.29. **^{19}F NMR** (376 MHz, $CDCl_3$) δ -100.96 (dd, *J* = 7.5, 4.5 Hz), -101.66 (dd, *J* = 7.6, 4.3 Hz), -102.20 (dd, *J* = 7.5, 5.3 Hz), -102.91 (dd, *J* = 7.3, 5.0 Hz), -104.00 (t, *J* = 4.4 Hz), -104.69 (t, *J* = 4.4 Hz), -104.98 (t, *J* = 3.8 Hz), -105.69 (t, *J* = 4.0 Hz). **HRMS** (ESI) calcd for $C_{17}H_{21}F_4NNaO_5S$ [M + Na]$^+$: 450.0969, found: 450.0977.

2-(Ethylsulfonyl)-4,4-difluoro-5-(phenylamino)pentan-1-ol (101)

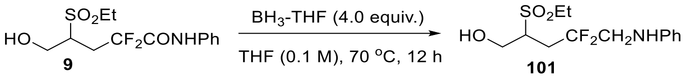

To a 25 mL flame-dried Young-type tube were added product **9** (321.3 mg, 1.0 mmol, 1.0 equiv.) and THF (10.0 mL) under N_2. Then BH_3-THF (4.0 mL, 4.0 mmol, 4 equiv.) was added dropwise to the mixture under N_2 atmosphere in room temperature. The reaction mixture was stirred at 70 ^{o}C. After stirring for 12 hours and monitored by TLC the reaction was cooled to room temperature. The reaction mixture was quenched by H_2O. Then aqueous phase was extracted with ethyl acetate (3 × 20 mL). The combined organic extracts were washed with brine, dried over Na_2SO_4, filtered, and concentrated in vacuo. The residue was purified by flash chromatography (EA:PE = 0-20%) to give the desired product **101** as a colorless oil (300.3 mg, 98% yield). **^{1}H NMR** (400 MHz, $CDCl_3$) δ 7.20 (t, *J* = 7.9 Hz, 2H), 6.78 (t, *J* = 7.3 Hz, 1H), 6.68 (d, *J* = 7.8 Hz, 2H), 4.18 (dd, *J* = 12.5, 2.7 Hz, 1H), 4.02 (dd, *J* = 12.5, 6.8 Hz, 1H), 3.57 (t, *J* = 13.2 Hz, 2H), 3.47 – 3.38 (m, 1H), 3.20 – 3.07 (m, 2H), 2.76 – 2.57 (m, 1H), 2.47 – 2.25 (m, 1H), 1.38 (t, *J* = 7.5 Hz, 3H). **^{13}C NMR** (101 MHz, $CDCl_3$) δ 146.99, 129.51, 123.12 (t, $^{1}J_{C\text{-}F}$ = 244.9 Hz), 118.90, 113.37, 60.45 (d, $^{3}J_{C\text{-}F}$ = 3.1 Hz), 57.61 (d, $^{3}J_{C\text{-}F}$ = 2.6 Hz), 49.23 (t, $^{2}J_{C\text{-}F}$ = 29.7 Hz), 47.48, 28.80 (t, $^{2}J_{C\text{-}F}$ = 24.2 Hz), 6.06. **^{19}F NMR** (376 MHz, $CDCl_3$) δ -101.49 (d, *J* = 245.8 Hz, 1F), -104.03 (d, *J* = 245.8 Hz, 1F). **HRMS** (ESI) calcd for $C_{13}H_{19}F_2NNaO_3S$ [M + Na]$^+$: 330.0946, found: 330.0957.

5-(Ethylsulfonyl)-3,3-difluoro-1-phenylpiperidine (102)

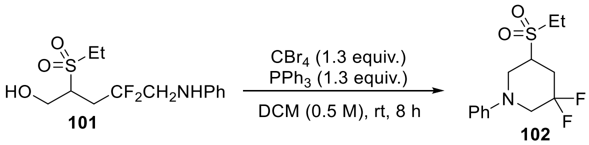

Alcohol **101** (123.0 mg, 0.4 mmol, 1.0 equiv.) and PPh_3 (136.4mg, 0.52 mmol, 1.3 equiv.) were dissolved in the dry DCM (1.0 mL), and the mixture was cooled to 0 ^{o}C. CBr_4 (172.4 mg, 0.52 mmol, 1.3 equiv.) was added to this solution. After 8 hours the reaction was monitored by TLC for completion. Once completion the reaction was quenched with H_2O. The aqueous layer was extracted with DCM and the combined organic layers were washed with brine, dried over Na_2SO_4, filtered, and concentrated under reduced pressure. The crude material was purified by flash chromatography (EA:PE = 0-20%) to give the desired product **102** as colorless oil (66.9 mg, 58% yield). **^{1}H NMR**

(400 MHz, $CDCl_3$) δ 7.34 – 7.27 (m, 2H), 7.03 – 6.87 (m, 3H), 4.14 – 4.04 (m, 1H), 3.95 – 3.82 (m, 1H), 3.58 – 3.45 (m, 1H), 3.23 – 2.94 (m, 4H), 2.71 – 2.57 (m, 1H), 2.38 – 2.17 (m, 1H), 1.45 (t, *J* = 7.5 Hz, 3H). **^{13}C NMR** (101 MHz, $CDCl_3$) δ 149.11, 129.61, 121.83, 119.02 (t, $^{1}J_{C-F}$ = 245.0 Hz), 117.65, 55.45 (dd, $^{2}J_{C-F}$ = 32.1, 25.9 Hz), 54.47 (d, $^{3}J_{C-F}$ = 8.3 Hz), 47.69, 45.86, 32.44 (t, $^{2}J_{C-F}$ = 26.3 Hz), 6.19. **^{19}F NMR** (376 MHz, $CDCl_3$) δ -99.38 (d, *J* = 243.8 Hz, 1F), -100.40 (d, *J* = 243.6 Hz, 1F). **HRMS** (ESI) calcd for $C_{13}H_{18}F_2NO_2S$ $[M + H]^+$: 290.1021, found: 290.1033.

N-(4-(Ethylsulfonyl)-2,2-difluoropent-4-en-1-yl)aniline (103)

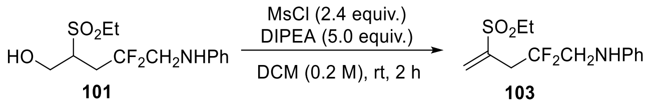

Alcohol **101** (61.5 mg, 0.2 mmol, 1.0 equiv.) was dissolved in DCM (1.0 mL), and the mixture was cooled to 0 °C. DIPEA (129.2 mg, 1.0 mmol, 5.0 equiv.) was added to this solution and methylsulfonyl chloride (55.0 mg, 0.48 mmol, 2.4 equiv.) sequentially. After two hours the reaction was monitored by TLC for completion. Once completion the reaction was quenched with saturated aqueous NH_4Cl. The aqueous layer was extracted with DCM and the combined organic layers were washed with brine, dried over Na_2SO_4, filtered, and concentrated under reduced pressure. The residue was purified by flash chromatography (EA:PE = 0-20%) to give the desired product **103** as yellow oil (53.6 mg, 93% yield). **^{1}H NMR** (400 MHz, $CDCl_3$) δ 7.20 (dd, *J* = 8.6, 7.4 Hz, 2H), 6.77 (tt, *J* = 7.3, 1.1 Hz, 1H), 6.71 – 6.65 (m, 2H), 6.52 (s, 1H), 6.25 (s, 1H), 4.18 (d, *J* = 6.9 Hz, 1H), 3.58 (td, *J* = 12.9, 5.9 Hz, 2H), 3.13 (td, *J* = 16.5, 1.1 Hz, 2H), 3.02 (q, *J* = 7.4 Hz, 2H), 1.31 (t, *J* = 7.4 Hz, 3H). **^{13}C NMR** (101 MHz, $CDCl_3$) δ 147.02, 140.14 (t, $^{4}J_{C-F}$ = 3.3 Hz), 132.59, 129.45, 122.41 (t, $^{1}J_{C-F}$ = 246.2 Hz), 118.70, 113.26, 48.14 (t, $^{2}J_{C-F}$ = 29.8 Hz), 47.13 (d, $^{4}J_{C-F}$ = 1.8 Hz), 34.01 (t, $^{2}J_{C-F}$ = 25.8 Hz), 6.98. **^{19}F NMR** (376 MHz, $CDCl_3$) δ -101.37 (s, 2F). **HRMS** (ESI) calcd for $C_{13}H_{18}F_2NO_2S$ $[M + H]^+$: 290.1021, found: 290.1029.

6.3 Gram-scale synthesis of 104

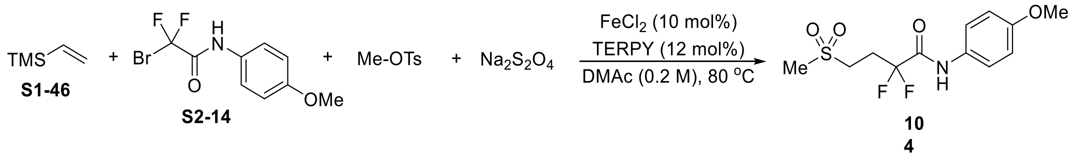

In a N_2 atmosphere-controlled glove box, $FeCl_2$ (50.7 mg, 10 mol%), TERPY (112.0 mg, 12 mol%), trimethyl(vinyl)silane (**S1-46**, 401.0 mg, 4.0 mmol, 1.0 equiv.), 2-bromo-2,2-difluoro-*N*-(4-methoxyphenyl)acetamide (**S2-14**, 1.34 g, 4.8 mmol, 1.2 equiv.), methyl 4-methylbenzenesulfonate (1.12 g, 6.0 mmol, 1.5 equiv.), $Na_2S_2O_4$ (1.04 g, 6.0 mmol, 1.5 equiv.) and DMAc (20.0 mL) were added to a 50 mL flame-dried Young-type tube. The reaction mixture was stirred at 80 °C. After stirring for 12 hours and monitored by TLC, the reaction was cooled to room temperature. The

reaction mixture was diluted with H_2O. Then aqueous phase was extracted with EtOAc (3 × 40 mL). The combined organic extracts were washed with brine, dried over Na_2SO_4, filtered, and concentrated in vacuo. The residue was purified by silica gel column chromatography (EA:PE = 0-50%) to give the desired product **104** (0.75 g, 61% yield). **^{1}H NMR** (400 MHz, DMSO-d_6) δ 10.55 (s, 1H), 7.58 (d, J = 9.0 Hz, 2H), 6.94 (d, J = 9.0 Hz, 2H), 3.74 (s, 3H), 3.41 – 3.29 (m, 2H), 3.09 (s, 3H), 2.70 – 2.54 (m, 2H). **^{13}C NMR** (101 MHz, DMSO-d_6) δ 160.80, 156.44, 130.01, 122.58, 116.69, 113.92, 55.26, 46.52, 40.20, 27.10 (t, $^2J_{C\text{-}F}$ = 24.8 Hz). **^{19}F NMR** (376 MHz, DMSO-d_6) δ -104.10 (s, 2F). **HRMS** (ESI) calcd for $C_{12}H_{15}F_2NNaO_4S$ [M + Na]$^+$: 330.0582, found: 330.0588.

6.4 Synthesis the intermediates of Erysolin and its derivatives

N-(2,2-Difluoro-4-(methylsulfonyl)butyl)-4-methoxyaniline (105)

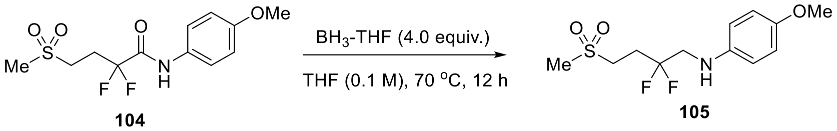

104 105

To a 25 mL flame-dried Young-type tube were added product **104** (153.7 mg, 0.5 mmol, 1.0 equiv.) and THF (5.0 mL) under N_2. Then BH_3-THF (2.0 mL, 2.0 mmol, 4 equiv.) was added dropwise to the mixture under N_2 atmosphere in room temperature. The reaction mixture was stirred at 70 °C. After stirring for 12 hours and monitored by TLC the reaction was cooled to room temperature. The reaction mixture was quenched by H_2O. Then aqueous phase was extracted with ethyl acetate (3 × 10 mL). The combined organic extracts were washed with brine, dried over Na_2SO_4, filtered, and concentrated in vacuo. The residue was purified by flash chromatography (EA:PE = 0-50%) to give the desired product **105** as a white solid (139.0 mg, 95% yield). **^{1}H NMR** (400 MHz, $CDCl_3$) δ 6.80 (d, J = 8.8 Hz, 2H), 6.64 (d, J = 8.8 Hz, 2H), 3.75 (s, 3H), 3.64 (d, J = 6.8 Hz, 1H), 3.52 (td, J = 12.8, 5.6 Hz, 2H), 3.31 – 3.20 (m, 2H), 2.94 (s, 3H), 2.61 – 2.41 (m, 2H). **^{13}C NMR** (126 MHz, $CDCl_3$) δ 153.21, 140.93, 122.60 (t, $^1J_{C\text{-}F}$ = 244.2 Hz), 115.11, 114.88, 55.87, 50.14 (t, $^2J_{C\text{-}F}$ = 29.5 Hz), 47.93 (t, $^3J_{C\text{-}F}$ = 4.1 Hz), 41.02, 27.72 (t, $^2J_{C\text{-}F}$ = 25.1 Hz). **^{19}F NMR** (376 MHz, $CDCl_3$) δ -103.91 (s, 2F). **HRMS** (ESI) calcd for $C_{12}H_{18}F_2NO_3S$ [M + H]$^+$: 294.0970, found: 294.0979.

2-Fluoro-*N*-(4-methoxyphenyl)-4-(methylsulfonyl)butanamide (106) and *N*-(4-methoxyphenyl)-4-(methylsulfonyl)butanamide (107)

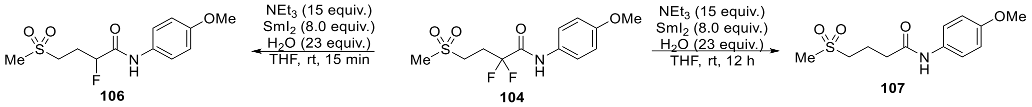

106 104 107

The product **104** (61.5 mg, 0.2 mmol, 1.0 equiv.) was solvated in dry THF (14.0 mL) and dry triethylamine (0.447 mL, 3.0 mmol, 15 equiv.) was added. A mixture of SmI_2 (16.0 mL, 1.6 mmol, 8.0 equiv.) and H_2O (82.8 mg, 4.6 mmol, 23 equiv.) was added dropwise to the amide solution at

room temperature. The reaction was stirred for 15 min and quenched by removing the lid and let air into the vessel, followed by adding 1 mL sat. $Na_2S_2O_3$ (aq.). The crude mixture was purified by column chromatography (EA:PE = 0-80%) and afforded the desired product **106** as brown solid in 31% yield. **^{1}H NMR** (500 MHz, $CDCl_3$) δ 7.93 (s, 1H), 7.45 (d, *J* = 9.0 Hz, 2H), 6.89 (d, *J* = 9.0 Hz, 2H), 5.18 (ddd, *J* = 48.9, 7.1, 4.6 Hz, 1H), 3.81 (s, 3H), 3.45 – 3.08 (m, 2H), 2.95 (s, 3H), 2.86 – 2.42 (m, 2H). **^{13}C NMR** (126 MHz, $CDCl_3$) δ 166.08 (d, $^2J_{C\text{-}F}$ = 18.0 Hz), 157.30, 129.32, 122.07, 114.48, 89.57 (d, $^1J_{C\text{-}F}$ = 190.4 Hz), 55.64, 49.95 (d, $^3J_{C\text{-}F}$ = 3.8 Hz), 40.83, 25.85 (d, $^2J_{C\text{-}F}$ = 21.1 Hz). ^{19}F NMR (471 MHz, $CDCl_3$) δ -188.34 (s, 1F). **HRMS** (ESI) calcd for $C_{12}H_{16}FNNaO_4S$ [M + Na]$^+$: 312.0676, found: 312.0683.

If the reaction was stirred for 12 h and quenched by removing the lid and let air into the vessel, followed by adding 1 mL sat. $Na_2S_2O_3$ (aq.). The crude mixture was purified by column chromatography (EA:PE = 0-100%) and afforded the desired product **107** as white solid in 27% yield. **^{1}H NMR** (500 MHz, $CDCl_3$) δ 7.40 (d, *J* = 9.0 Hz, 3H), 6.86 (d, *J* = 8.9 Hz, 2H), 3.79 (s, 3H), 3.17 (t, *J* = 7.2 Hz, 2H), 2.94 (s, 3H), 2.60 (t, *J* = 6.8 Hz, 2H), 2.28 (p, *J* = 7.0 Hz, 2H). **^{13}C NMR** (126 MHz, $CDCl_3$) δ 169.47, 156.71, 130.78, 121.94, 114.33, 55.63, 53.42, 41.05, 34.78, 18.67. **HRMS** (ESI) calcd for $C_{12}H_{17}NNaO_4S$ [M + Na]$^+$: 294.0770, found: 294.0777.

7. Mechanistic Experiments

7.1 Radical clock experiments

7-(Ethylsulfonyl)-2,2-difluoro-*N*,4-diphenylhept-4-enamide (108)

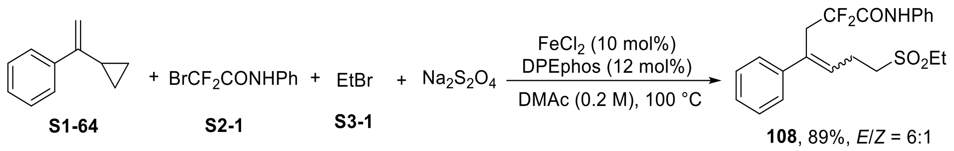

108, 89%, *E*/*Z* = 6:1

The title compound was prepared according to the ***General procedure I*** and purified by column chromatography (EA:PE = 0-10%) as a light yellow oil (145.1 mg, 89% yield). **^{1}H NMR** (400 MHz, $CDCl_3$) δ 8.08 (s, 1H), 7.48 – 7.38 (m, 2H), 7.34 – 7.19 (m, 7H), 7.16 – 7.10 (m, 1H), 5.87 (t, *J* = 7.5 Hz, 0.86H), 5.73(t, *J* = 7.3 Hz, 0.14H), 3.46 (t, *J* = 16.4 Hz, 1.7H), 3.26 (t, *J* = 16.3 Hz, 0.3H), 3.08 – 3.02 (m, 1.4H), 2.97 (q, *J* = 7.5 Hz, 1.7H), 2.84 (dd, *J* = 8.9, 6.8 Hz, 0.6H), 2.82 – 2.73 (m, 1.7H), 2.48 (q, *J* = 7.5 Hz, 0.3H), 1.93 (dd, *J* = 5.1, 2.6 Hz, 0.3H), 1.36 (t, *J* = 7.5 Hz, 2.57H), 1.22 (t, *J* = 7.5 Hz, 0.43H). **^{13}C NMR** (101 MHz, $CDCl_3$) δ 161.72 (t, $^2J_{C-F}$ = 28.5 Hz), 141.41, 138.49, 135.94, 136.00, 133.35 (t, $^3J_{C-F}$ = 3.7 Hz), 134.42, 130.89, 129.78, 129.06, 129.10, 128.46, 128.56, 127.71, 128.19, 126.66, 127.74, 125.57, 125.55, 120.41, 120.33, 117.06 (t, $^1J_{C-F}$ = 256.2 Hz), 51.07, 51.13, 47.36, 47.13, 34.76 (t, $^2J_{C-F}$ = 24.1 Hz), 42.99 (t, $^2J_{C-F}$ = 23.3 Hz), 22.18, 21.95, 6.52, 6.46. **^{19}F NMR** (376 MHz, $CDCl_3$) δ **Major isomer** -102.68; **Minor isomer** -103.43. **HRMS** (ESI) calcd for $C_{21}H_{23}F_2NNaO_3S$ [M + Na]$^+$: 430.1259, found: 430.1266.

3-(4-((Ethylsulfonyl)methyl)-1-tosylpyrrolidin-3-yl)-2,2-difluoro-*N*-phenylpropanamide (109)

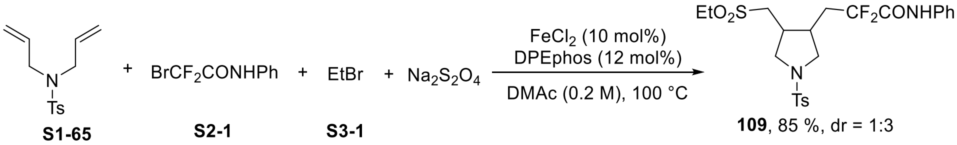

109, 85 %, dr = 1:3

The title compound was prepared according to the ***General procedure I*** and purified by column chromatography (EA:PE = 0-20%) as a white foam (175.0 mg, 85% yield). **^{1}H NMR** (400 MHz, $CDCl_3$) δ 8.48 (s, 1H), 7.74 – 7.63 (m, 2H), 7.61 – 7.53 (m, 2H), 7.41 – 7.23 (m, 4H), 7.19 (t, *J* = 7.4 Hz, 1H), 3.80 – 3.29 (m, 2.9H), 3.26 – 3.06 (m, 1.1H), 3.04 – 2.90 (m, 2H), 2.90 – 2.81 (m, 1H), 2.82 – 2.71 (m, 1H), 2.68 – 2.59 (m, 1H), 2.58 – 2.49 (m, 1H), 2.40 (d, *J* = 3.8 Hz, 3H), 2.35 – 2.03 (m, 2H), 1.40 – 1.22 (m, 3H). **^{13}C NMR** (101 MHz, $CDCl_3$) δ 161.49 (t, $^2J_{C-F}$ = 28.5 Hz), 144.11, 144.16, 136.01, 136.00, 133.14, 132.57, 130.04, 130.01, 129.22, 127.49, 127.62, 125.80, 120.52, 120.56, 117.42 (t, $^1J_{C-F}$ = 256.0 Hz), 117.28 (t, $^1J_{C-F}$ = 256.0 Hz), 53.41, 52.56, 51.57, 51.95, 49.27, 51.27, 48.54, 48.33, 37.31, 37.75, 35.03, 35.45, 31.92 (t, $^2J_{C-F}$ = 23.2 Hz), 35.66, 21.57, 21.60, 6.50, 6.59. **^{19}F NMR** (376 MHz, $CDCl_3$) **Major isomer** δ -105.50 (d, *J* = 256.8 Hz), -105.50 (d, *J* = 256.8 Hz); **Minor isomer** δ -102.22 (d, *J* = 257.9 Hz), -105.59 (d, *J* = 257.9 Hz). **HRMS** (ESI) calcd for

$C_{23}H_{29}F_2N_2O_5S_2$ [M + H]$^+$: 515.1480, found: 515.1492.

7.2 Reaction with stoichiometric amount of [Fe]

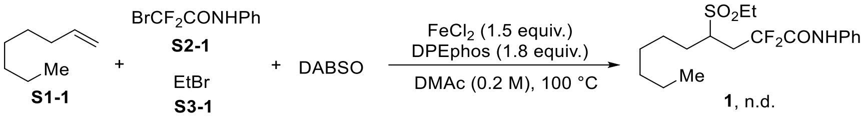

In a N_2 atmosphere-controlled glove box, $FeCl_2$ (38.0 mg, 0.3 mmol, 1.5 equiv.), DPEphos (193.9 mg, 0.36 mmol, 1.8 equiv.), 1-octene (**S1-1**, 22.4 mg, 0.2 mmol, 1.0 equiv.), 2-bromo-2,2-difluoro-*N*-phenylacetamide (**S2-1**, 60.0 mg, 0.24 mmol, 1.2 equiv.), bromoethane (**S3-1**, 32.7 mg, 0.3 mmol, 1.5 equiv.), DABSO (72.1 mg, 0.3 mmol, 1.5 equiv.) and DMAc (1.0 mL) were added to a 25 mL flame-dried Young-type tube. The reaction mixture was stirred at 100 ^{o}C. After stirring for 12 hours and monitored by TLC, the reaction was cooled to room temperature. Yields were determined by GC with octadecane as the internal standard. No desired product was obtained.

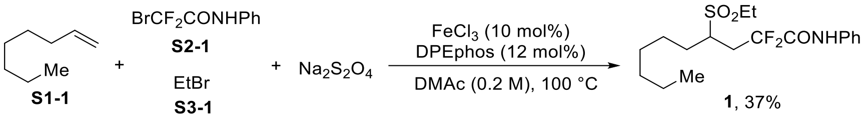

In a N_2 atmosphere-controlled glove box, $FeCl_3$ (3.2 mg, 10 mol%), DPEphos (12.9 mg, 12 mol%), 1-octene (**S1-1**, 22.4 mg, 0.2 mmol, 1.0 equiv.), 2-bromo-2,2-difluoro-*N*-phenylacetamide (**S2-1**, 60.0 mg, 0.24 mmol, 1.2 equiv.), bromoethane (**S3-1**, 32.7 mg, 0.3 mmol, 1.5 equiv.), $Na_2S_2O_4$ (52.2 mg, 0.3 mmol, 1.5 equiv.) and DMAc (1.0 mL) were added to a 25 mL flame-dried Young-type tube. The reaction mixture was stirred at 100 ^{o}C. After stirring for 12 hours and monitored by TLC, the reaction was cooled to room temperature. Yields were determined by GC with octadecane as the internal standard. The desired product **1** was obtained in 37% yield.

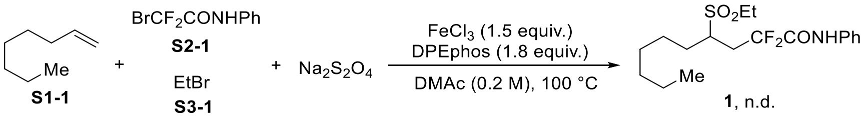

In a N_2 atmosphere-controlled glove box, $FeCl_3$ (48.7 mg, 0.3 mmol, 1.5 equiv.), DPEphos (193.9 mg, 0.36 mmol, 1.8 equiv.), 1-octene (**S1-1**, 22.4 mg, 0.2 mmol, 1.0 equiv.), 2-bromo-2,2-difluoro-*N*-phenylacetamide (**S2-1**, 75.0 mg, 0.3 mmol, 1.5 equiv.), bromoethane (**S3-1**, 32.7 mg, 0.3 mmol, 1.5 equiv.), $Na_2S_2O_4$ (52.2 mg, 0.3 mmol, 1.5 equiv.) and DMAc (1.0 mL) were added to a 25 mL flame-dried Young-type tube. The reaction mixture was stirred at 100 ^{o}C. After stirring for 12 hours and monitored by TLC, the reaction was cooled to room temperature. Yields were determined by GC with octadecane as the internal standard. No desired product was obtained.

7.3 Sparging experiment set up

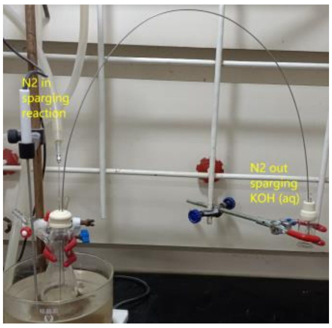

Supplementary Figure 4. Sparging experiment set up

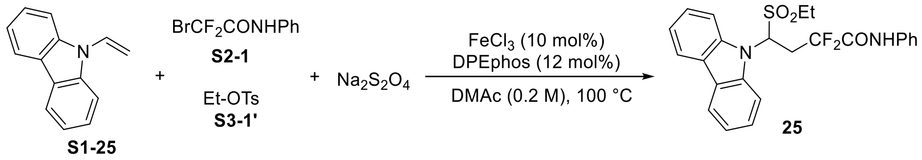

To a 50 mL Schlenk tube equipped N_2 vent line through KOH (5N) trap was charged with $FeCl_2$ (12.7 mg, 0.10 mmol, 10 mol%), DPEphos (64.6 mg, 0.12 mmol, 12 mol%), 9-vinyl-9*H*-carbazole (**S1-25**, 193.2 mg, 1.0 mmol, 1.0 equiv.), 2-bromo-2,2-difluoro-*N*-phenylacetamide (**S2-1**, 300.0 mg, 1.2 mmol, 1.2 equiv.), ethyl 4-methylbenzenesulfona (**S3-1'**, 300.4 mg, 1.5 mmol, 1.5 equiv.), $Na_2S_2O_4$ (261.2 mg, 1.5 mmol, 1.5 equiv.) and DMAc (5.0 mL). The mixture was stirred and was degassed via sparging with N_2 at ambient temperature for 1 h, whereupon the mixture was heated to 100 ℃ with continued stirring and sparging for 12 h. The solution was cooled to ambient temperature. The reaction mixture was diluted with H_2O. Then aqueous phase was extracted with EtOAc (3 × 10 mL). The combined organic extracts were washed with brine, dried over Na_2SO_4, filtered, and concentrated in vacuo. The residue was purified by flash chromatography (PE/EA = 5/1) to give the desired product **23** in 37% isolated yield. The KOH solution (liquid **A**) was detected by potassium dichromate solution.

$$SO_2 + 2KOH \longrightarrow K_2SO_3 + H_2O$$

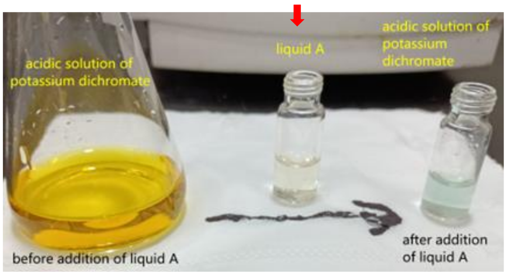

Chemical equation:

$$3SO_3^{2-}{}_{(aq)} + Cr_2O_7^{2-}{}_{(aq)} + 8H^+{}_{(aq)} \longrightarrow 3SO_4^{2-}{}_{(aq)} + 2Cr^{3+}{}_{(aq)} + 4H_2O$$

Supplementary Figure 5. Test for liquid **A** by potassium dichromate solution.

Comment: To investigate the liquid **A** containing potassium sulfite, an acidic solution of potassium

dichromate was prepared. When liquid A was added, the color of the solution changed from orange (Cr^{6+}) to green-purple (Cr^{3+}) in one minute, which could support the presence of potassium sulfite according to the above classical redox reaction.

7.4 Reaction profiles

The reaction profiles were produced according to the ***General procedure I*** and yields were determined by GC with octadecane as the internal standard.

Reaction profiles under standard conditions

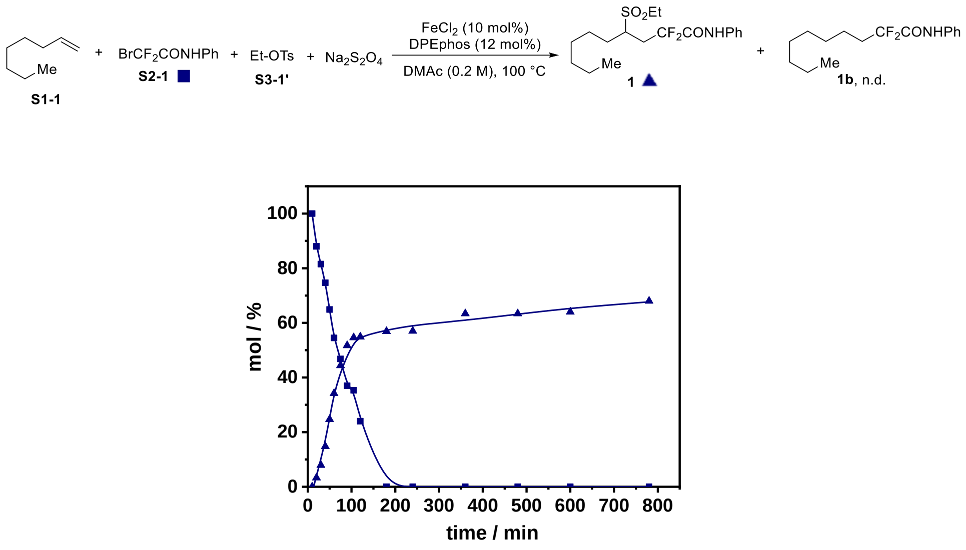

Supplementary Figure 6. Reaction profiles under standard conditions.

Reaction profiles under standard conditions without $FeCl_2$ and DPEphos

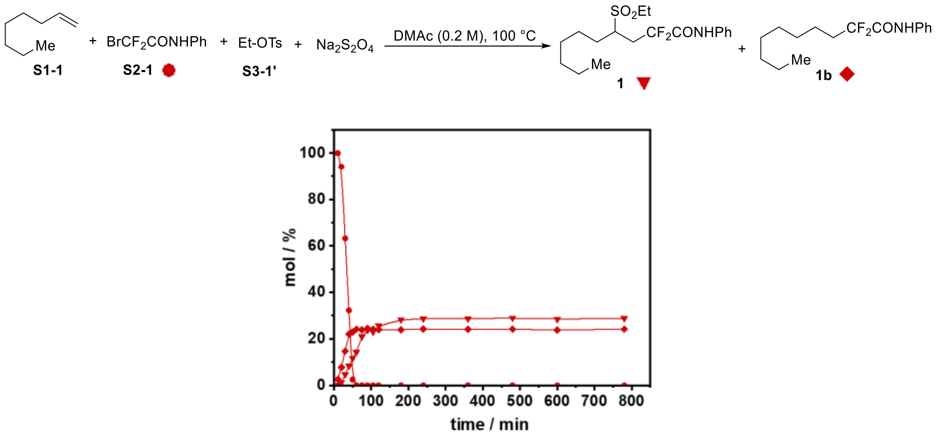

Supplementary Figure 7. Reaction profiles under standard conditions without $FeCl_2$ and DPEphos.

8. X-ray Crystal Structure Analysis

8.1 X-ray crystal structure of 27

Crystallographic files (CDCC: 2294876 (**27**)).The structure is of publishable quality (no A-alerts and B-alerts, see CIF/checkCIF) with a R1 value of 3.89%.

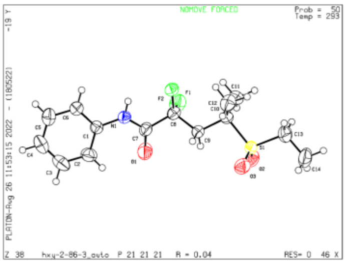

Supplementary Table 5. Crystal data and structure refinement for **27**

Identification code	hxy-2-86-3_auto
Empirical formula	$C_{14}H_{19}F_2NO_3S$
Formula weight	319.36
Temperature/K	293(2)
Crystal system	orthorhombic
Space group	$P2_12_12_1$
a/Å	8.25632(18)
b/Å	10.4634(2)
c/Å	17.7497(4)
α/°	90
β/°	90
γ/°	90
Volume/Å^3	1533.39(6)
Z	4
ρ_{calc}g/cm^3	1.383
μ/mm^{-1}	2.171
F(000)	672.0
Crystal size/mm^3	0.22 × 0.21 × 0.17
Radiation	Cu Kα (λ = 1.54184)
2Θ range for data collection/°	9.812 to 145.85
Index ranges	$-10 \leq h \leq 9$, $-9 \leq k \leq 12$, $-18 \leq l \leq 21$
Reflections collected	3897
Independent reflections	2485 [R_{int} = 0.0187, R_{sigma} = 0.0246]
Data/restraints/parameters	2485/0/194
Goodness-of-fit on F^2	1.076
Final R indexes [I>=2σ (I)]	R_1 = 0.0389, wR_2 = 0.1056
Final R indexes [all data]	R_1 = 0.0397, wR_2 = 0.1068
Largest diff. peak/hole / e Å^{-3}	0.37/-0.32

8.2 X-ray crystal structure of 38

Crystallographic files (CDCC: 2294877 (**38**)). The structure is of publishable quality (no A-alerts and B-alerts, see CIF/checkCIF) with a R1 value of 4.16%.

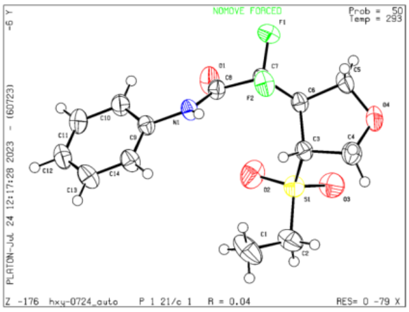

Supplementary Table 6. Crystal data and structure refinement for **38**

Identification code	HXY-0724_auto
Empirical formula	$C_{14}H_{17}F_2NO_4S$
Formula weight	333.34
Temperature/K	293
Crystal system	monoclinic
Space group	$P2_1/c$
a/Å	15.7106(3)
b/Å	5.16460(10)
c/Å	18.3287(4)
α/°	90
β/°	94.834(2)
γ/°	90
Volume/Å^3	1481.88(5)
Z	4
ρ_{calc}g/cm^3	1.494
μ/mm^{-1}	2.328
F(000)	696.0
Crystal size/mm^3	0.2 × 0.15 × 0.1
Radiation	Cu Kα (λ = 1.54184)
2Θ range for data collection/°	9.686 to 145.79
Index ranges	$-19 \leq h \leq 17$, $-4 \leq k \leq 6$, $-22 \leq l \leq 22$
Reflections collected	5355
Independent reflections	2889 [R_{int} = 0.0230, R_{sigma} = 0.0293]
Data/restraints/parameters	2889/0/200
Goodness-of-fit on F^2	1.064
Final R indexes [I>=2σ (I)]	R_1 = 0.0416, wR_2 = 0.1055
Final R indexes [all data]	R_1 = 0.0467, wR_2 = 0.1102
Largest diff. peak/hole / e Å^{-3}	0.20/-0.44

8.3 X-ray crystal structure of 40

Crystallographic files (CDCC: 2294886 (**40**)). The structure is of publishable quality (no A-alerts and B-alerts, see CIF/checkCIF) with a R1 value of 5.90%.

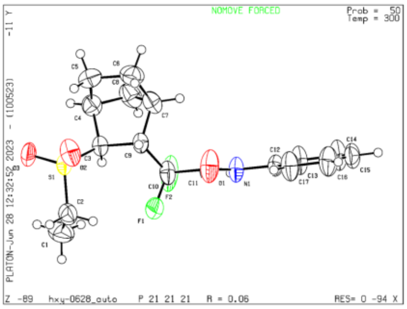

Supplementary Table 7. Crystal data and structure refinement for **40**

Identification code	HXY-0628_auto
Empirical formula	$C_{17}H_{21}F_2NO_3S$
Formula weight	357.41
Temperature/K	300
Crystal system	orthorhombic
Space group	$P2_12_12_1$
a/Å	8.5637(4)
b/Å	10.4718(3)
c/Å	19.3782(6)
α/°	90
β/°	90
γ/°	90
Volume/Å^3	1737.79(11)
Z	4
ρ_{calc}g/cm^3	1.366
μ/mm^{-1}	1.978
F(000)	752.0
Crystal size/mm^3	0.2 × 0.15 × 0.1
Radiation	Cu Kα (λ = 1.54184)
2Θ range for data collection/ °	9.128 to 146.216
Index ranges	-10 ≤ h ≤ 7, -12 ≤ k ≤ 12, -23 ≤ l ≤ 21
Reflections collected	4513
Independent reflections	3020 [R_{int} = 0.0395, R_{sigma} = 0.0484]
Data/restraints/parameters	3020/0/218
Goodness-of-fit on F^2	1.065
Final R indexes [I>=2σ (I)]	R_1 = 0.0590, wR_2 = 0.1556
Final R indexes [all data]	R_1 = 0.0657, wR_2 = 0.1710
Largest diff. peak/hole / e Å^{-3}	0.25/-0.59

8.4 X-ray crystal structure of Fe(DPEphos)Cl_2

Crystallographic files (CDCC: 2294887 (Fe(DPEphos)Cl_2)). The structure is of publishable quality (no A-alerts and B-alerts, see CIF/checkCIF) with a R1 value of 4.61%.

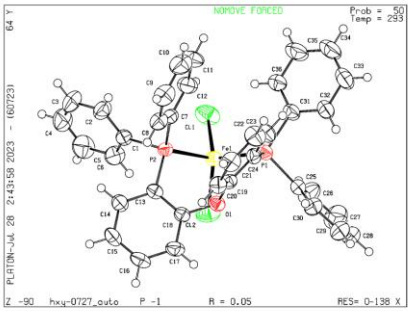

Supplementary Table 8. Crystal data and structure refinement for Fe(DPEphos)Cl_2

Identification code	HXY-0727_auto
Empirical formula	$C_{36}H_{28}Cl_2FeO_3P_2$
Formula weight	697.27
Temperature/K	293
Crystal system	triclinic
Space group	P-1
a/Å	10.1882(3)
b/Å	11.3490(3)
c/Å	18.0469(6)
α/°	78.732(2)
β/°	74.949(3)
γ/°	76.654(2)
Volume/Å^3	1940.20(11)
Z	2
ρ_{calc}g/cm^3	1.194
μ/mm^{-1}	5.402
F(000)	716.0
Crystal size/mm^3	0.2 × 0.15 × 0.1
Radiation	Cu Kα (λ = 1.54184)
2Θ range for data collection/°	8.092 to 145.854
Index ranges	$-12 \leq h \leq 12$, $-12 \leq k \leq 14$, $-22 \leq l \leq 21$
Reflections collected	13699
Independent reflections	7523 [R_{int} = 0.0357, R_{sigma} = 0.0435]
Data/restraints/parameters	7523/0/379
Goodness-of-fit on F^2	1.011
Final R indexes [I>=2σ (I)]	R_1 = 0.0461, wR_2 = 0.1227
Final R indexes [all data]	R_1 = 0.0546, wR_2 = 0.1294
Largest diff. peak/hole / e Å^{-3}	0.62/-0.59

9. Supplementary NMR Spectra of Products

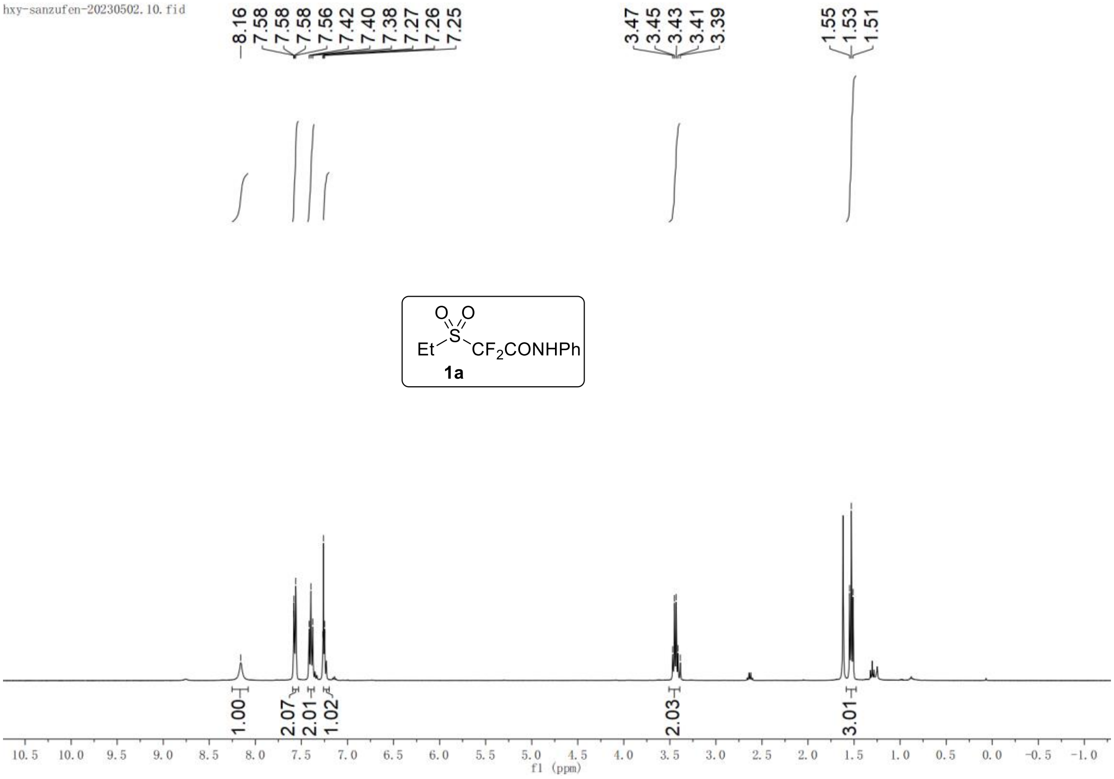

Supplementary Figure 8. ^{1}H NMR (400 MHz, $CDCl_3$) spectra of **1a**

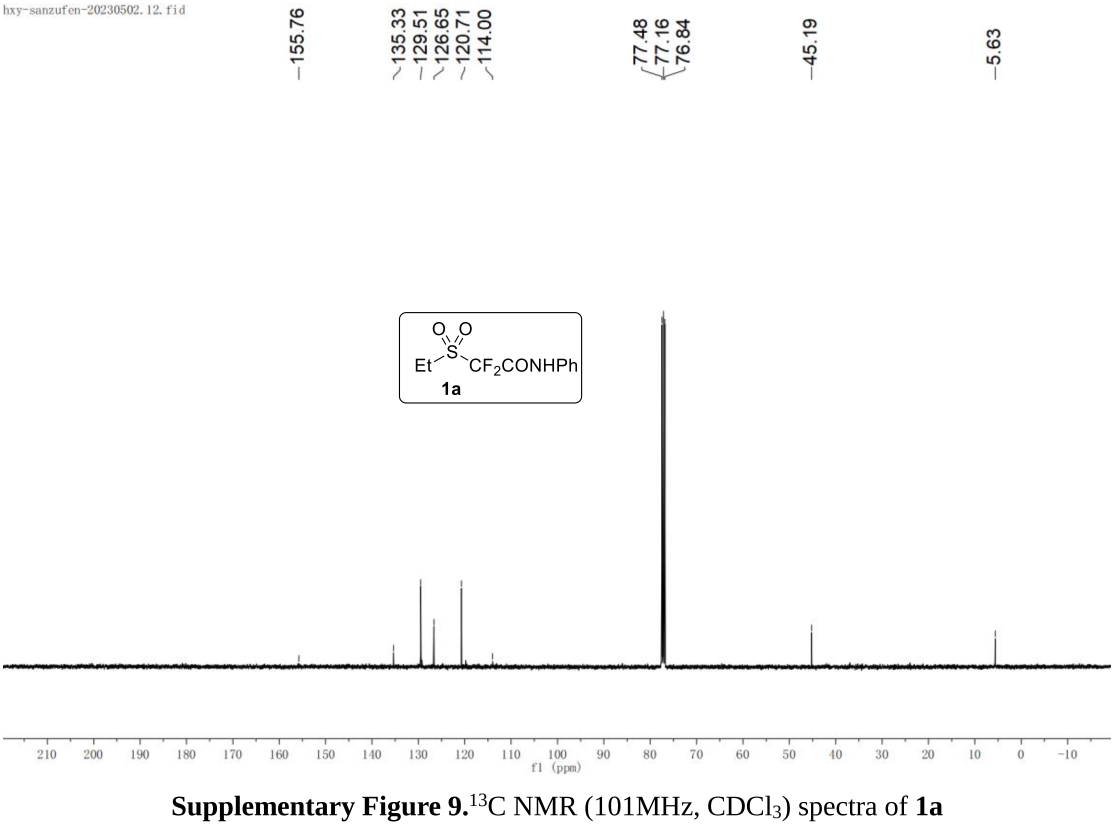

Supplementary Figure 9.^{13}C NMR (101MHz, $CDCl_3$) spectra of **1a**

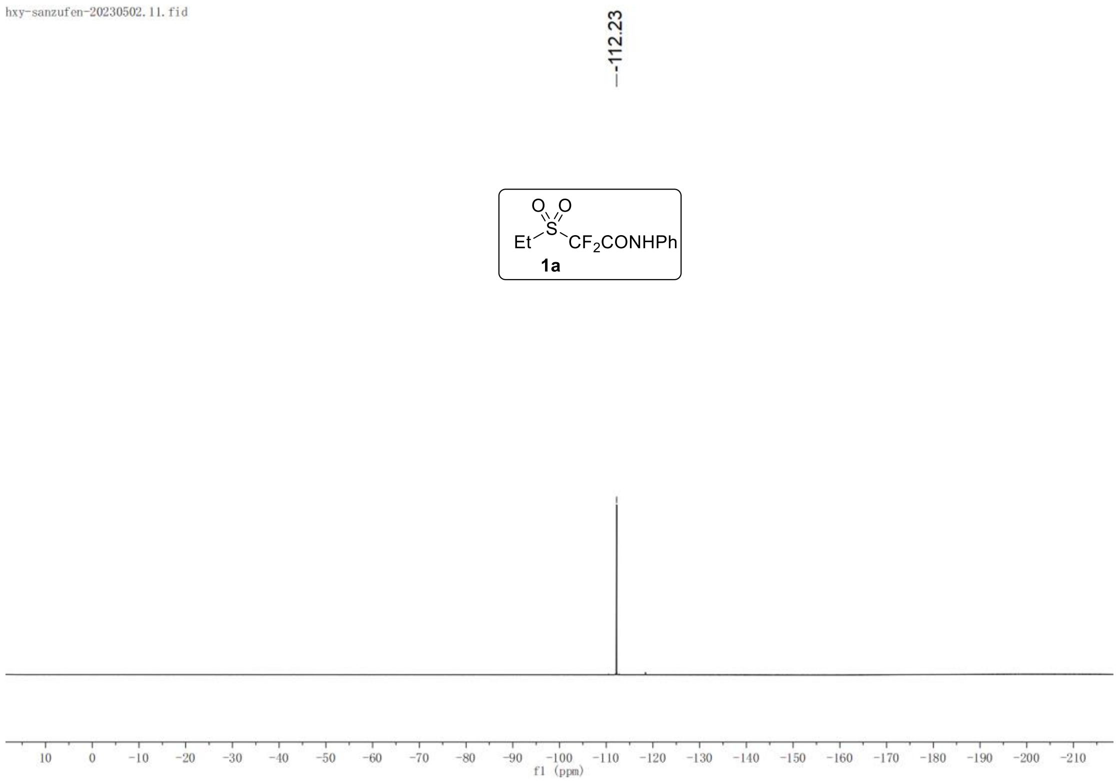

Supplementary Figure 10. ^{19}F NMR (376 MHz, $CDCl_3$) spectra of **1a**

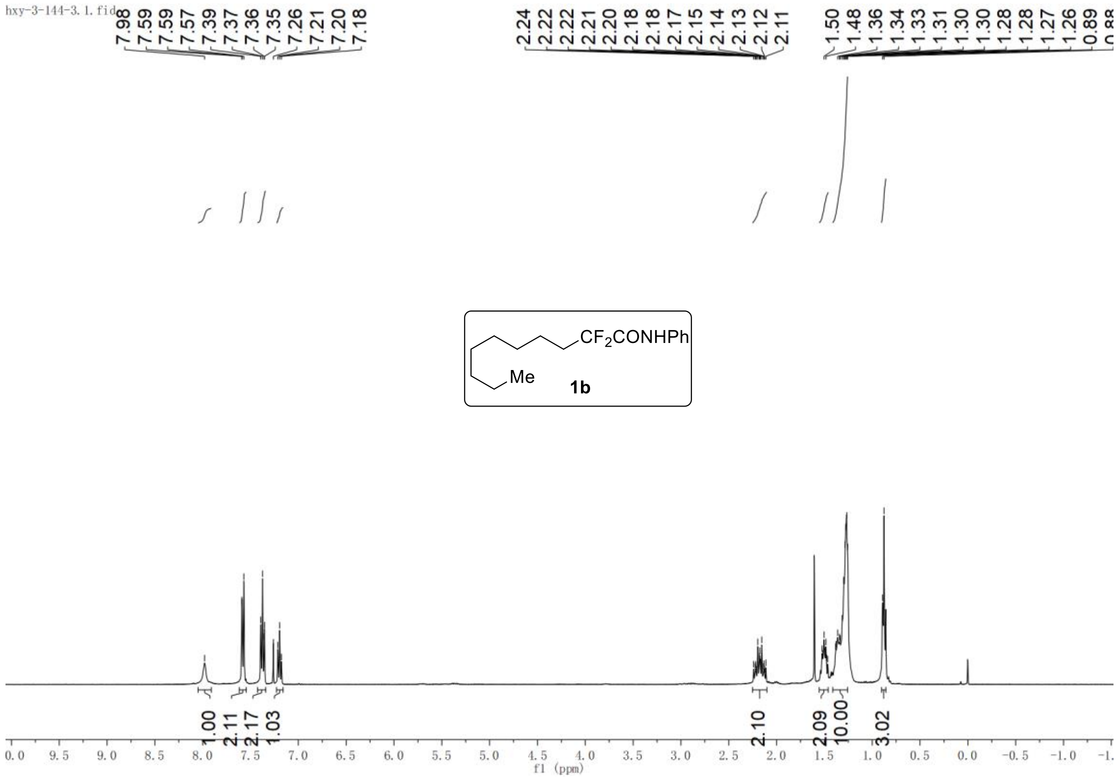

Supplementary Figure 11. ^{1}H NMR (400 MHz, $CDCl_3$) spectra of **1b**

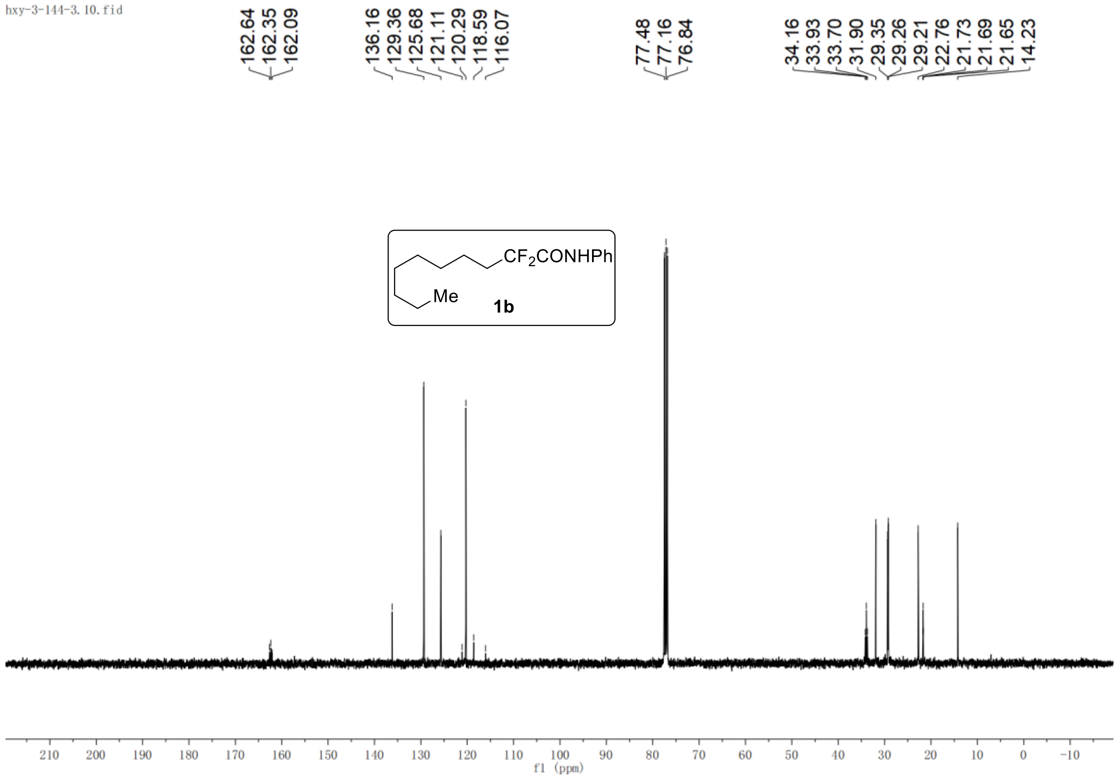

Supplementary Figure 12. ^{13}C NMR (101 MHz, $CDCl_3$) spectra of **1b**

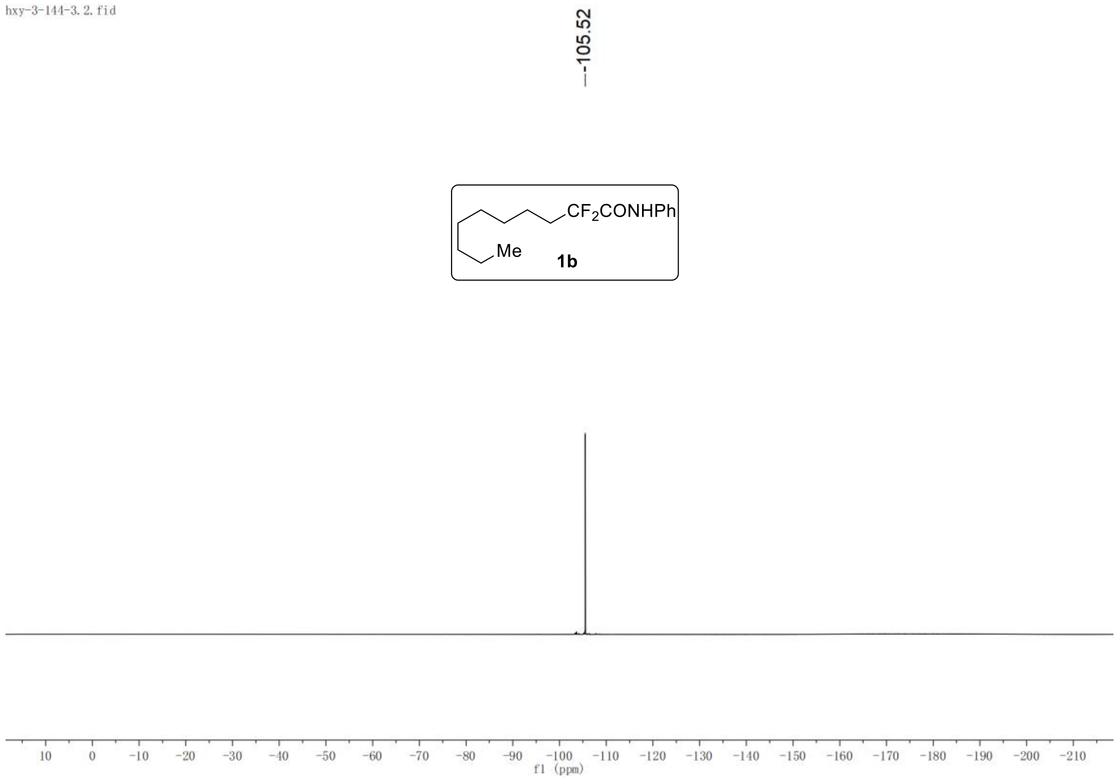

Supplementary Figure 13. ^{19}F NMR (376 MHz, $CDCl_3$) spectra of **1b**

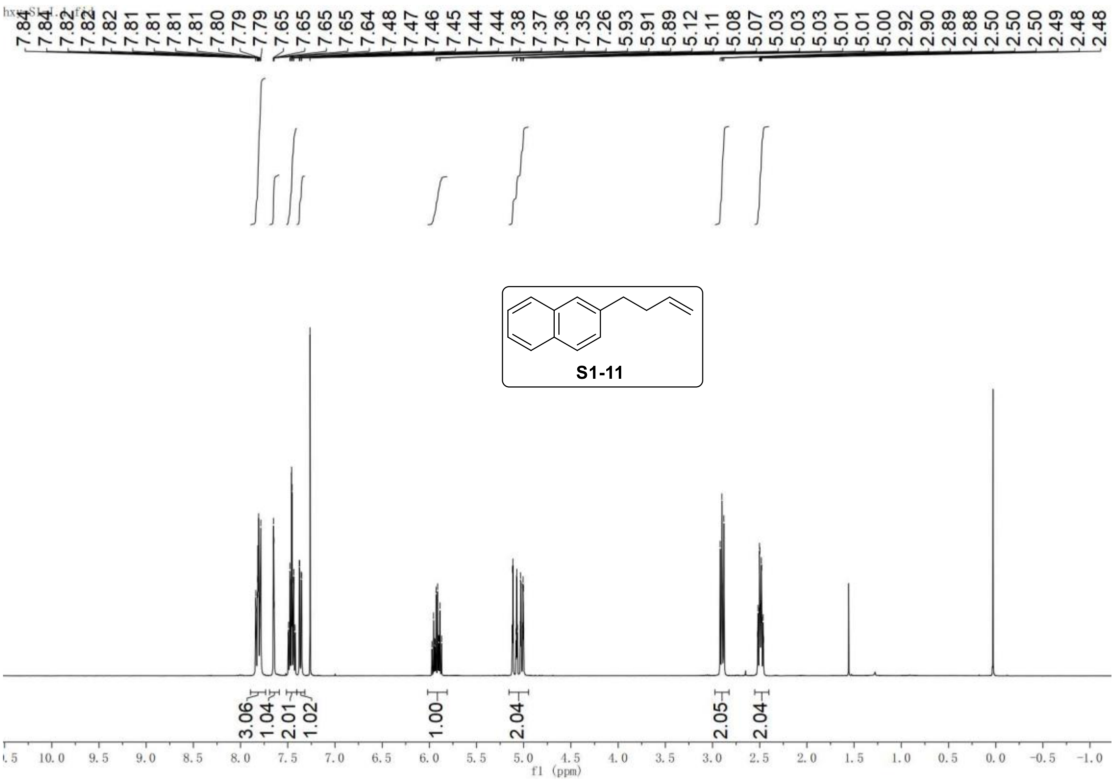

Supplementary Figure 14. ^{1}H NMR (400 MHz, $CDCl_3$) spectra of **S1-11**

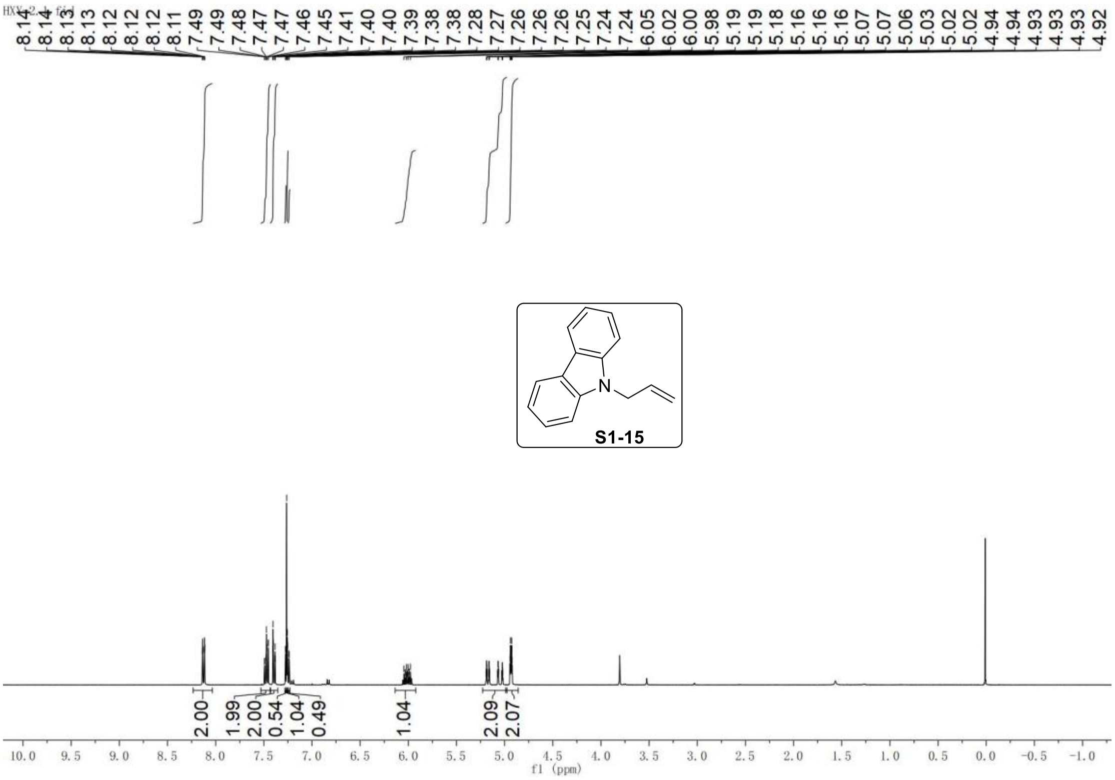

Supplementary Figure 15. ^{1}H NMR (400 MHz, $CDCl_3$) spectra of **S1-15**

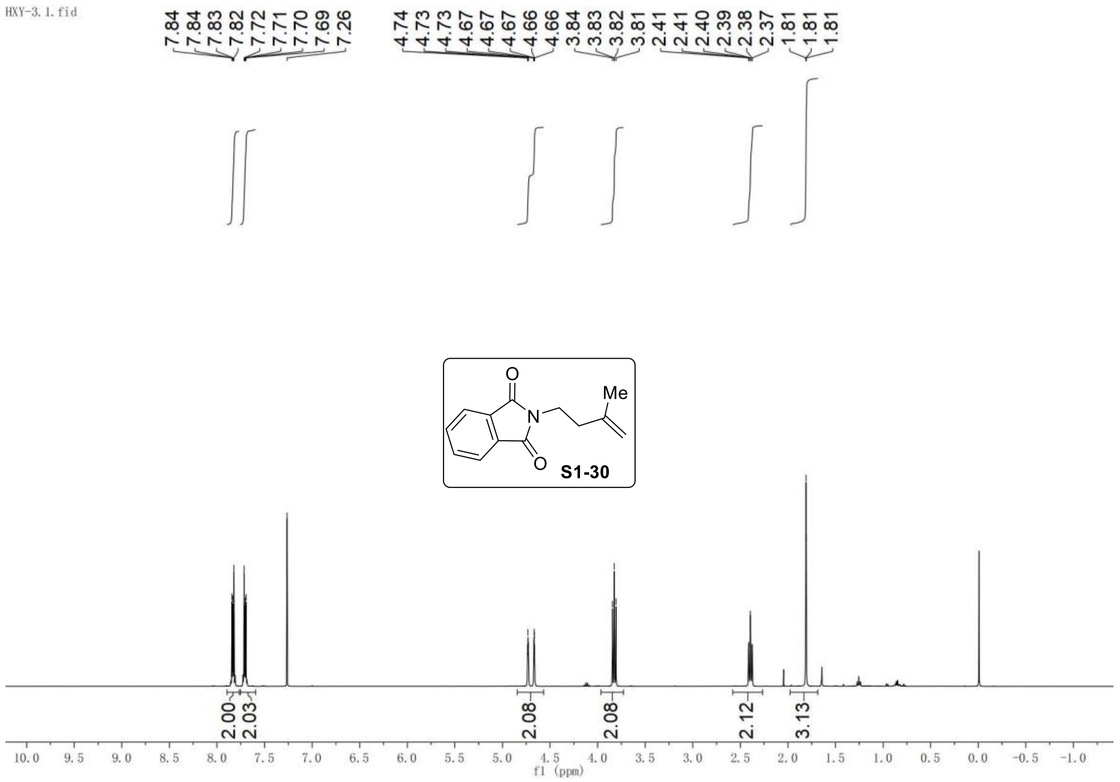

Supplementary Figure 16. ^{1}H NMR (400 MHz, $CDCl_3$) spectra of **S1-30**

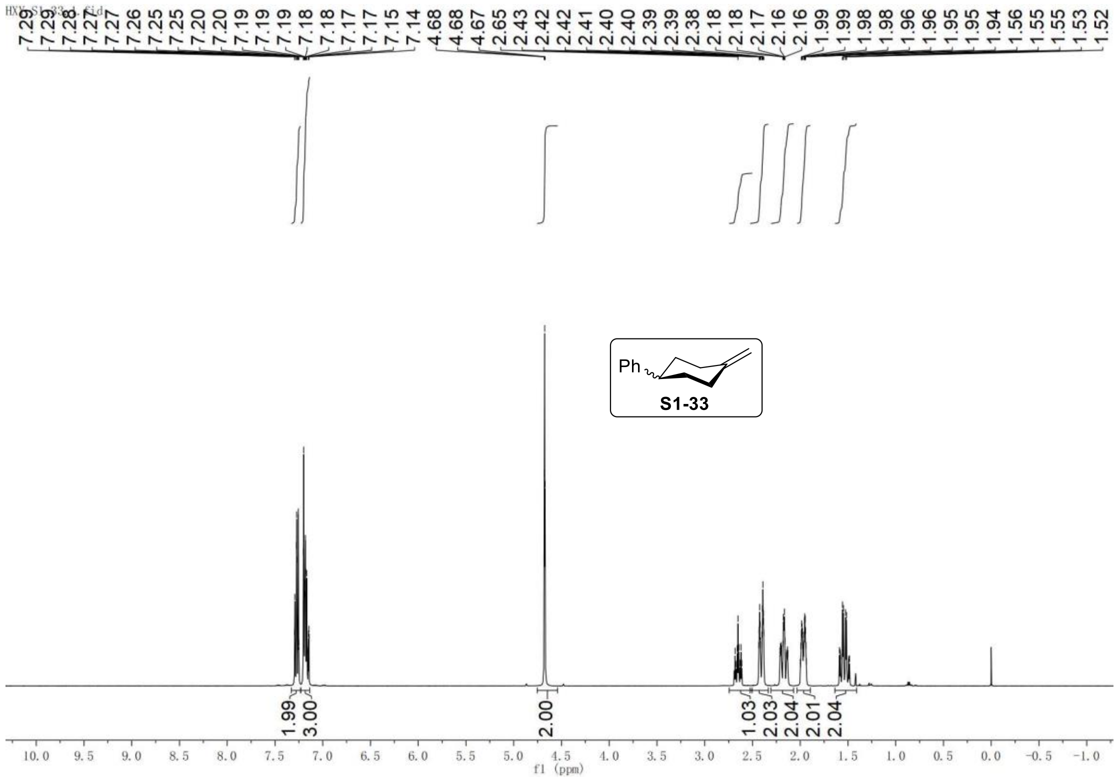

Supplementary Figure 17. ^{1}H NMR (400 MHz, $CDCl_3$) spectra of **S1-33**

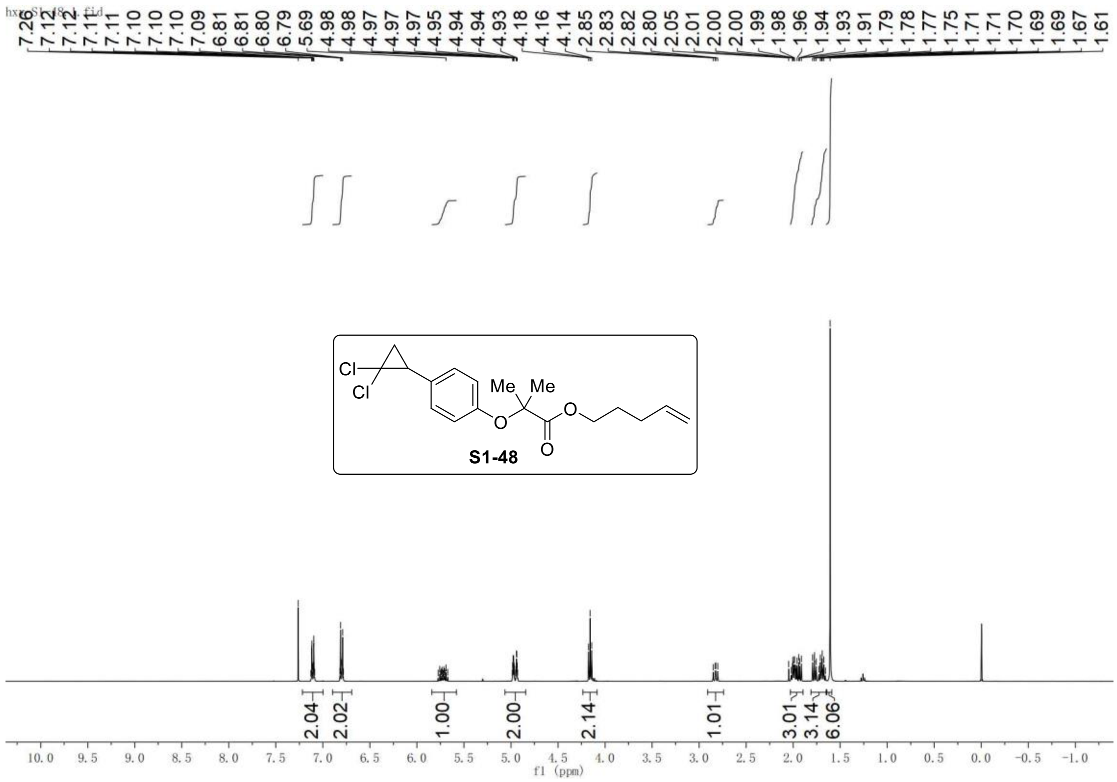

Supplementary Figure 18. ^{1}H NMR (400 MHz, $CDCl_3$) spectra of **S1-48**

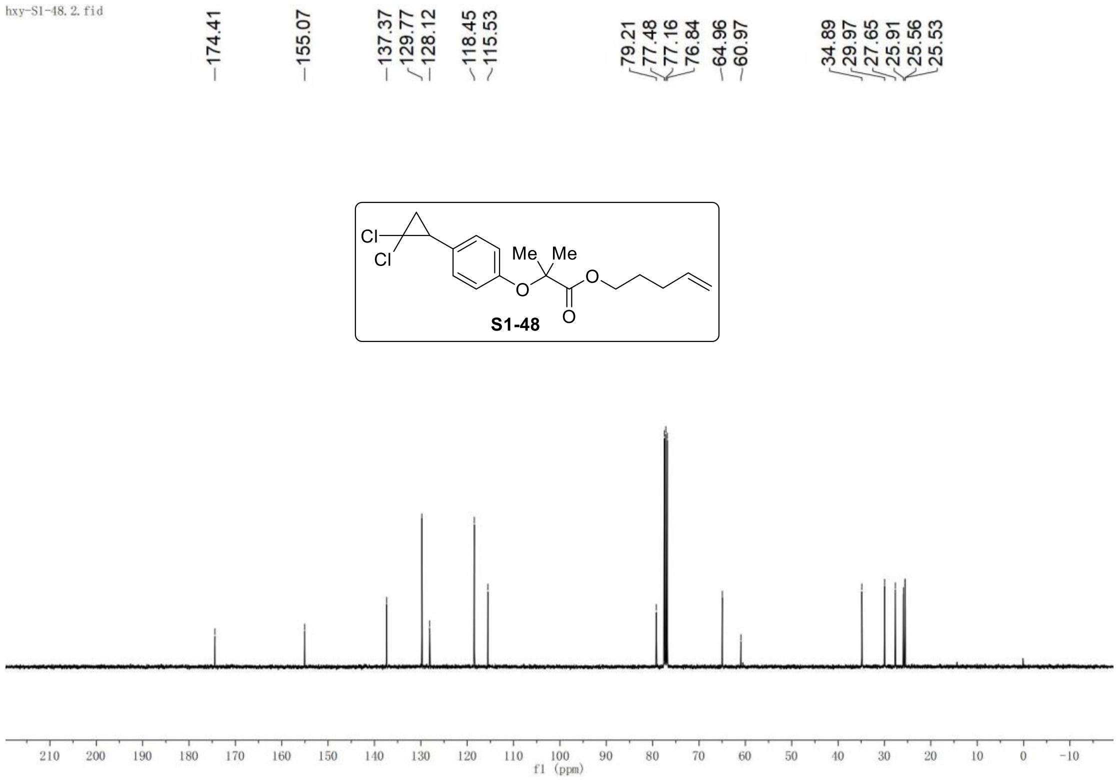

Supplementary Figure 19. ^{13}C NMR (101 MHz, $CDCl_3$) spectra of **S1-48**

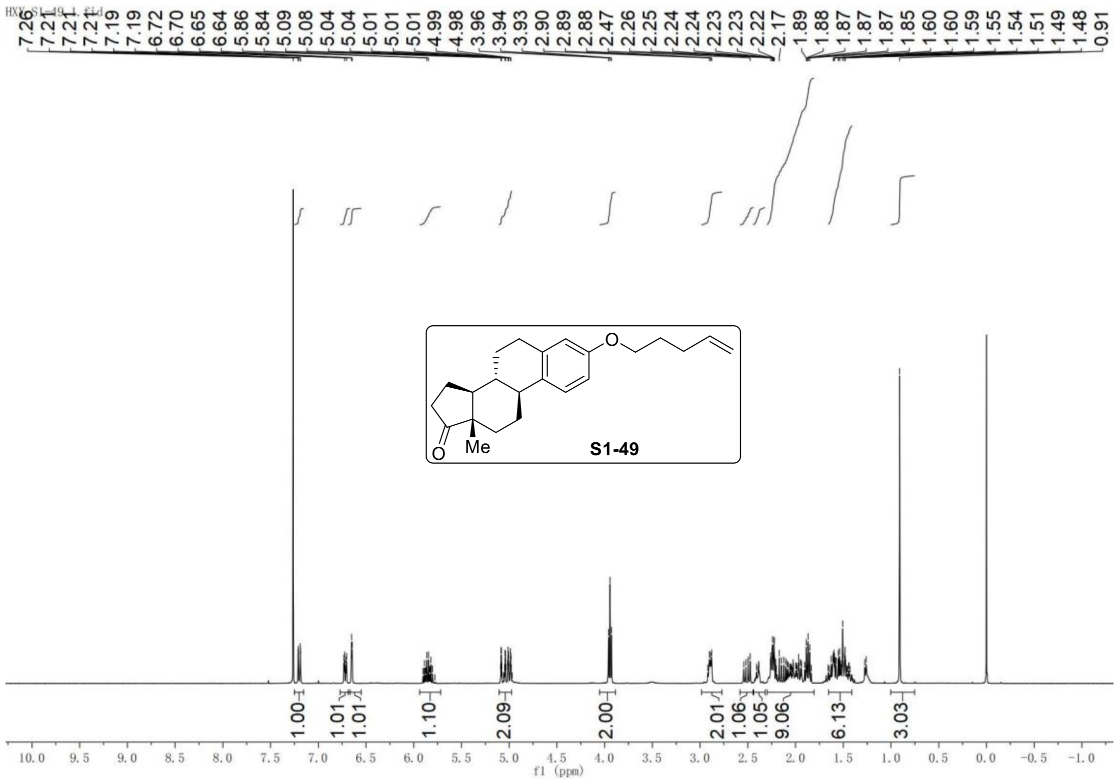

Supplementary Figure 20. ^{1}H NMR (400 MHz, $CDCl_3$) spectra of **S1-49**

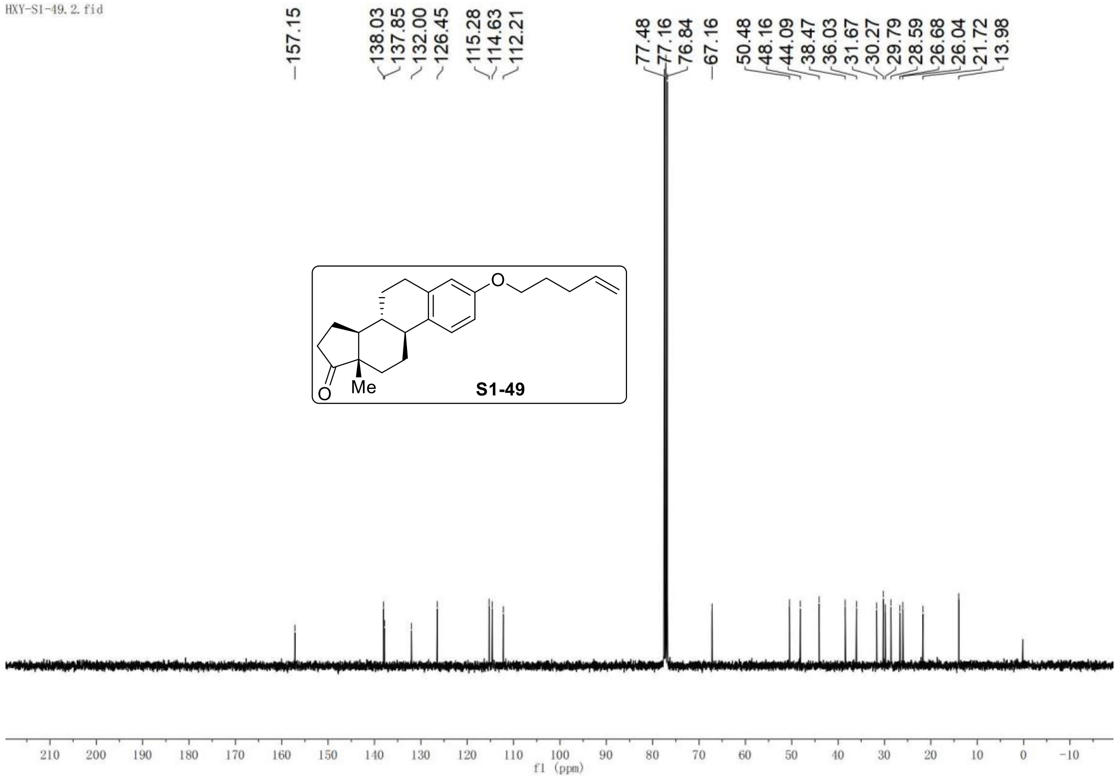

Supplementary Figure 21. ^{13}C NMR (101 MHz, $CDCl_3$) spectra of **S1-49**

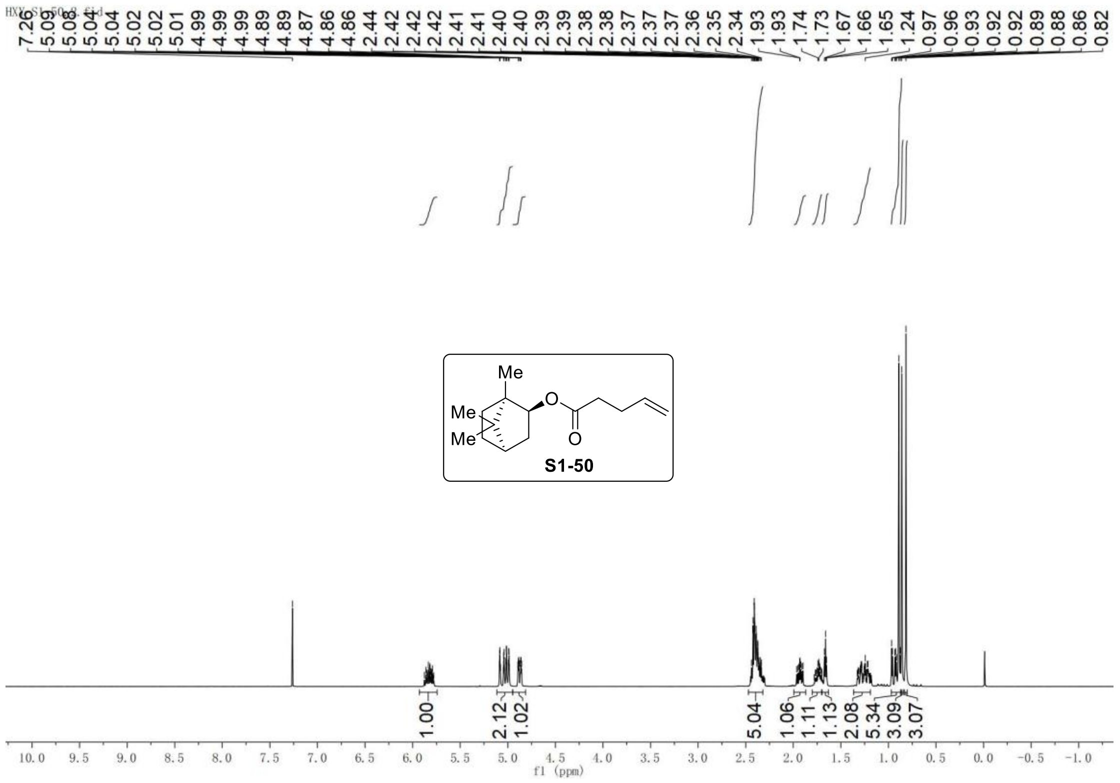

Supplementary Figure 22. ^{1}H NMR (400 MHz, $CDCl_3$) spectra of **S1-50**

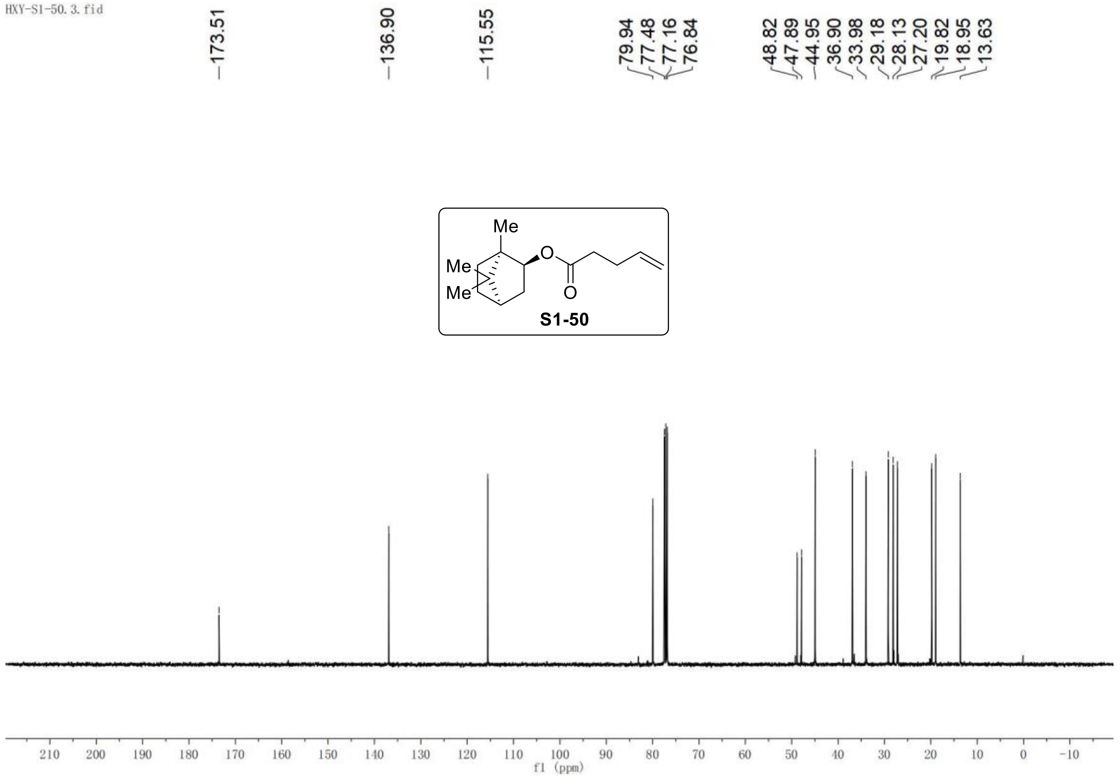

Supplementary Figure 23. ^{13}C NMR (101 MHz, $CDCl_3$) spectra of **S1-50**

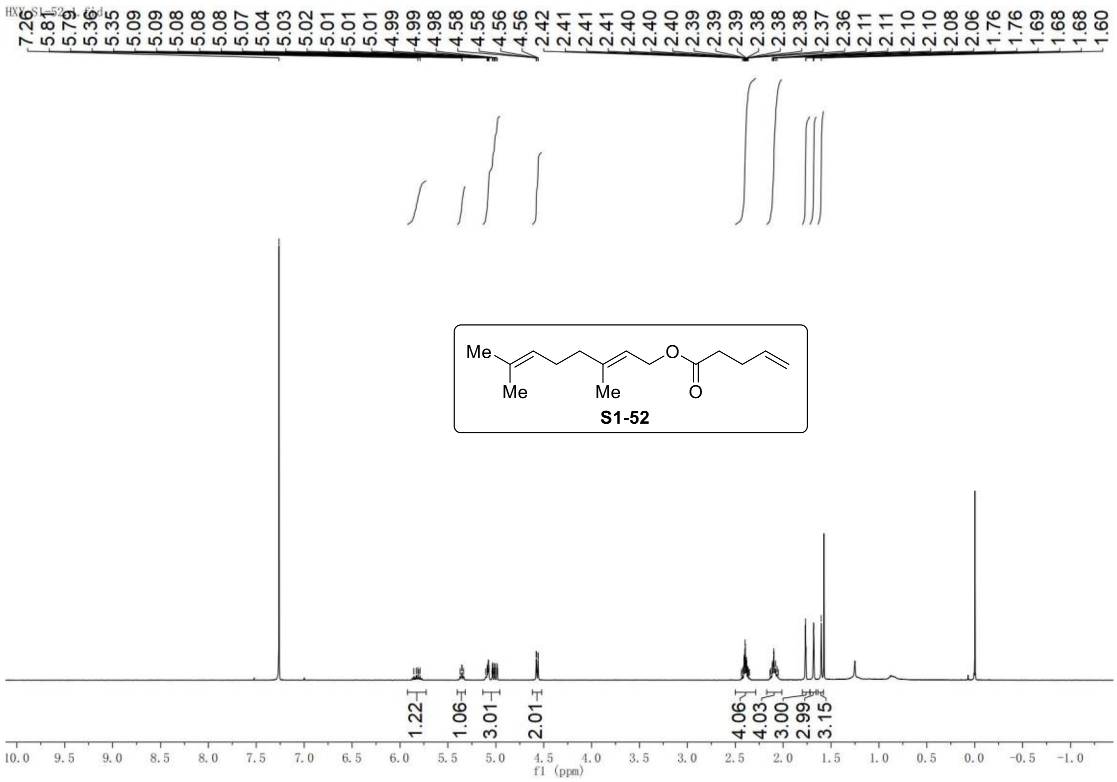

Supplementary Figure 24. ^{1}H NMR (400 MHz, $CDCl_3$) spectra of **S1-52**

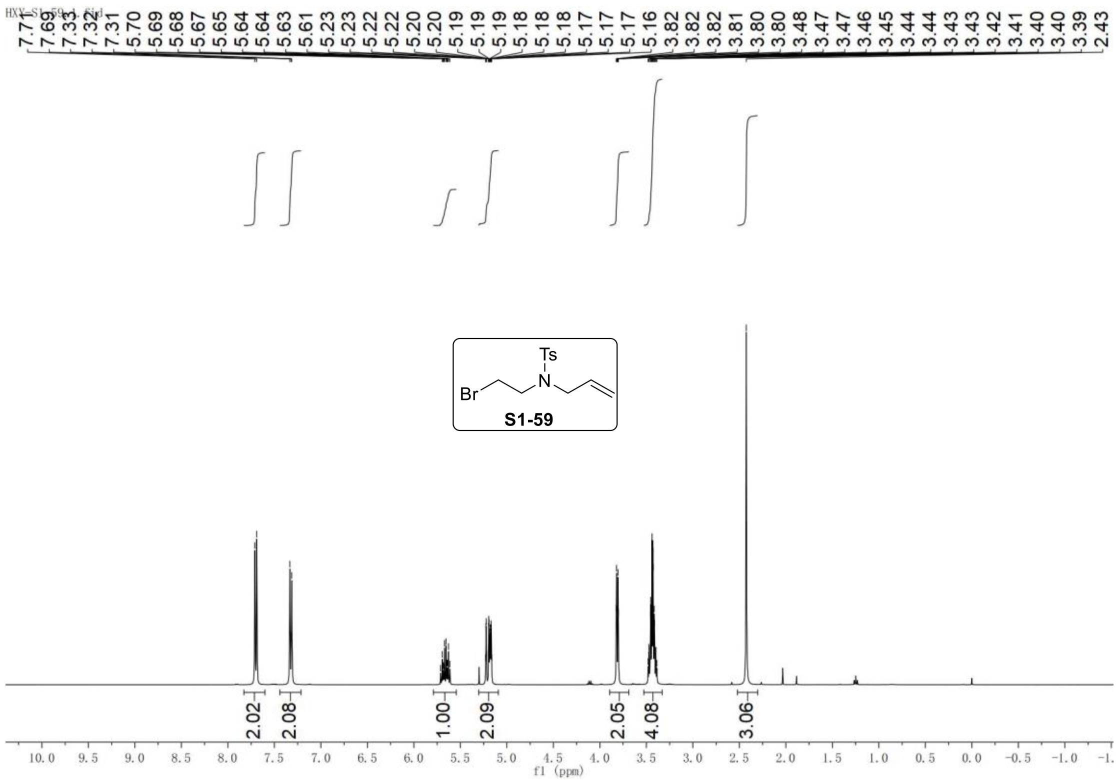

Supplementary Figure 25. ^{1}H NMR (400 MHz, $CDCl_3$) spectra of **S1-59**

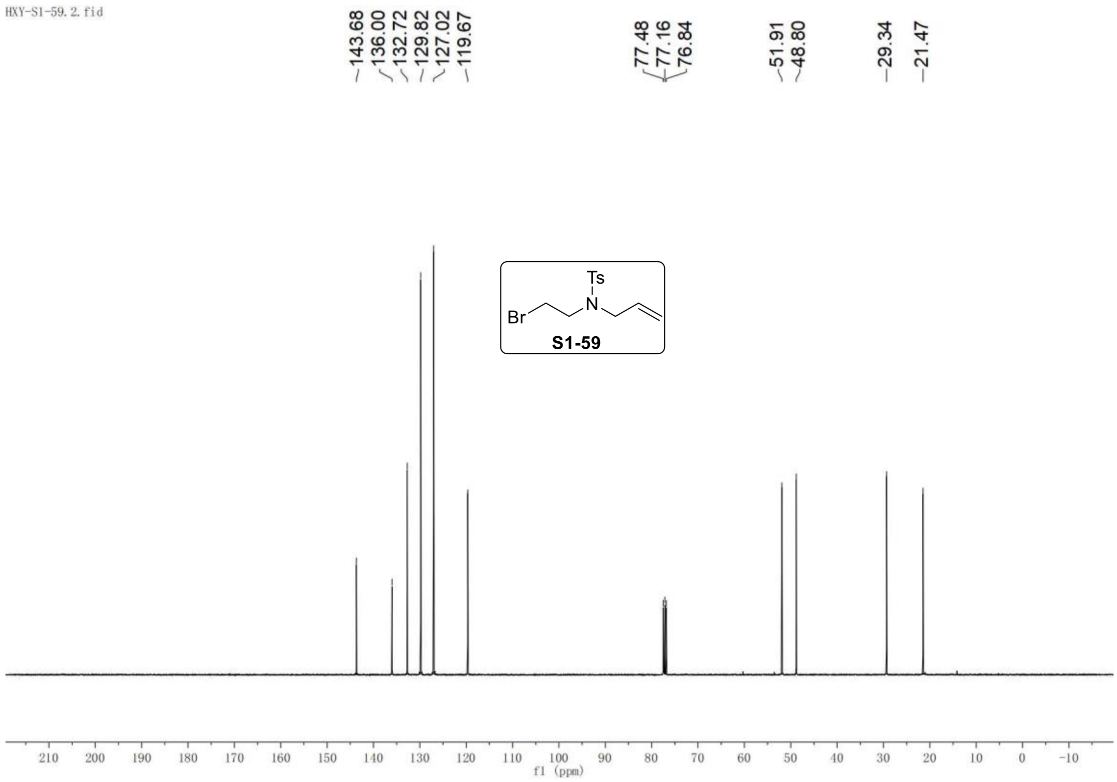

Supplementary Figure 26. ^{13}C NMR (101 MHz, $CDCl_3$) spectra of **S1-59**

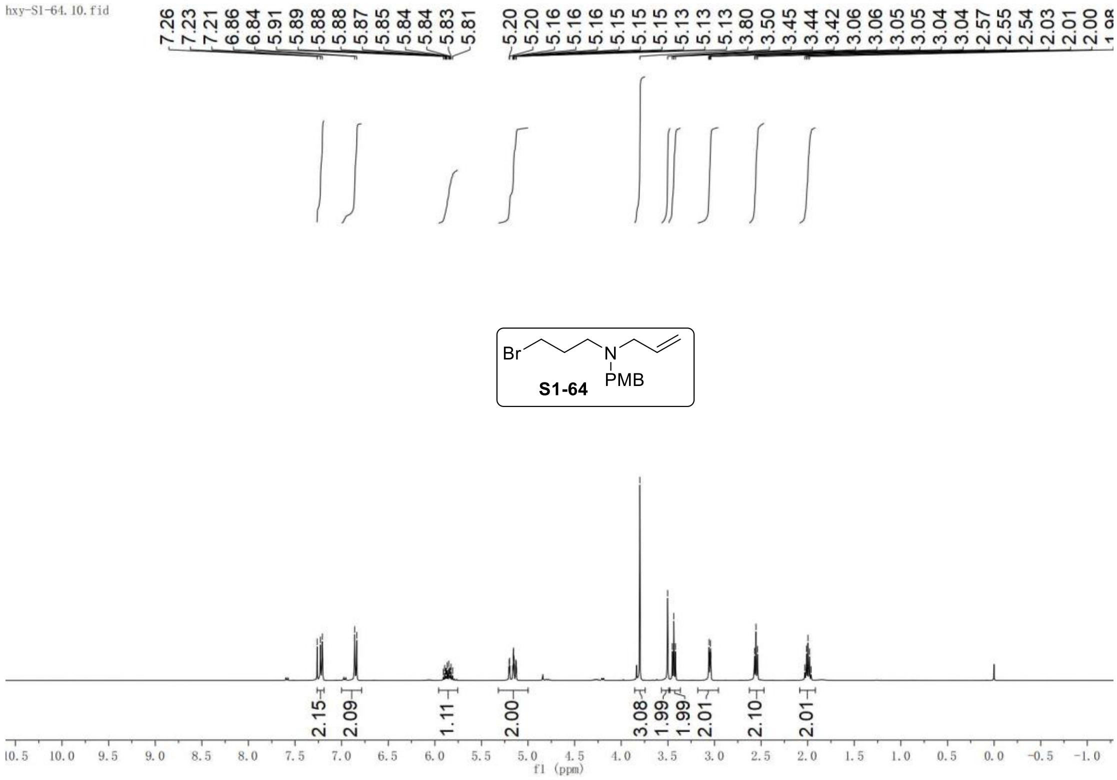

Supplementary Figure 27. ^{1}H NMR (400 MHz, $CDCl_3$) spectra of **S1-64**

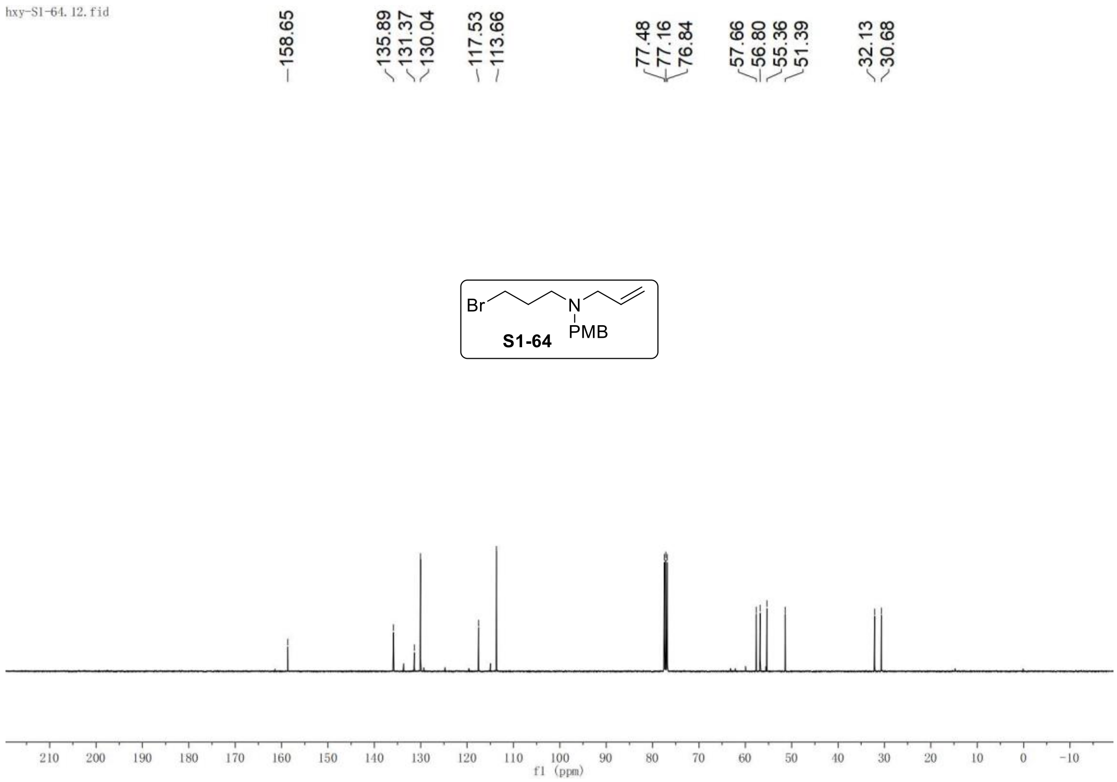

Supplementary Figure 28. ^{13}C NMR (101 MHz, $CDCl_3$) spectra of **S1-64**

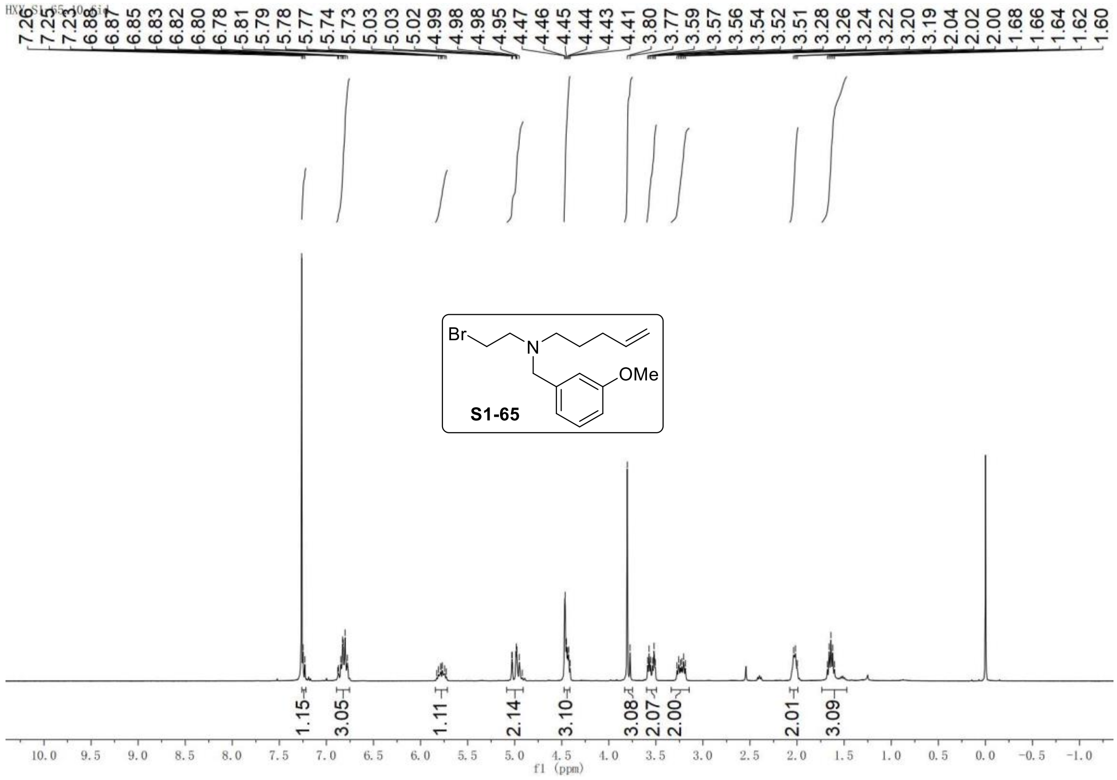

Supplementary Figure 29. ^{1}H NMR (400 MHz, $CDCl_3$) spectra of **S1-65**

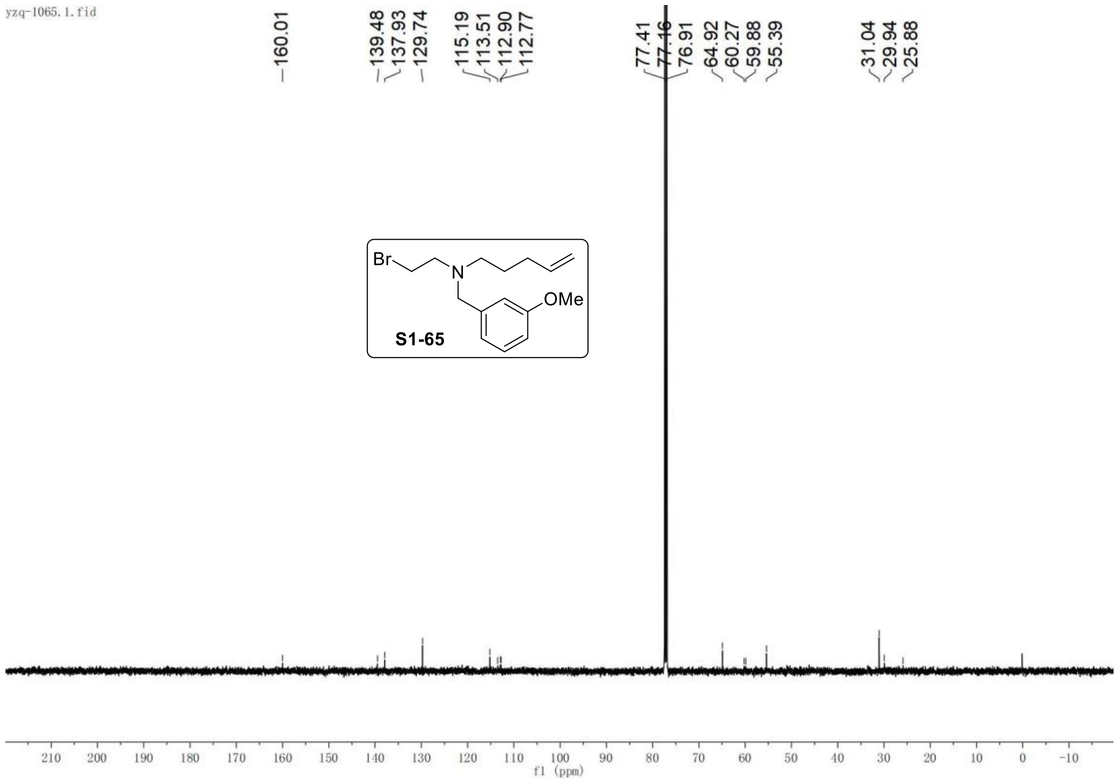

Supplementary Figure 30. ^{13}C NMR (126 MHz, $CDCl_3$) spectra of **S1-65**

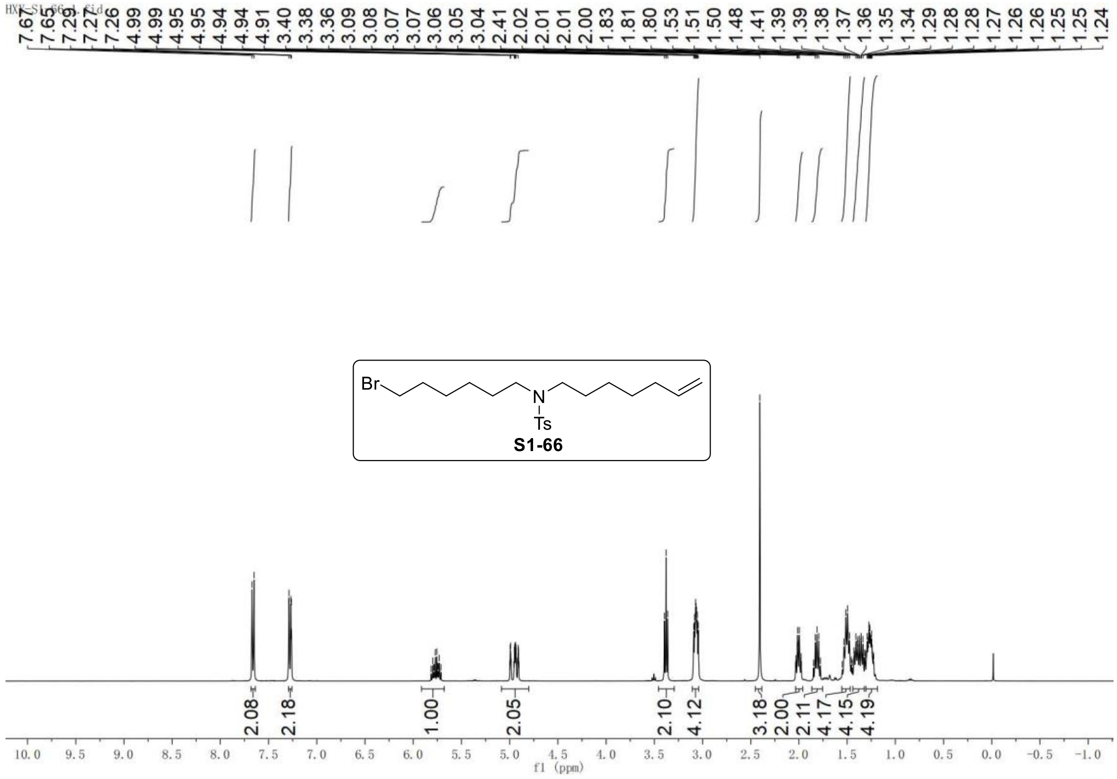

Supplementary Figure 31. ^{1}H NMR (400 MHz, $CDCl_3$) spectra of **S1-66**

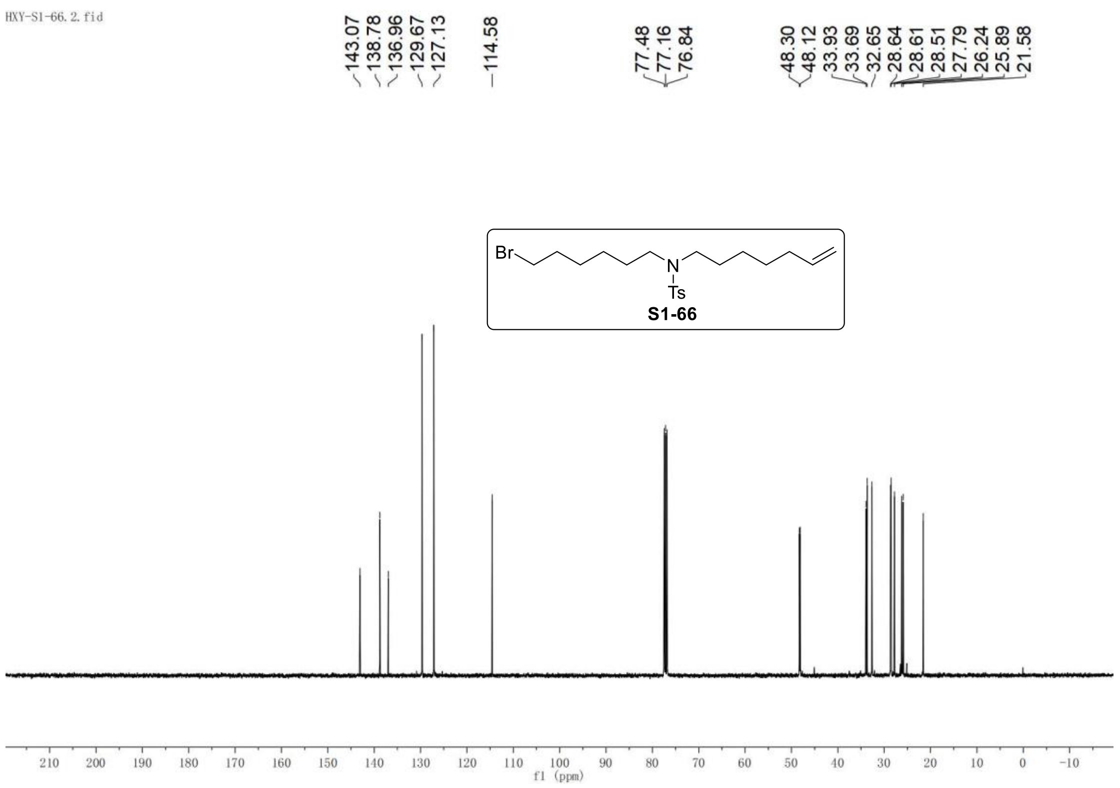

Supplementary Figure 32. ^{13}C NMR (101 MHz, $CDCl_3$) spectra of **S1-66**

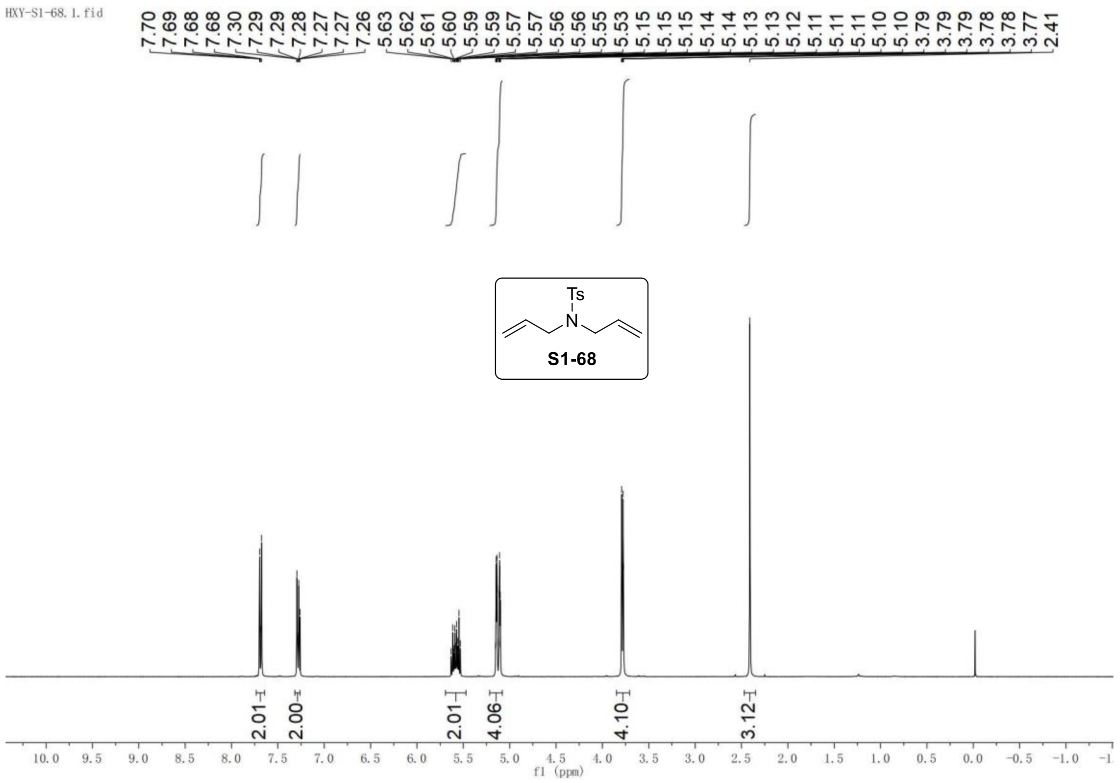

Supplementary Figure 33. ^{1}H NMR (400 MHz, $CDCl_3$) spectra of **S1-68**

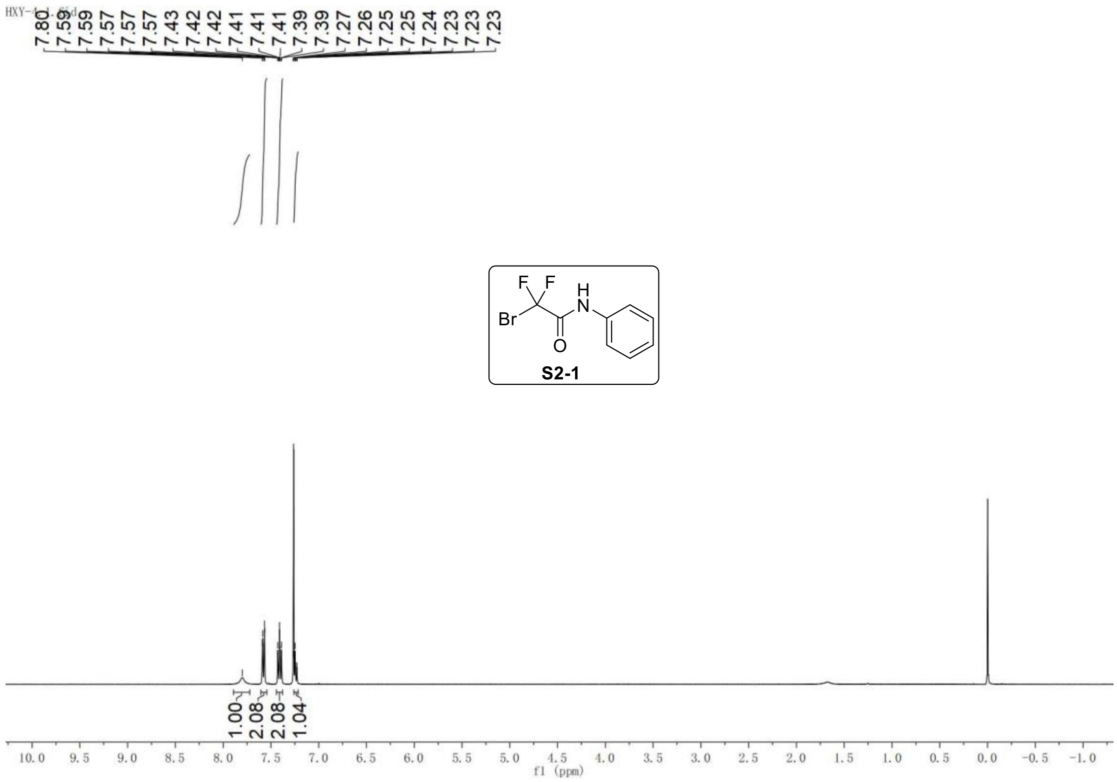

Supplementary Figure 34. ^{1}H NMR (400 MHz, $CDCl_3$) spectra of **S2-1**

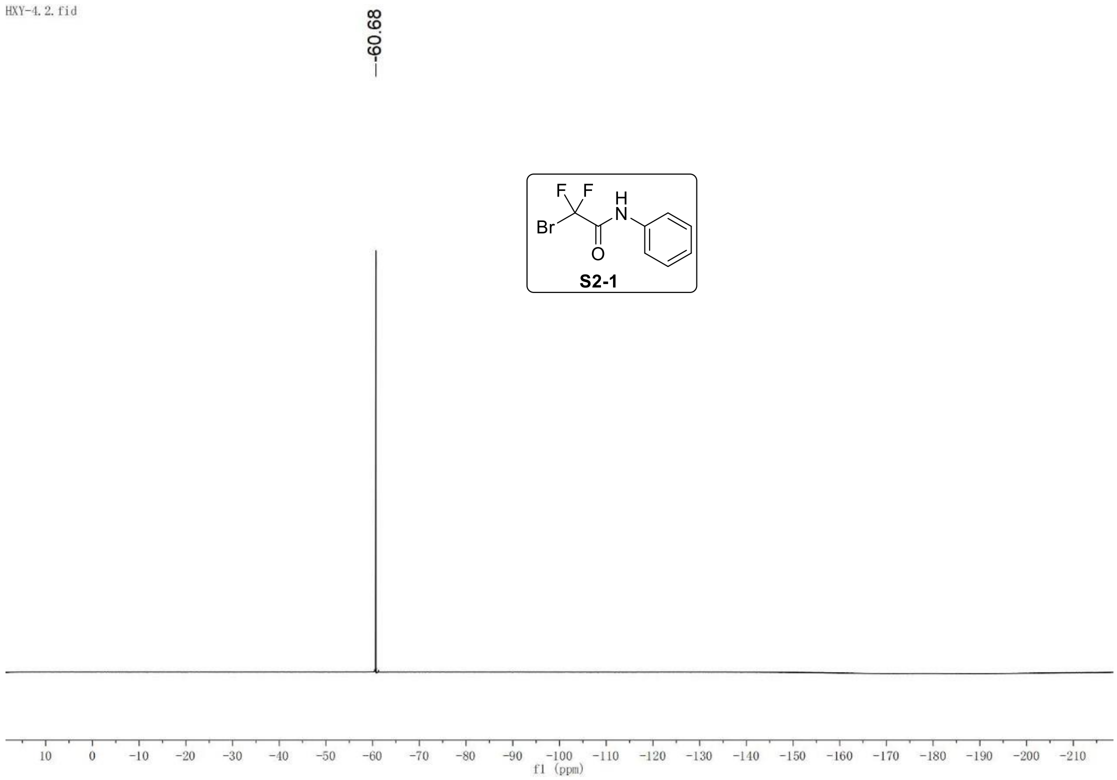

Supplementary Figure 35. ^{19}F NMR (376 MHz, $CDCl_3$) spectra of **S2-1**

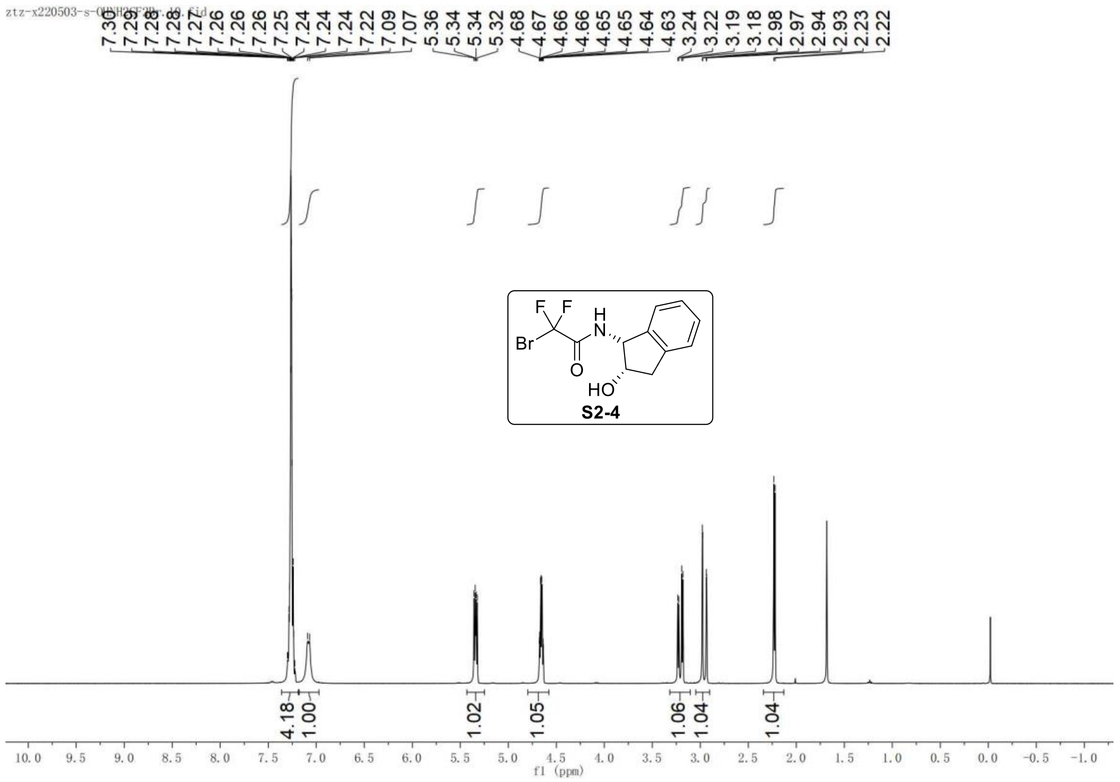

Supplementary Figure 36. ^{1}H NMR (400 MHz, $CDCl_3$) spectra of **S2-4**

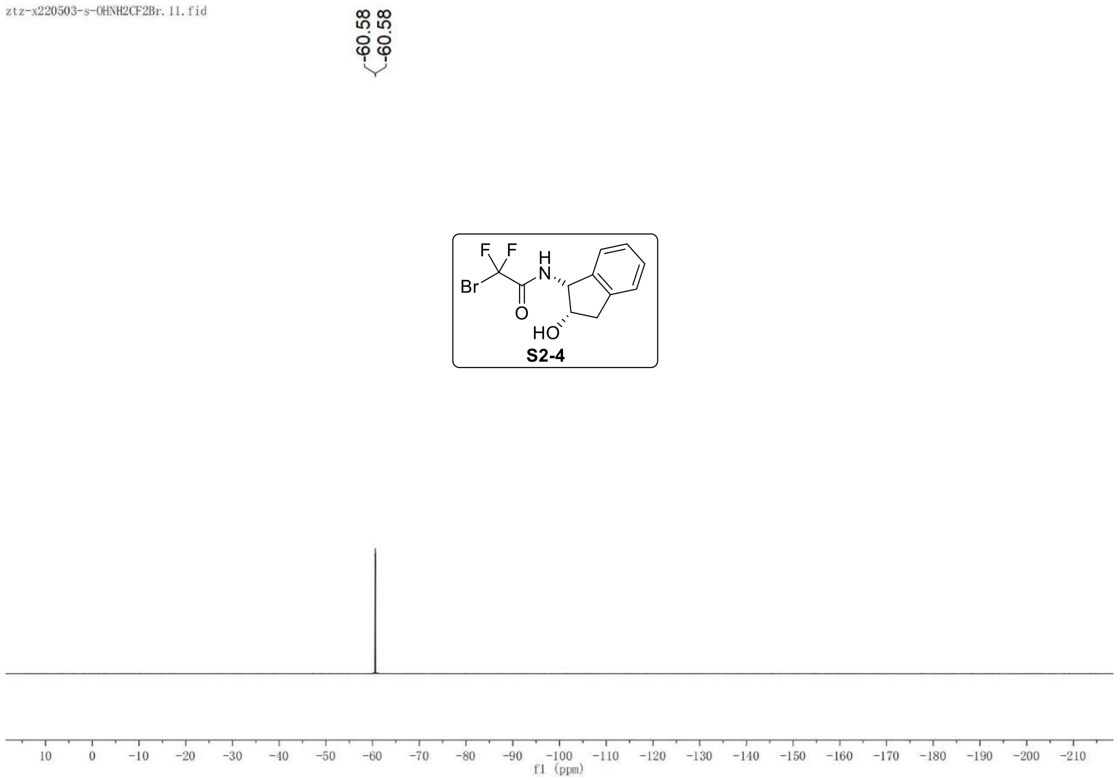

Supplementary Figure 37. ^{19}F NMR (376 MHz, $CDCl_3$) spectra of **S2-4**

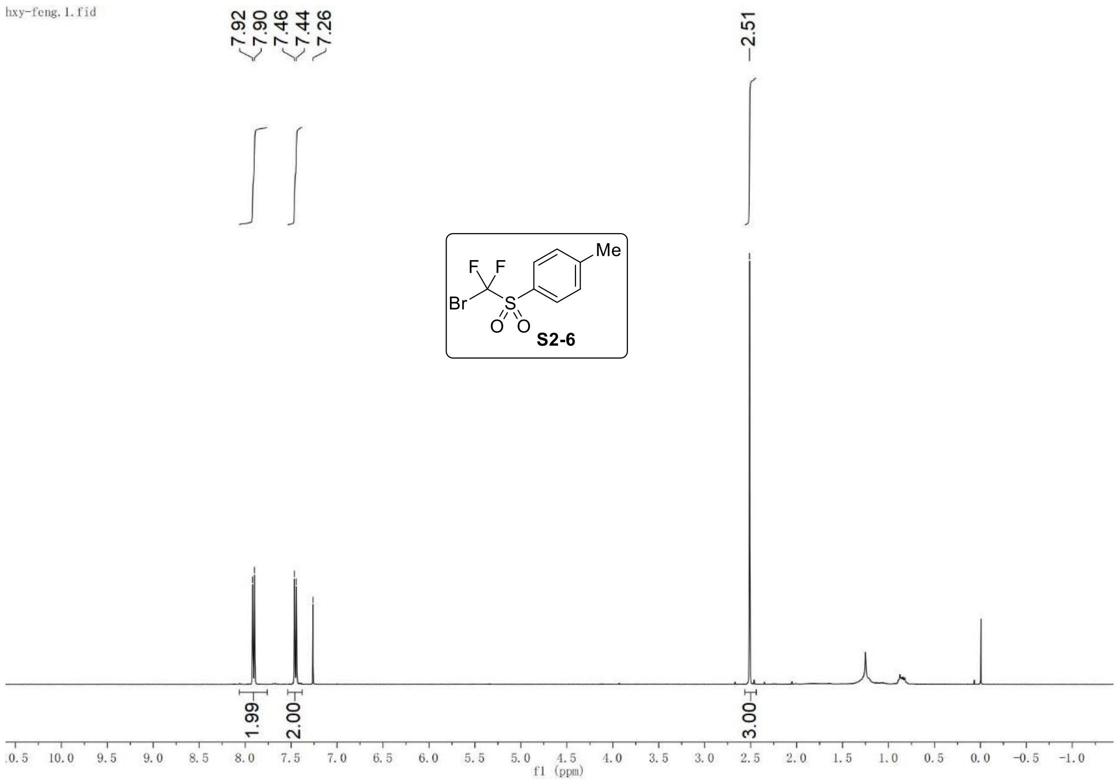

Supplementary Figure 38. ^{1}H NMR (400 MHz, $CDCl_3$) spectra of **S2-6**

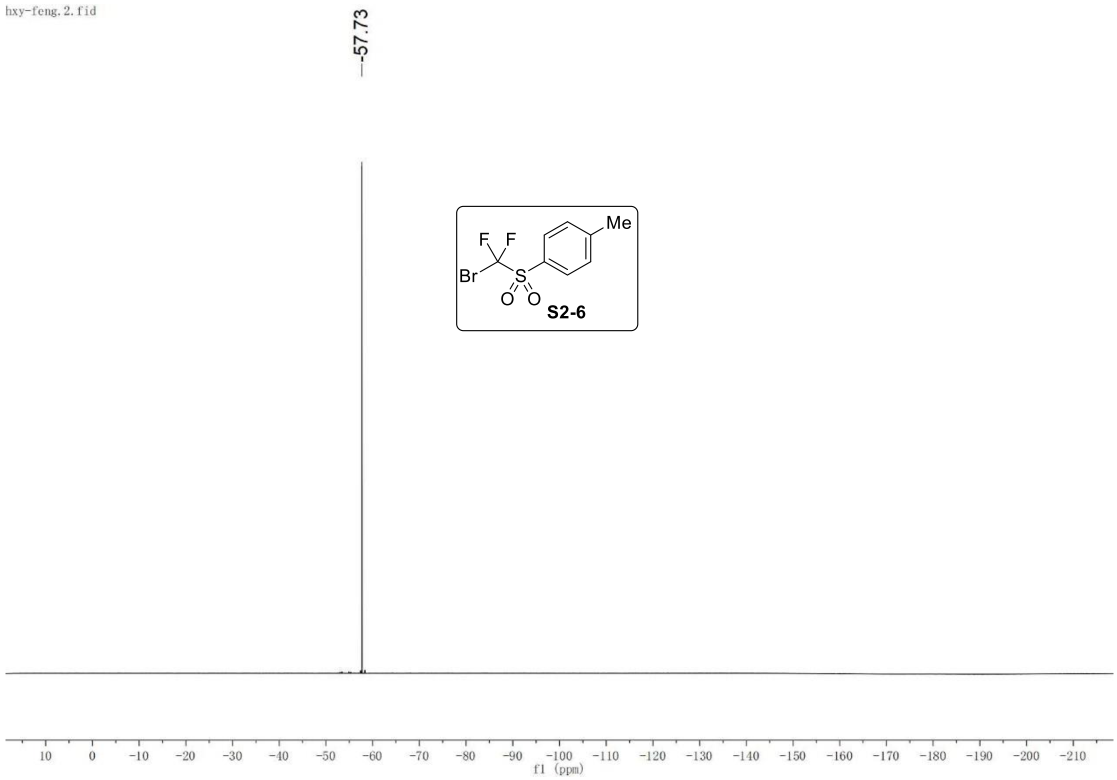

Supplementary Figure 39. ^{19}F NMR (376 MHz, $CDCl_3$) spectra of **S2-6**

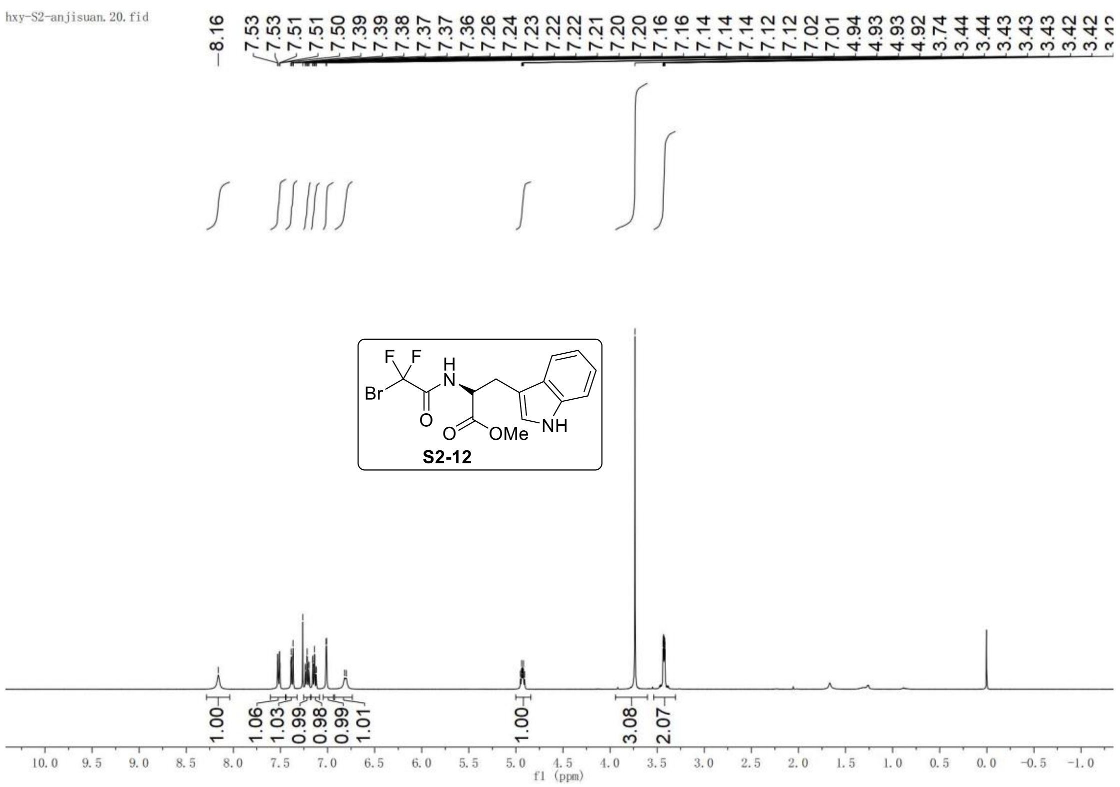

Supplementary Figure 40. ^{1}H NMR (400 MHz, $CDCl_3$) spectra of **S2-12**

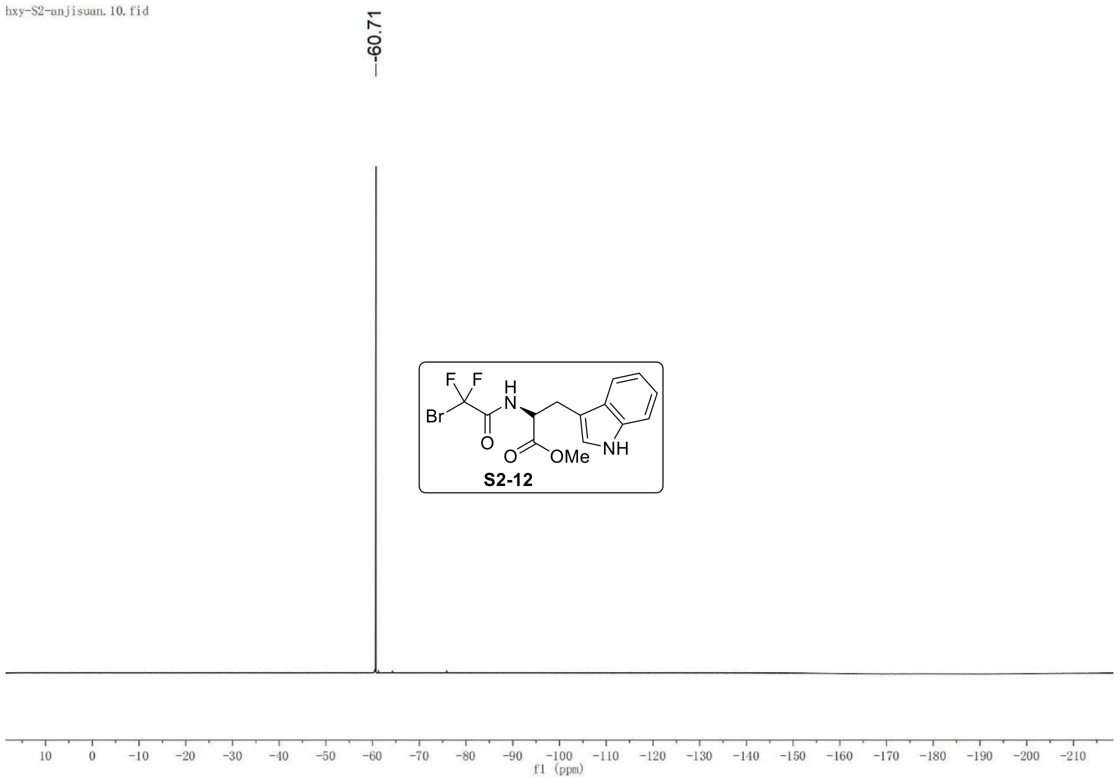

Supplementary Figure 41. ^{19}F NMR (376 MHz, $CDCl_3$) spectra of **S2-12**

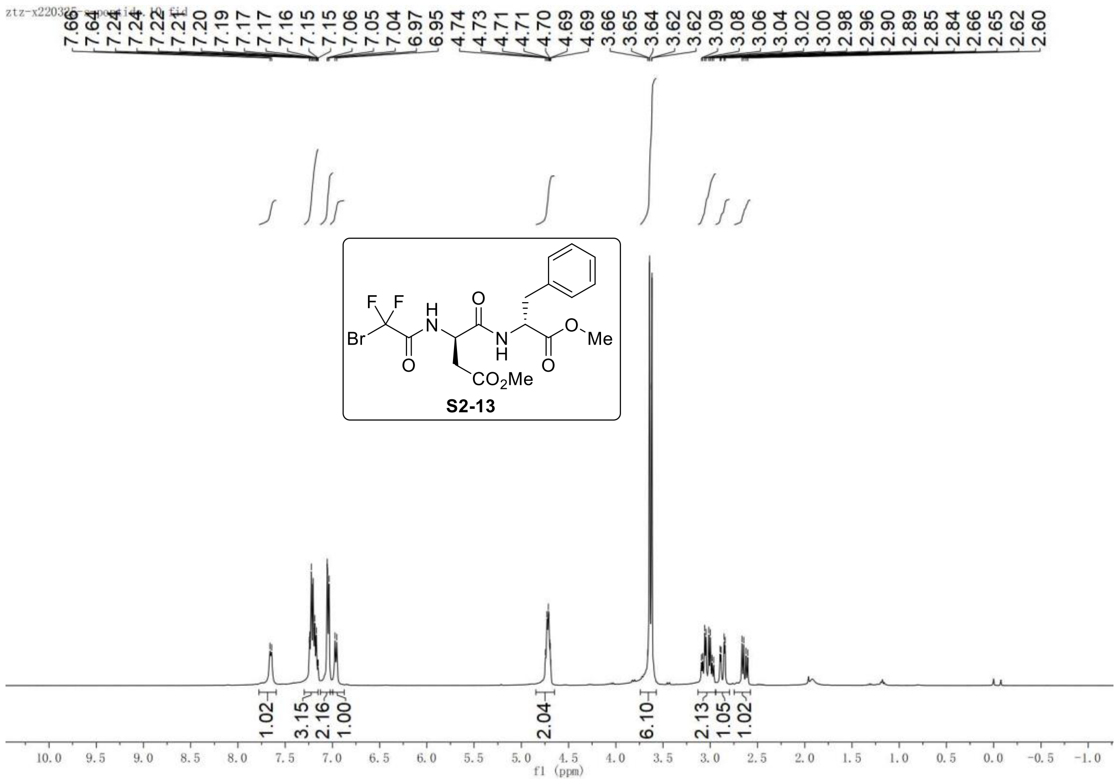

Supplementary Figure 42. ^{1}H NMR (400 MHz, $CDCl_3$) spectra of **S2-13**

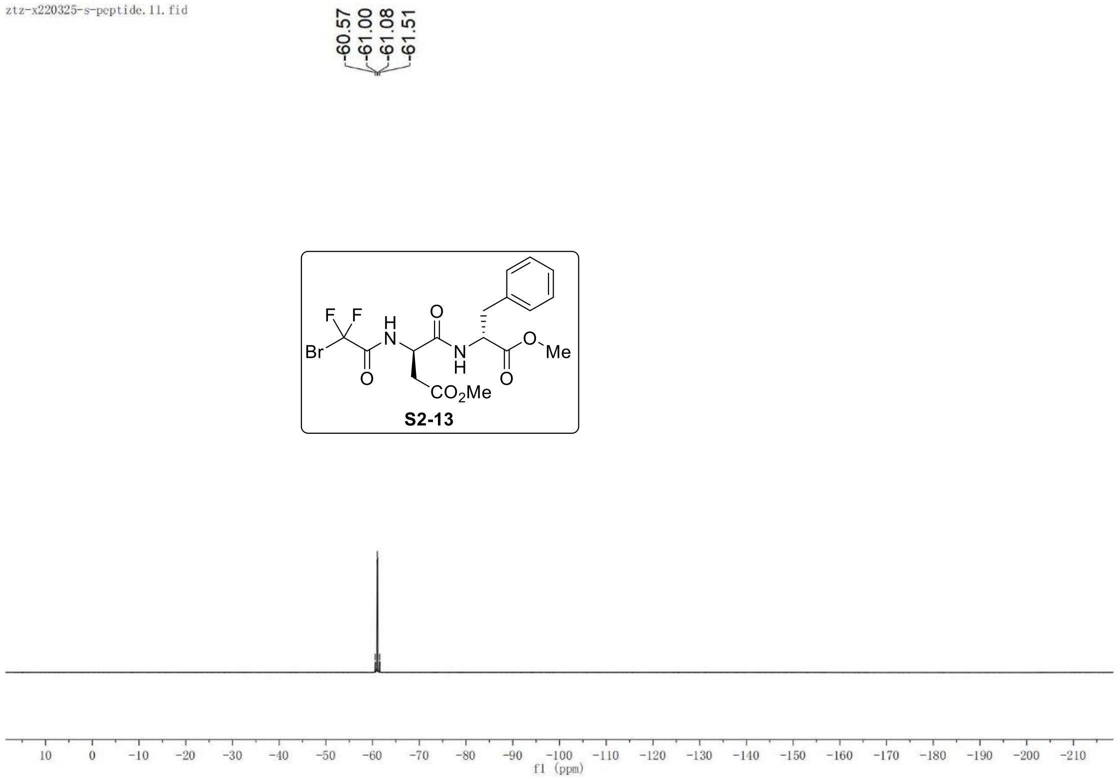

Supplementary Figure 43. ^{19}F NMR (376 MHz, $CDCl_3$) spectra of **S2-13**

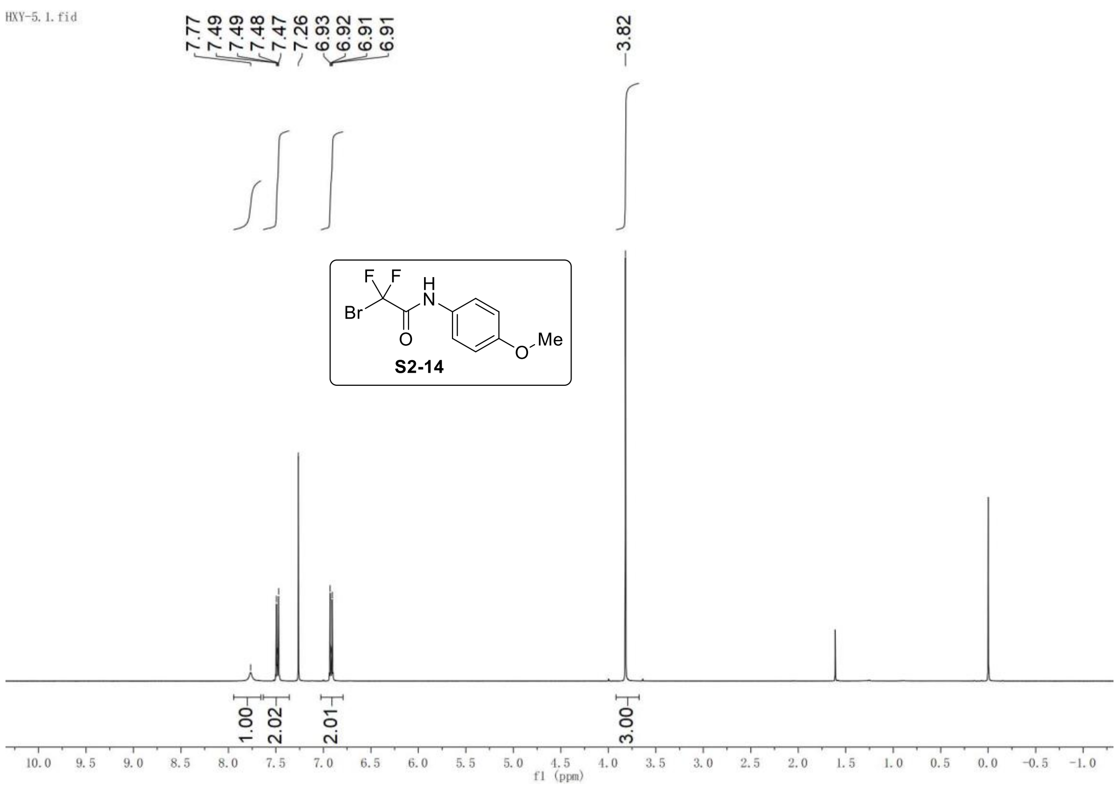

Supplementary Figure 44. ^{1}H NMR (400 MHz, $CDCl_3$) spectra of **S2-14**

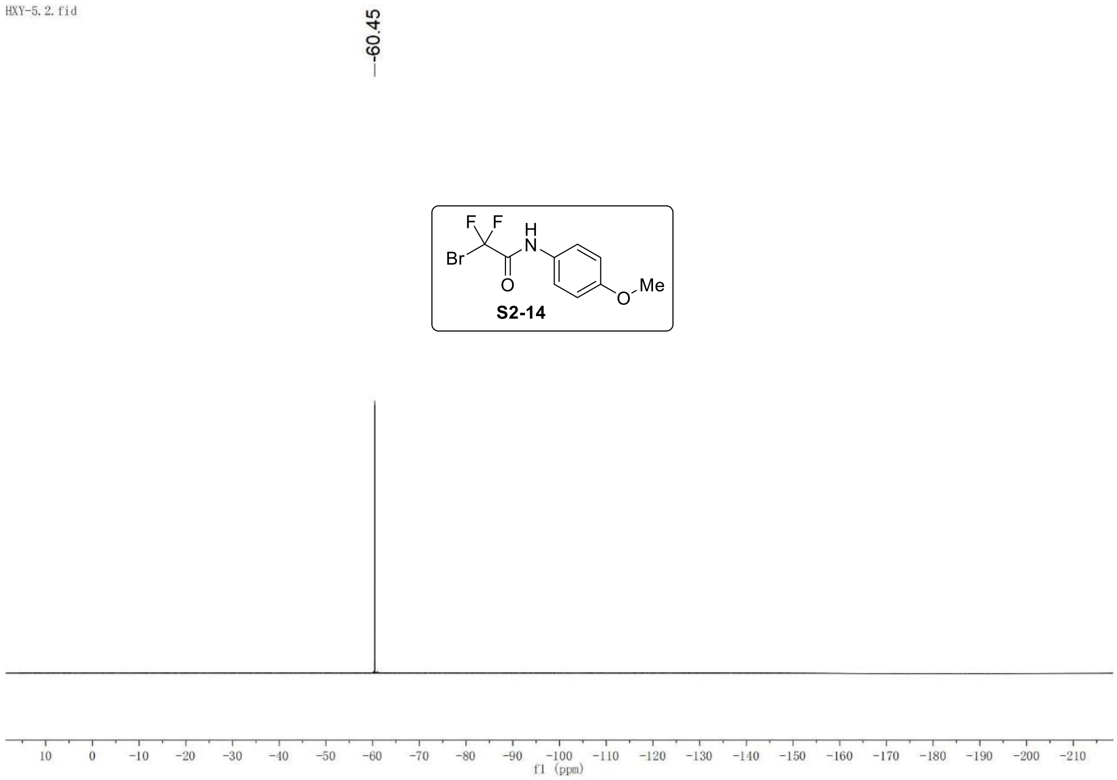

Supplementary Figure 45. ^{19}F NMR (376 MHz, $CDCl_3$) spectra of **S2-14**

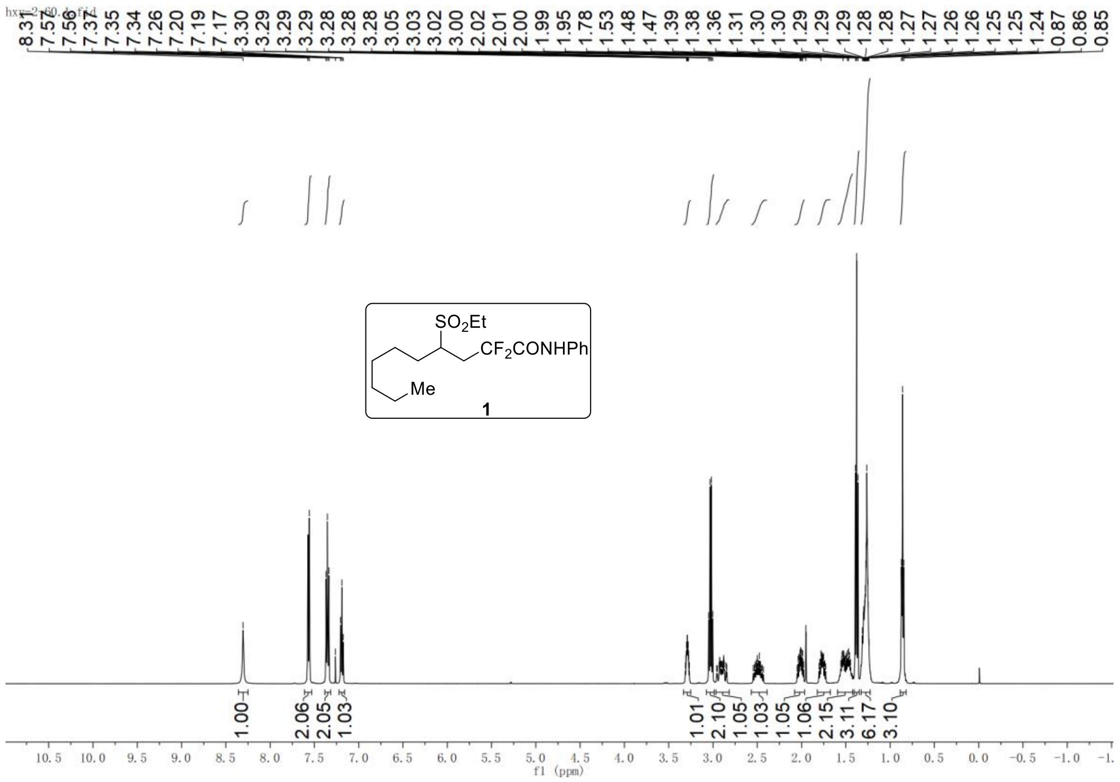

Supplementary Figure 46. ^{1}H NMR (500 MHz, $CDCl_3$) spectra of **1**

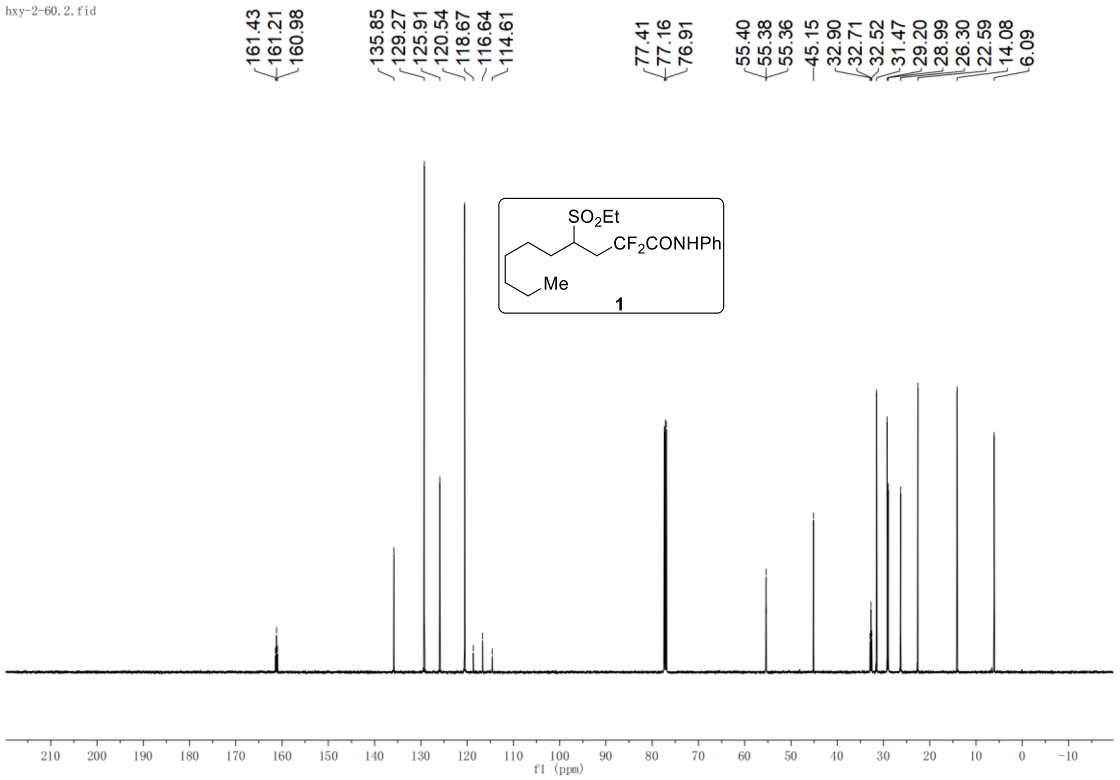

Supplementary Figure 47. ^{13}C NMR (126 MHz, $CDCl_3$) spectra of **1**

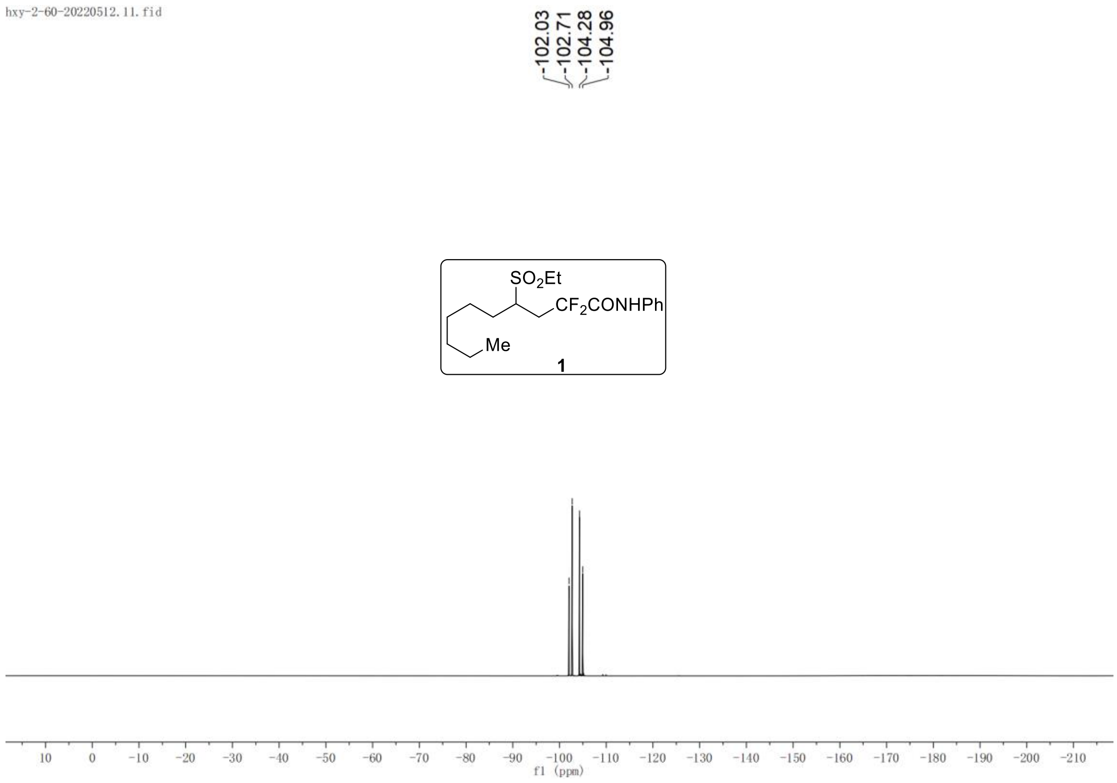

Supplementary Figure 48. ^{19}F NMR (376 MHz, $CDCl_3$) spectra of **1**

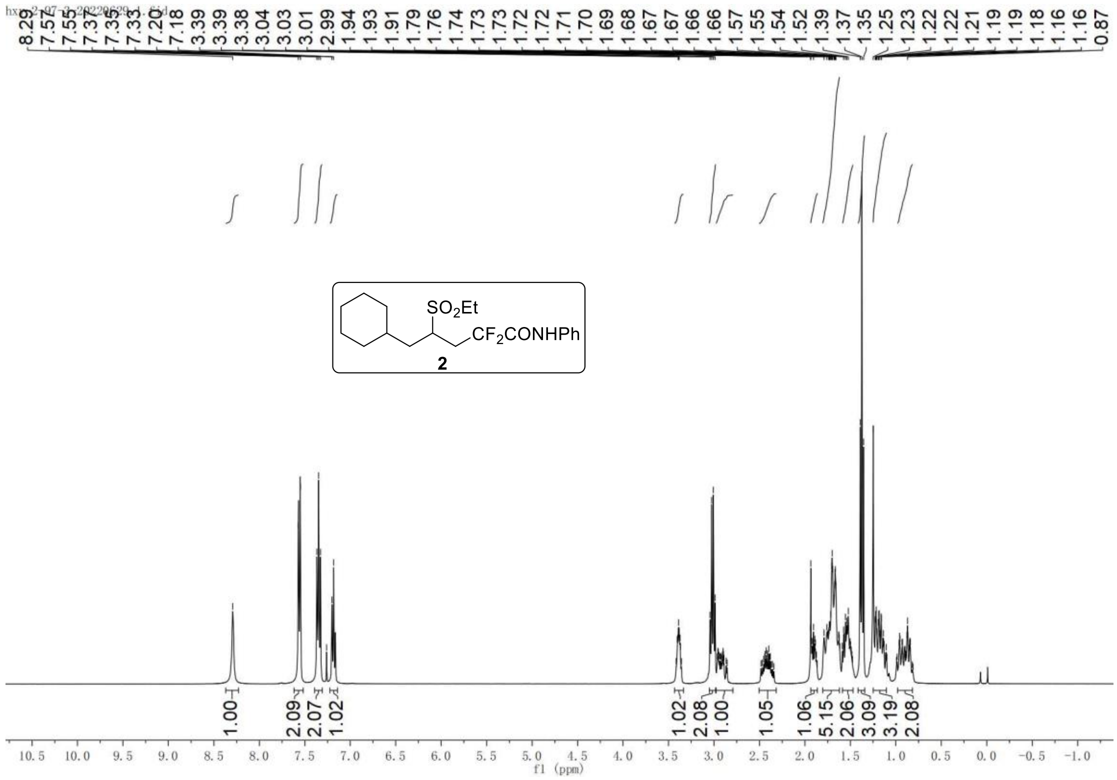

Supplementary Figure 49. ^{1}H NMR (400 MHz, $CDCl_3$) spectra of **2**

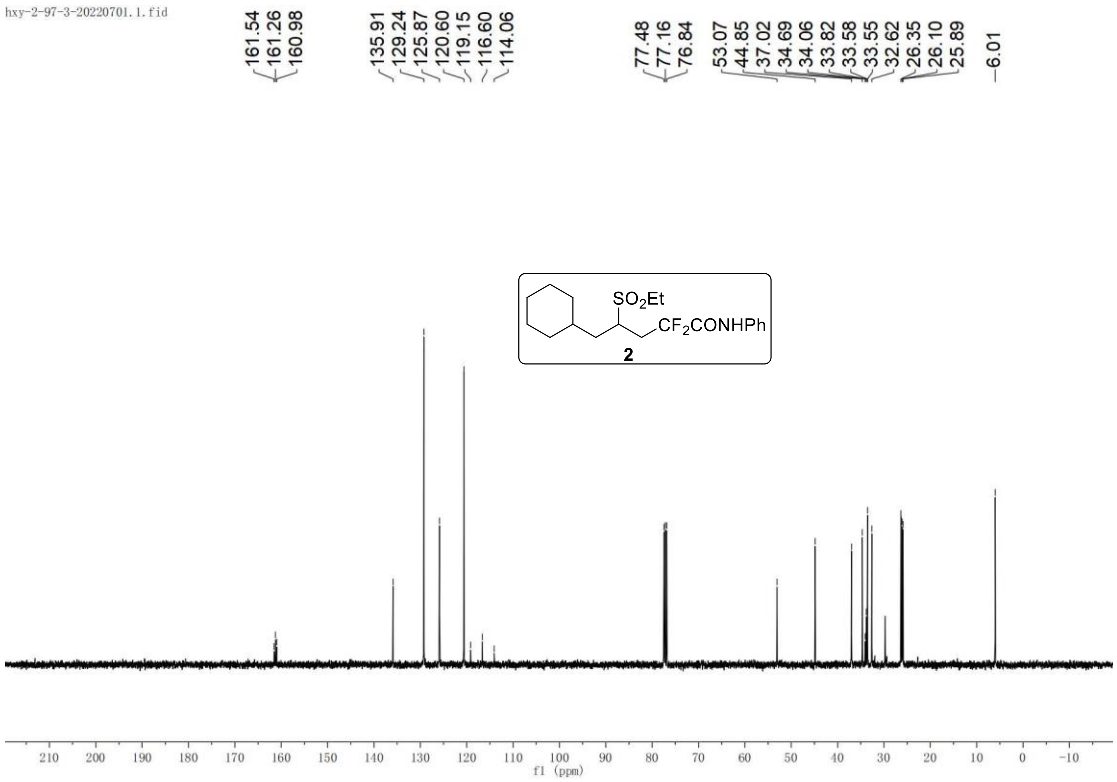

Supplementary Figure 50. ^{13}C NMR (101 MHz, $CDCl_3$) spectra of **2**

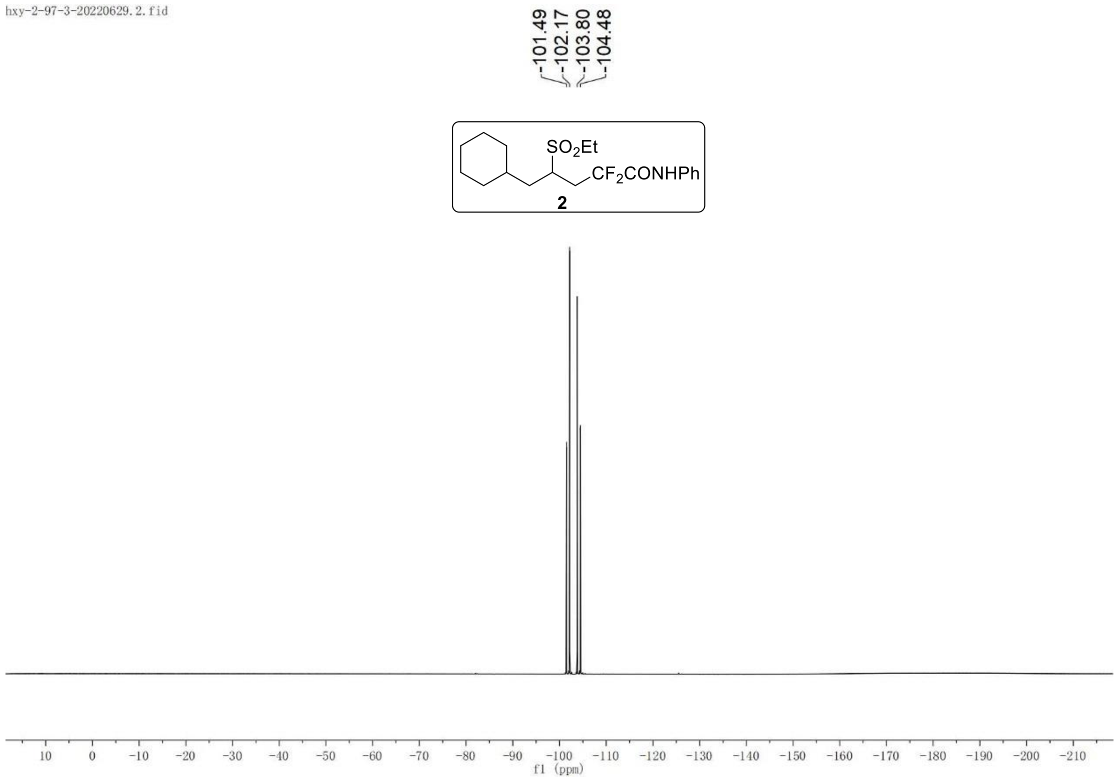

Supplementary Figure 51. ^{19}F NMR (376 MHz, $CDCl_3$) spectra of **2**

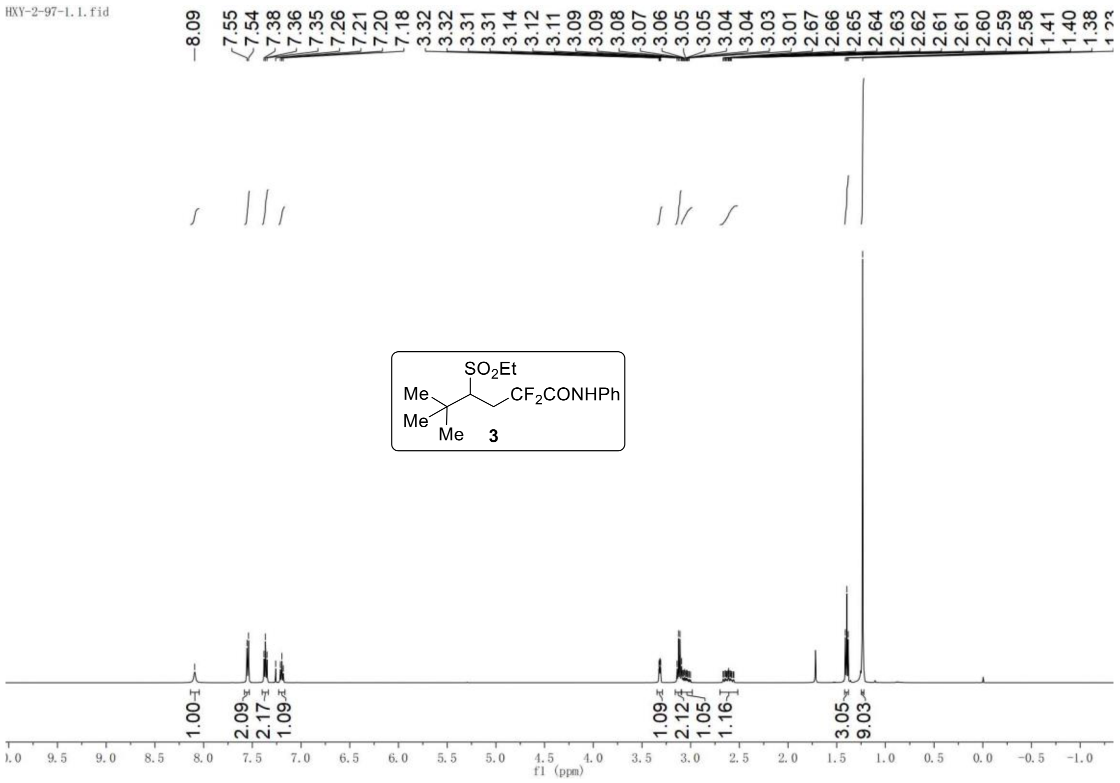

Supplementary Figure 52. ^{1}H NMR (500 MHz, $CDCl_3$) spectra of **3**

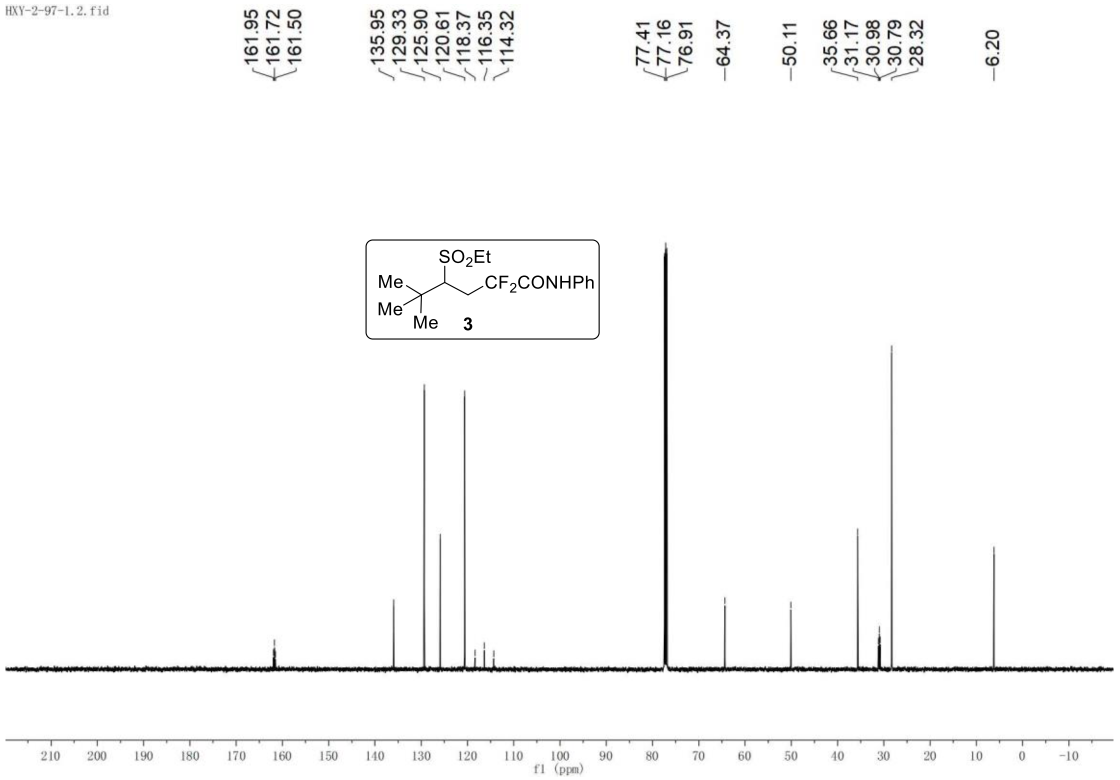

Supplementary Figure 53. ^{13}C NMR (126 MHz, $CDCl_3$) spectra of **3**

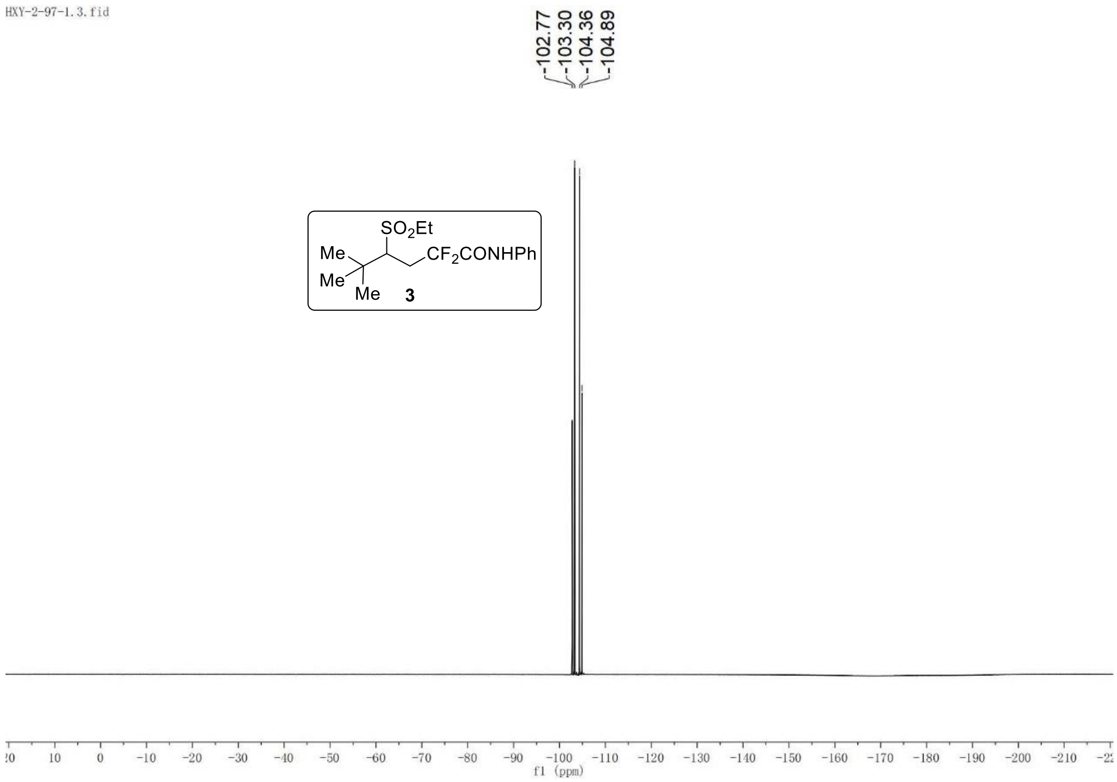

Supplementary Figure 54. ^{19}F NMR (471 MHz, $CDCl_3$) spectra of **3**

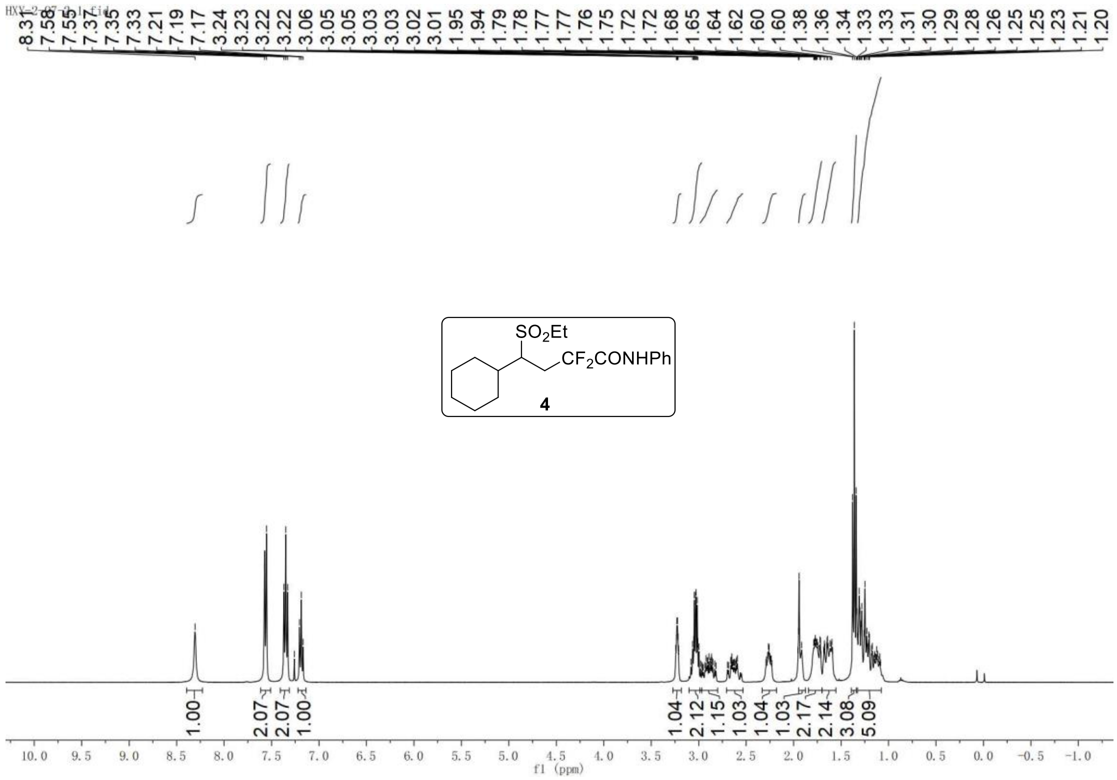

Supplementary Figure 55. ^{1}H NMR (400 MHz, $CDCl_3$) spectra of **4**

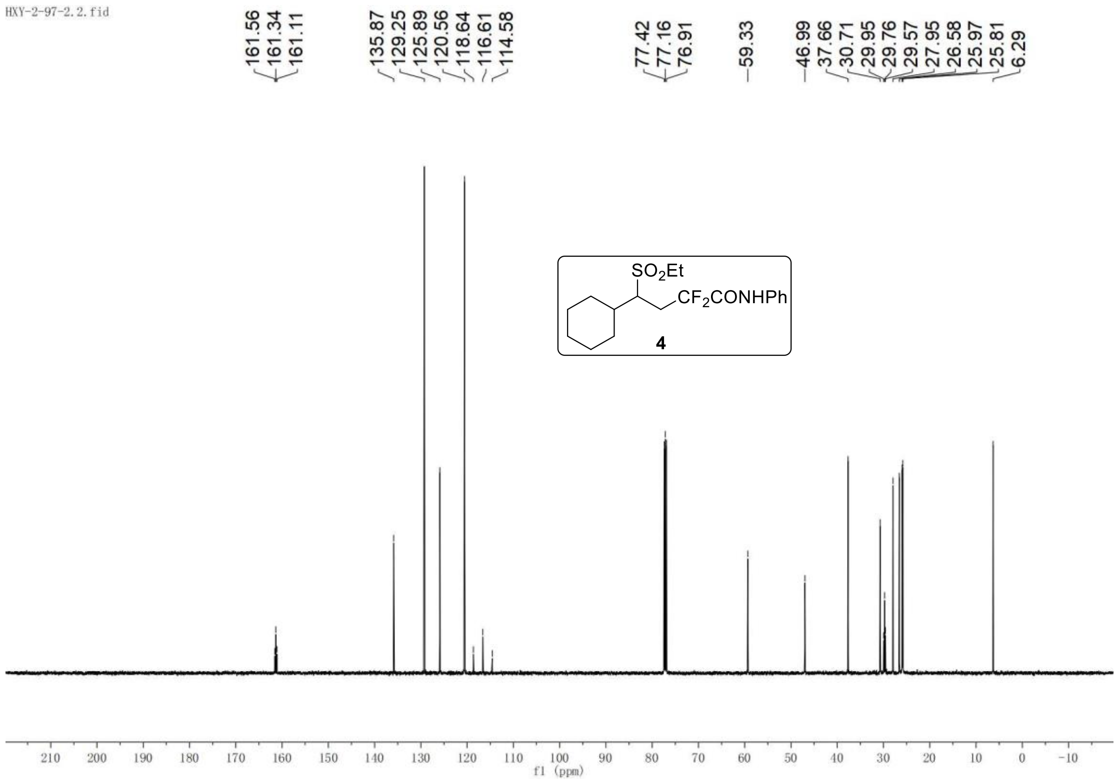

Supplementary Figure 56. ^{13}C NMR (126 MHz, $CDCl_3$) spectra of **4**

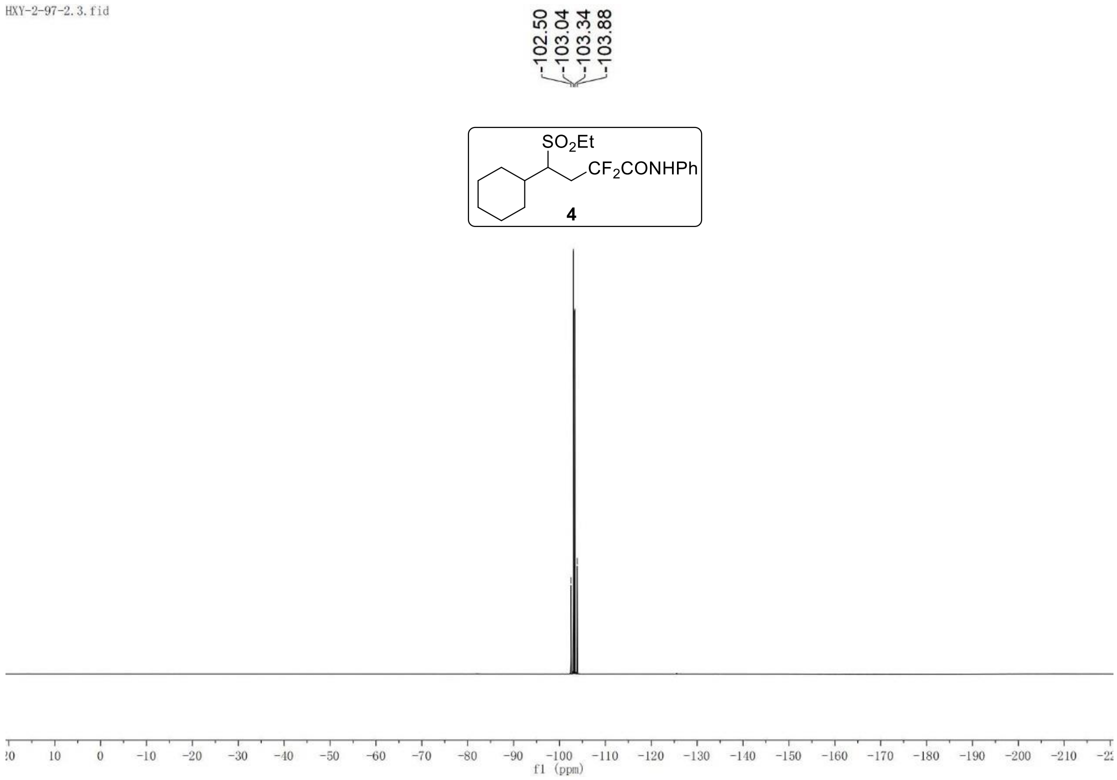

Supplementary Figure 57. ^{19}F NMR (471 MHz, $CDCl_3$) spectra of **4**

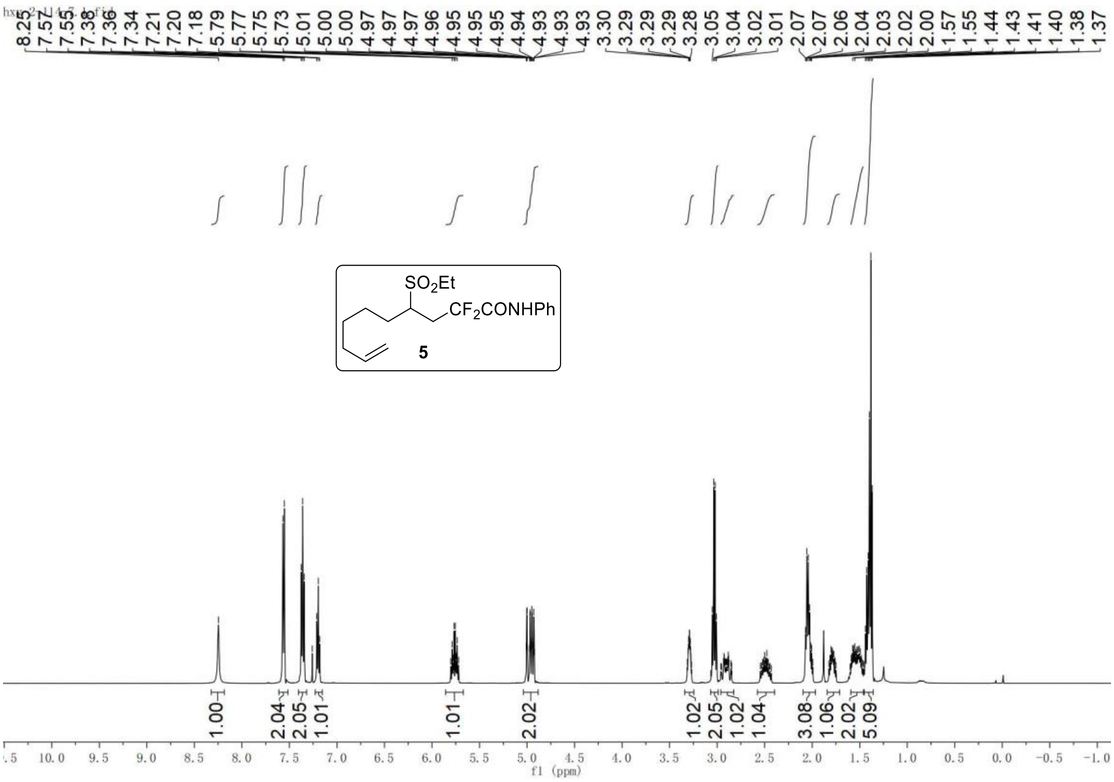

Supplementary Figure 58. ^{1}H NMR (500 MHz, $CDCl_3$) spectra of **5**

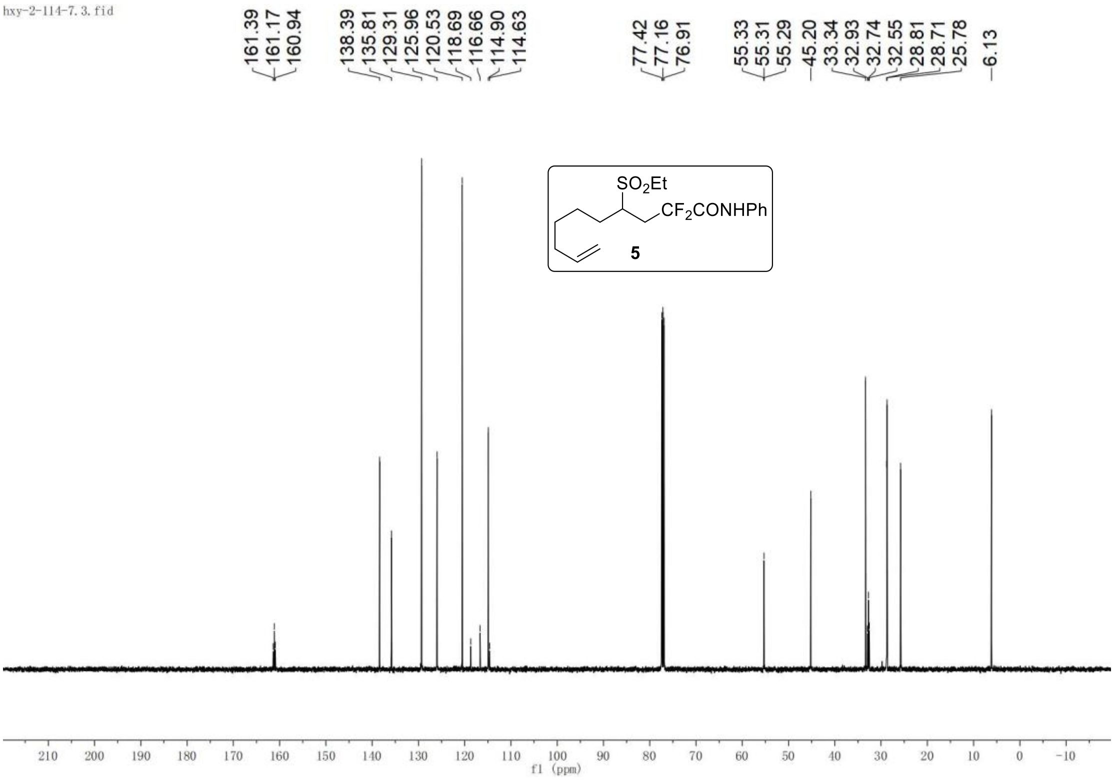

Supplementary Figure 59. ^{13}C NMR (126 MHz, $CDCl_3$) spectra of **5**

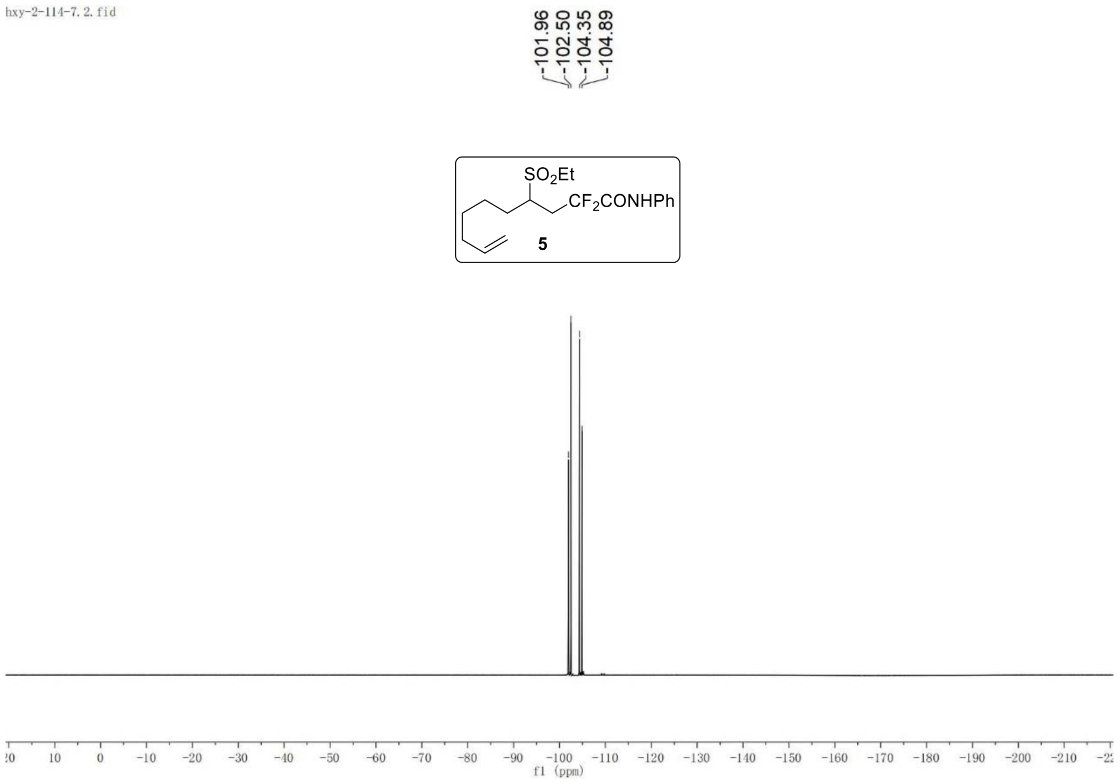

Supplementary Figure 60. ^{19}F NMR (471 MHz, $CDCl_3$) spectra of **5**

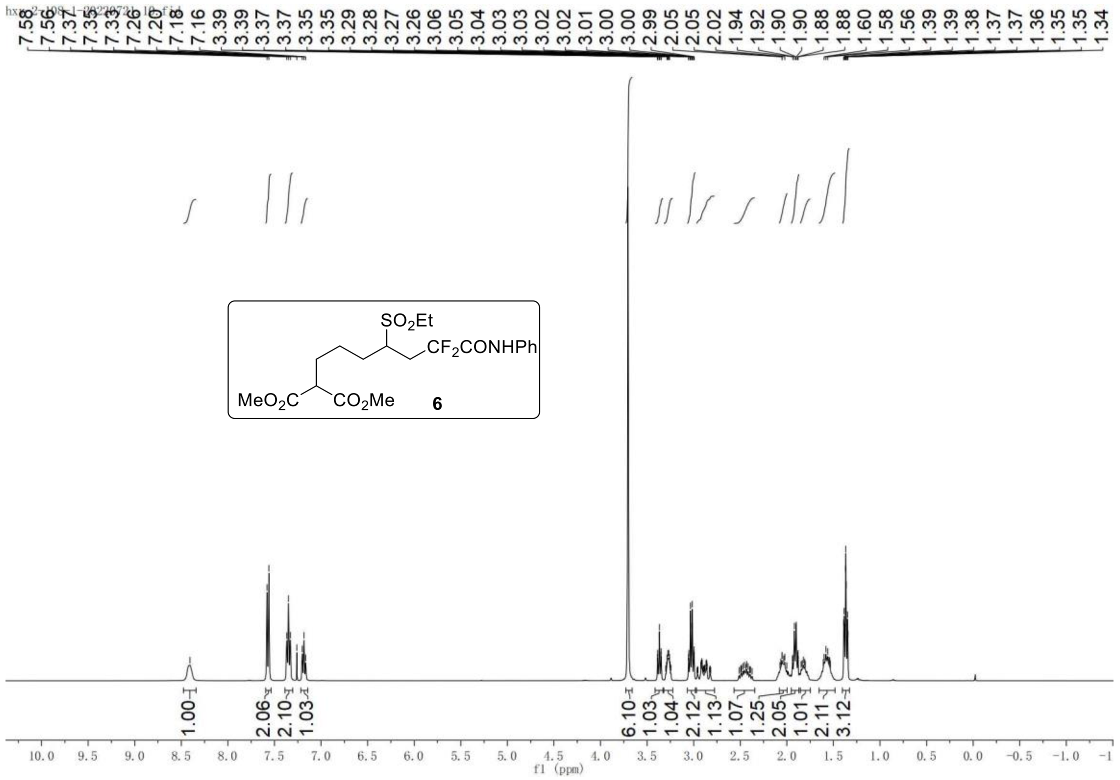

Supplementary Figure 61. ^{1}H NMR (400 MHz, $CDCl_3$) spectra of **6**

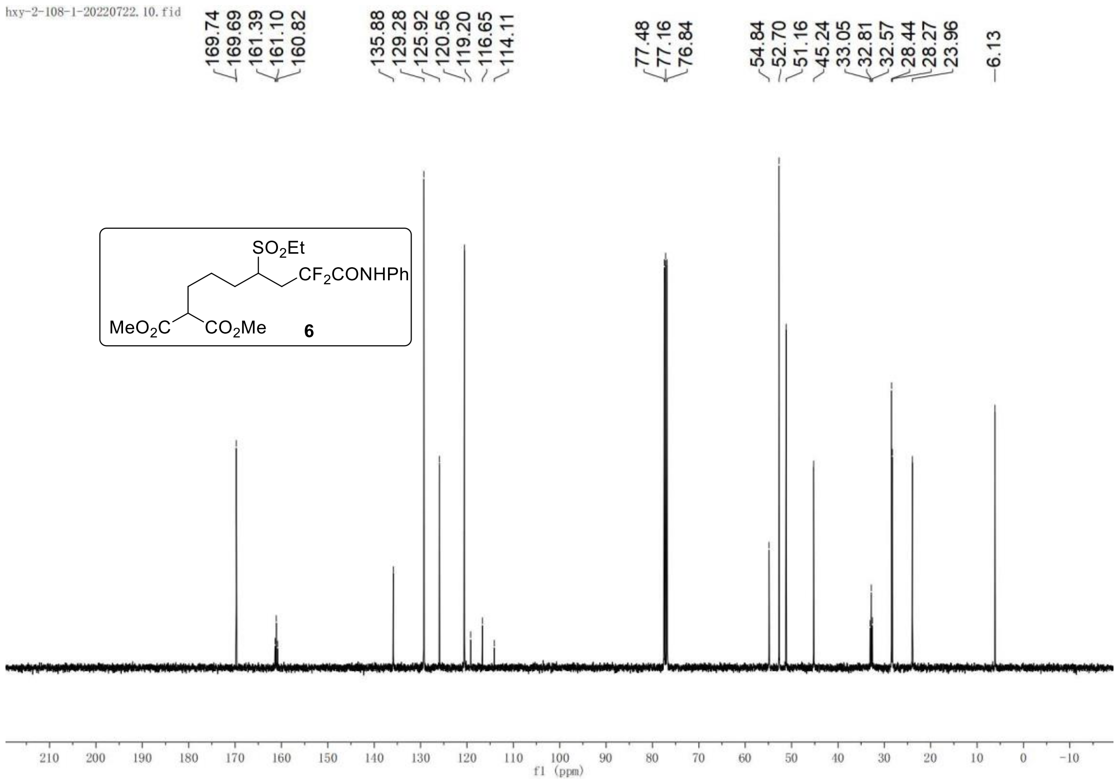

Supplementary Figure 62. ^{13}C NMR (101 MHz, $CDCl_3$) spectra of **6**

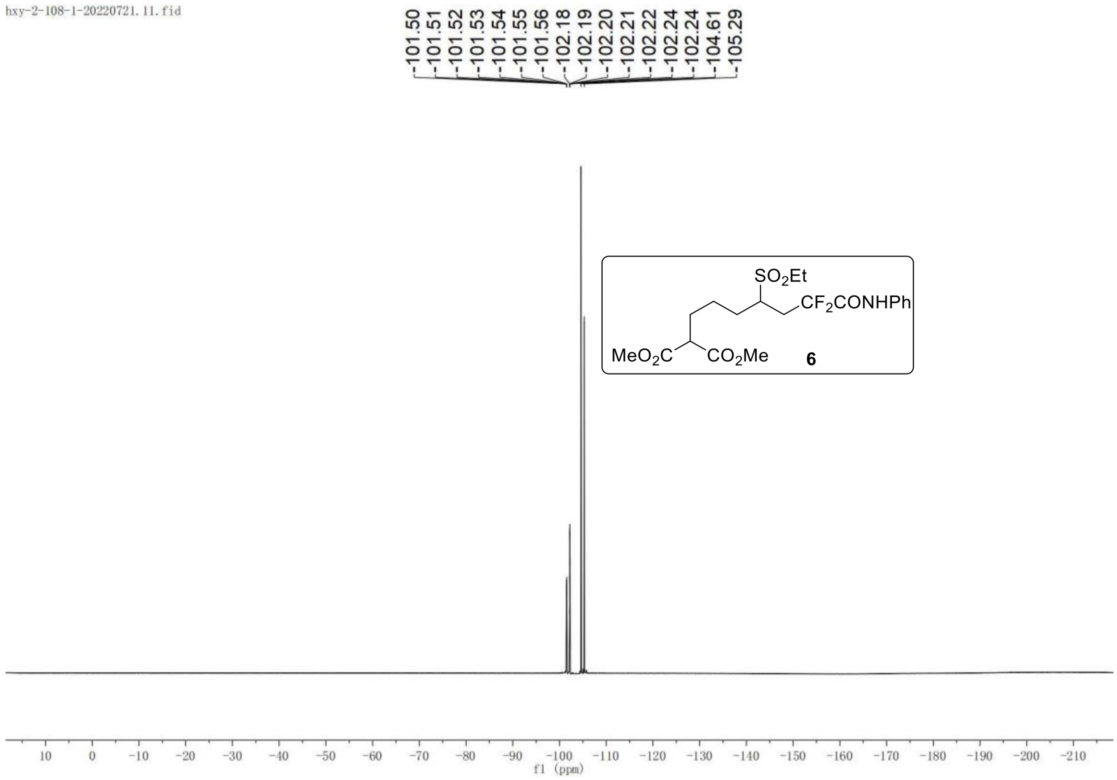

Supplementary Figure 63. ^{19}F NMR (376 MHz, $CDCl_3$) spectra of **6**

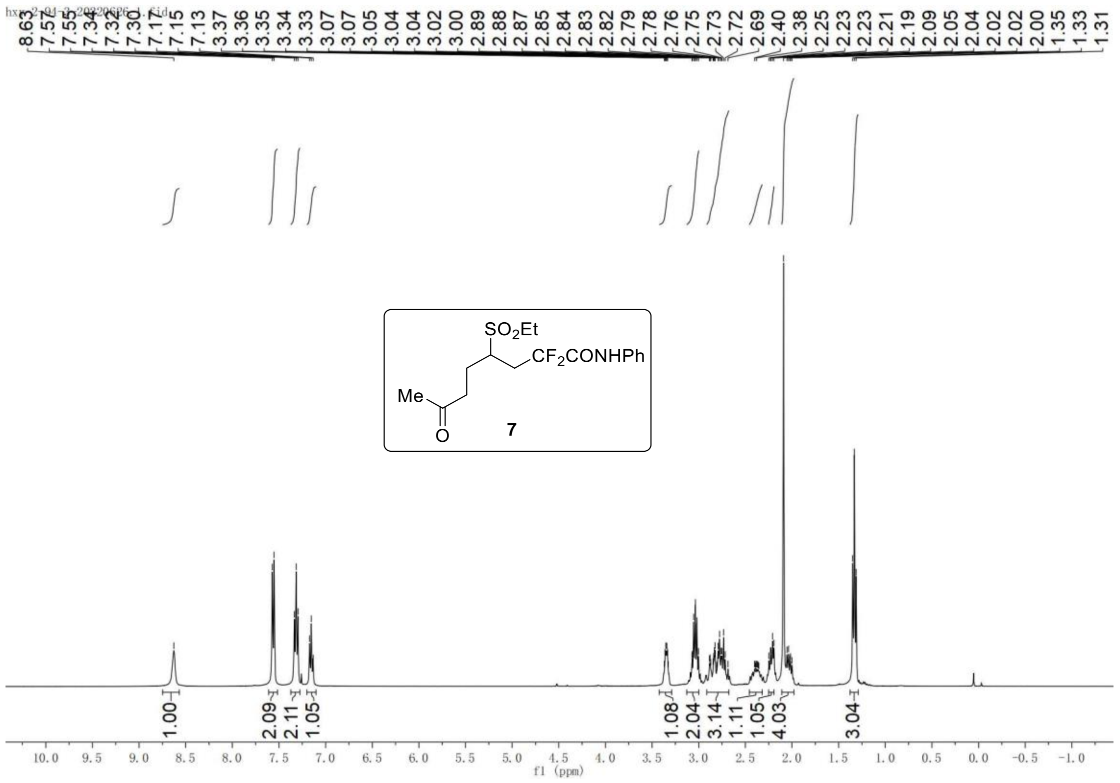

Supplementary Figure 64. ^{1}H NMR (400 MHz, $CDCl_3$) spectra of **7**

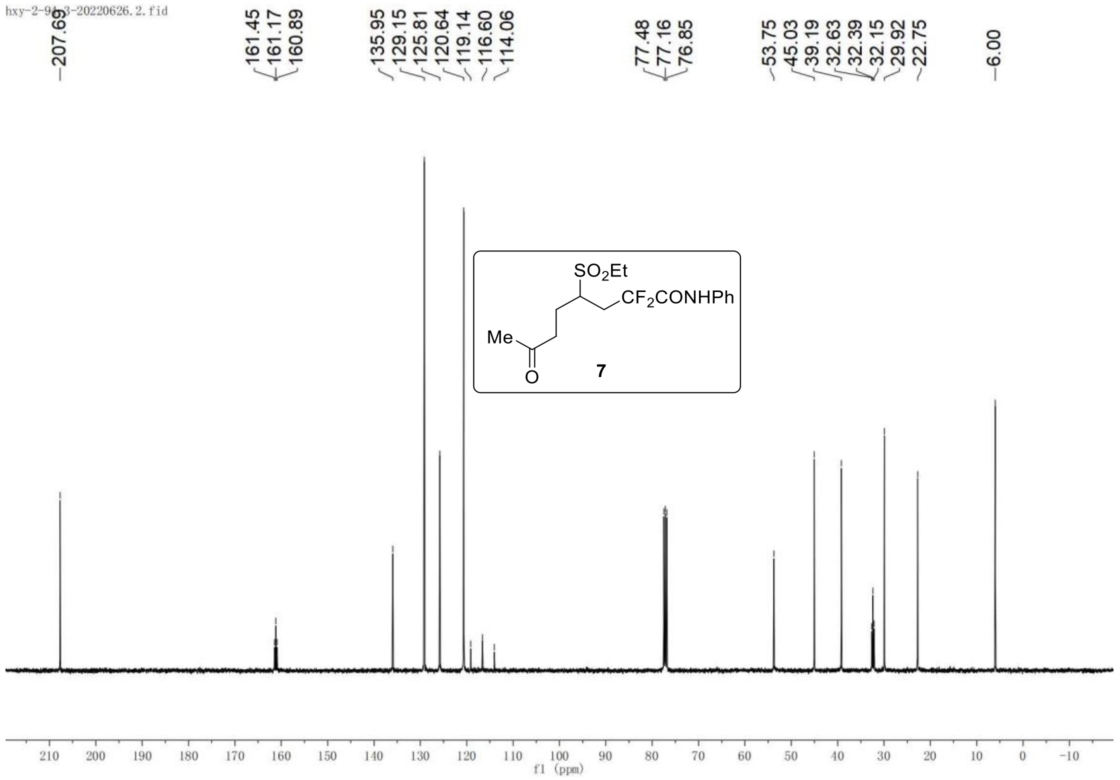

Supplementary Figure 65. ^{13}C NMR (101 MHz, $CDCl_3$) spectra of **7**

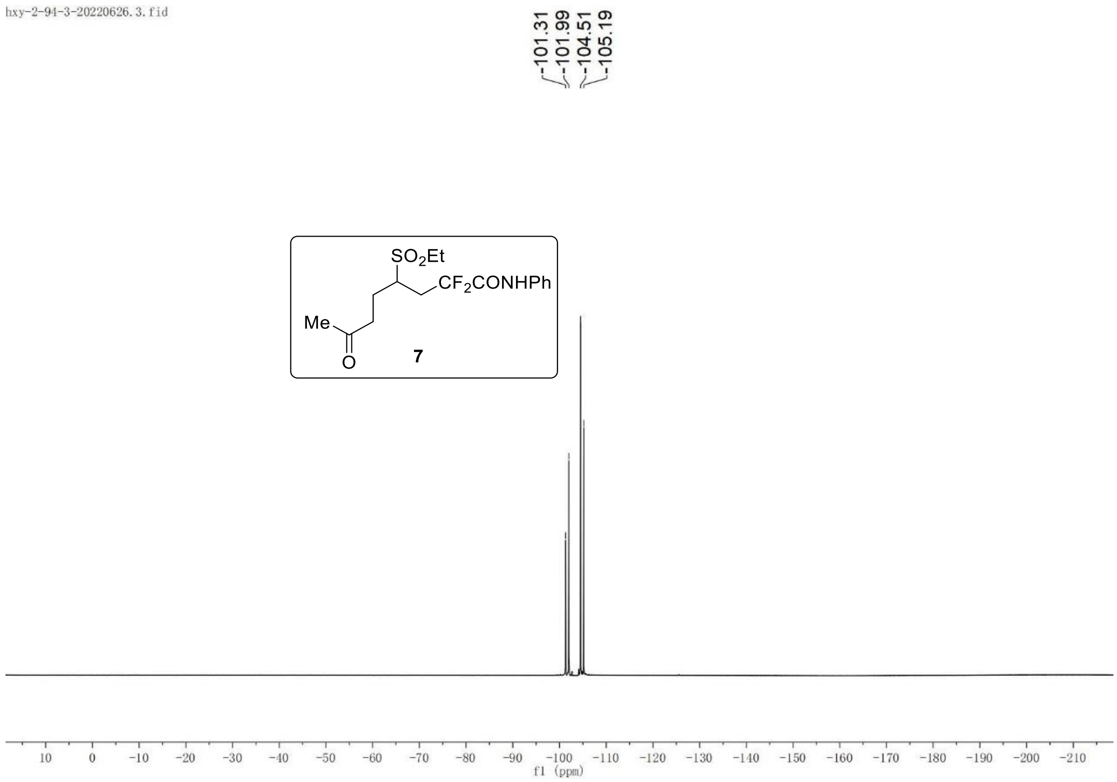

Supplementary Figure 66. ^{19}F NMR (376 MHz, $CDCl_3$) spectra of **7**

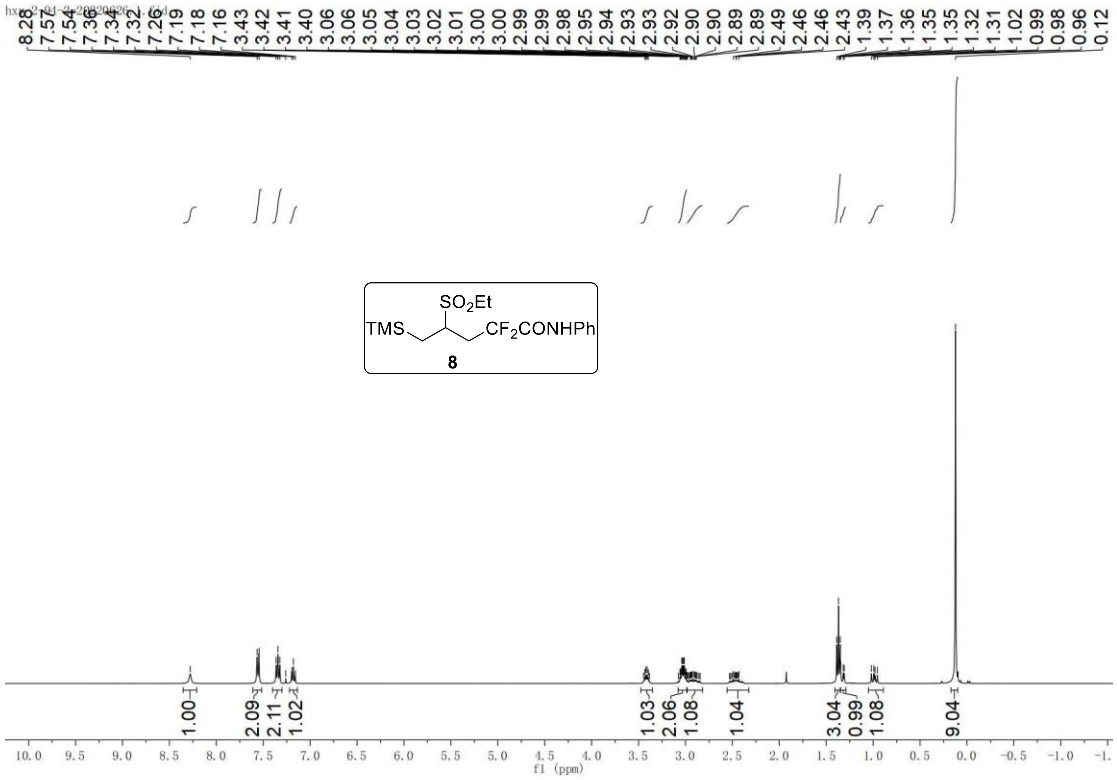

Supplementary Figure 67. ^{1}H NMR (400 MHz, $CDCl_3$) spectra of **8**

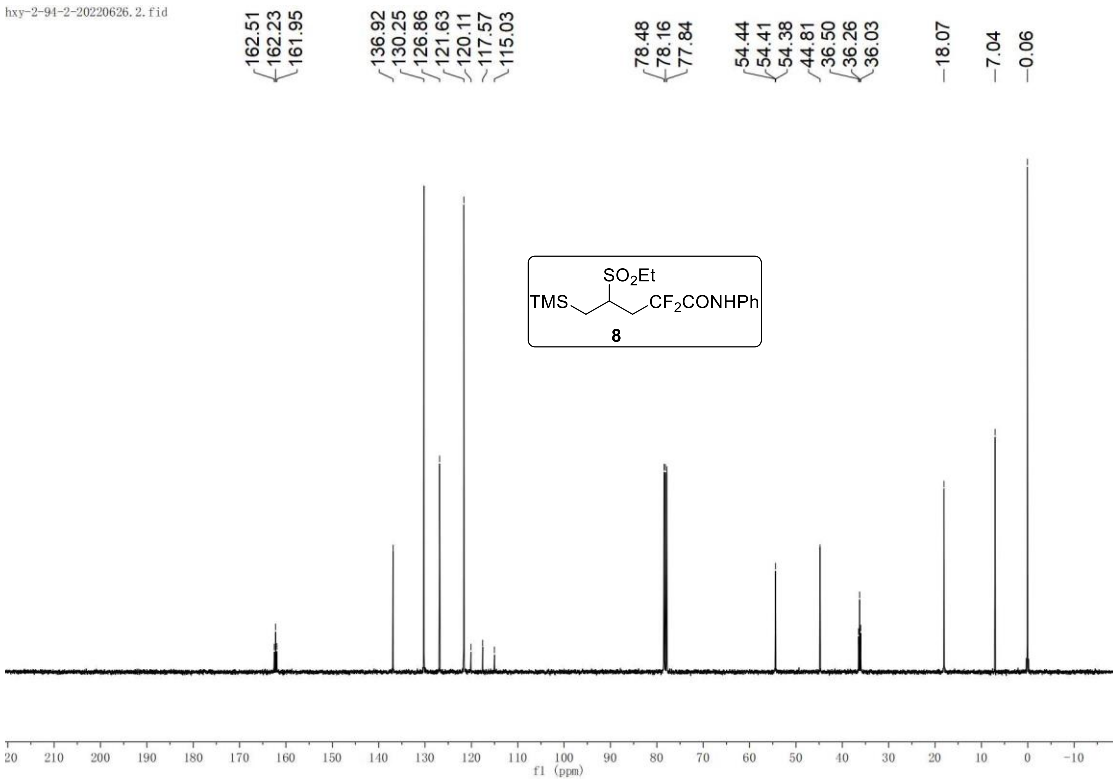

Supplementary Figure 68. ^{13}C NMR (101 MHz, $CDCl_3$) spectra of **8**

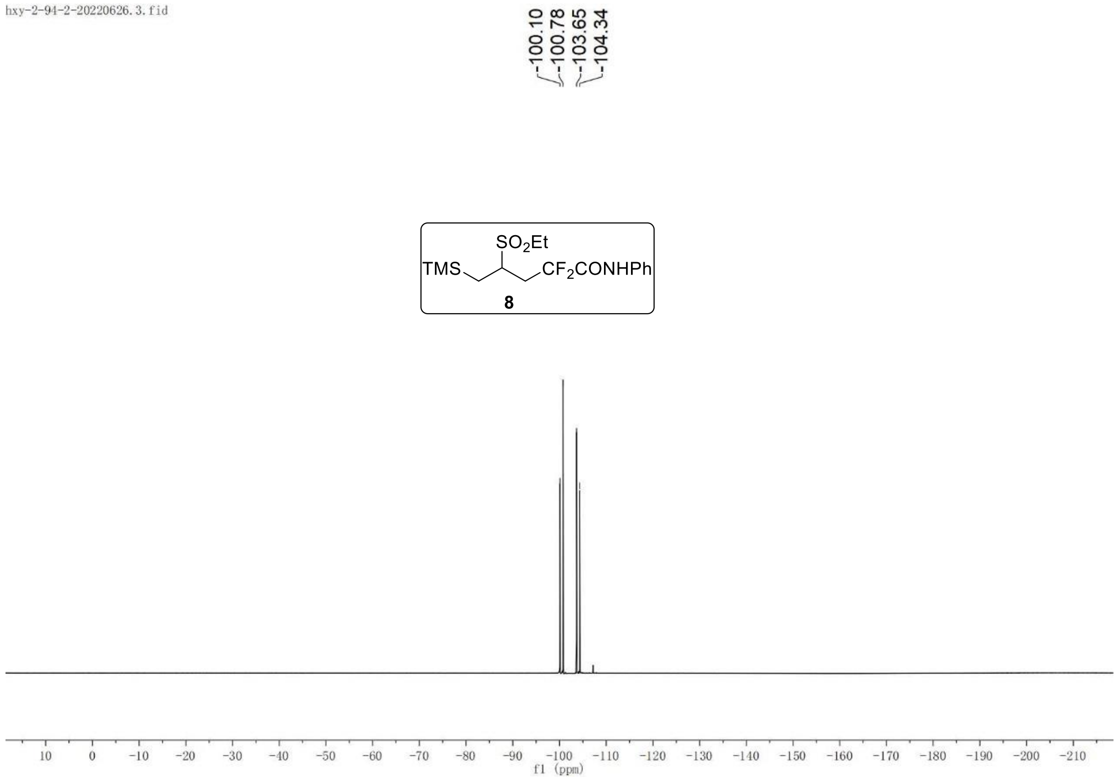

Supplementary Figure 69. ^{19}F NMR (376 MHz, $CDCl_3$) spectra of **8**

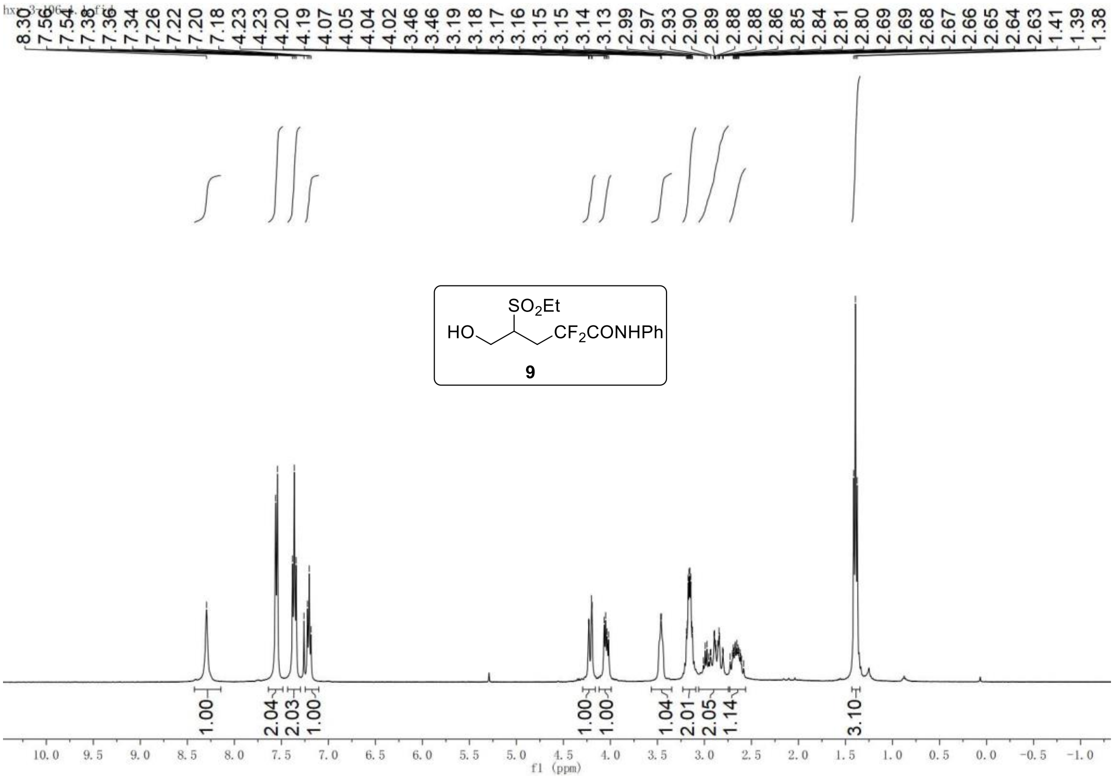

Supplementary Figure 70. ^{1}H NMR (400 MHz, $CDCl_3$) spectra of **9**

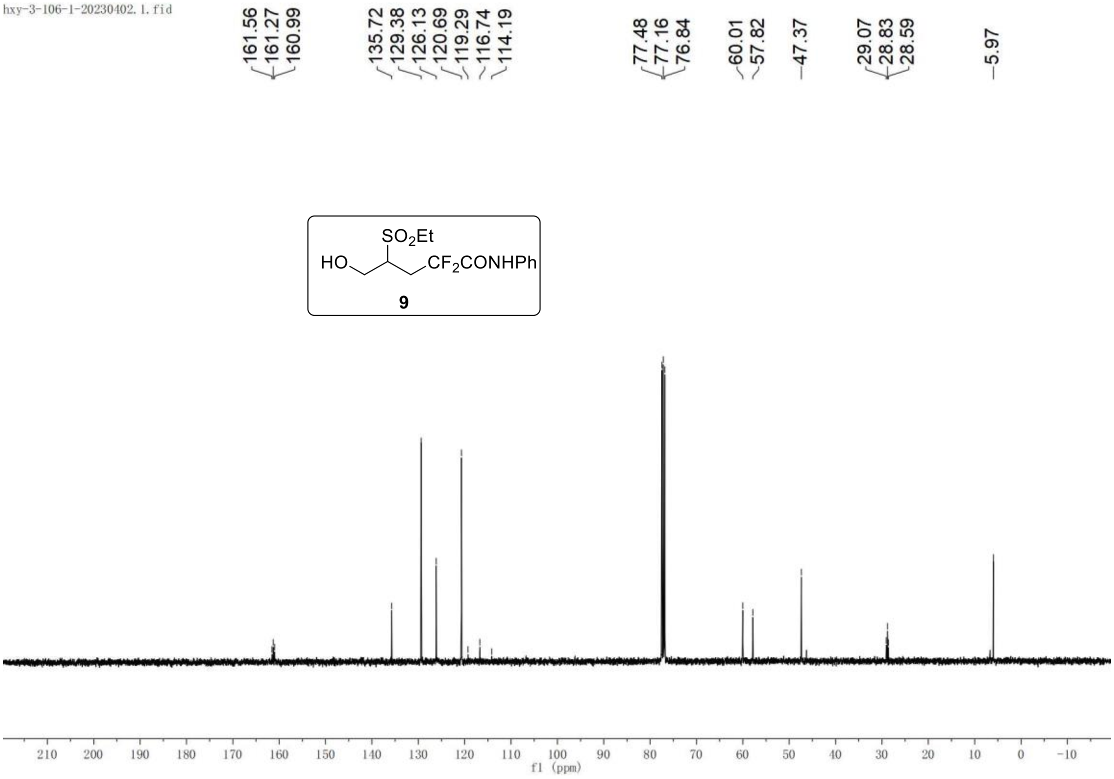

Supplementary Figure 71. ^{13}C NMR (101 MHz, $CDCl_3$) spectra of **9**

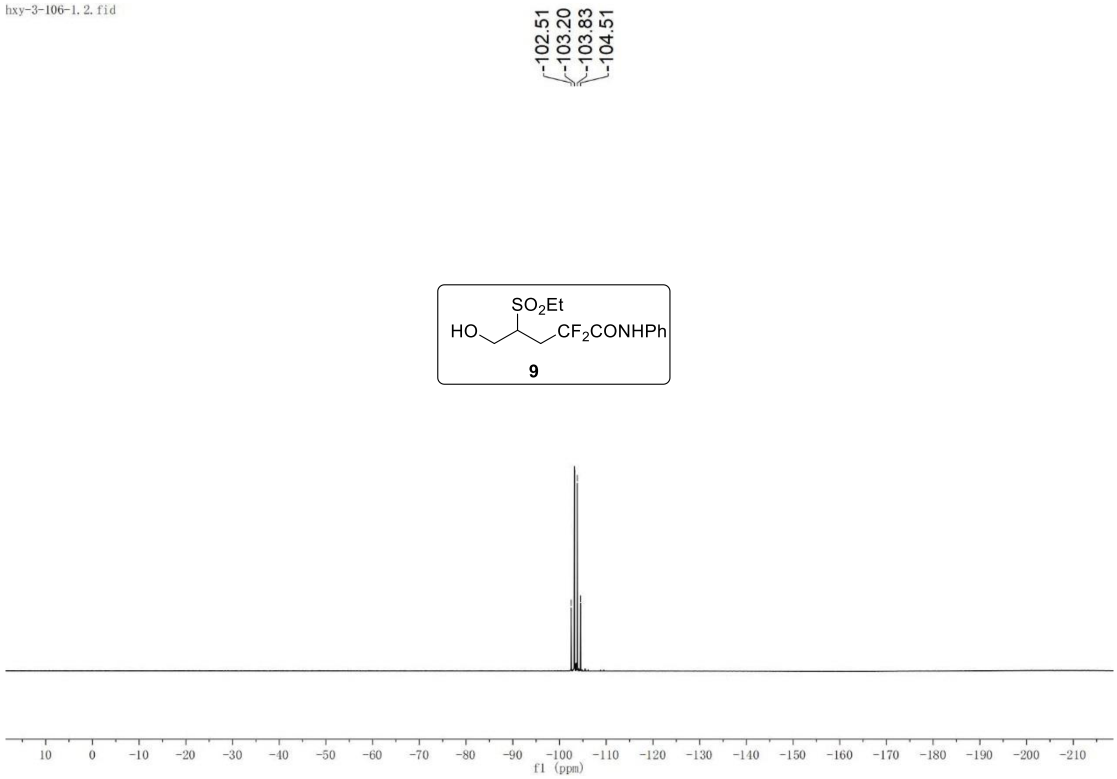

Supplementary Figure 72. ^{19}F NMR (376 MHz, $CDCl_3$) spectra of **9**

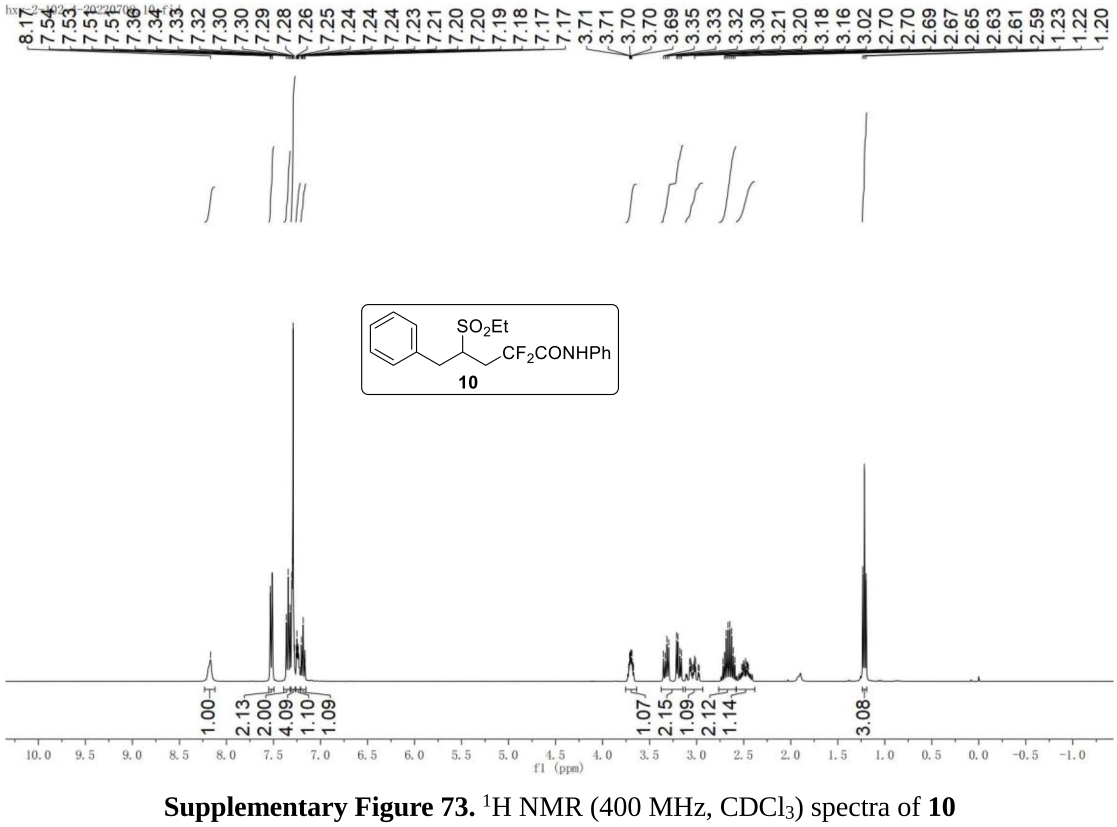

Supplementary Figure 73. ^{1}H NMR (400 MHz, $CDCl_3$) spectra of **10**

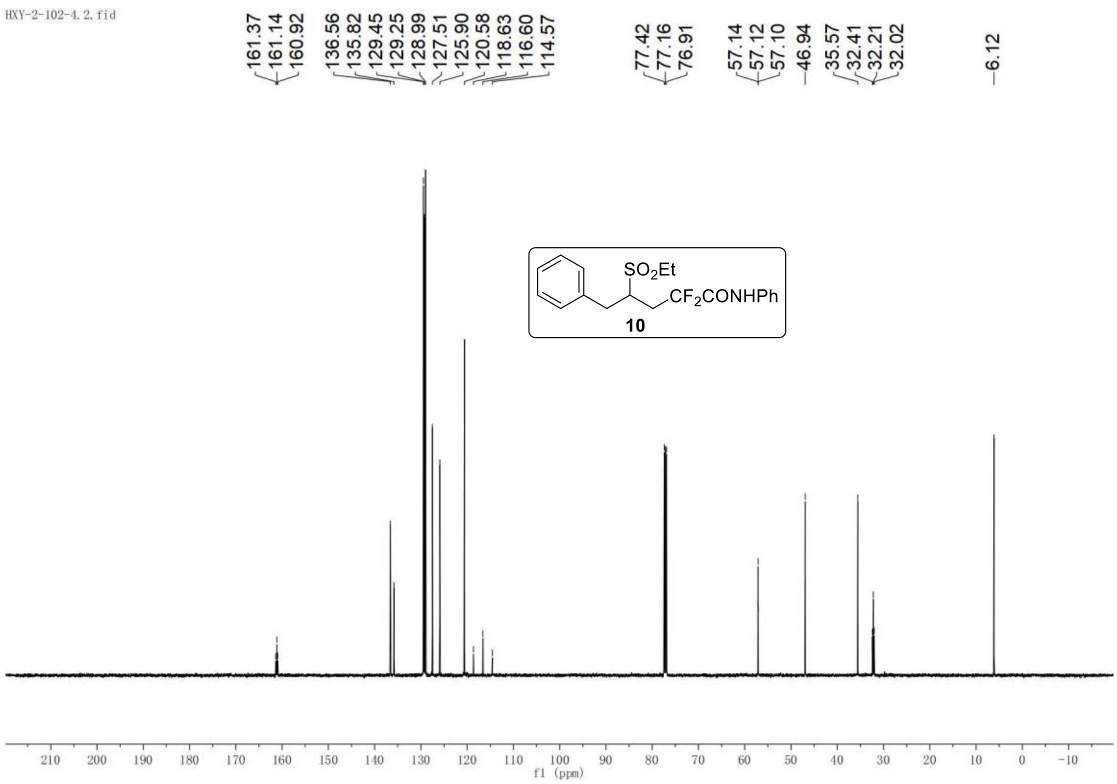

Supplementary Figure 74. ^{13}C NMR (126 MHz, $CDCl_3$) spectra of **10**

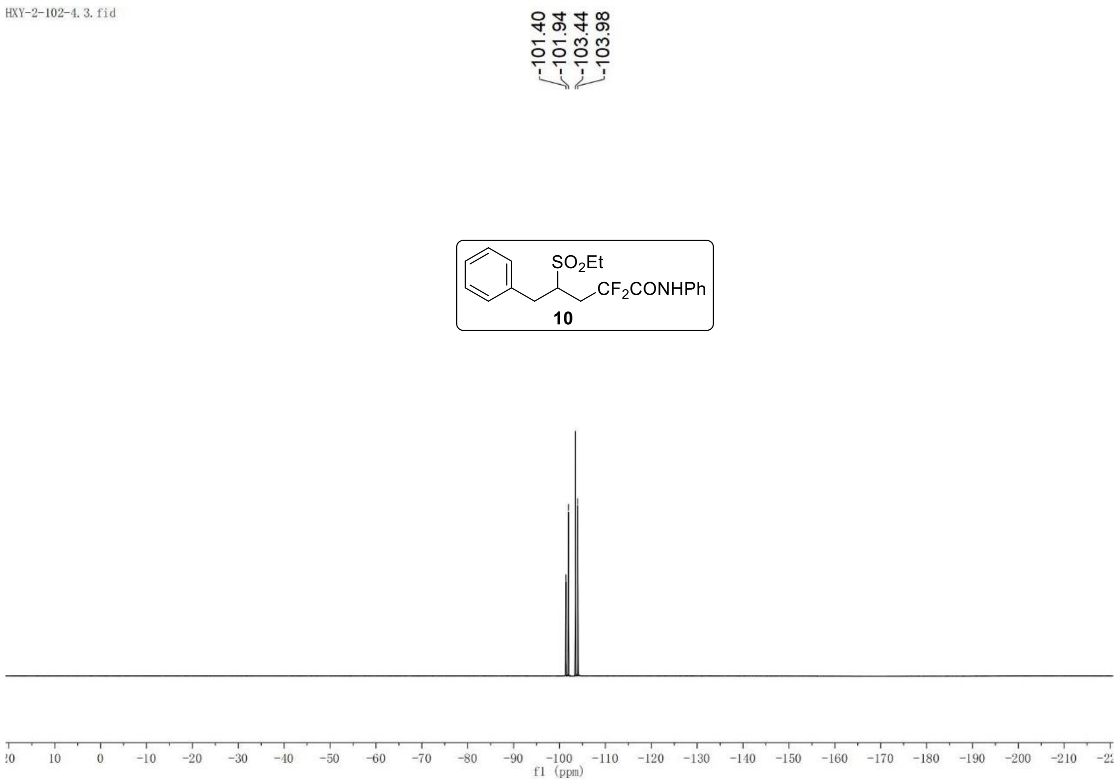

Supplementary Figure 75. ^{19}F NMR (471 MHz, $CDCl_3$) spectra of **10**

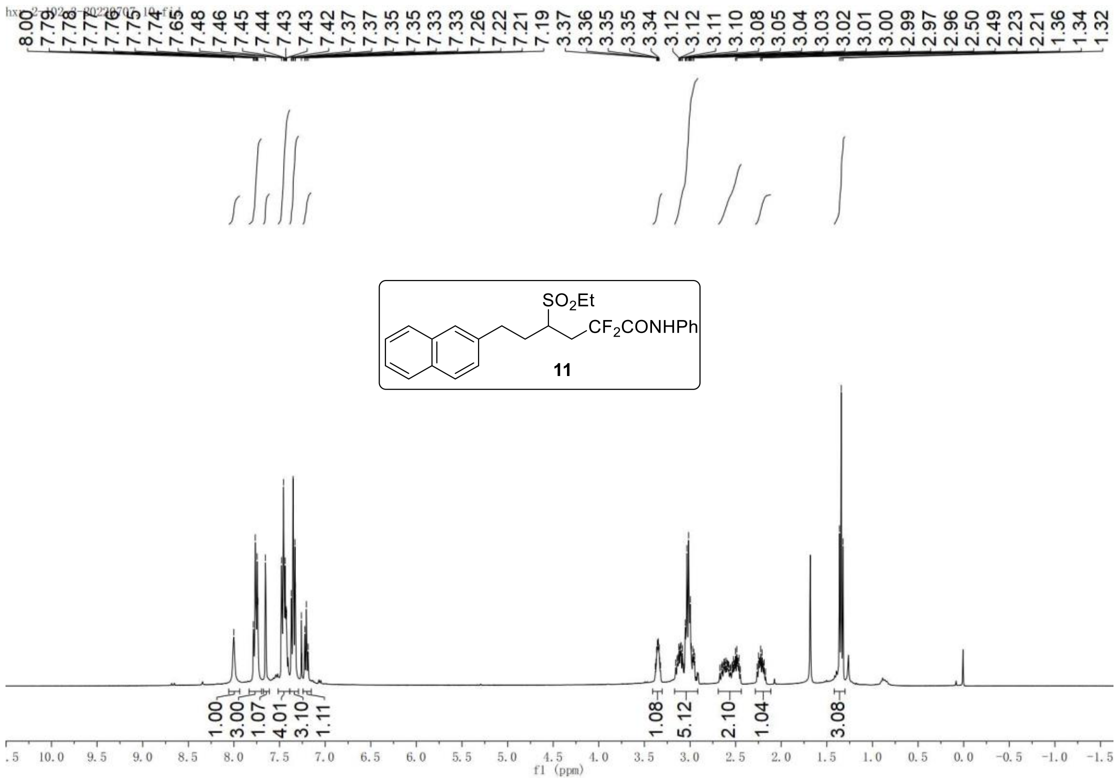

Supplementary Figure 76. ^{1}H NMR (400 MHz, $CDCl_3$) spectra of **11**

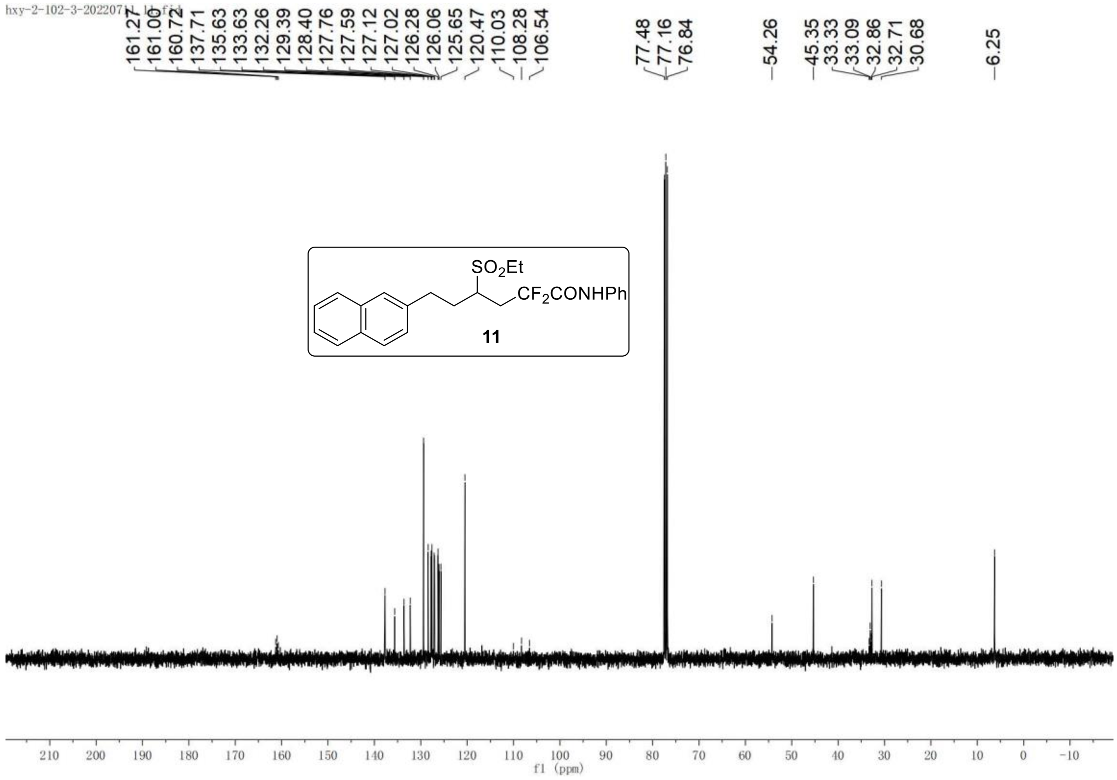

Supplementary Figure 77. ^{13}C NMR (101 MHz, $CDCl_3$) spectra of **11**

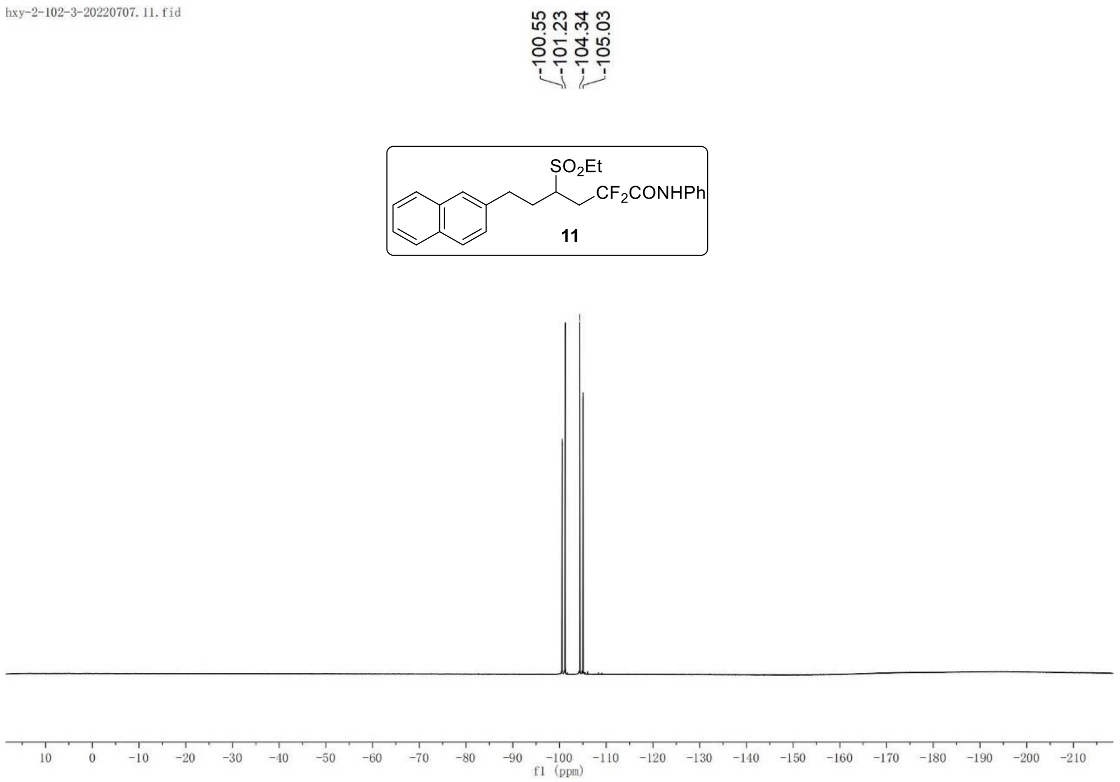

Supplementary Figure 78. ^{19}F NMR (376 MHz, $CDCl_3$) spectra of **11**

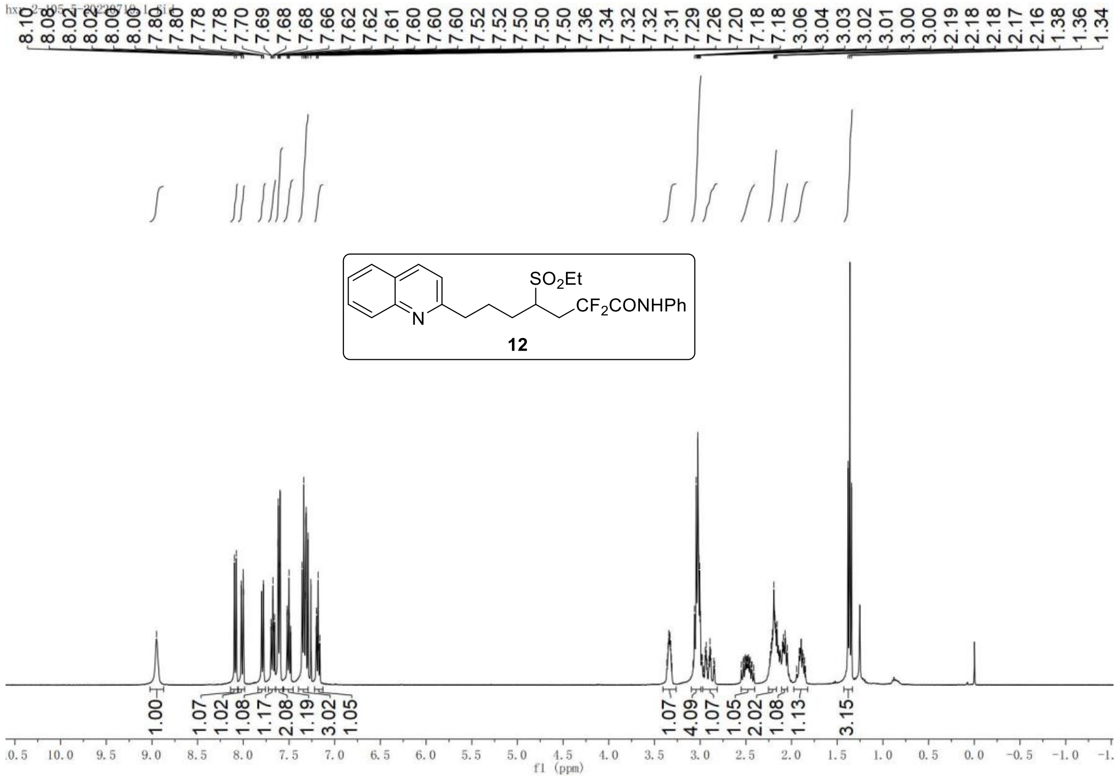

Supplementary Figure 79. ^{1}H NMR (400 MHz, $CDCl_3$) spectra of **12**

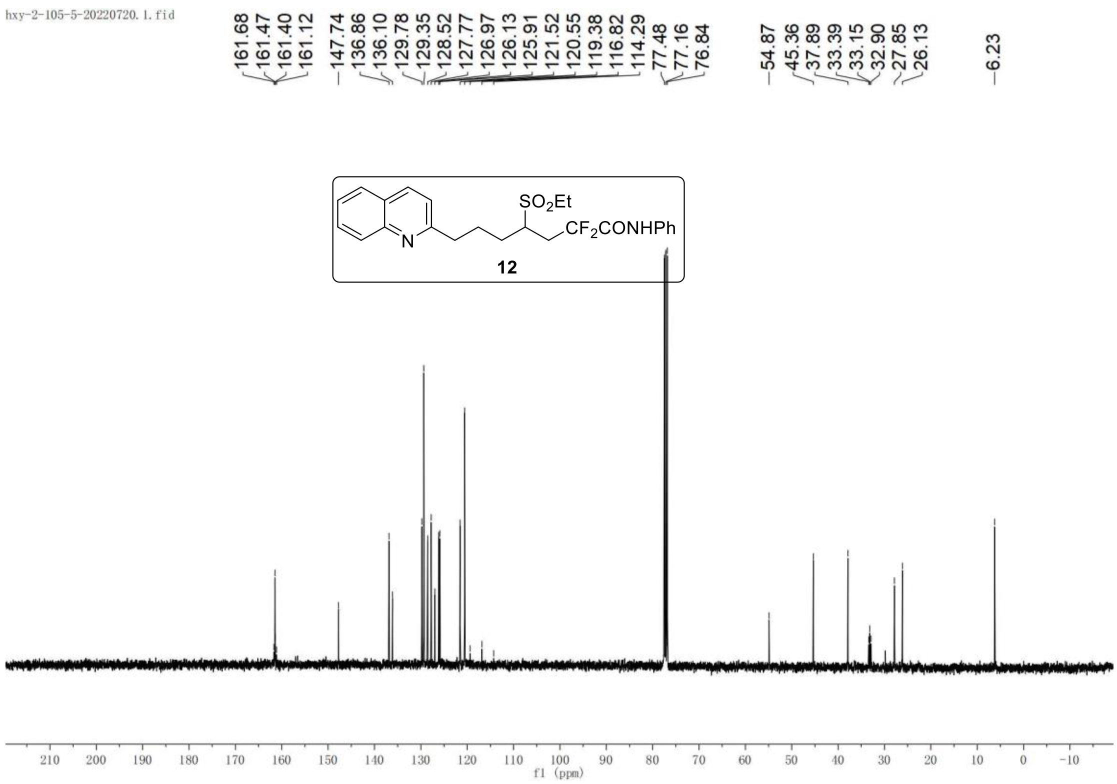

Supplementary Figure 80. ^{13}C NMR (101 MHz, $CDCl_3$) spectra of **12**

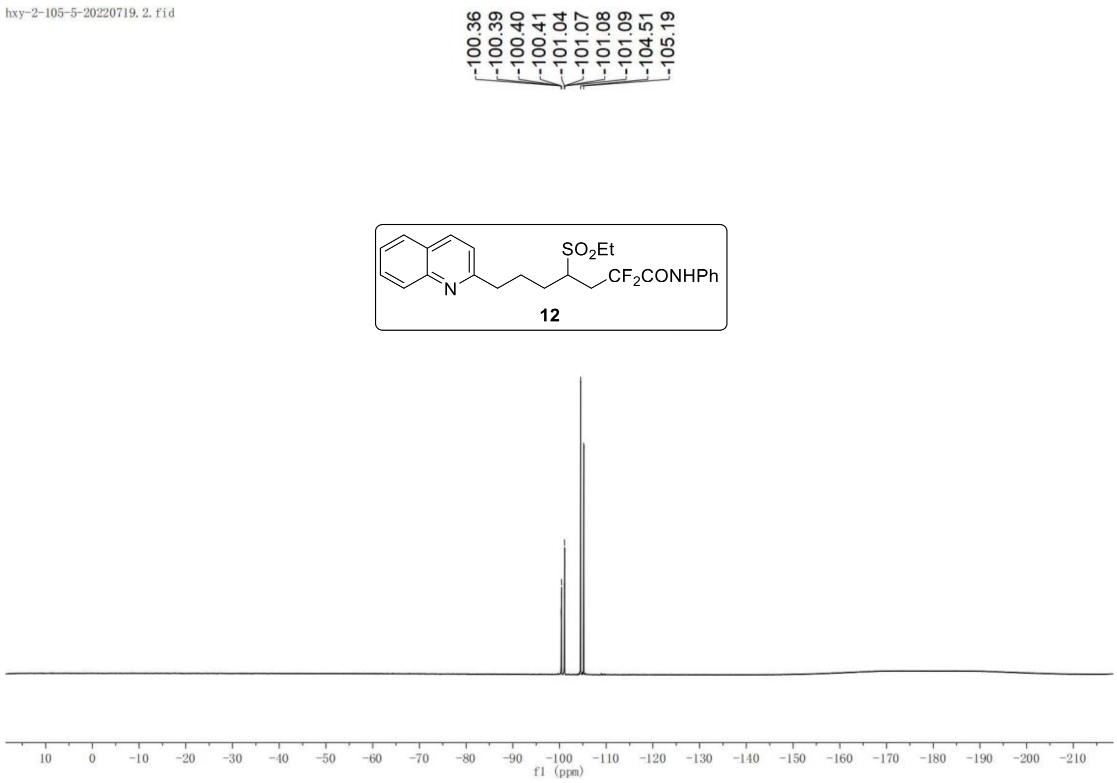

Supplementary Figure 81. ^{19}F NMR (376 MHz, $CDCl_3$) spectra of **12**

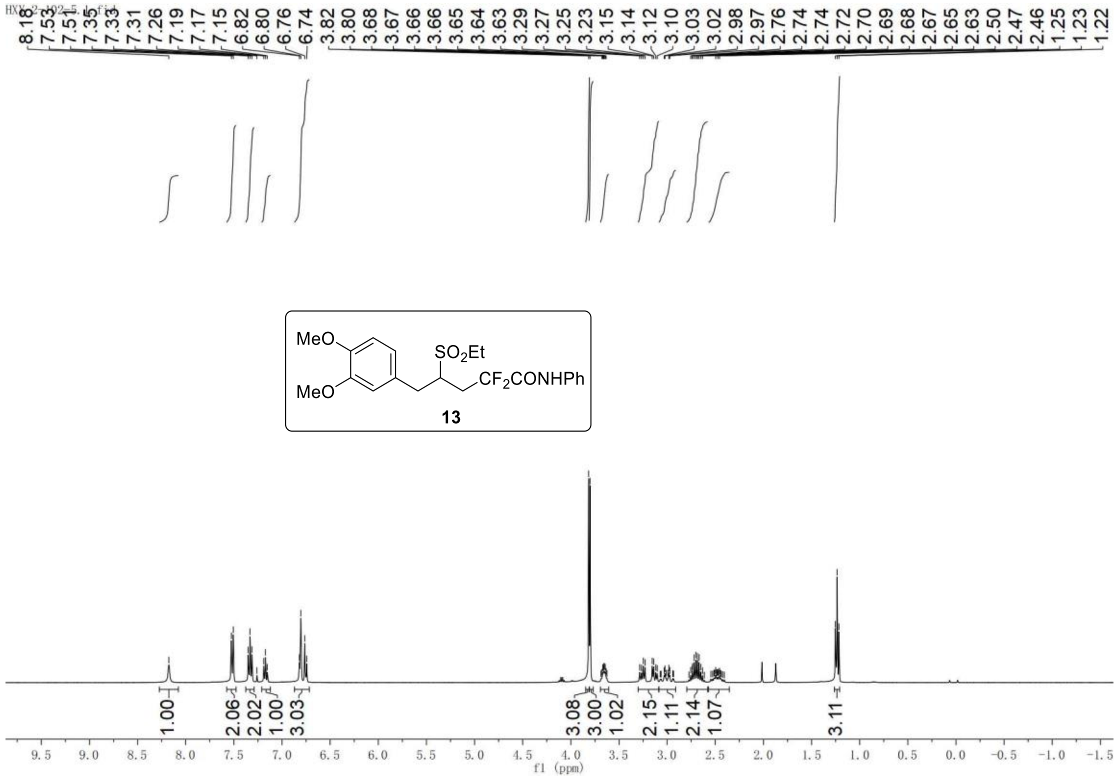

Supplementary Figure 82. ^{1}H NMR (400 MHz, $CDCl_3$) spectra of **13**

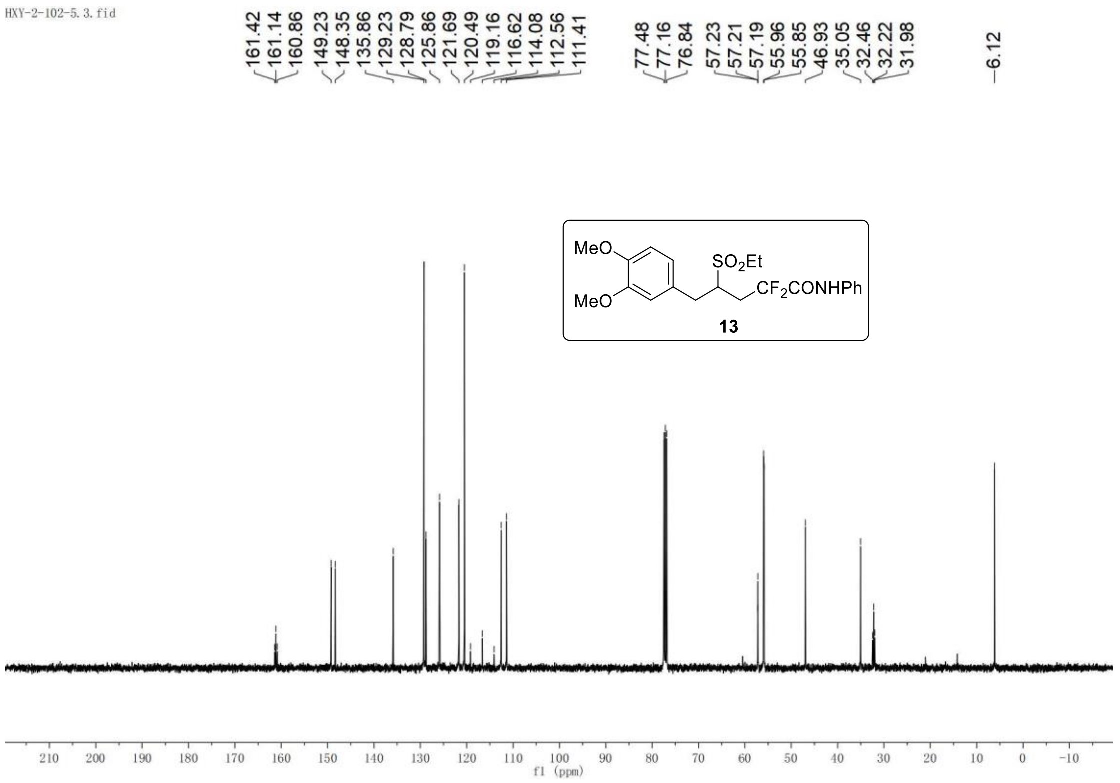

Supplementary Figure 83. ^{13}C NMR (101 MHz, $CDCl_3$) spectra of **13**

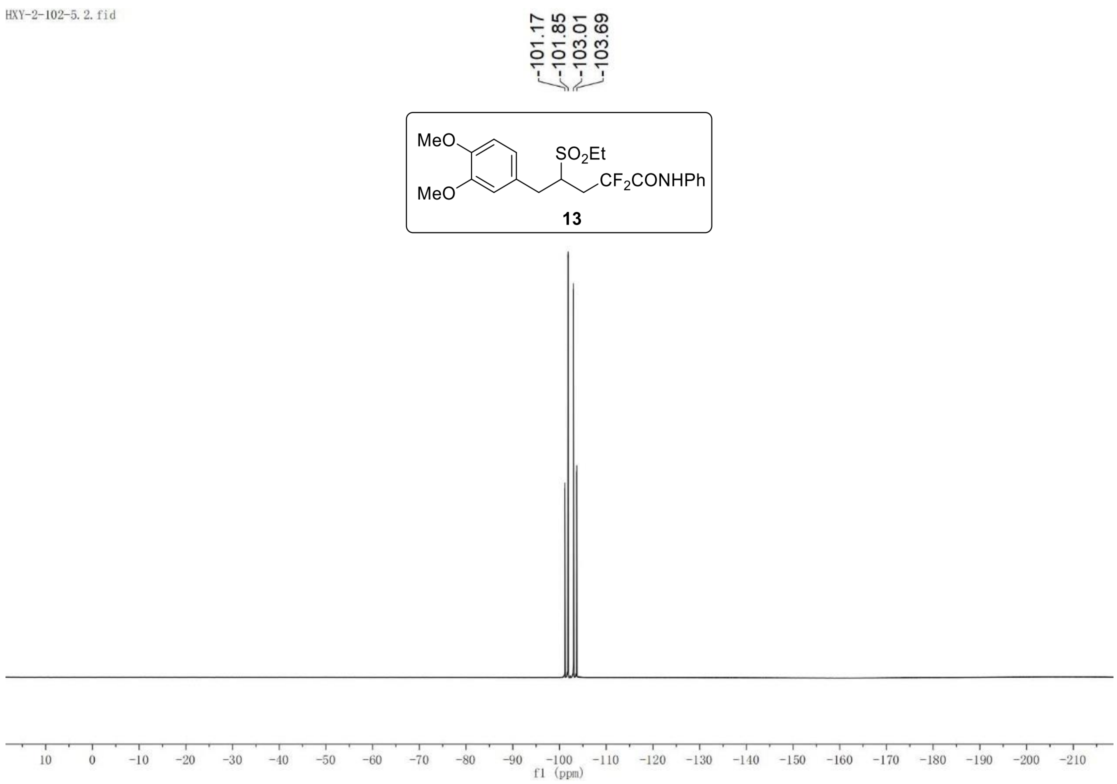

Supplementary Figure 84. ^{19}F NMR (376 MHz, $CDCl_3$) spectra of **13**

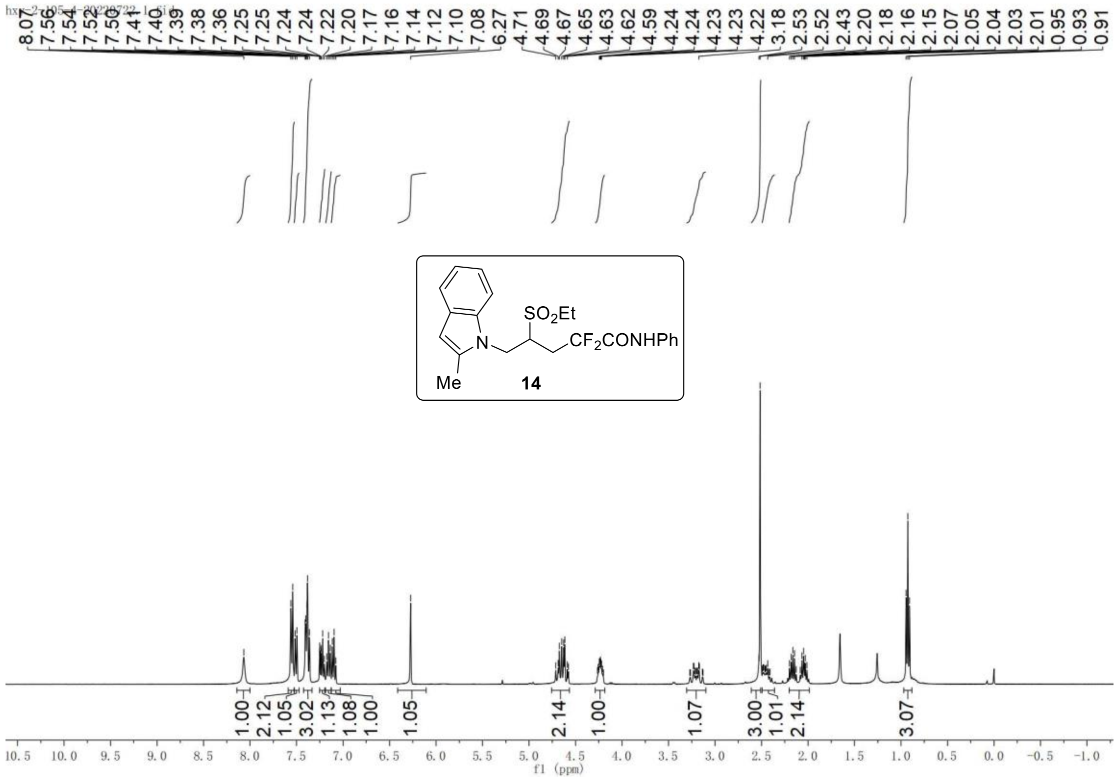

Supplementary Figure 85. ^{1}H NMR (400 MHz, $CDCl_3$) spectra of **14**

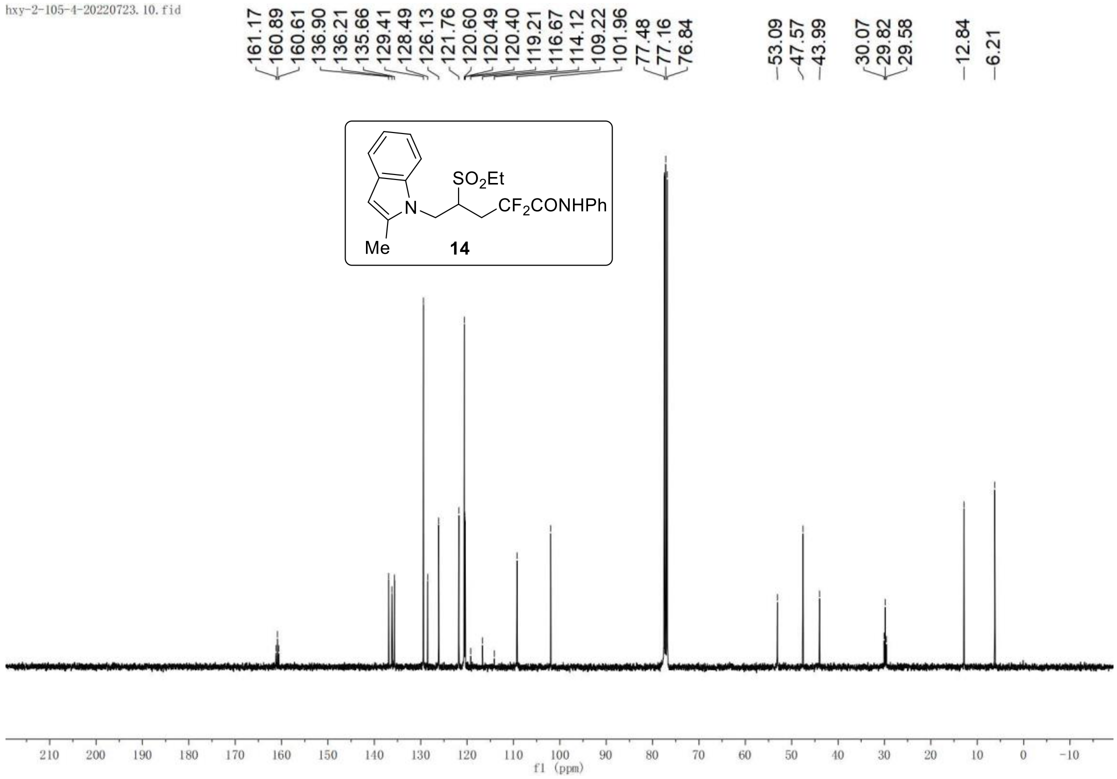

Supplementary Figure 86. ^{13}C NMR (101 MHz, $CDCl_3$) spectra of **14**

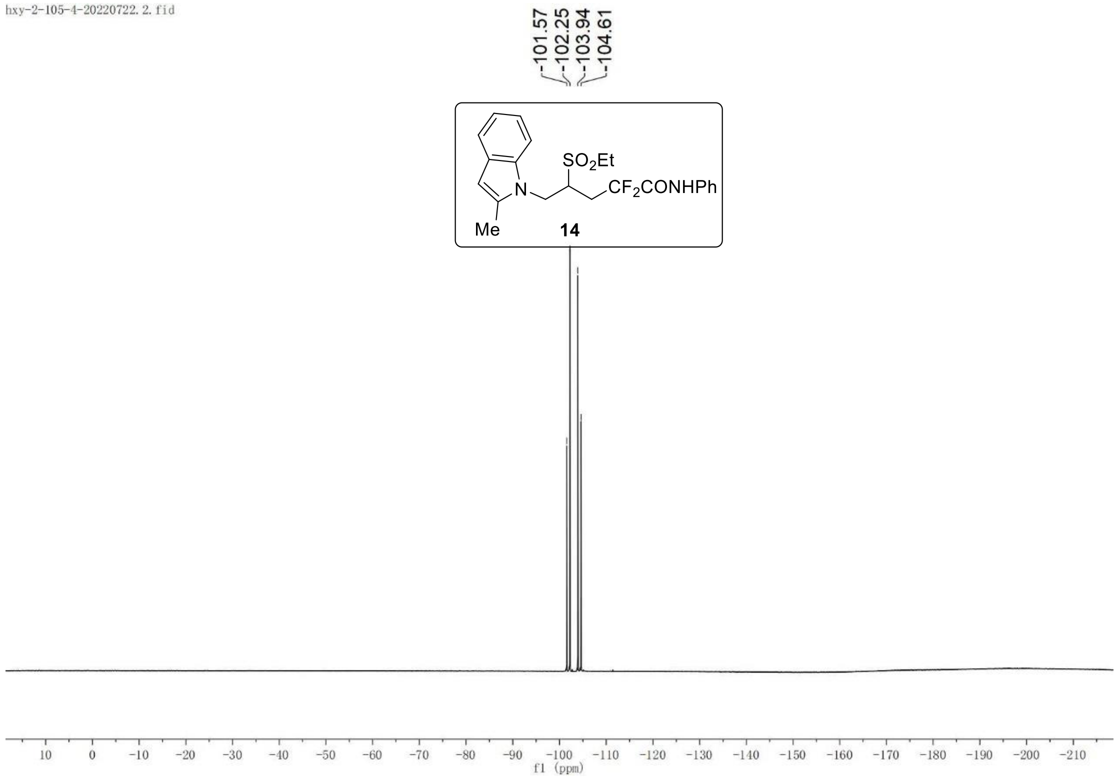

Supplementary Figure 87. ^{19}F NMR (376 MHz, $CDCl_3$) spectra of **14**

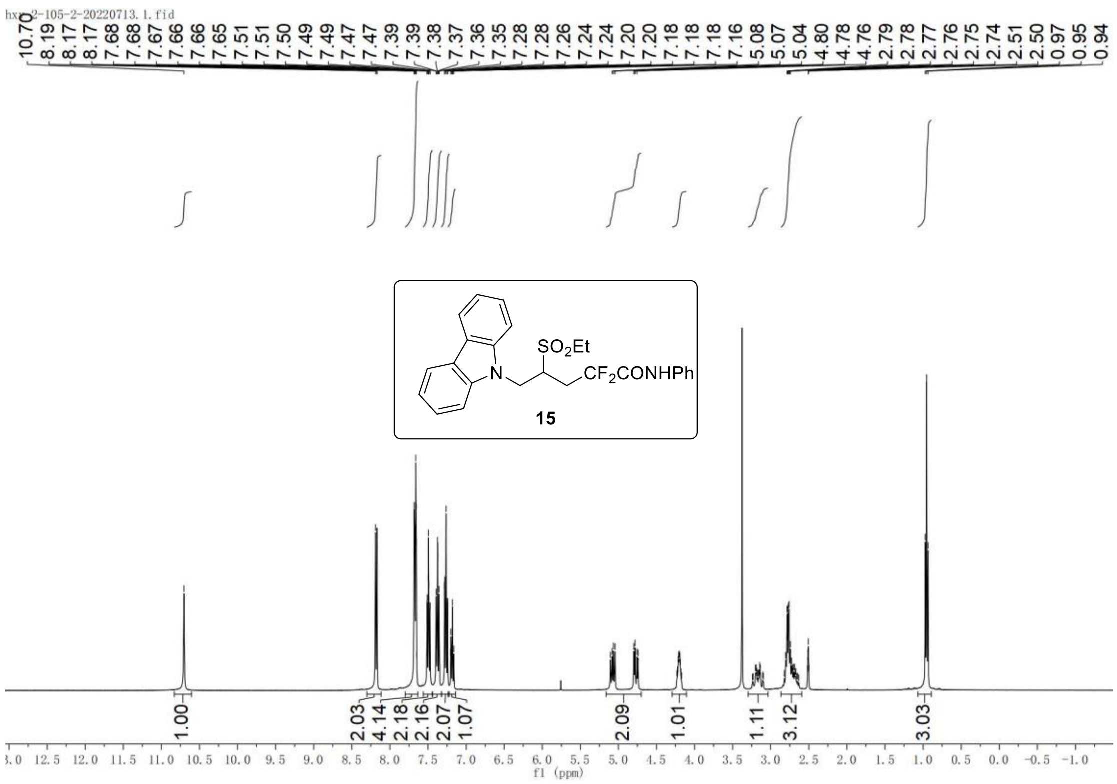

Supplementary Figure 88. ^{1}H NMR (400 MHz, DMSO-d_6) spectra of **15**

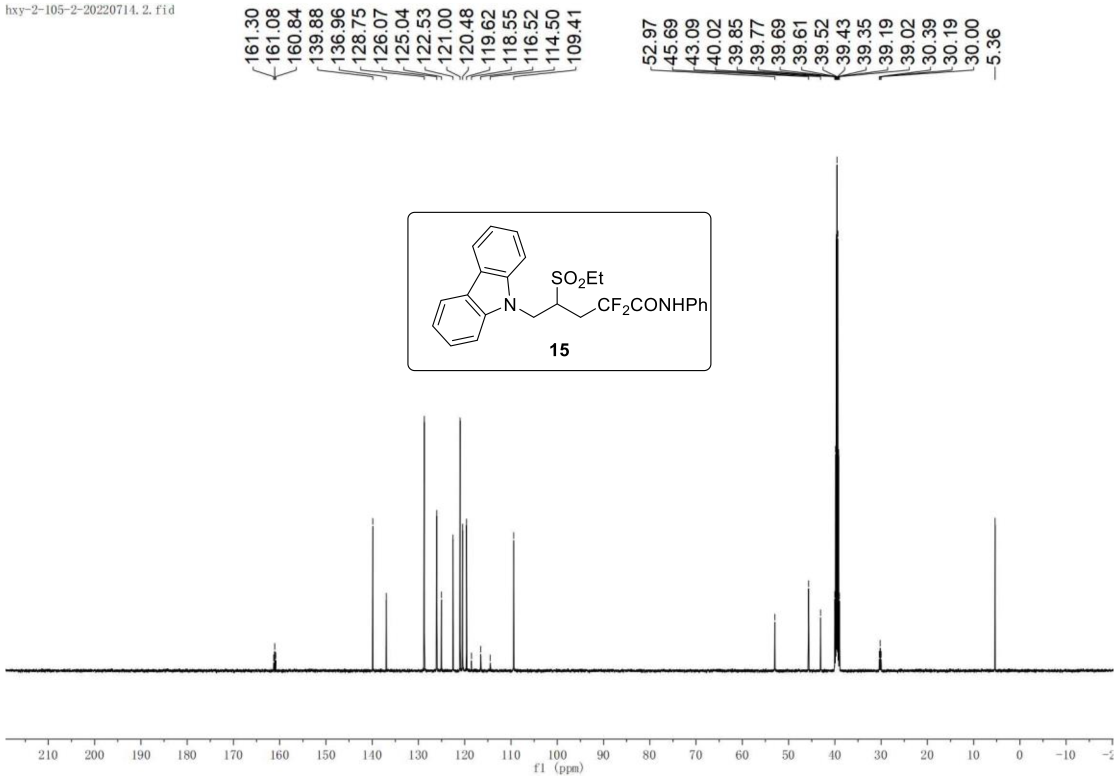

Supplementary Figure 89. ^{13}C NMR (126 MHz, DMSO-d_6) spectra of **15**

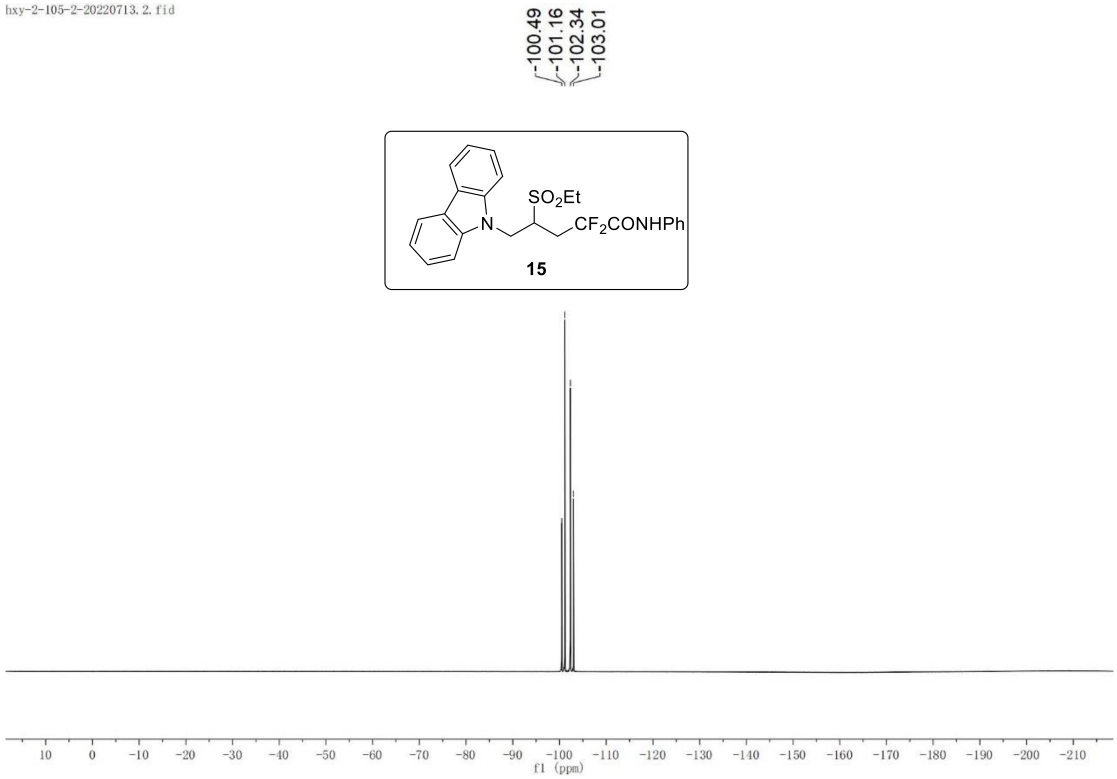

Supplementary Figure 90. ^{19}F NMR (376 MHz, DMSO-d_6) spectra of **15**

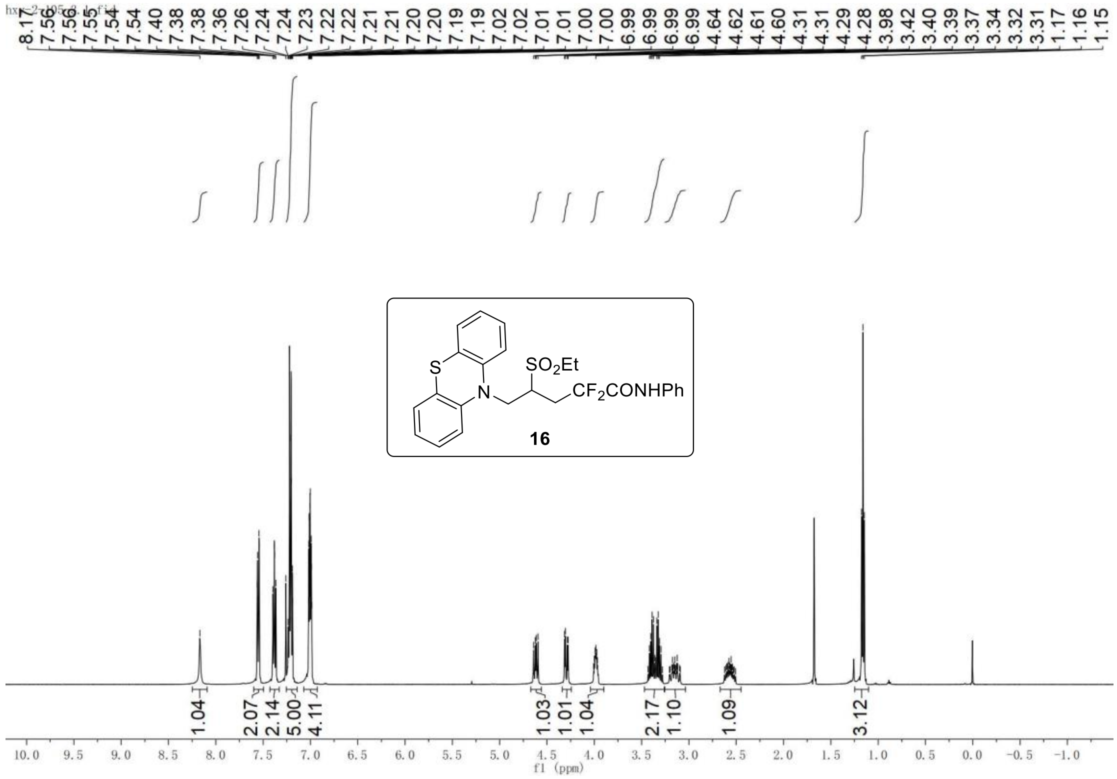

Supplementary Figure 91. ^{1}H NMR (500 MHz, $CDCl_3$) spectra of **16**

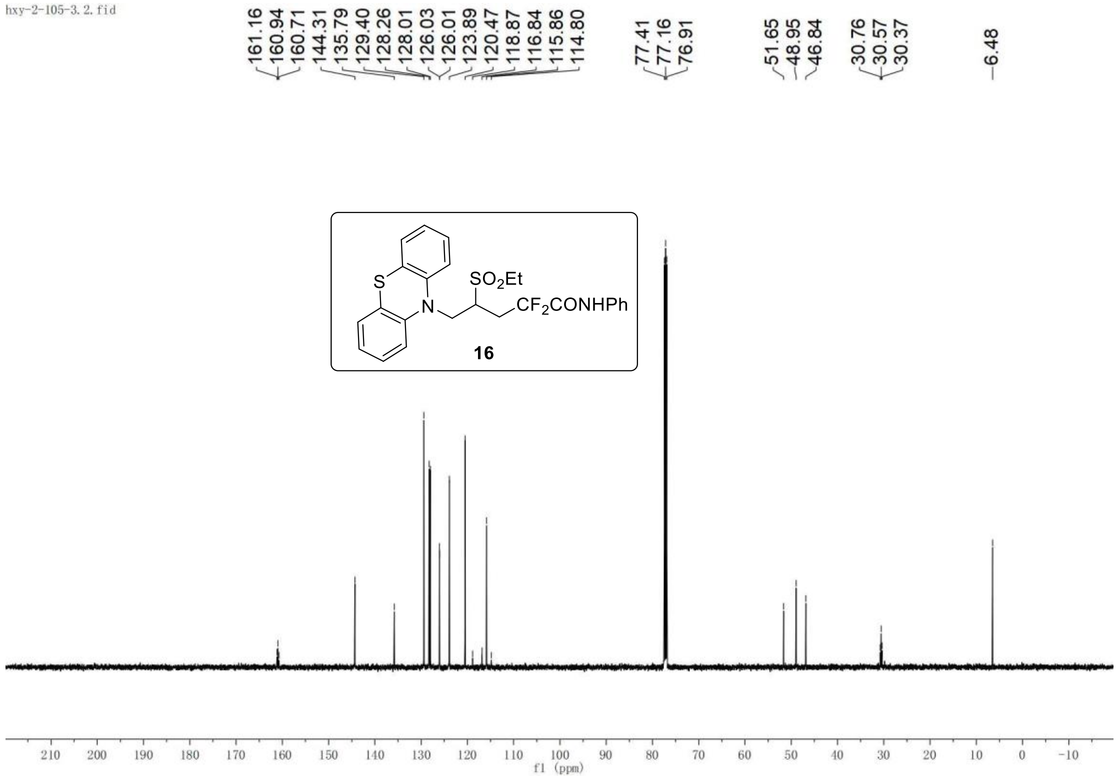

Supplementary Figure 92. ^{13}C NMR (126 MHz, $CDCl_3$) spectra of **16**

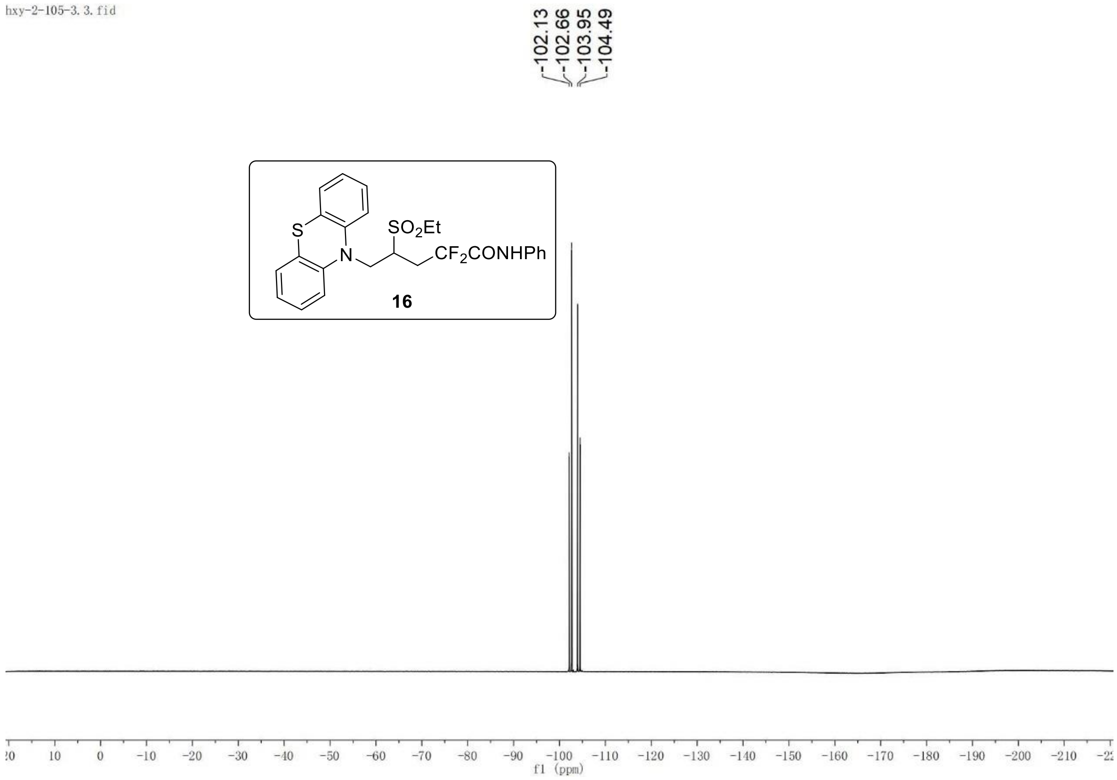

Supplementary Figure 93. ^{19}F NMR (471 MHz, $CDCl_3$) spectra of **16**

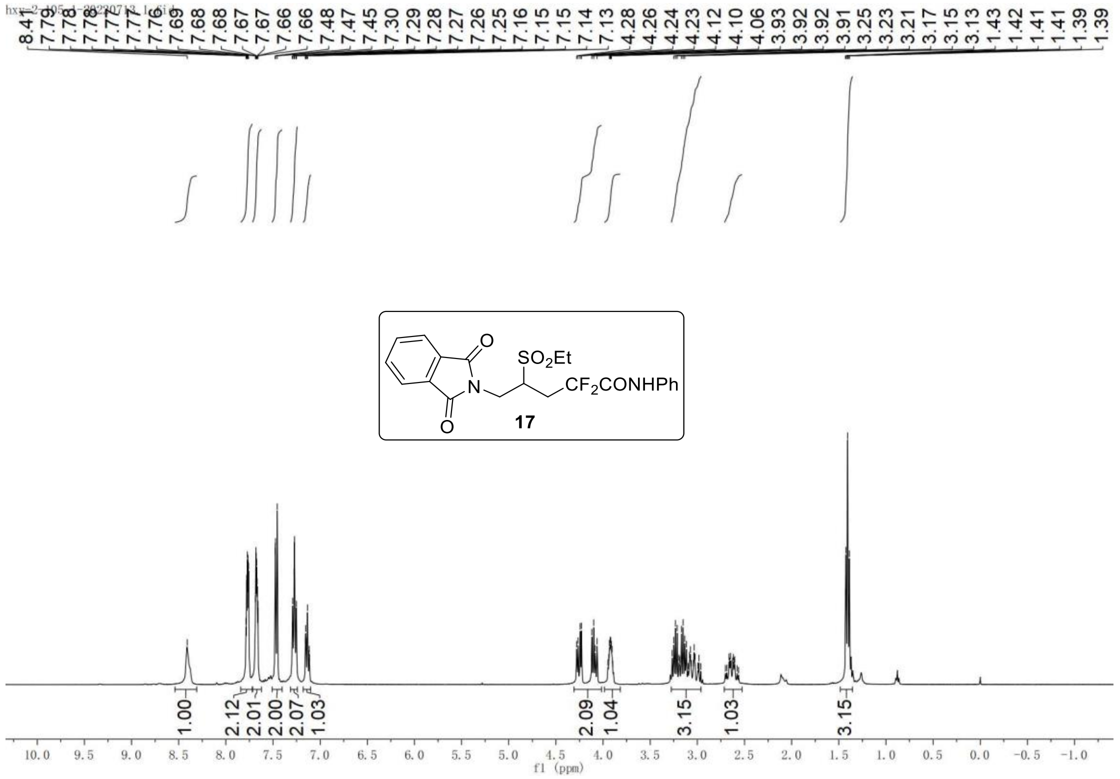

Supplementary Figure 94. ^{1}H NMR (400 MHz, $CDCl_3$) spectra of **17**

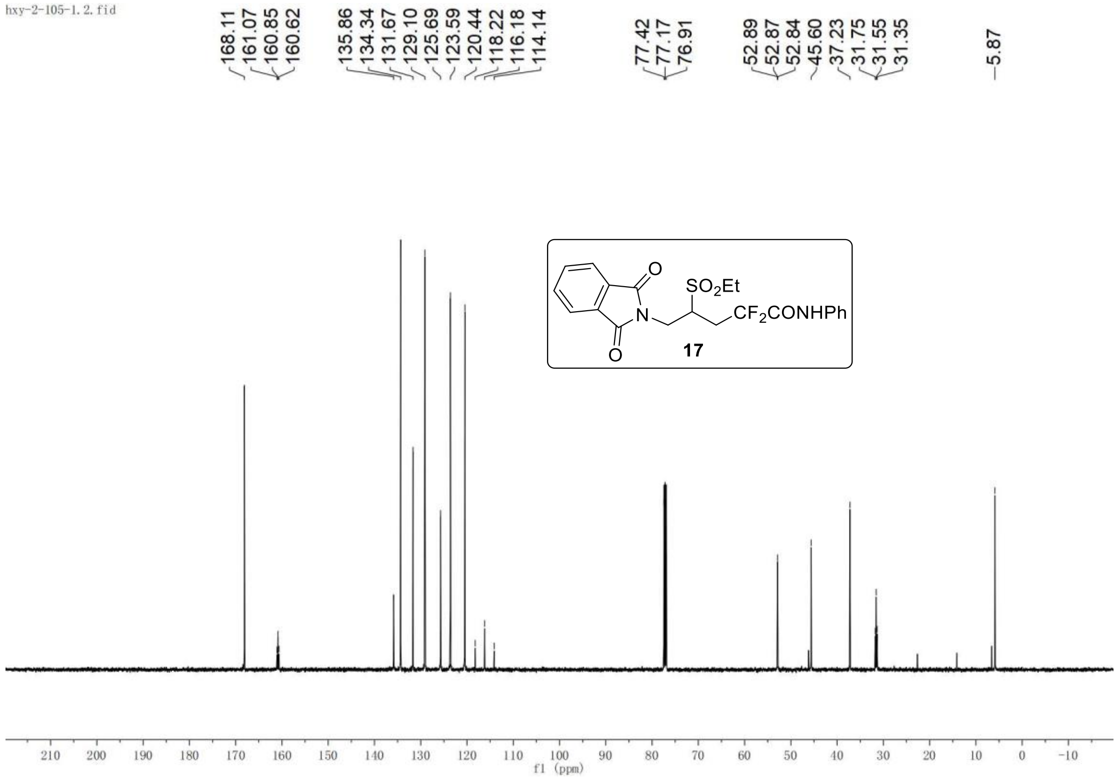

Supplementary Figure 95. ^{13}C NMR (126 MHz, $CDCl_3$) spectra of **17**

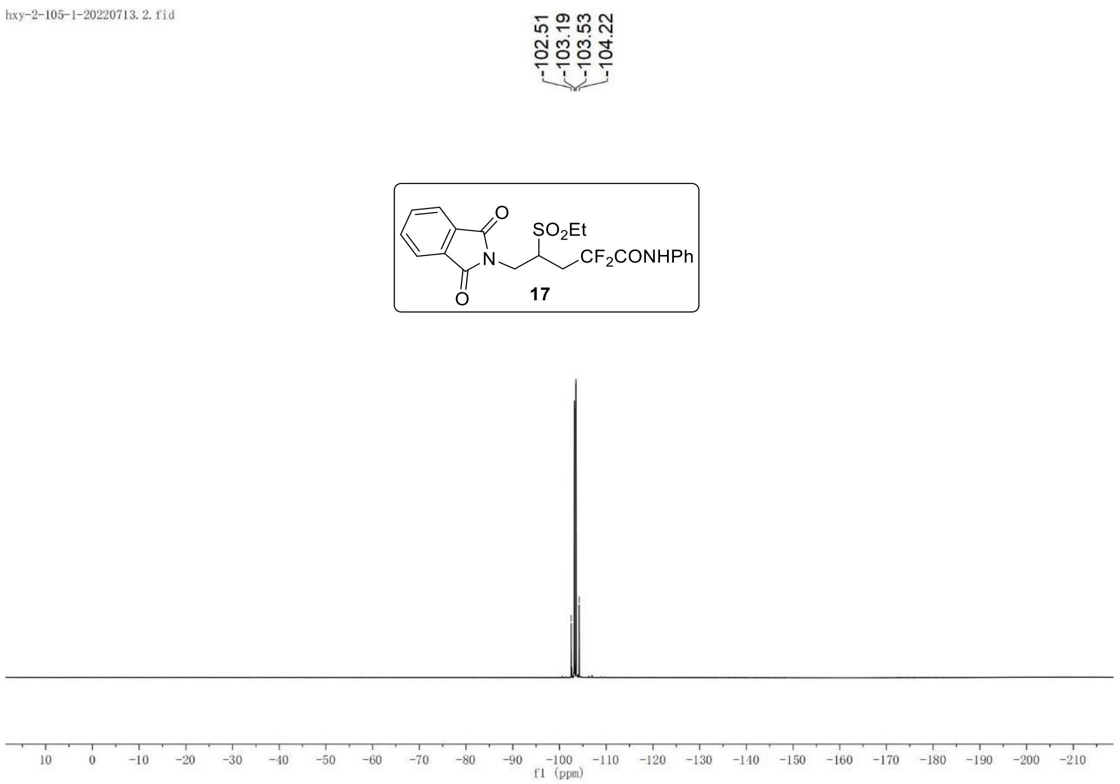

Supplementary Figure 96. ^{19}F NMR (376 MHz, $CDCl_3$) spectra of **17**

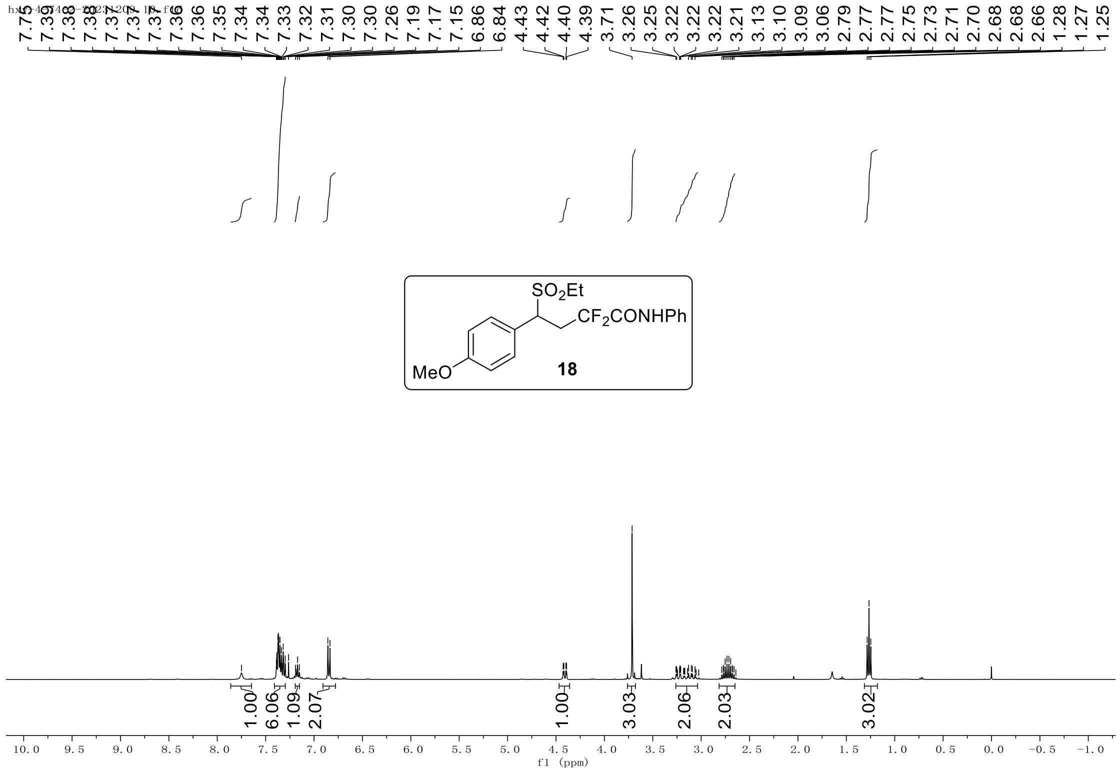

Supplementary Figure 97. ^{1}H NMR (400 MHz, $CDCl_3$) spectra of **18**

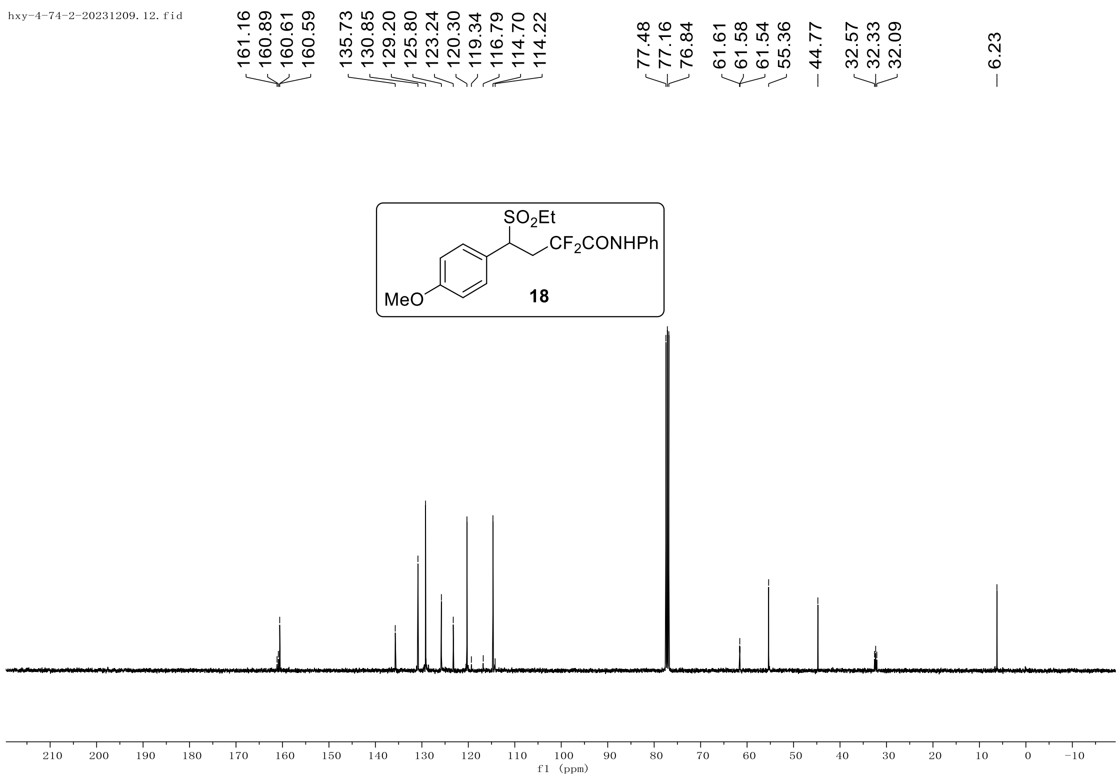

Supplementary Figure 98. ^{13}C NMR (101 MHz, $CDCl_3$) spectra of **18**

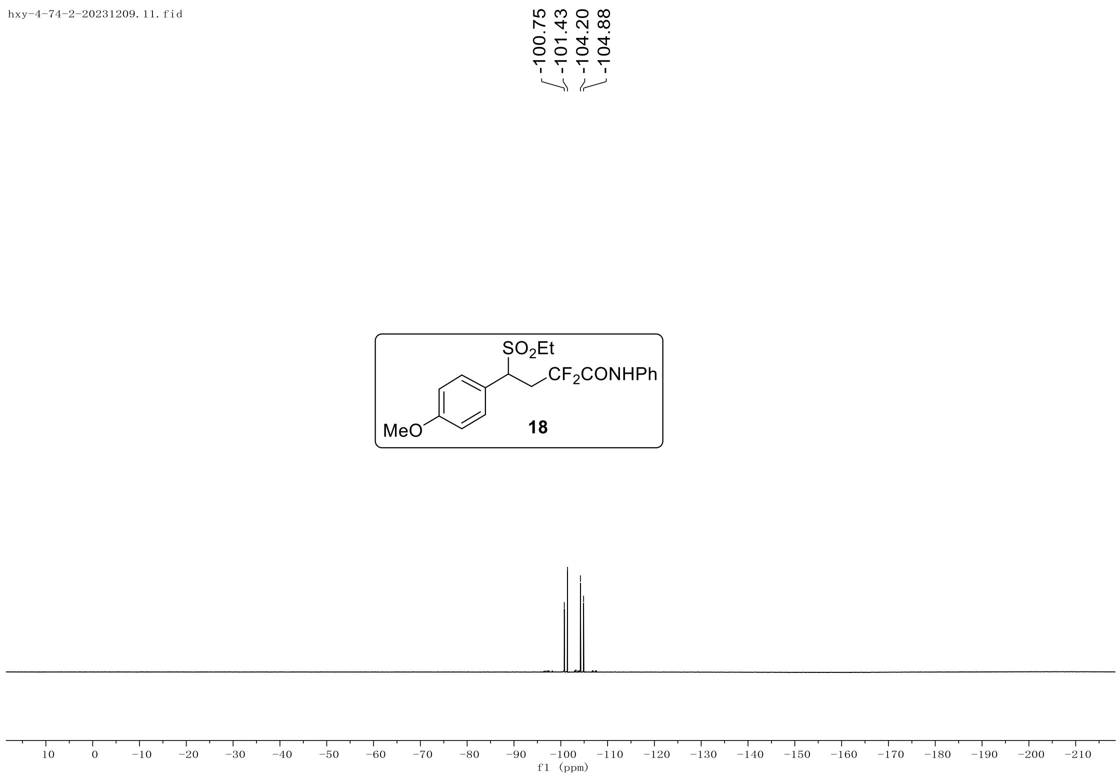

Supplementary Figure 99. ^{19}F NMR (376 MHz, $CDCl_3$) spectra of **18**

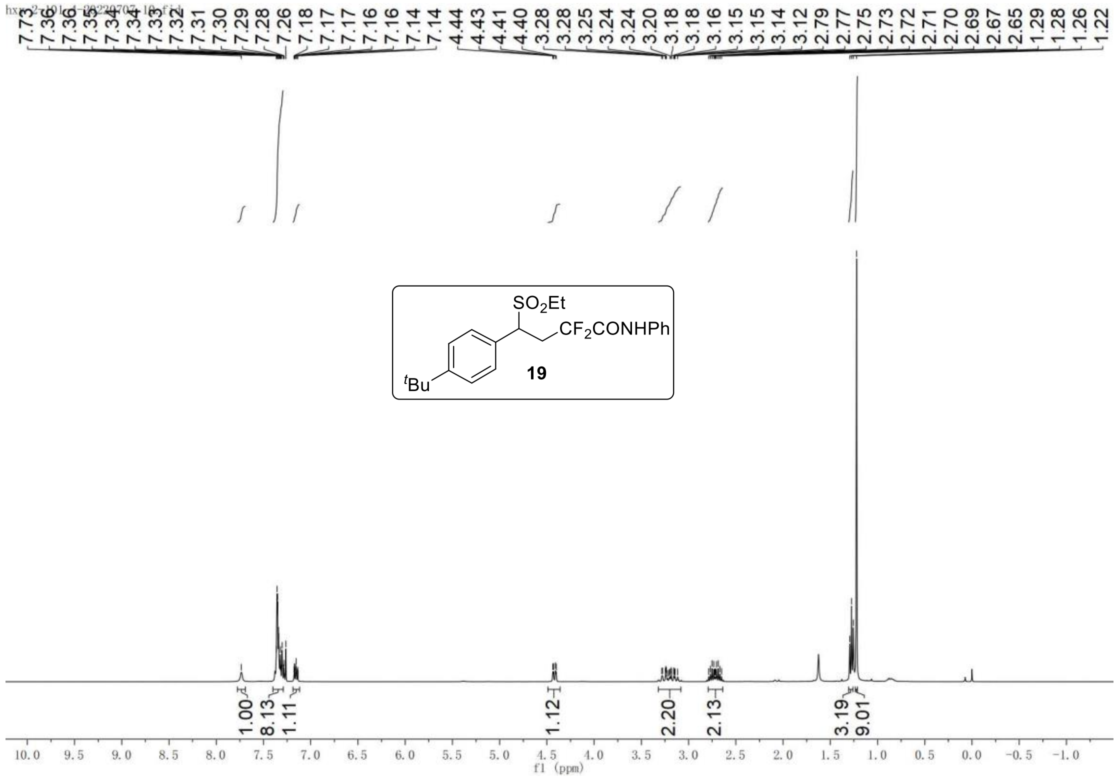

Supplementary Figure 100. ^{1}H NMR (400 MHz, $CDCl_3$) spectra of **19**

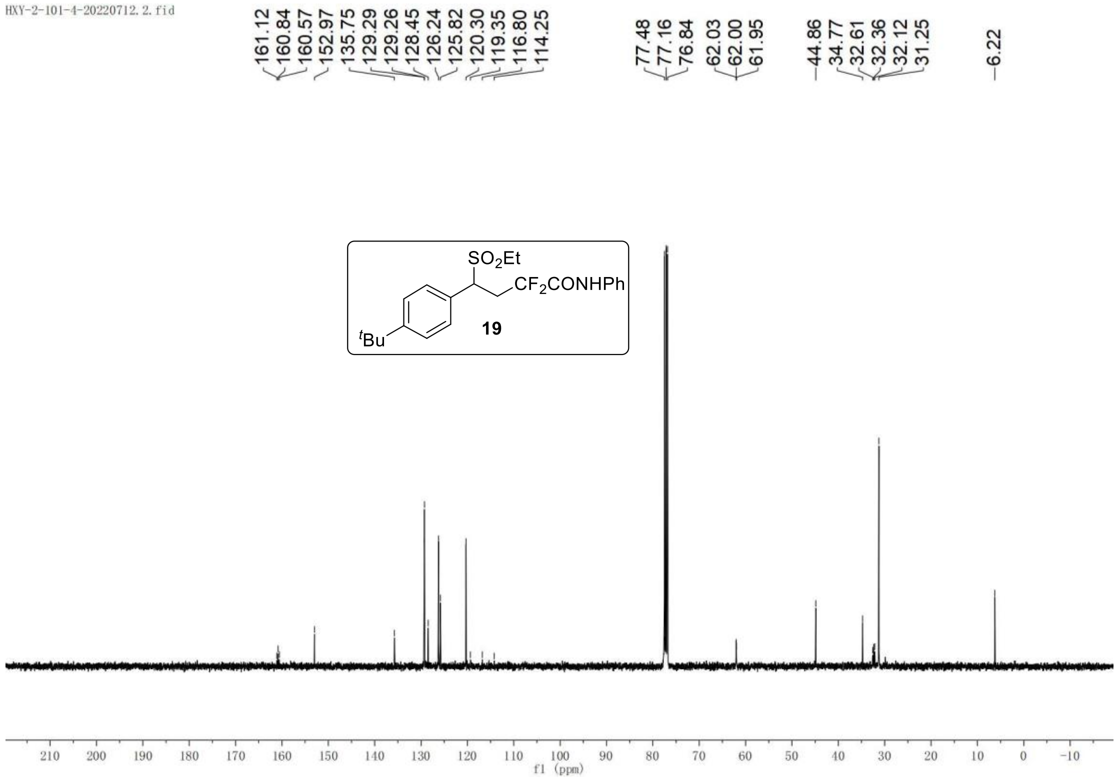

Supplementary Figure 101. ^{13}C NMR (101 MHz, $CDCl_3$) spectra of **19**

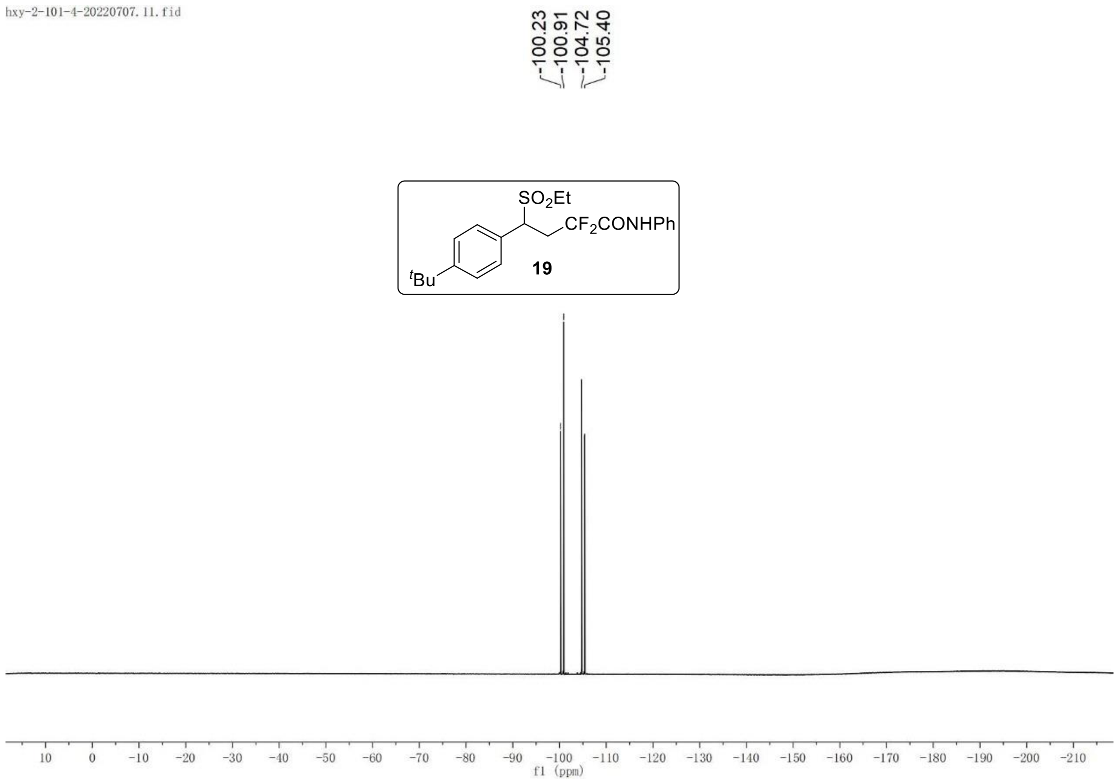

Supplementary Figure 102. ^{19}F NMR (376 MHz, $CDCl_3$) spectra of **19**

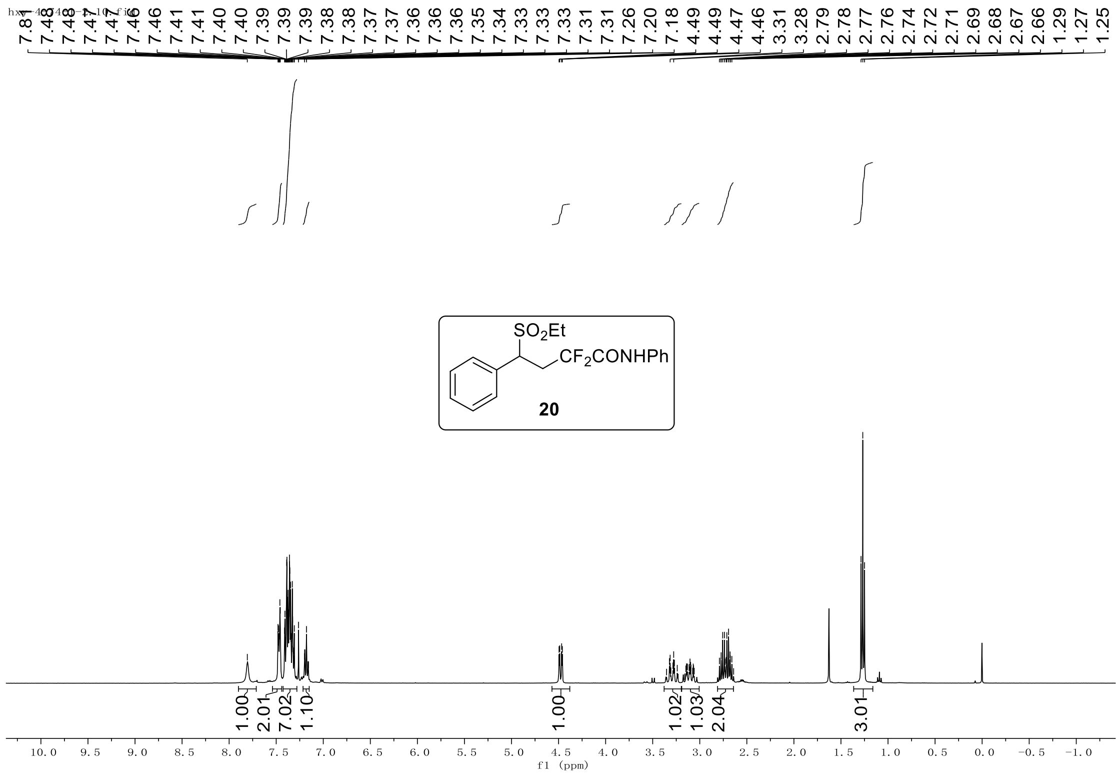

Supplementary Figure 103. ^{1}H NMR (400 MHz, $CDCl_3$) spectra of **20**

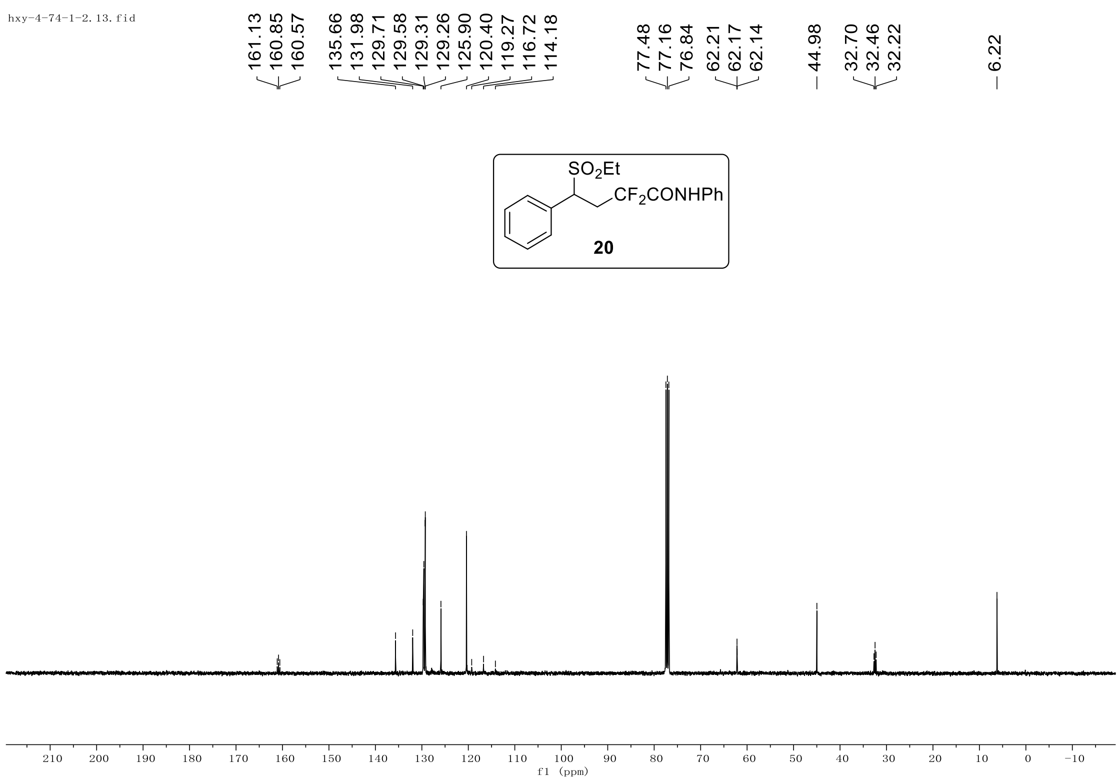

Supplementary Figure 104. ^{13}C NMR (101MHz, $CDCl_3$) spectra of **20**

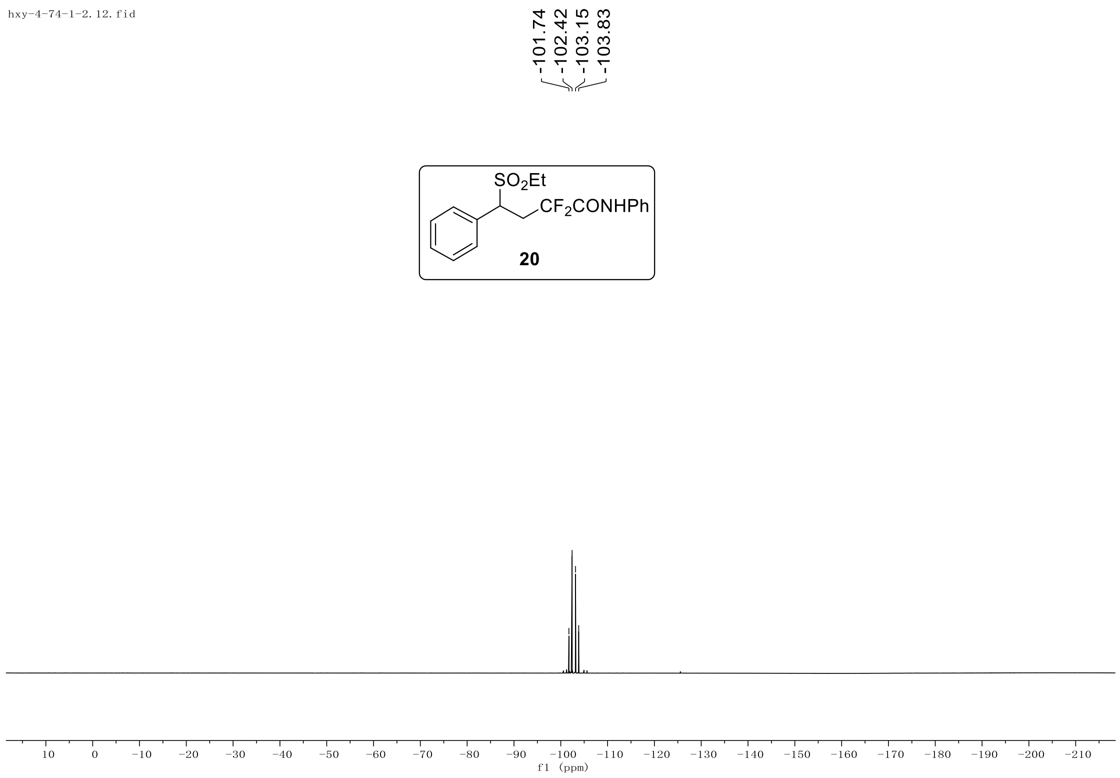

Supplementary Figure 105. ^{19}F NMR (376 MHz, $CDCl_3$) spectra of **20**

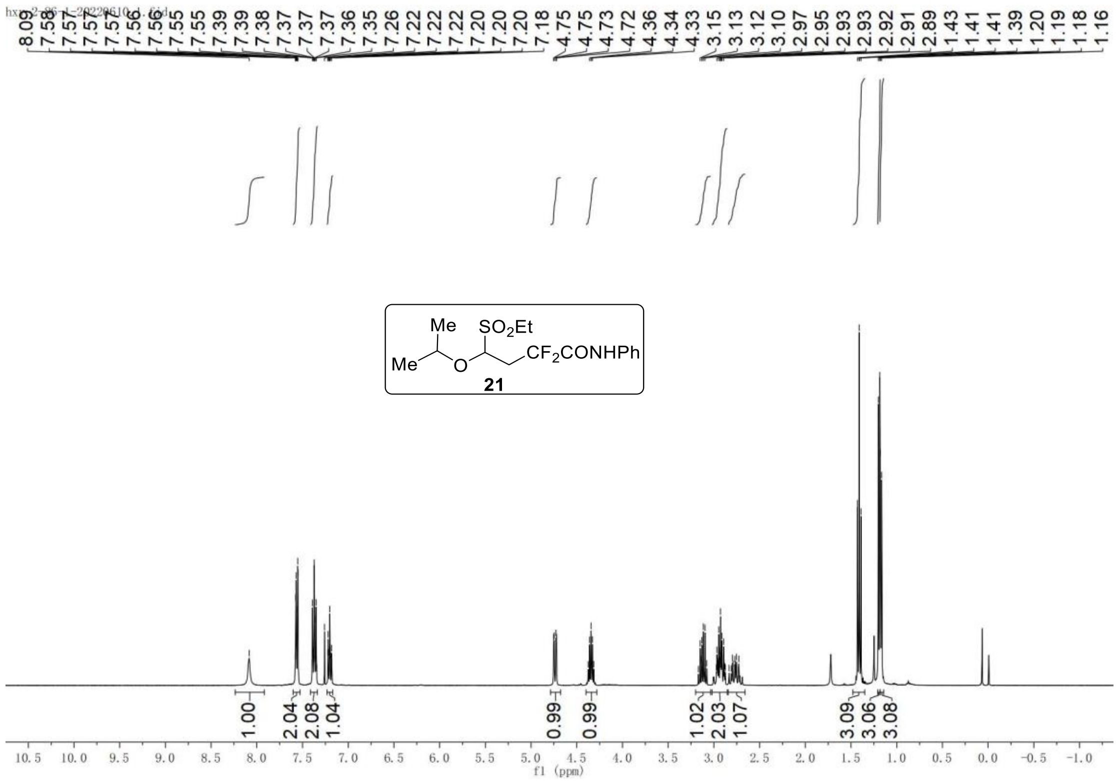

Supplementary Figure 106. ^{1}H NMR (400 MHz, $CDCl_3$) spectra of **21**

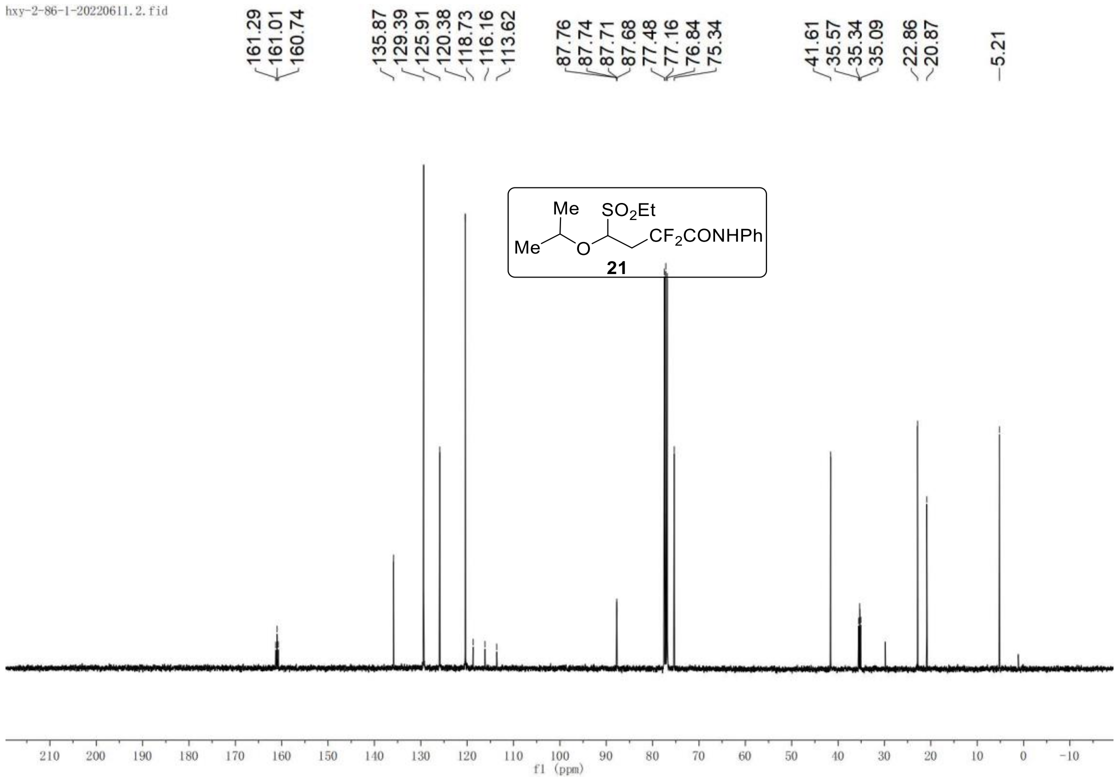

Supplementary Figure 107. ^{13}C NMR (101 MHz, $CDCl_3$) spectra of **21**

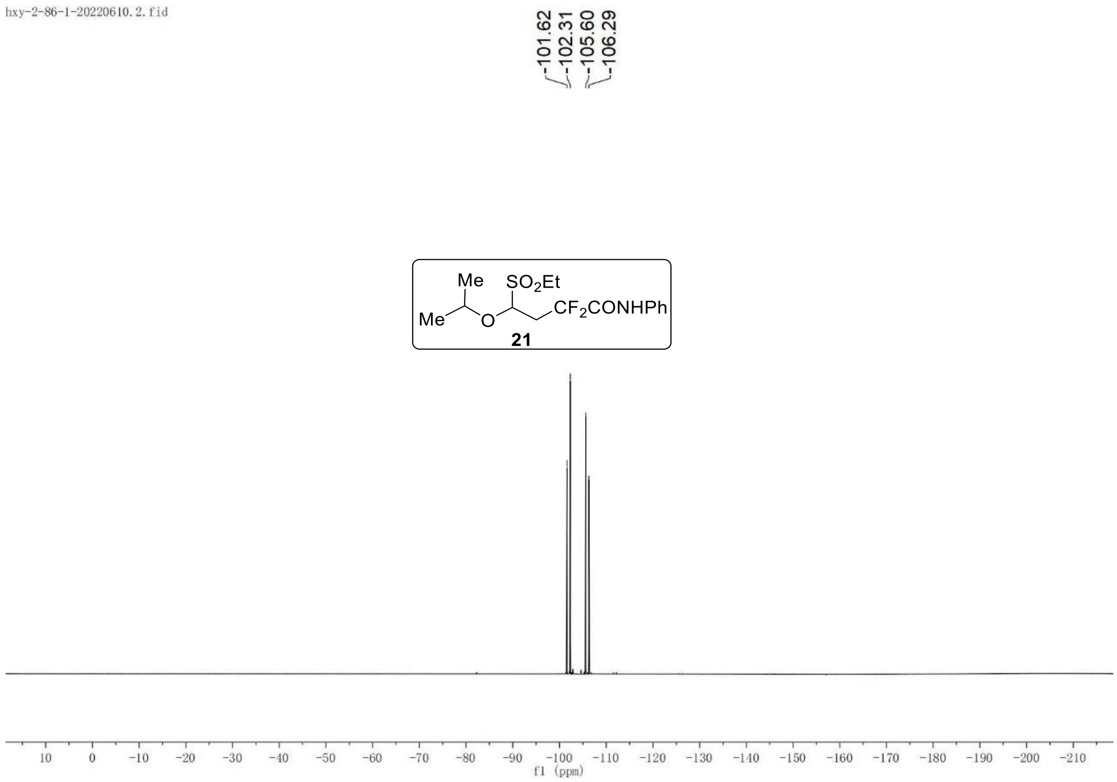

Supplementary Figure 108. ^{19}C NMR (376 MHz, $CDCl_3$) spectra of **21**

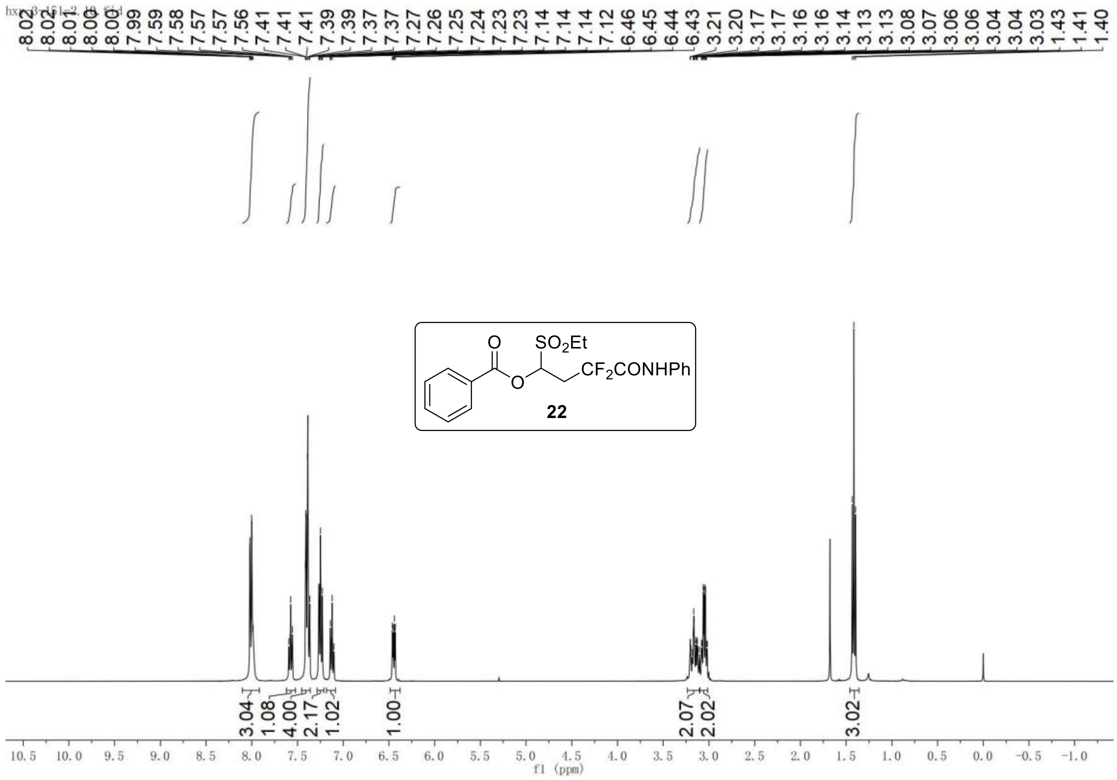

Supplementary Figure 109. ^{1}H NMR (400 MHz, $CDCl_3$) spectra of **22**

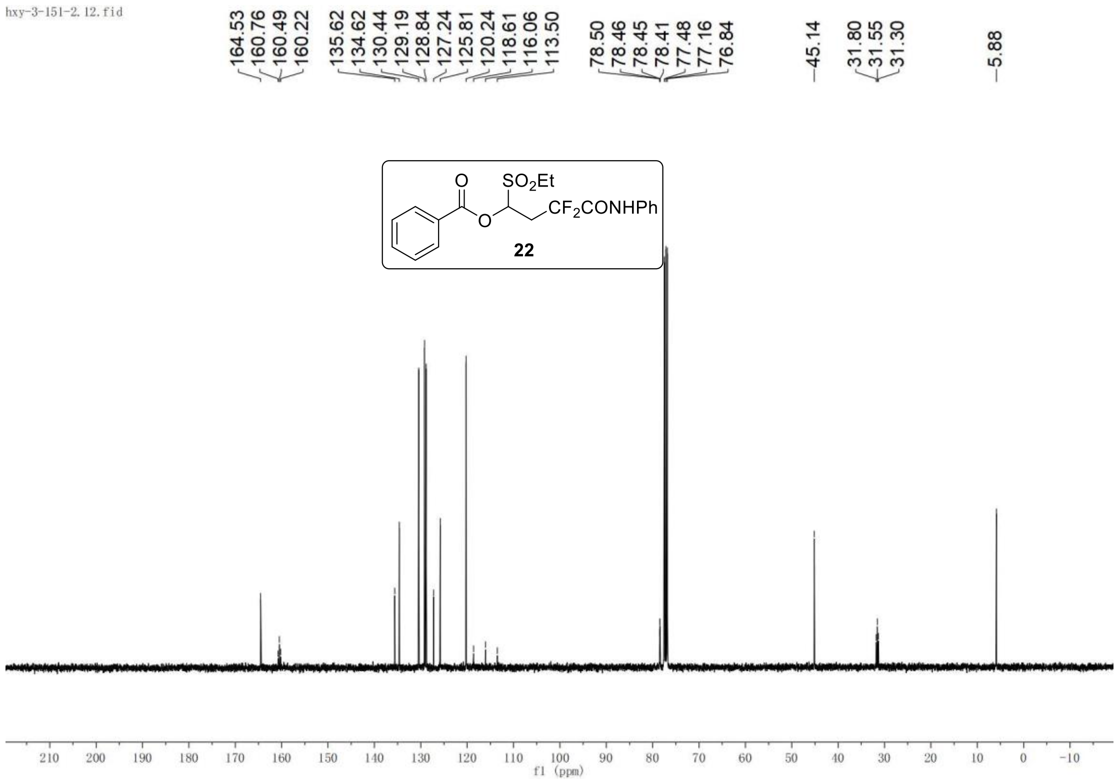

Supplementary Figure 110. ^{13}C NMR (101 MHz, $CDCl_3$) spectra of **22**

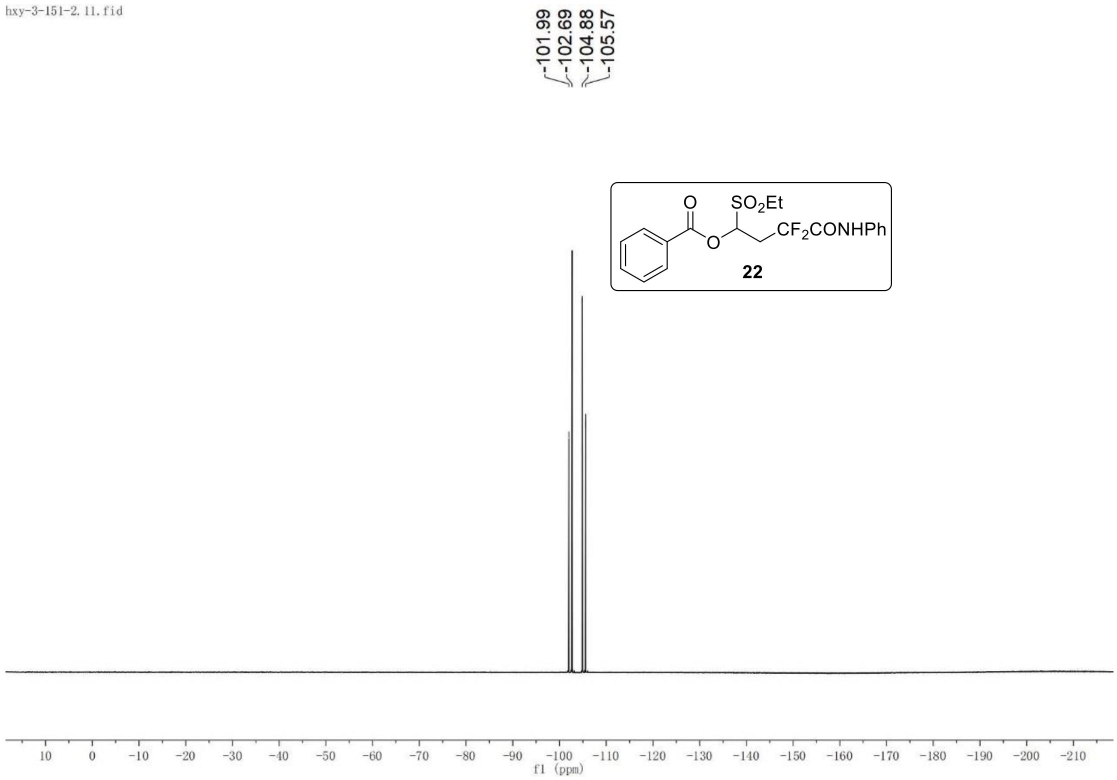

Supplementary Figure 111. ^{19}F NMR (376 MHz, $CDCl_3$) spectra of **22**

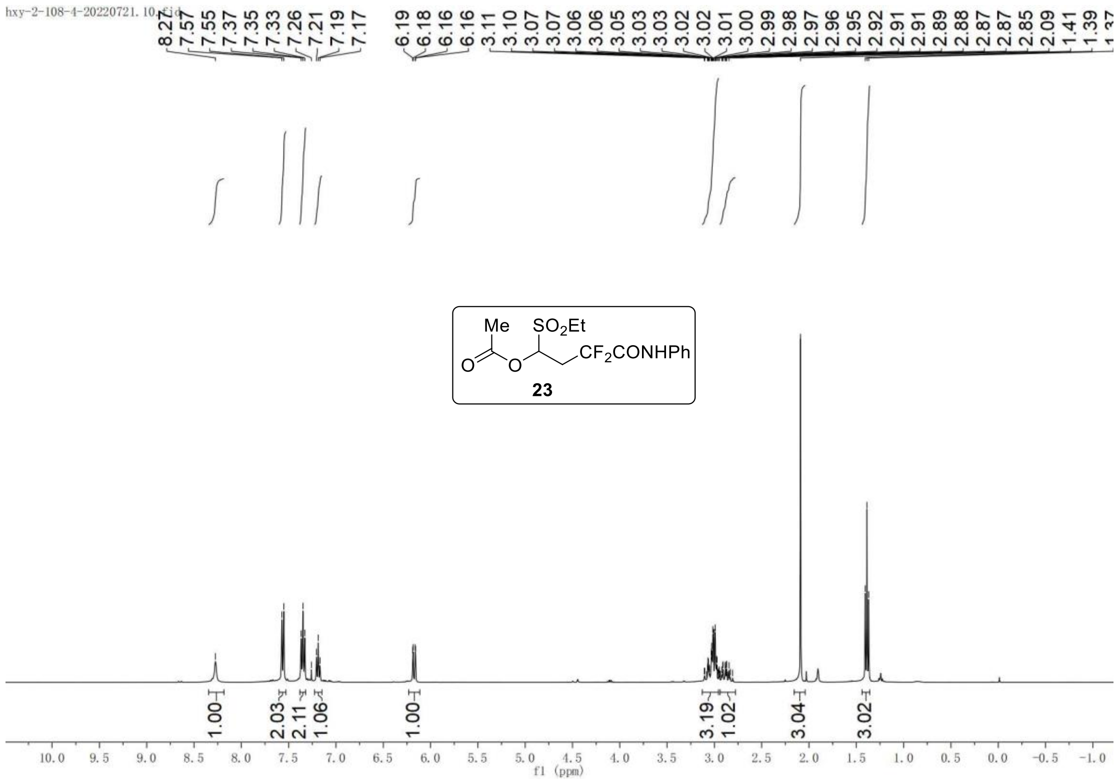

Supplementary Figure 112. ^{1}H NMR (400 MHz, $CDCl_3$) spectra of **23**

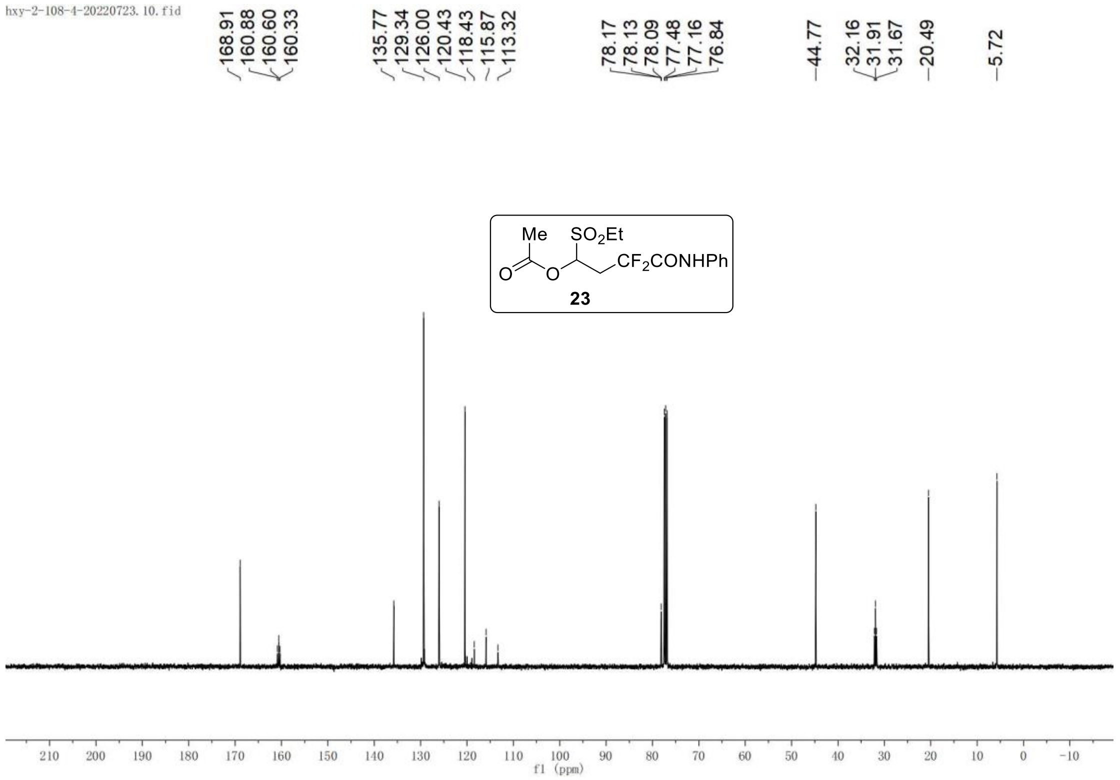

Supplementary Figure 113. ^{13}C NMR (101 MHz, $CDCl_3$) spectra of **23**

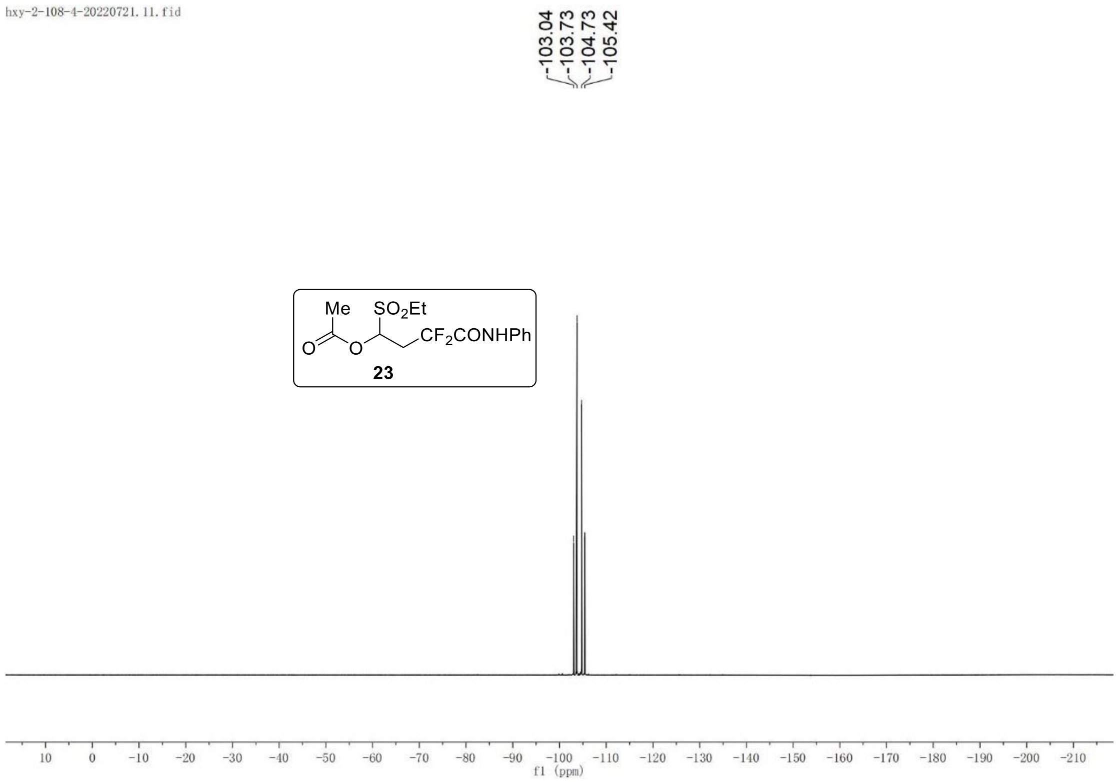

Supplementary Figure 114. ^{19}F NMR (376 MHz, $CDCl_3$) spectra of **23**

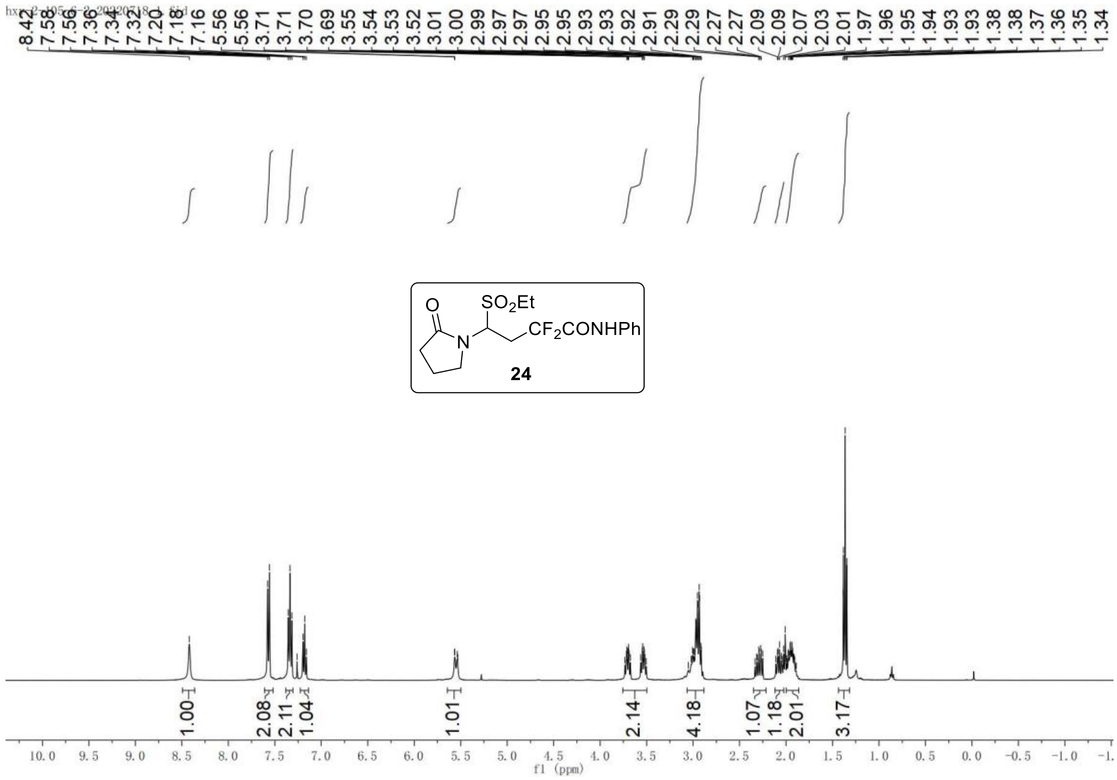

Supplementary Figure 115. ^{1}H NMR (400 MHz, $CDCl_3$) spectra of **24**

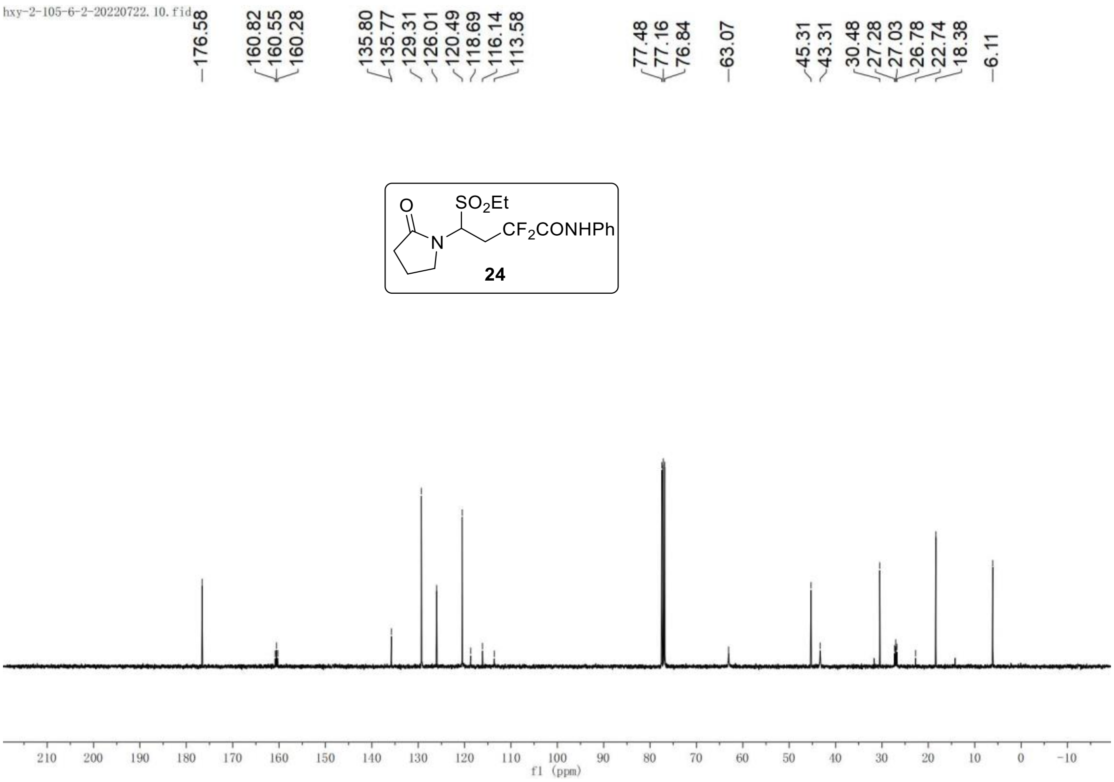

Supplementary Figure 116. ^{13}C NMR (101 MHz, $CDCl_3$) spectra of **24**

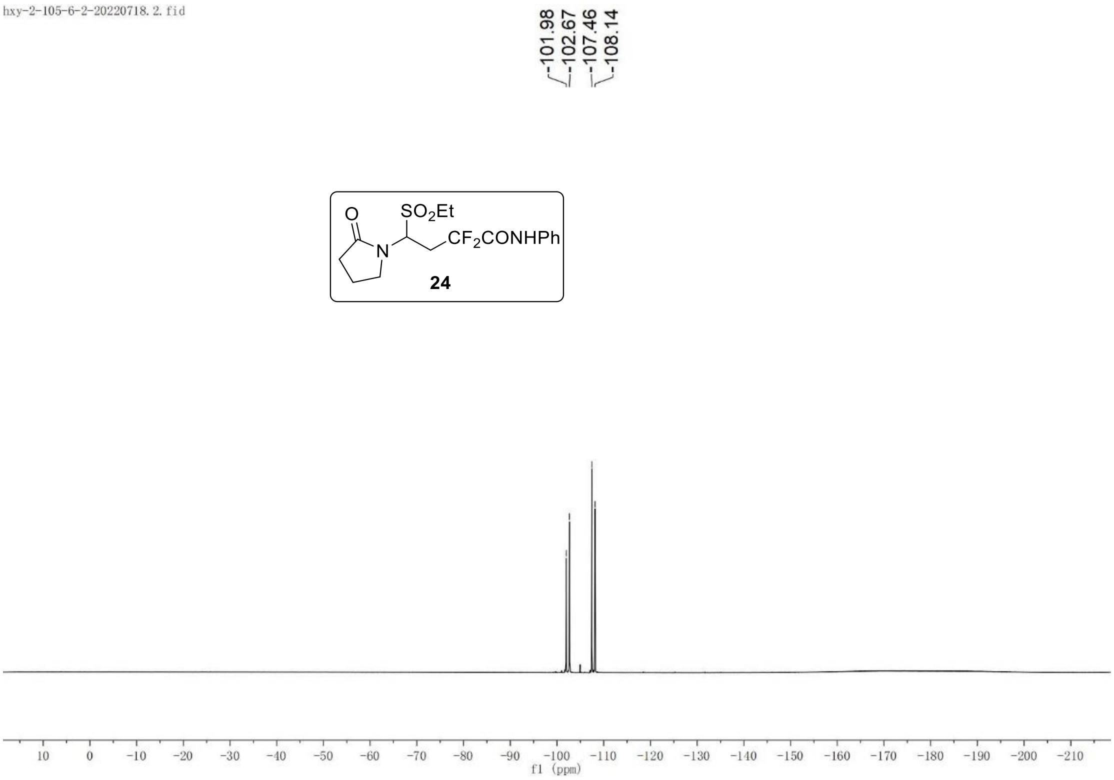

Supplementary Figure 117. ^{19}F NMR (376 MHz, $CDCl_3$) spectra of **24**

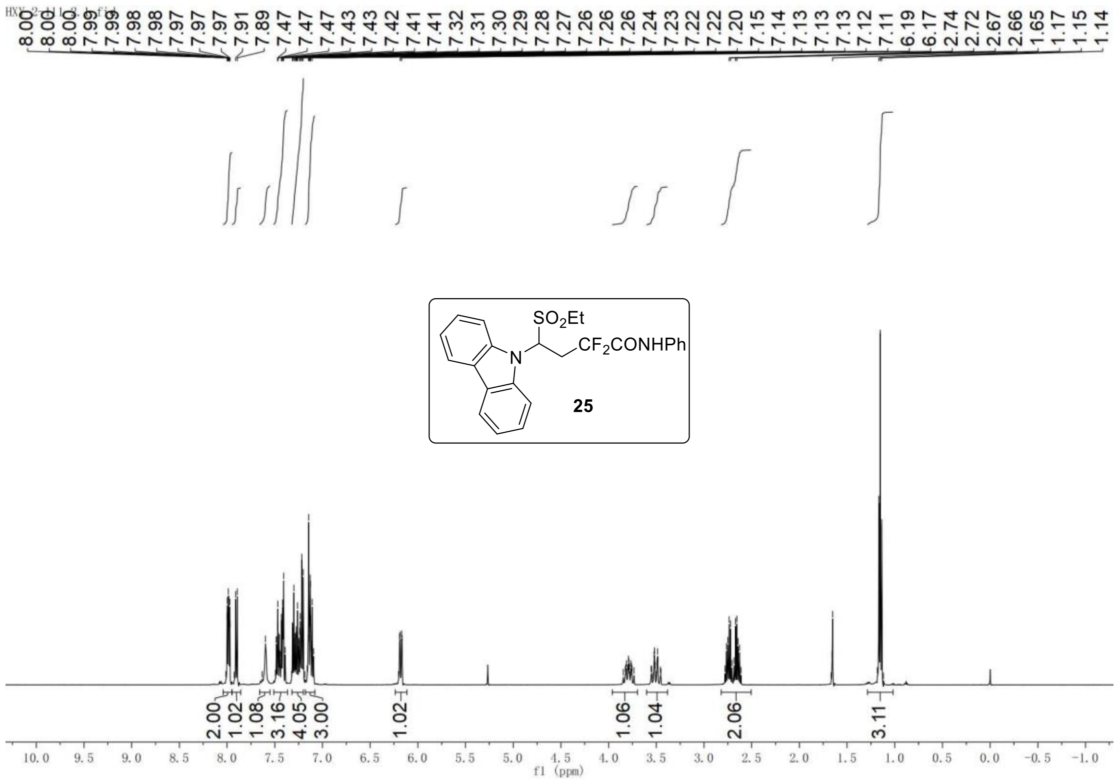

Supplementary Figure 118. ^{1}H NMR (500 MHz, $CDCl_3$) spectra of **25**

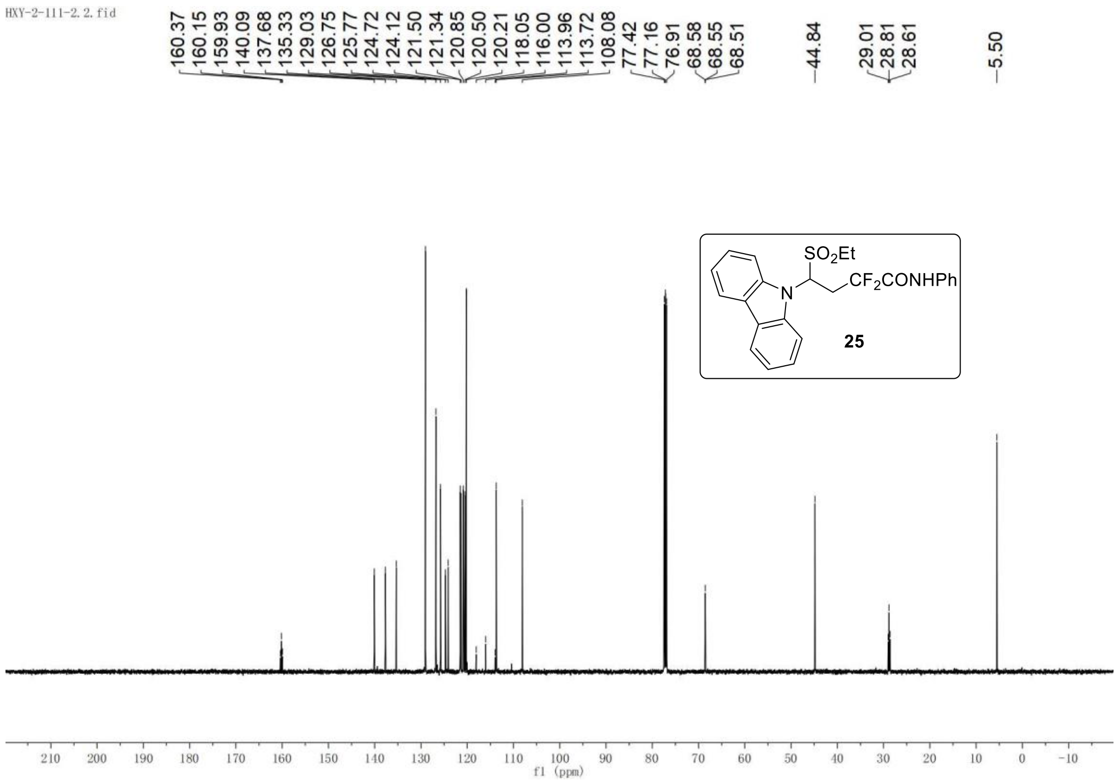

Supplementary Figure 119. ^{13}C NMR (126 MHz, $CDCl_3$) spectra of **25**

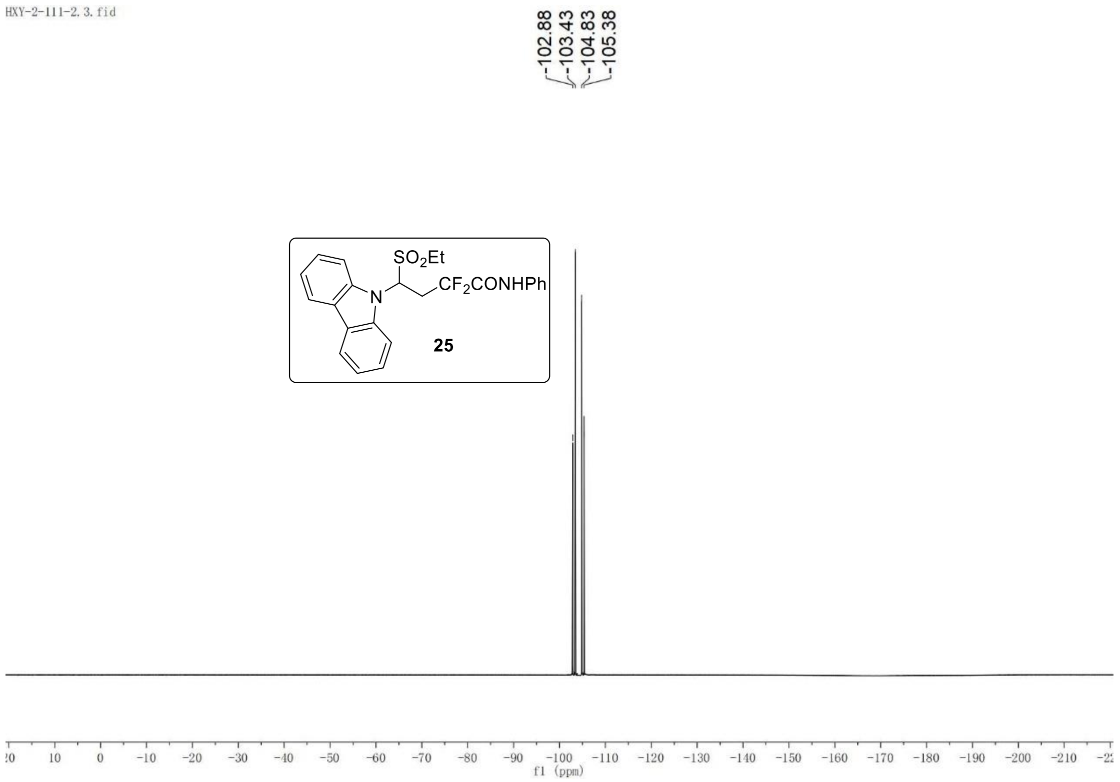

Supplementary Figure 120. ^{19}F NMR (471 MHz, $CDCl_3$) spectra of **25**

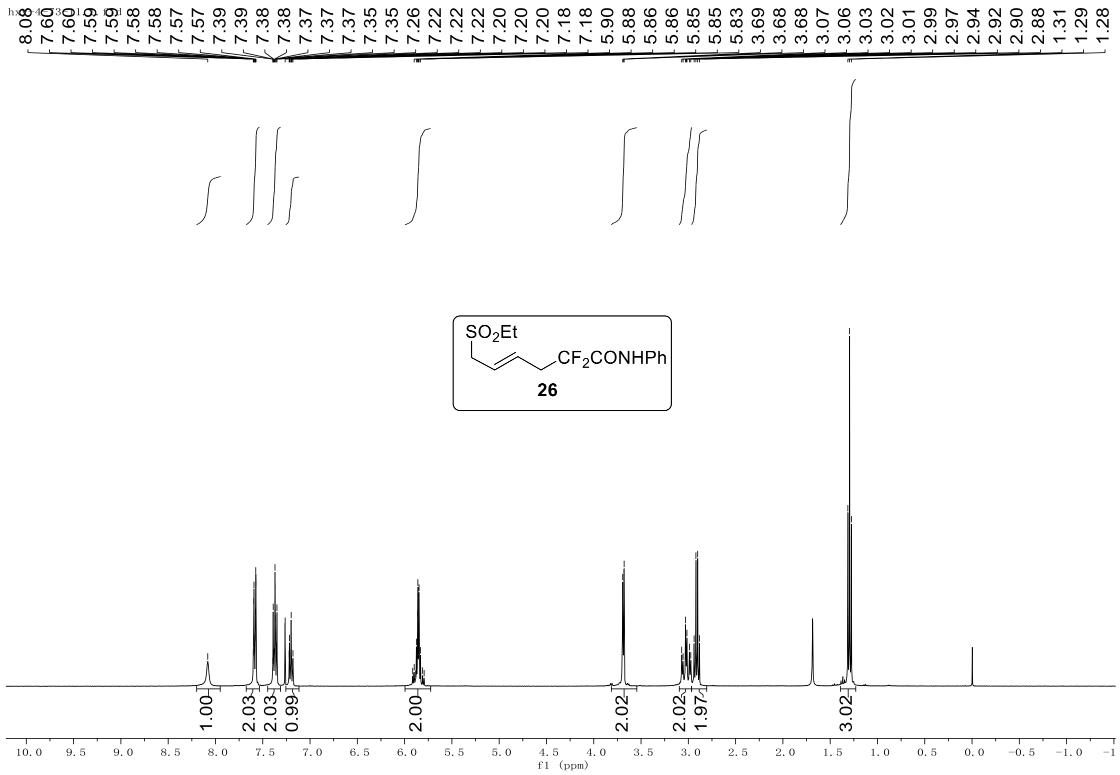

Supplementary Figure 121. ^{1}H NMR (400 MHz, $CDCl_3$) spectra of **26**

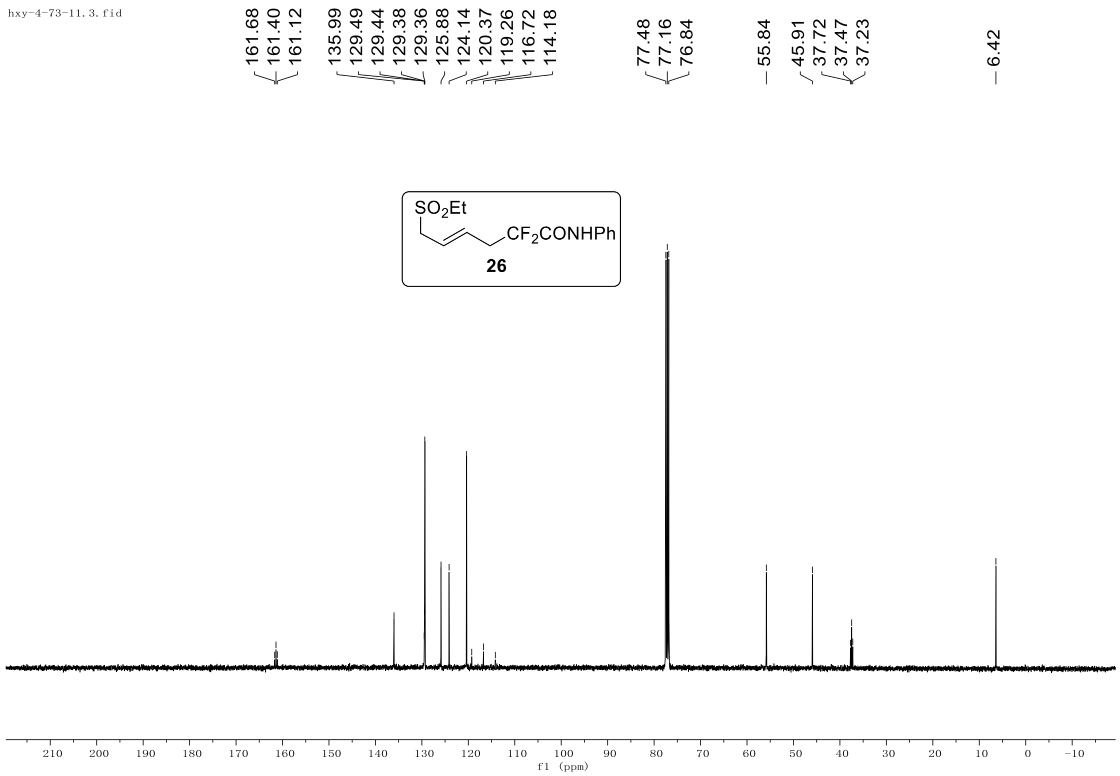

Supplementary Figure 122. ^{13}C NMR (101 MHz, $CDCl_3$) spectra of **26**

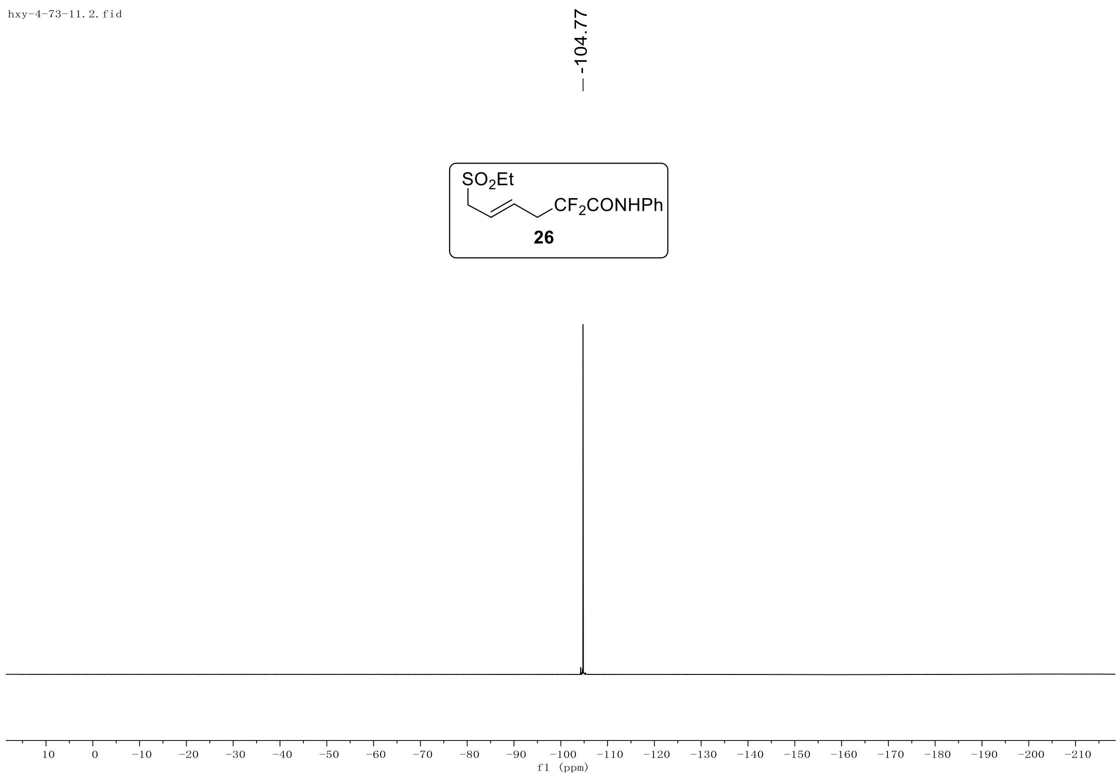

Supplementary Figure 123. ^{19}F NMR (376 MHz, $CDCl_3$) spectra of **26**

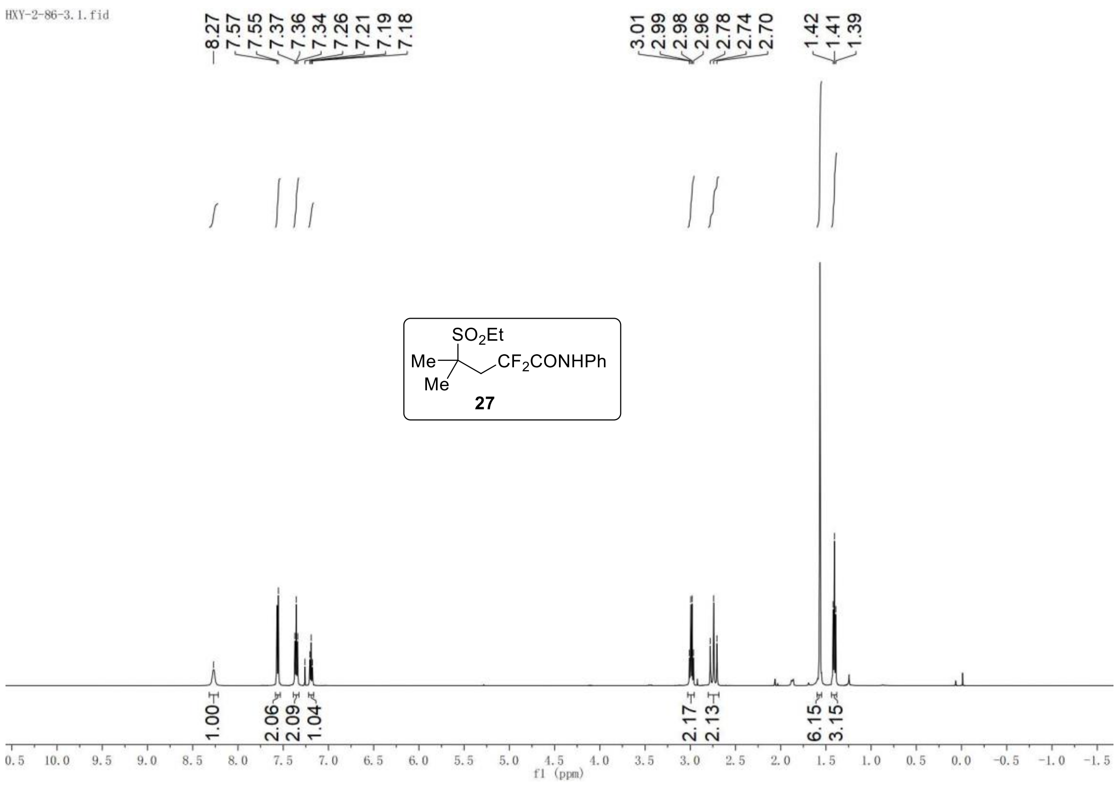

Supplementary Figure 124. ^{1}H NMR (500 MHz, $CDCl_3$) spectra of **27**

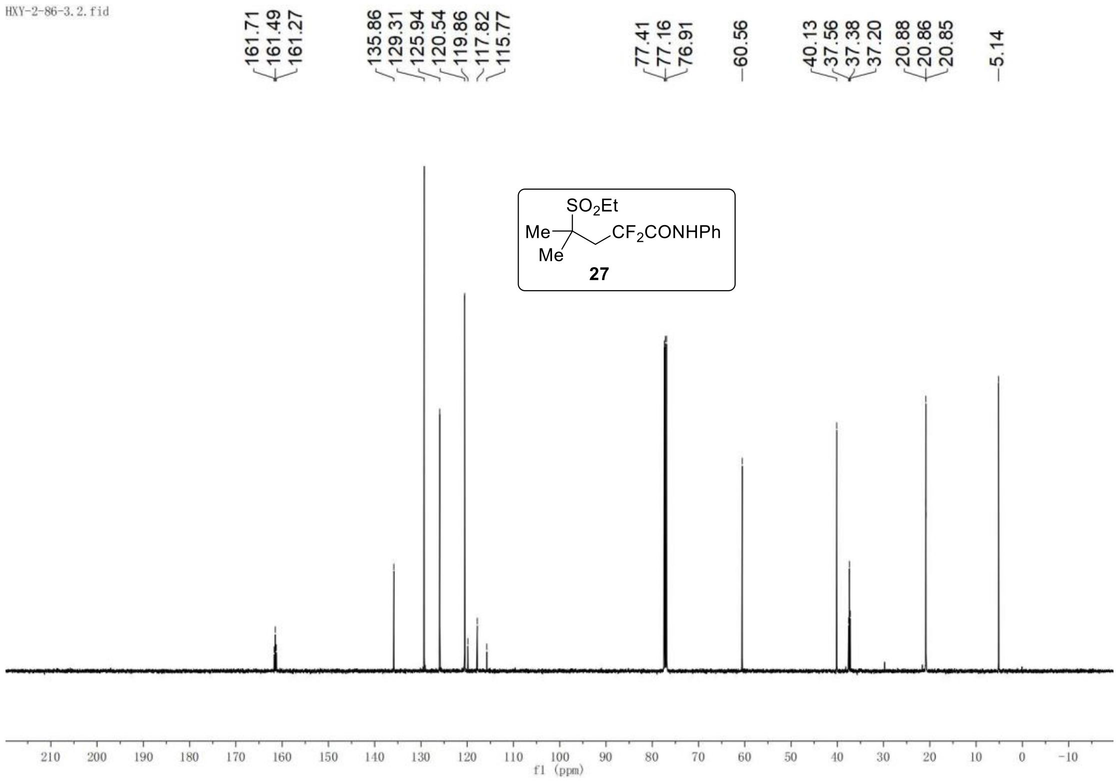

Supplementary Figure 125. ^{13}C NMR (126 MHz, $CDCl_3$) spectra of **27**

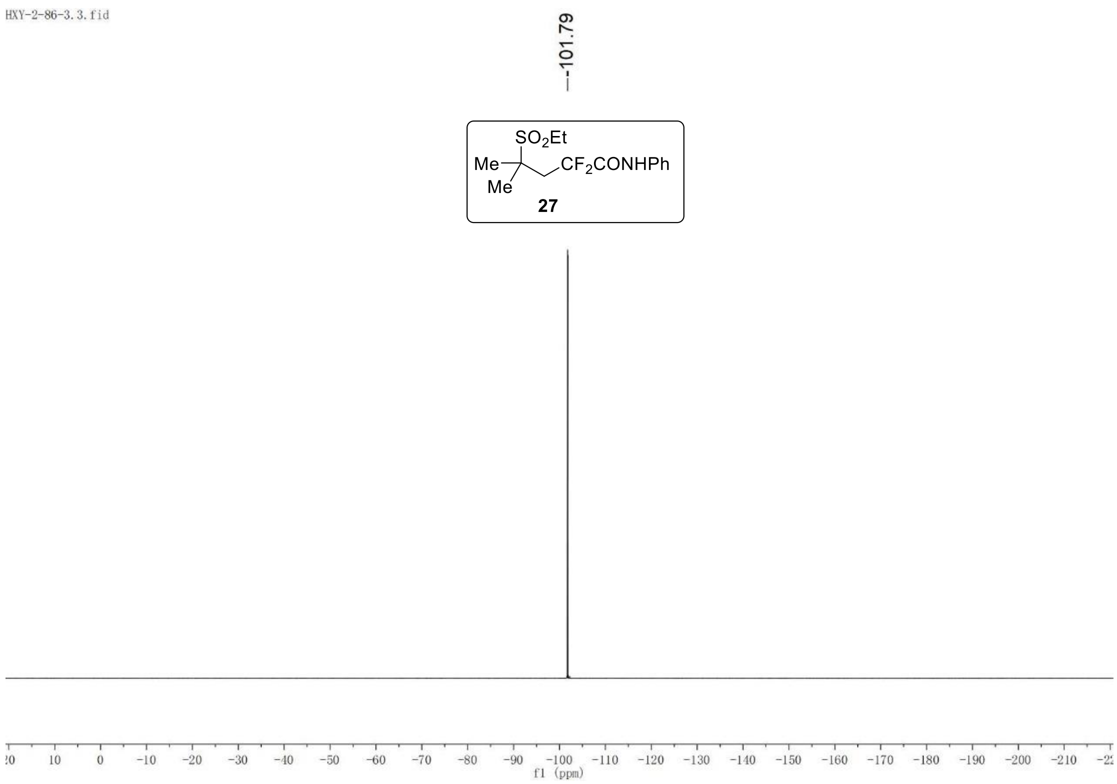

Supplementary Figure 126. ^{19}F NMR (471 MHz, $CDCl_3$) spectra of **27**

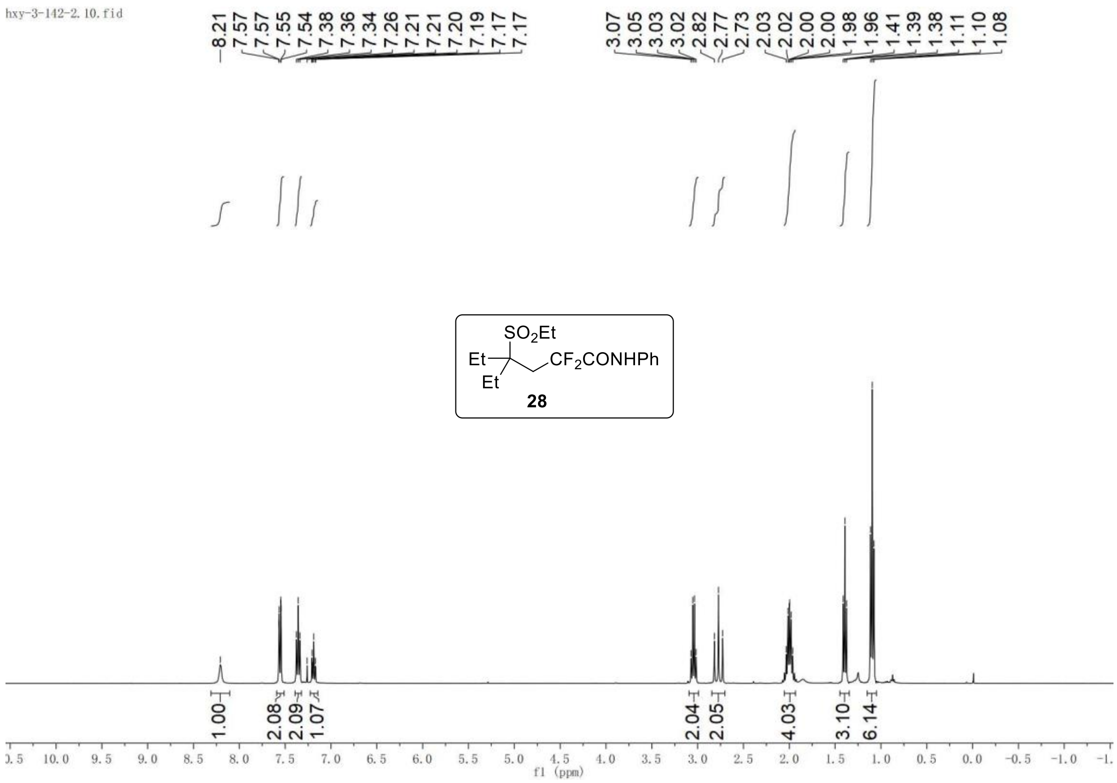

Supplementary Figure 127. ^{1}H NMR (400 MHz, $CDCl_3$) spectra of **28**

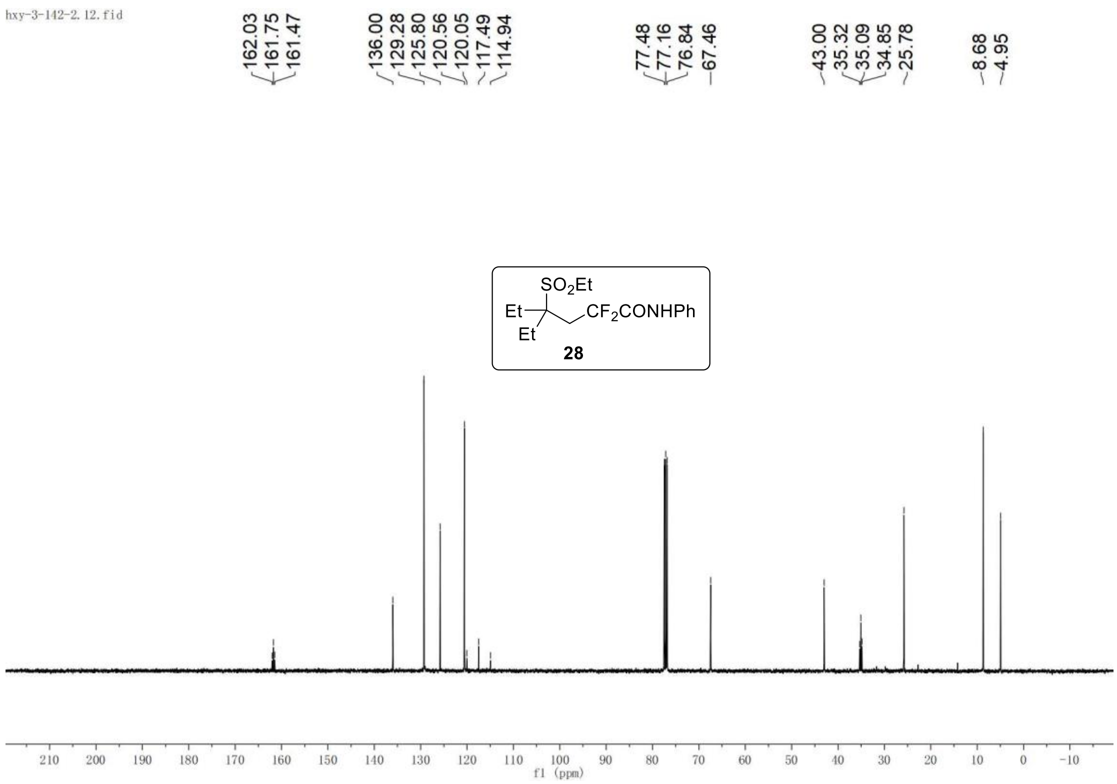

Supplementary Figure 128. ^{13}C NMR (101 MHz, $CDCl_3$) spectra of **28**

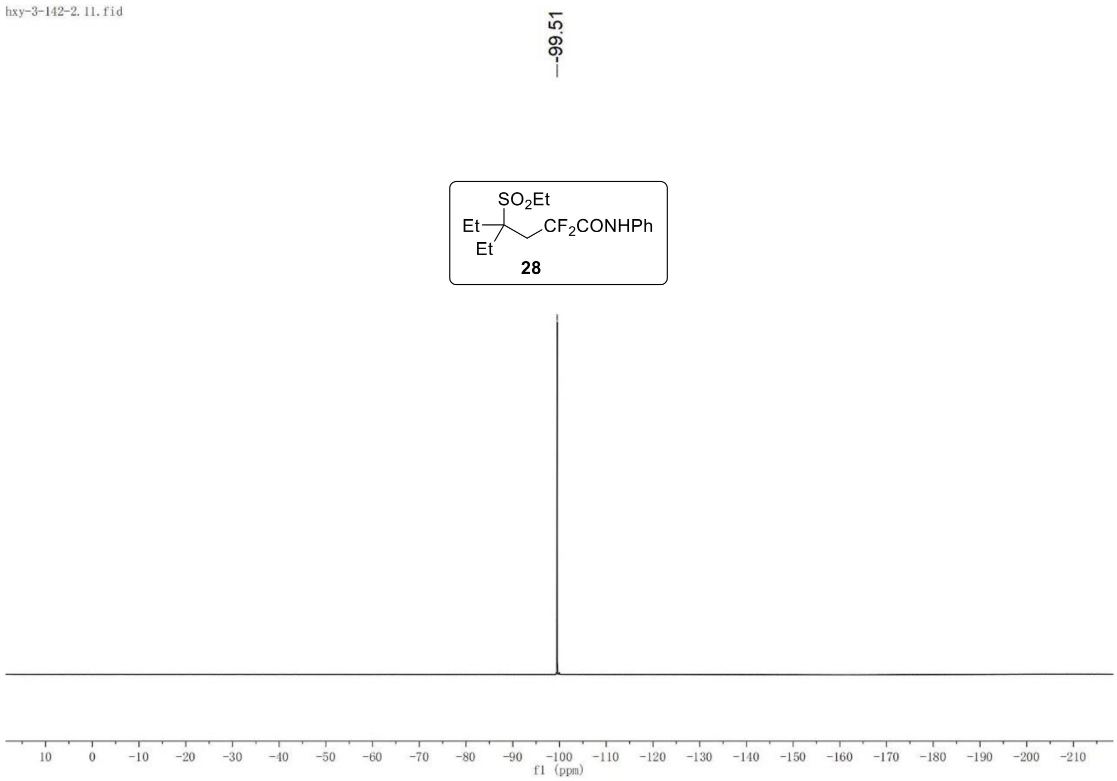

Supplementary Figure 129. ^{19}F NMR (376 MHz, $CDCl_3$) spectra of **28**

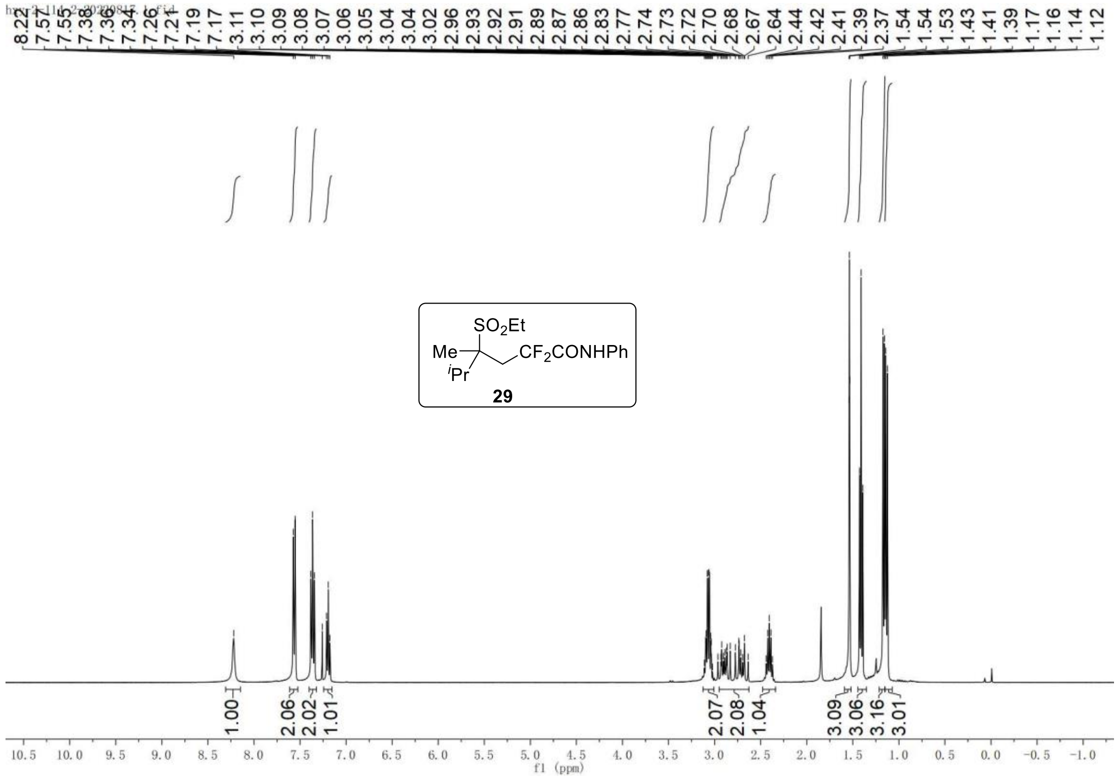

Supplementary Figure 130. ^{1}H NMR (400 MHz, $CDCl_3$) spectra of **29**

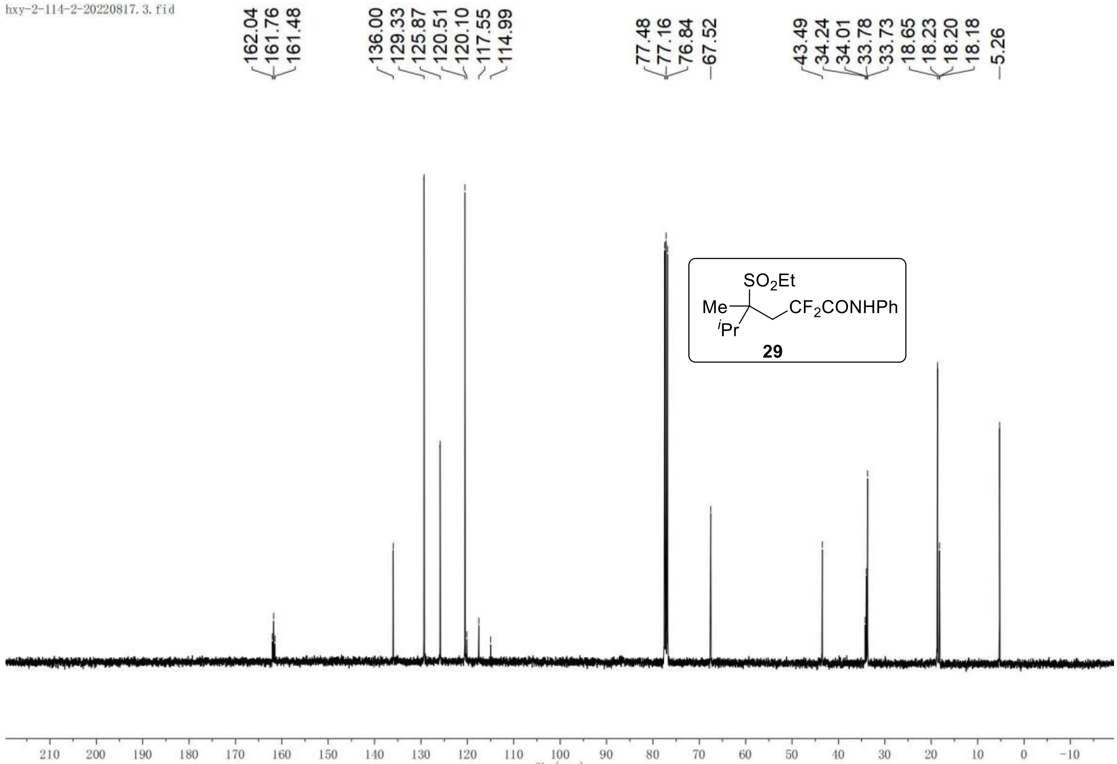

Supplementary Figure 131. ^{13}C NMR (101 MHz, $CDCl_3$) spectra of **29**

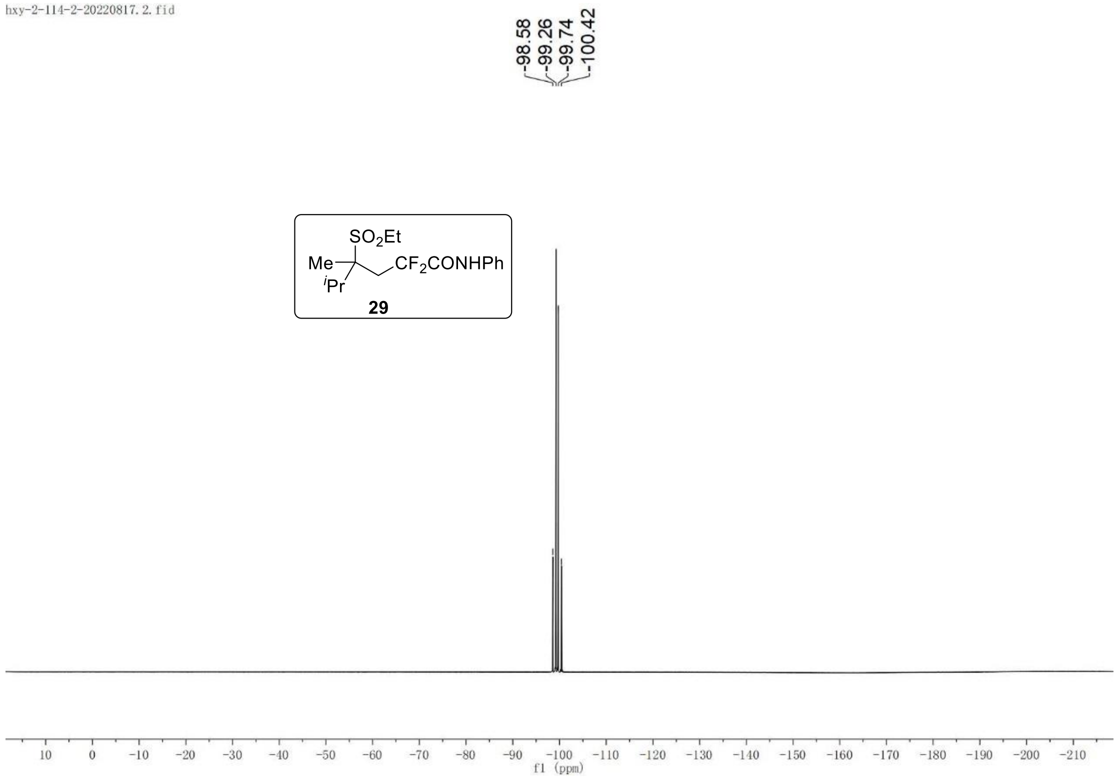

Supplementary Figure 132. ^{19}F NMR (376 MHz, $CDCl_3$) spectra of **29**

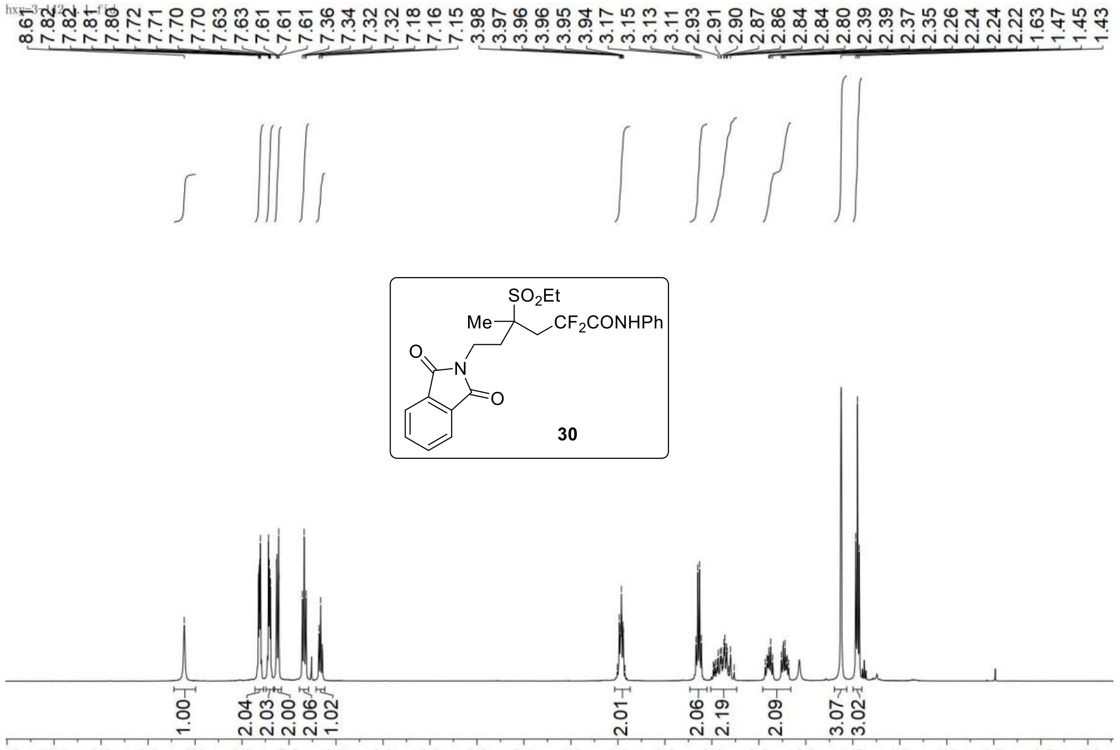

Supplementary Figure 133. ^{1}H NMR (400 MHz, $CDCl_3$) spectra of **30**

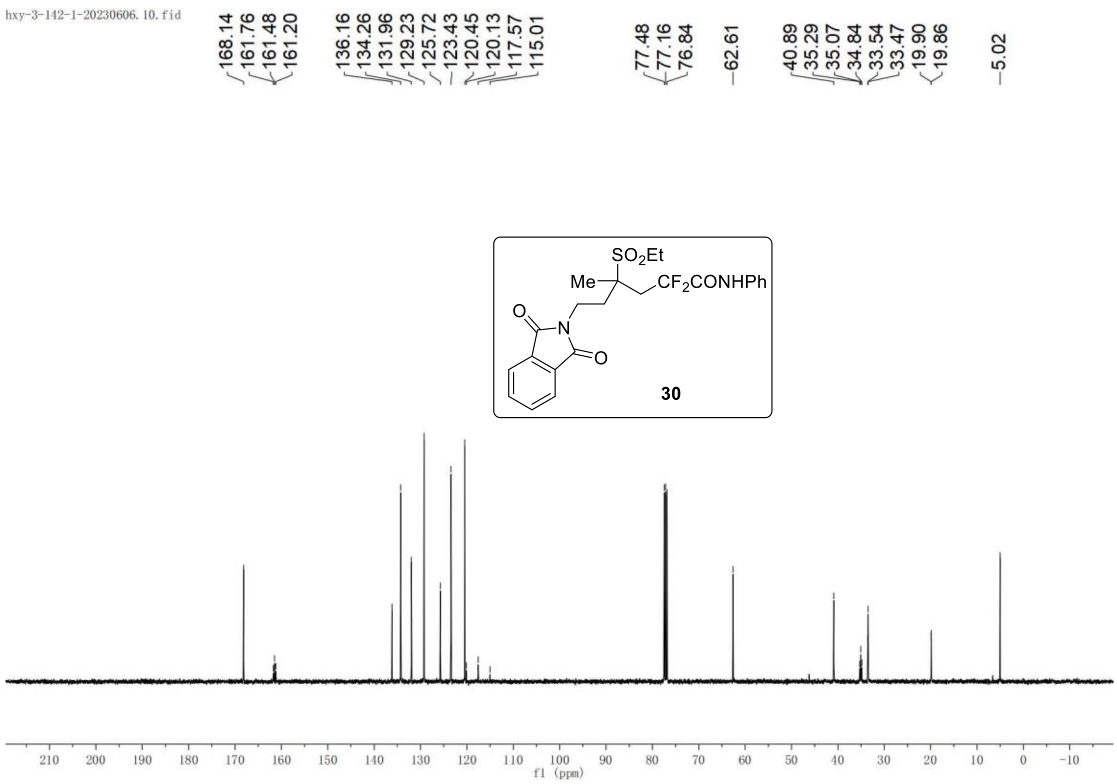

Supplementary Figure 134. ^{13}C NMR (101 MHz, $CDCl_3$) spectra of **30**

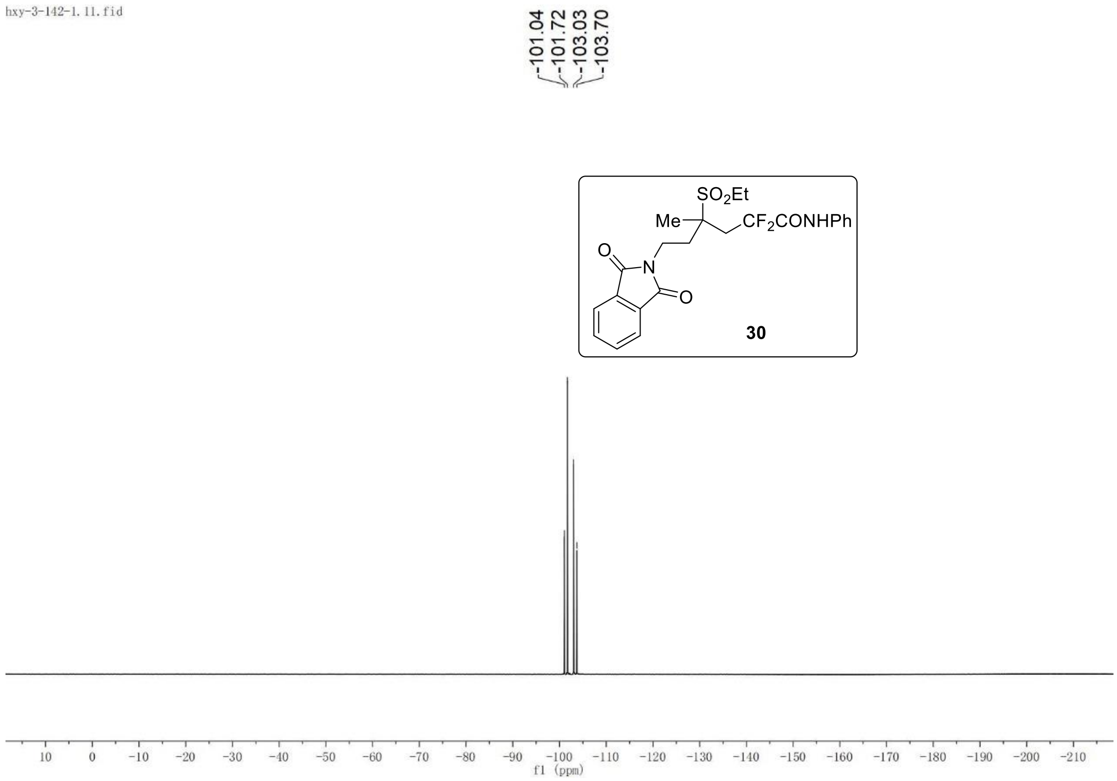

Supplementary Figure 135. ^{19}F NMR (376 MHz, $CDCl_3$) spectra of **30**

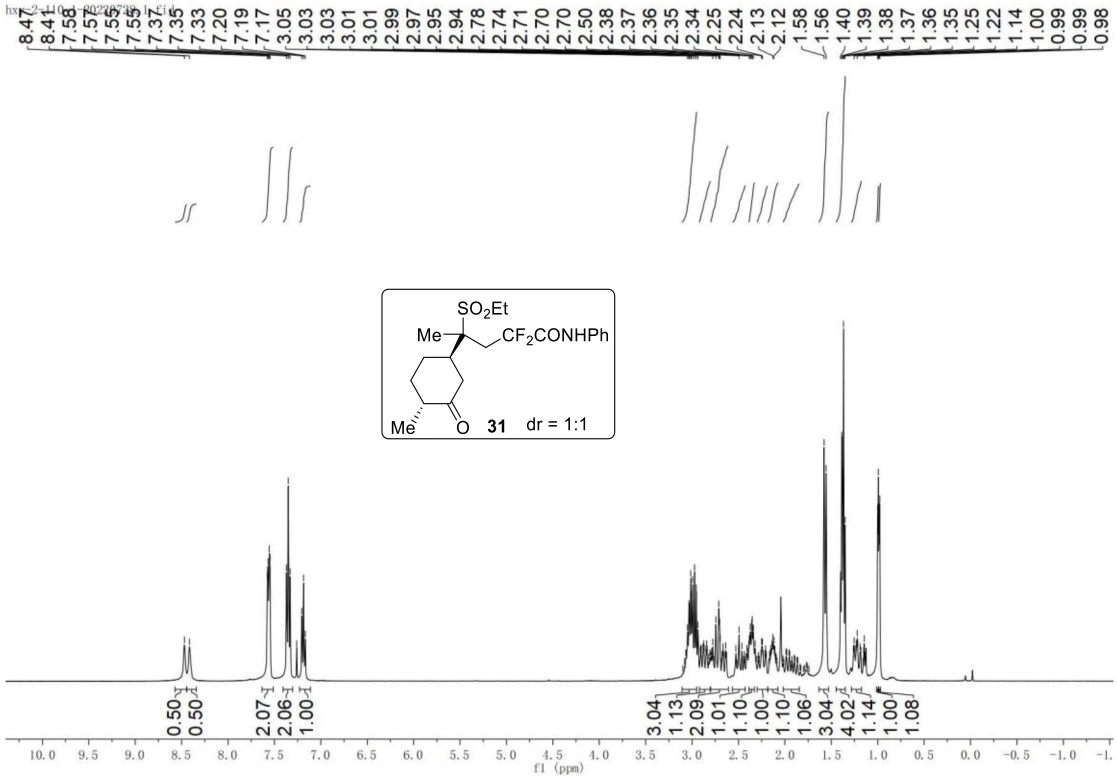

Supplementary Figure 136. ^{1}H NMR (400 MHz, $CDCl_3$) spectra of **31**

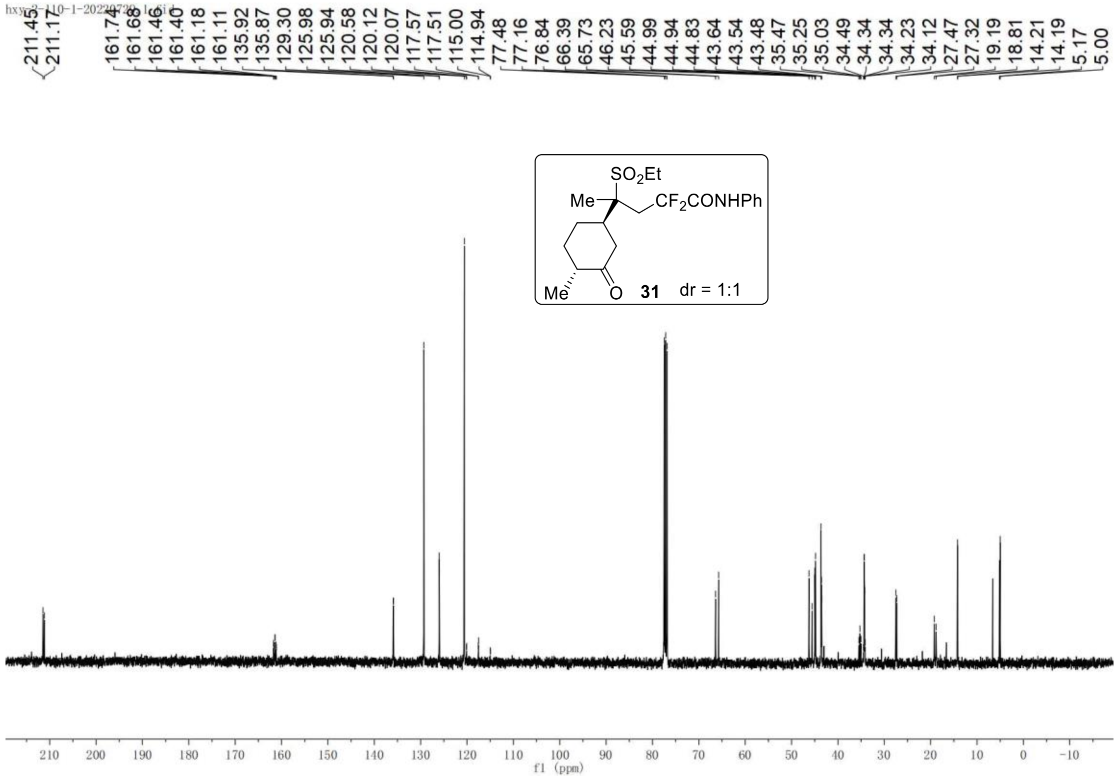

Supplementary Figure 137. ^{13}C NMR (101 MHz, $CDCl_3$) spectra of **31**

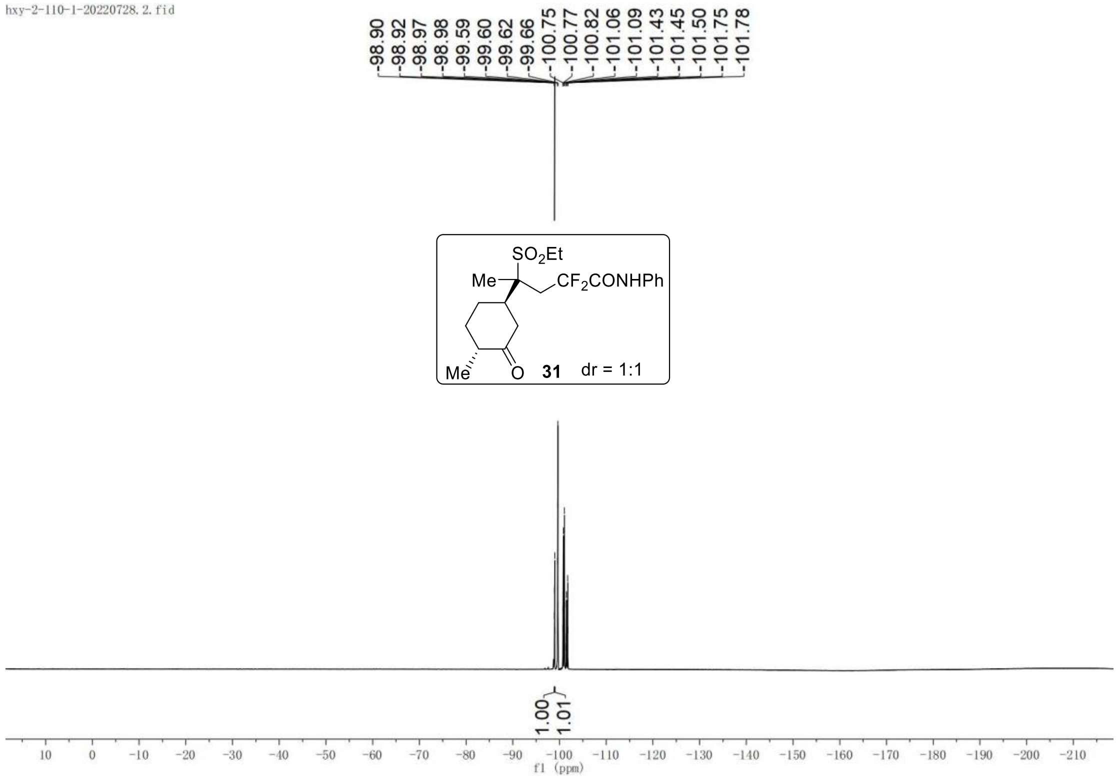

Supplementary Figure 138. ^{19}F NMR (376 MHz, $CDCl_3$) spectra of **31**

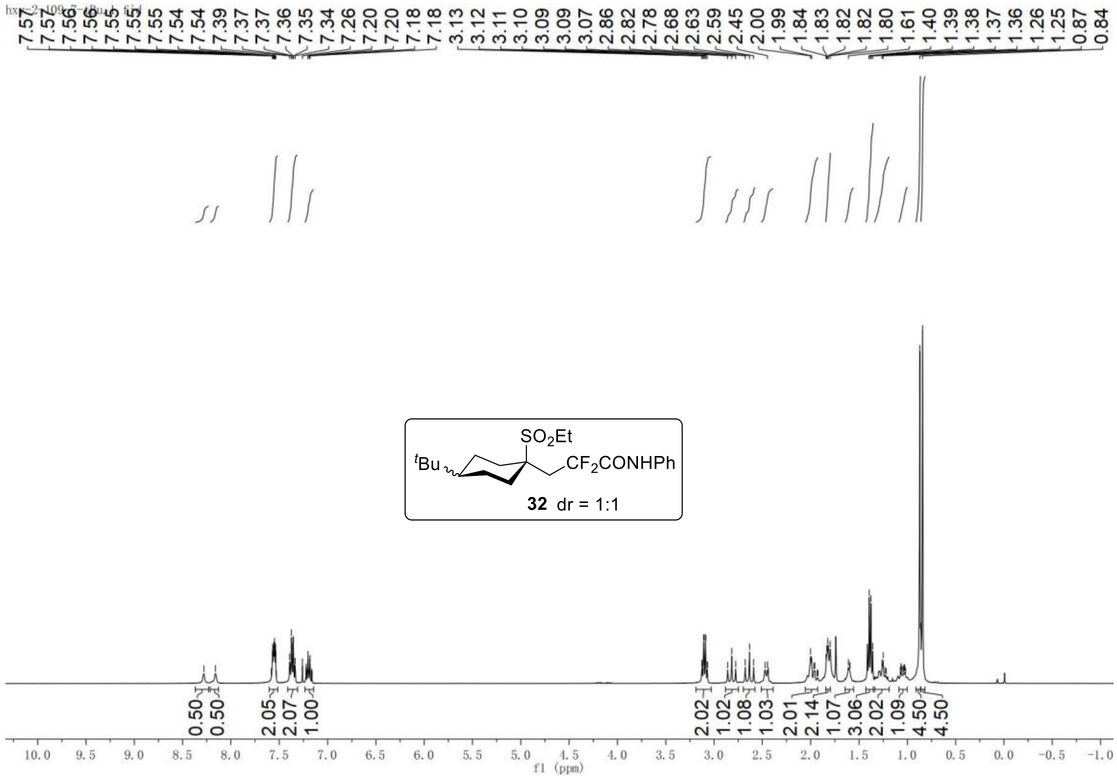

Supplementary Figure 139. ^{1}H NMR (400 MHz, $CDCl_3$) spectra of **32**

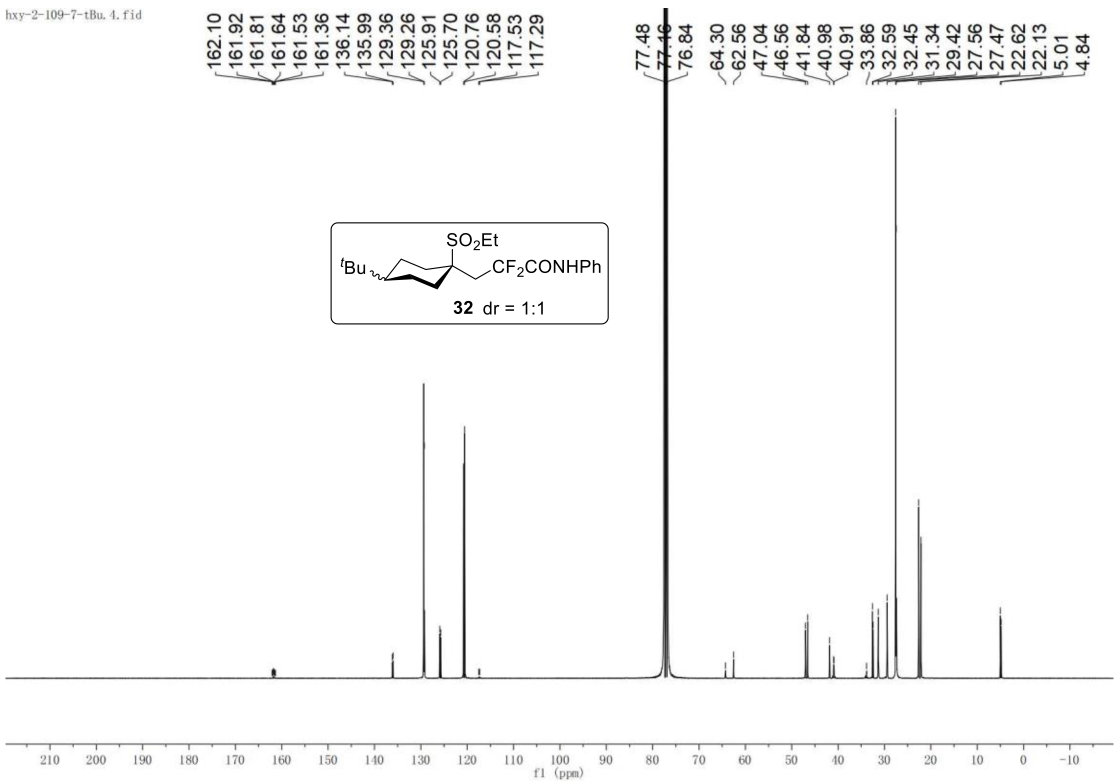

Supplementary Figure 140. ^{13}C NMR (101 MHz, $CDCl_3$) spectra of **32**

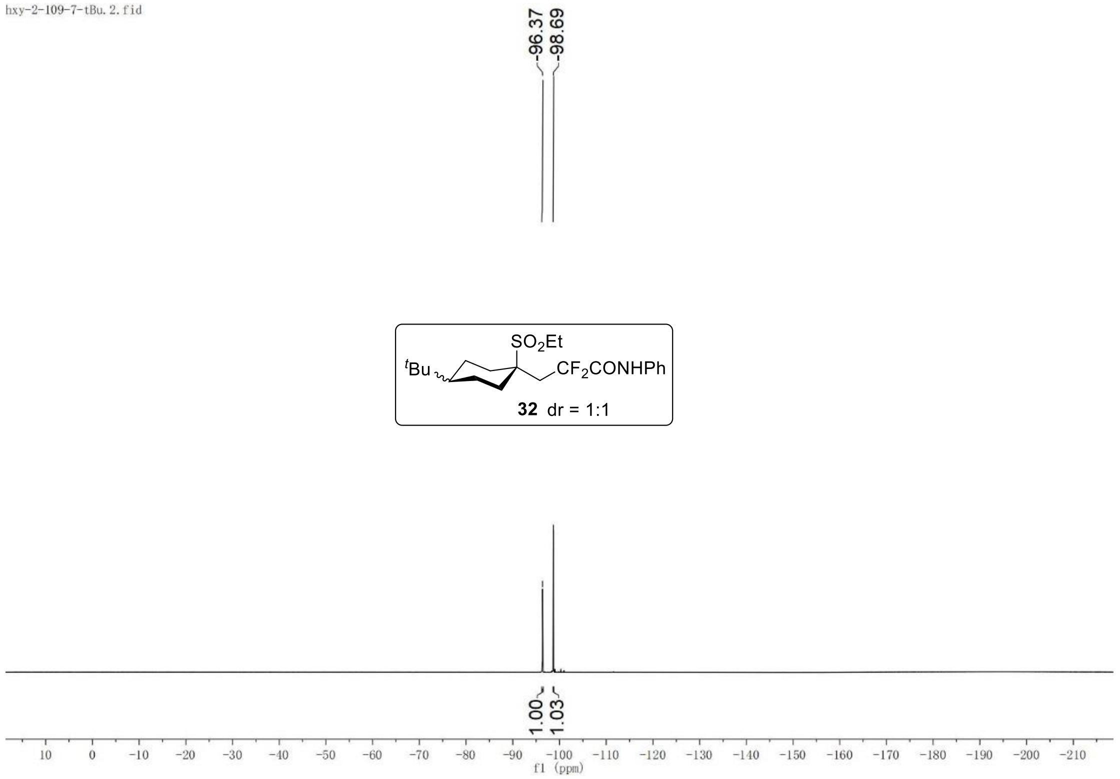

Supplementary Figure 141. ^{19}F NMR (376 MHz, $CDCl_3$) spectra of **32**

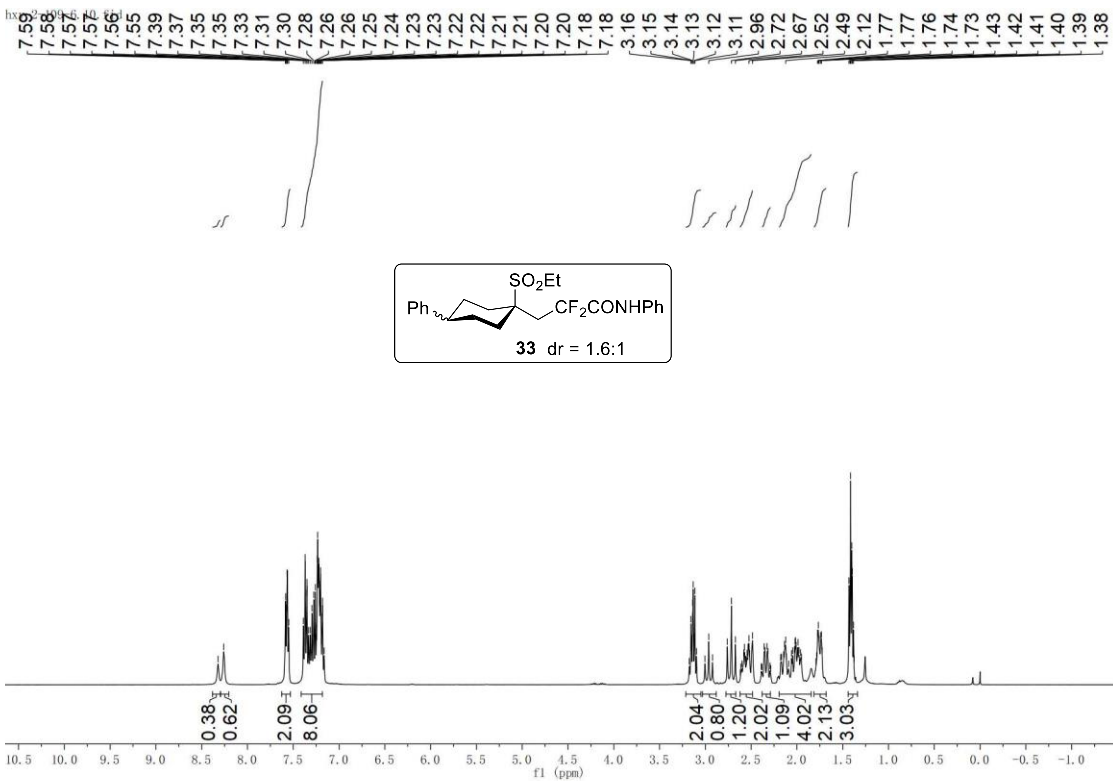

Supplementary Figure 142. ^{1}H NMR (400 MHz, $CDCl_3$) spectra of **33**

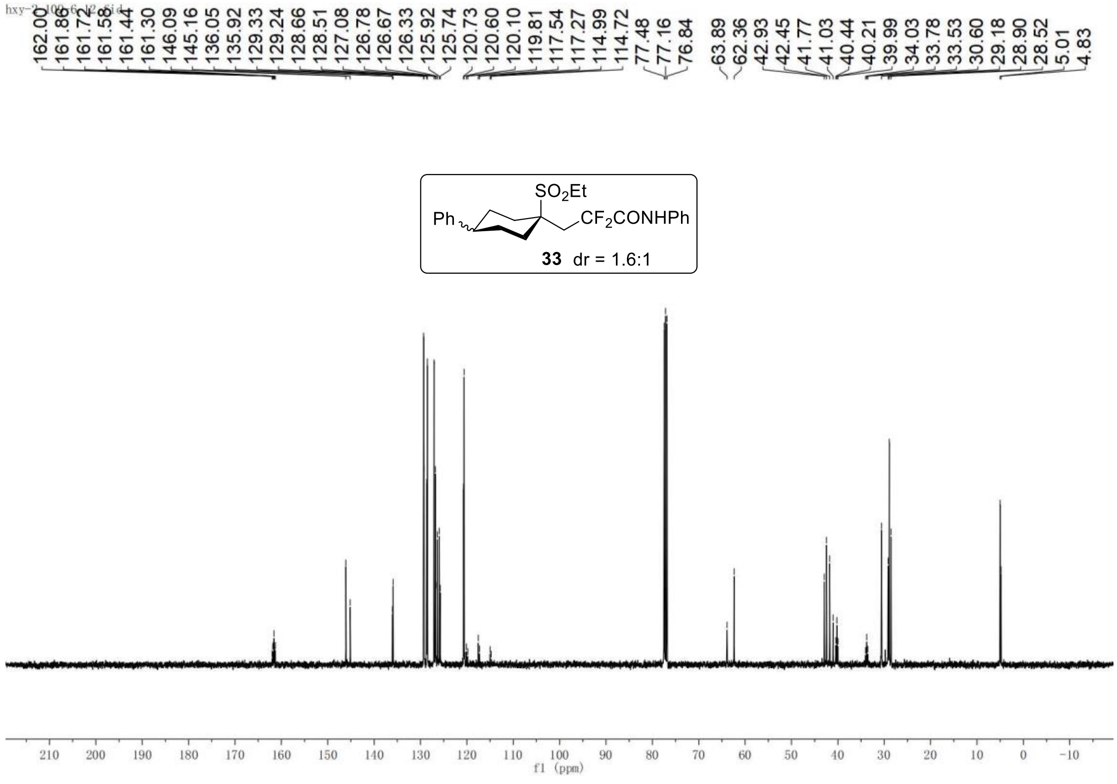

Supplementary Figure 143. ^{13}C NMR (101 MHz, $CDCl_3$) spectra of **33**

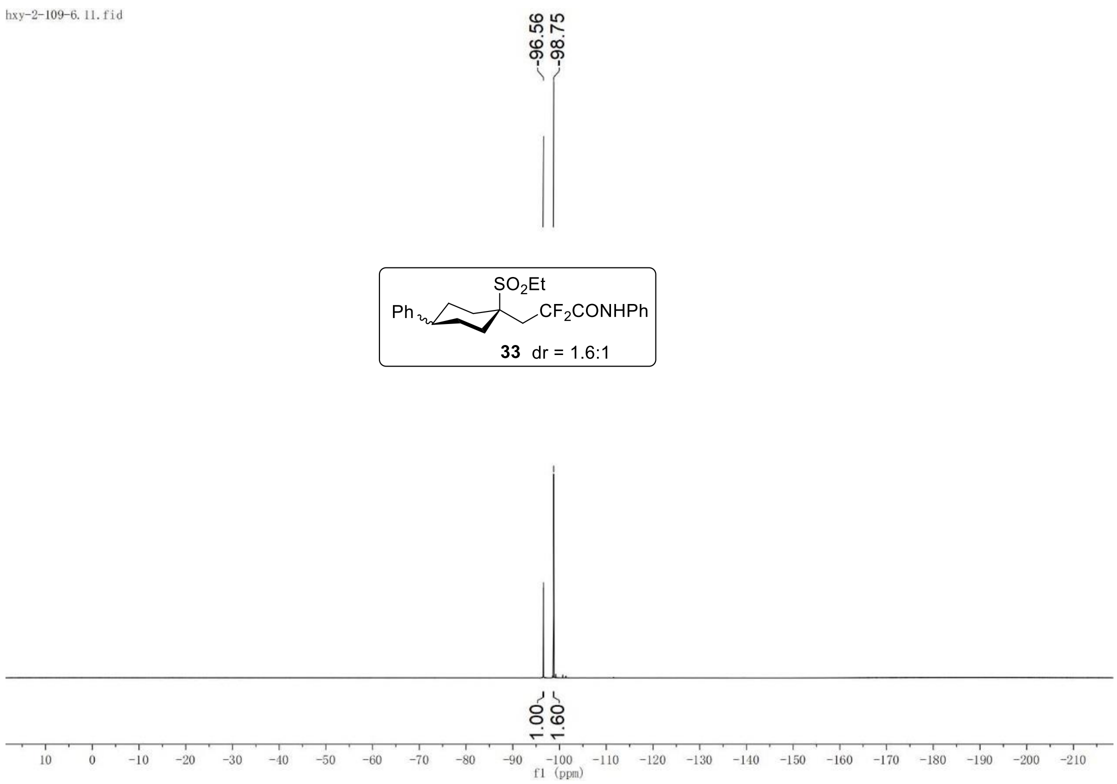

Supplementary Figure 144. ^{19}F NMR (376 MHz, $CDCl_3$) spectra of **33**

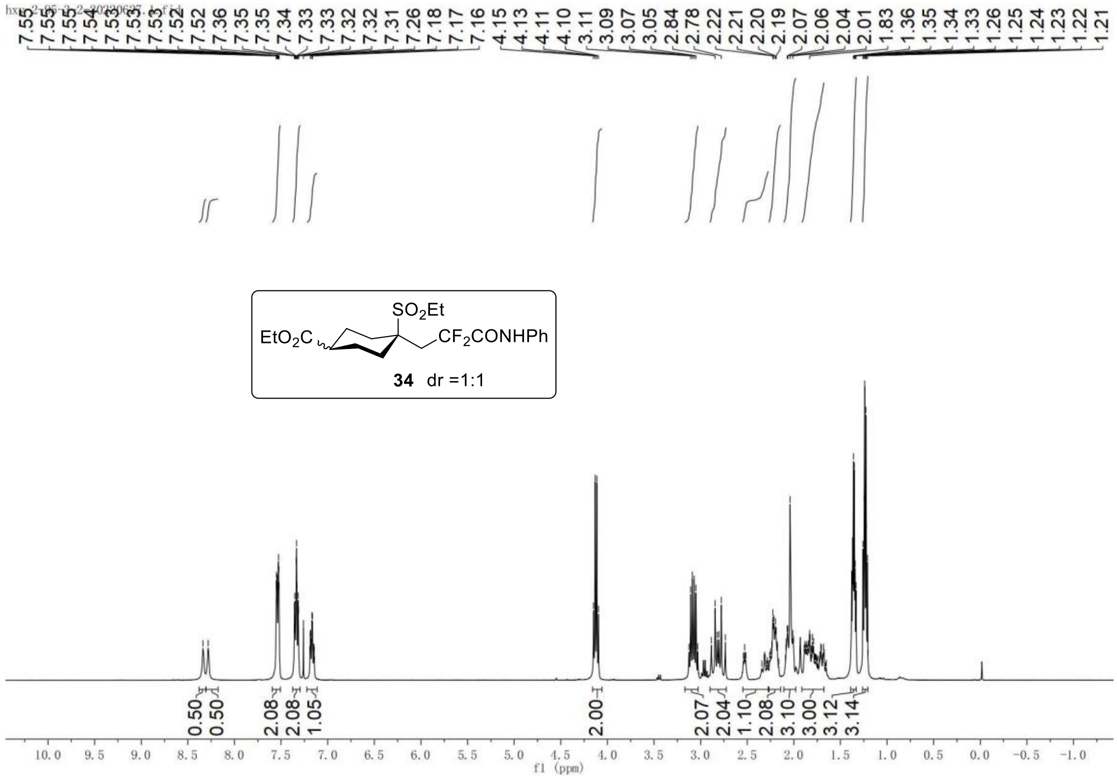

Supplementary Figure 145. ^{1}H NMR (400 MHz, $CDCl_3$) spectra of **34**

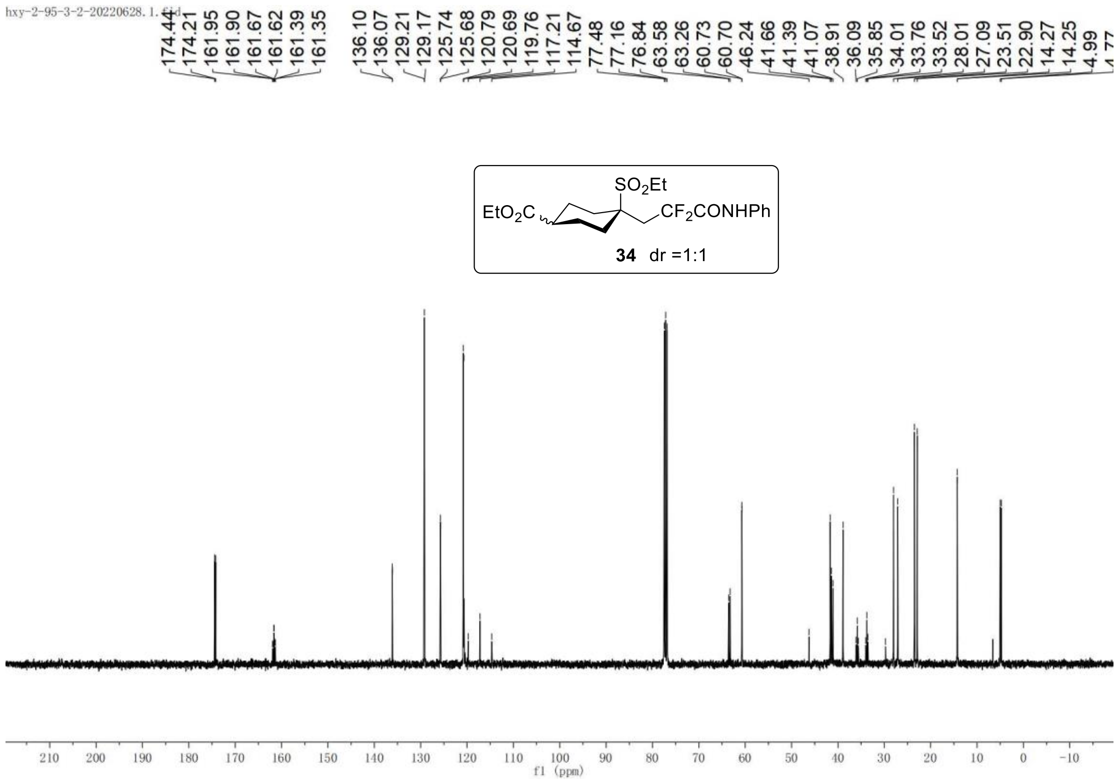

Supplementary Figure 146. ^{13}C NMR (101 MHz, $CDCl_3$) spectra of **34**

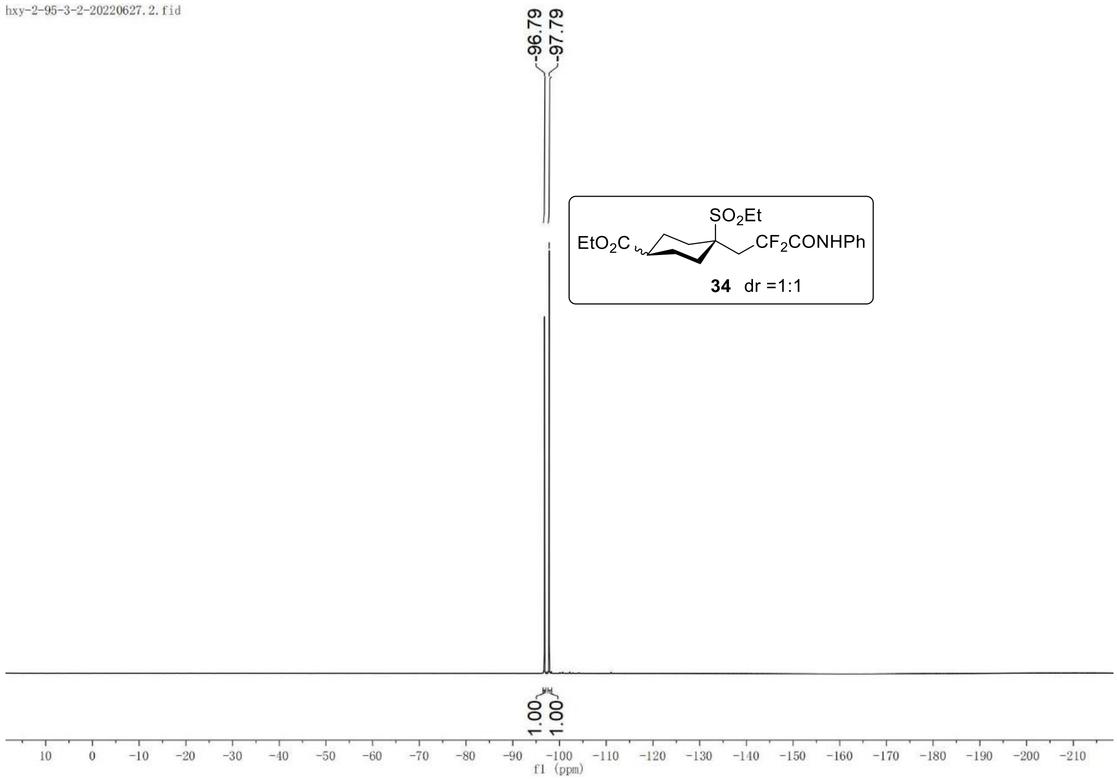

Supplementary Figure 147. ^{19}F NMR (376 MHz, $CDCl_3$) spectra of **34**

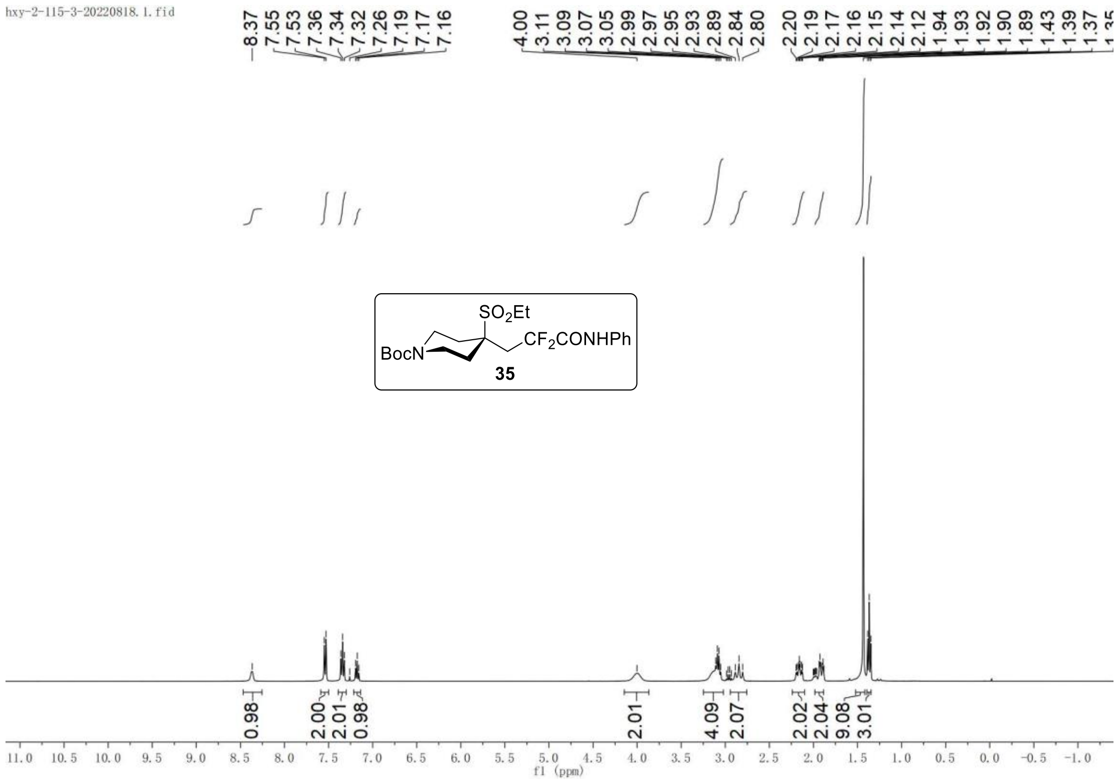

Supplementary Figure 148. ^{1}H NMR (400 MHz, $CDCl_3$) spectra of **35**

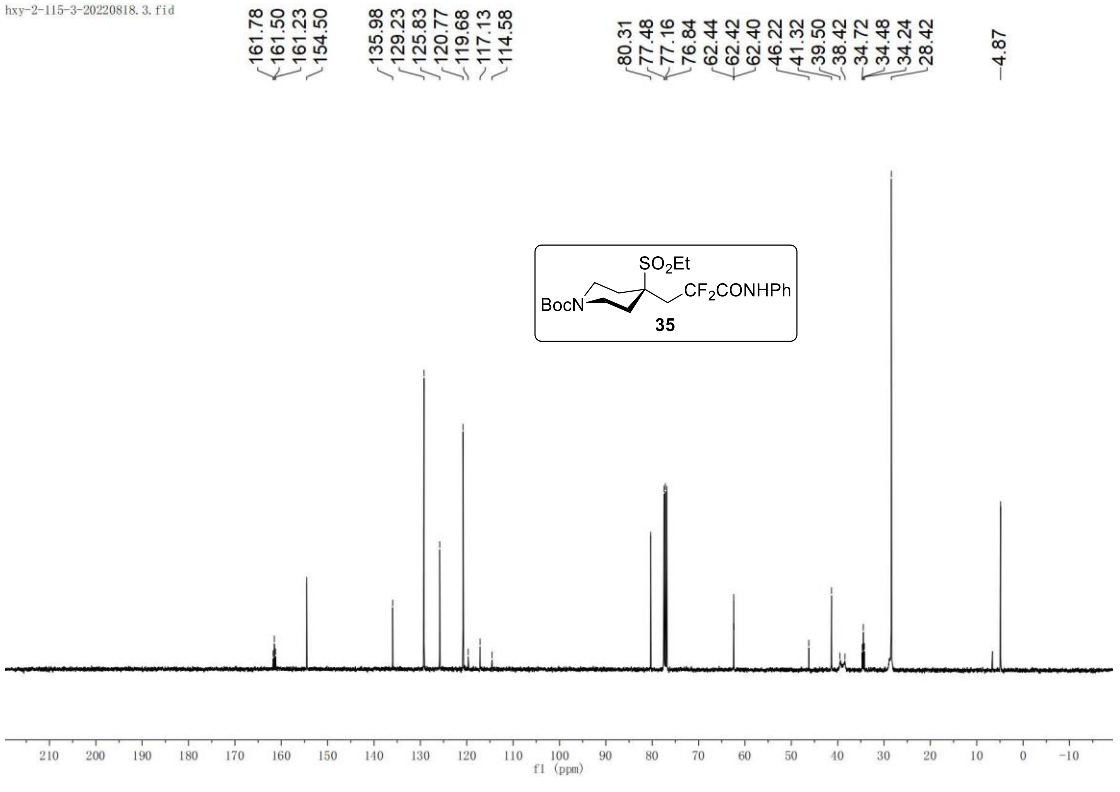

Supplementary Figure 149. ^{13}C NMR (101 MHz, $CDCl_3$) spectra of **35**

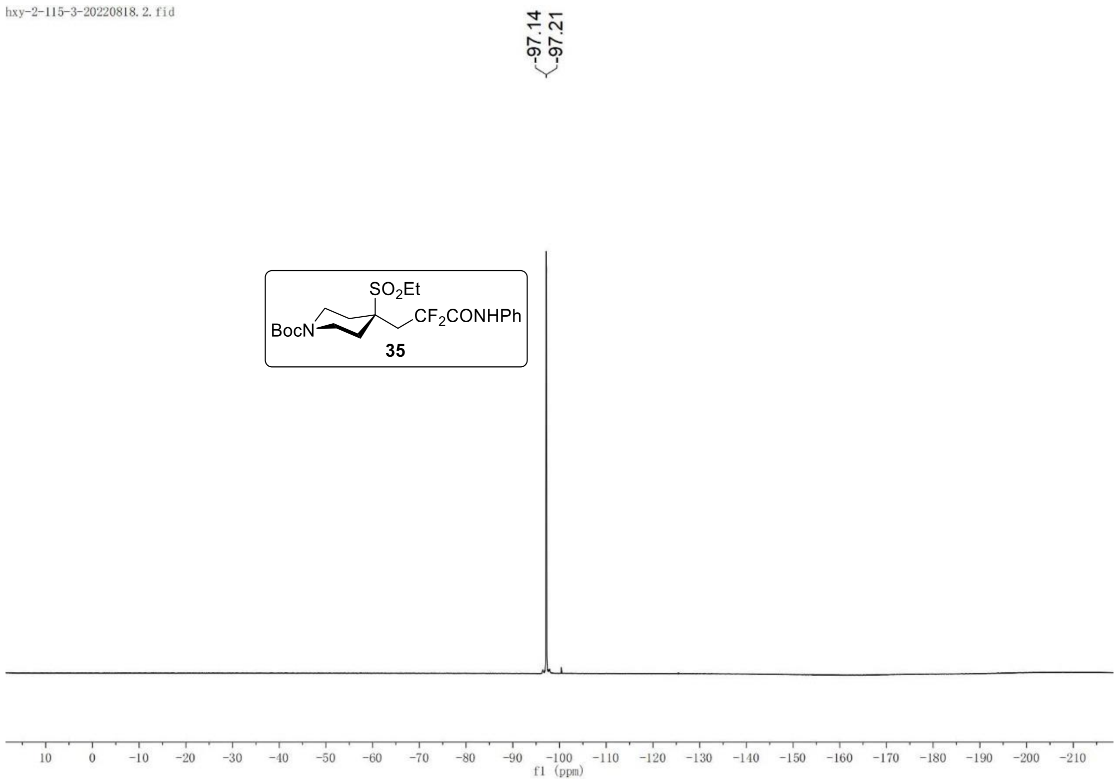

Supplementary Figure 150. ^{19}F NMR (376 MHz, $CDCl_3$) spectra of **35**

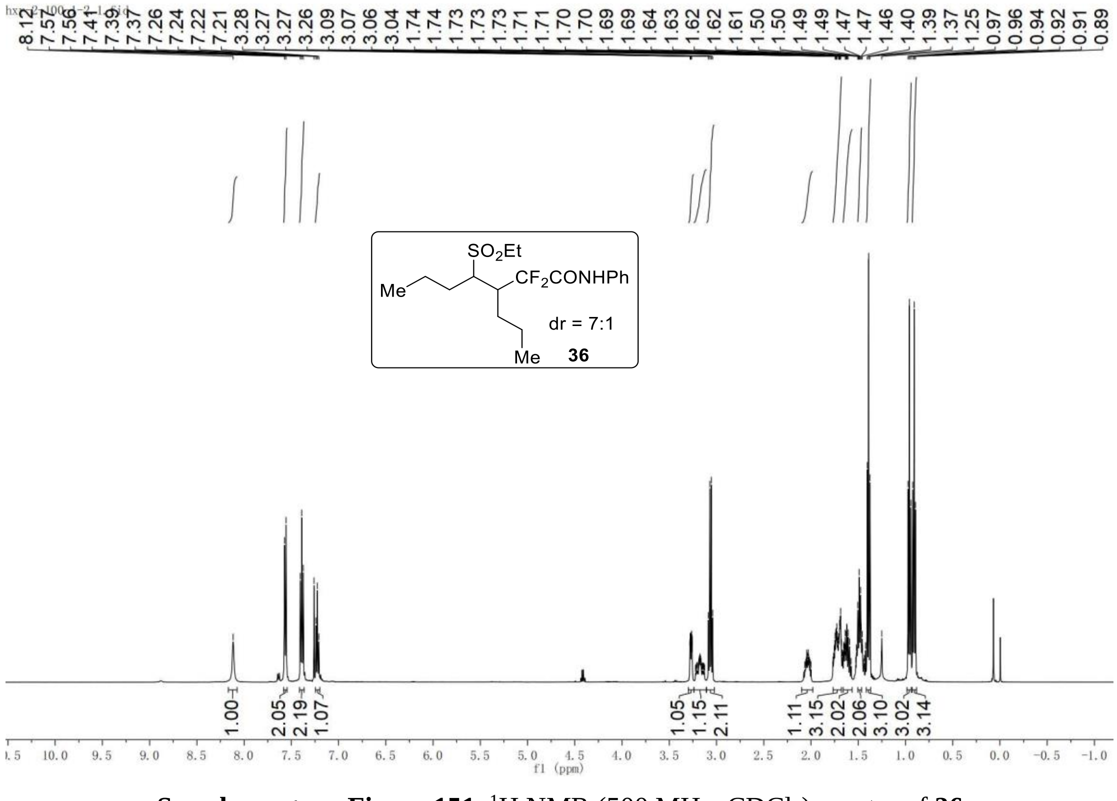

Supplementary Figure 151. ^{1}H NMR (500 MHz, $CDCl_3$) spectra of **36**

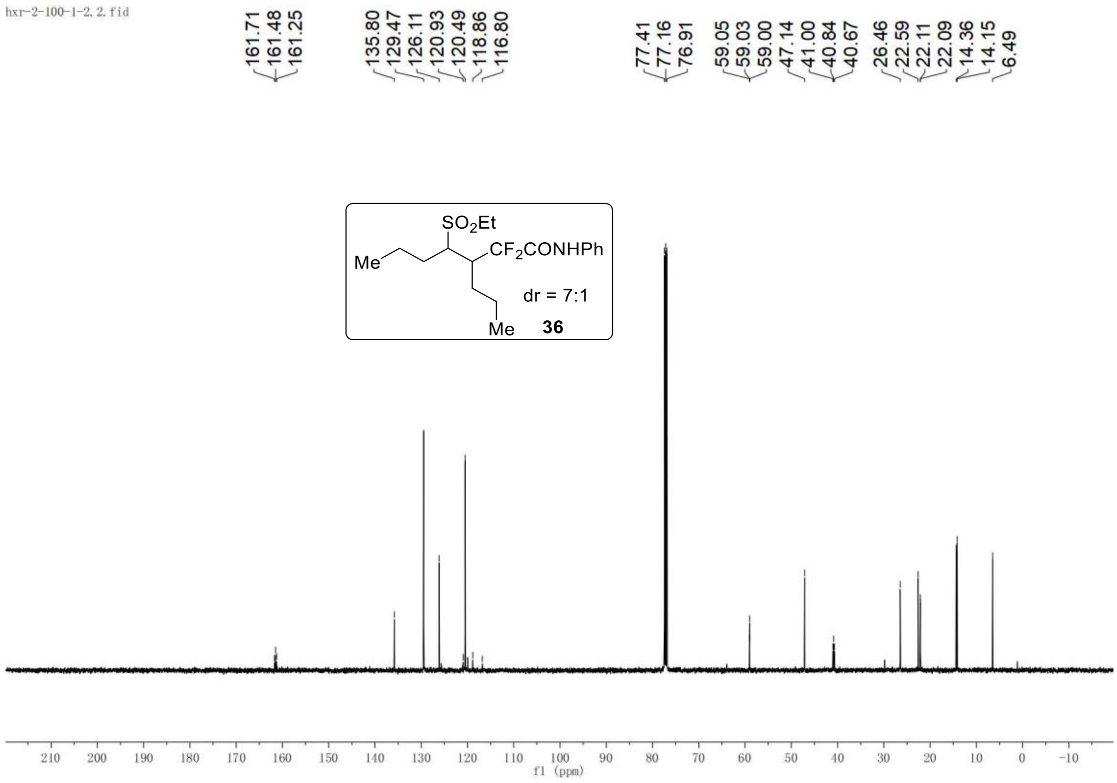

Supplementary Figure 152. ^{13}C NMR (126MHz, $CDCl_3$) spectra of **36**

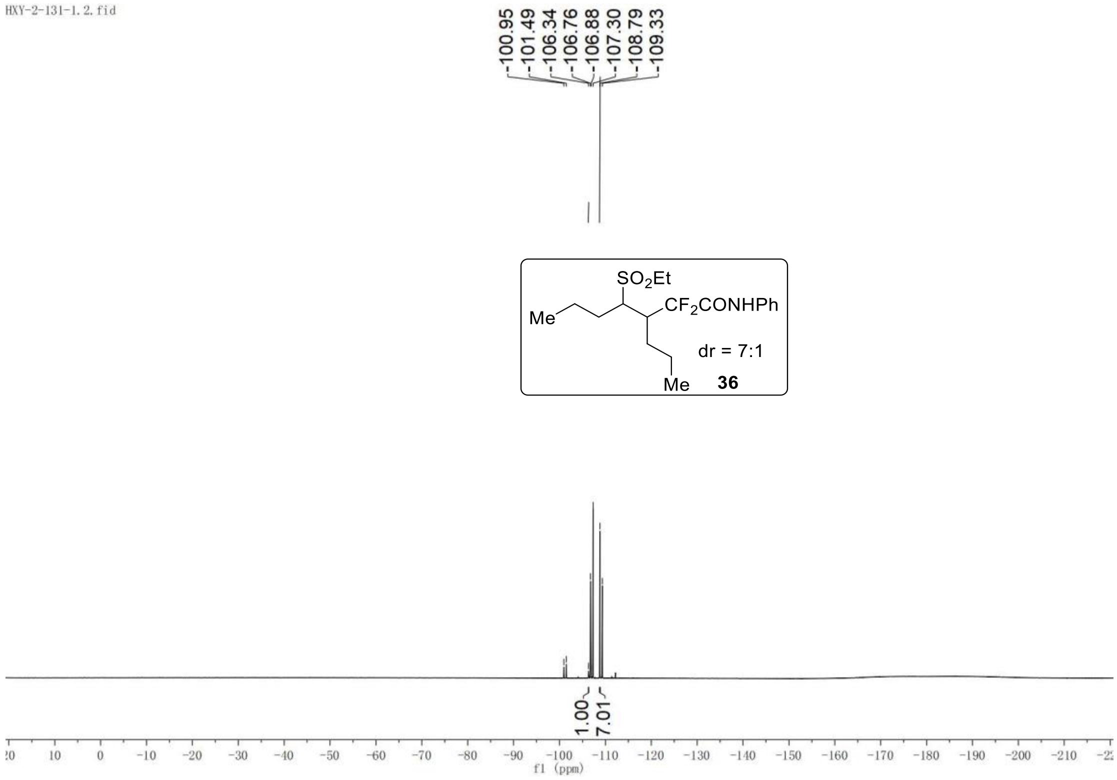

Supplementary Figure 153. ^{19}F NMR (471 MHz, $CDCl_3$) spectra of **36**

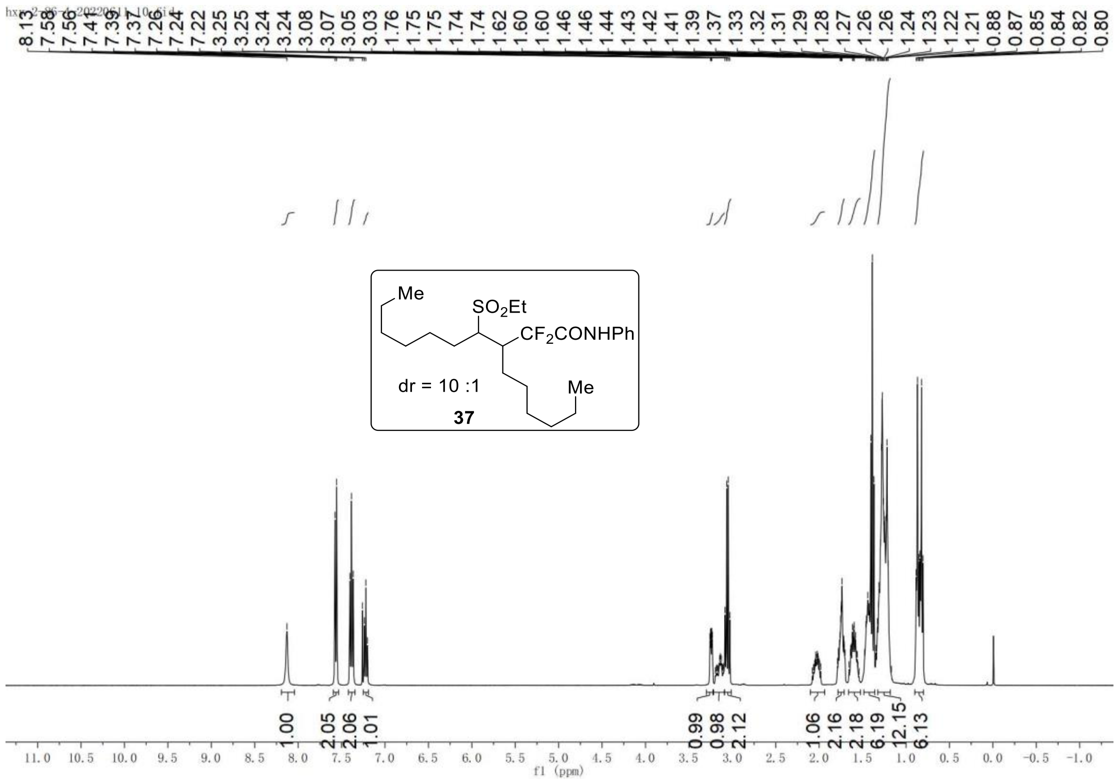

Supplementary Figure 154. ^{1}H NMR (400 MHz, $CDCl_3$) spectra of **37**

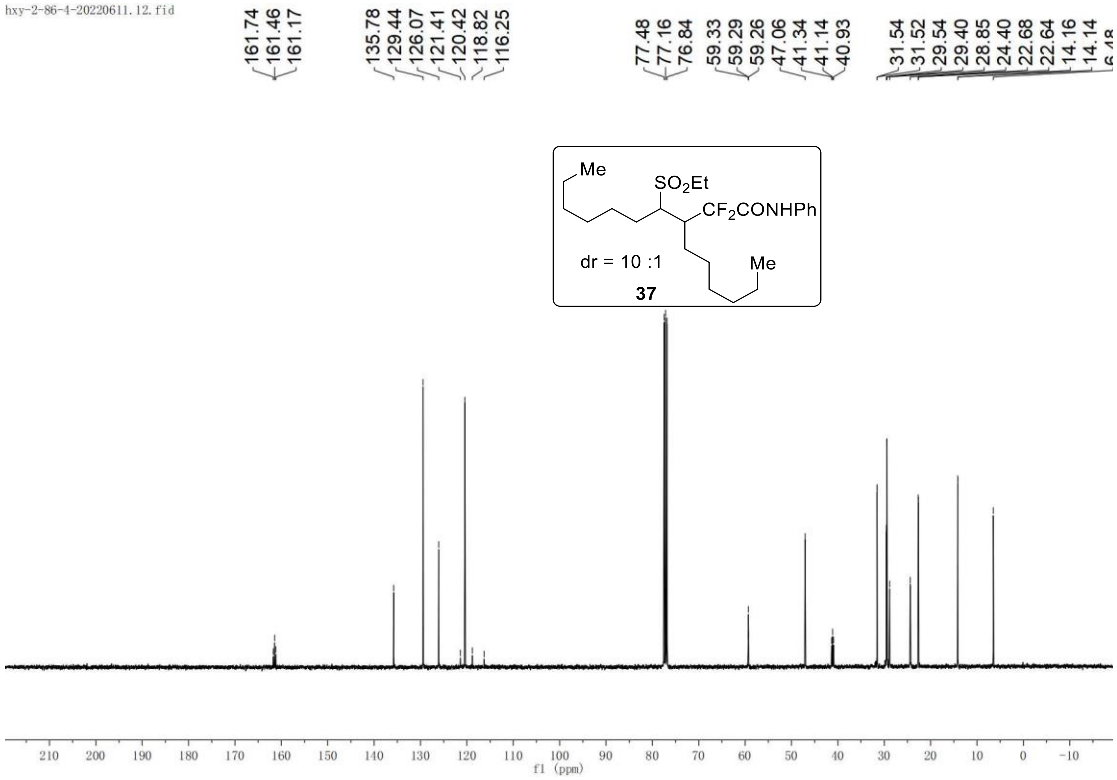

Supplementary Figure 155. ^{13}C NMR (101 MHz, $CDCl_3$) spectra of **37**

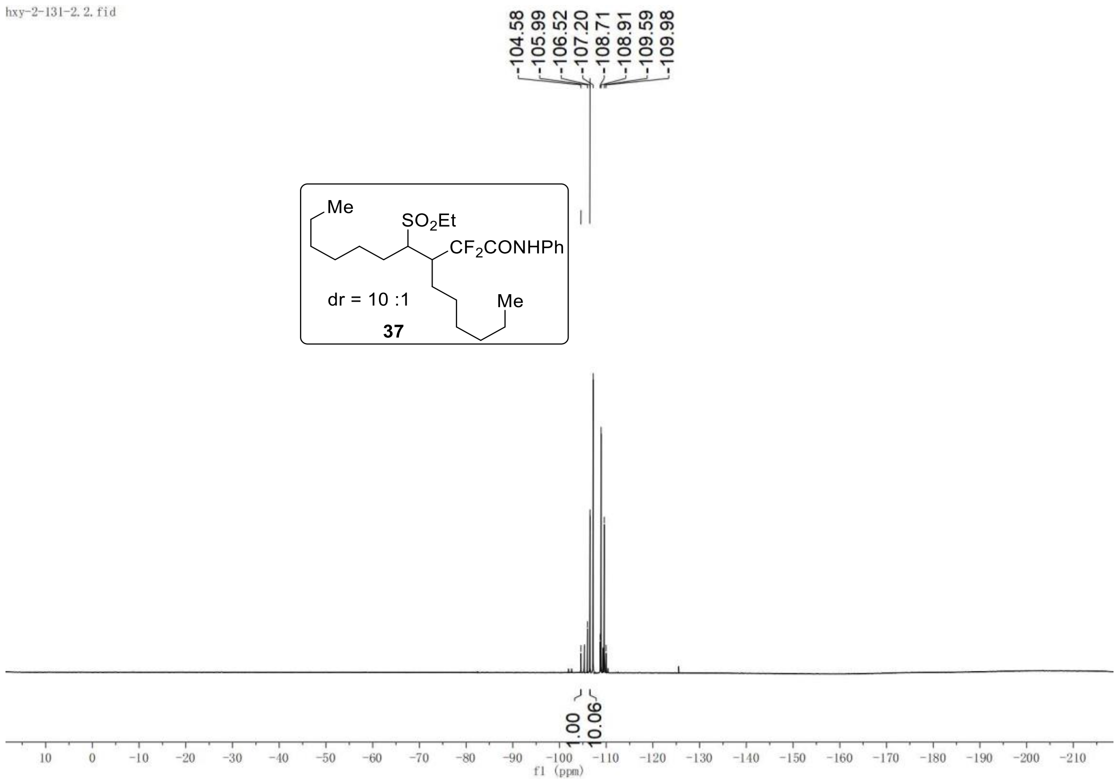

Supplementary Figure 156. ^{19}F NMR (376 MHz, $CDCl_3$) spectra of **37**

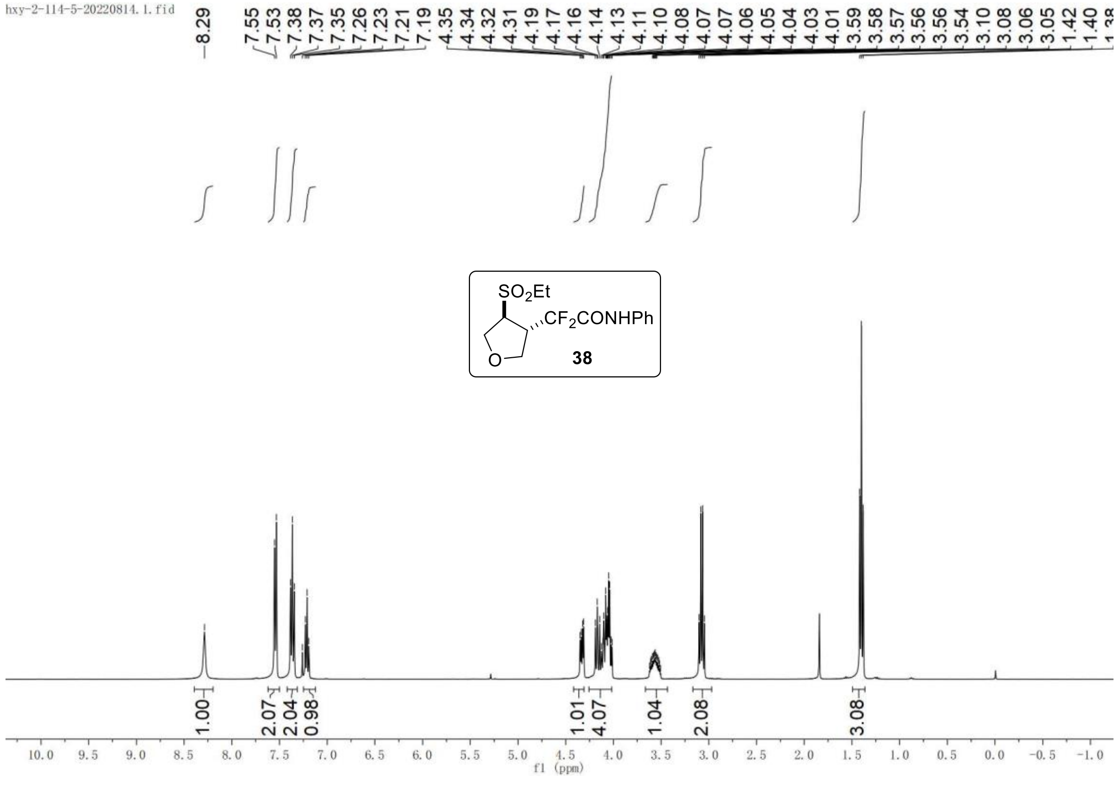

Supplementary Figure 157. ^{1}H NMR (400 MHz, $CDCl_3$) spectra of **38**

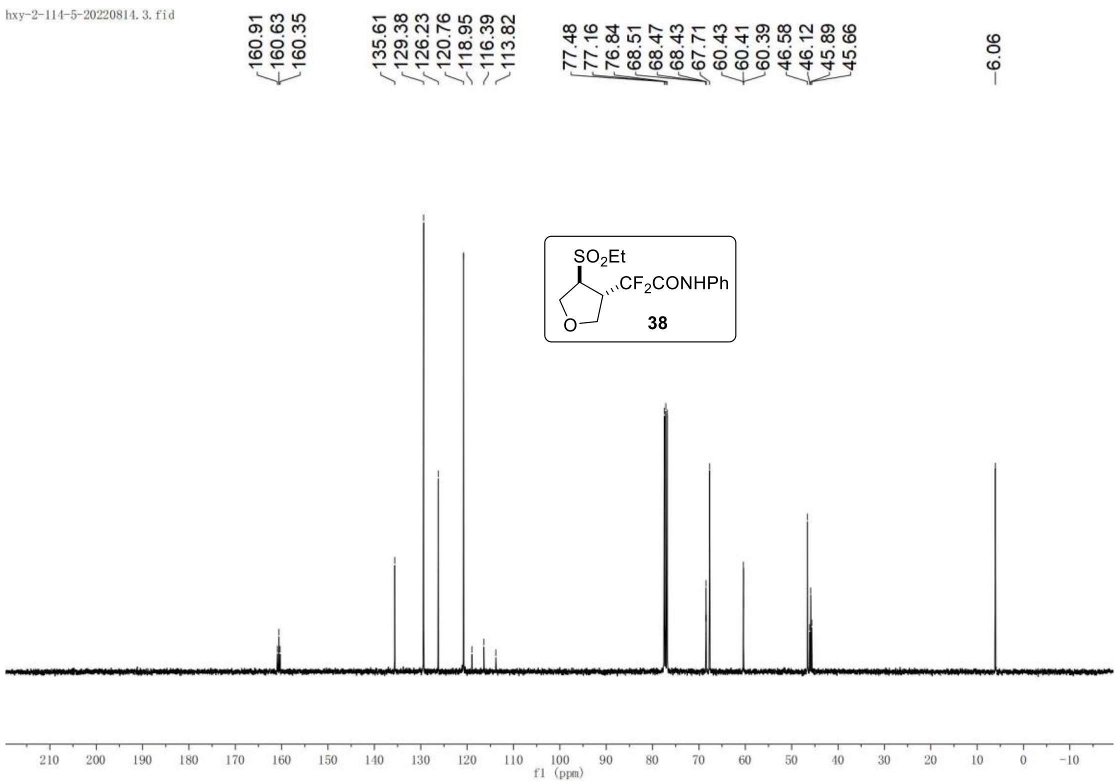

Supplementary Figure 158. ^{13}C NMR (101 MHz, $CDCl_3$) spectra of **38**

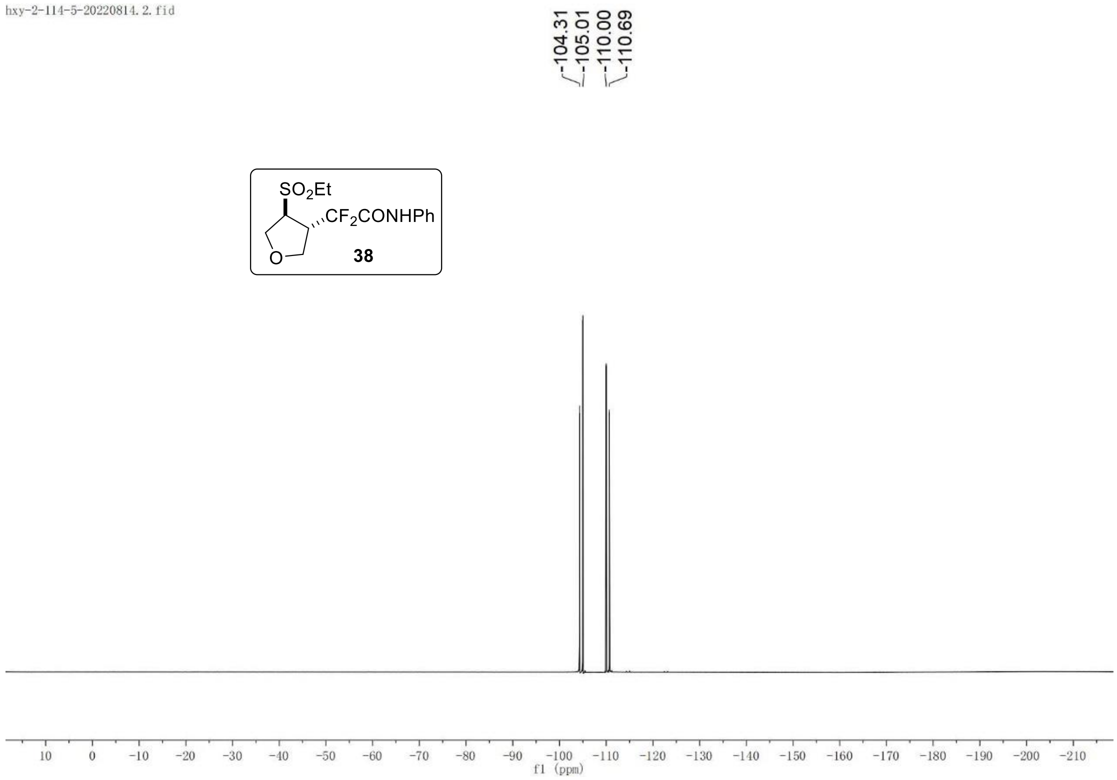

Supplementary Figure 159. ^{19}F NMR (376 MHz, $CDCl_3$) spectra of **38**

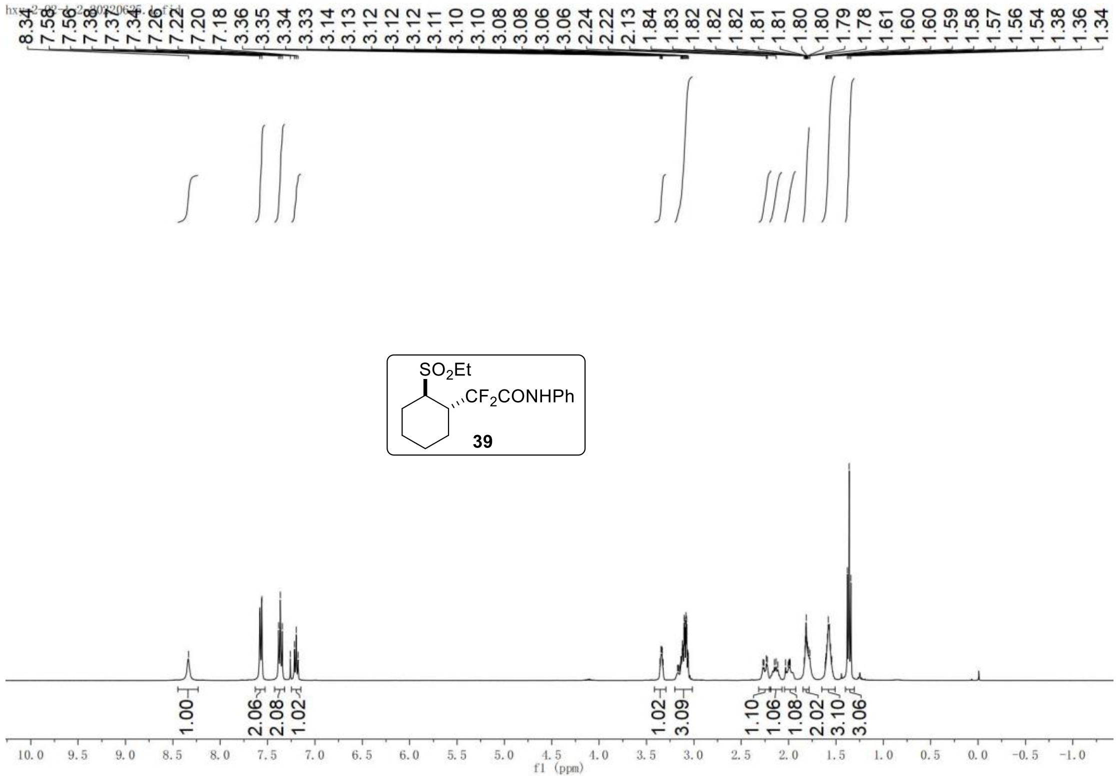

Supplementary Figure 160. ^{1}H NMR (400 MHz, $CDCl_3$) spectra of **39**

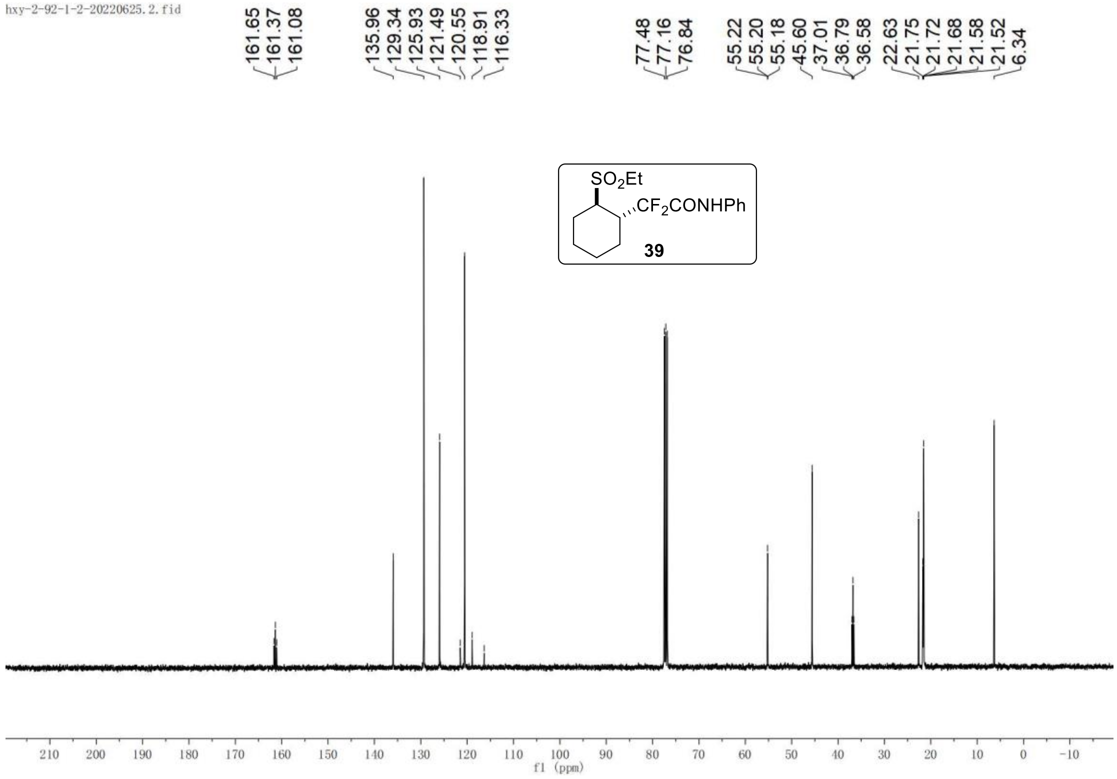

Supplementary Figure 161. ^{13}C NMR (101 MHz, $CDCl_3$) spectra of **39**

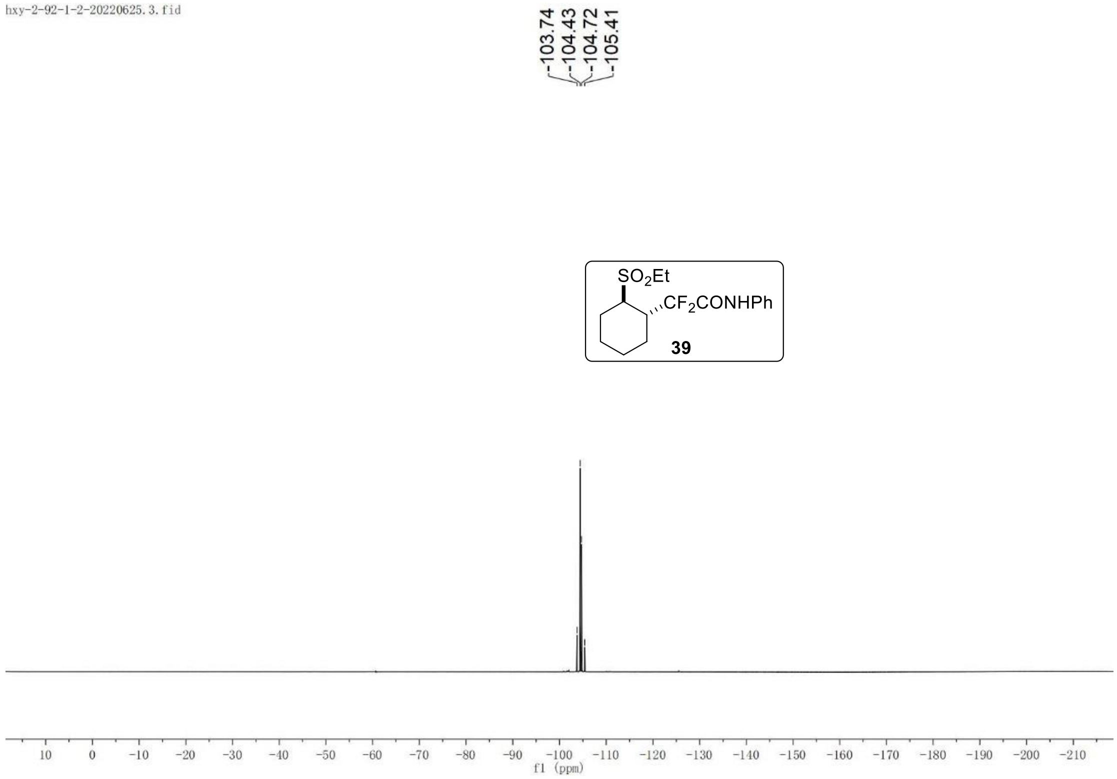

Supplementary Figure 162. ^{19}F NMR (376 MHz, $CDCl_3$) spectra of **39**

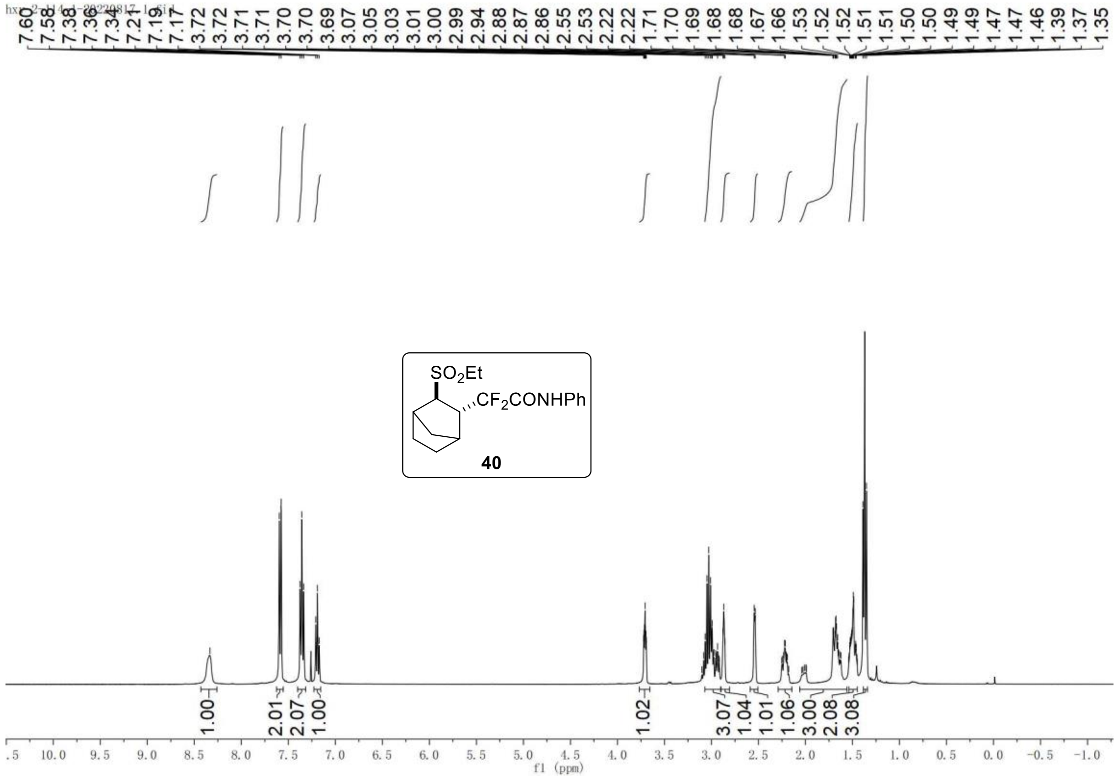

Supplementary Figure 163. ^{1}H NMR (400 MHz, $CDCl_3$) spectra of **40**

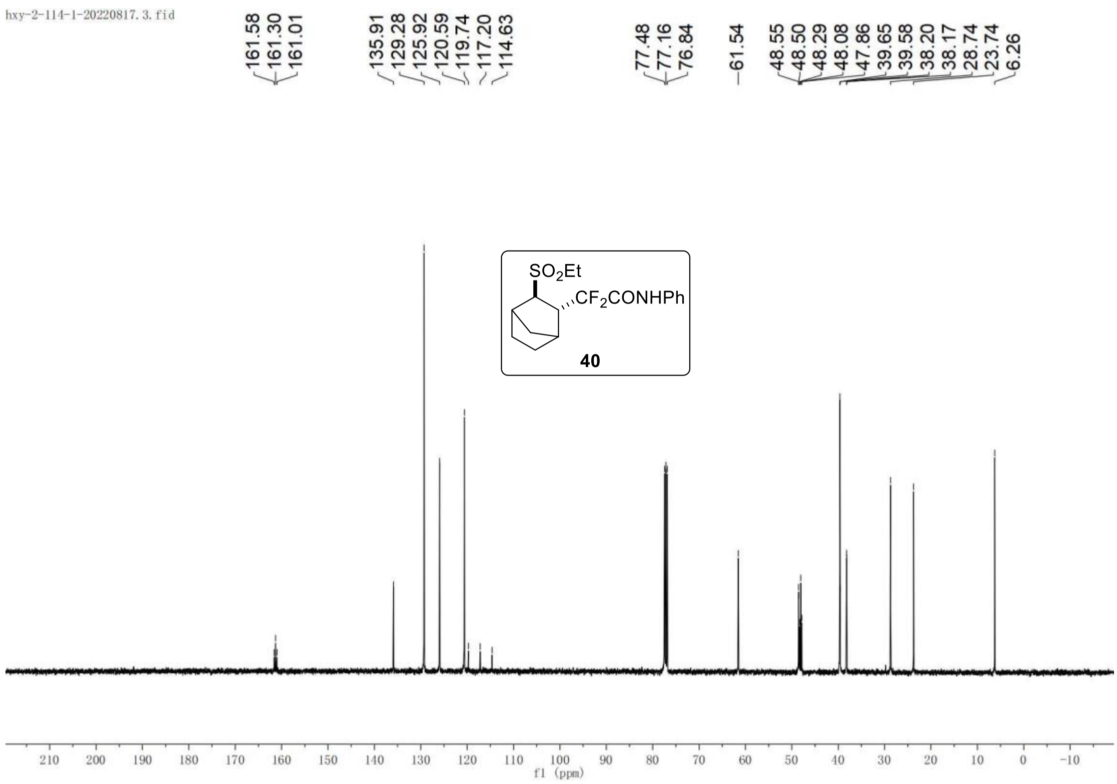

Supplementary Figure 164. ^{13}C NMR (101 MHz, $CDCl_3$) spectra of **40**

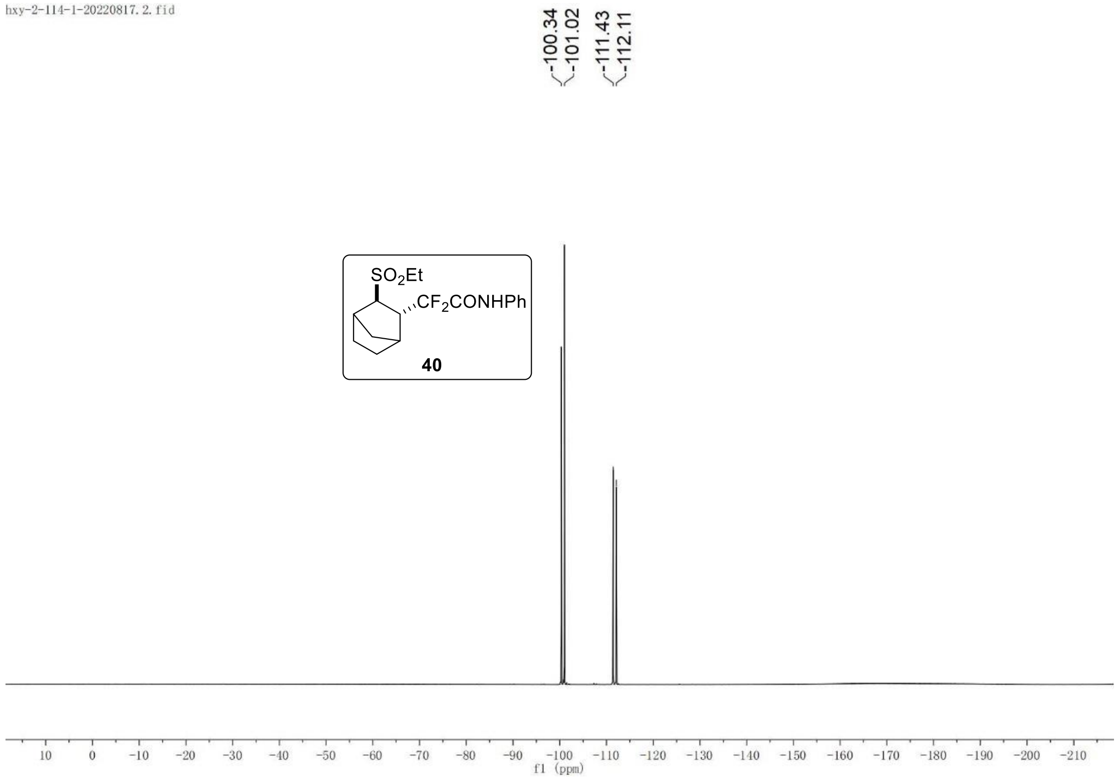

Supplementary Figure 165. ^{19}F NMR (376 MHz, $CDCl_3$) spectra of **40**

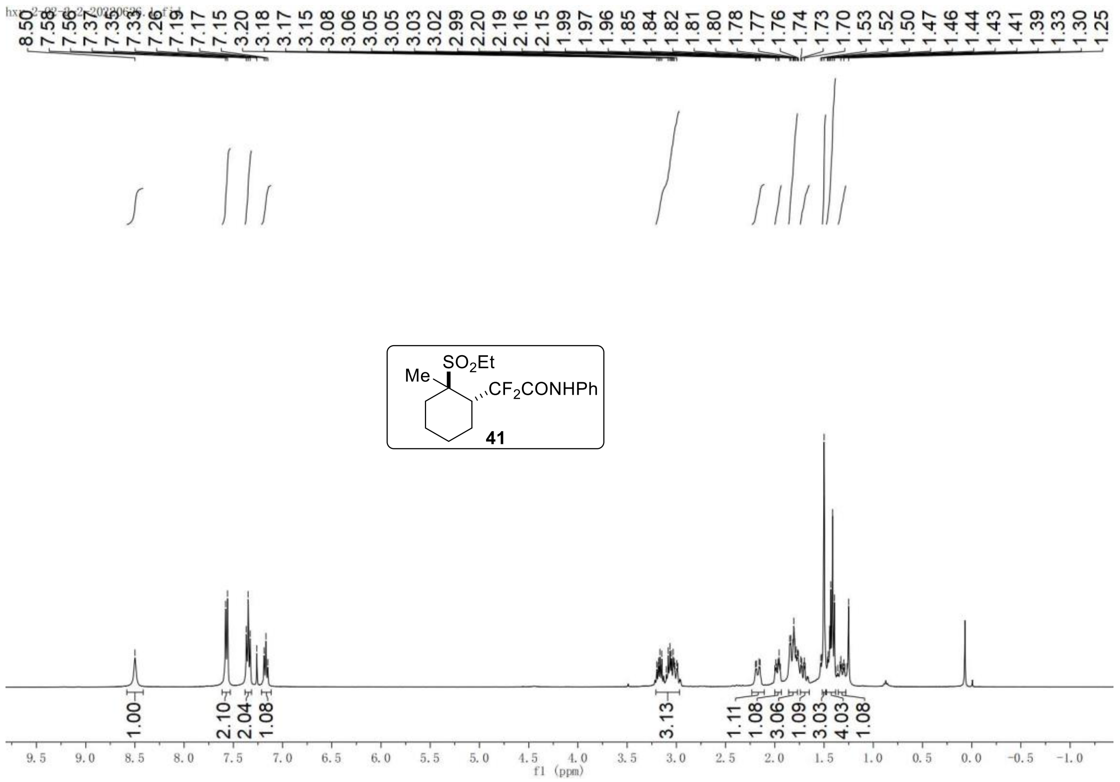

Supplementary Figure 166. ^{1}H NMR (400 MHz, $CDCl_3$) spectra of **41**

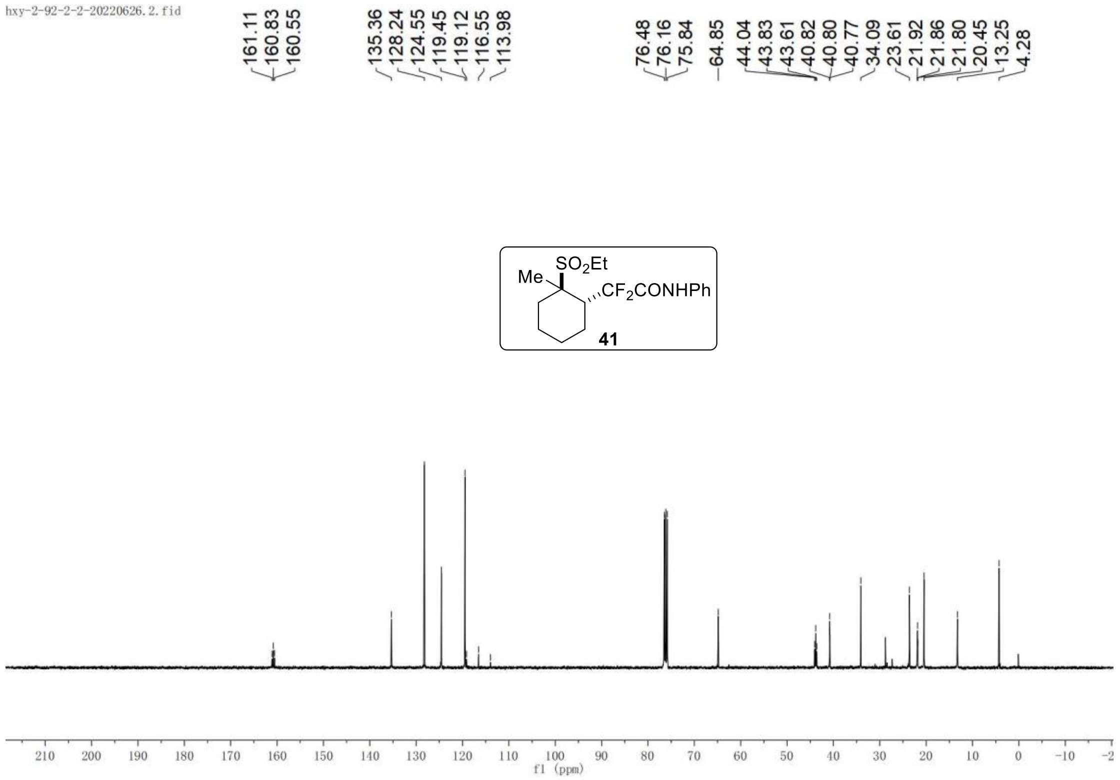

Supplementary Figure 167. ^{13}C NMR (101 MHz, $CDCl_3$) spectra of **41**

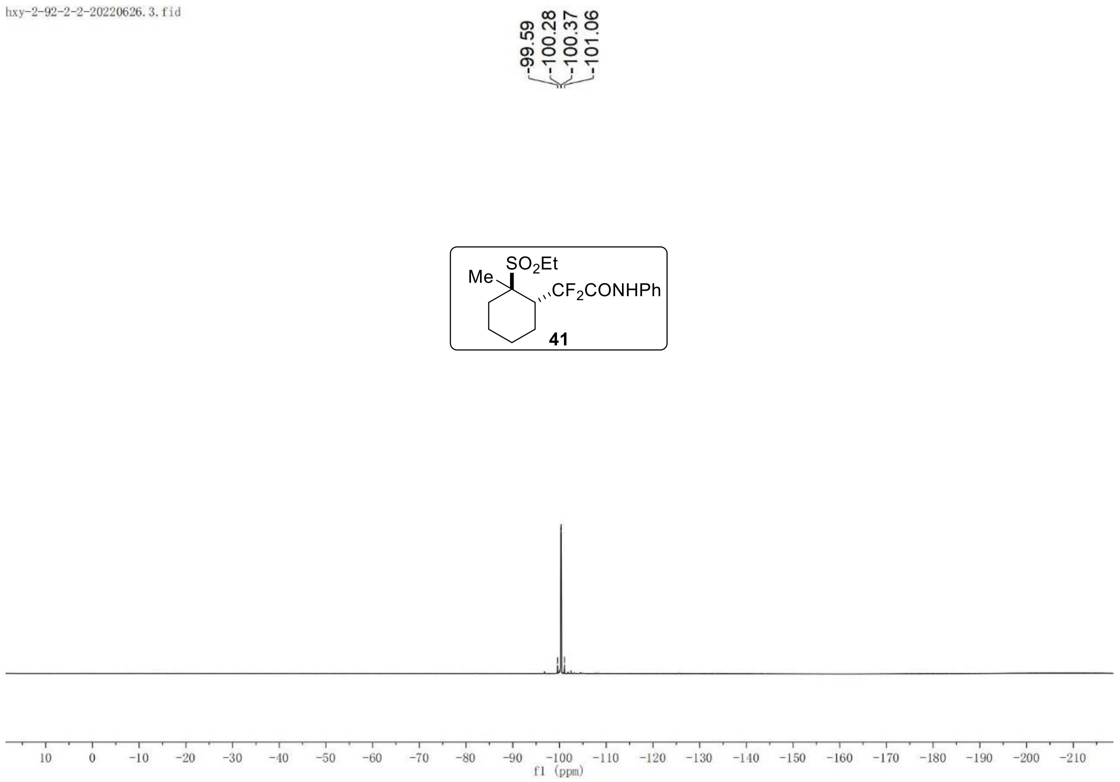

Supplementary Figure 168. ^{19}F NMR (376MHz, $CDCl_3$) spectra of **41**

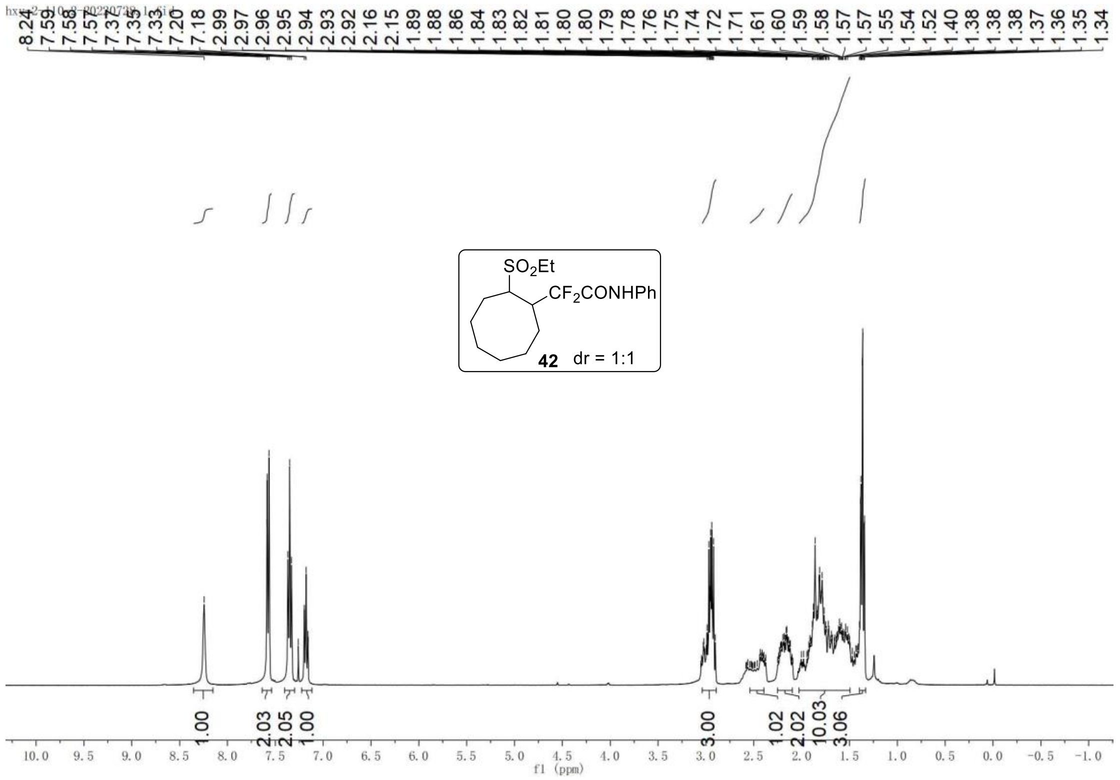

Supplementary Figure 169. ^{1}H NMR (400 MHz, $CDCl_3$) spectra of **42**

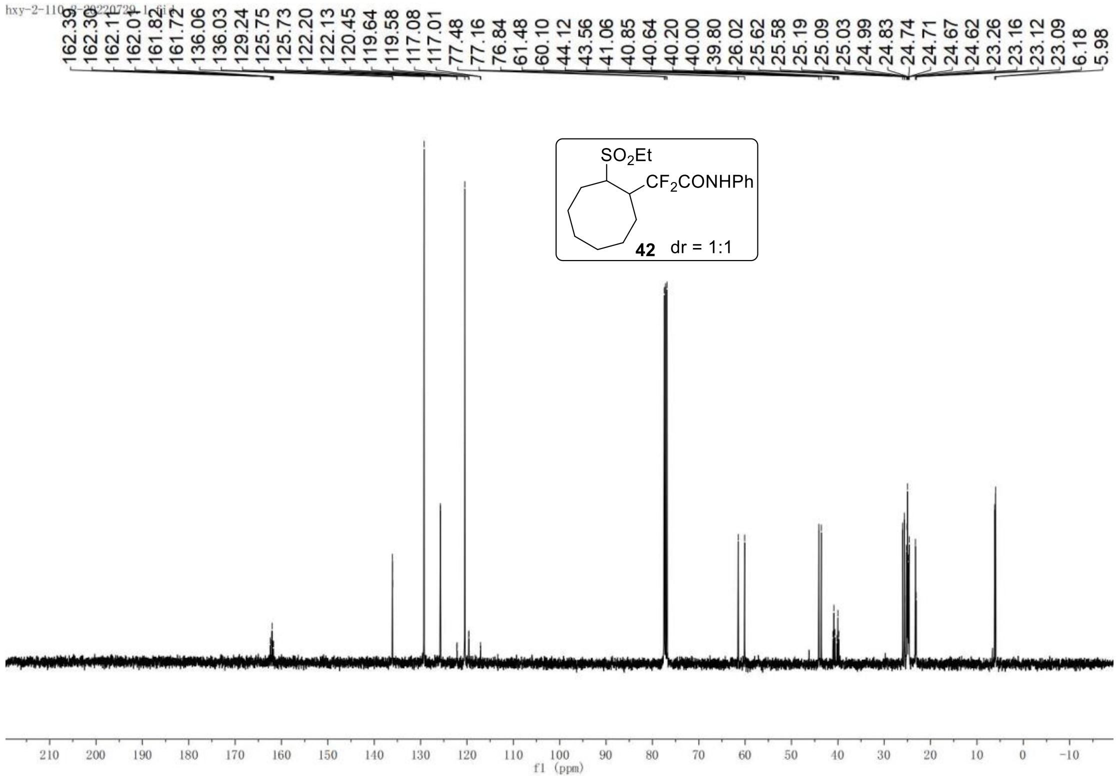

Supplementary Figure 170. ^{13}C NMR (101 MHz, $CDCl_3$) spectra of **42**

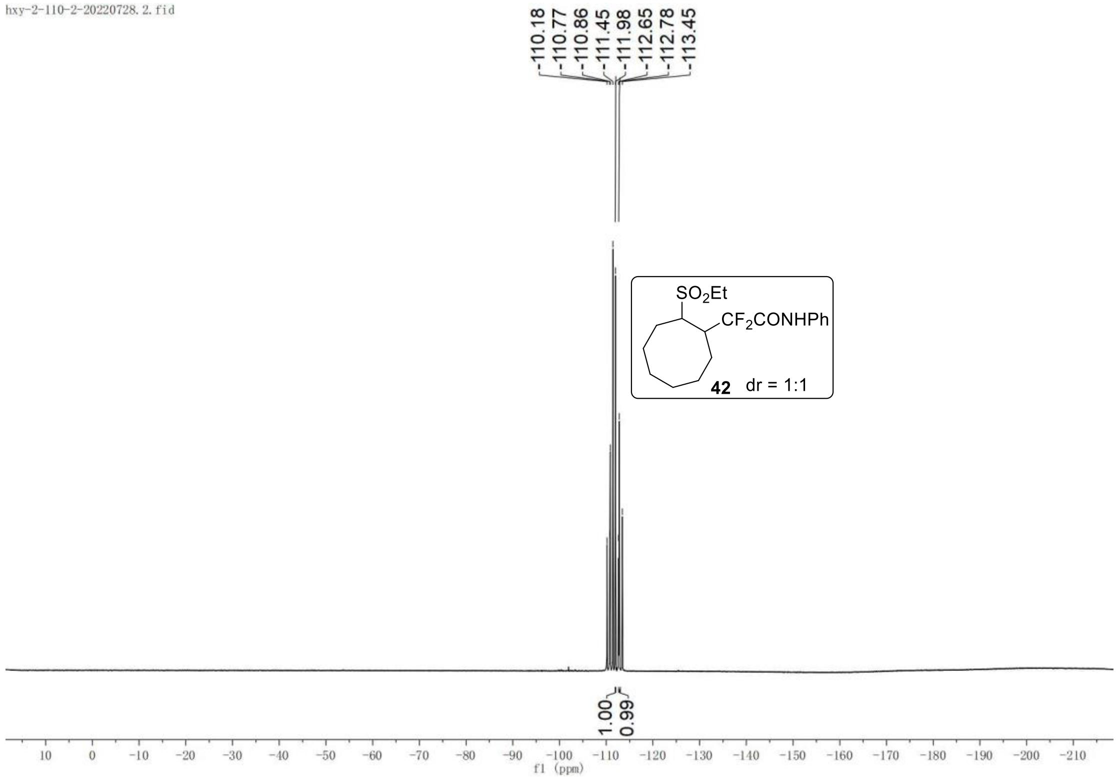

Supplementary Figure 171. ^{19}F NMR (376 MHz, $CDCl_3$) spectra of **42**

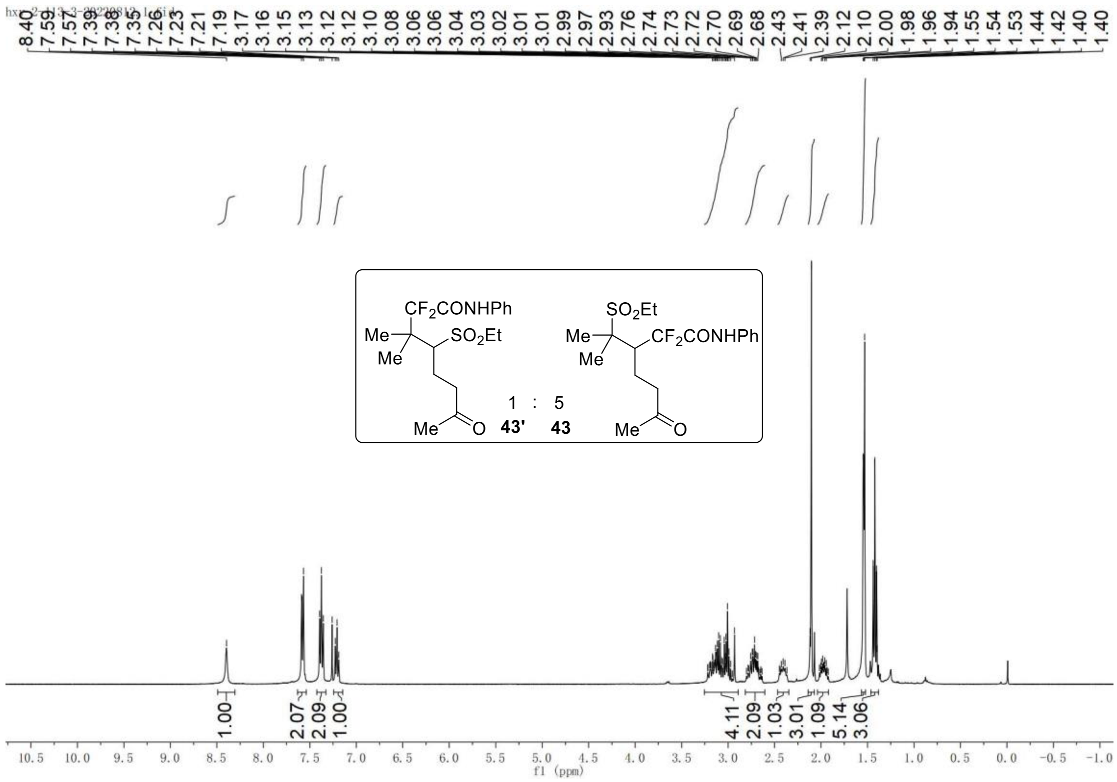

Supplementary Figure 172. ^{1}H NMR (400 MHz, $CDCl_3$) spectra of **43**

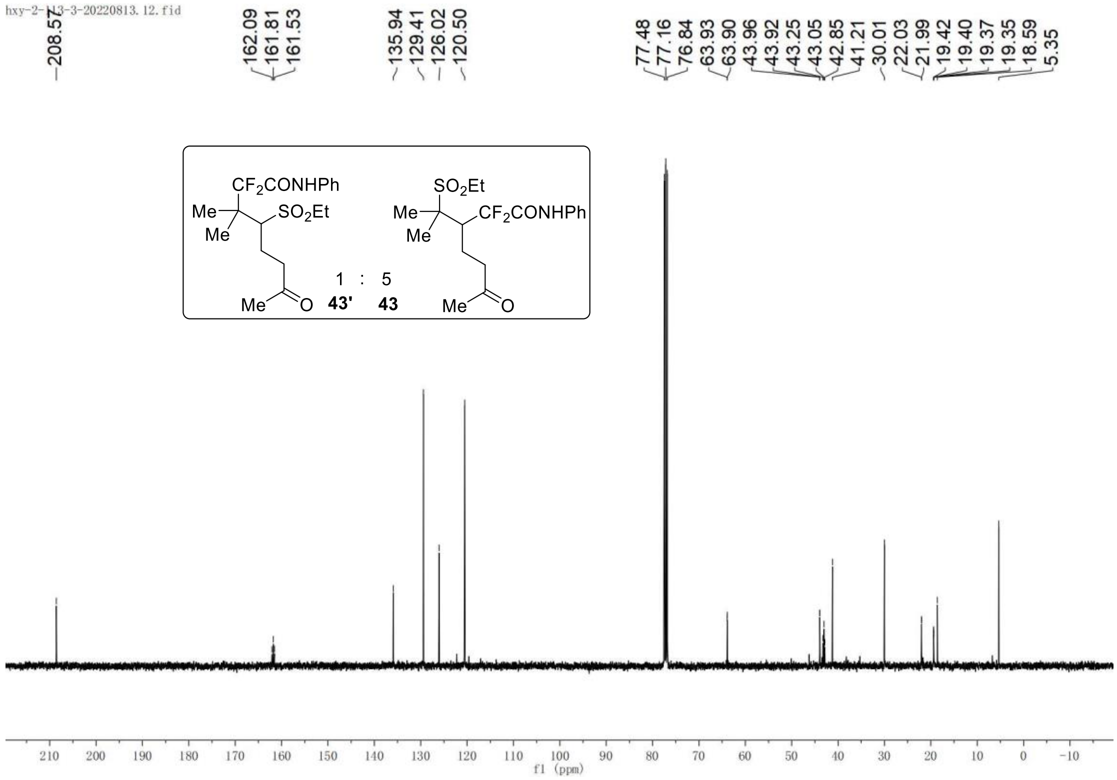

Supplementary Figure 173. ^{13}C NMR (101 MHz, $CDCl_3$) spectra of **43**

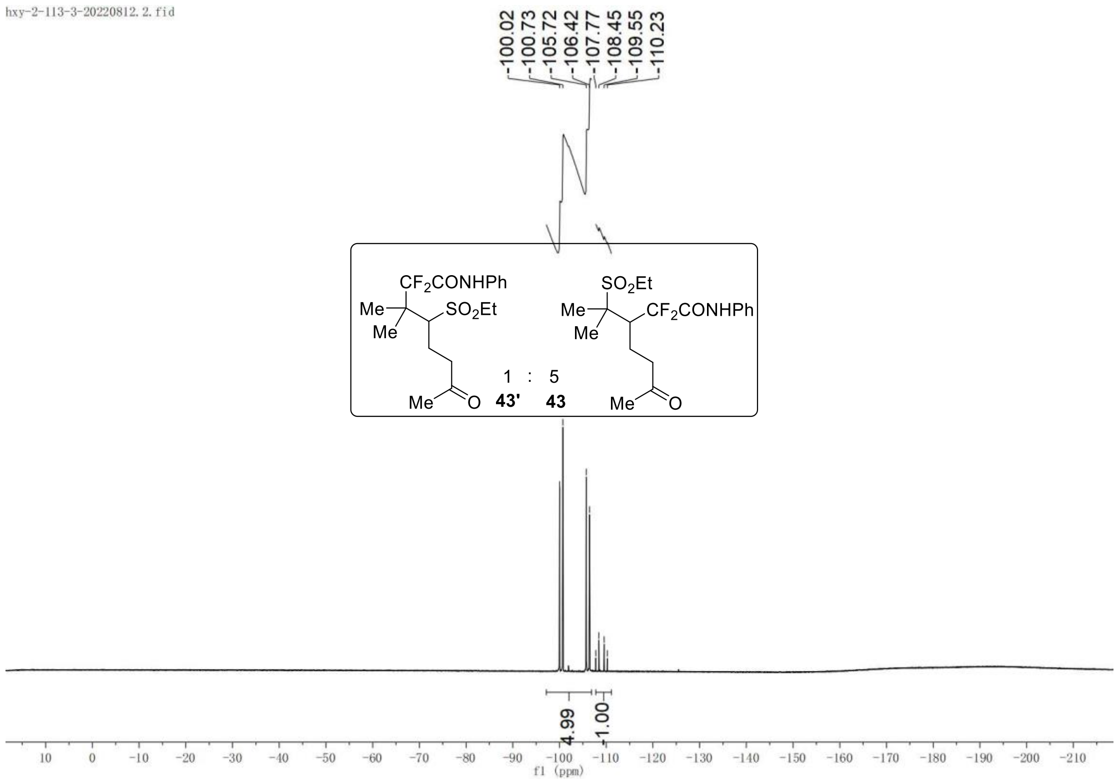

Supplementary Figure 174. ^{19}F NMR (376 MHz, $CDCl_3$) spectra of **43**

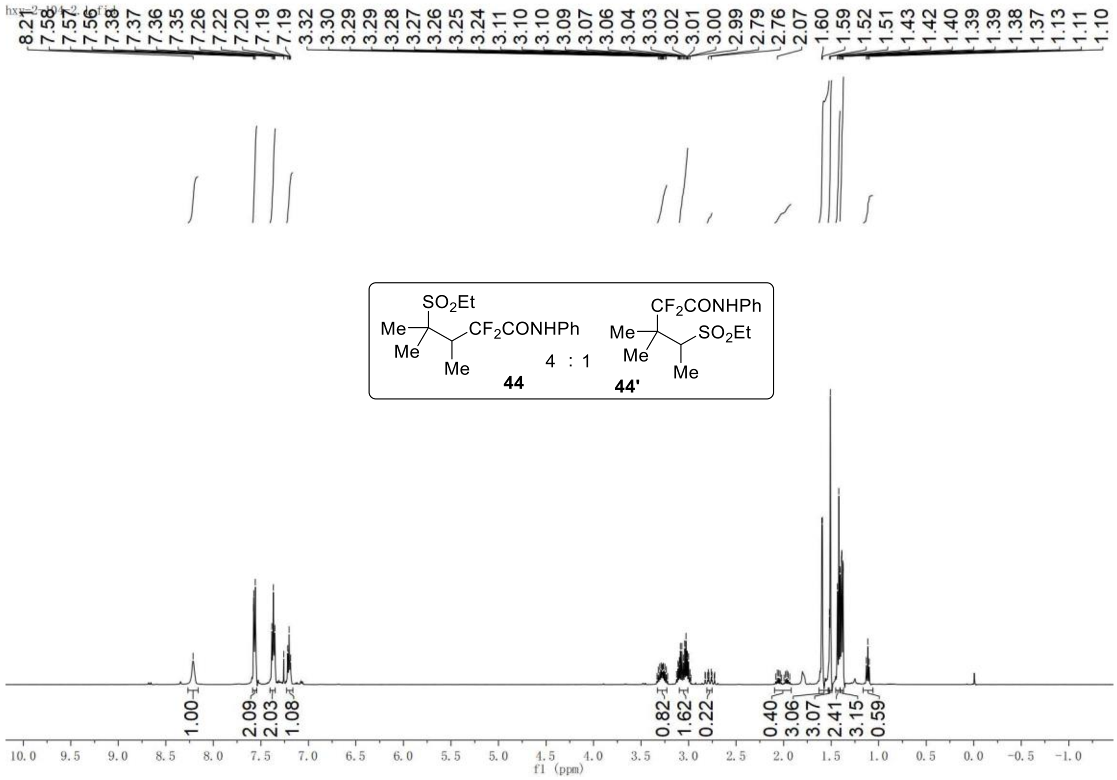

Supplementary Figure 175. ^{1}H NMR (500 MHz, $CDCl_3$) spectra of **44**

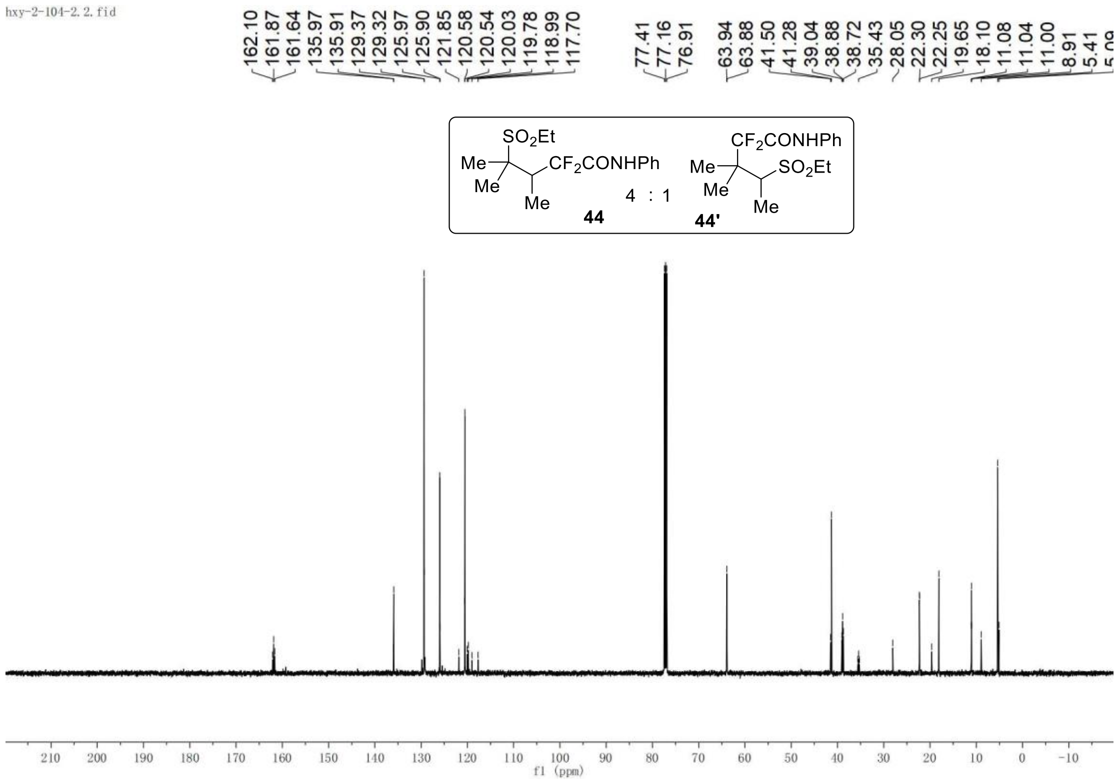

Supplementary Figure 176. ^{13}C NMR (126 MHz, $CDCl_3$) spectra of **44**

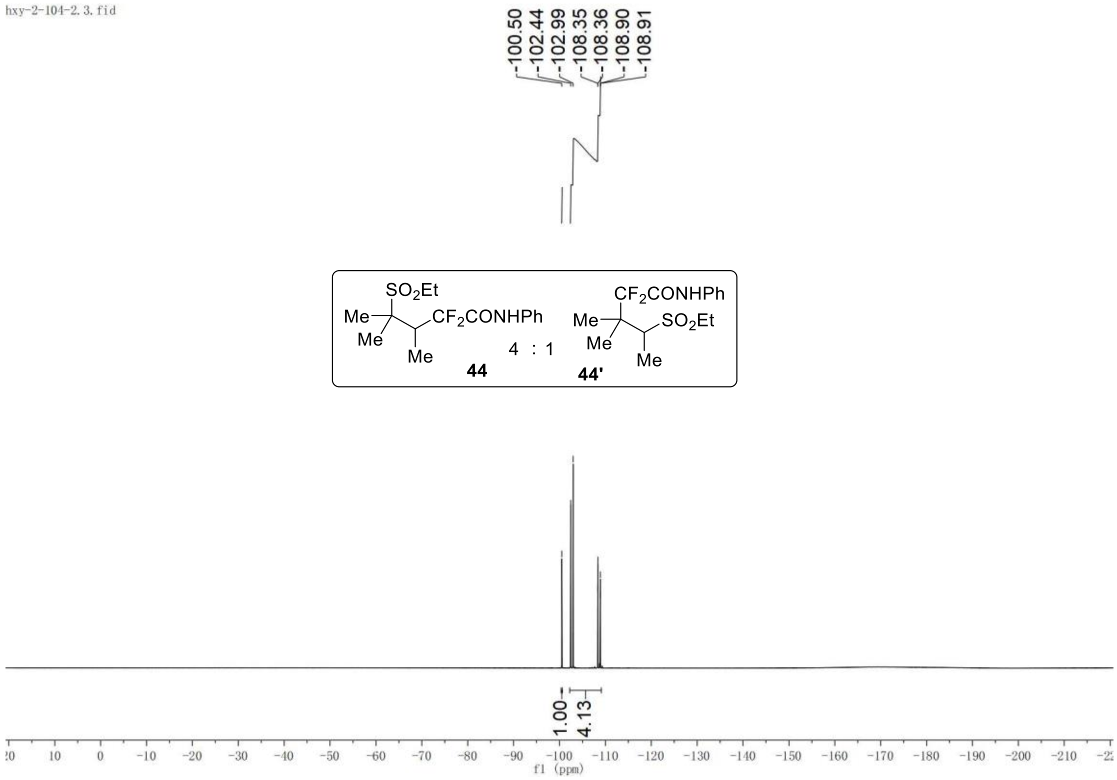

Supplementary Figure 177. ^{19}F NMR (471 MHz, $CDCl_3$) spectra of **44**

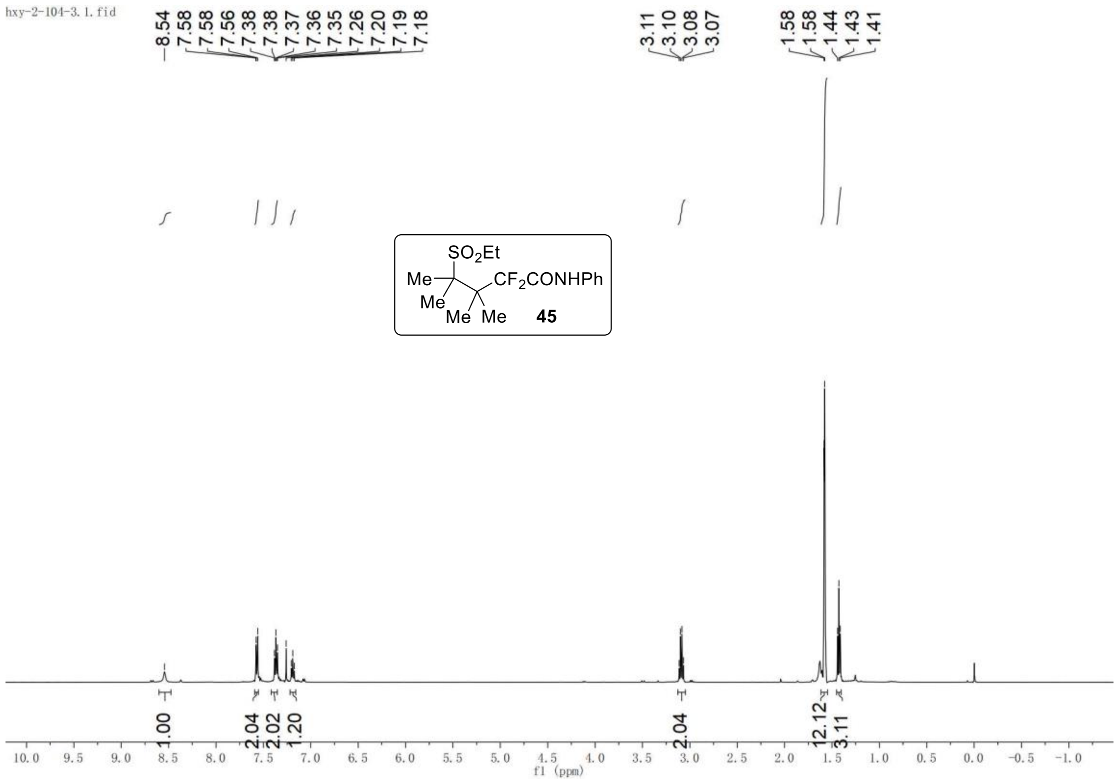

Supplementary Figure 178. ^{1}H NMR (500 MHz, $CDCl_3$) spectra of **45**

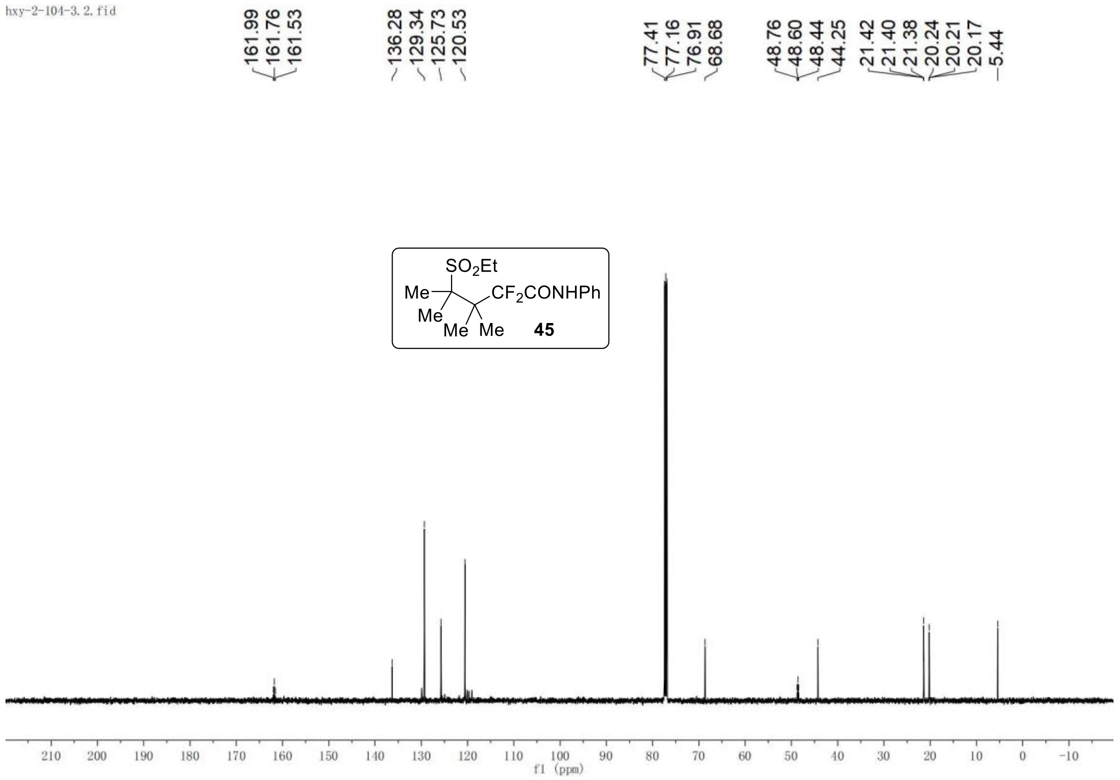

Supplementary Figure 179. ^{13}C NMR (126 MHz, $CDCl_3$) spectra of **45**

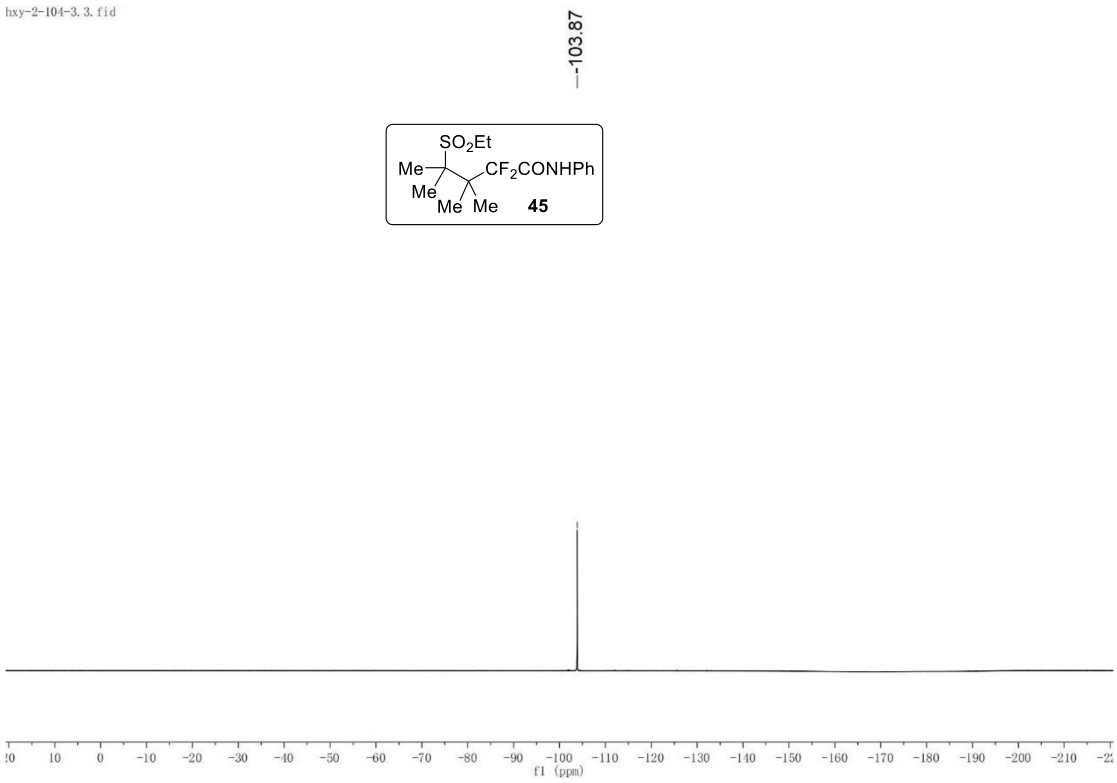

Supplementary Figure 180. ^{19}F NMR (471 MHz, $CDCl_3$) spectra of **45**

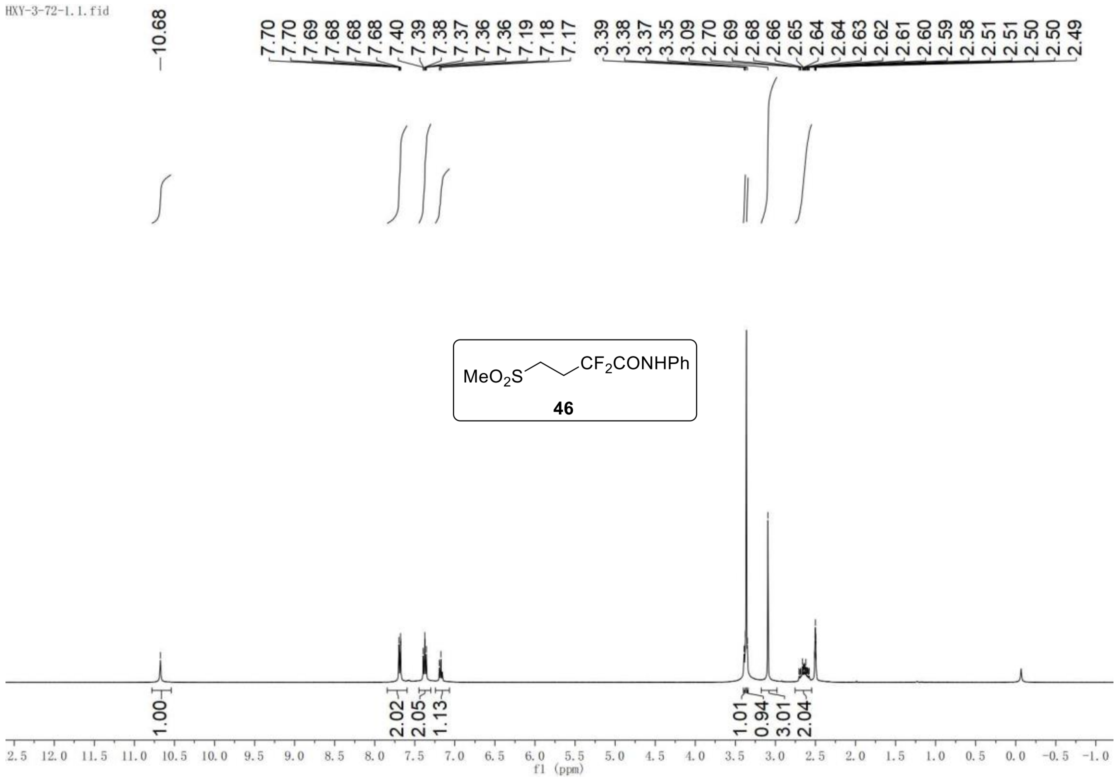

Supplementary Figure 181. ^{1}H NMR (400 MHz, DMSO-d_6) spectra of **46**

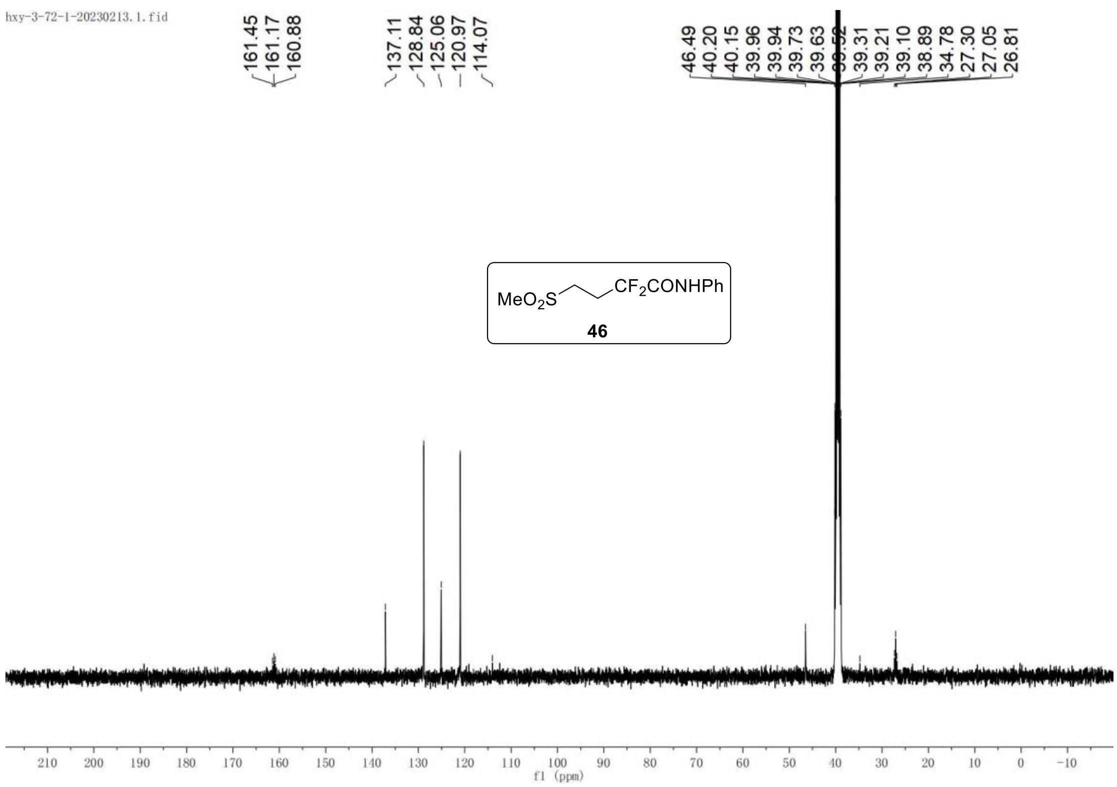

Supplementary Figure 182. ^{13}C NMR (101 MHz, DMSO-d_6) spectra of **46**

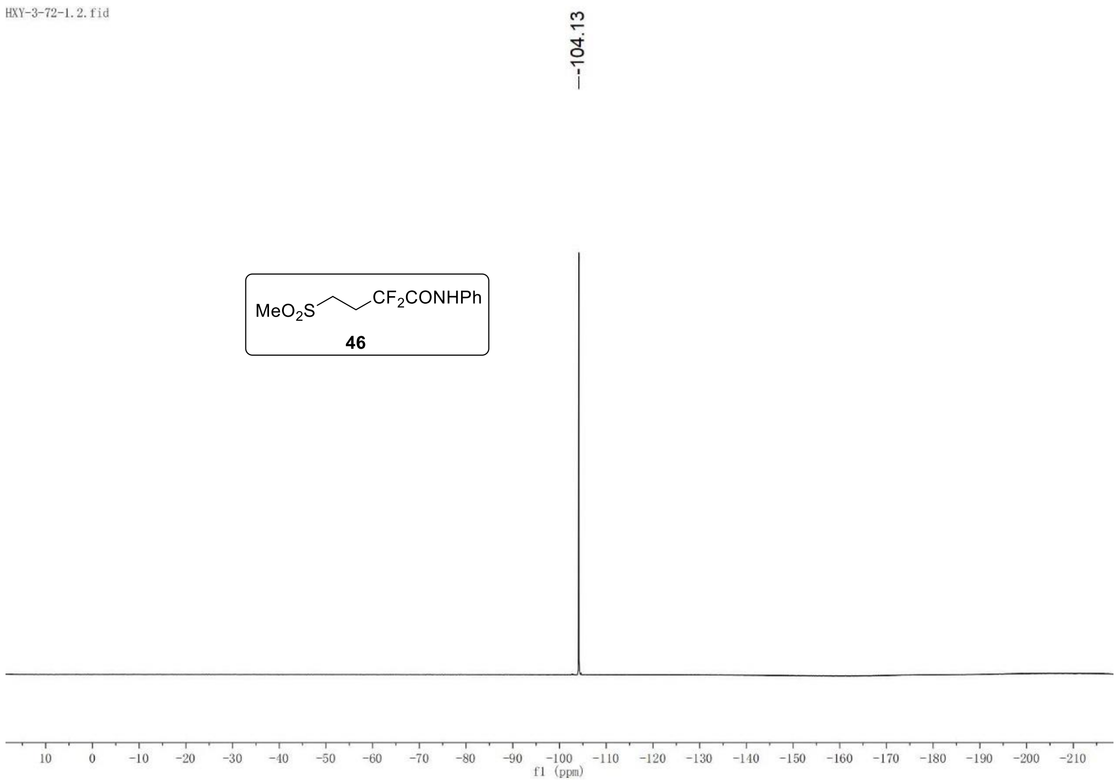

Supplementary Figure 183. ^{19}F NMR (376 MHz, DMSO-d_6) spectra of **46**

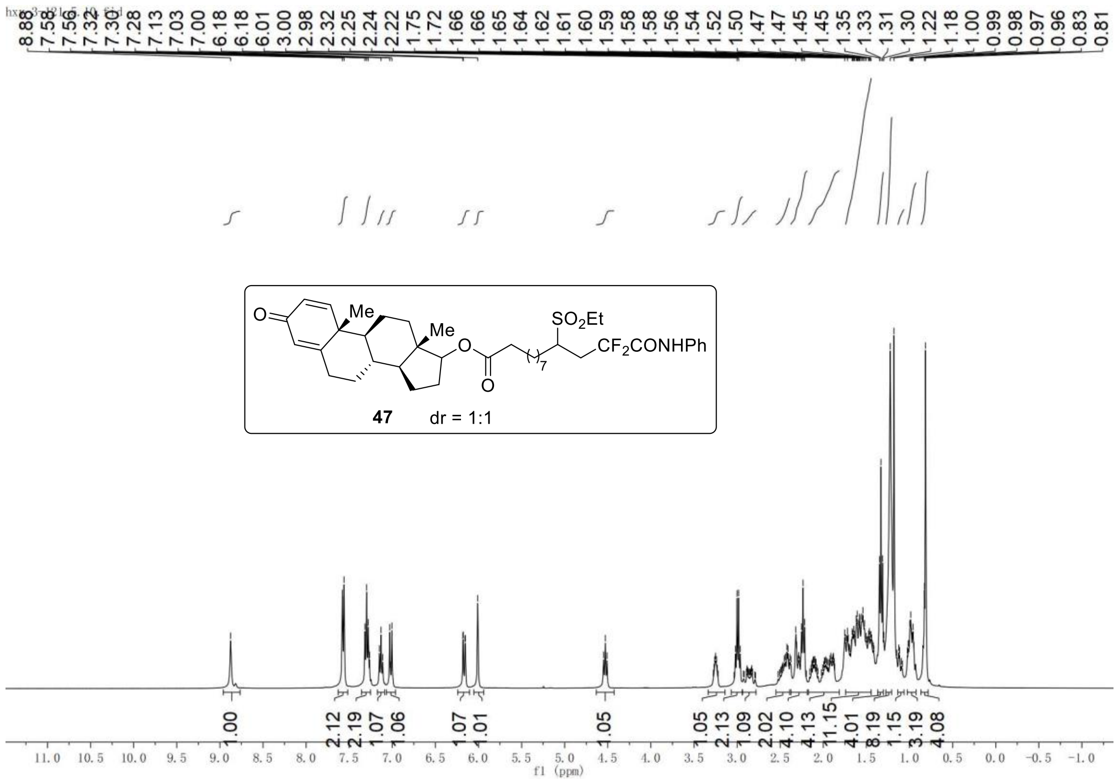

Supplementary Figure 184. ^{1}H NMR (400 MHz, $CDCl_3$) spectra of **47**

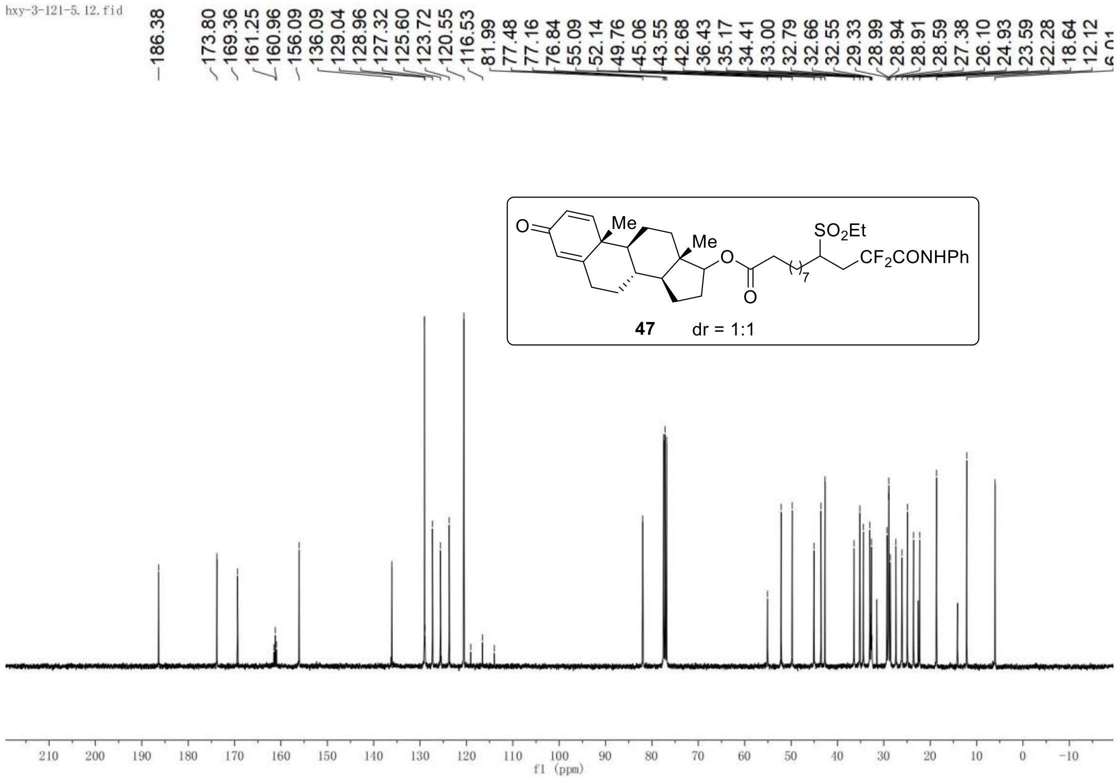

Supplementary Figure 185. ^{13}C NMR (101 MHz, $CDCl_3$) spectra of **47**

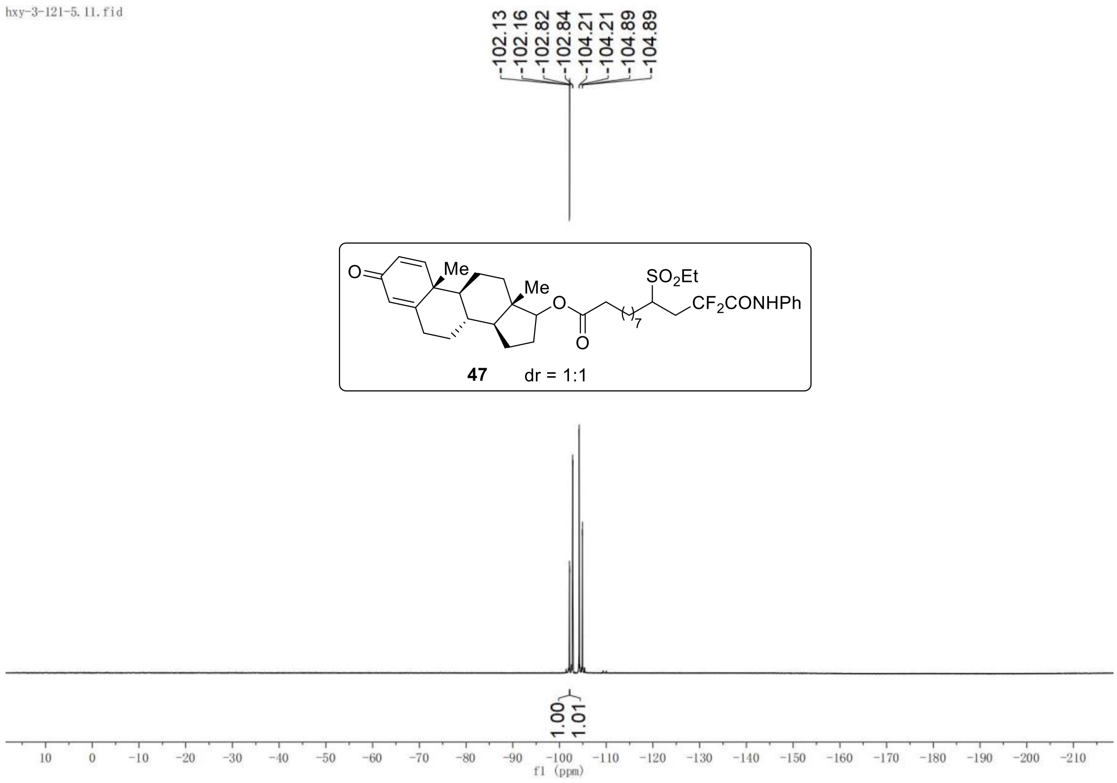

Supplementary Figure 186. ^{19}F NMR (376 MHz, $CDCl_3$) spectra of **47**

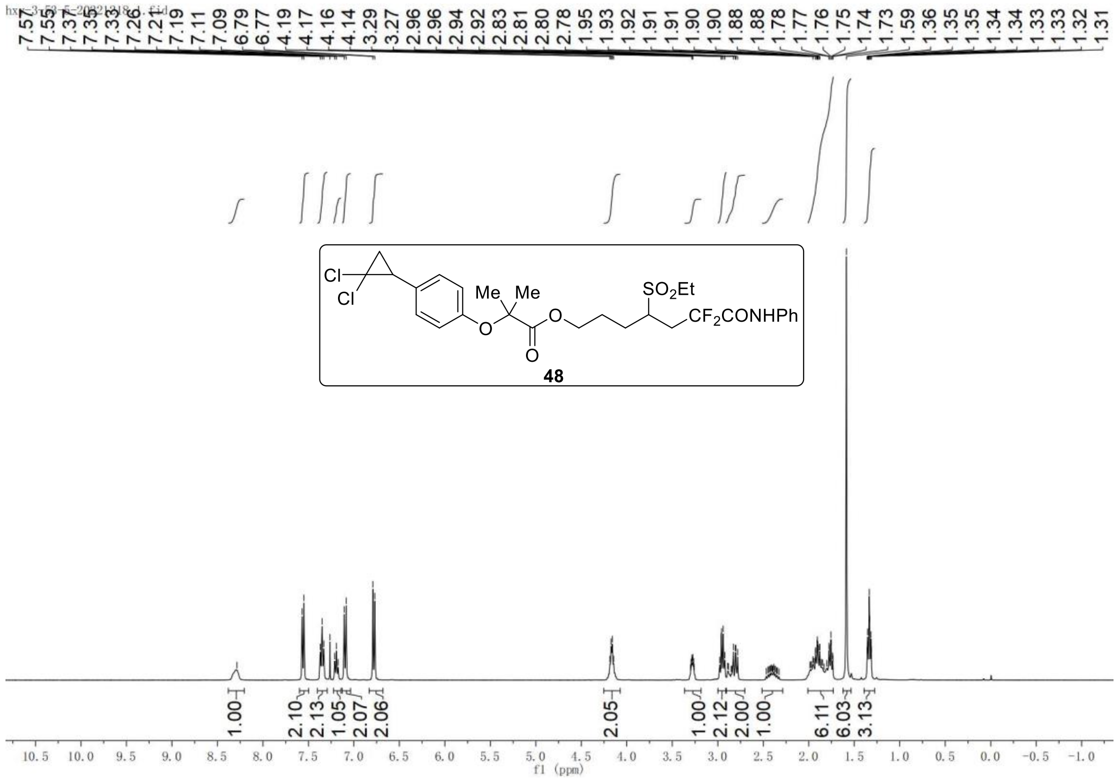

Supplementary Figure 187. ^{1}H NMR (400 MHz, $CDCl_3$) spectra of **48**

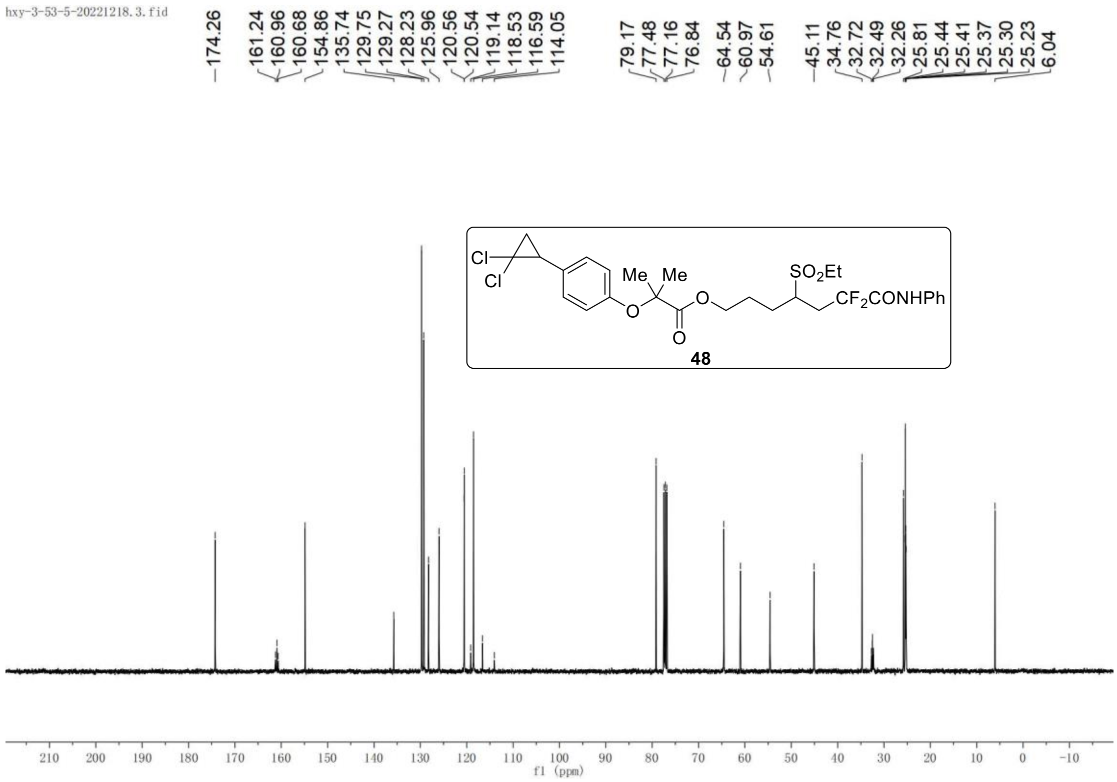

Supplementary Figure 188. ^{13}C NMR (101 MHz, $CDCl_3$) spectra of **48**

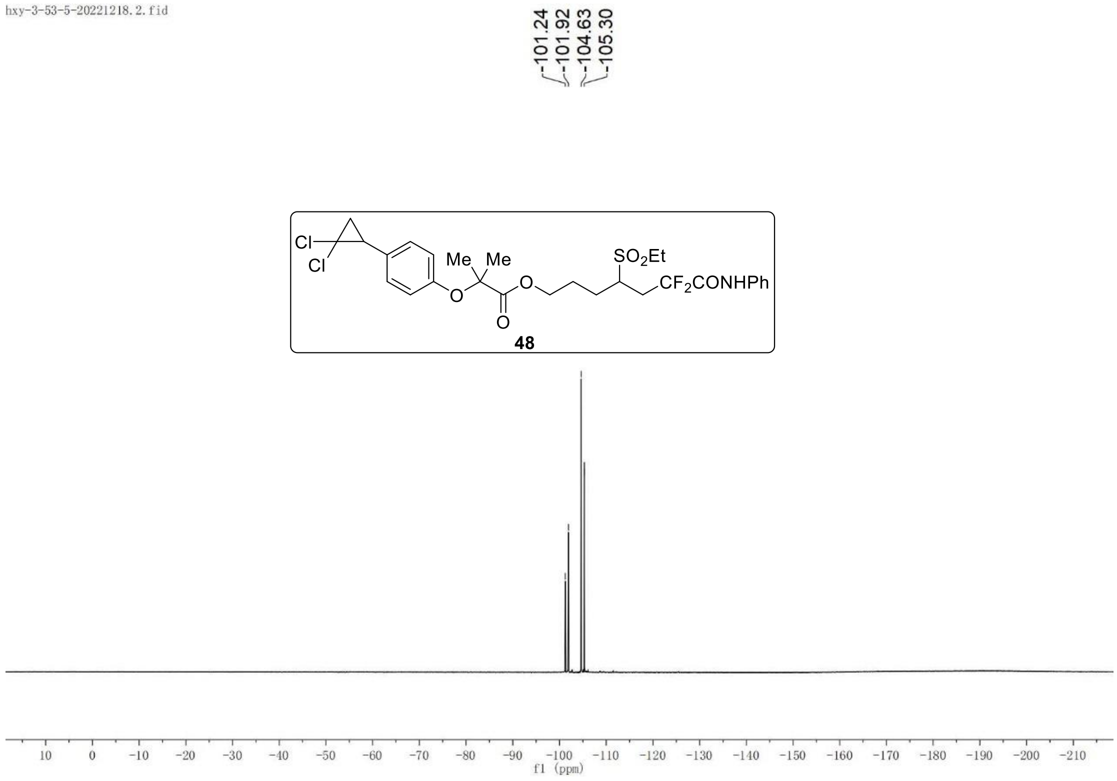

Supplementary Figure 189. ^{19}F NMR (376 MHz, $CDCl_3$) spectra of **48**

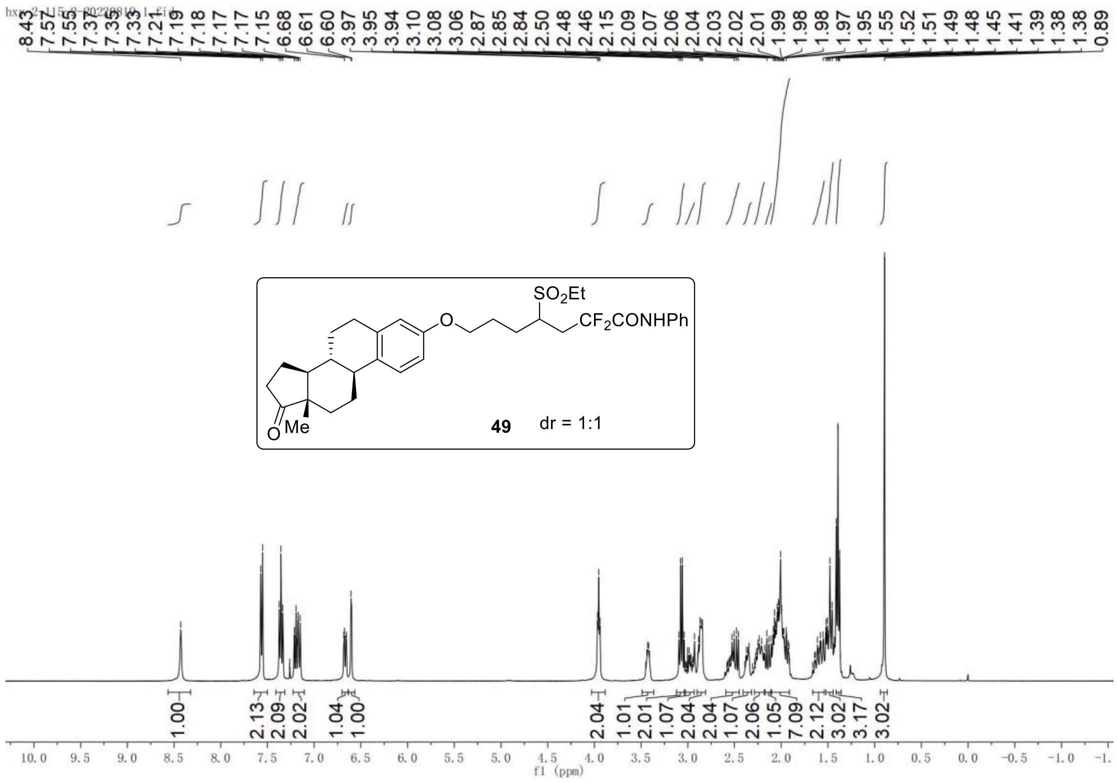

Supplementary Figure 190. ^{1}H NMR (400 MHz, $CDCl_3$) spectra of **49**

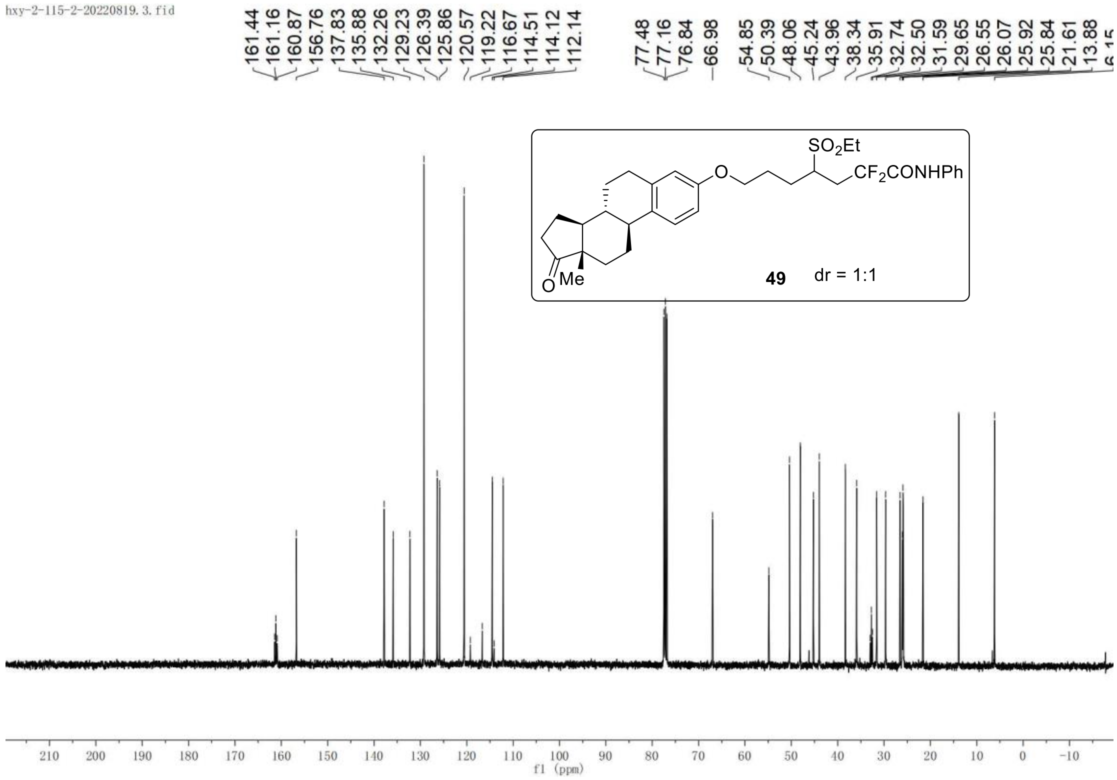

Supplementary Figure 191. ^{13}C NMR (101 MHz, $CDCl_3$) spectra of **49**

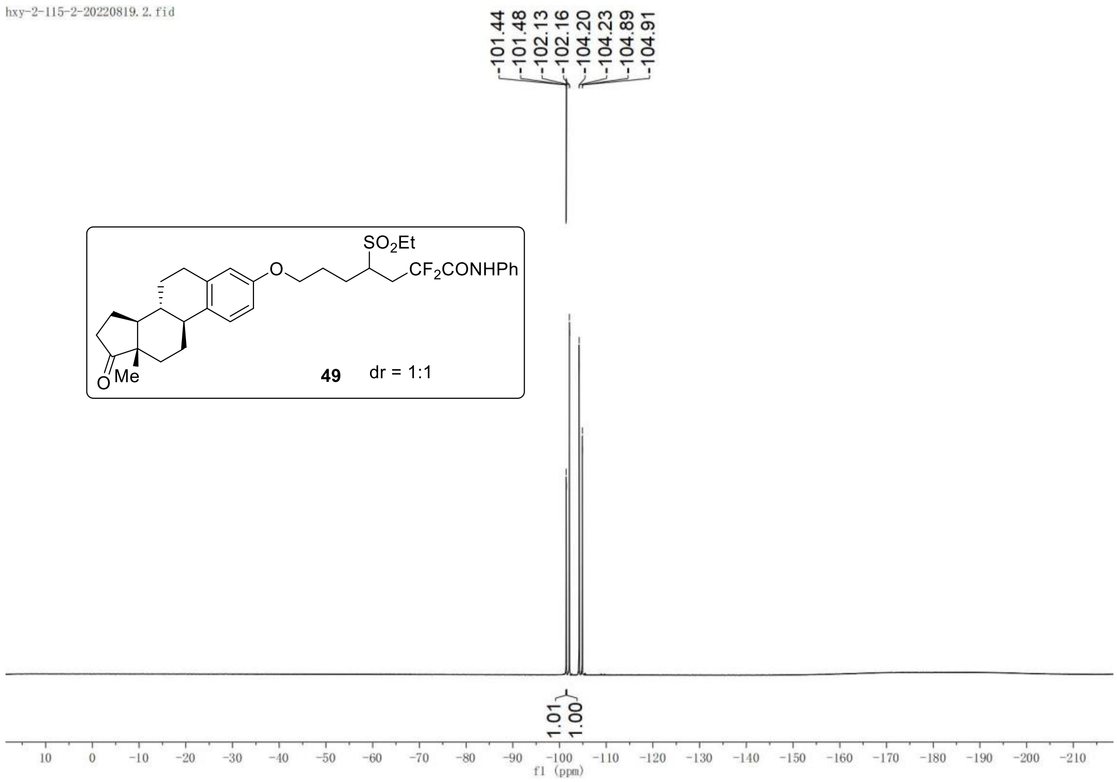

Supplementary Figure 192. ^{19}F NMR (376 MHz, $CDCl_3$) spectra of **49**

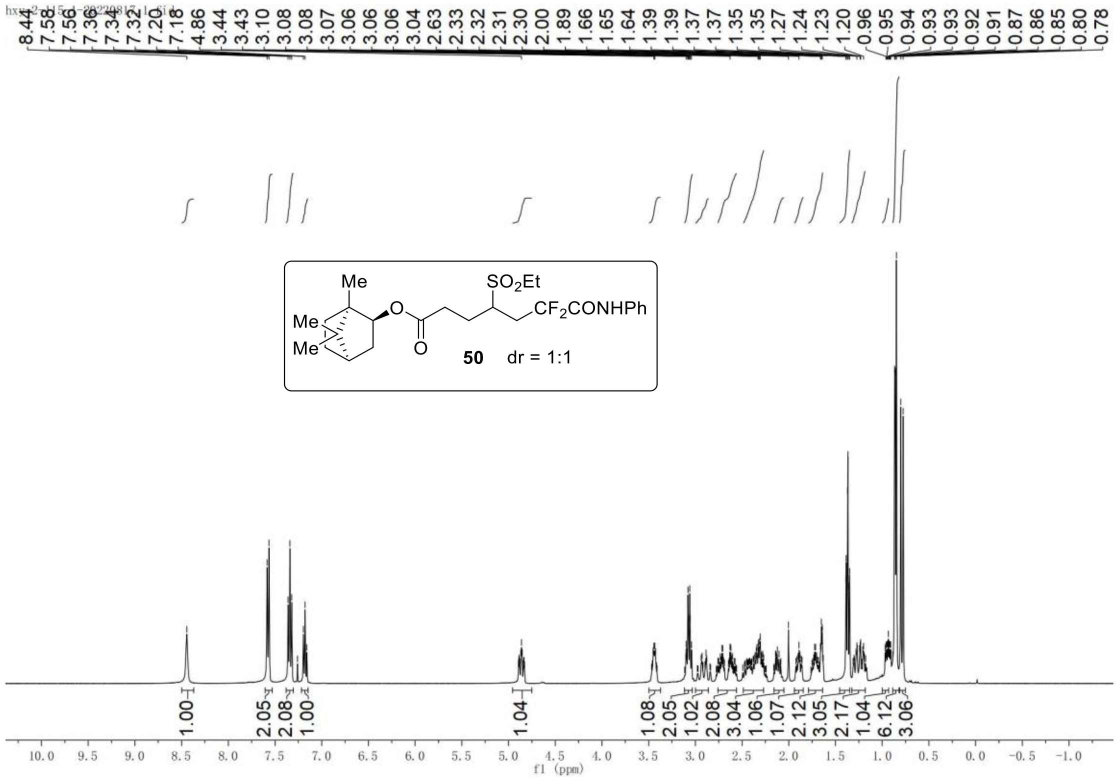

Supplementary Figure 193. ^{1}H NMR (400 MHz, $CDCl_3$) spectra of **50**

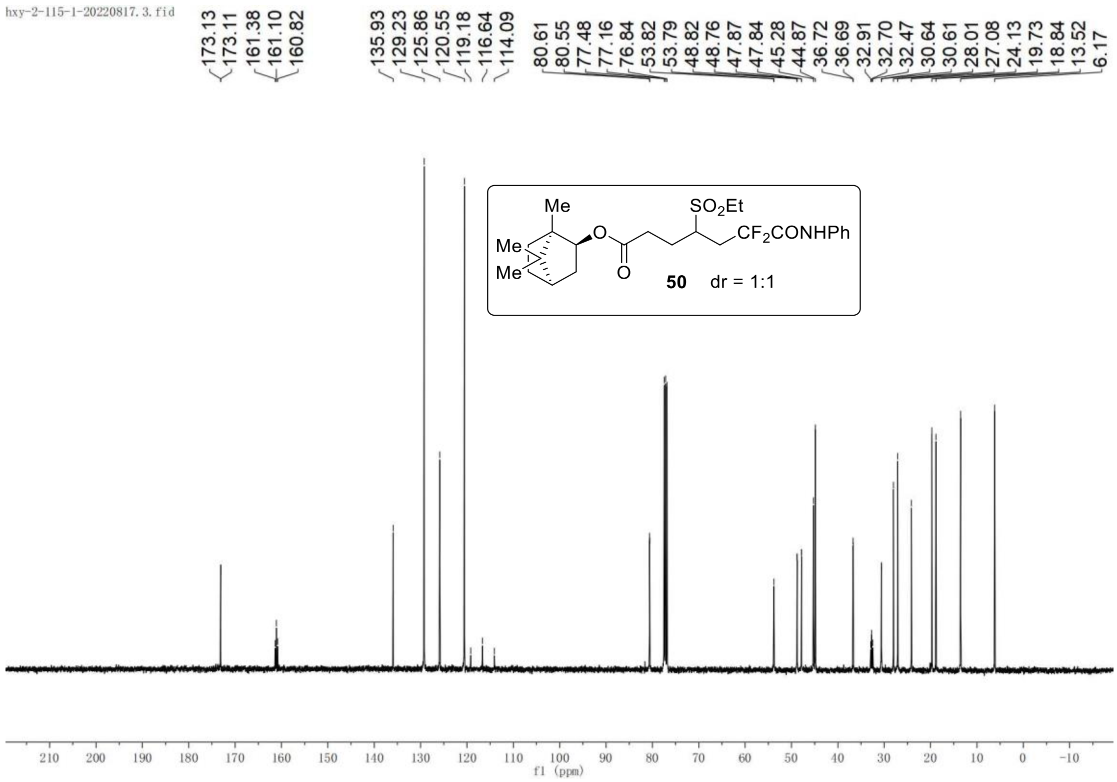

Supplementary Figure 194. ^{13}C NMR (101 MHz, $CDCl_3$) spectra of **50**

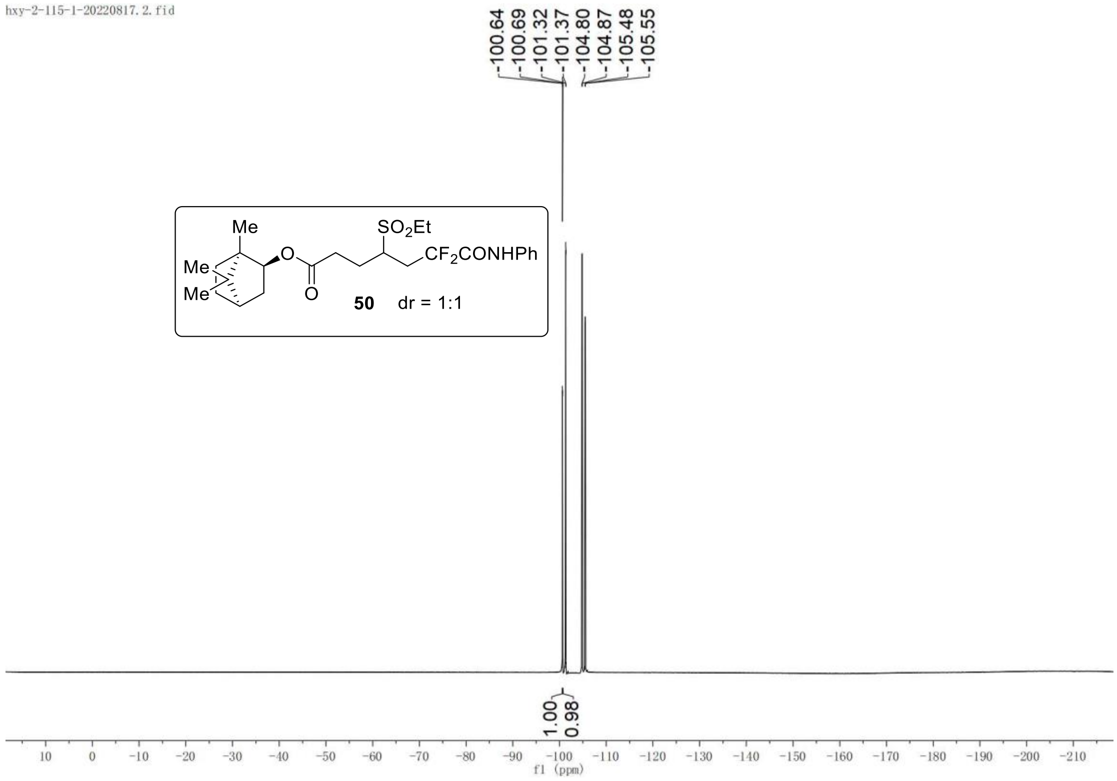

Supplementary Figure 195. ^{19}F NMR (376 MHz, $CDCl_3$) spectra of **50**

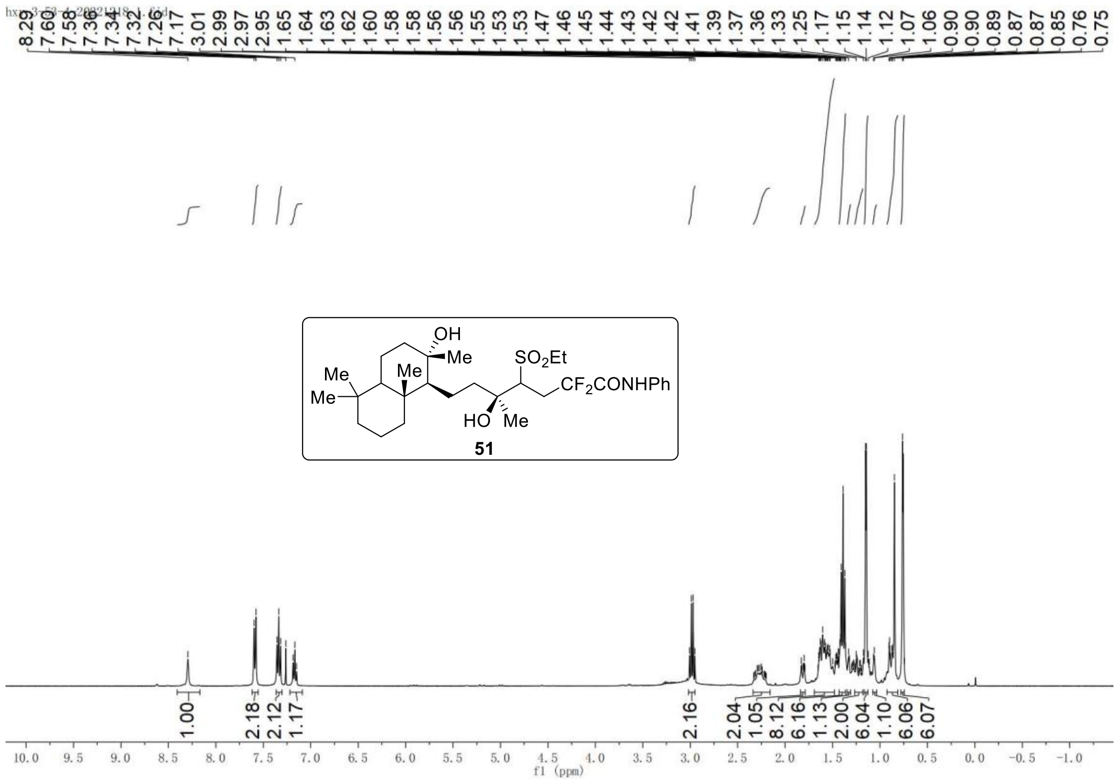

Supplementary Figure 196. ^{1}H NMR (400 MHz, $CDCl_3$) spectra of **51**

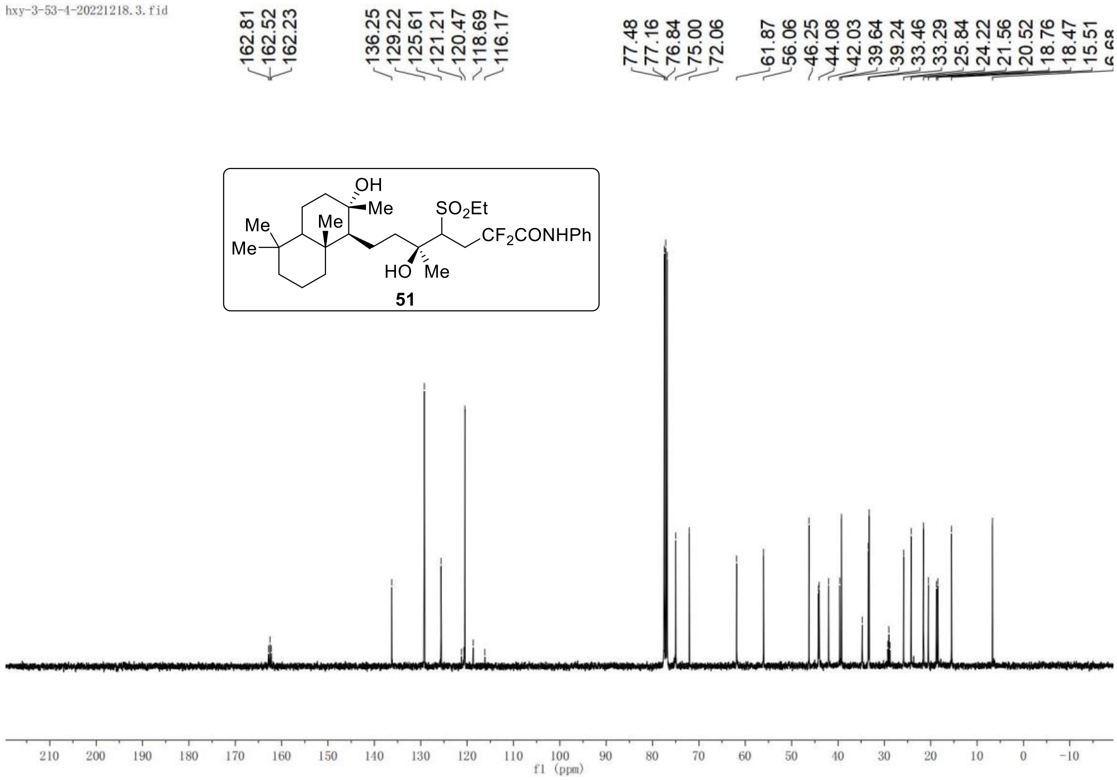

Supplementary Figure 197. ^{13}C NMR (101 MHz, $CDCl_3$) spectra of **51**

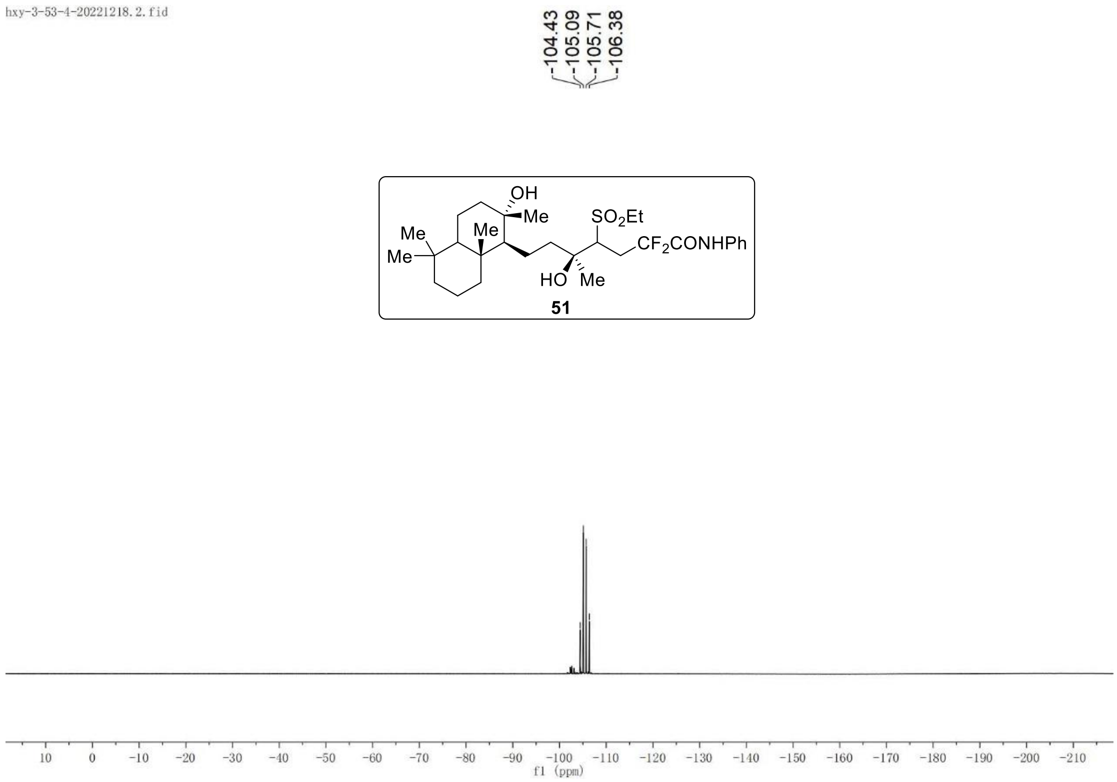

Supplementary Figure 198. ^{19}F NMR (376 MHz, $CDCl_3$) spectra of **51**

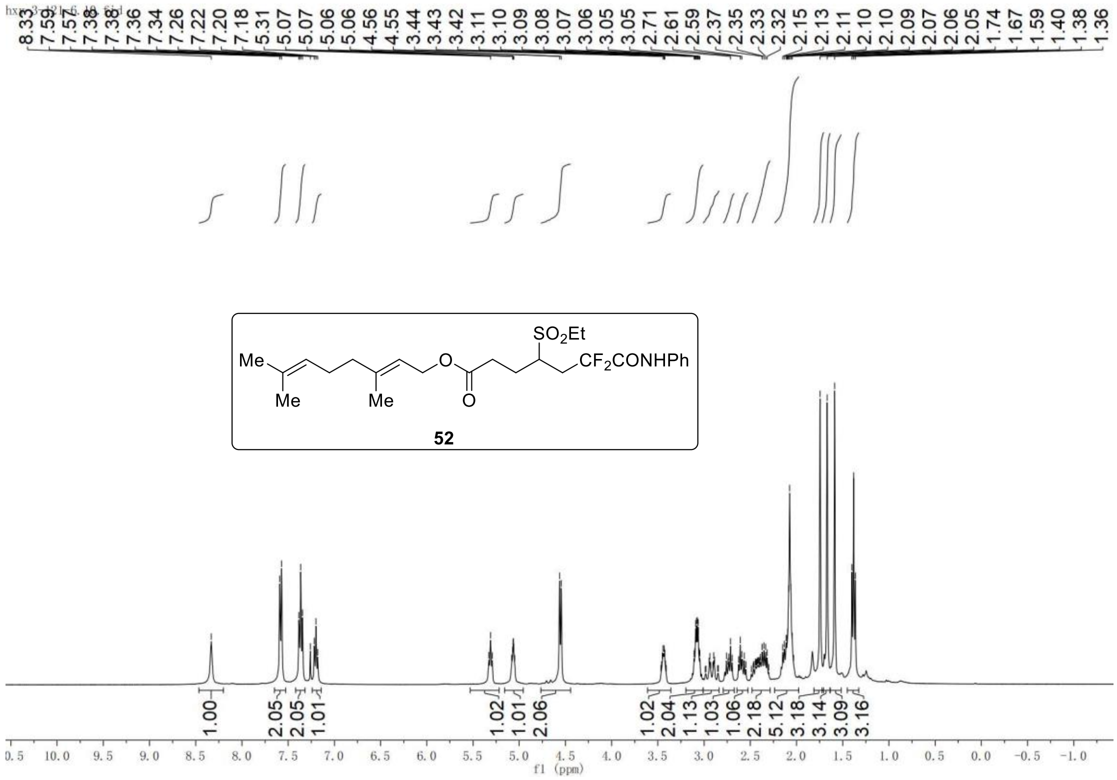

Supplementary Figure 199. ^{1}H NMR (400 MHz, $CDCl_3$) spectra of **52**

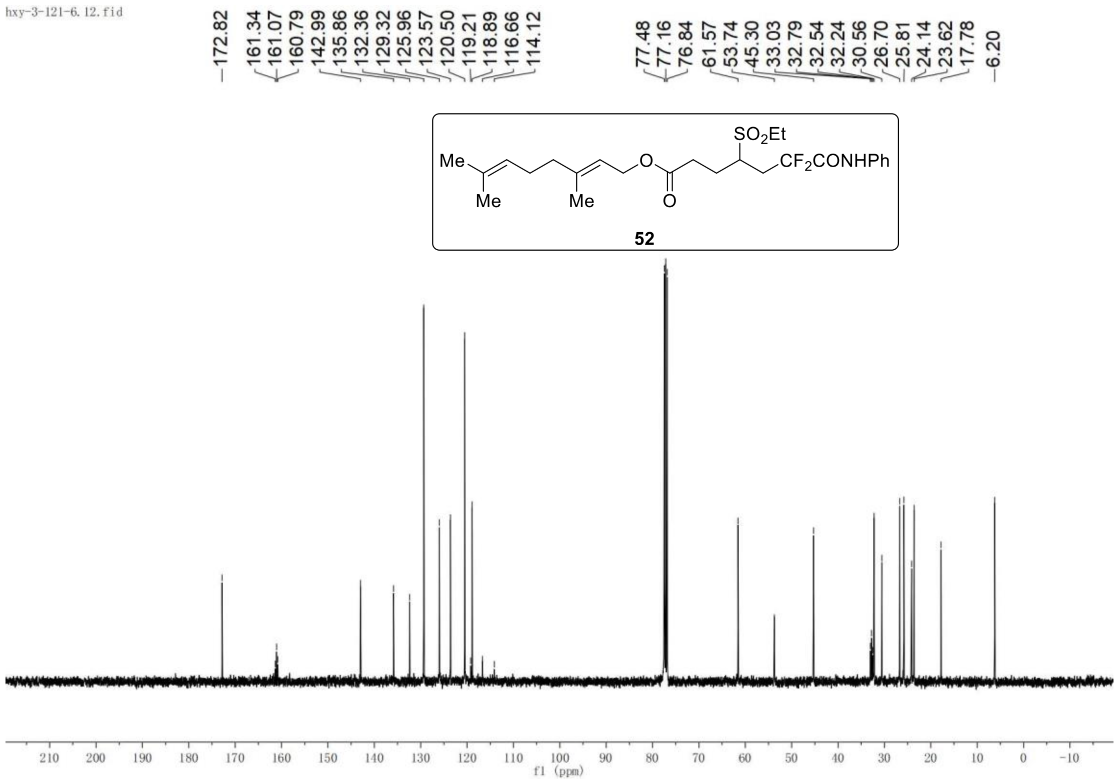

Supplementary Figure 200. ^{13}C NMR (101 MHz, $CDCl_3$) spectra of **52**

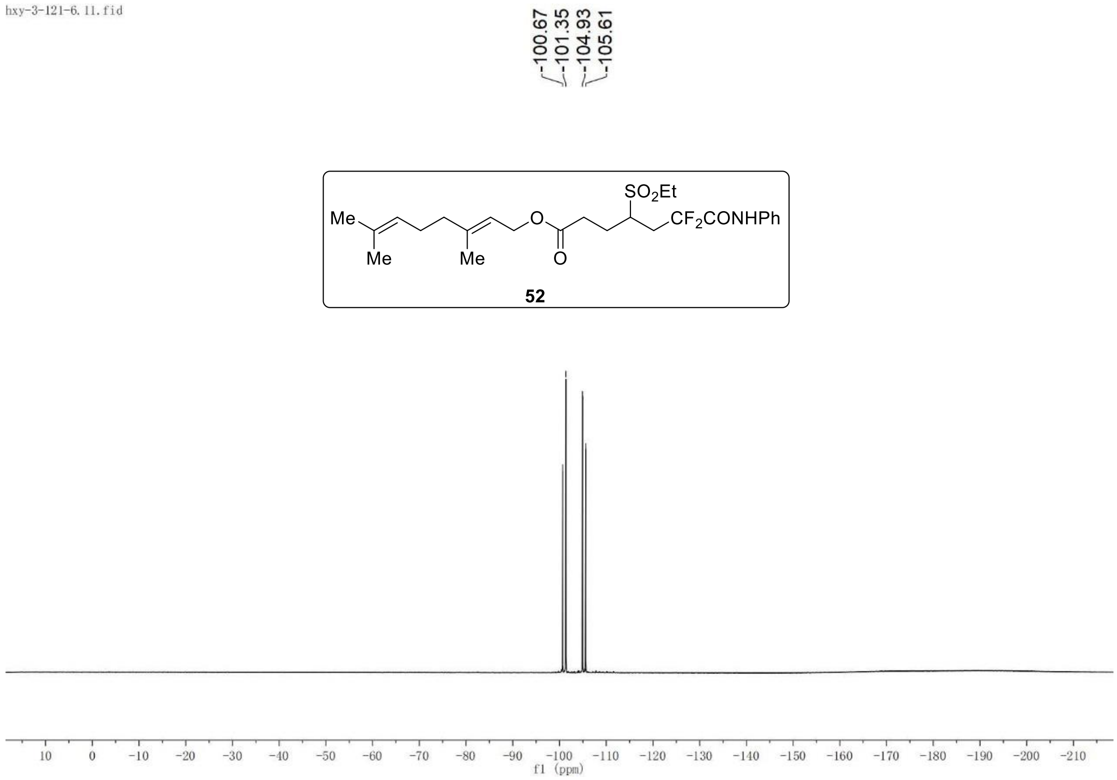

Supplementary Figure 201. ^{19}F NMR (376 MHz, $CDCl_3$) spectra of **52**

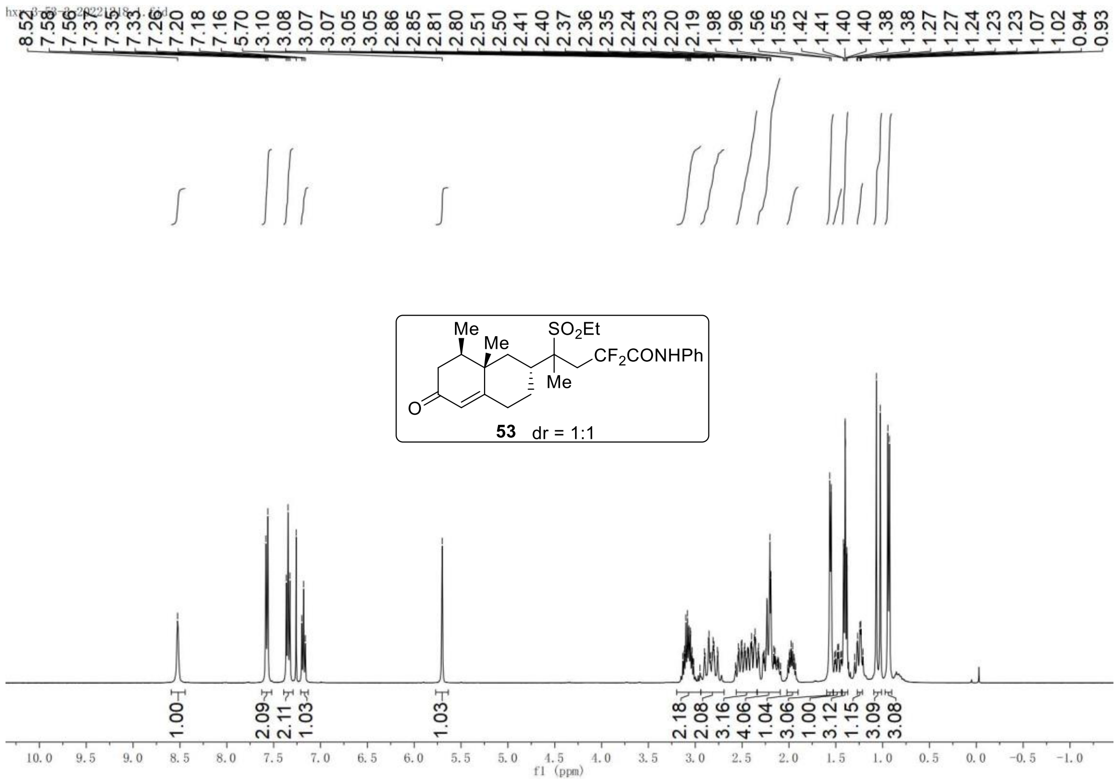

Supplementary Figure 202. ^{1}H NMR (400 MHz, $CDCl_3$) spectra of **53**

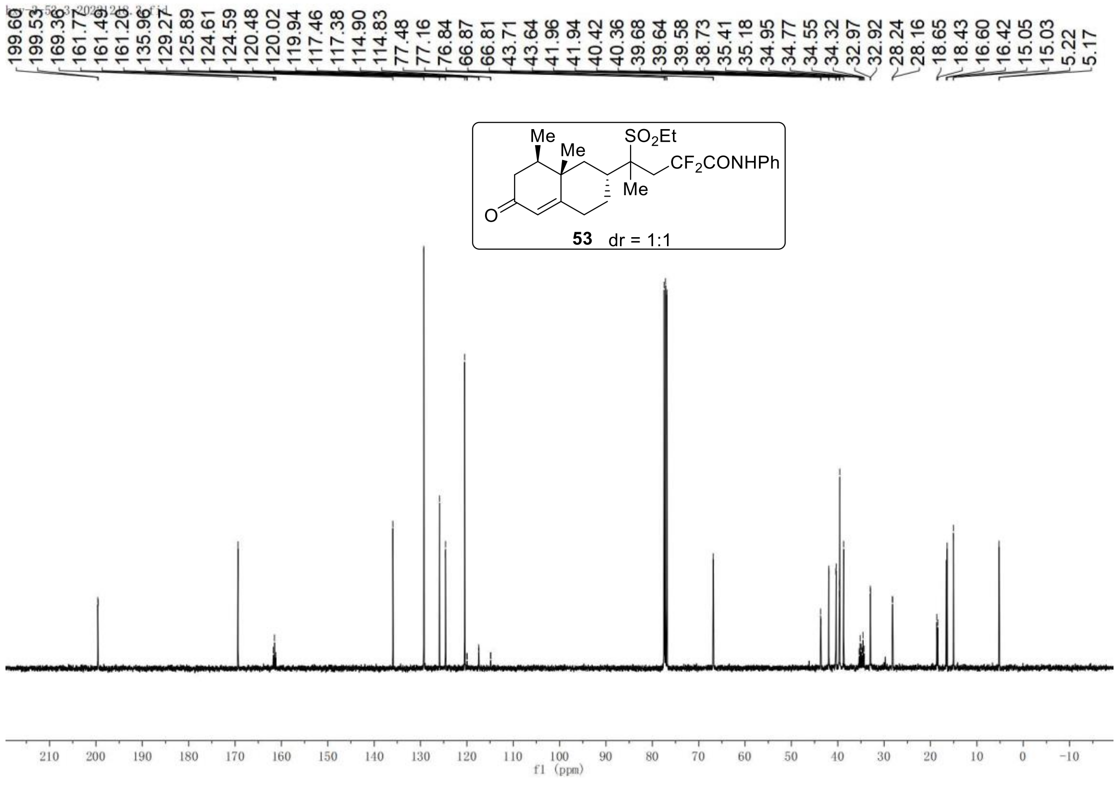

Supplementary Figure 203. ^{13}C NMR (101 MHz, $CDCl_3$) spectra of **53**

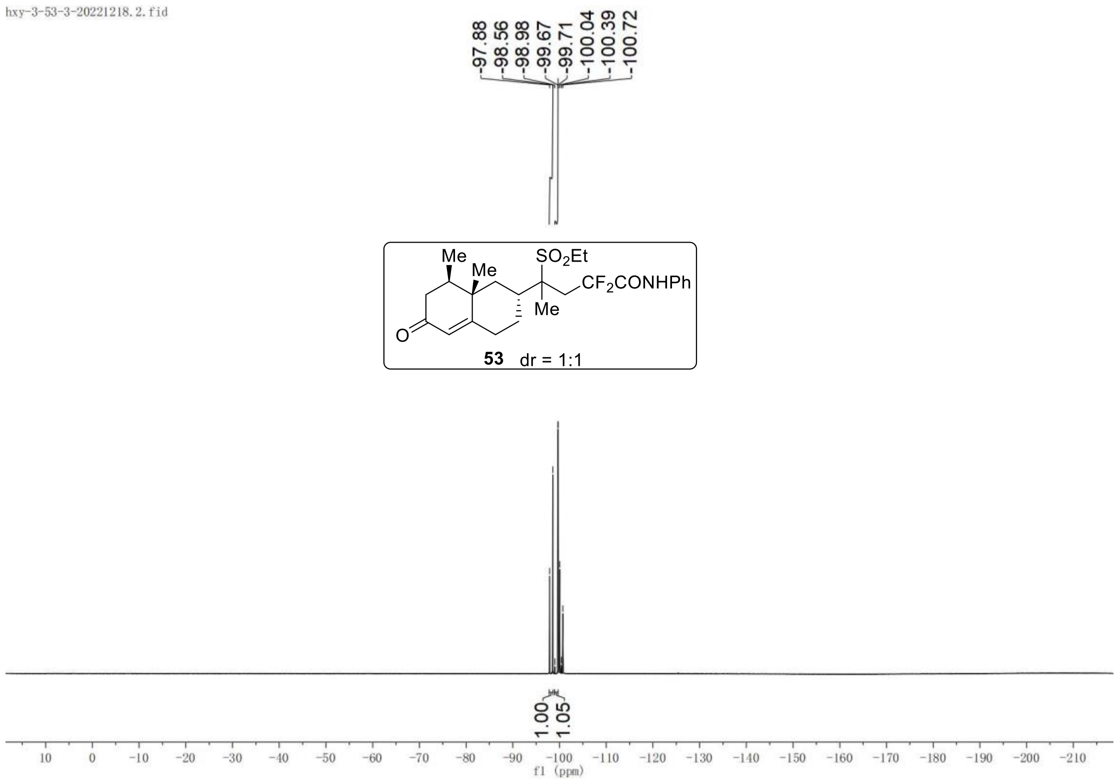

Supplementary Figure 204. ^{19}F NMR (376 MHz, $CDCl_3$) spectra of **53**

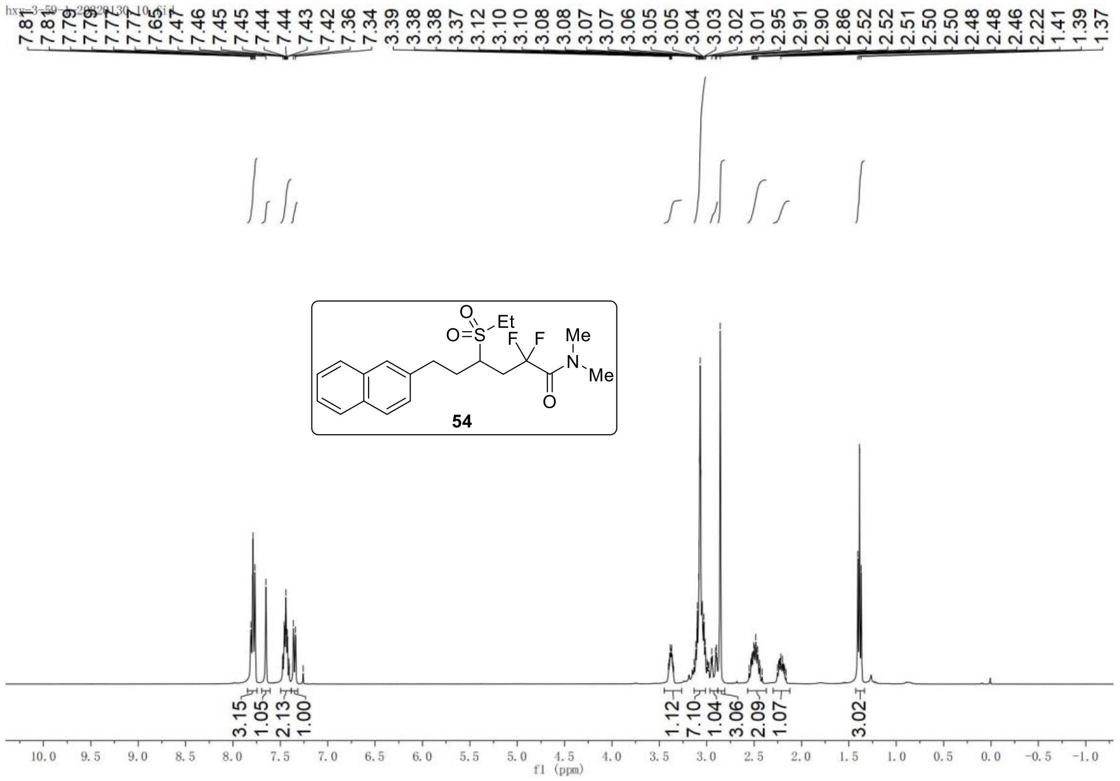

Supplementary Figure 205. ^{1}H NMR (400 MHz, $CDCl_3$) spectra of **54**

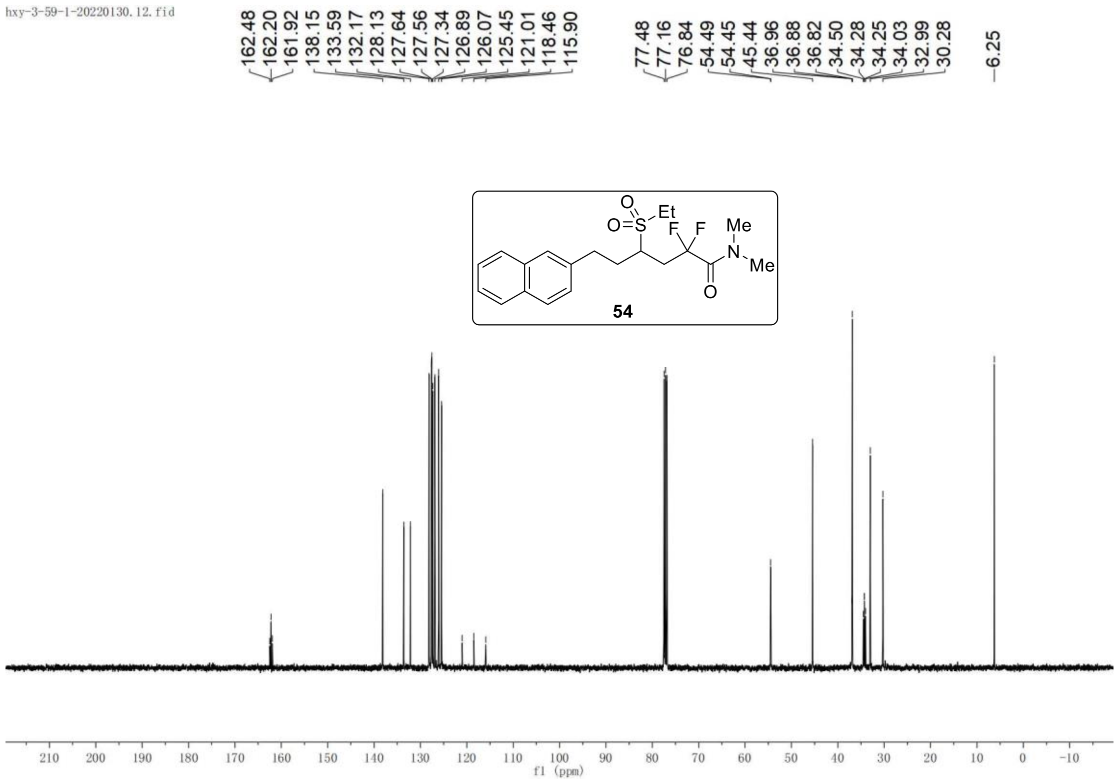

Supplementary Figure 206. ^{13}C NMR (101 MHz, $CDCl_3$) spectra of **54**

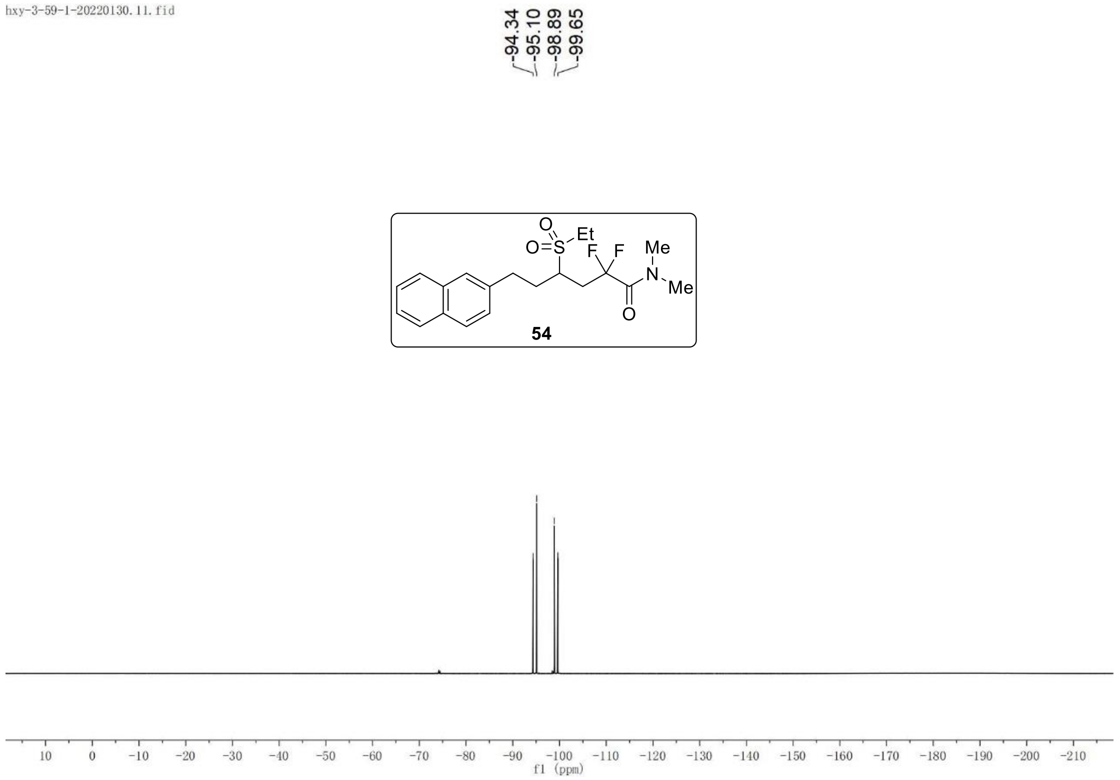

Supplementary Figure 207. ^{19}F NMR (376 MHz, $CDCl_3$) spectra of **54**

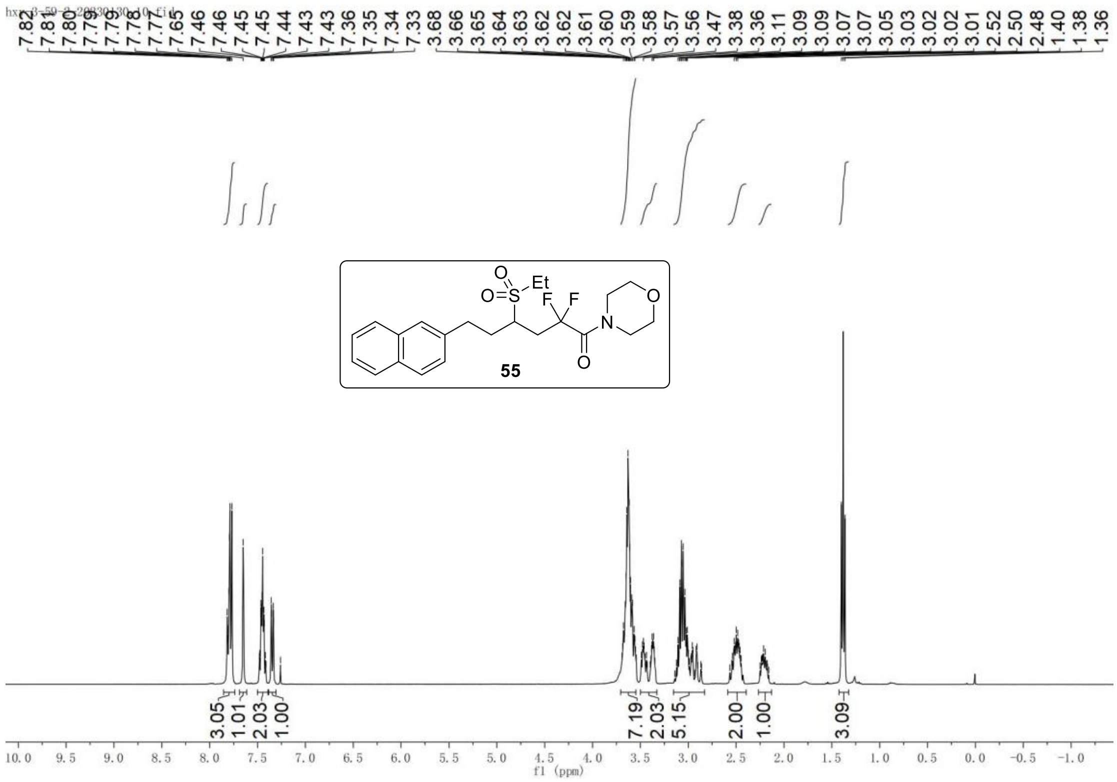

Supplementary Figure 208. ^{1}H NMR (400 MHz, $CDCl_3$) spectra of **55**

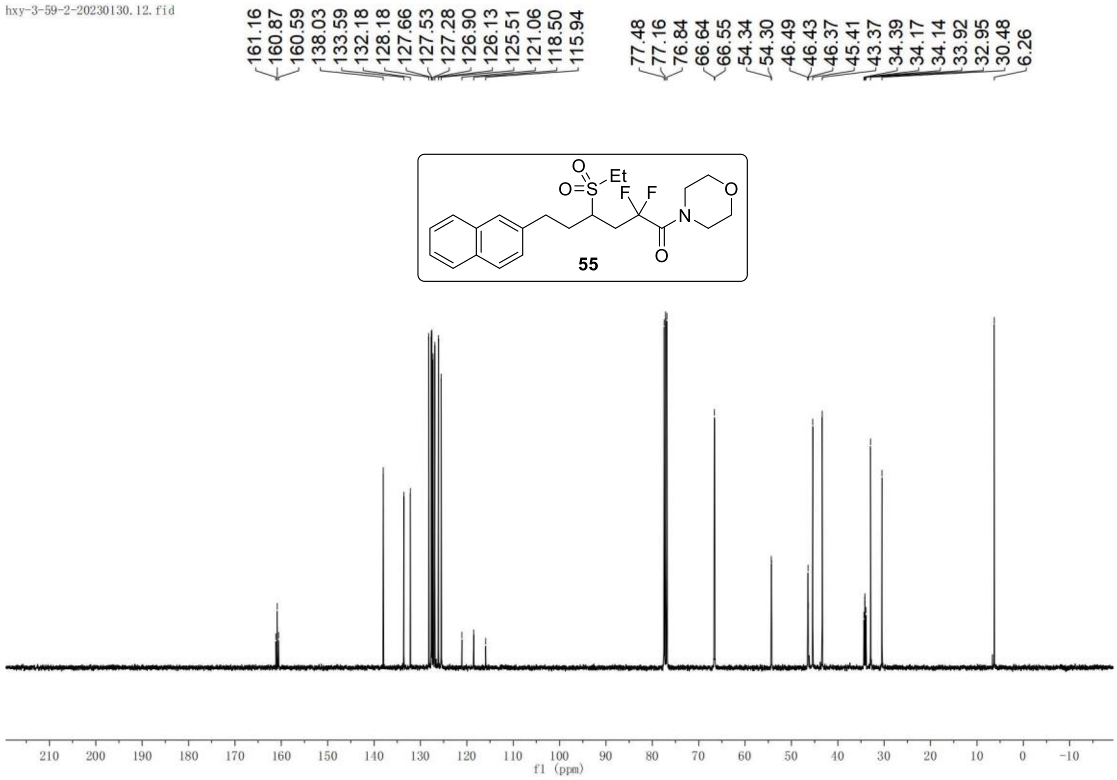

Supplementary Figure 209. ^{13}C NMR (101 MHz, $CDCl_3$) spectra of **55**

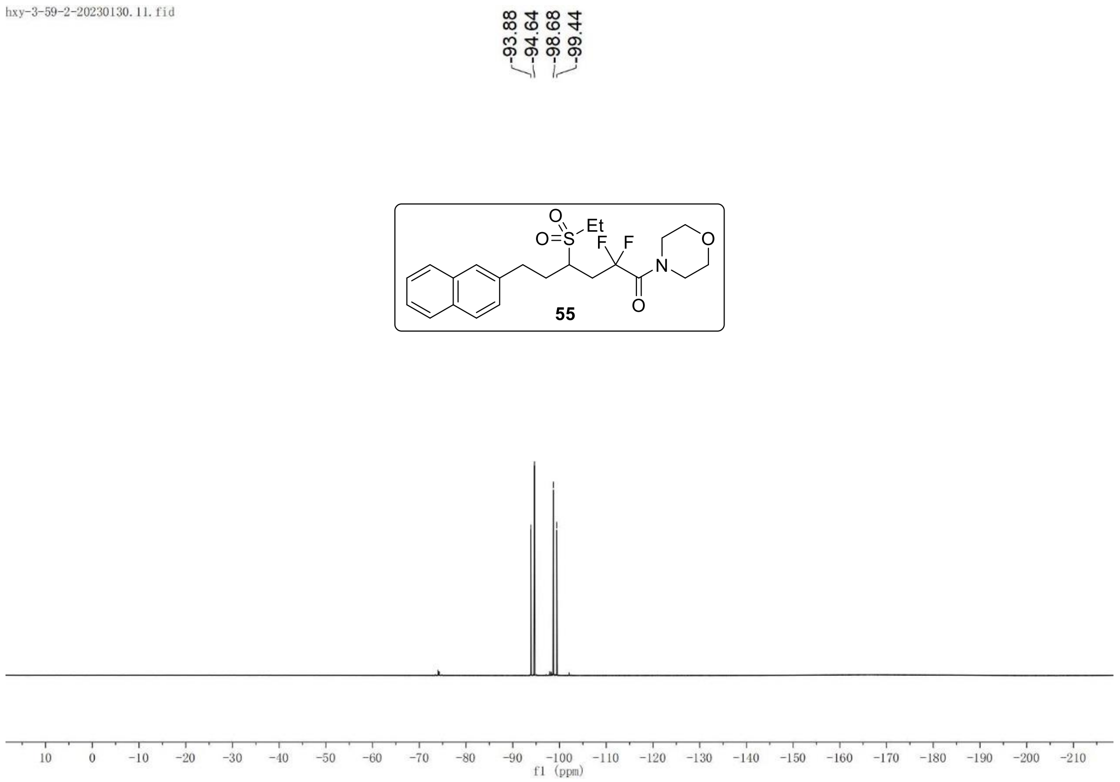

Supplementary Figure 210. ^{19}F NMR (376 MHz, $CDCl_3$) spectra of **55**

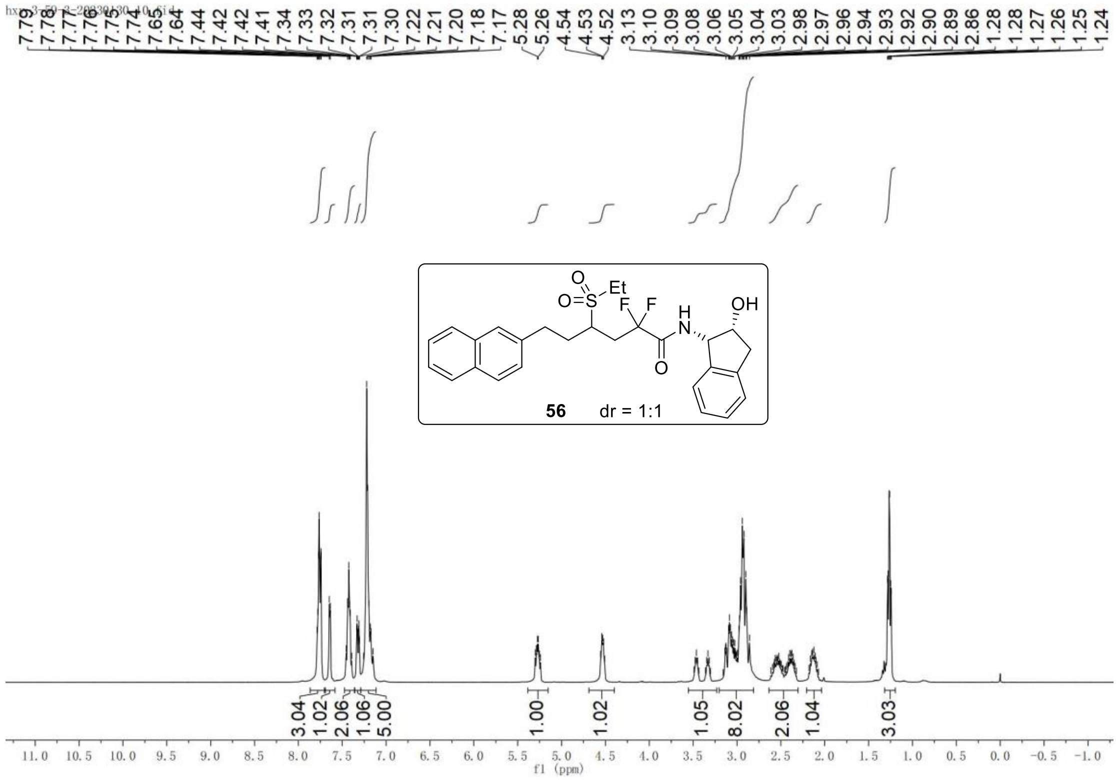

Supplementary Figure 211. ^{1}H NMR (400 MHz, $CDCl_3$) spectra of **56**

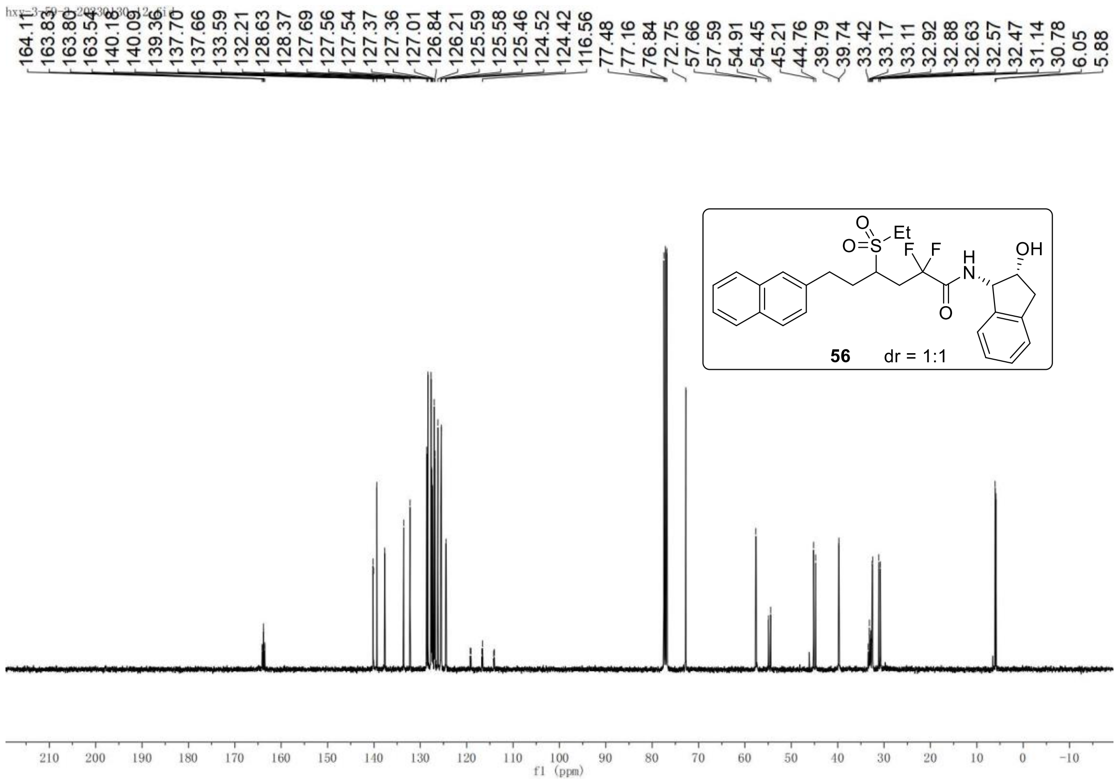

Supplementary Figure 212. ^{13}C NMR (101 MHz, $CDCl_3$) spectra of **56**

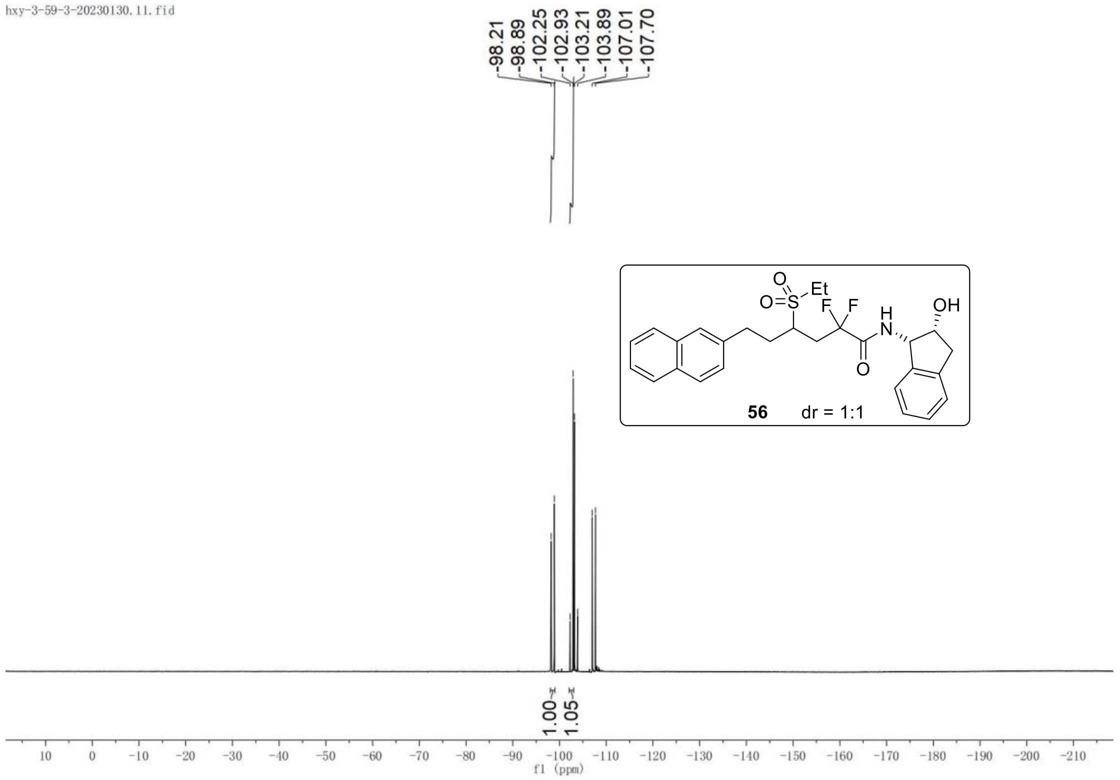

Supplementary Figure 213. ^{19}F NMR (376 MHz, $CDCl_3$) spectra of **56**

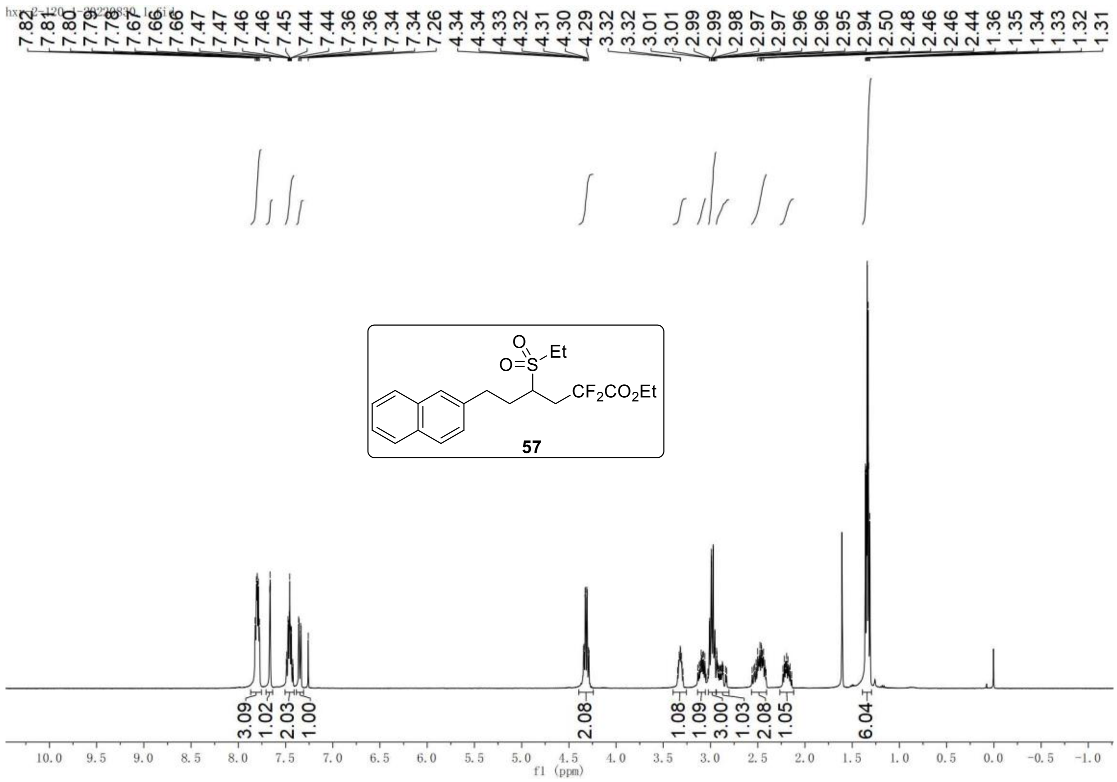

Supplementary Figure 214. ^{1}H NMR (400 MHz, $CDCl_3$) spectra of **57**

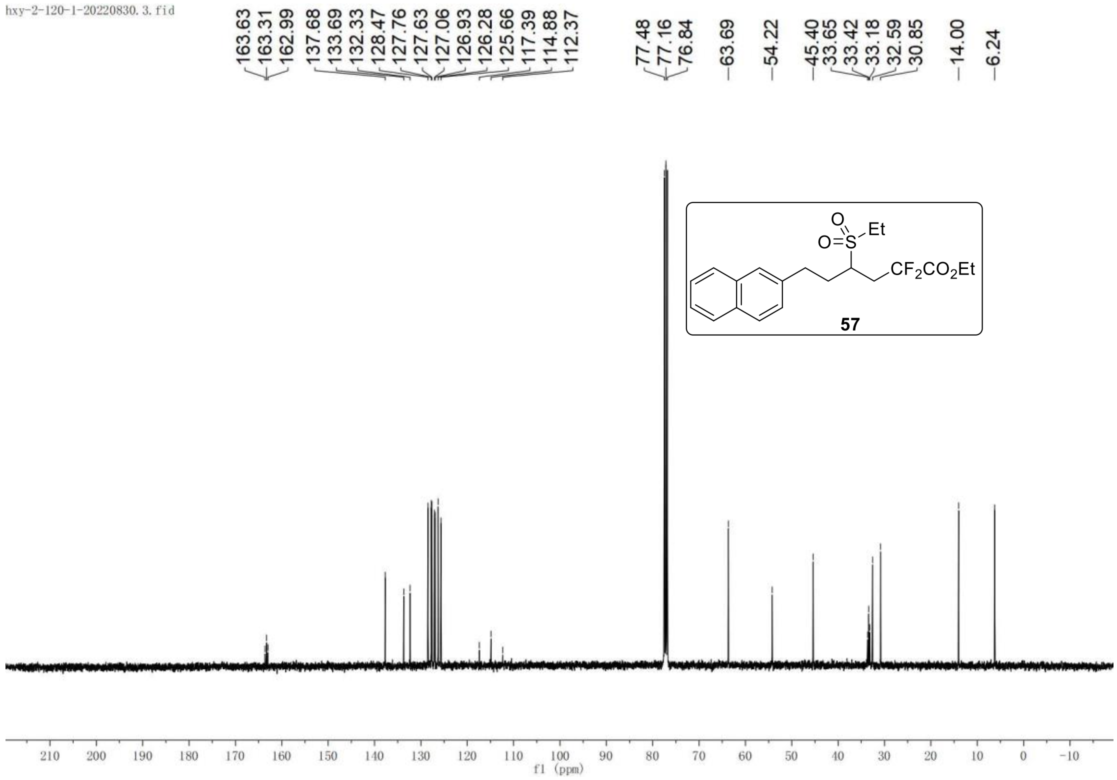

Supplementary Figure 215. ^{13}C NMR (101 MHz, $CDCl_3$) spectra of **57**

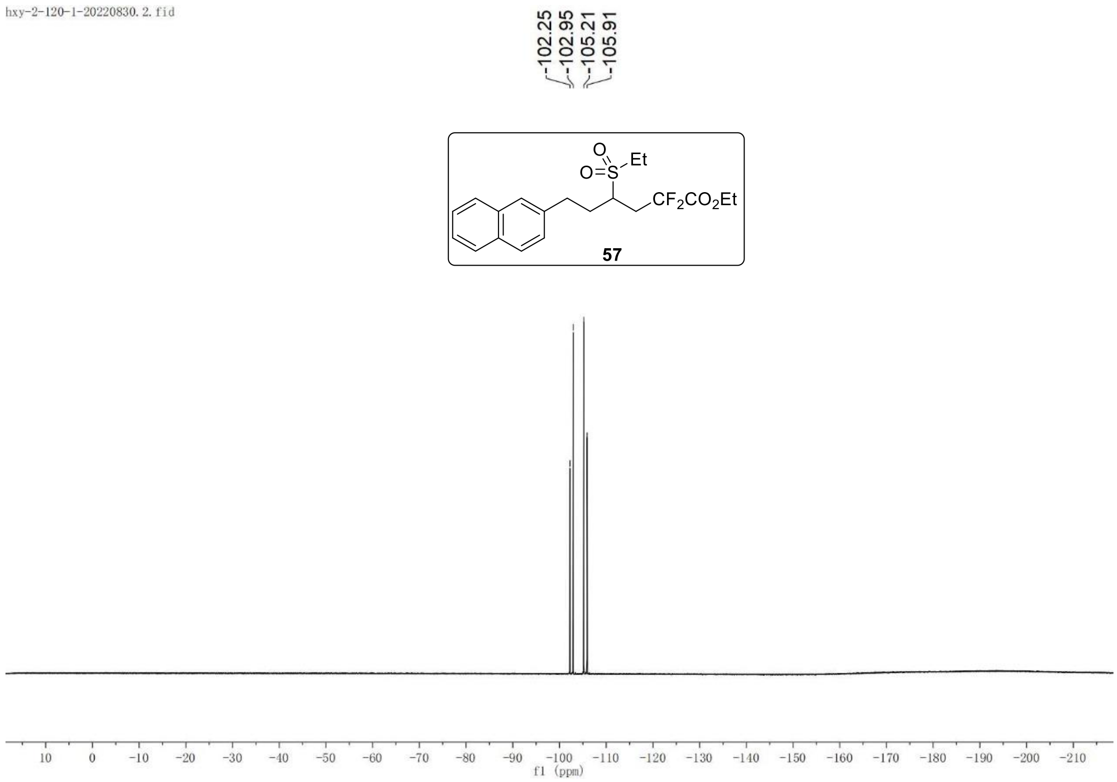

Supplementary Figure 216. ^{19}F NMR (376 MHz, $CDCl_3$) spectra of **57**

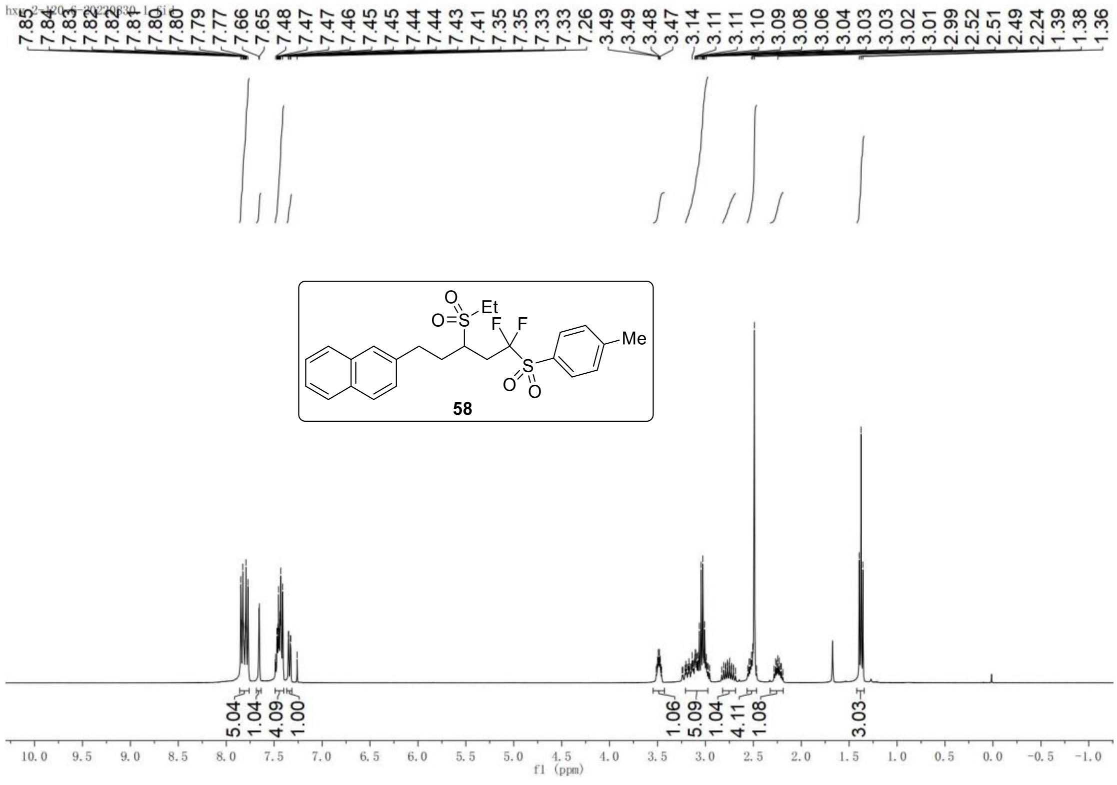

Supplementary Figure 217. ^{1}H NMR (400 MHz, $CDCl_3$) spectra of **58**

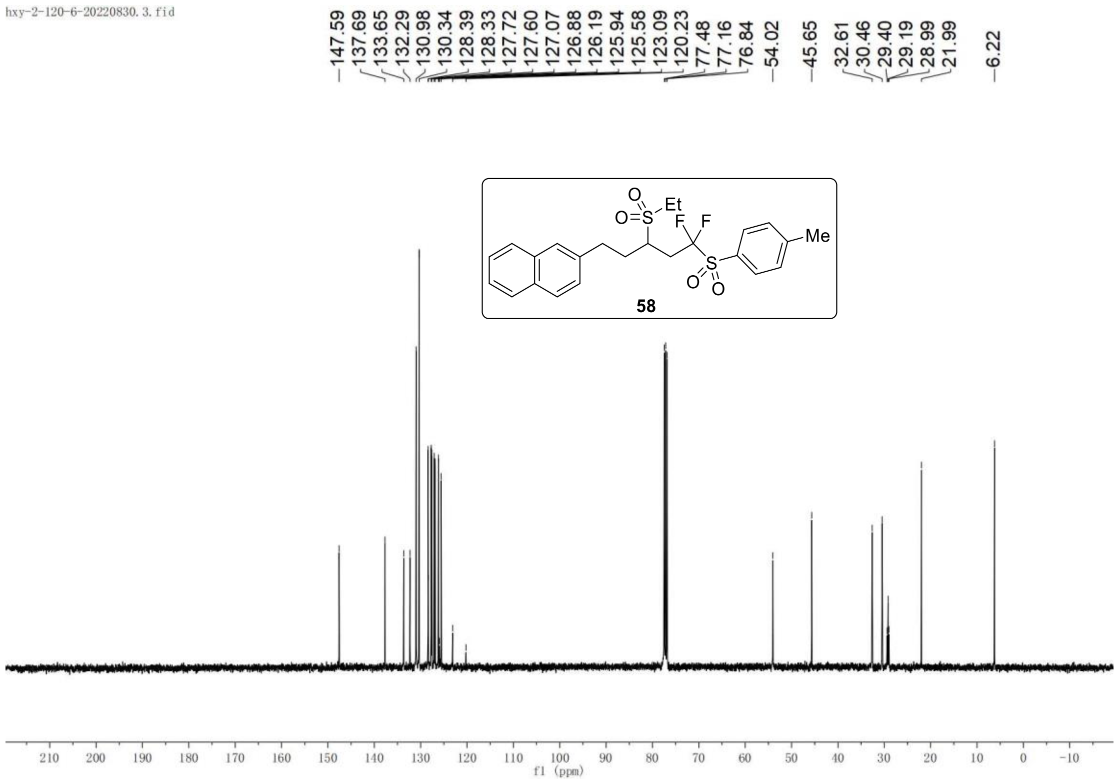

Supplementary Figure 218. ^{13}C NMR (101 MHz, $CDCl_3$) spectra of **58**

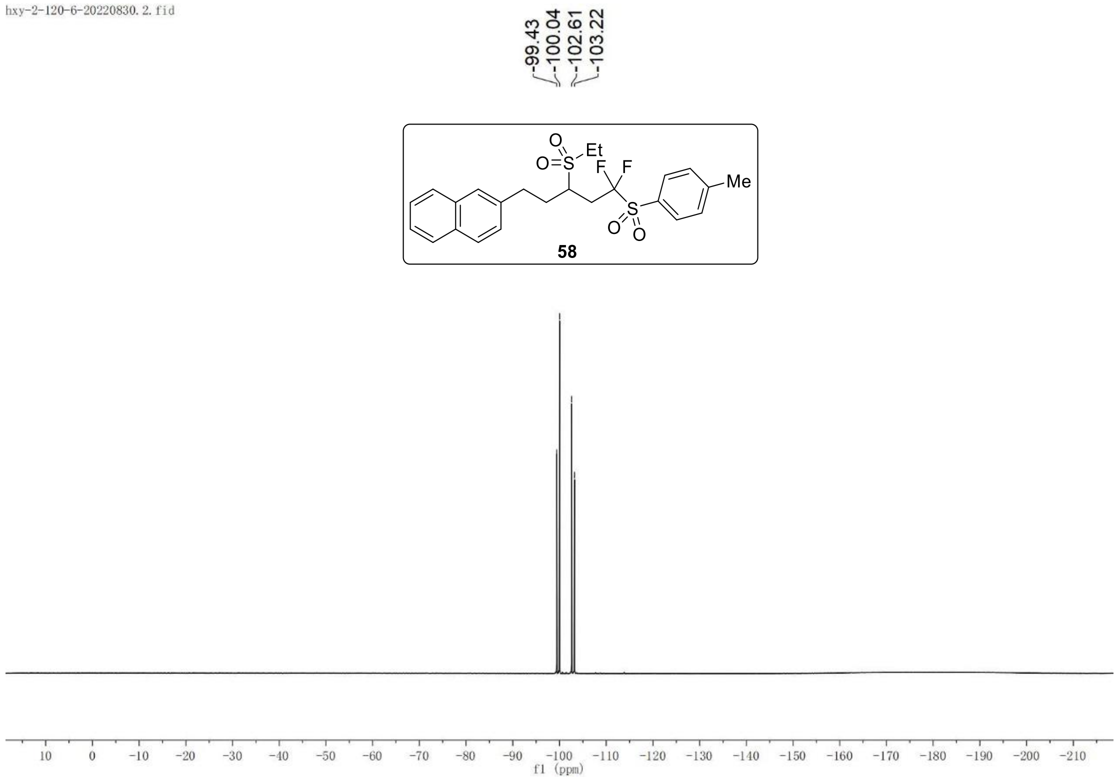

Supplementary Figure 219. ^{19}F NMR (376 MHz, $CDCl_3$) spectra of **58**

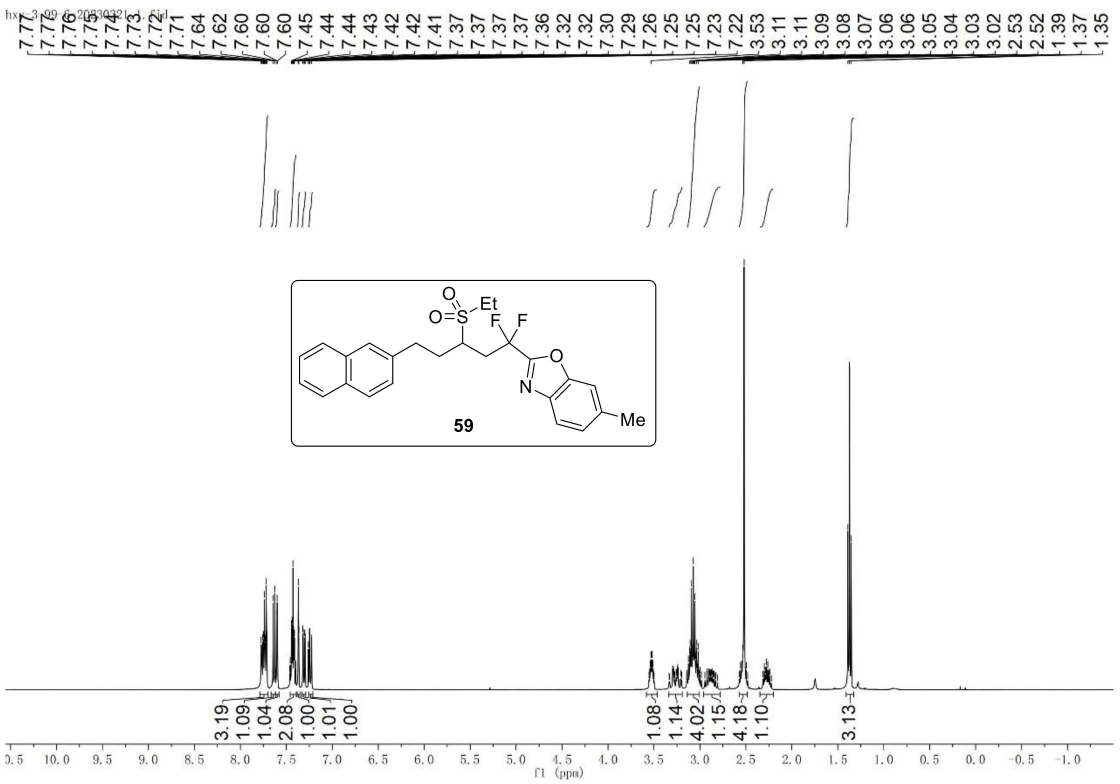

Supplementary Figure 220. ^{1}H NMR (400 MHz, $CDCl_3$) spectra of **59**

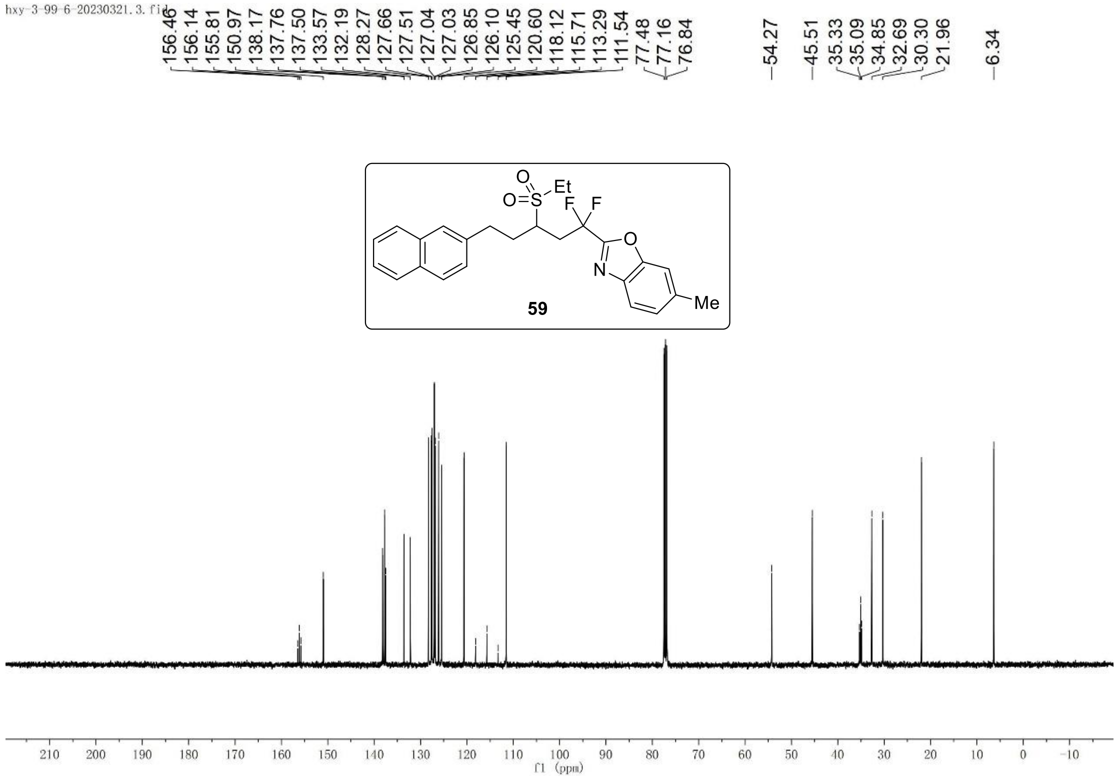

Supplementary Figure 221. ^{13}C NMR (101 MHz, $CDCl_3$) spectra of **59**

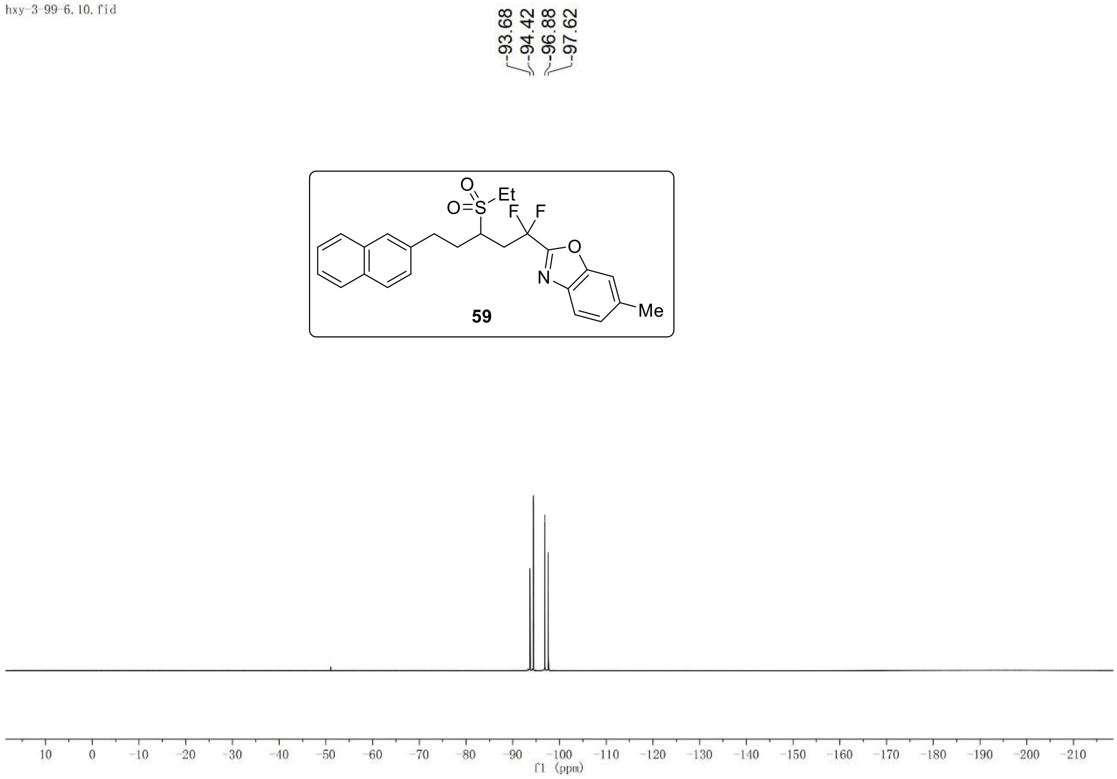

Supplementary Figure 222. ^{19}F NMR (376 MHz, $CDCl_3$) spectra of **59**

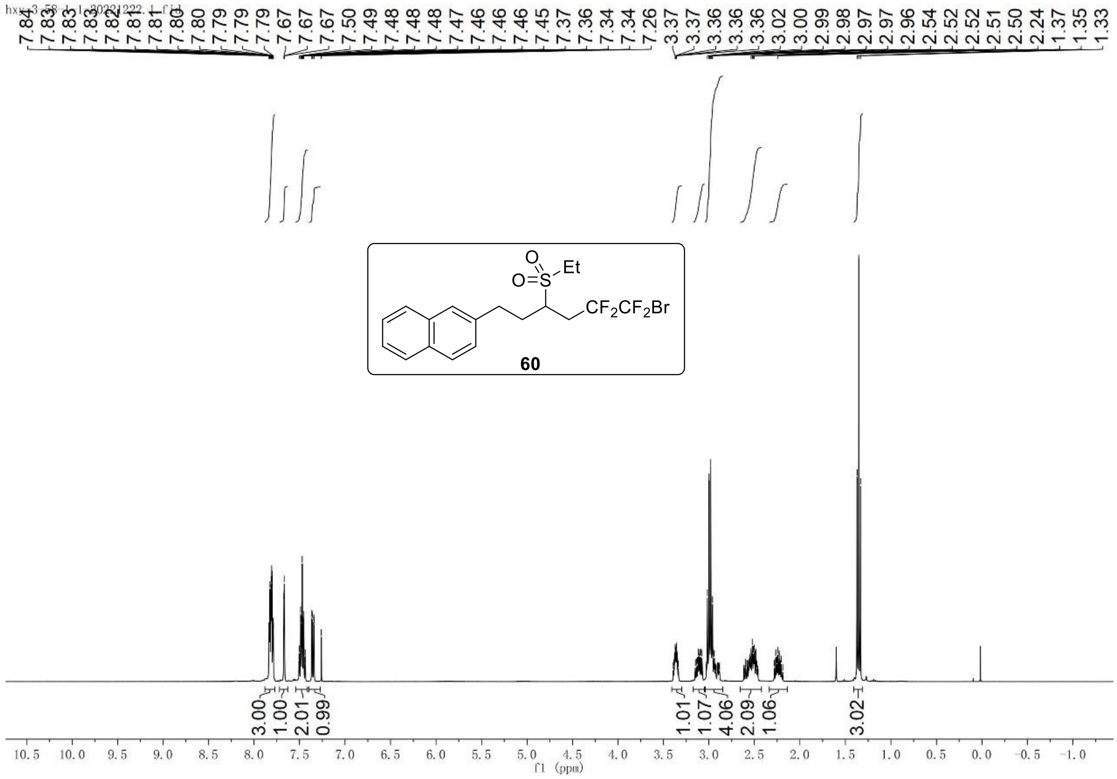

Supplementary Figure 223. ^{1}H NMR (400 MHz, $CDCl_3$) spectra of **60**

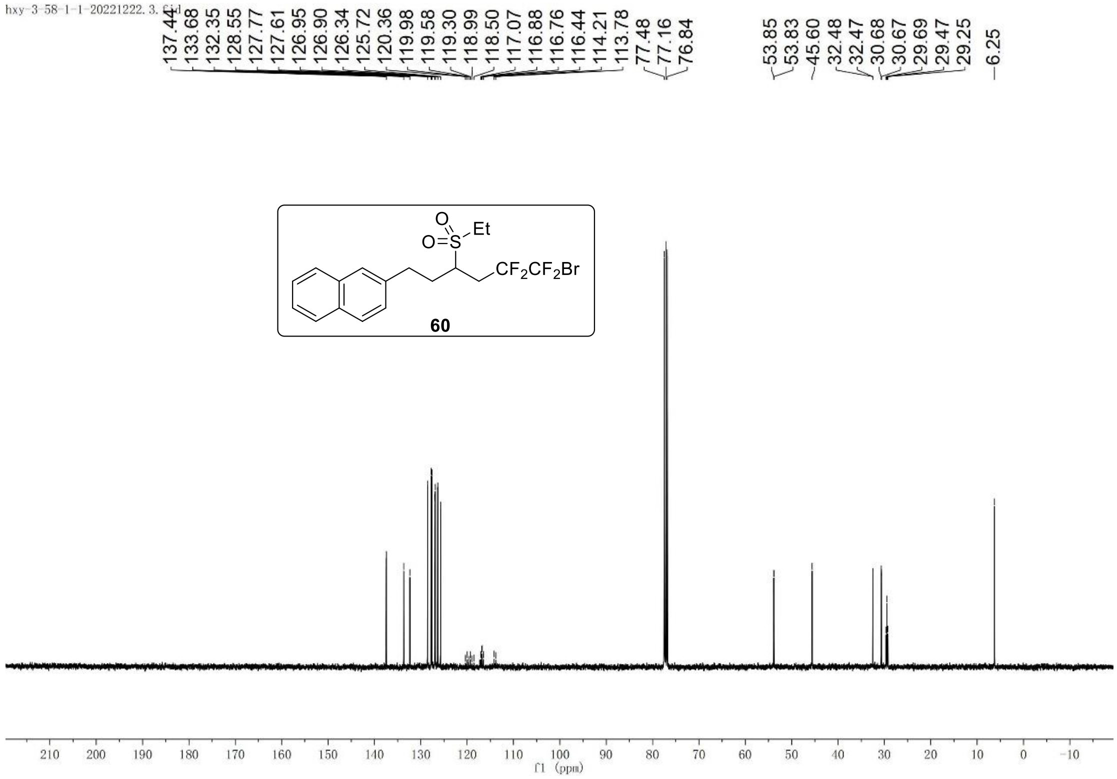

Supplementary Figure 224. ^{13}C NMR (101 MHz, $CDCl_3$) spectra of **60**

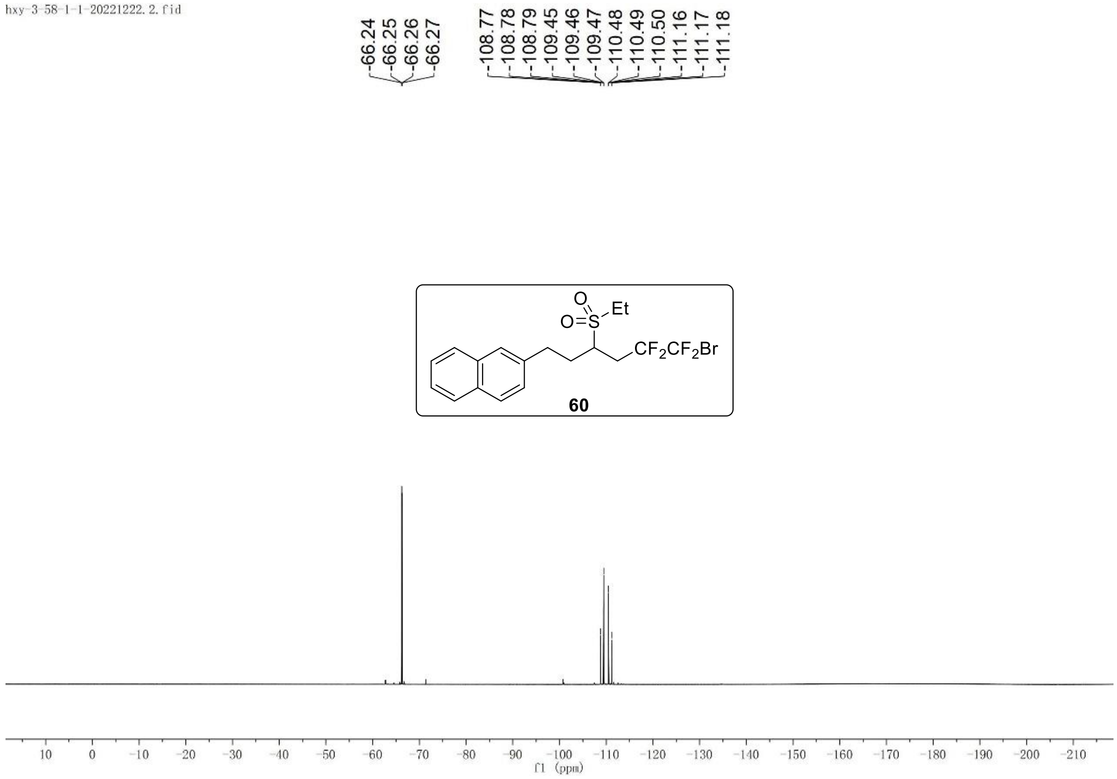

Supplementary Figure 225. ^{19}F NMR (376 MHz, $CDCl_3$) spectra of **60**

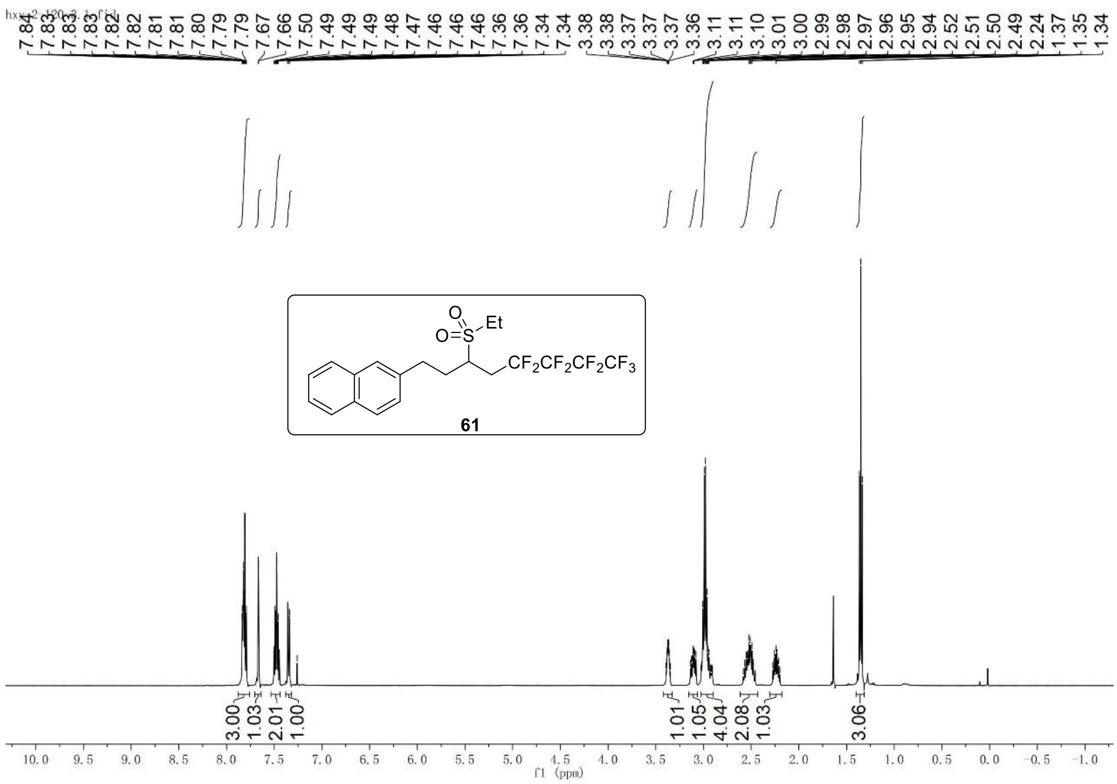

Supplementary Figure 226. ^{1}H NMR (500 MHz, $CDCl_3$) spectra of **61**

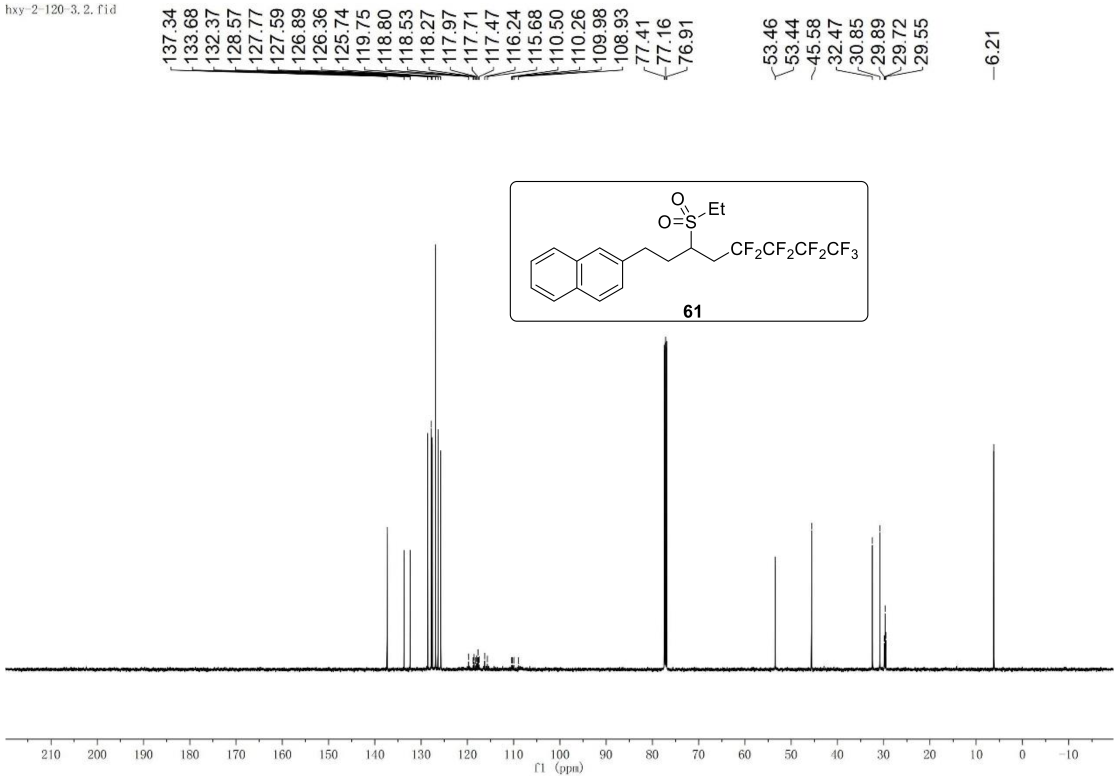

Supplementary Figure 227. ^{13}C NMR (126 MHz, $CDCl_3$) spectra of **61**

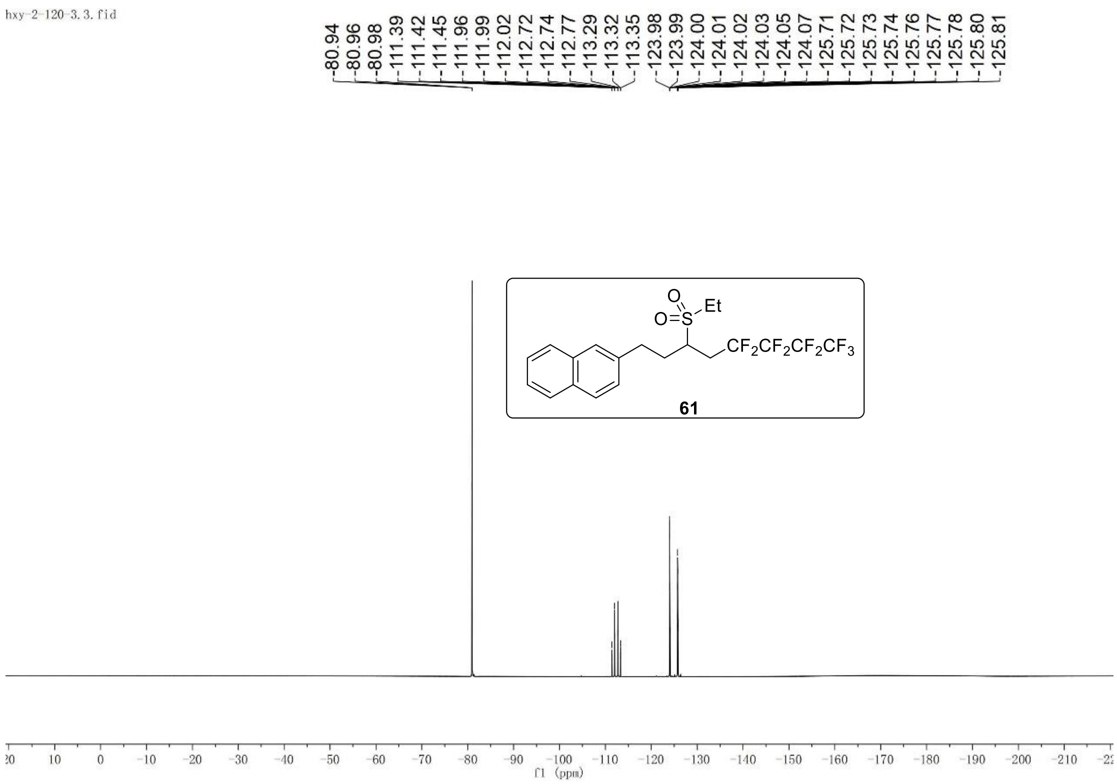

Supplementary Figure 228. ^{19}F NMR (471 MHz, $CDCl_3$) spectra of **61**

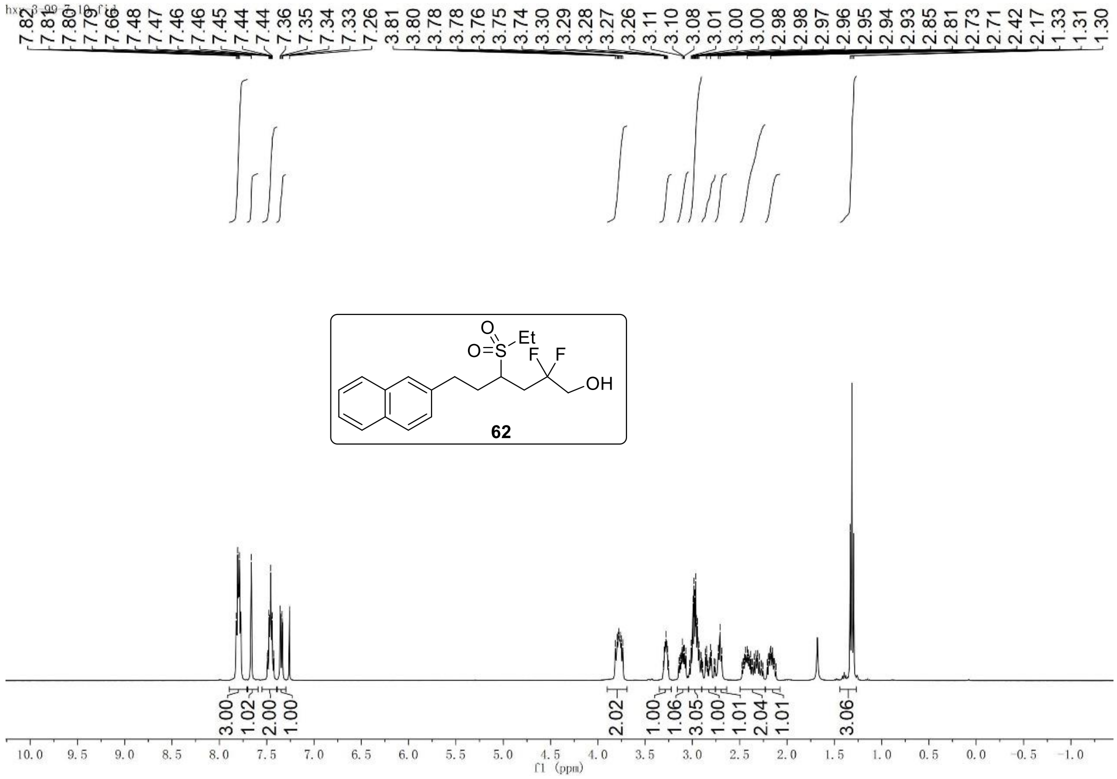

Supplementary Figure 229. ^{1}H NMR (400 MHz, $CDCl_3$) spectra of **62**

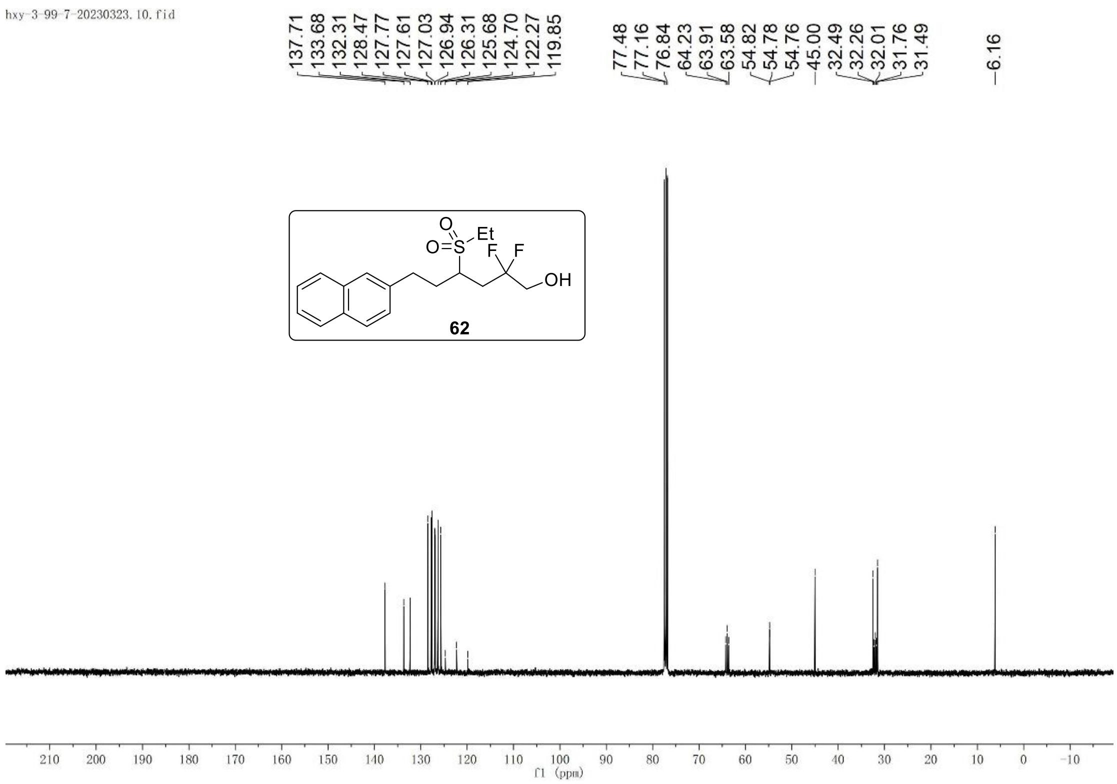

Supplementary Figure 230. ^{13}C NMR (101 MHz, $CDCl_3$) spectra of **62**

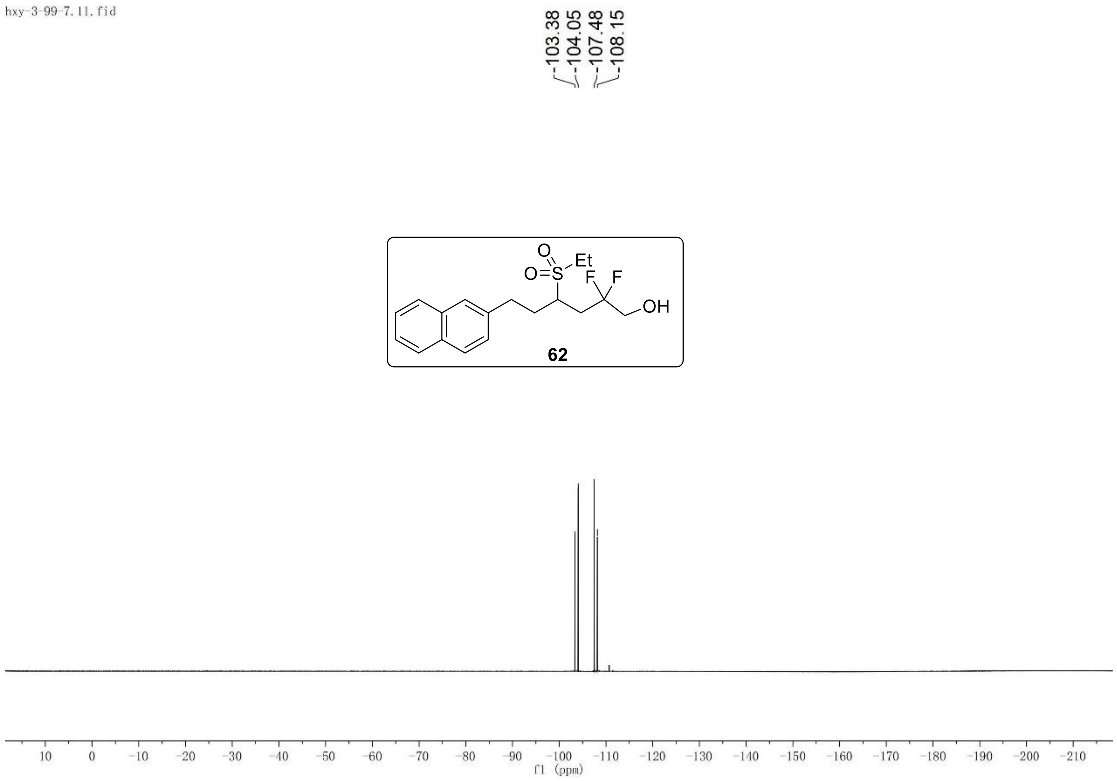

Supplementary Figure 231. ^{19}F NMR (376 MHz, $CDCl_3$) spectra of **62**

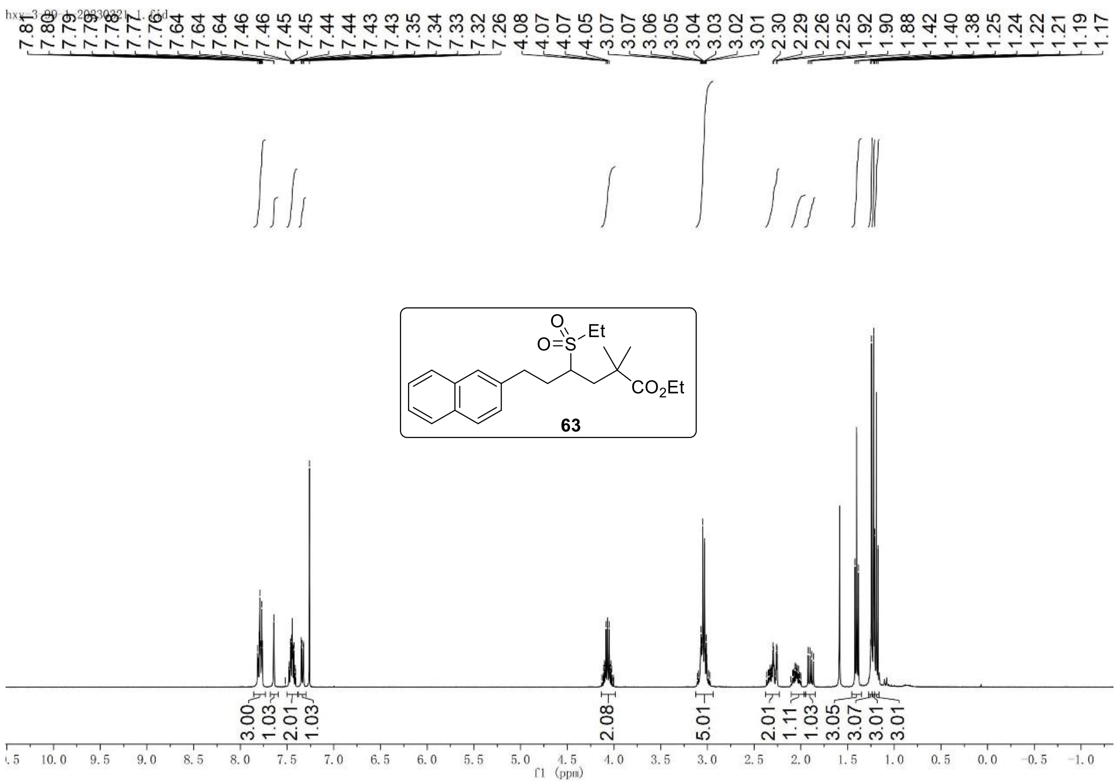

Supplementary Figure 232. ^{1}H NMR (400 MHz, $CDCl_3$) spectra of **63**

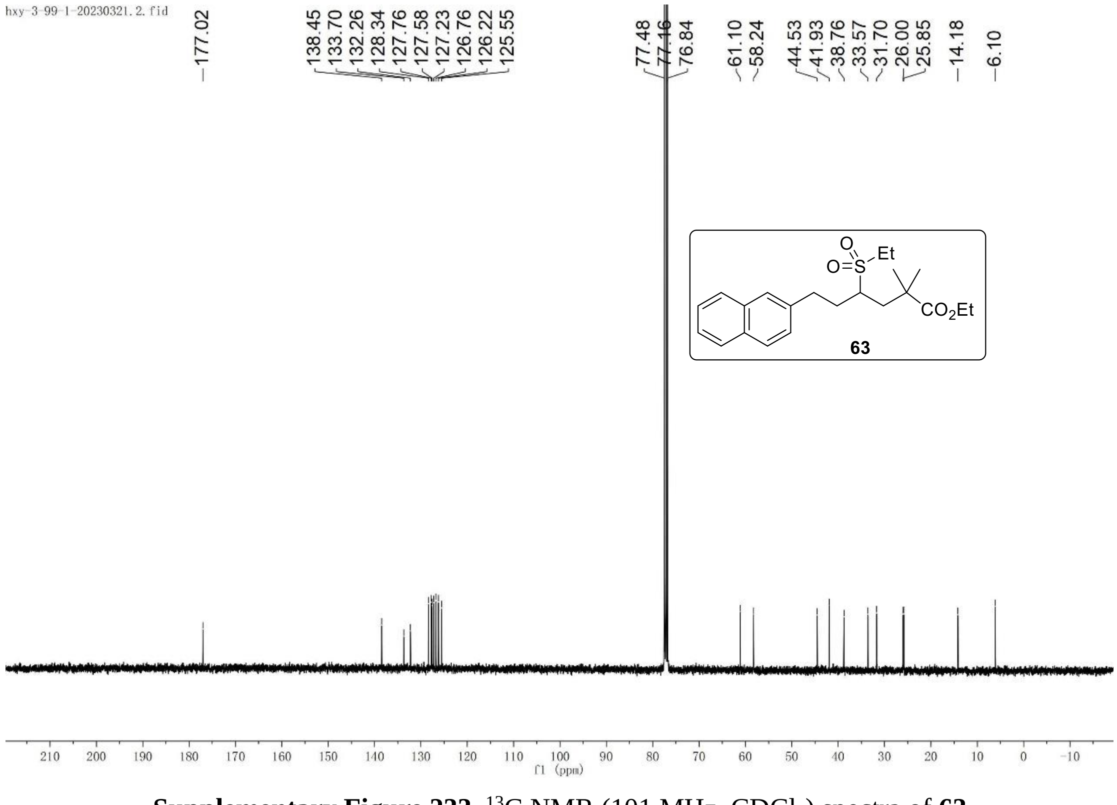

Supplementary Figure 233. ^{13}C NMR (101 MHz, $CDCl_3$) spectra of **63**

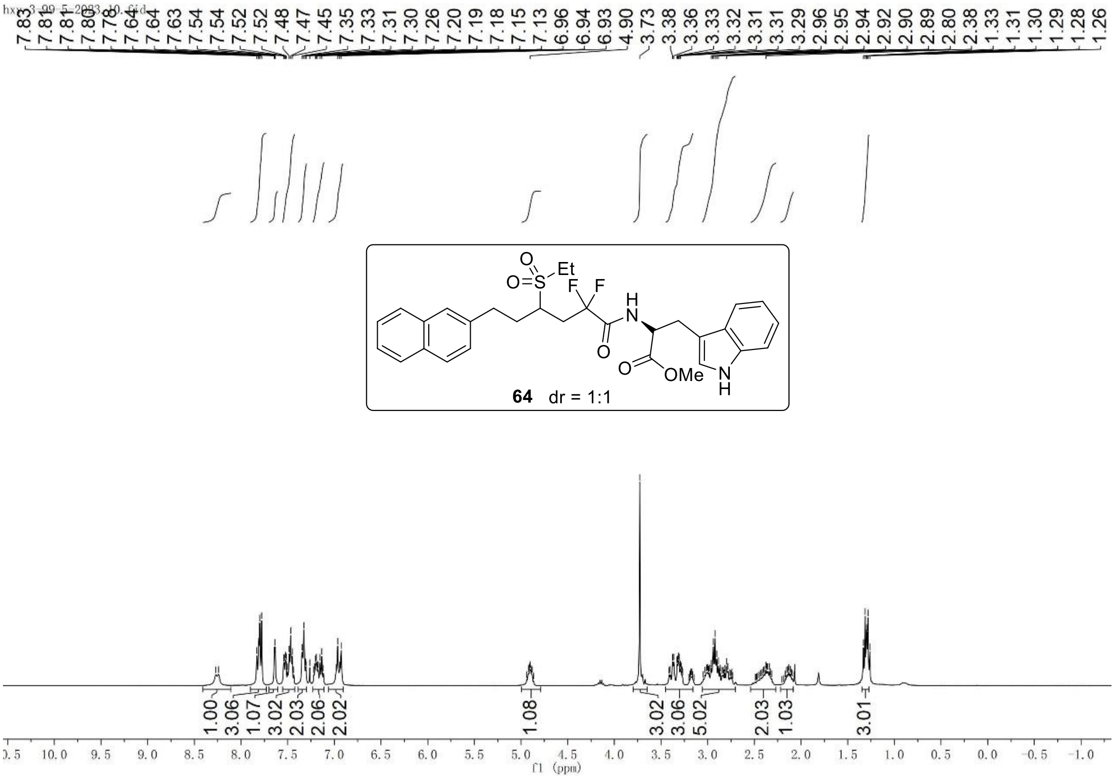

Supplementary Figure 234. ^{1}H NMR (400 MHz, $CDCl_3$) spectra of **64**

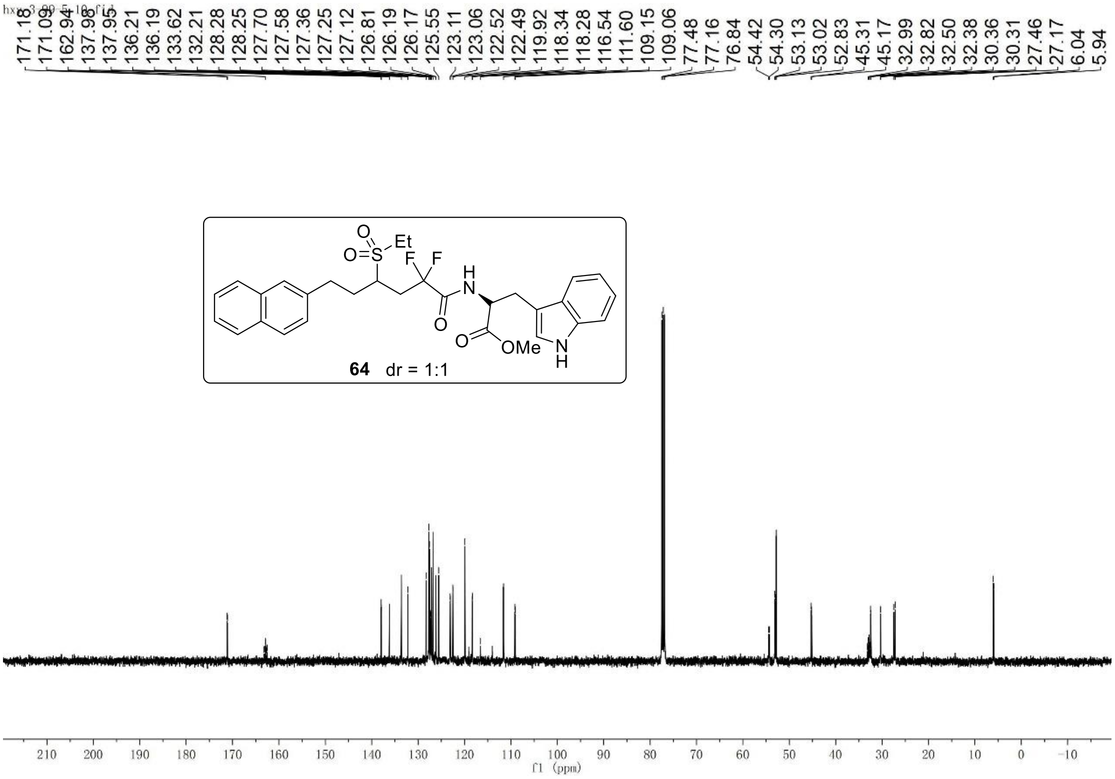

Supplementary Figure 235. ^{13}C NMR (101 MHz, $CDCl_3$) spectra of **64**

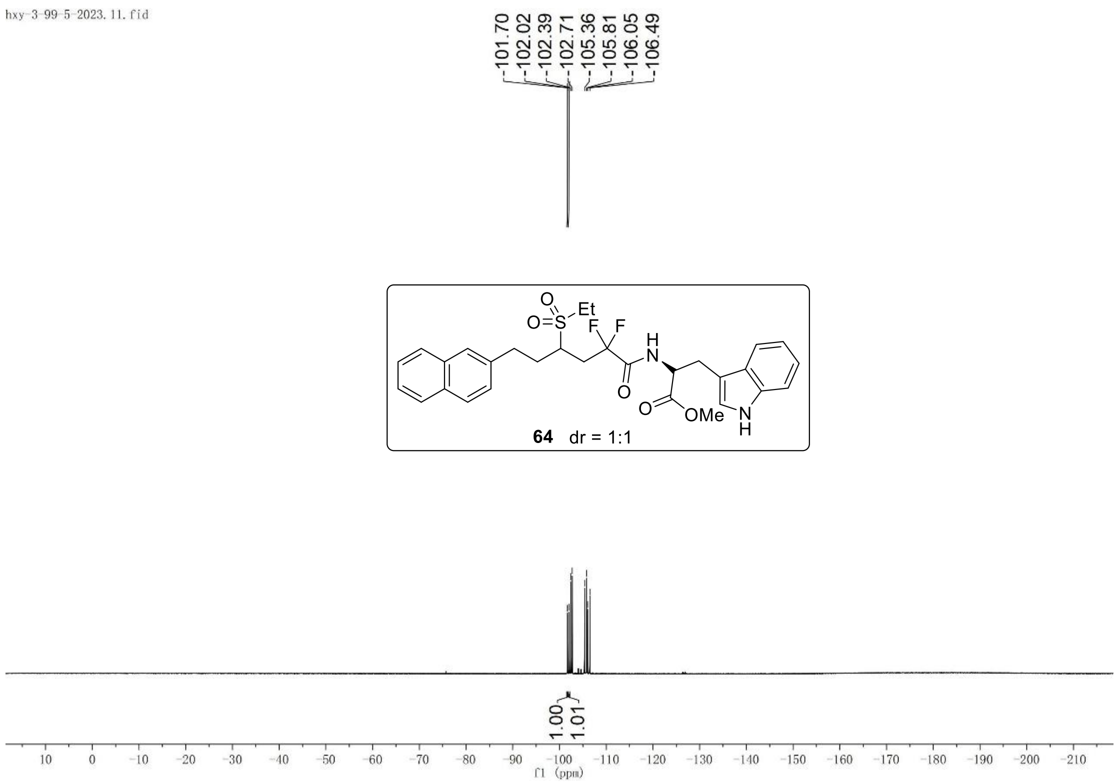

Supplementary Figure 236. ^{19}F NMR (376 MHz, $CDCl_3$) spectra of **64**

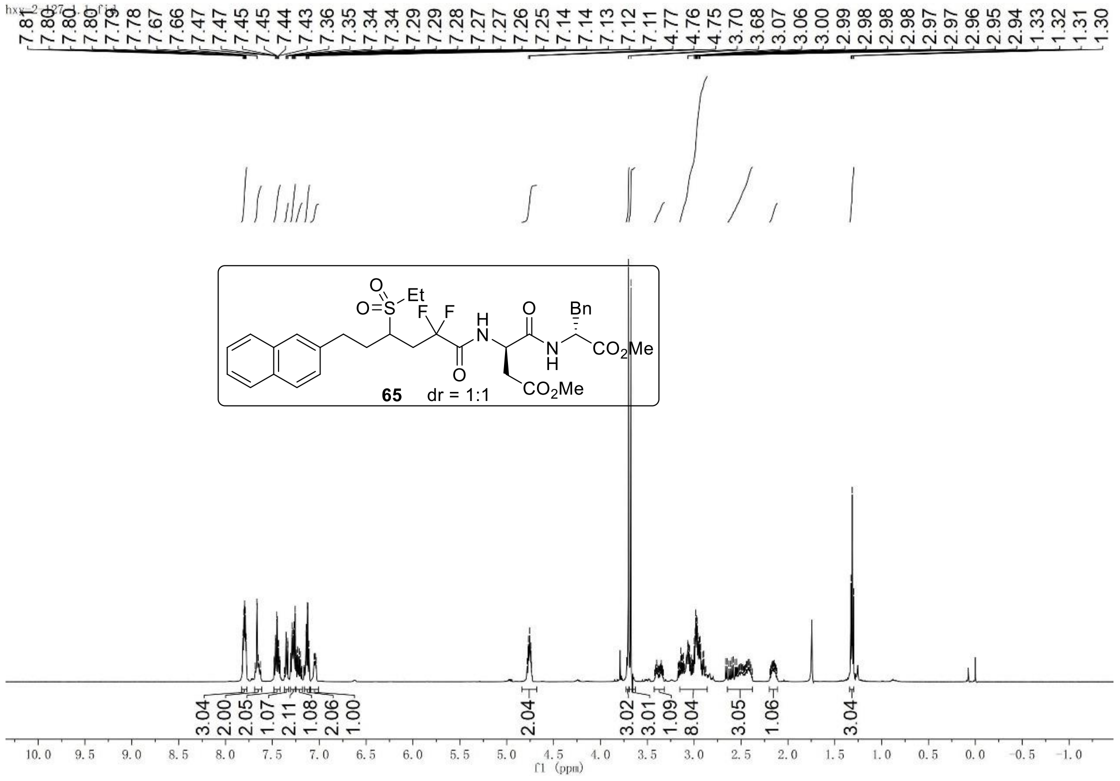

Supplementary Figure 237. ^{1}H NMR (500 MHz, $CDCl_3$) spectra of **65**

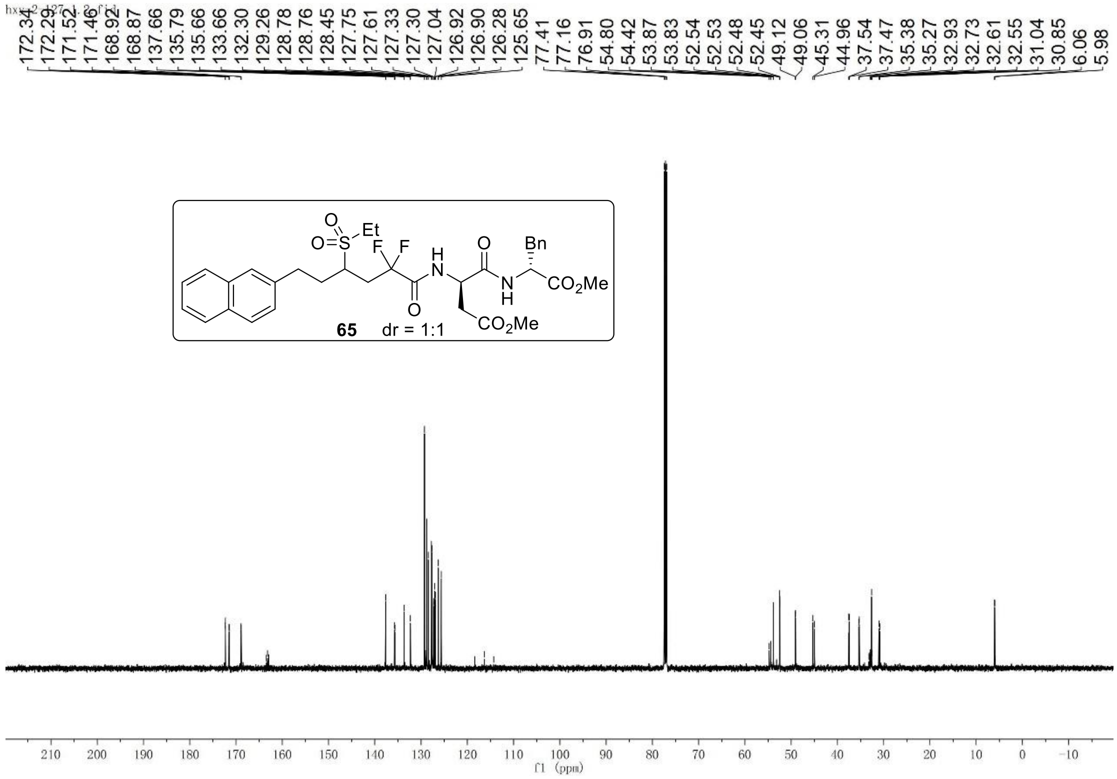

Supplementary Figure 238. ^{13}C NMR (126 MHz, $CDCl_3$) spectra of **65**

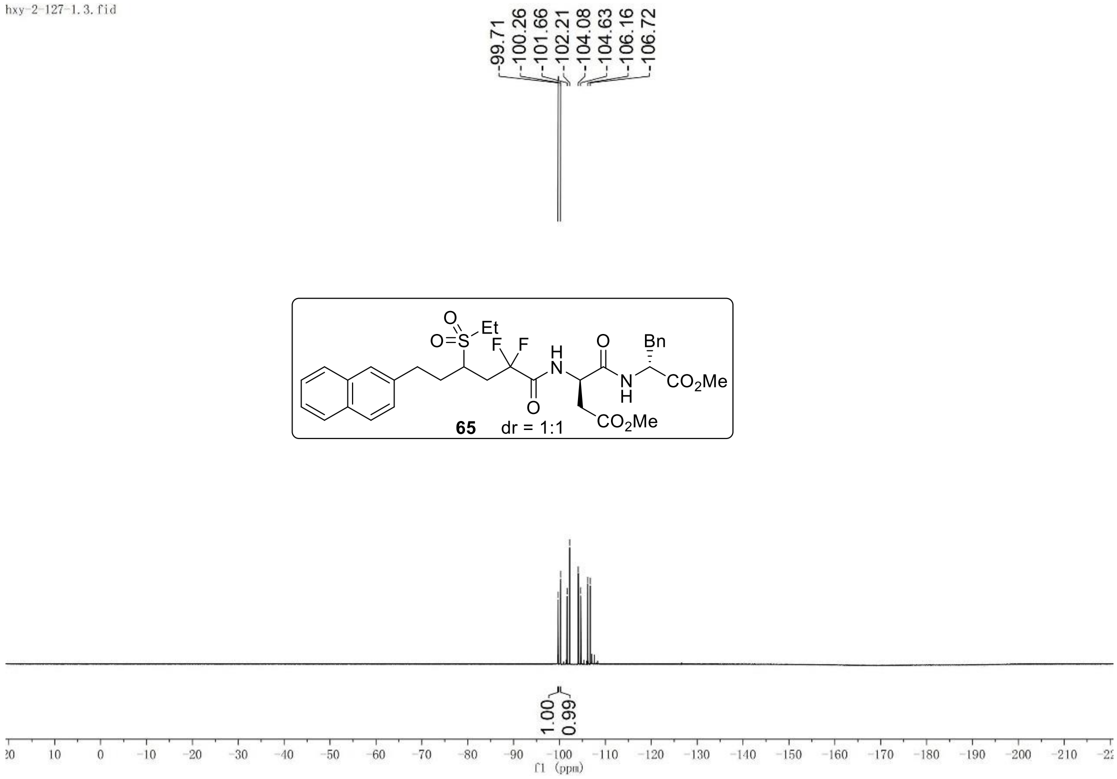

Supplementary Figure 239. ^{19}F NMR (471 MHz, $CDCl_3$) spectra of **65**

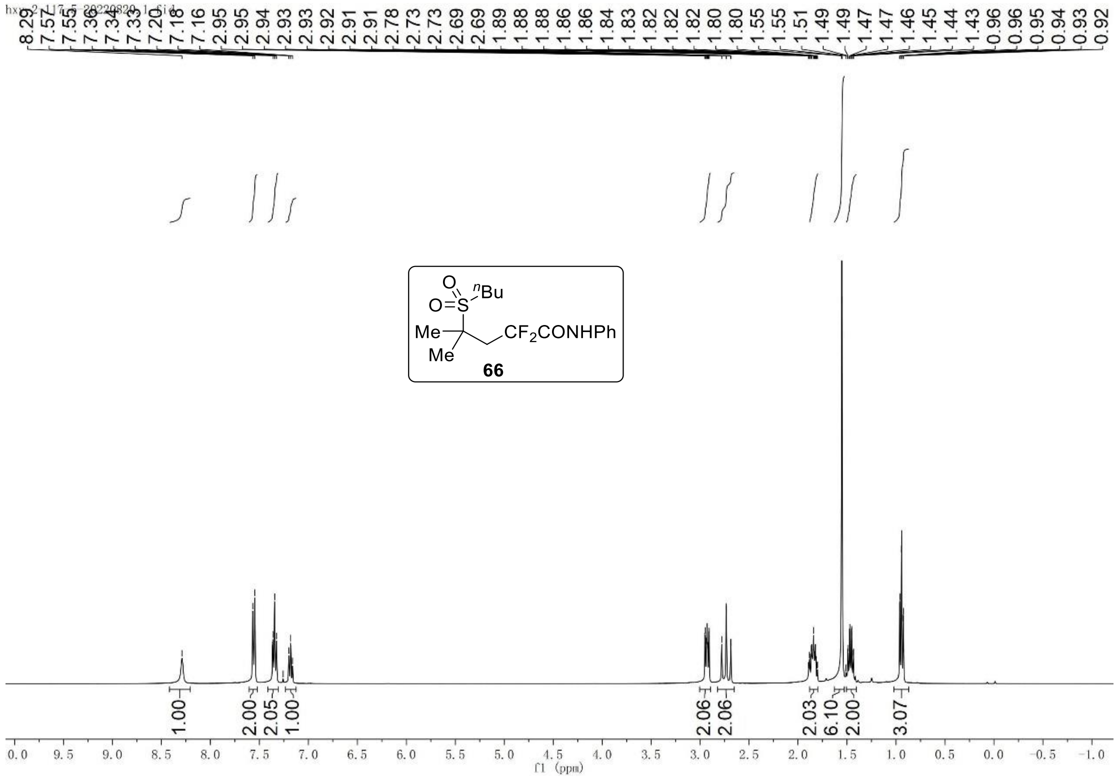

Supplementary Figure 240. ^{1}H NMR (400 MHz, $CDCl_3$) spectra of **66**

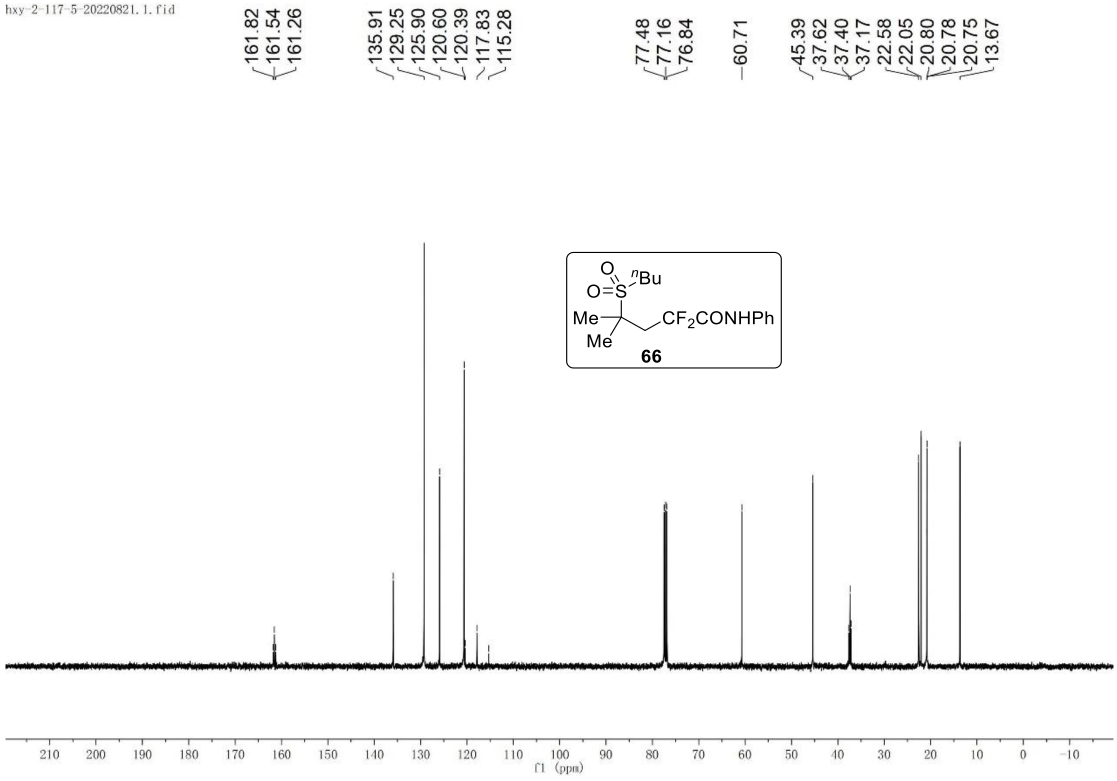

Supplementary Figure 241. ^{13}C NMR (101 MHz, $CDCl_3$) spectra of **66**

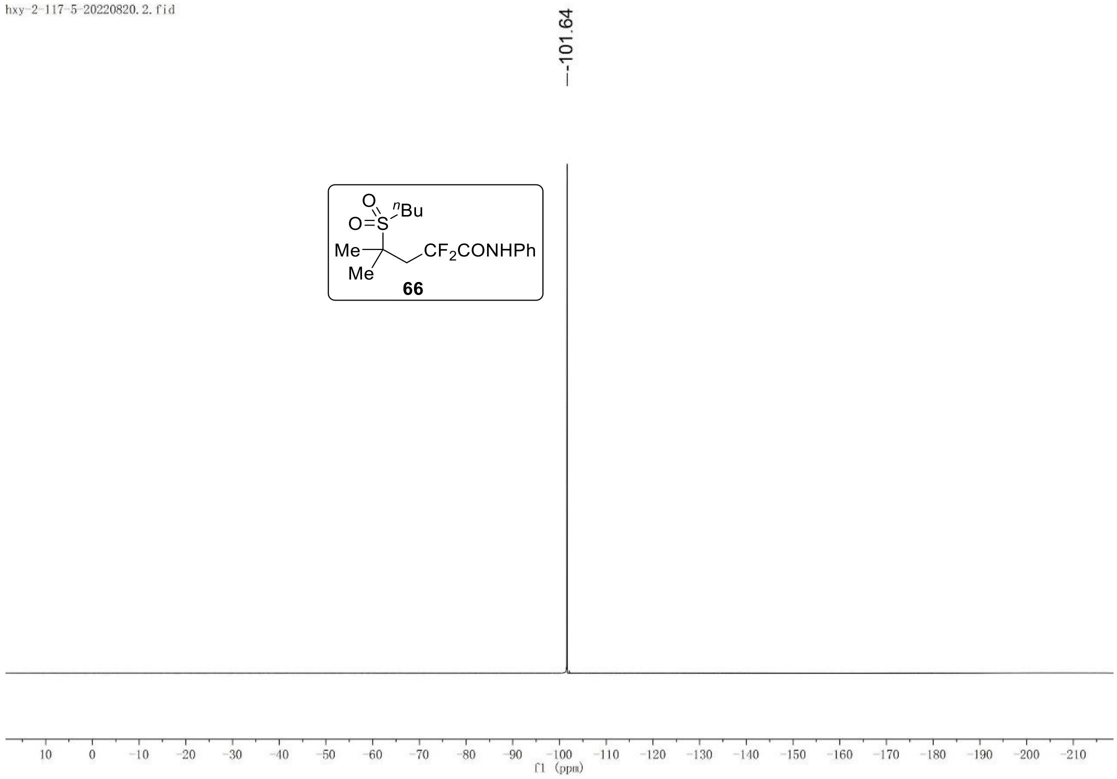

Supplementary Figure 242. ^{19}F NMR (376 MHz, $CDCl_3$) spectra of **66**

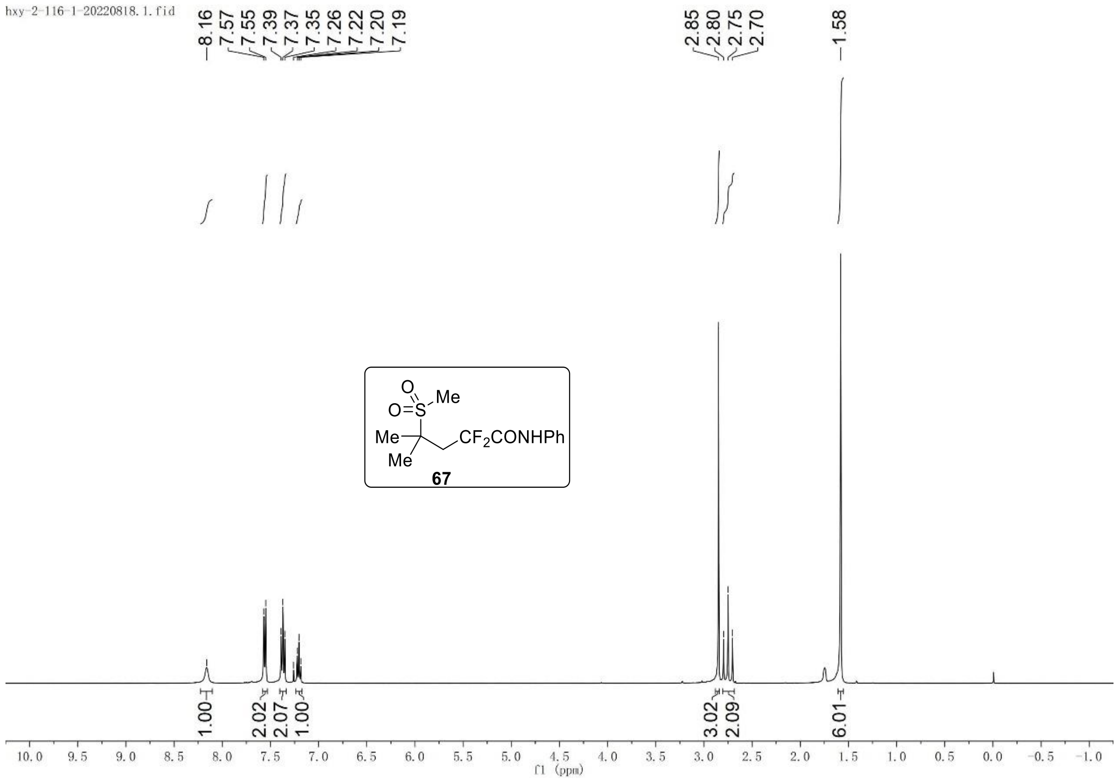

Supplementary Figure 243. ^{1}H NMR (400 MHz, $CDCl_3$) spectra of **67**

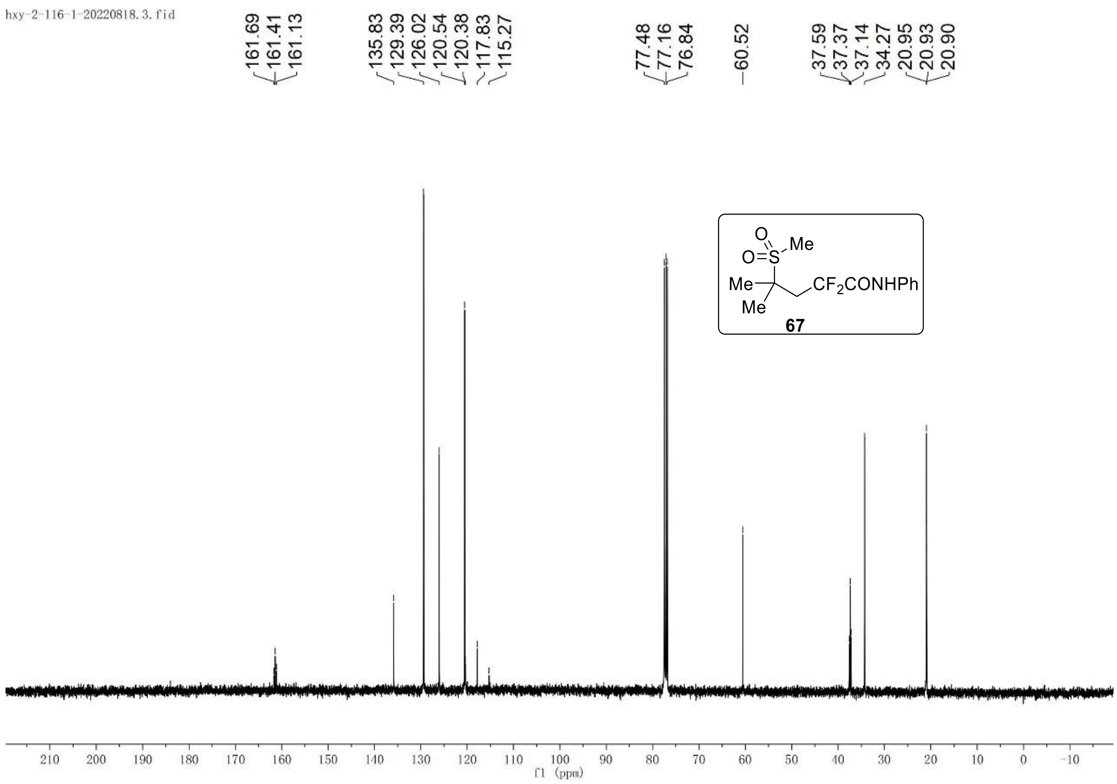

Supplementary Figure 244. ^{13}C NMR (101 MHz, $CDCl_3$) spectra of **67**

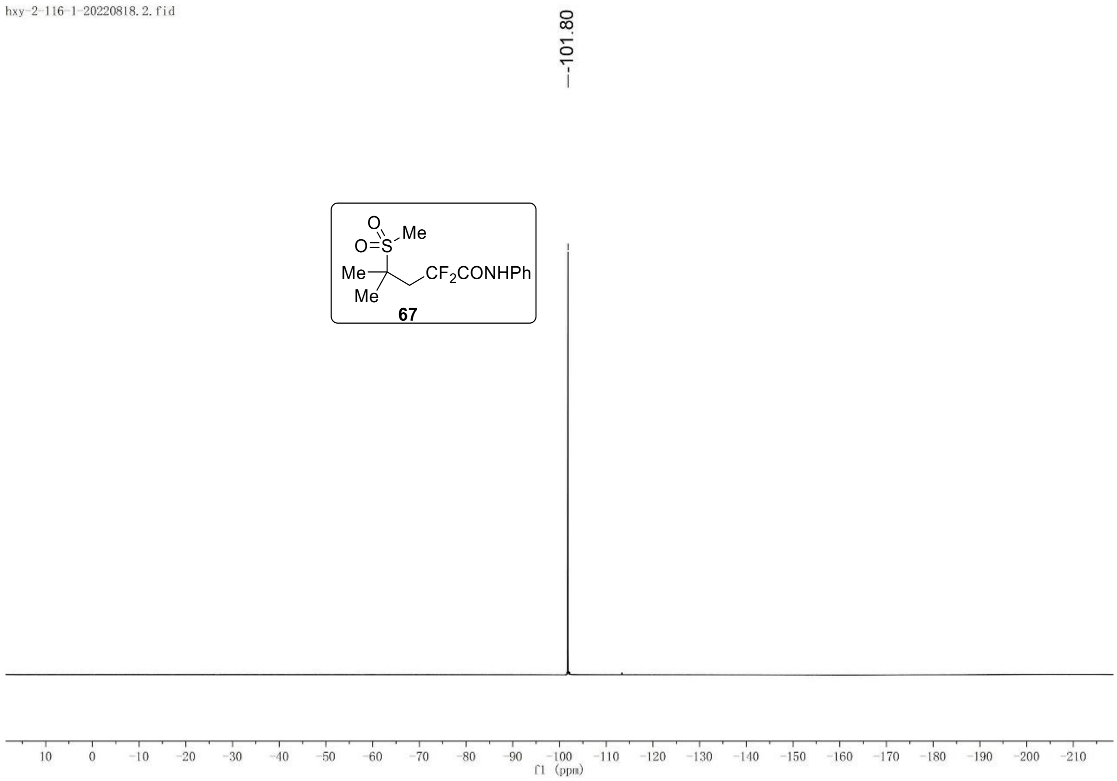

Supplementary Figure 245. ^{19}F NMR (376 MHz, $CDCl_3$) spectra of **67**

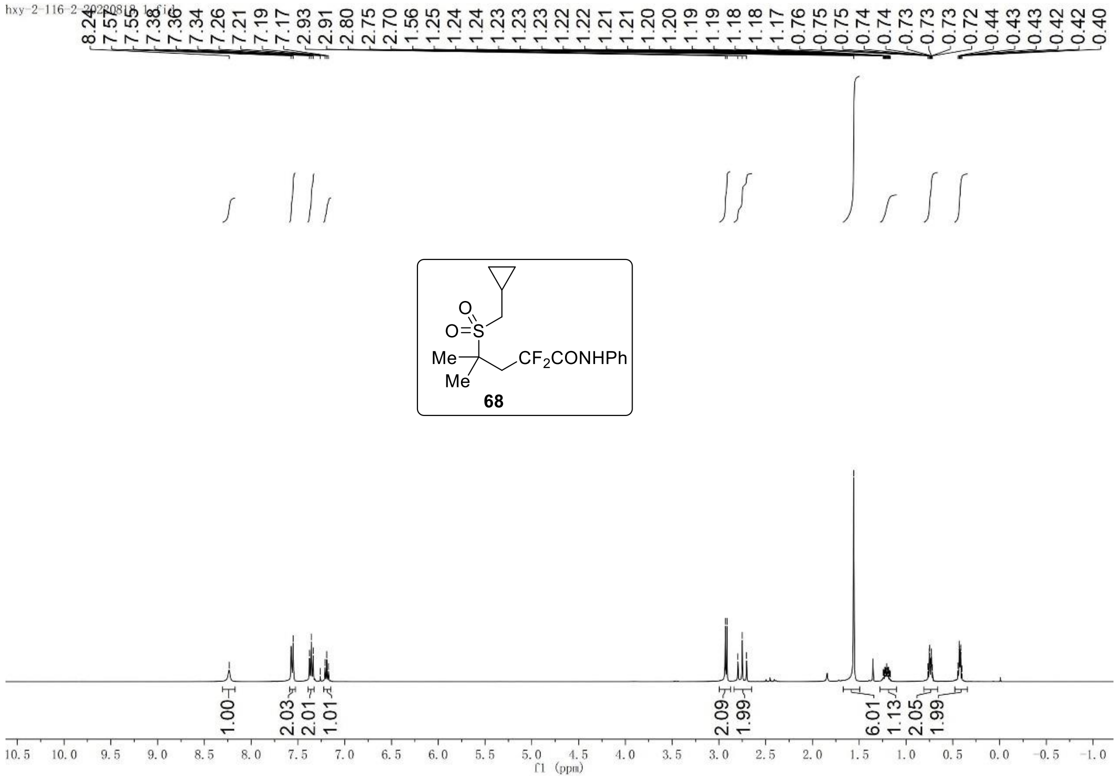

Supplementary Figure 246. ^{1}H NMR (400 MHz, $CDCl_3$) spectra of **68**

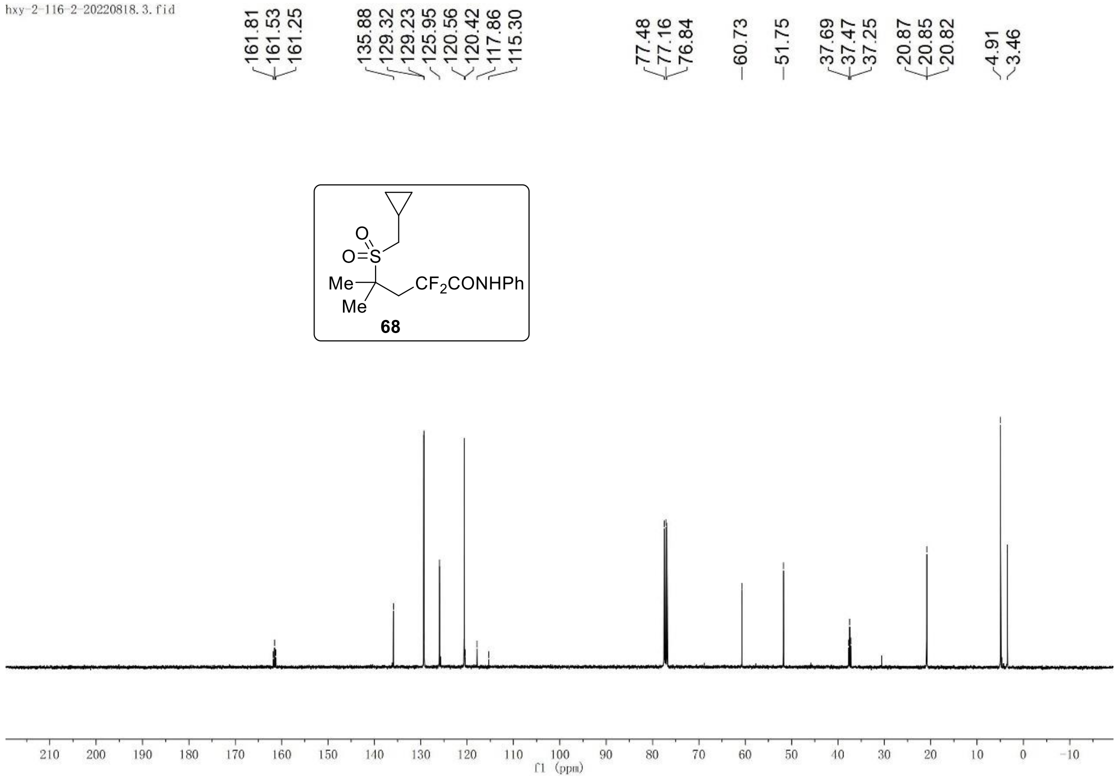

Supplementary Figure 247. ^{13}C NMR (101 MHz, $CDCl_3$) spectra of **68**

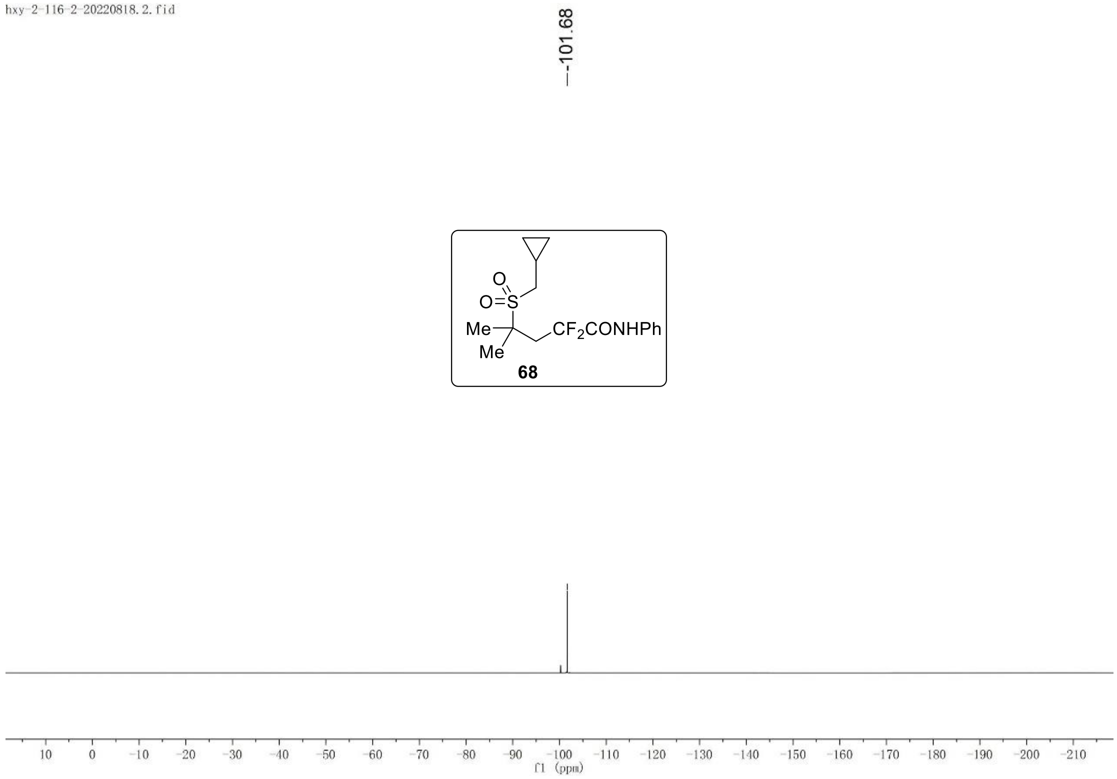

Supplementary Figure 248. ^{19}F NMR (376 MHz, $CDCl_3$) spectra of **68**

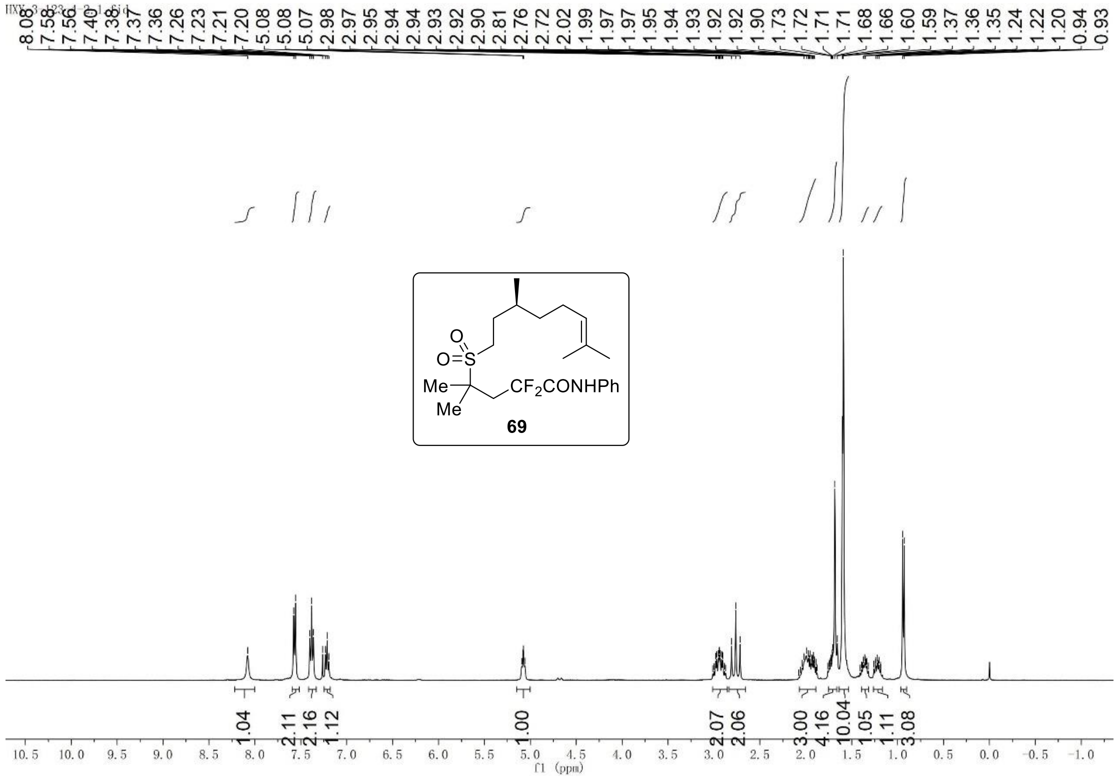

Supplementary Figure 249. ^{1}H NMR (400 MHz, $CDCl_3$) spectra of **69**

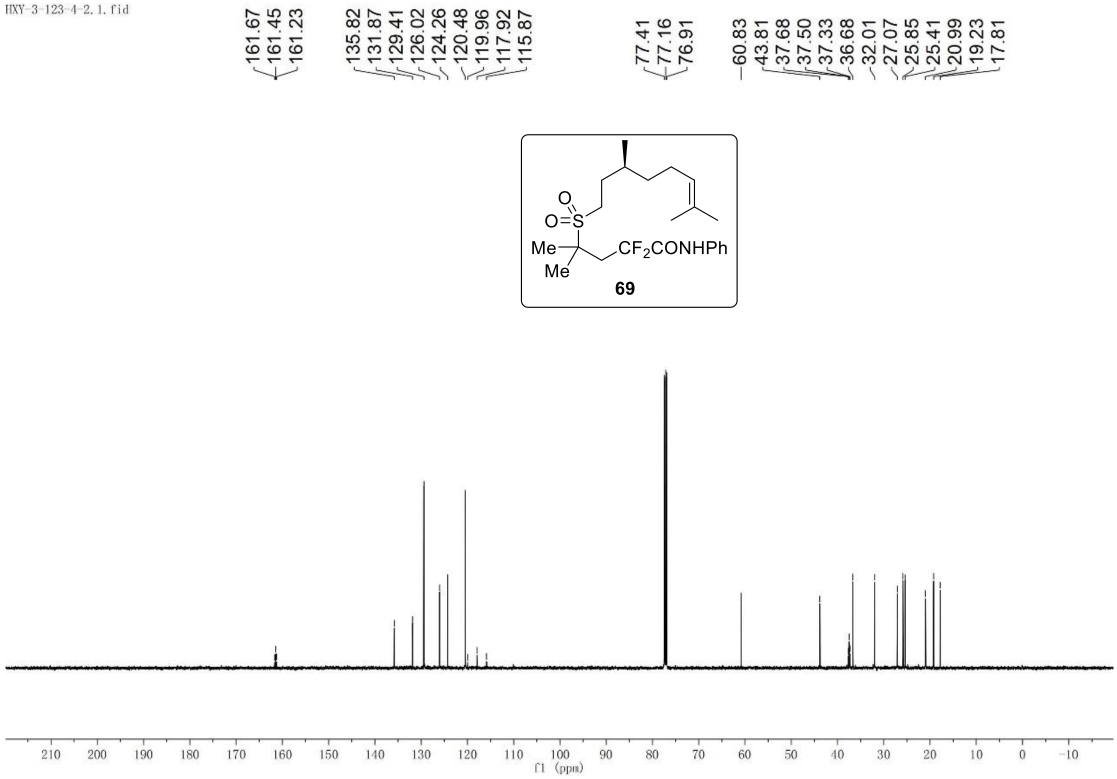

Supplementary Figure 250. ^{13}C NMR (101 MHz, $CDCl_3$) spectra of **69**

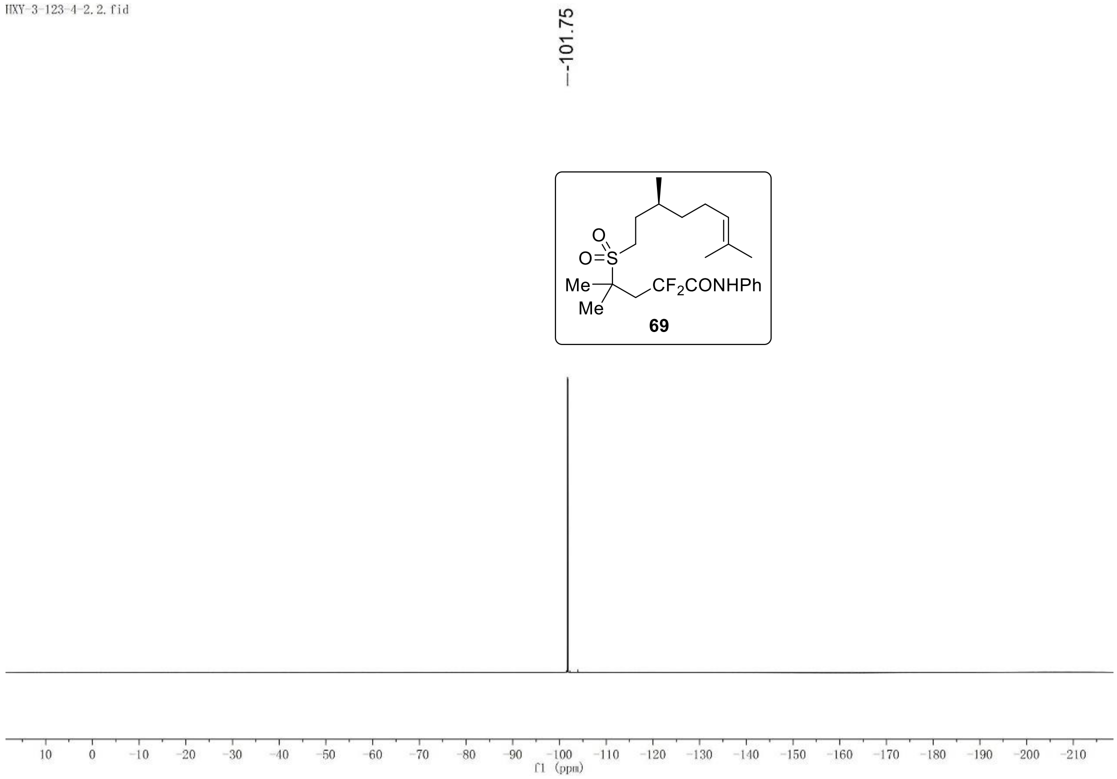

Supplementary Figure 251. ^{19}F NMR (376 MHz, $CDCl_3$) spectra of **69**

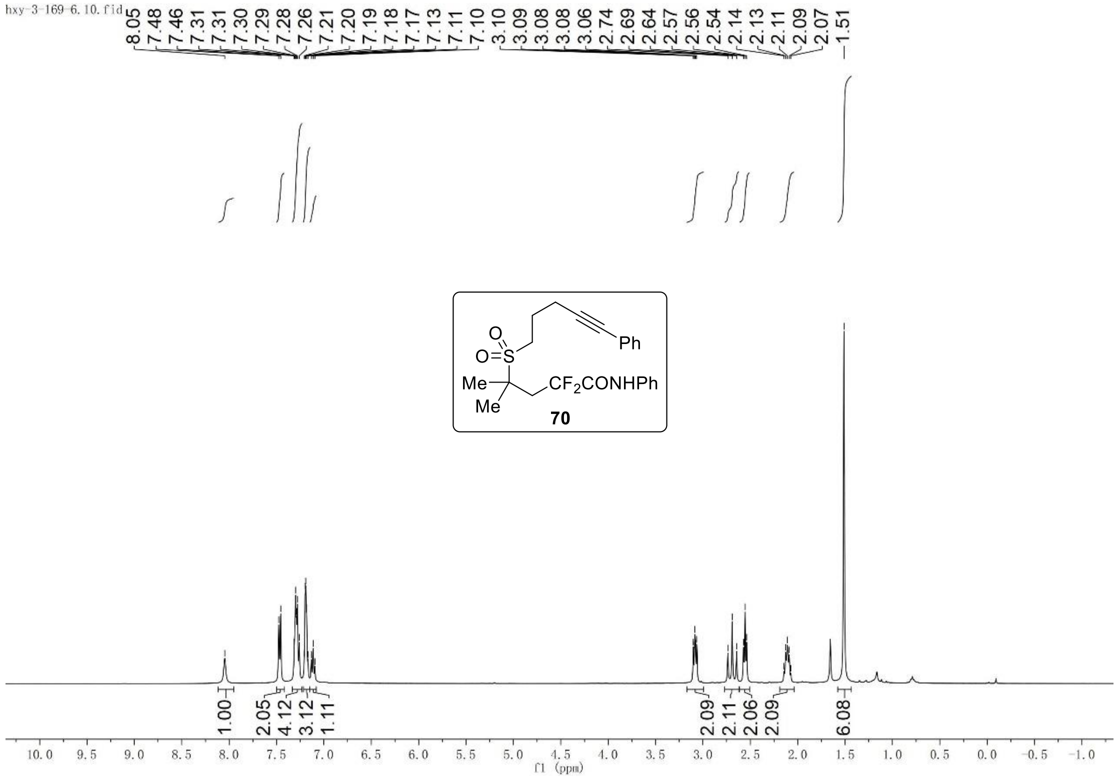

Supplementary Figure 252. ^{1}H NMR (400 MHz, CDCl3) spectra of **70**

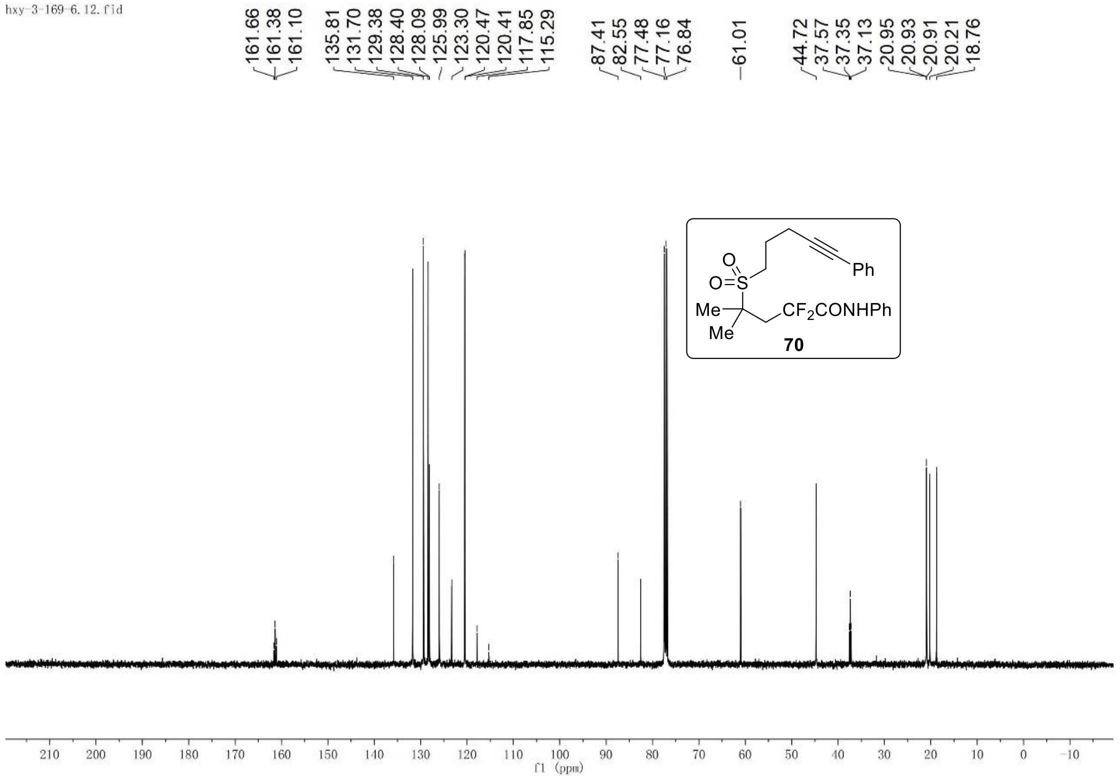

Supplementary Figure 253. ^{13}C NMR (101 MHz, $CDCl_3$) spectra of **70**

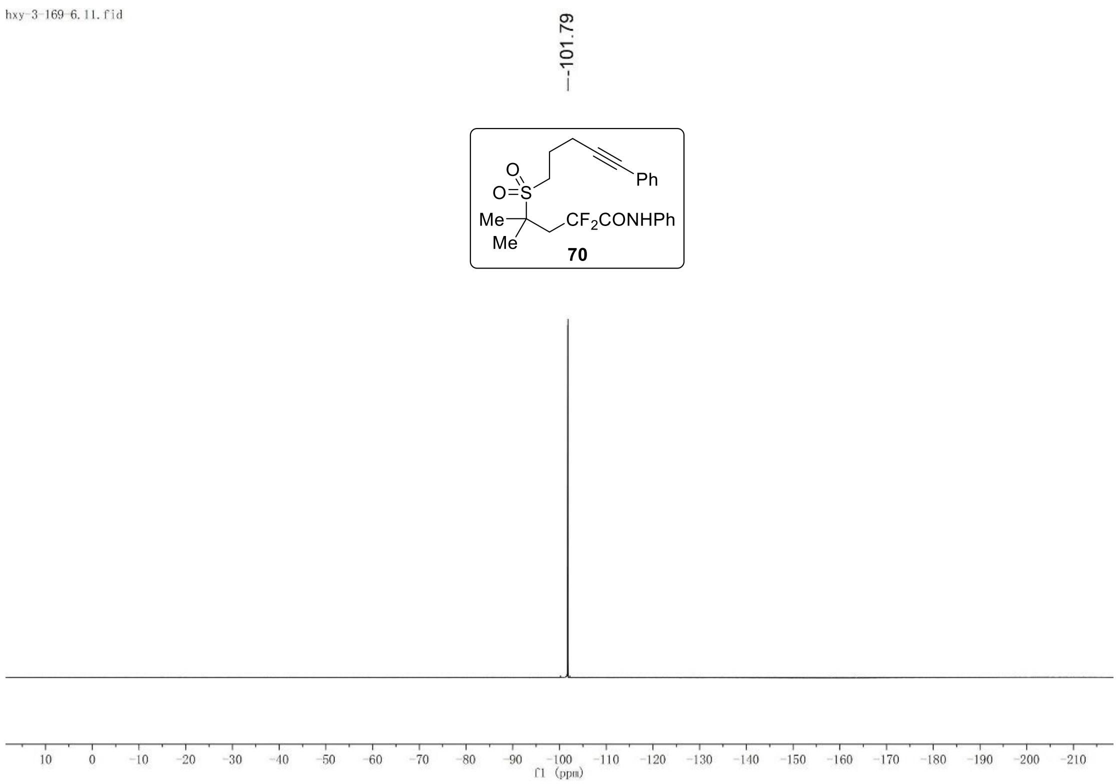

Supplementary Figure 254. ^{19}F NMR (376 MHz, $CDCl_3$) spectra of **70**

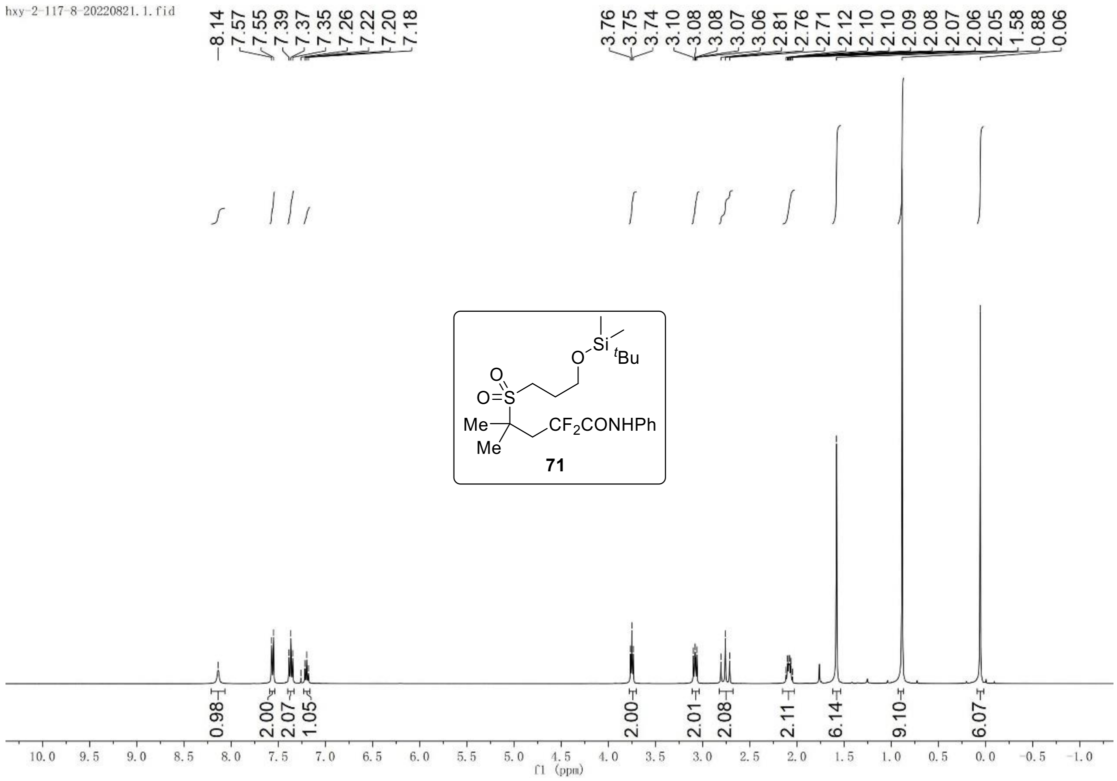

Supplementary Figure 255. ^{1}H NMR (400 MHz, $CDCl_3$) spectra of **71**

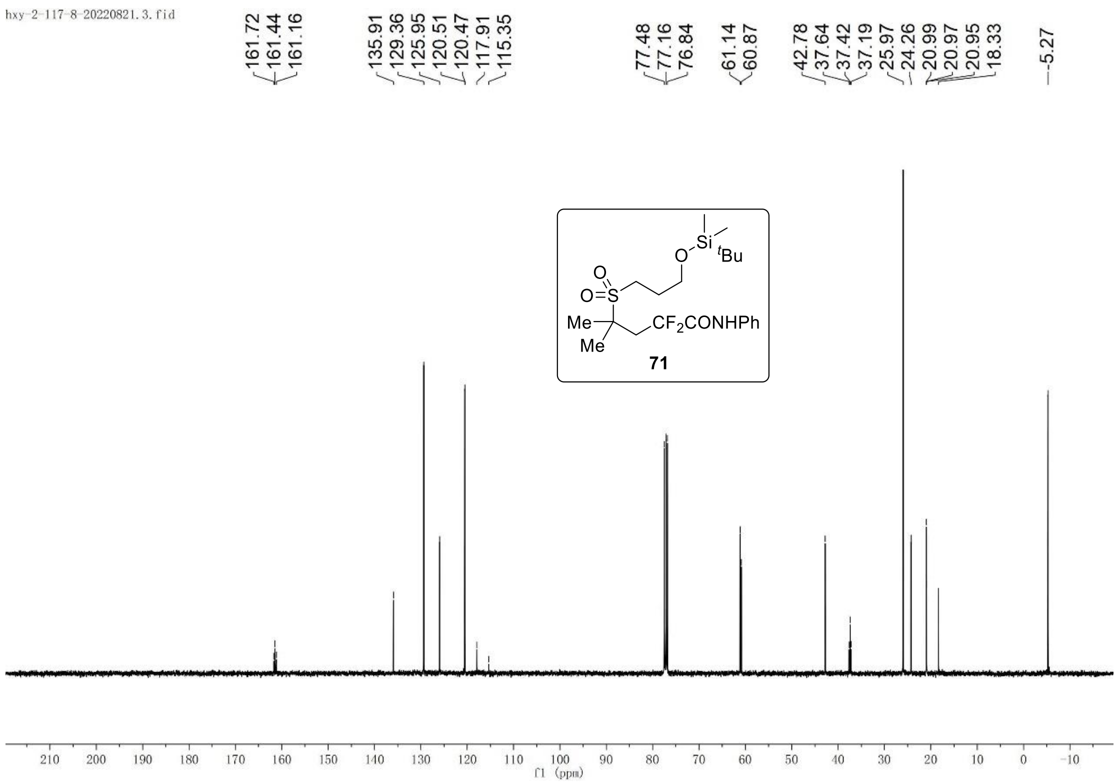

Supplementary Figure 256. ^{13}C NMR (101 MHz, $CDCl_3$) spectra of **71**

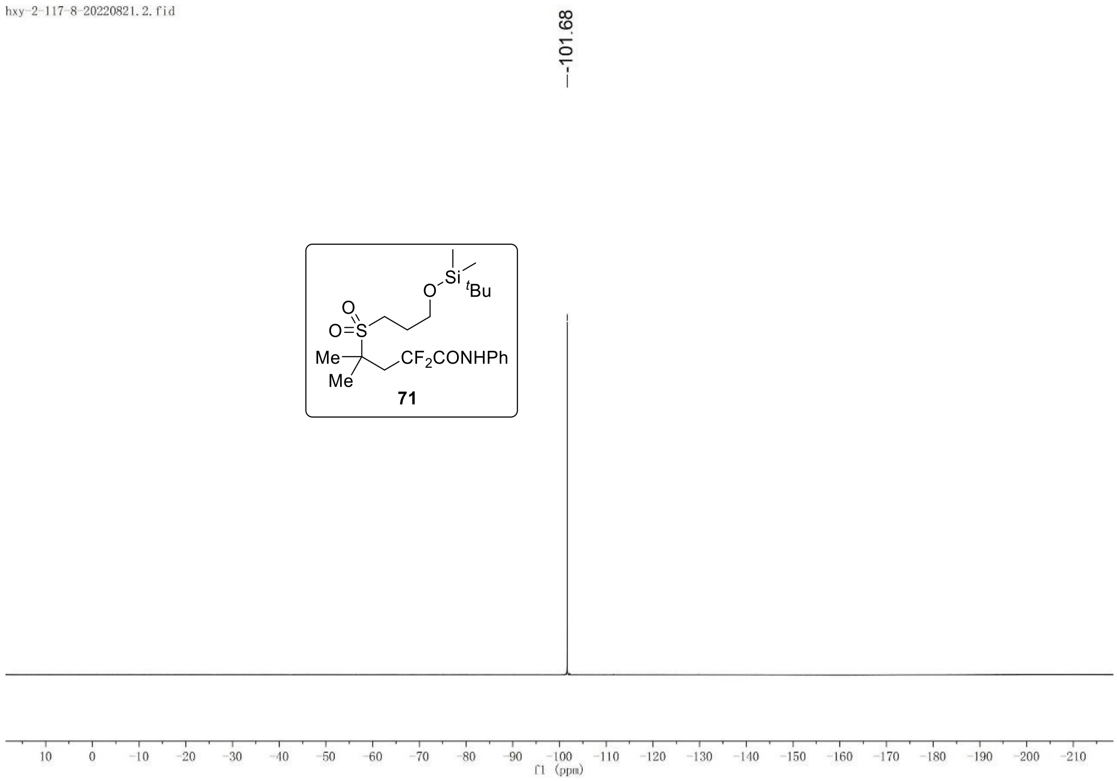

Supplementary Figure 257. ^{19}F NMR (376 MHz, $CDCl_3$) spectra of **71**

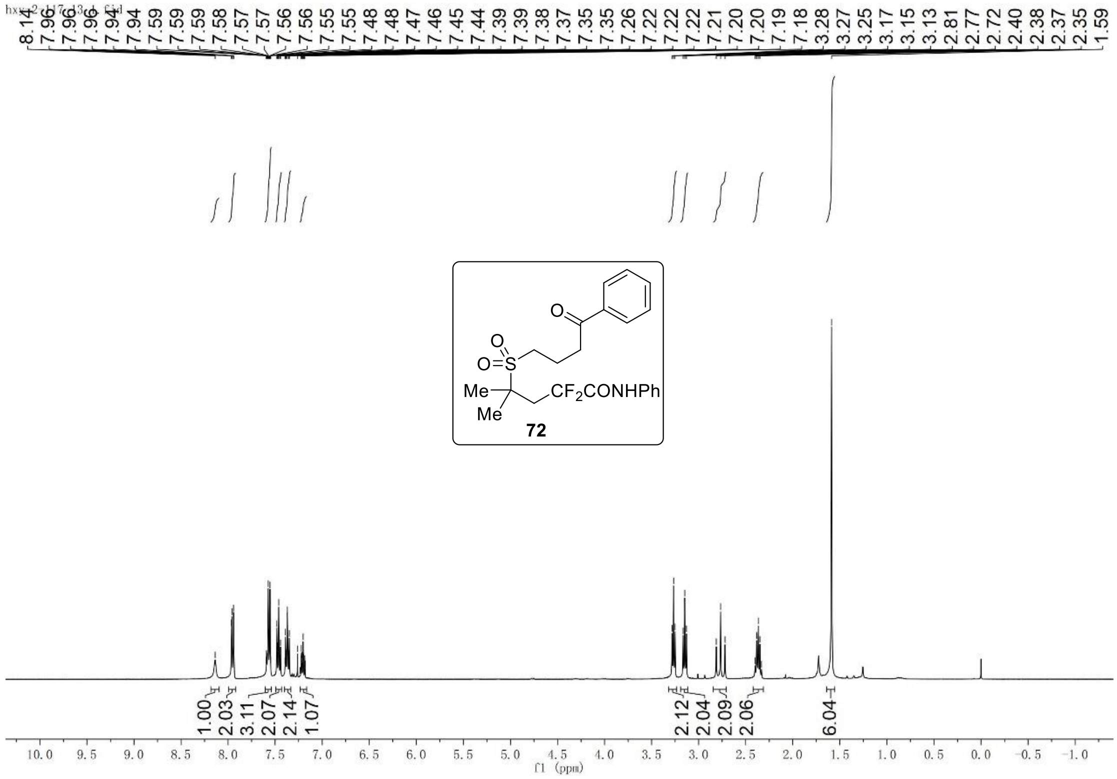

Supplementary Figure 258. ^{1}H NMR (400 MHz, $CDCl_3$) spectra of **72**

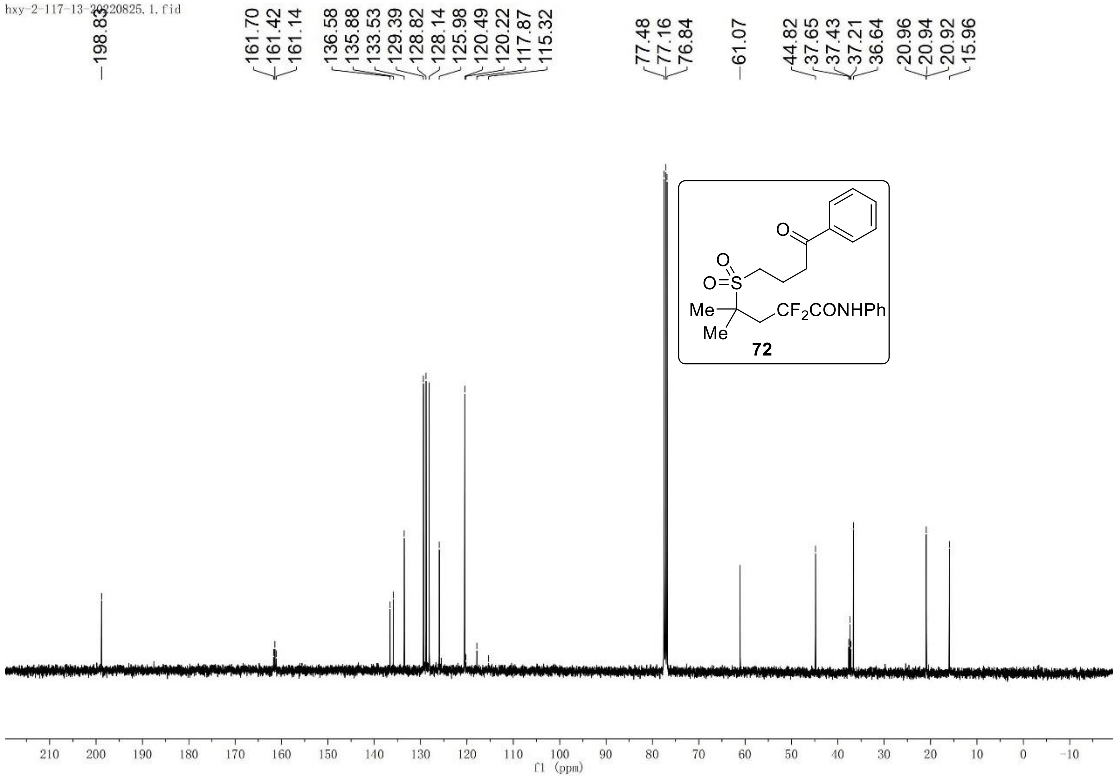

Supplementary Figure 259. ^{13}C NMR (101 MHz, $CDCl_3$) spectra of **72**

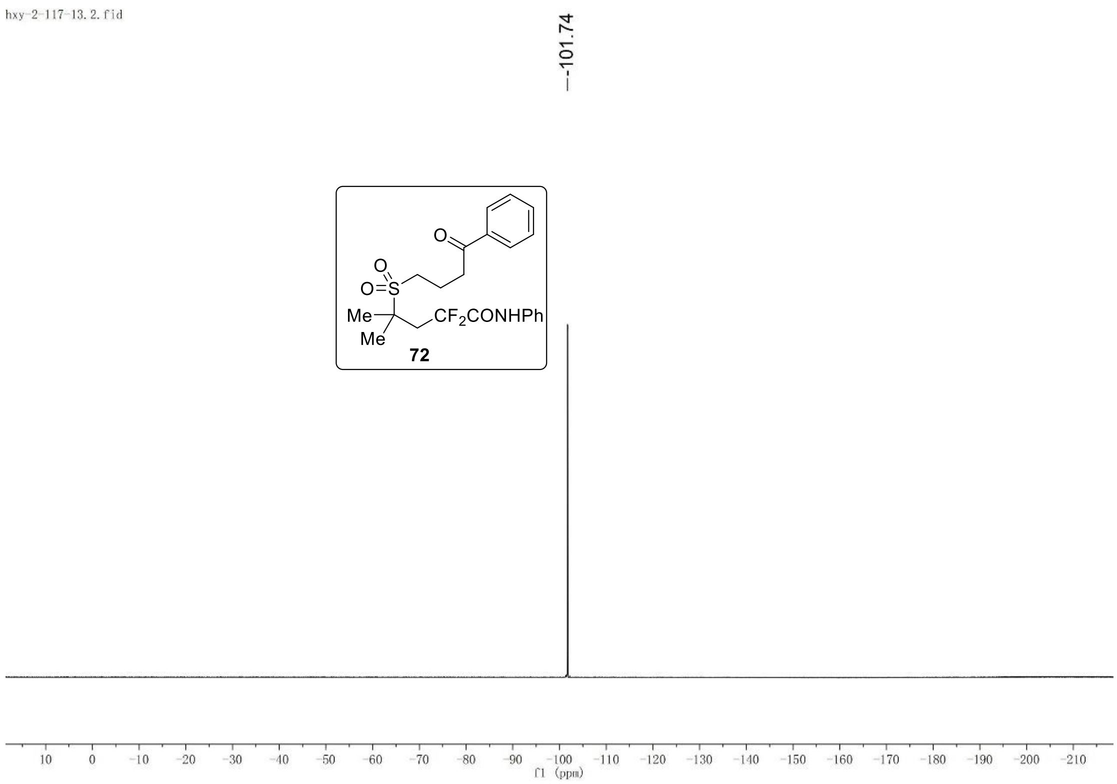

Supplementary Figure 260. ^{19}F NMR (376 MHz, $CDCl_3$) spectra of **72**

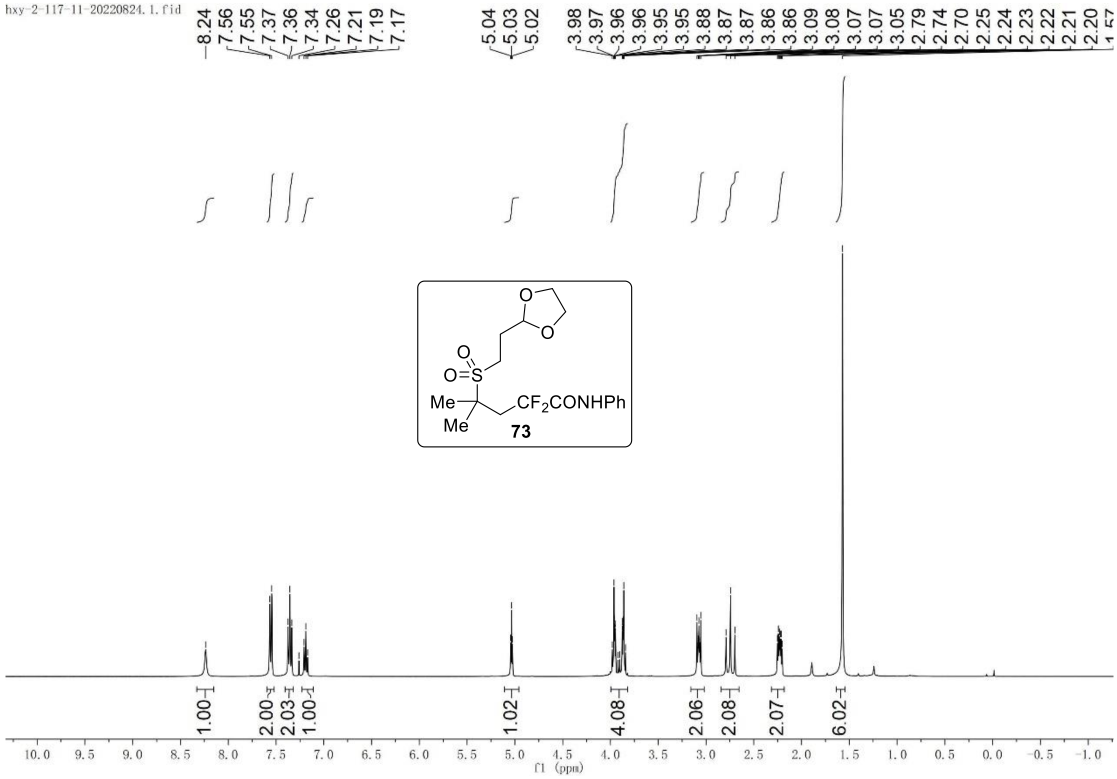

Supplementary Figure 261. ^{1}H NMR (400 MHz, $CDCl_3$) spectra of **73**

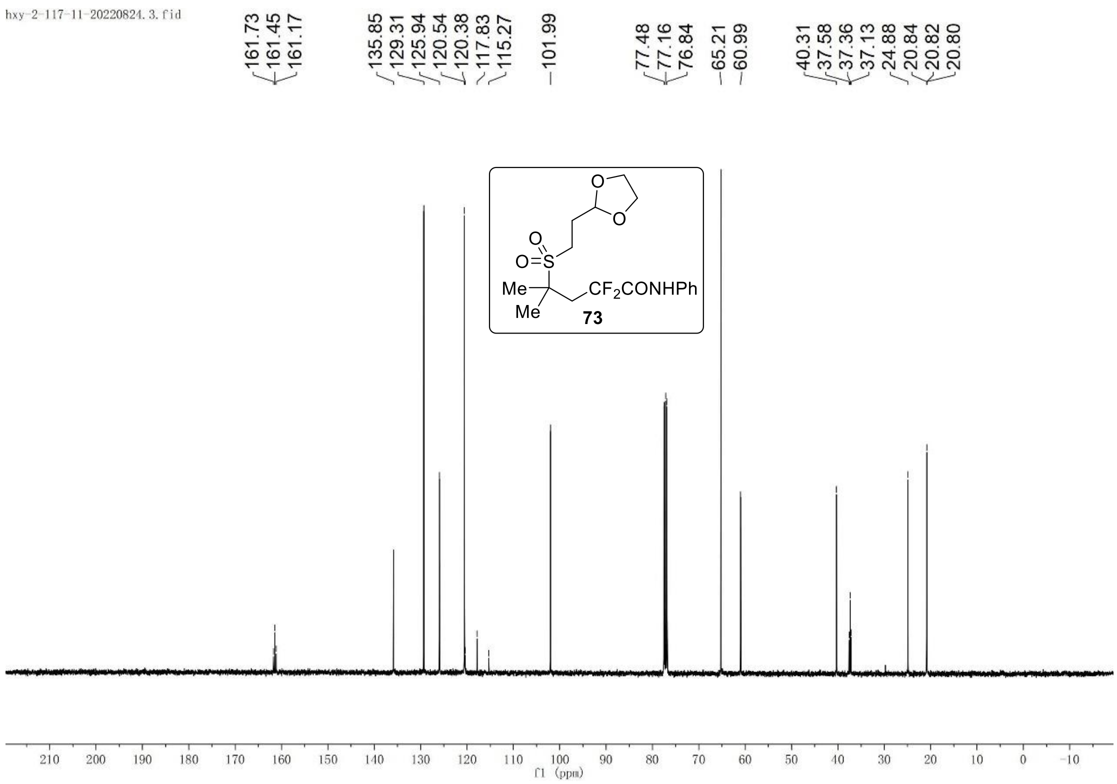

Supplementary Figure 262. ^{13}C NMR (101 MHz, $CDCl_3$) spectra of **73**

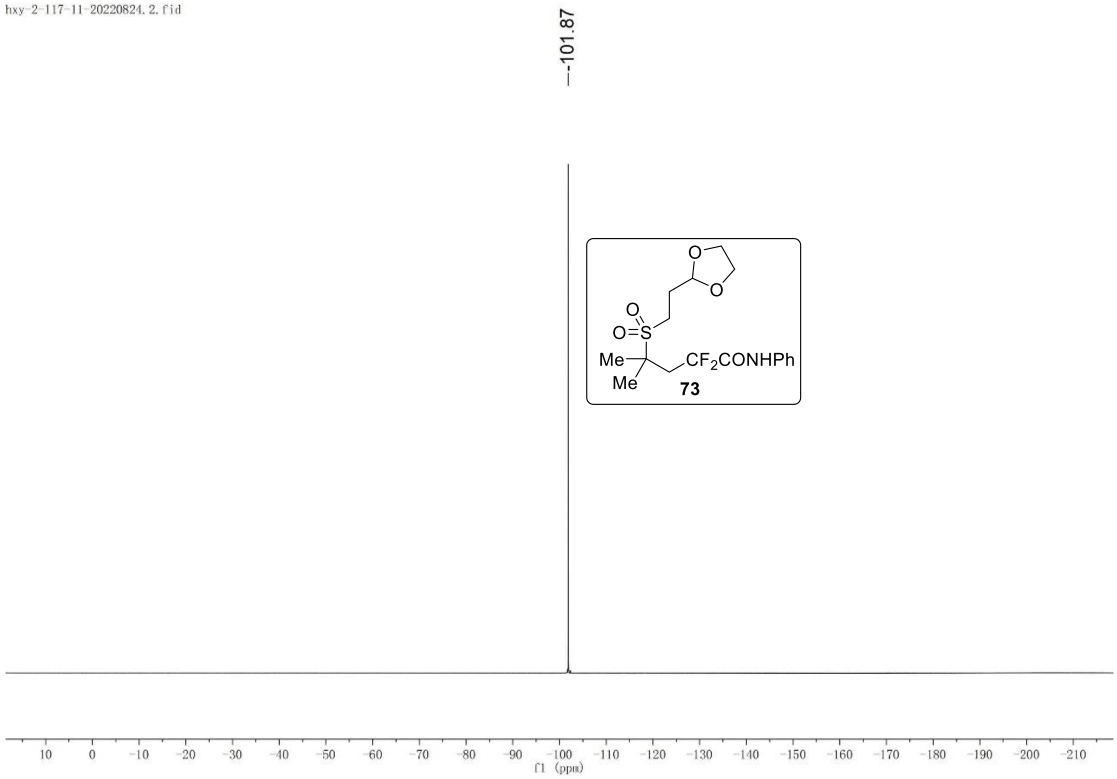

Supplementary Figure 263. ^{19}F NMR (376 MHz, $CDCl_3$) spectra of **73**

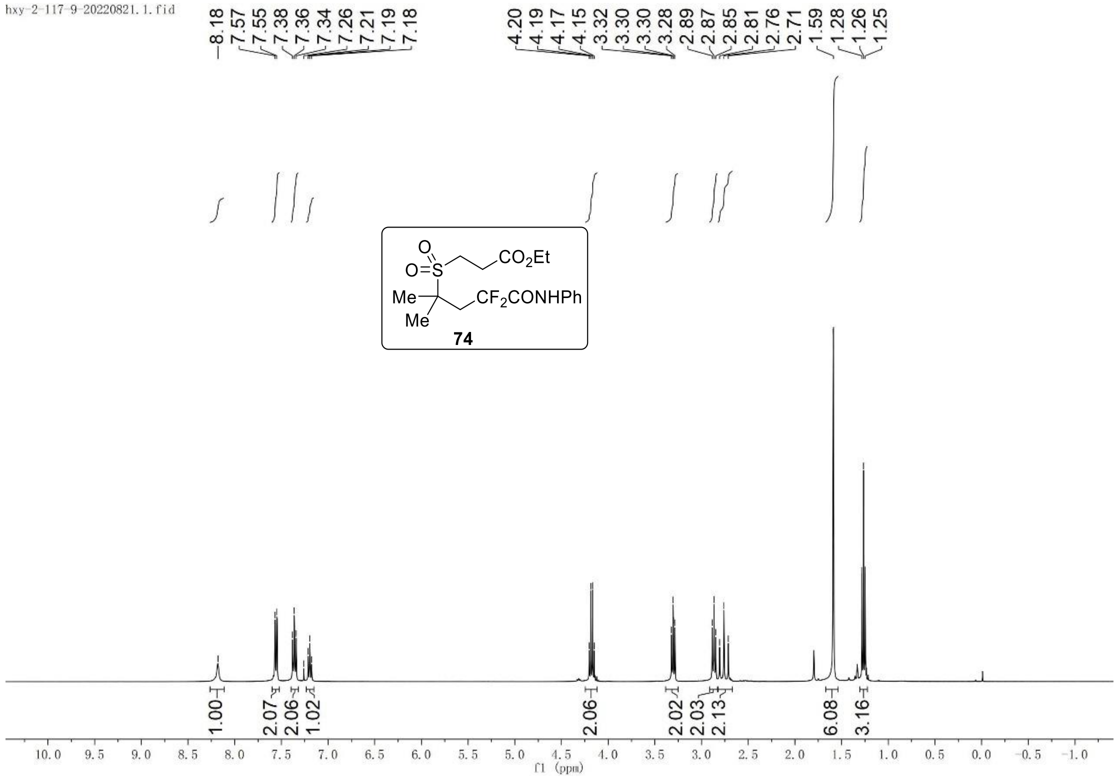

Supplementary Figure 264. ^{1}H NMR (400 MHz, $CDCl_3$) spectra of **74**

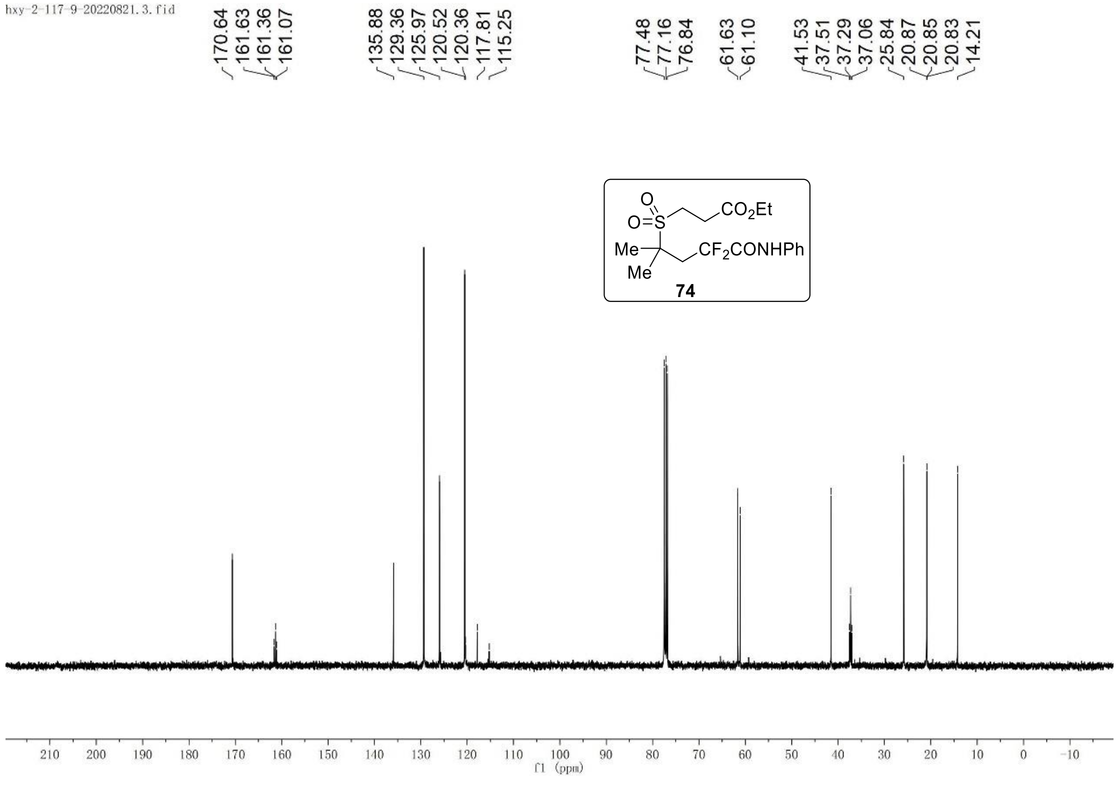

Supplementary Figure 265. ^{13}C NMR (101 MHz, $CDCl_3$) spectra of **74**

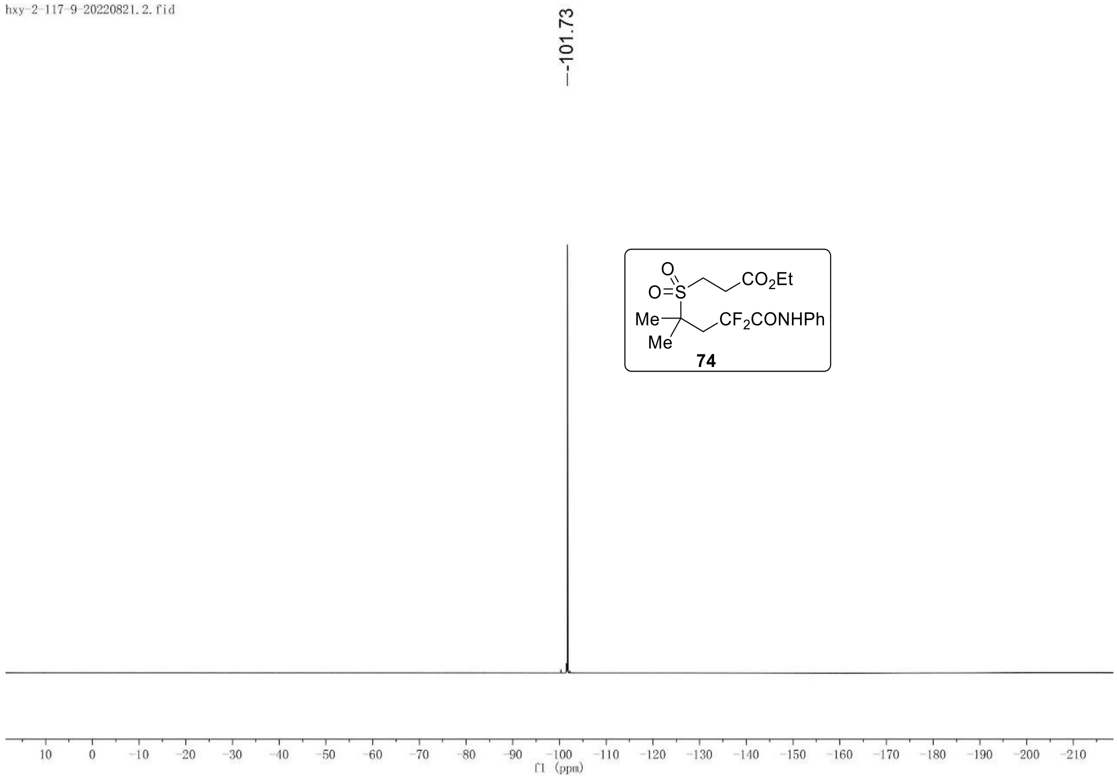

Supplementary Figure 266. ^{19}F NMR (376 MHz, $CDCl_3$) spectra of **74**

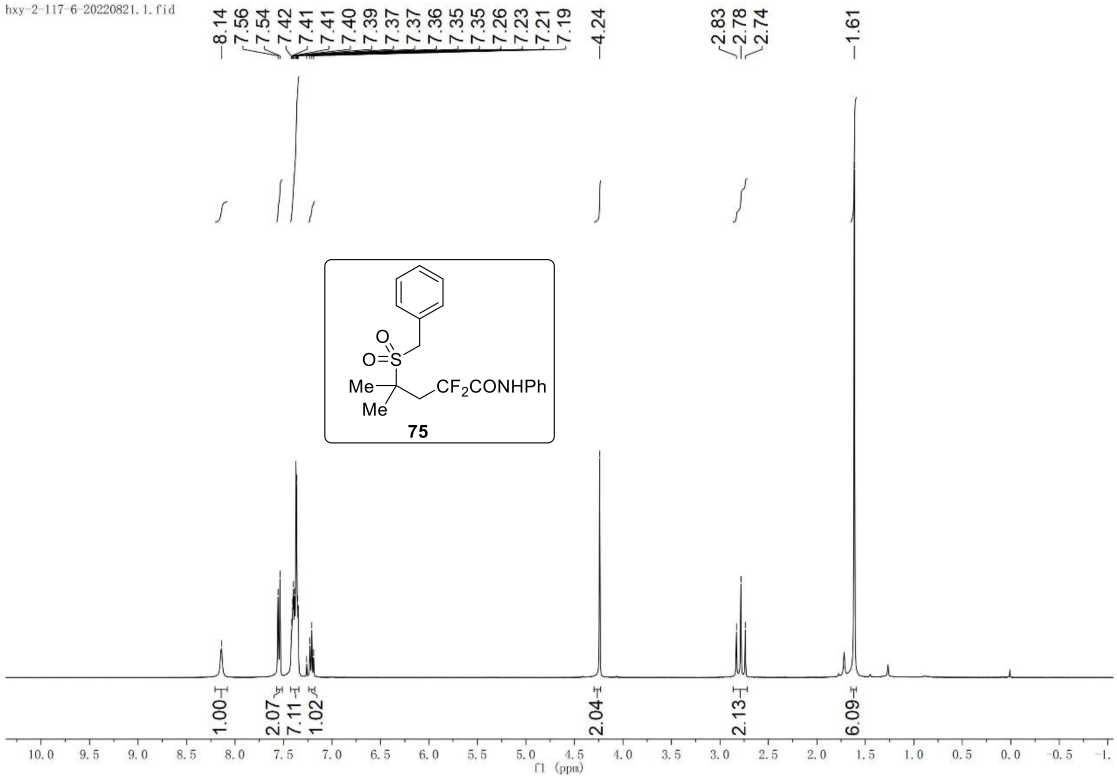

Supplementary Figure 267. ^{1}H NMR (400 MHz, $CDCl_3$) spectra of **75**

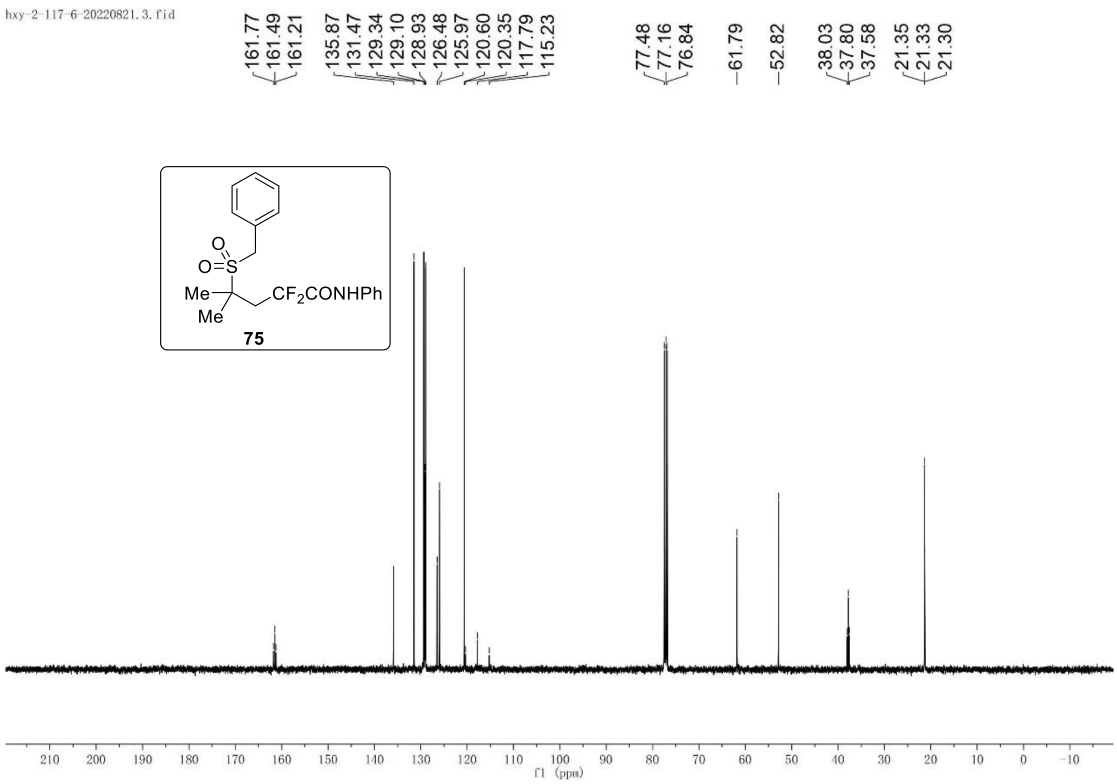

Supplementary Figure 268. ^{13}C NMR (101 MHz, $CDCl_3$) spectra of **75**

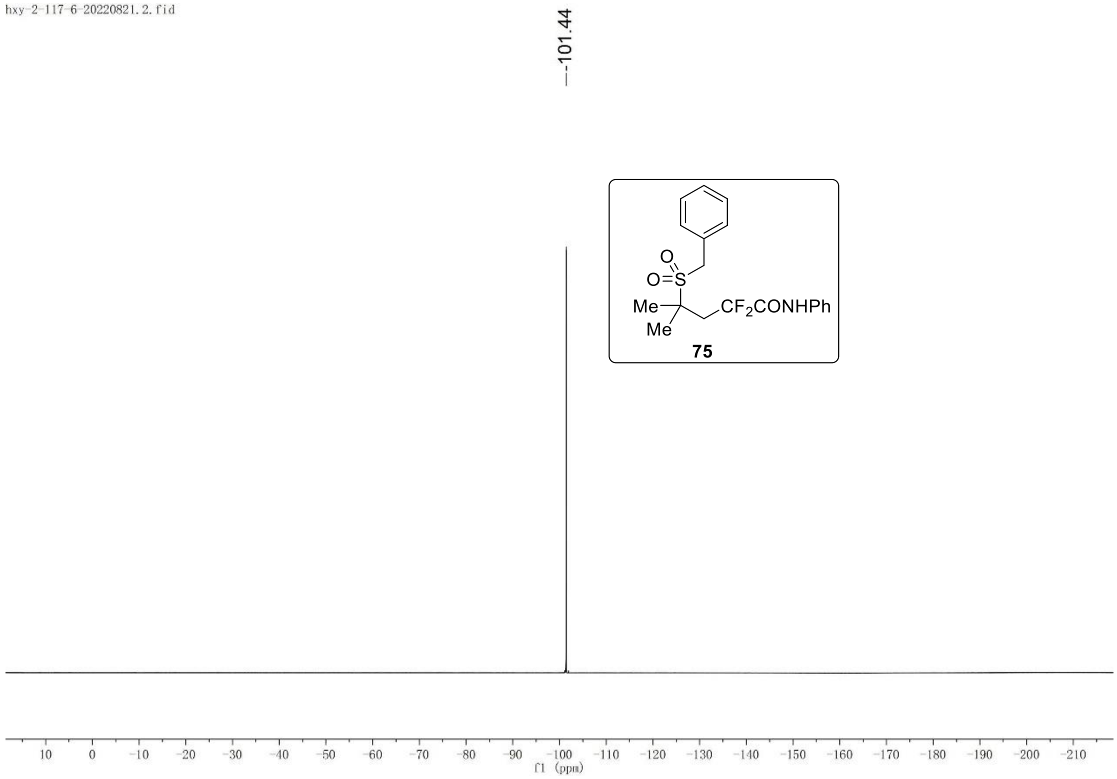

Supplementary Figure 269. ^{19}F NMR (376 MHz, $CDCl_3$) spectra of **75**

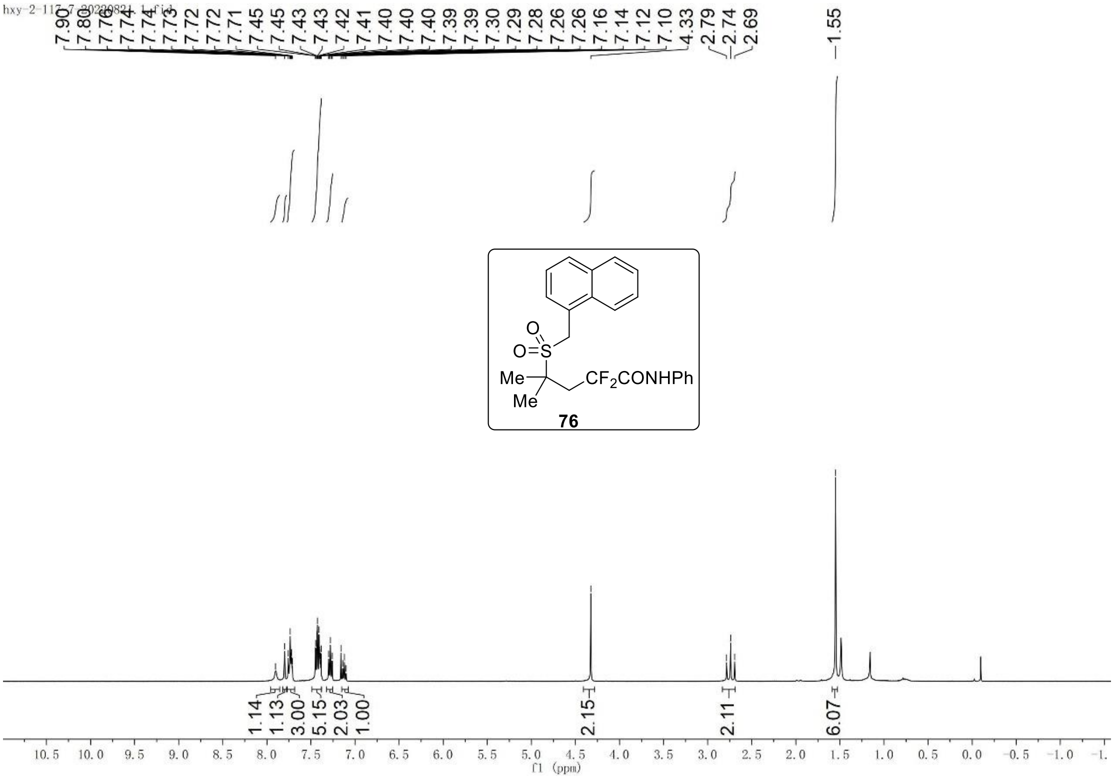

Supplementary Figure 270. ^{1}H NMR (400 MHz, $CDCl_3$) spectra of **76**

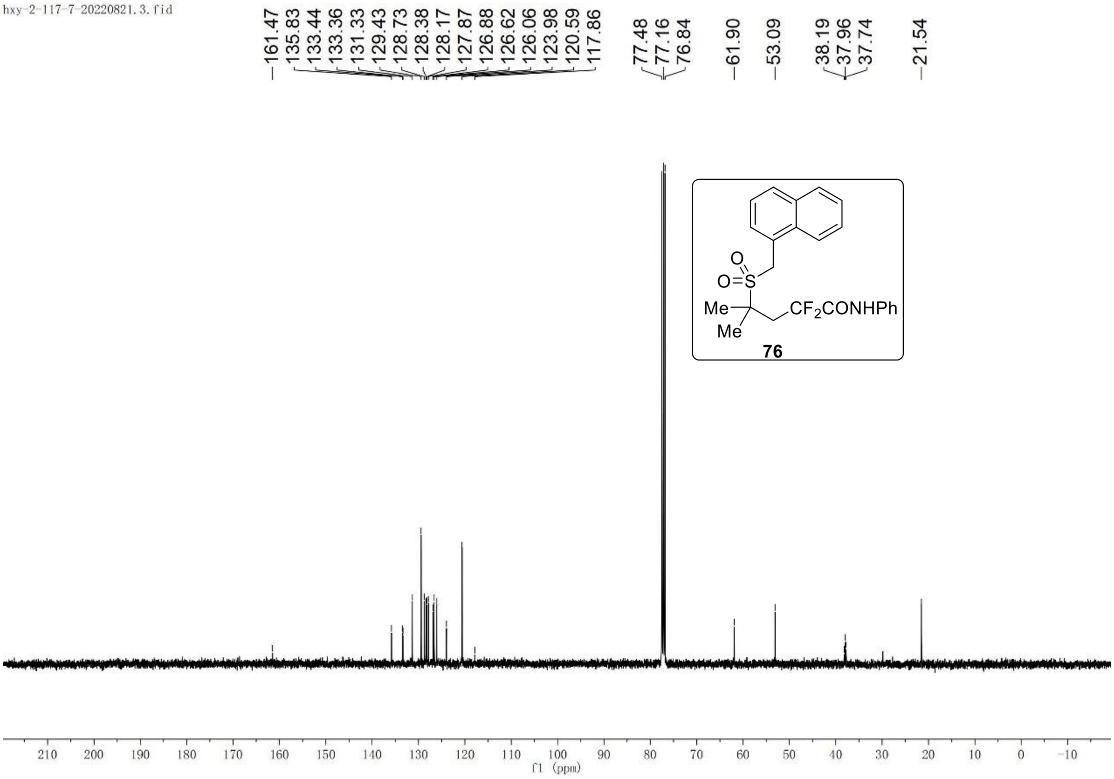

Supplementary Figure 271. ^{13}C NMR (101 MHz, $CDCl_3$) spectra of **76**

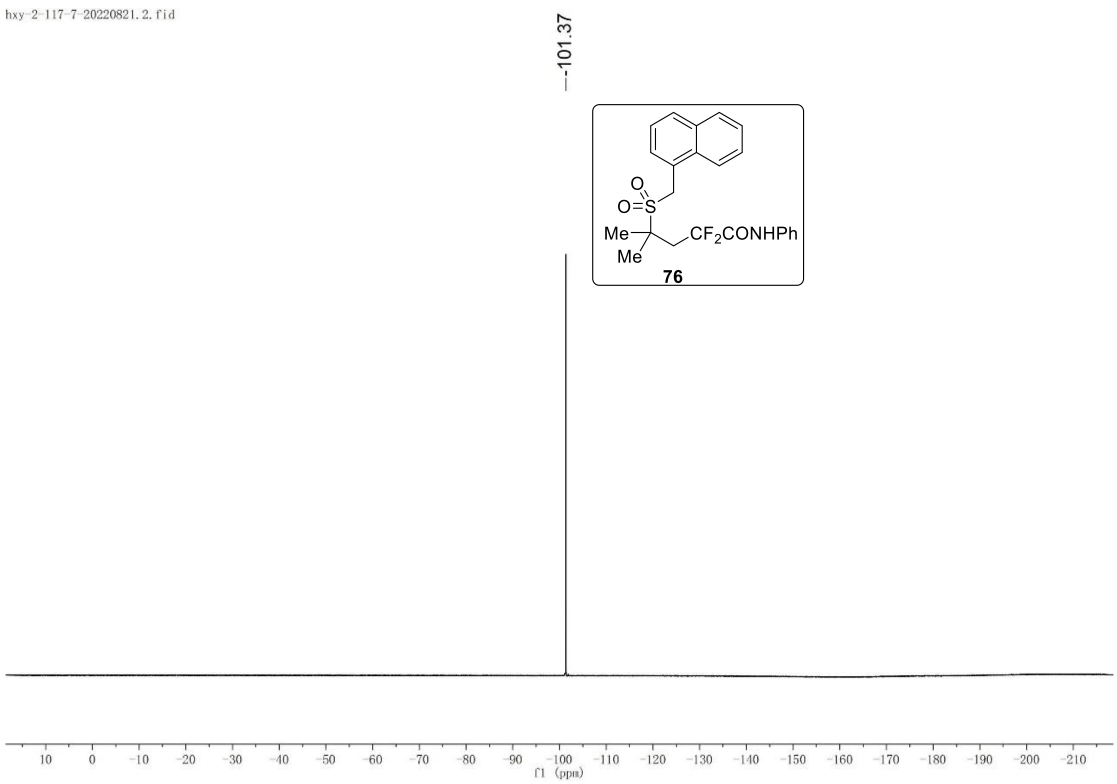

Supplementary Figure 272. ^{19}F NMR (376 MHz, $CDCl_3$) spectra of **76**

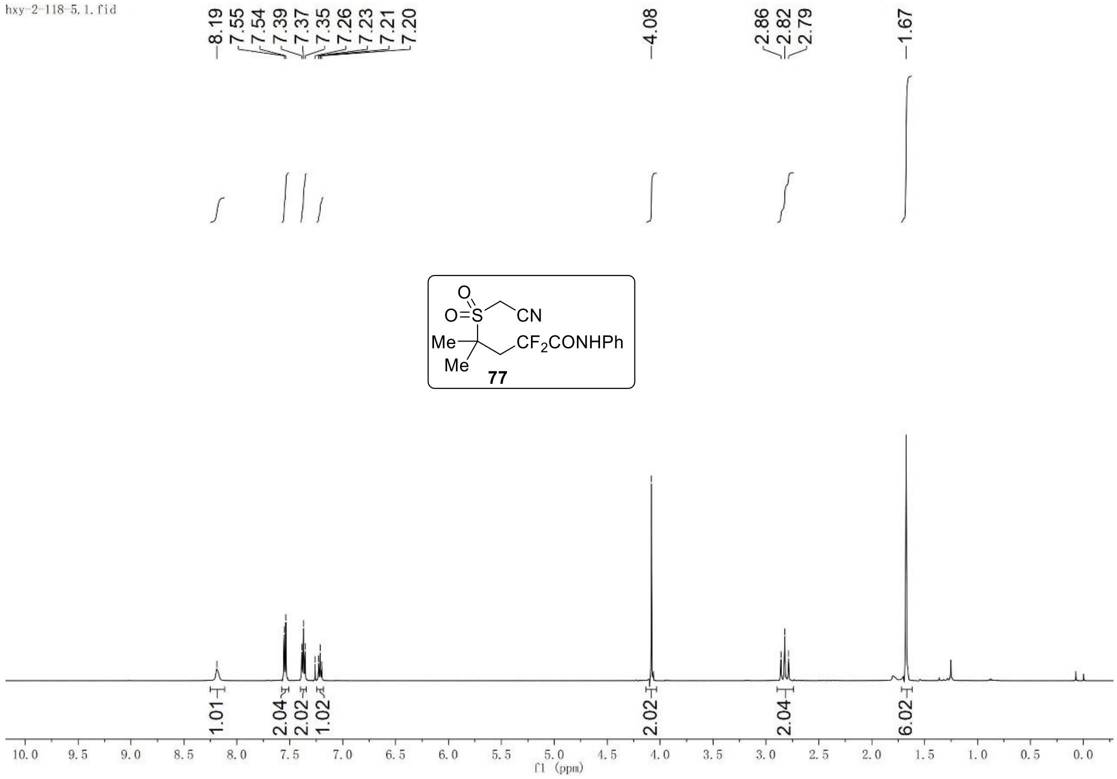

Supplementary Figure 273. ^{1}H NMR (500 MHz, $CDCl_3$) spectra of **77**

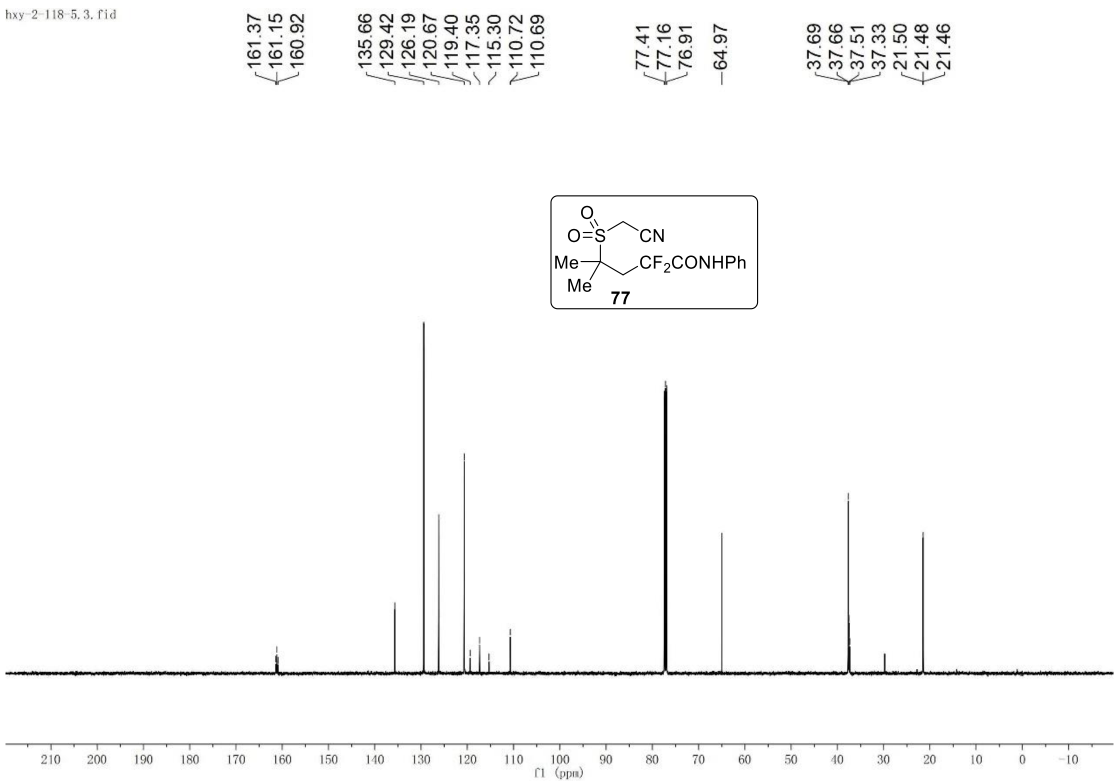

Supplementary Figure 274. ^{13}C NMR (126 MHz, $CDCl_3$) spectra of **77**

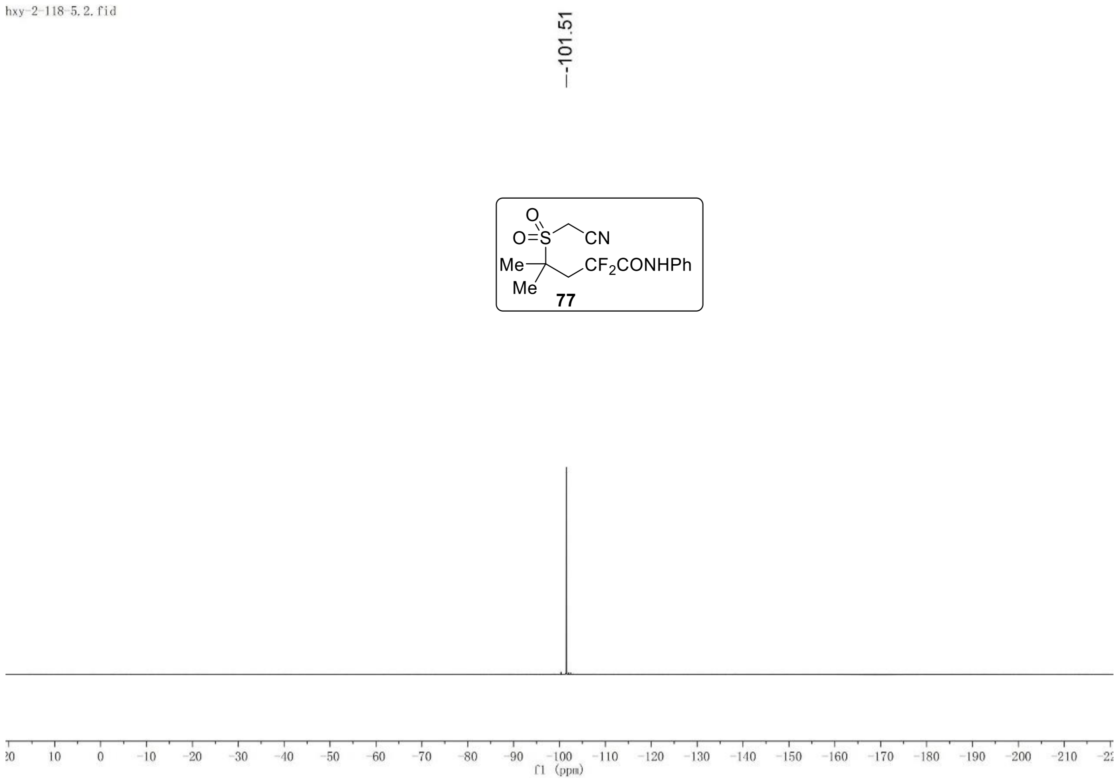

Supplementary Figure 275. ^{19}F NMR (471 MHz, $CDCl_3$) spectra of **77**

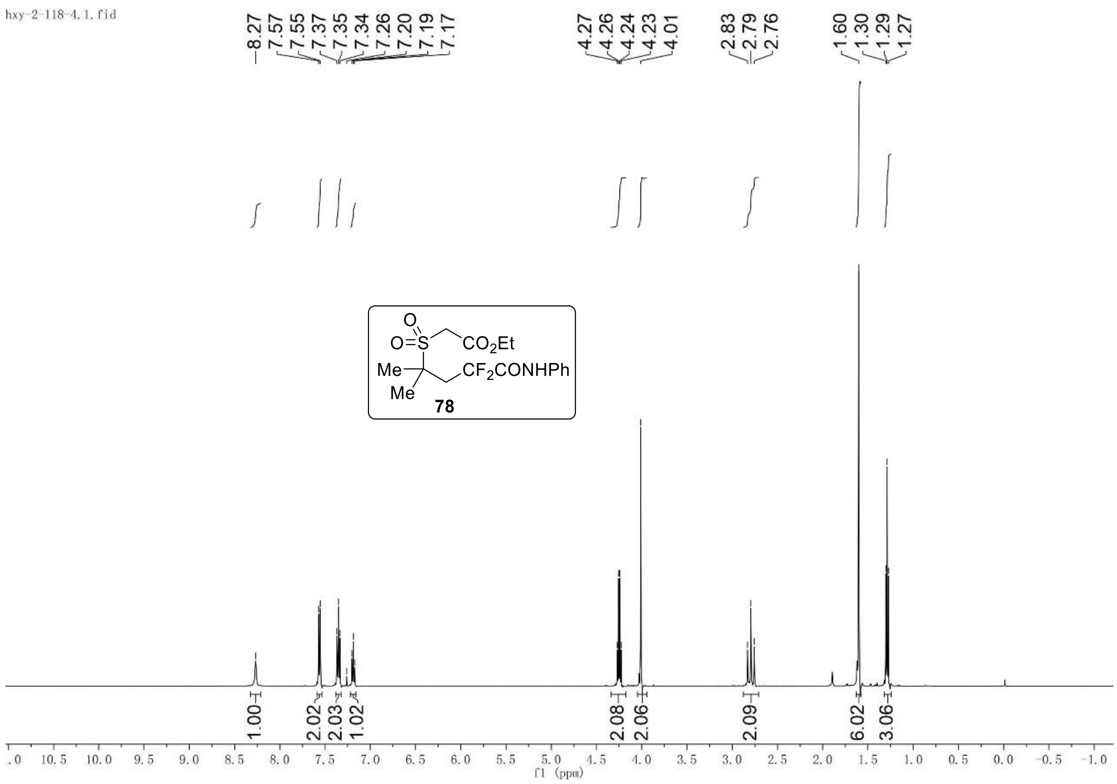

Supplementary Figure 276. ^{1}H NMR (500 MHz, $CDCl_3$) spectra of **78**

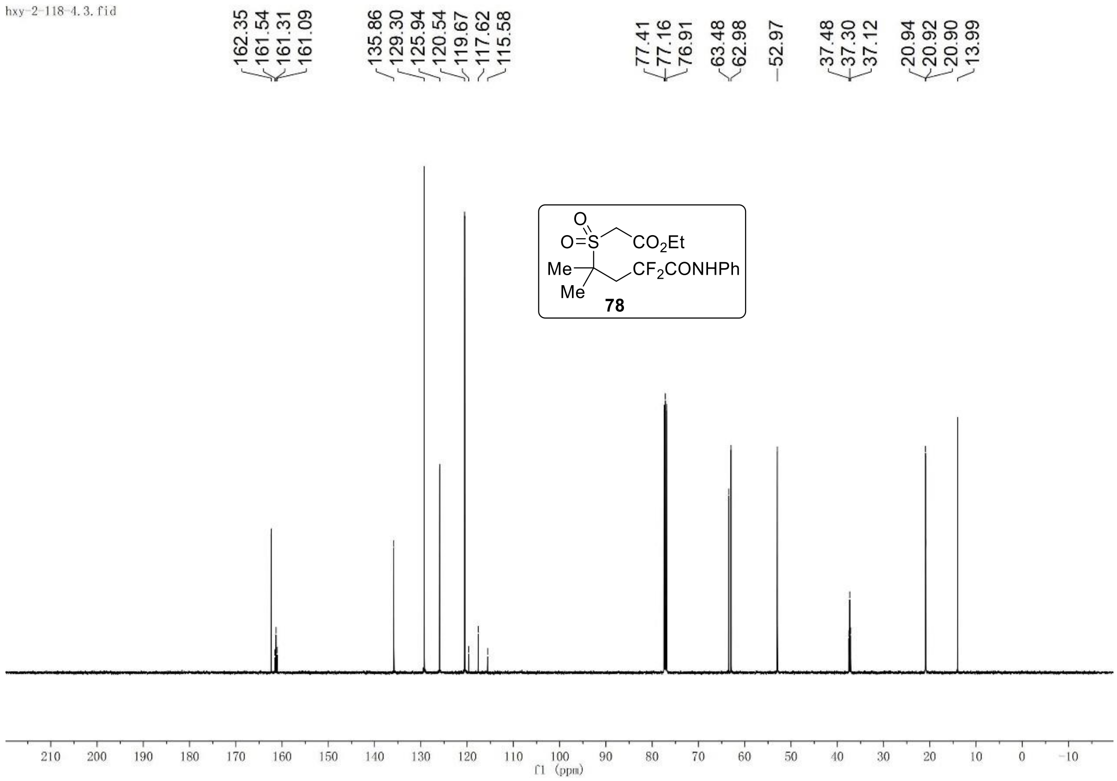

Supplementary Figure 277. ^{13}C NMR (126 MHz, $CDCl_3$) spectra of **78**

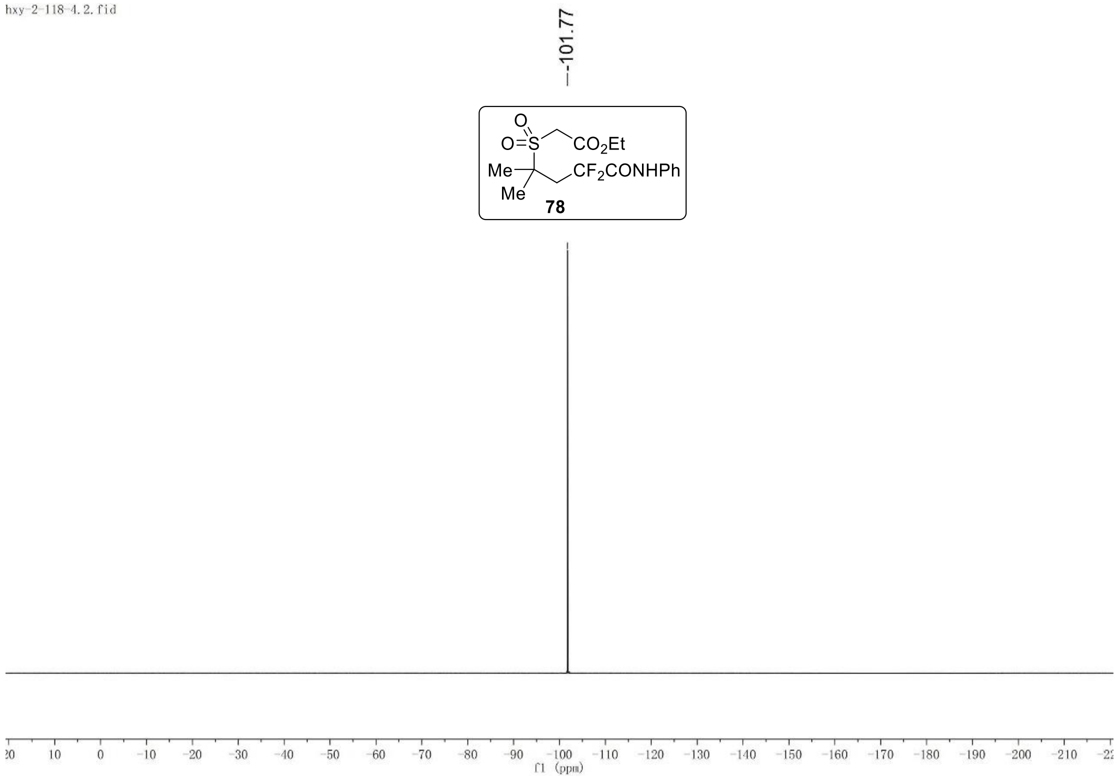

Supplementary Figure 278. ^{19}F NMR (471 MHz, $CDCl_3$) spectra of **78**

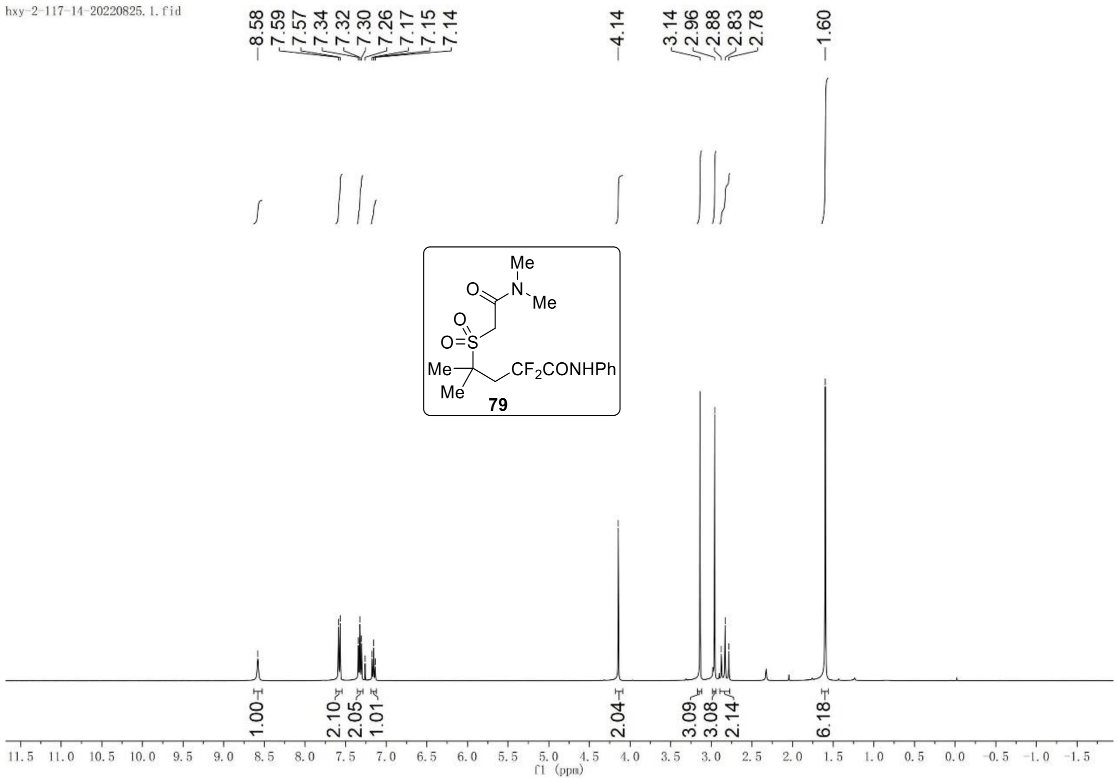

Supplementary Figure 279. ^{1}H NMR (400 MHz, $CDCl_3$) spectra of **79**

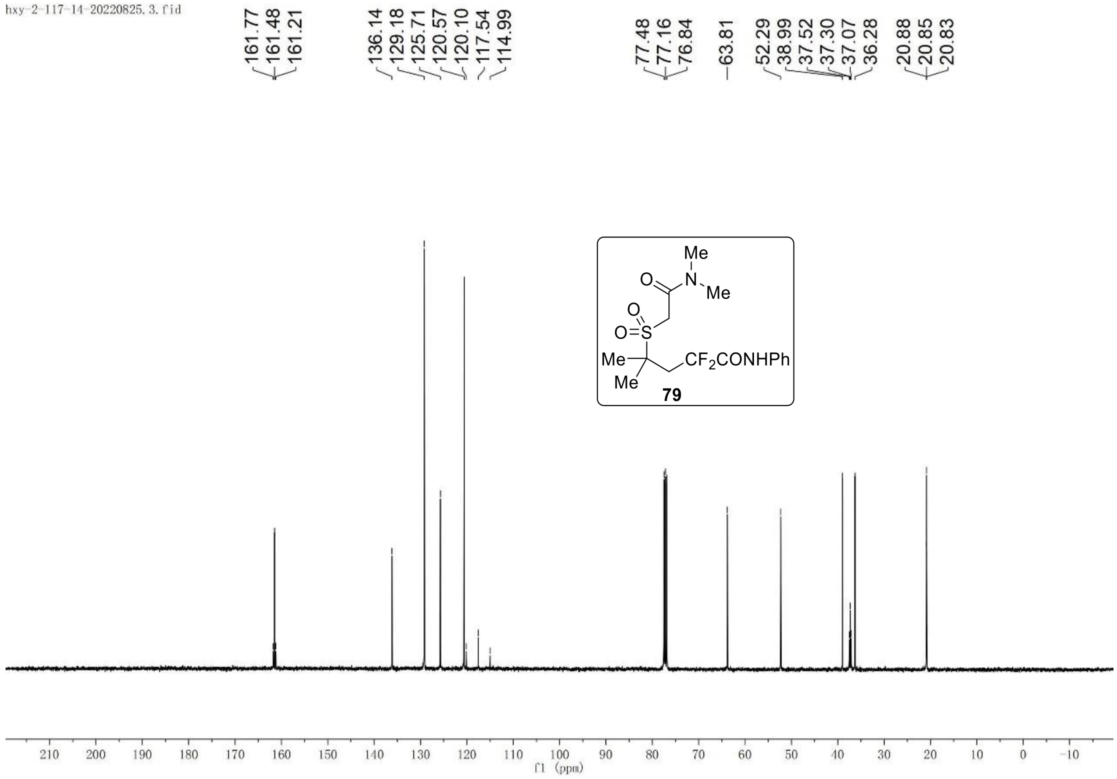

Supplementary Figure 280. ^{13}C NMR (101 MHz, $CDCl_3$) spectra of **79**

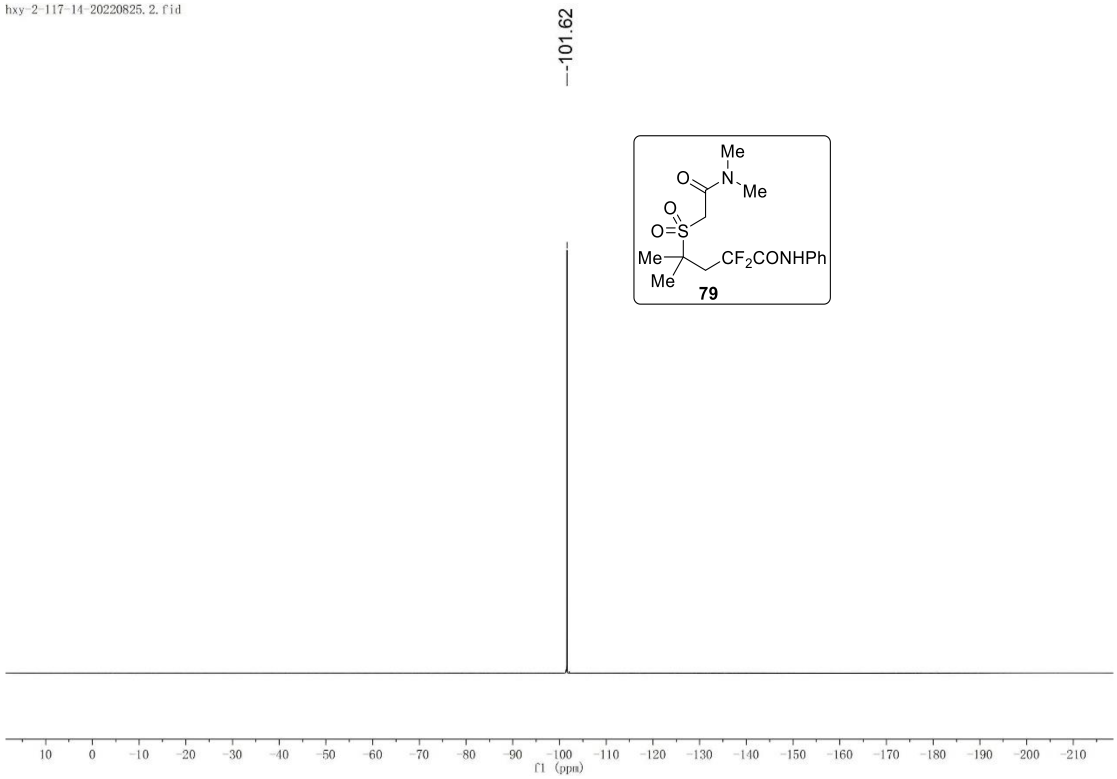

Supplementary Figure 281. ^{19}F NMR (376 MHz, $CDCl_3$) spectra of **79**

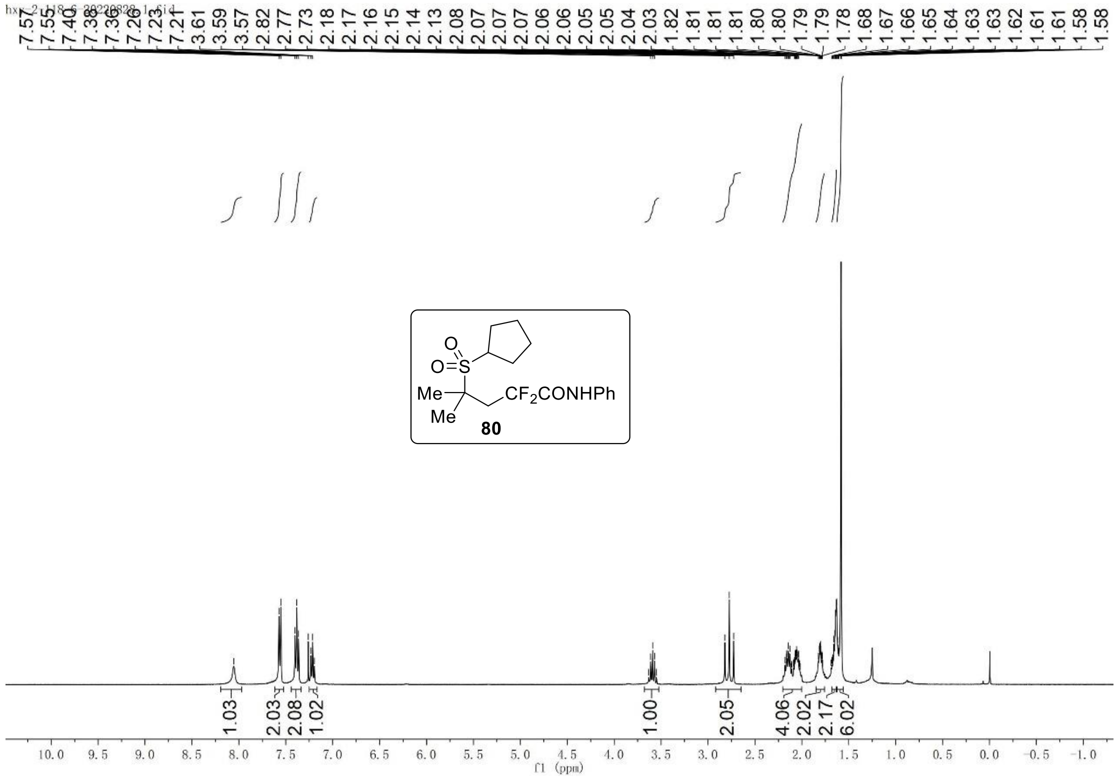

Supplementary Figure 282. ^{1}H NMR (400 MHz, $CDCl_3$) spectra of **80**

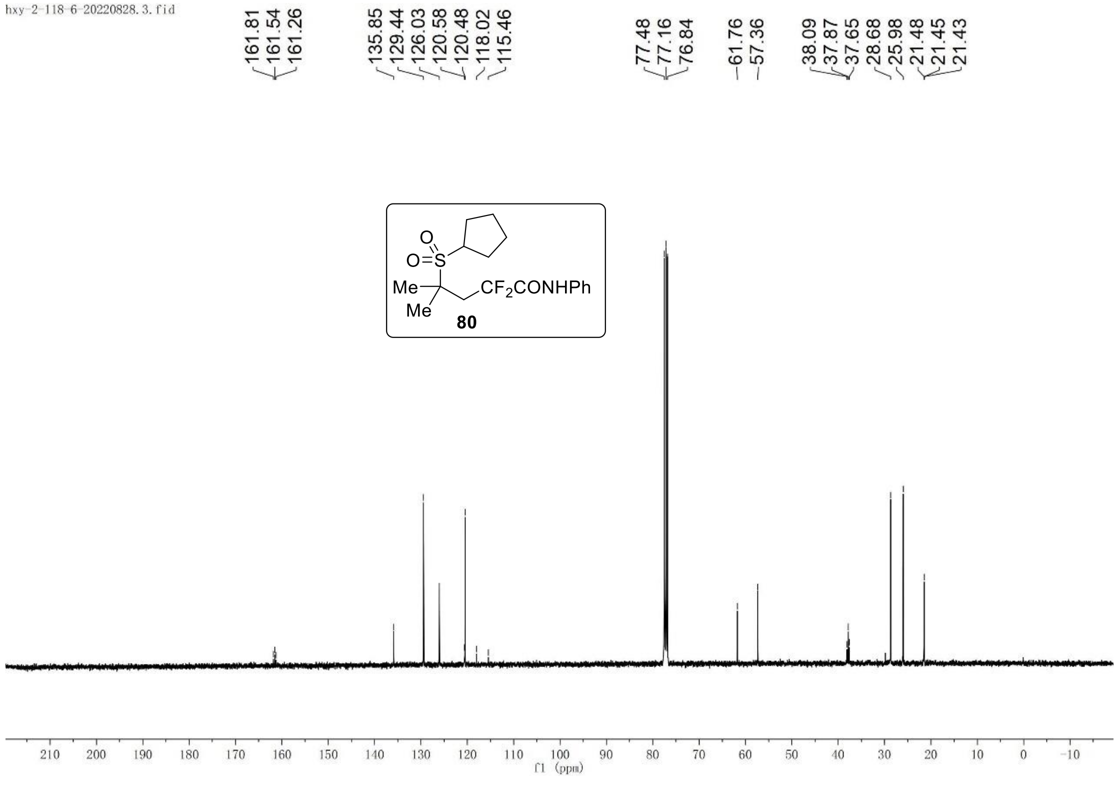

Supplementary Figure 283. ^{13}C NMR (101 MHz, $CDCl_3$) spectra of **80**

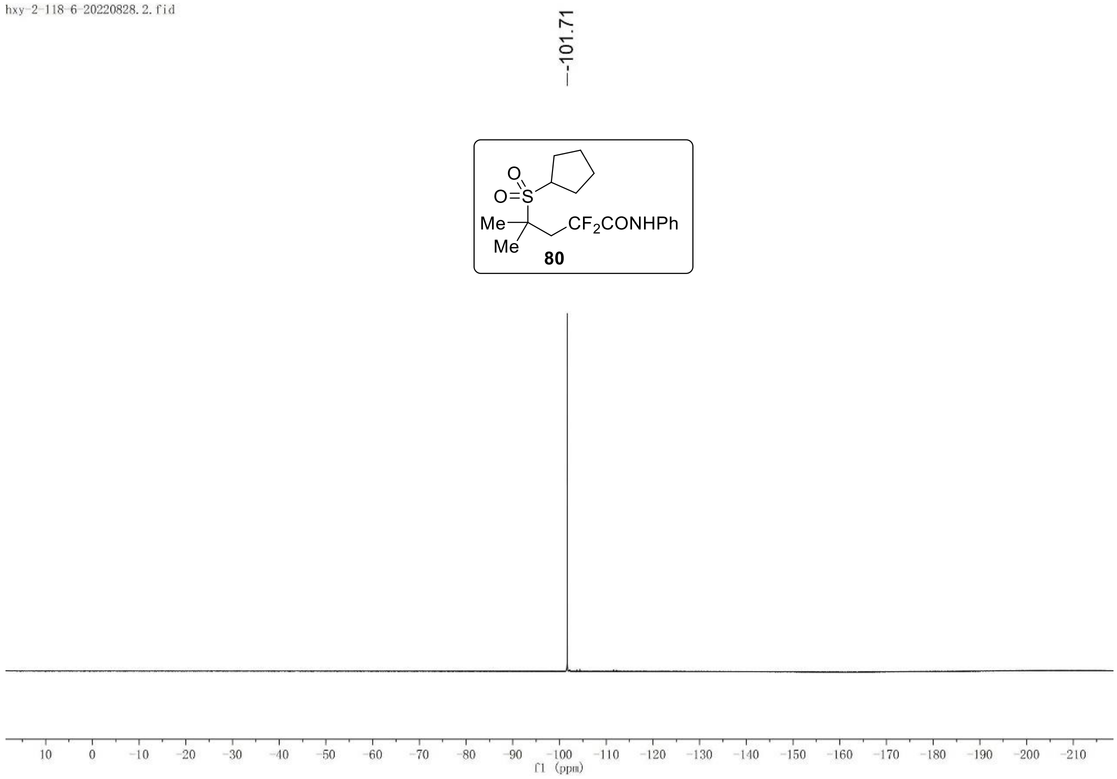

Supplementary Figure 284. ^{19}F NMR (376 MHz, $CDCl_3$) spectra of **80**

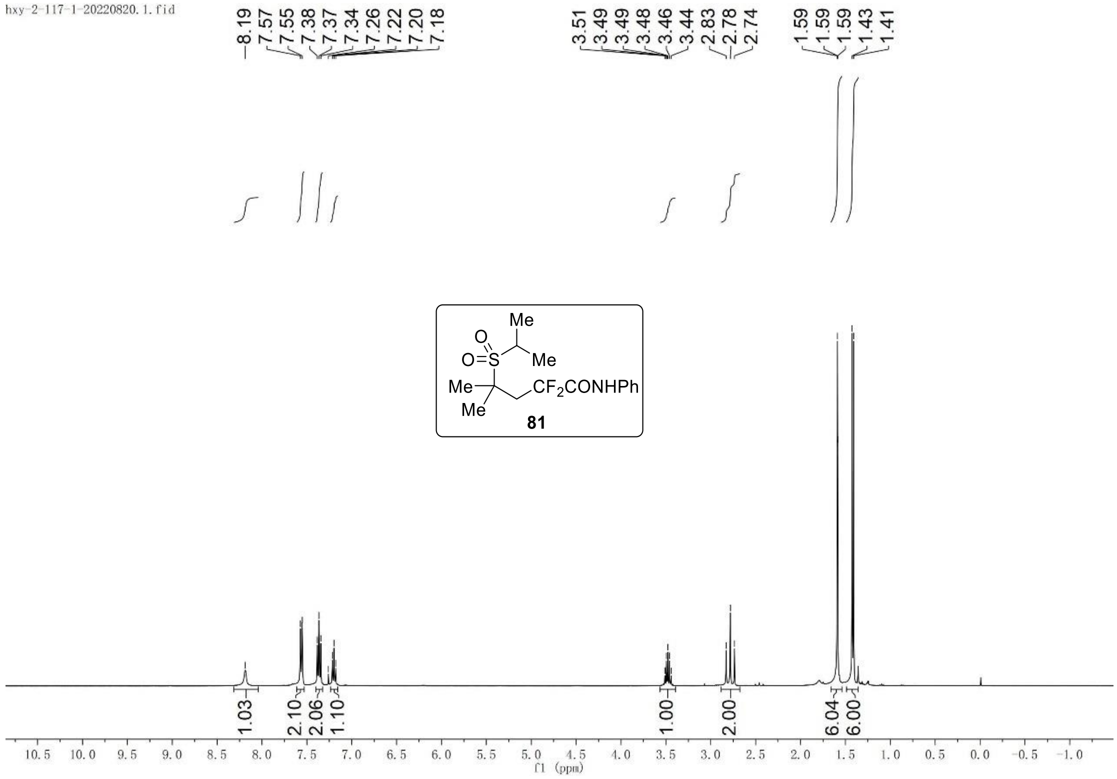

Supplementary Figure 285. ^{1}H NMR (400 MHz, $CDCl_3$) spectra of **81**

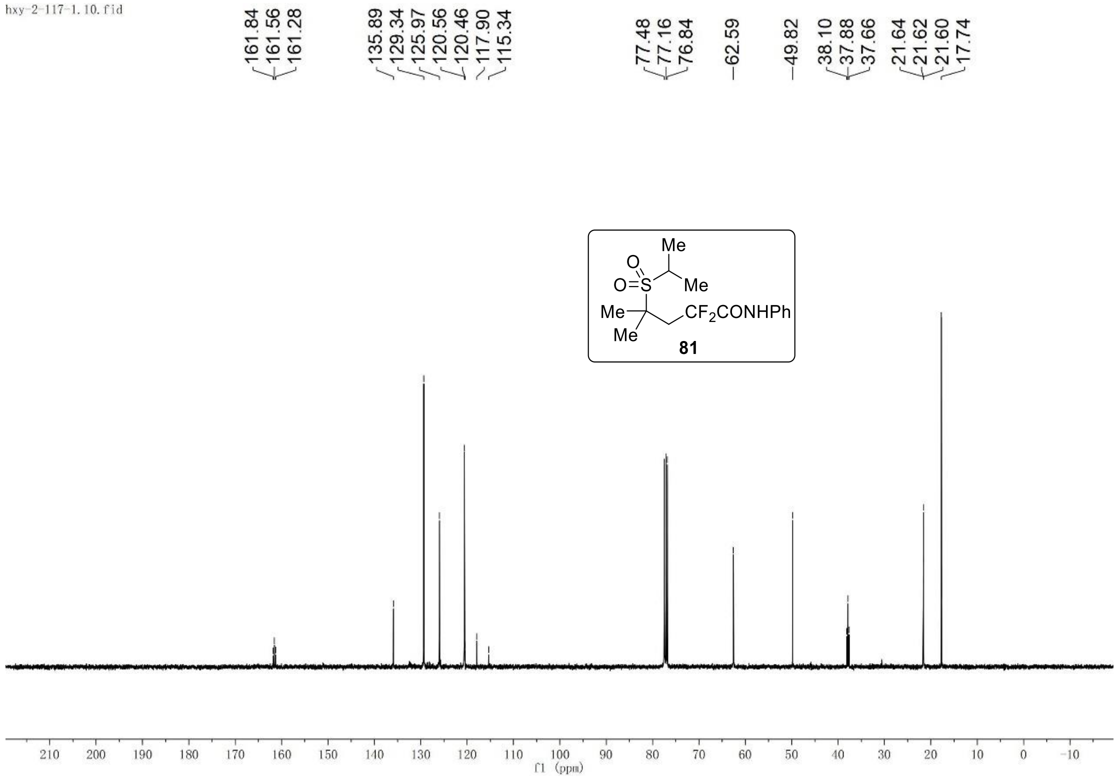

Supplementary Figure 286. ^{13}C NMR (101 MHz, $CDCl_3$) spectra of **81**

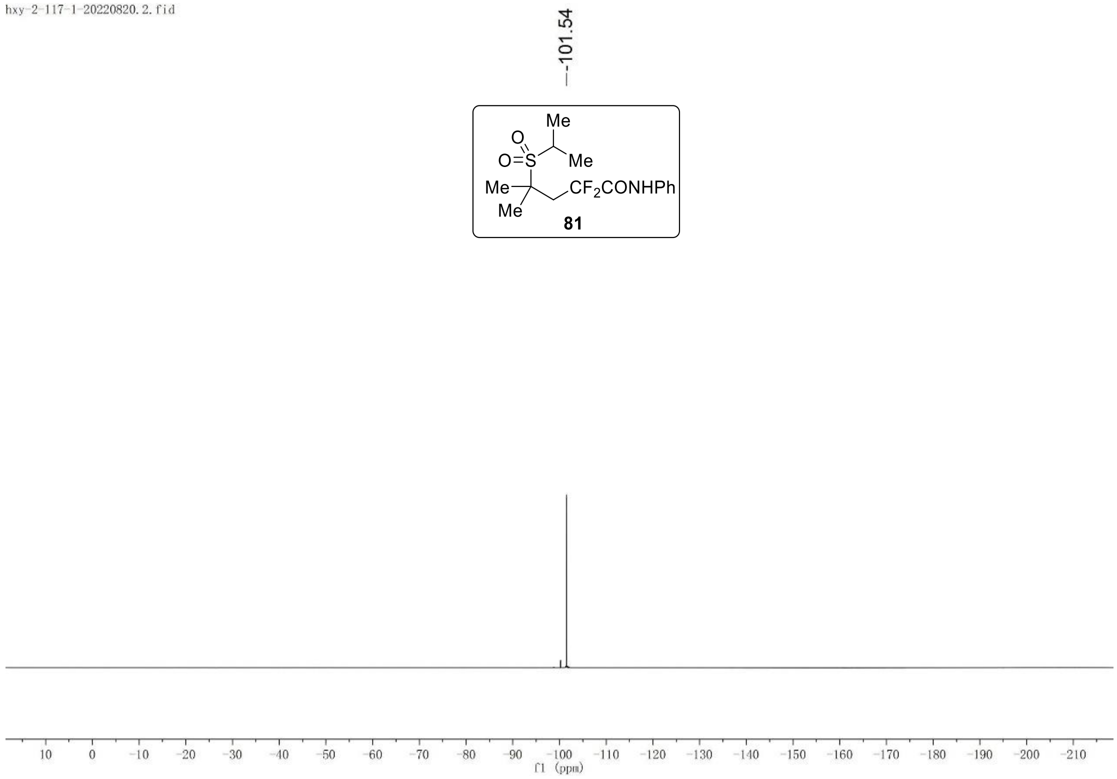

Supplementary Figure 287. ^{19}F NMR (376 MHz, $CDCl_3$) spectra of **81**

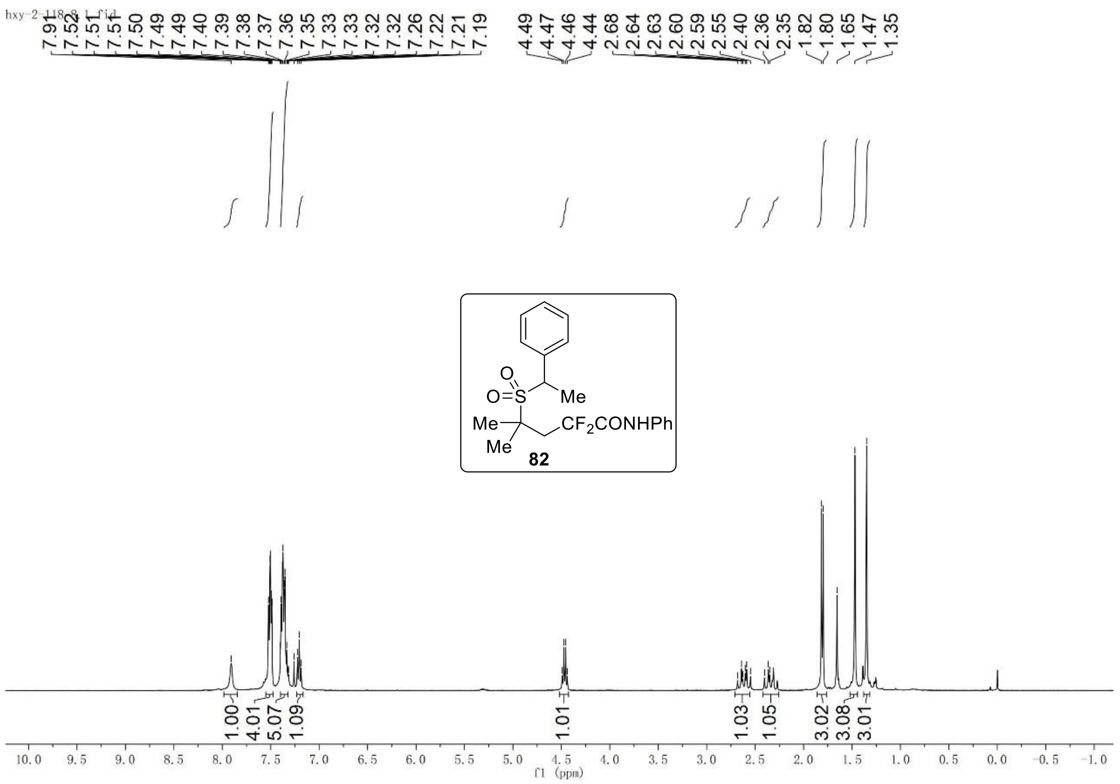

Supplementary Figure 288. ^{1}H NMR (400 MHz, $CDCl_3$) spectra of **82**

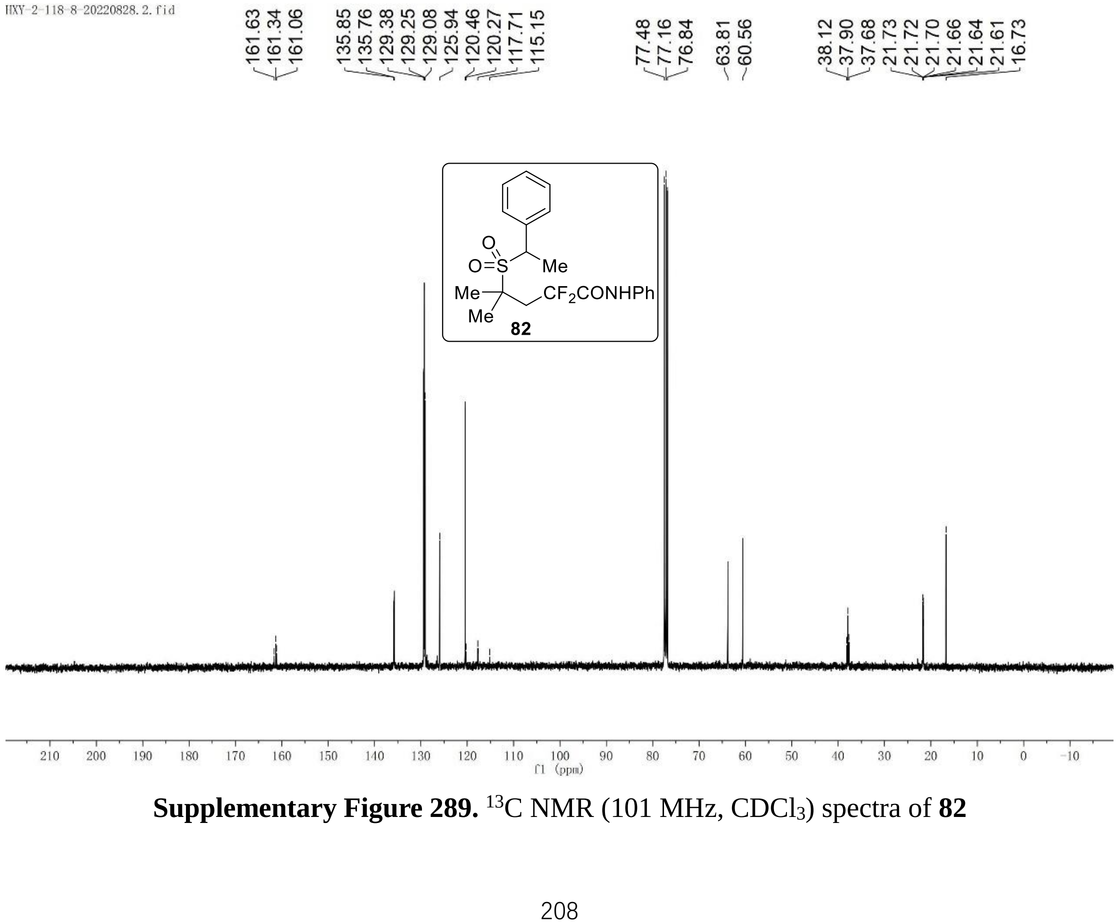

Supplementary Figure 289. ^{13}C NMR (101 MHz, $CDCl_3$) spectra of **82**

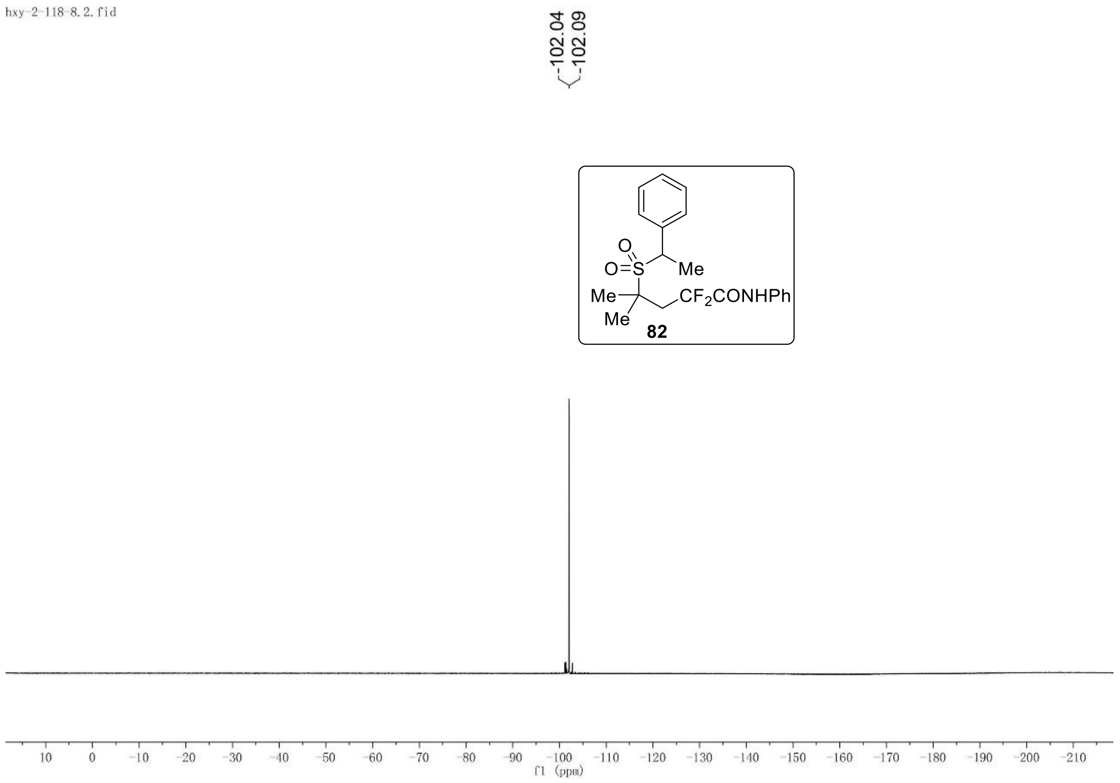

Supplementary Figure 290. ^{19}F NMR (376 MHz, $CDCl_3$) spectra of **82**

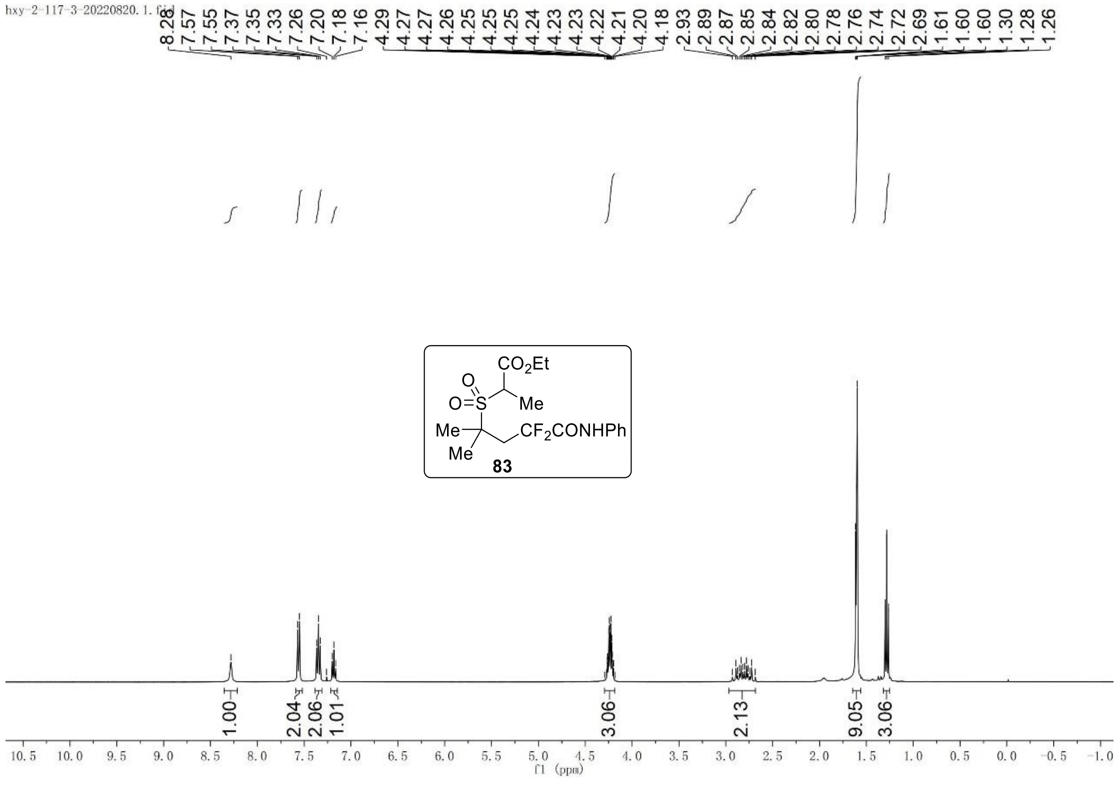

Supplementary Figure 291. ^{1}H NMR (400 MHz, $CDCl_3$) spectra of **83**

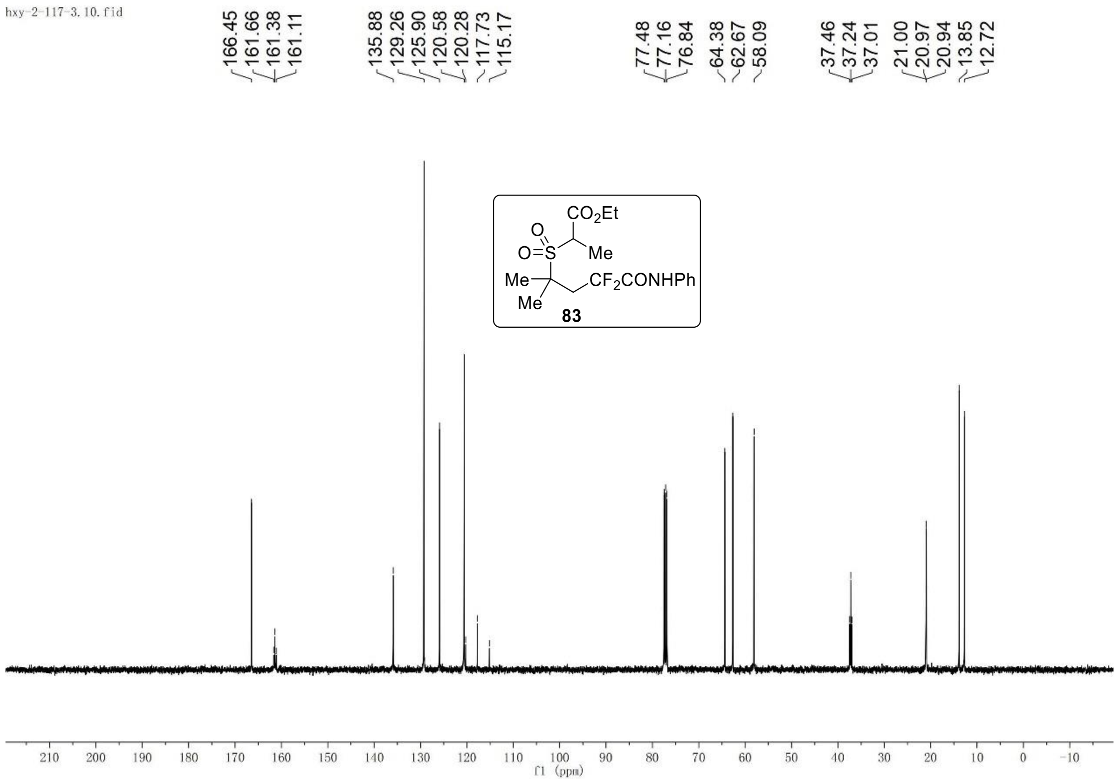

Supplementary Figure 292. ^{13}C NMR (101 MHz, $CDCl_3$) spectra of **83**

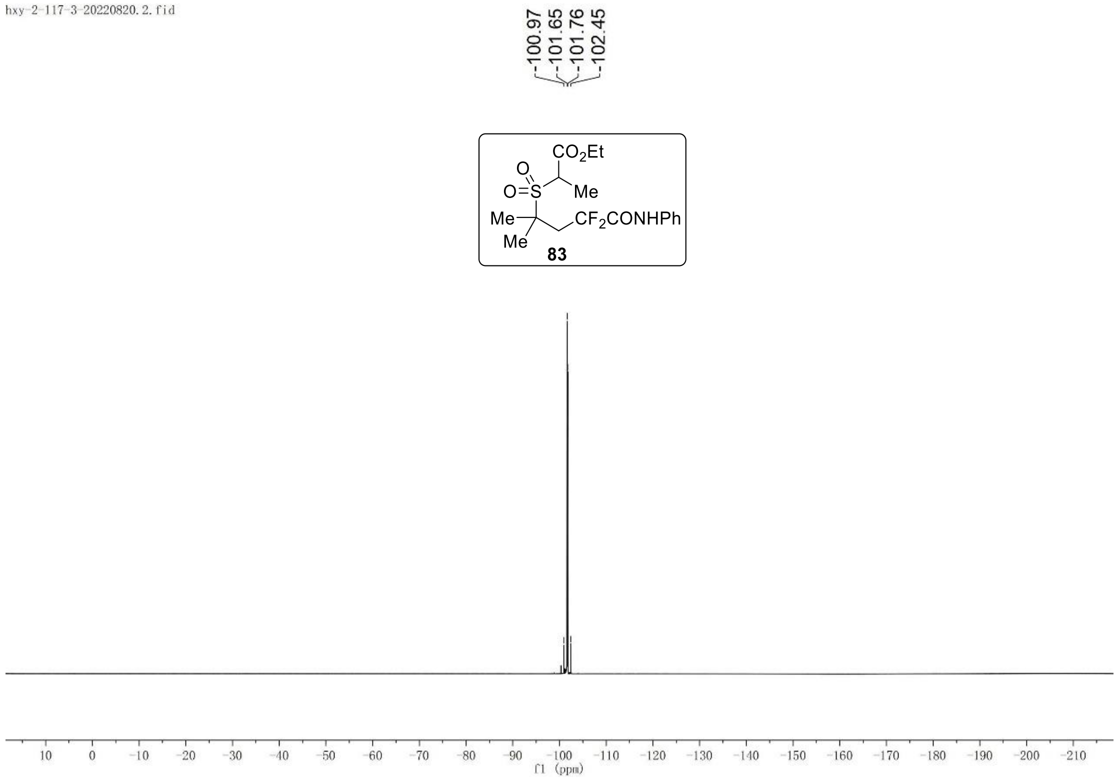

Supplementary Figure 293. ^{19}F NMR (376 MHz, $CDCl_3$) spectra of **83**

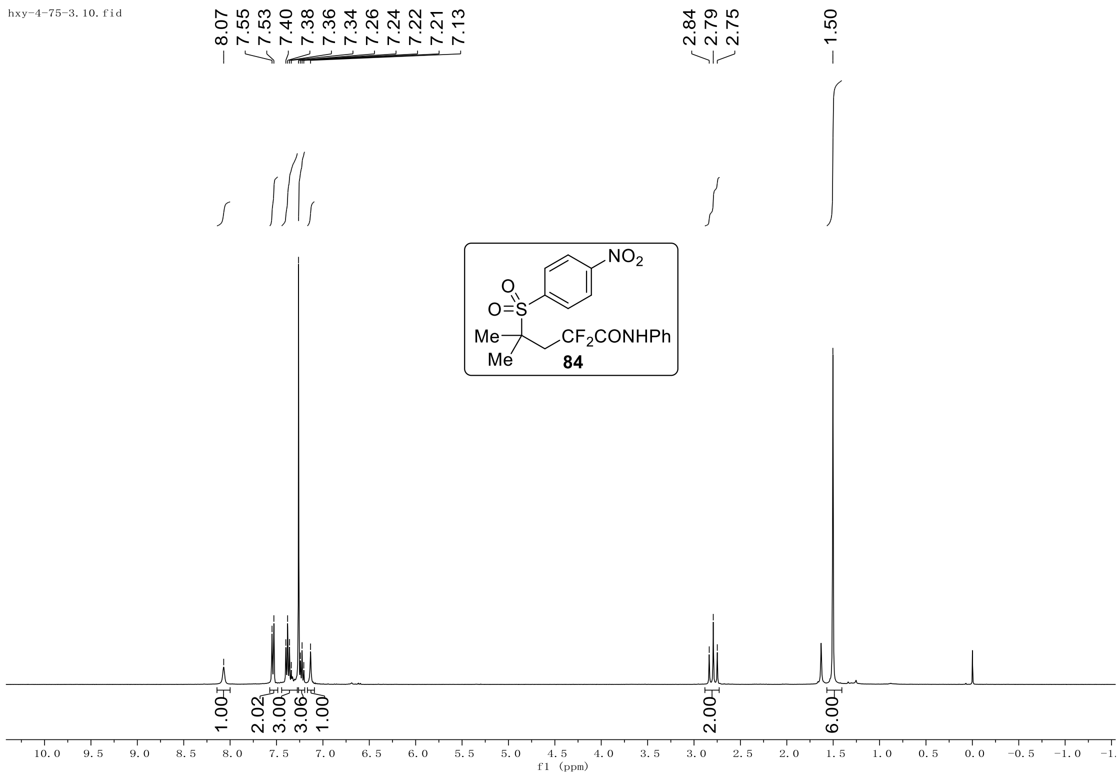

Supplementary Figure 294. ^{1}H NMR (400 MHz, $CDCl_3$) spectra of **84**

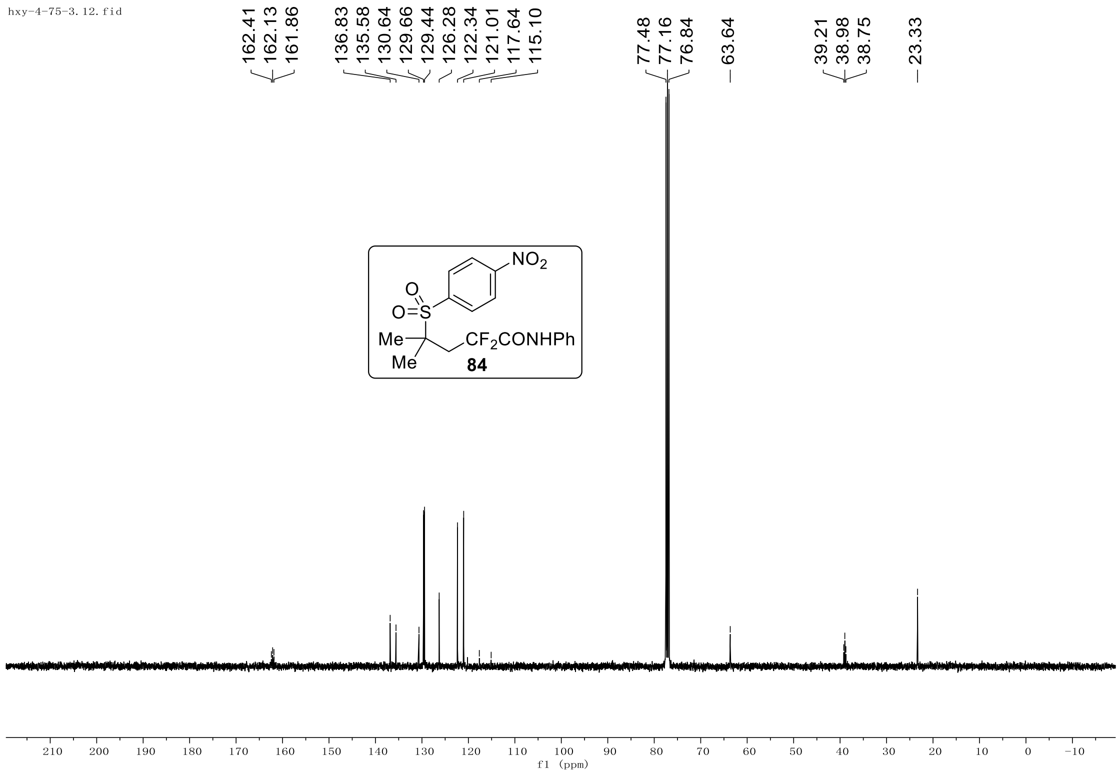

Supplementary Figure 295. ^{13}C NMR (101 MHz, $CDCl_3$) spectra of **84**

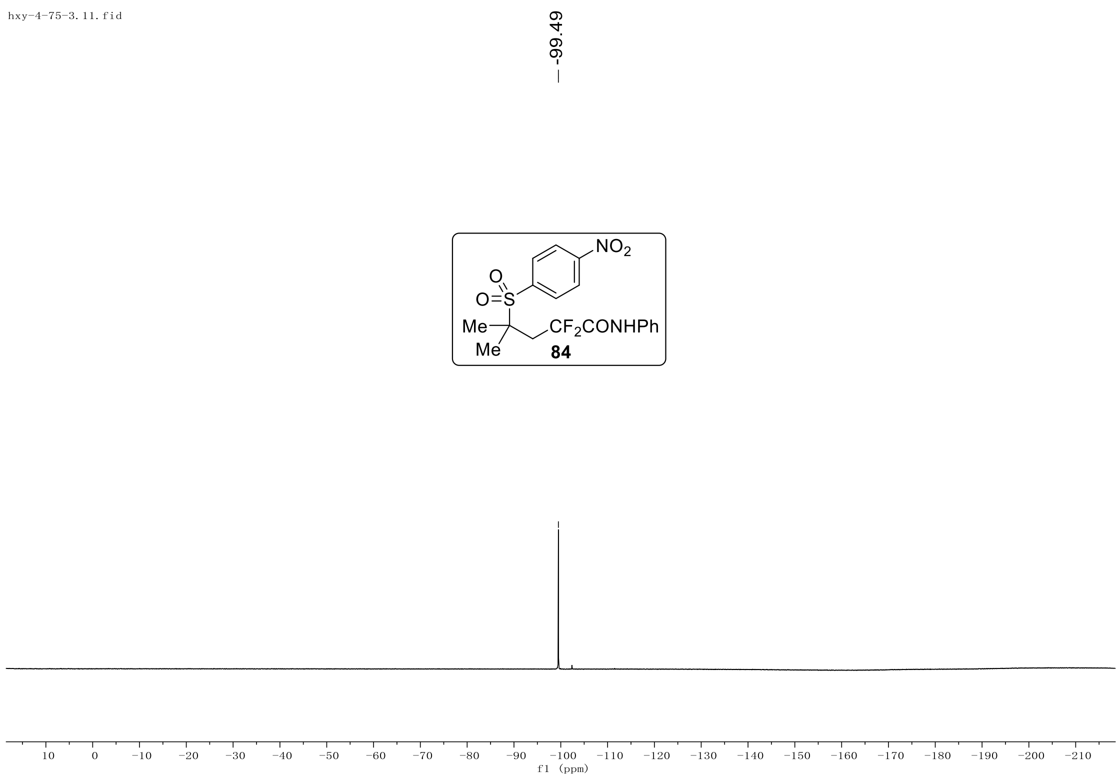

Supplementary Figure 296. ^{19}F NMR (376 MHz, $CDCl_3$) spectra of **84**

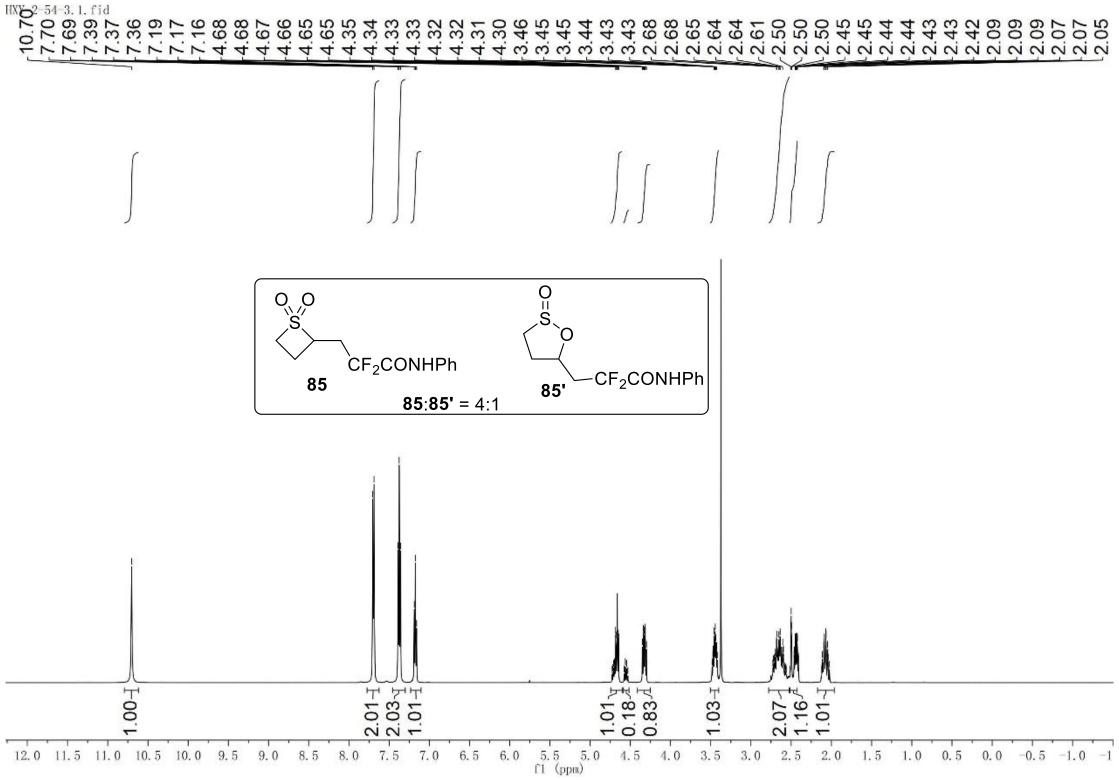

Supplementary Figure 297. ^{1}H NMR (500 MHz, DMSO-d_6) spectra of **85** and **85’**

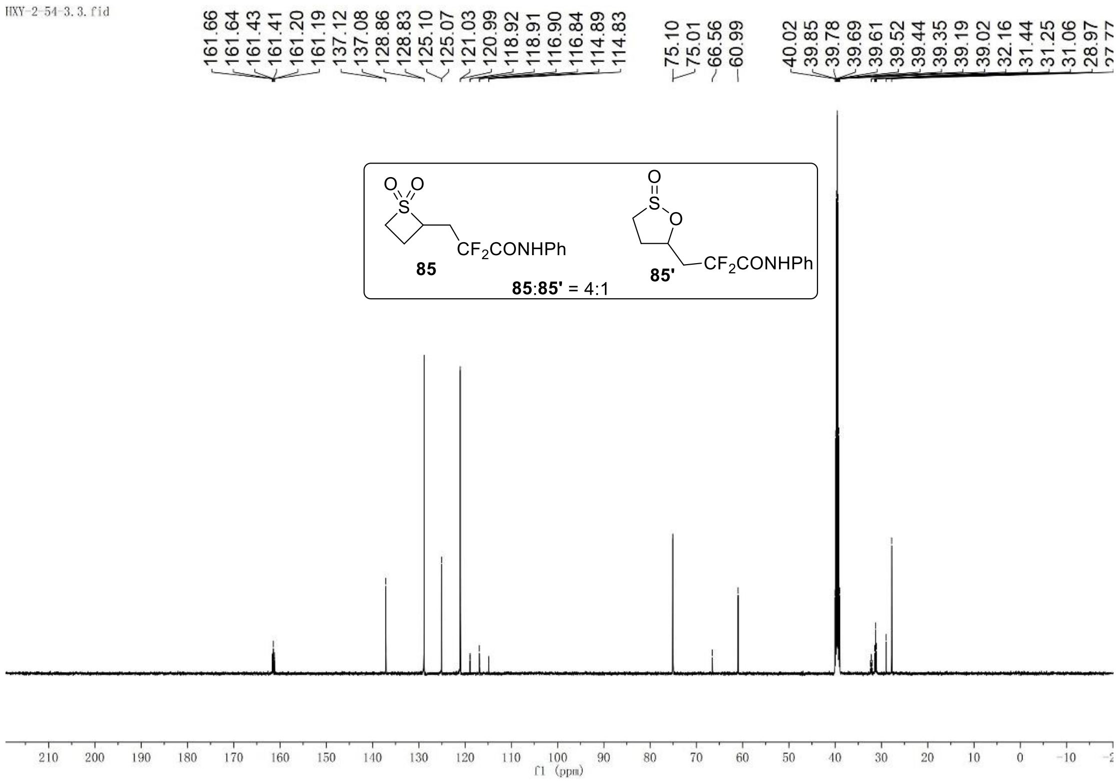

Supplementary Figure 298. ^{13}C NMR (126 MHz, DMSO-d_6) spectra of **85** and **85’**

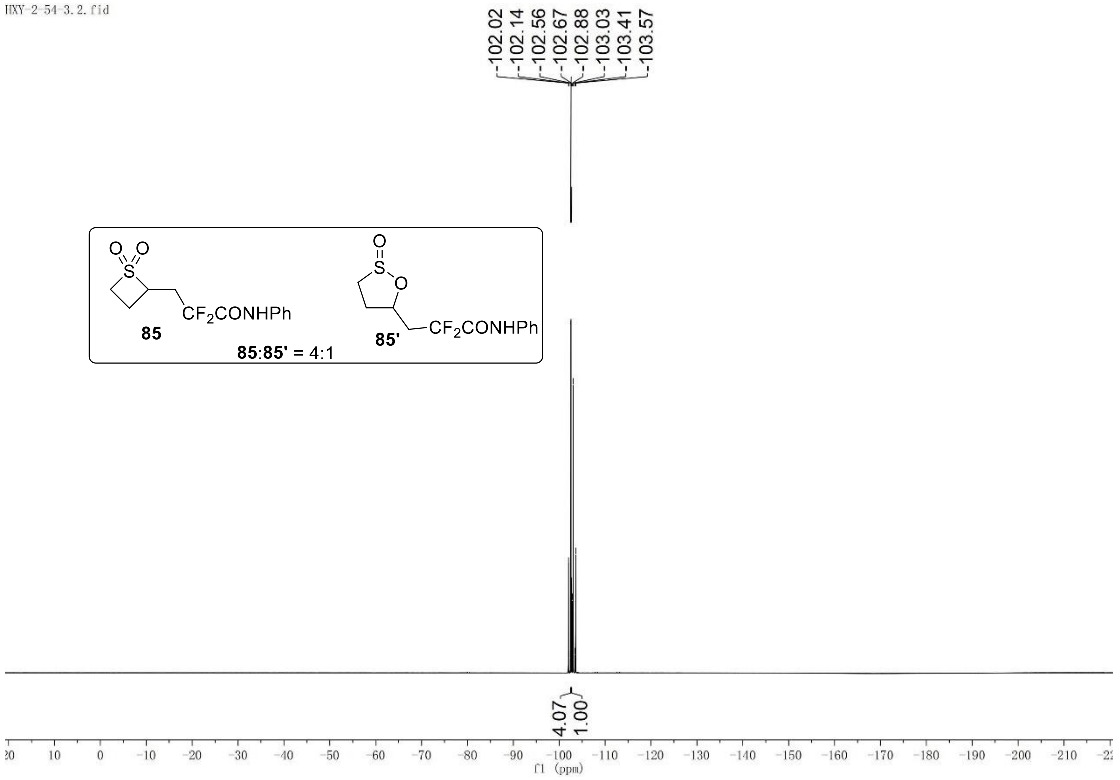

Supplementary Figure 299. ^{19}F NMR (471 MHz, DMSO-d_6) spectra of **85** and **85’**

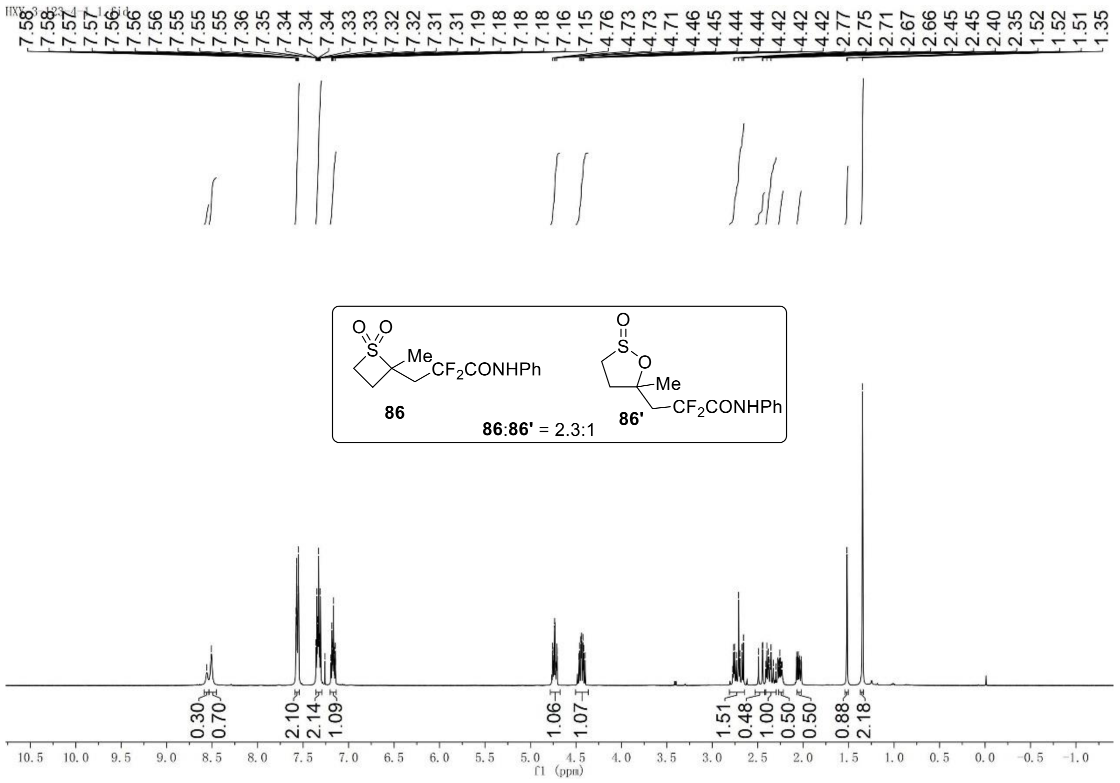

Supplementary Figure 300. ^{1}H NMR (400 MHz, $CDCl_3$) spectra of **86** and **86’**

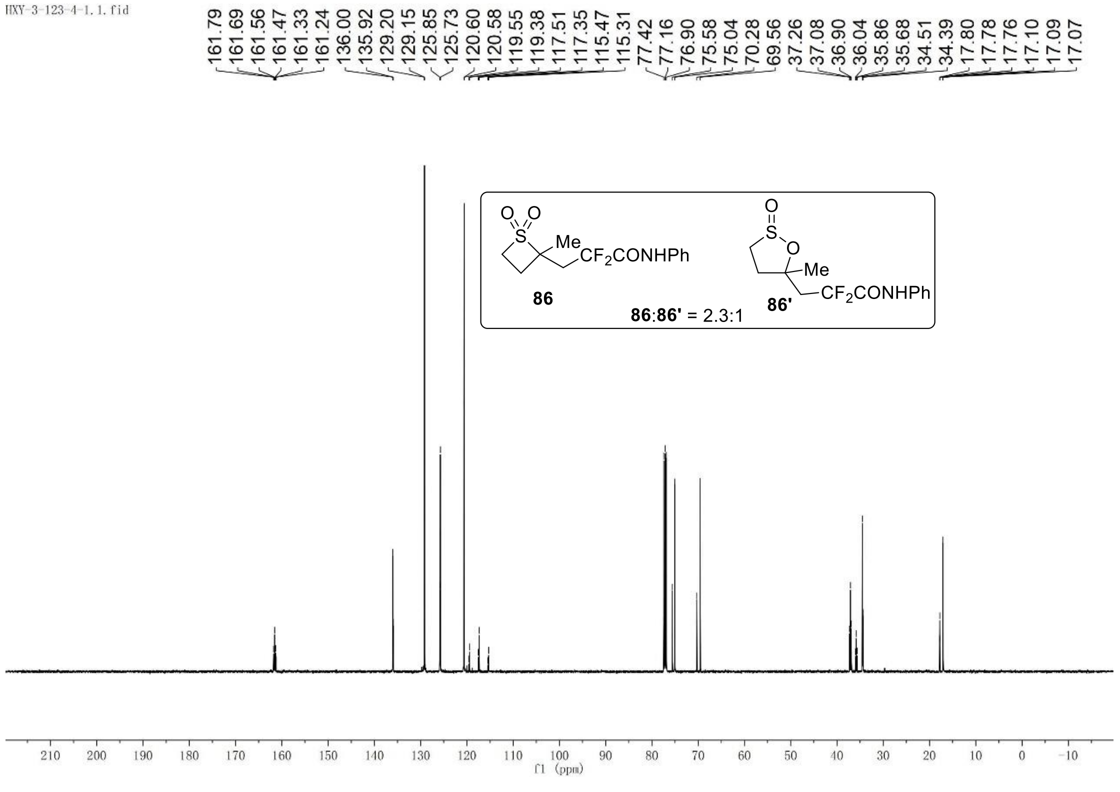

Supplementary Figure 301. ^{13}C NMR (126 MHz, $CDCl_3$) spectra of **86** and **86’**

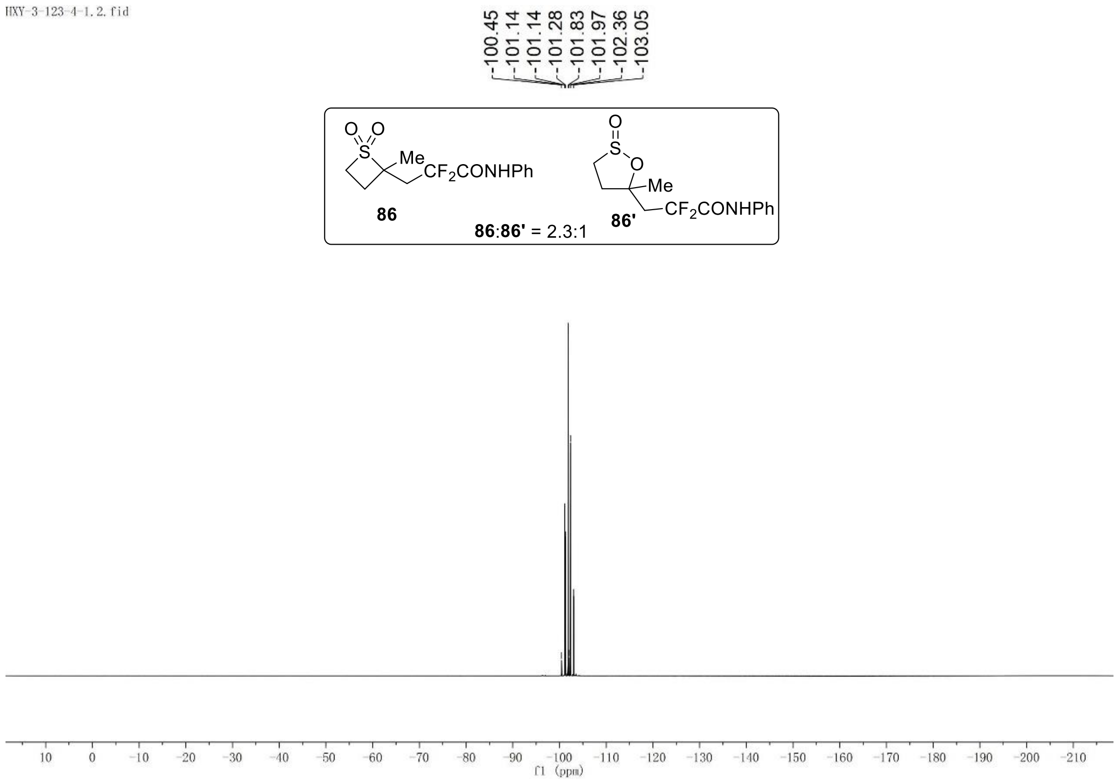

Supplementary Figure 302. ^{19}F NMR (376 MHz, $CDCl_3$) spectra of **86** and **86’**

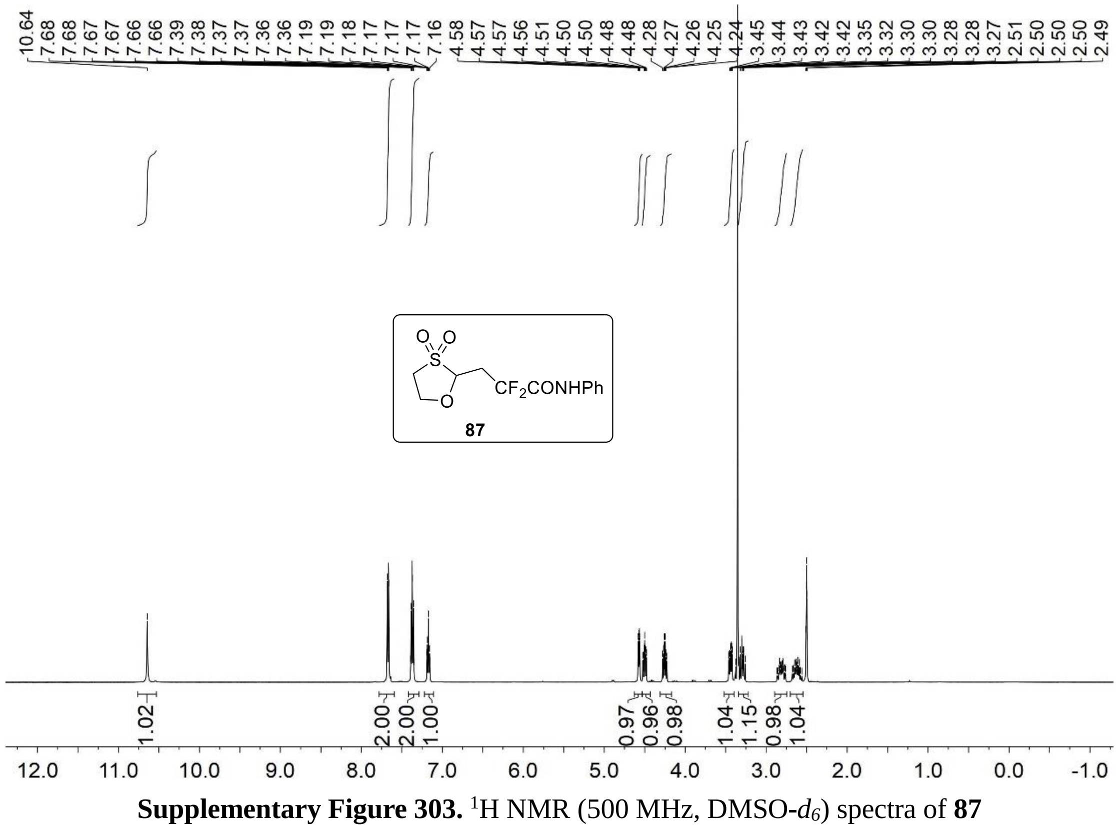

Supplementary Figure 303. ^{1}H NMR (500 MHz, DMSO-d_6) spectra of **87**

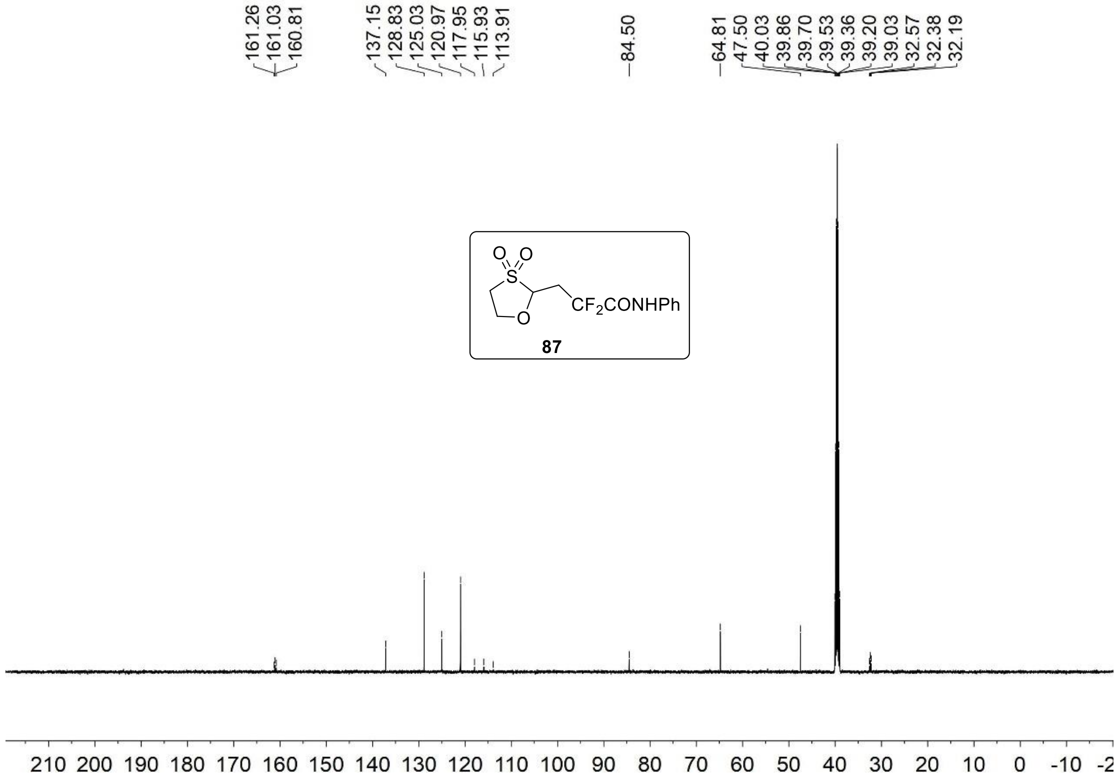

Supplementary Figure 304. ^{13}C NMR (126 MHz, DMSO-d_6) spectra of **87**

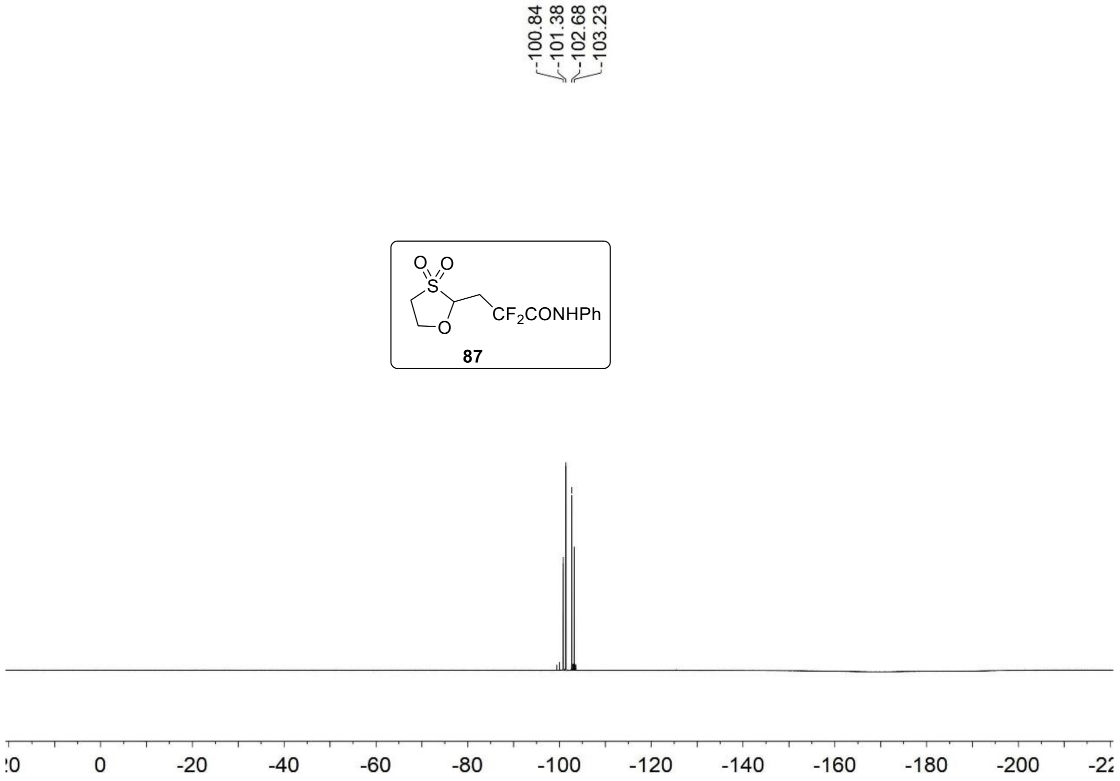

Supplementary Figure 305. ^{19}F NMR (471 MHz, DMSO-d_6) spectra of **87**

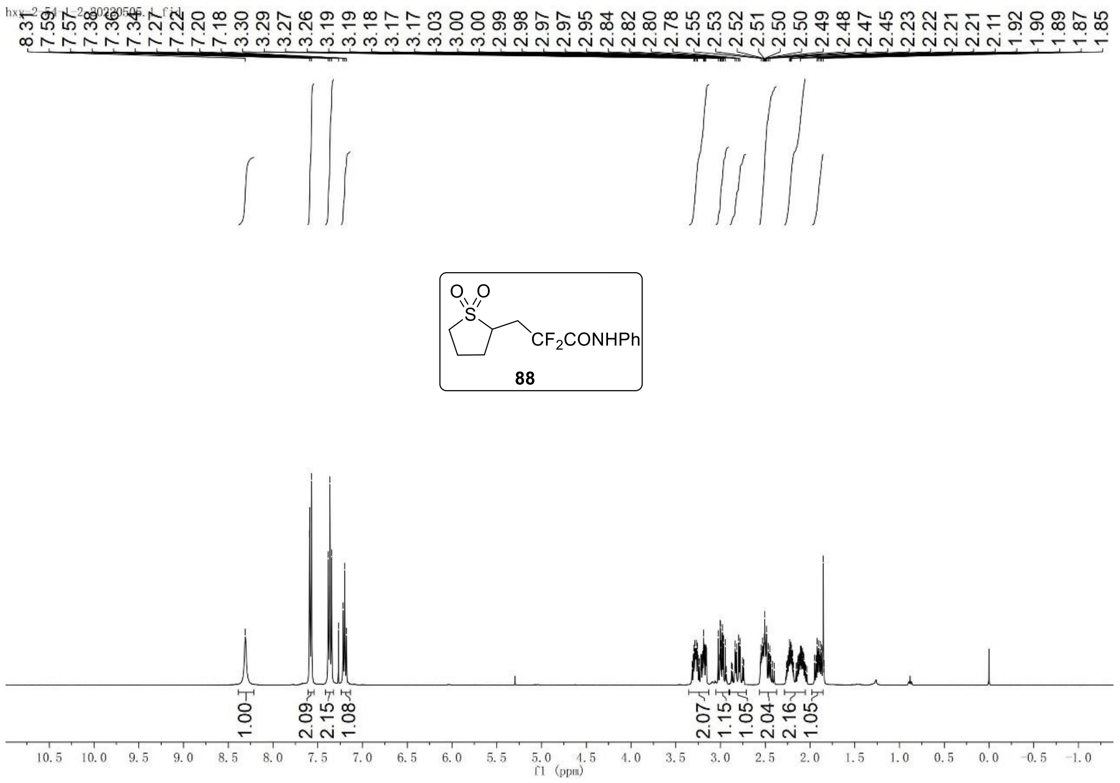

Supplementary Figure 306. ^{1}H NMR (400 MHz, $CDCl_3$) spectra of **88**

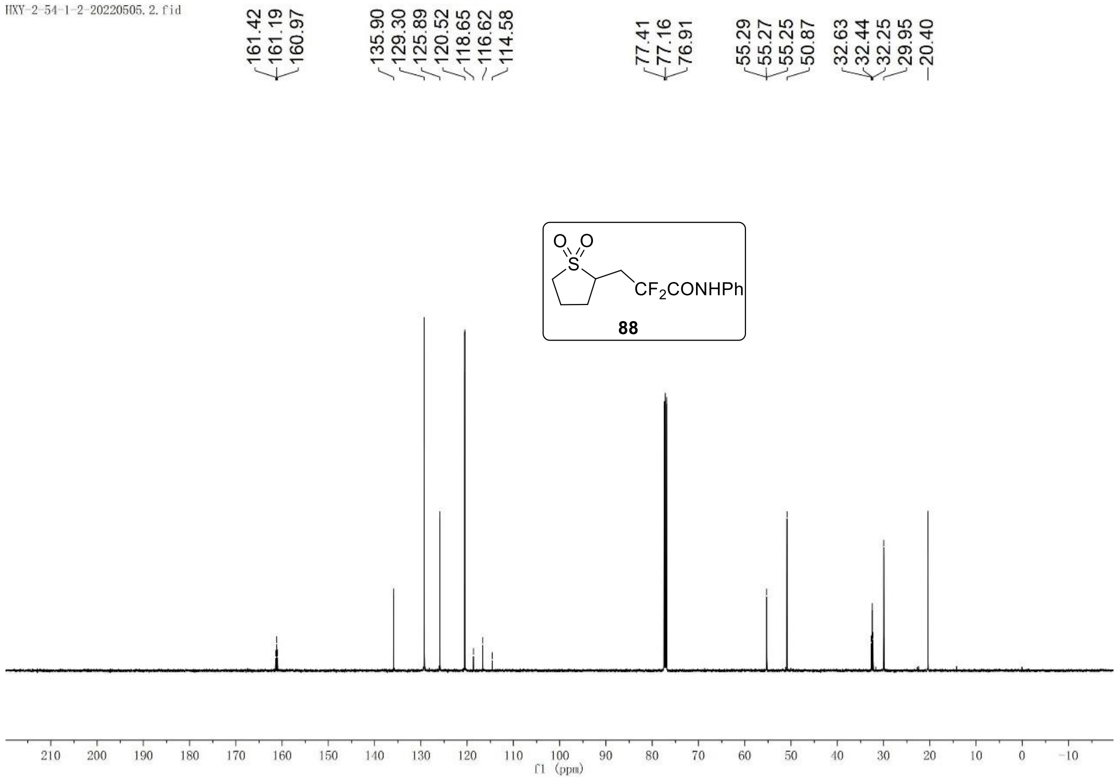

Supplementary Figure 307. ^{13}C NMR (126 MHz, $CDCl_3$) spectra of **88**

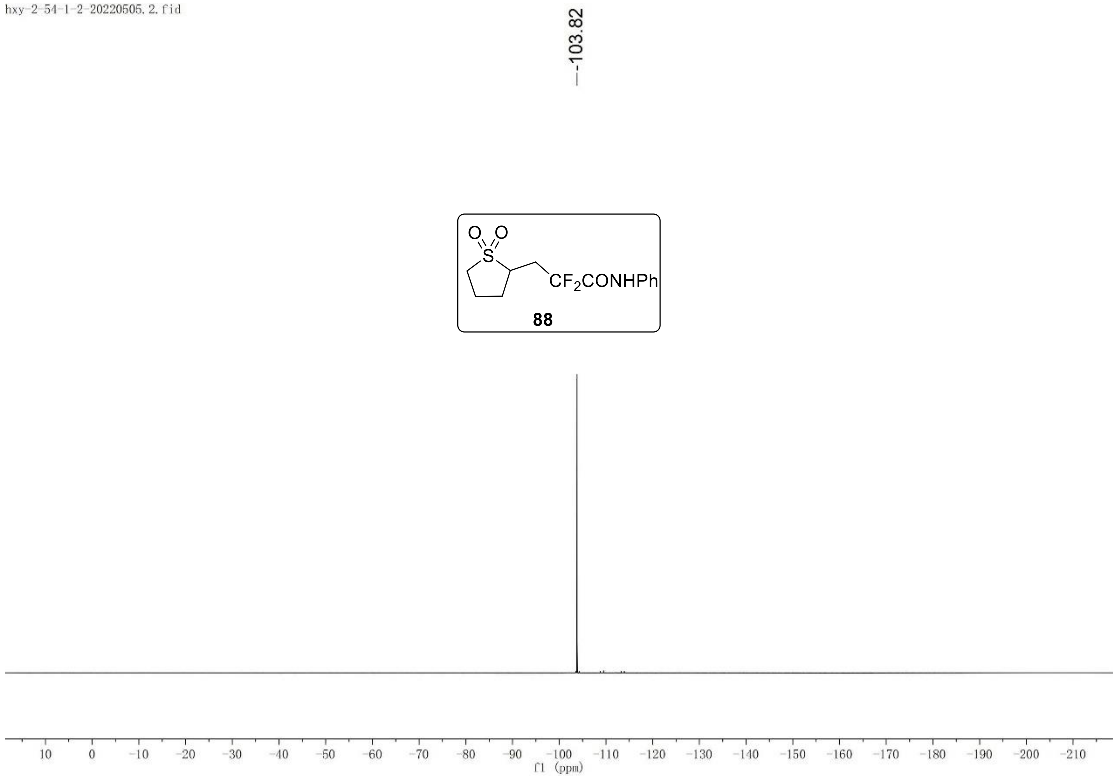

Supplementary Figure 308. ^{19}F NMR (376 MHz, $CDCl_3$) spectra of **88**

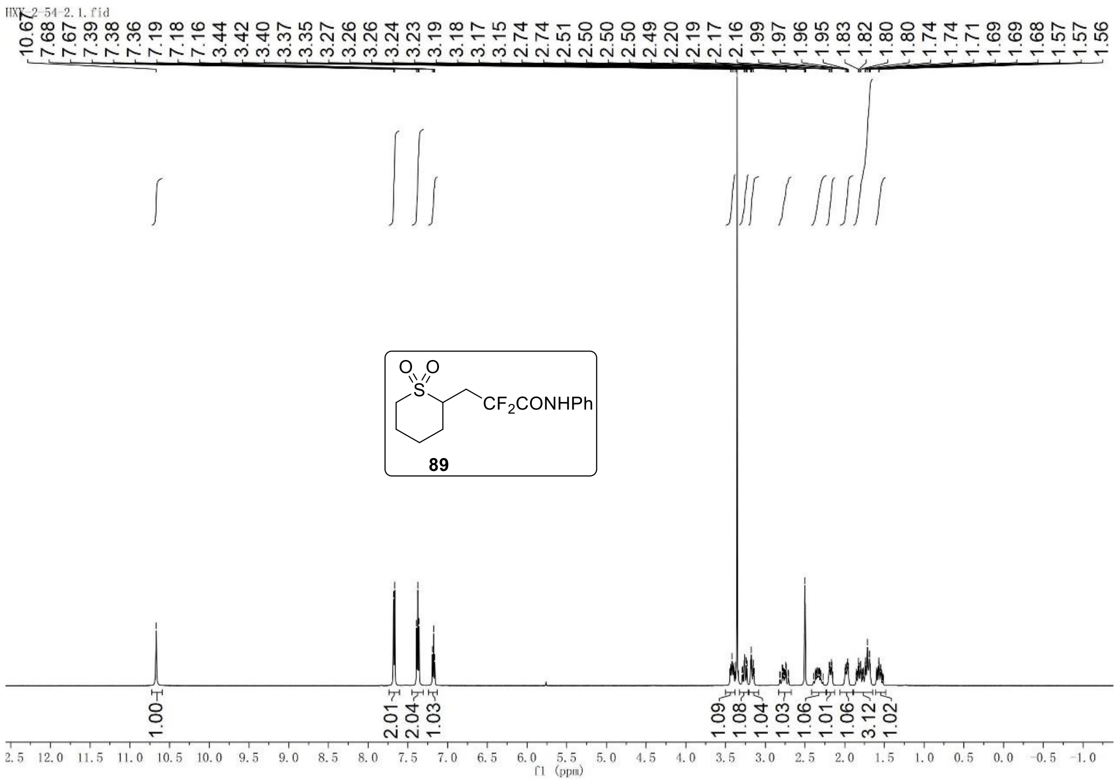

Supplementary Figure 309. ^{1}H NMR (500 MHz, DMSO-d_6) spectra of **89**

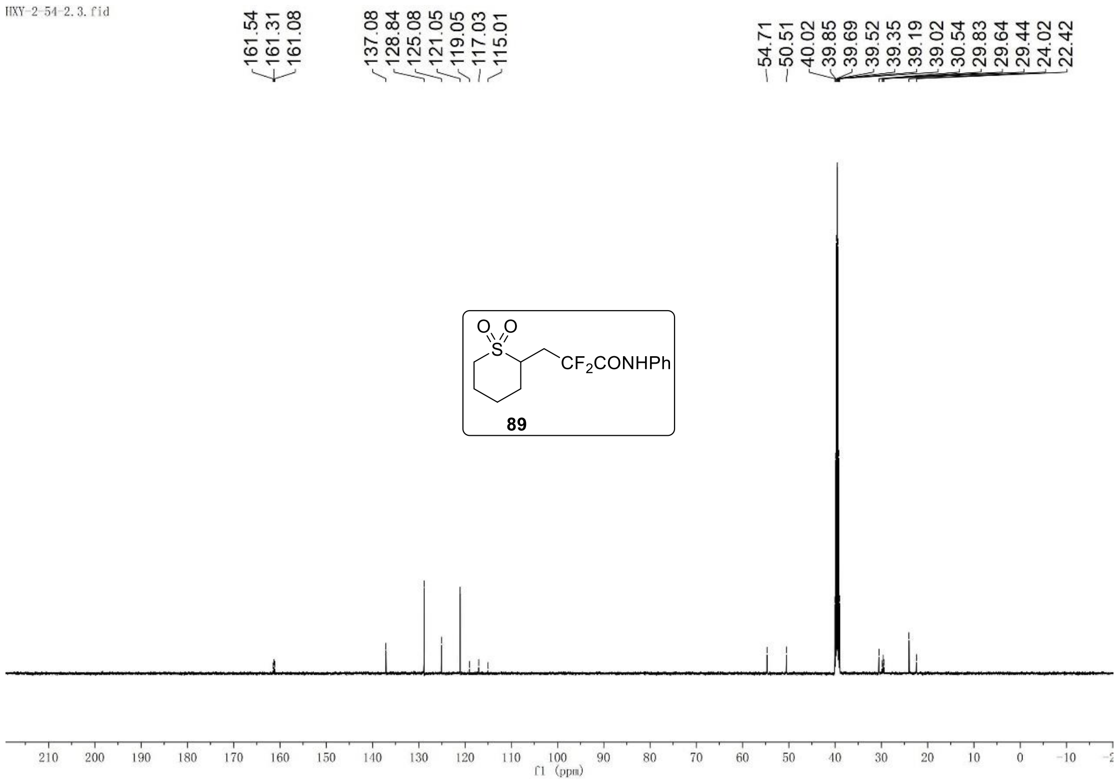

Supplementary Figure 310. ^{13}C NMR (126 MHz, DMSO-d_6) spectra of **89**

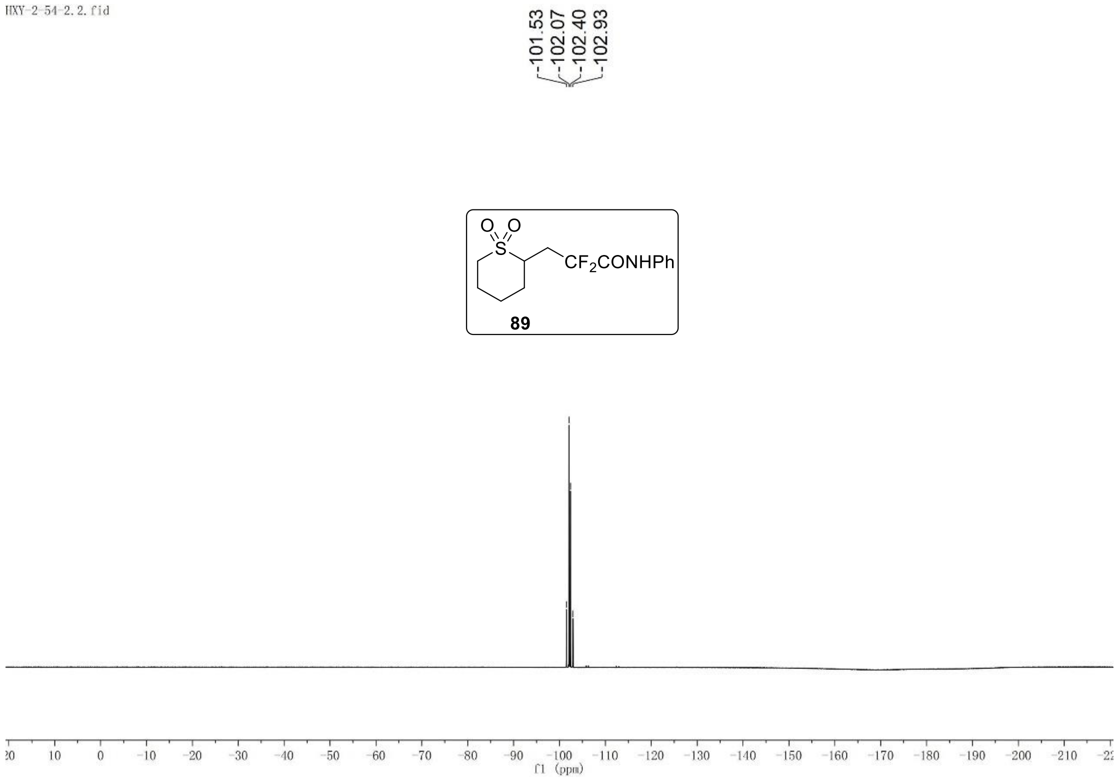

Supplementary Figure 311. ^{19}F NMR (471 MHz, DMSO-d_6) spectra of **89**

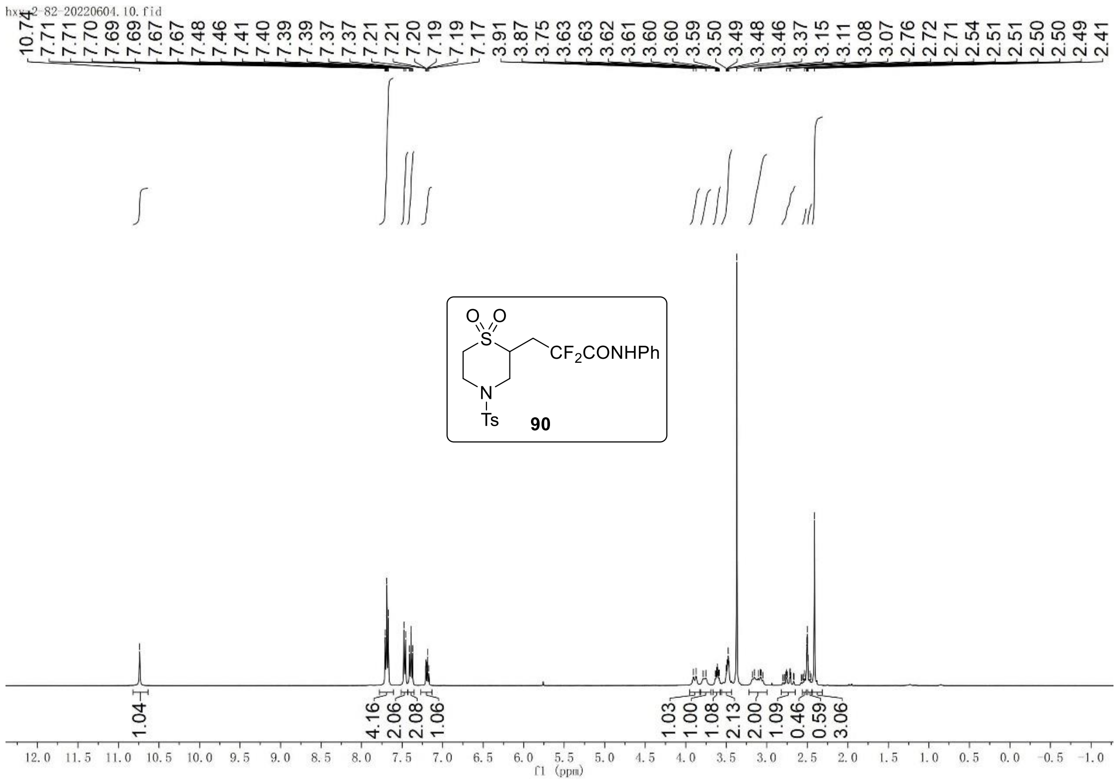

Supplementary Figure 312. ^{1}H NMR (400 MHz, DMSO-d_6) spectra of **90**

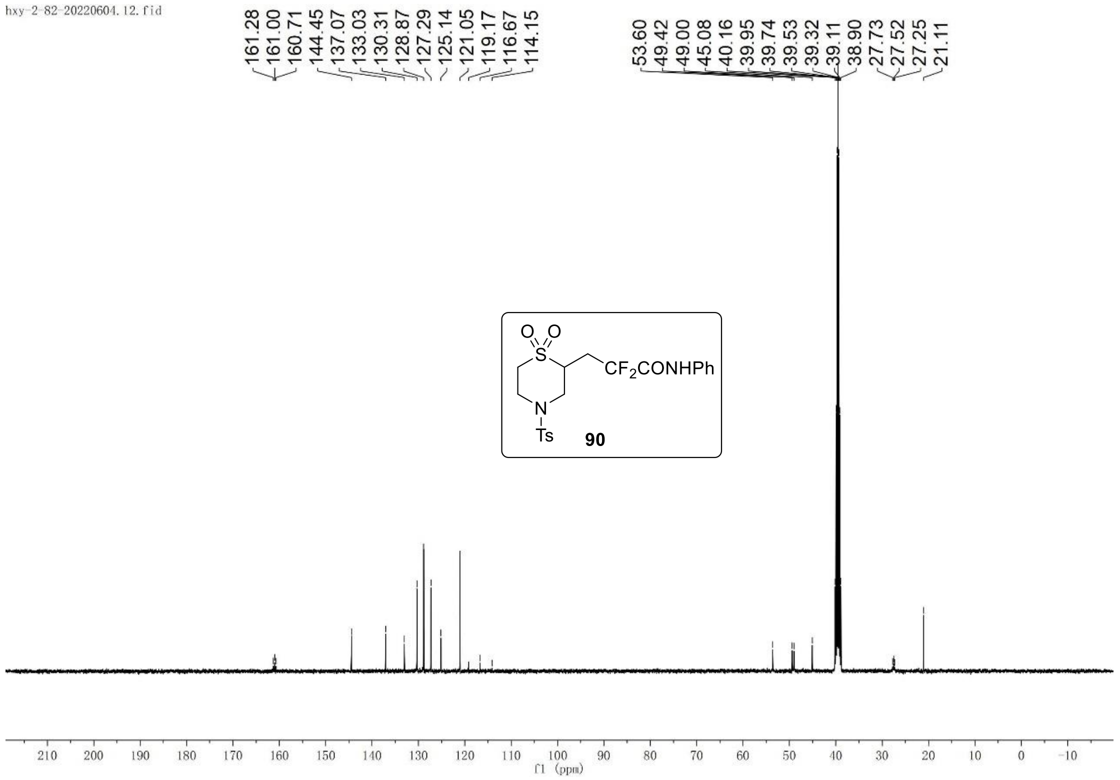

Supplementary Figure 313. ^{13}C NMR (101 MHz, DMSO-d_6) spectra of **90**

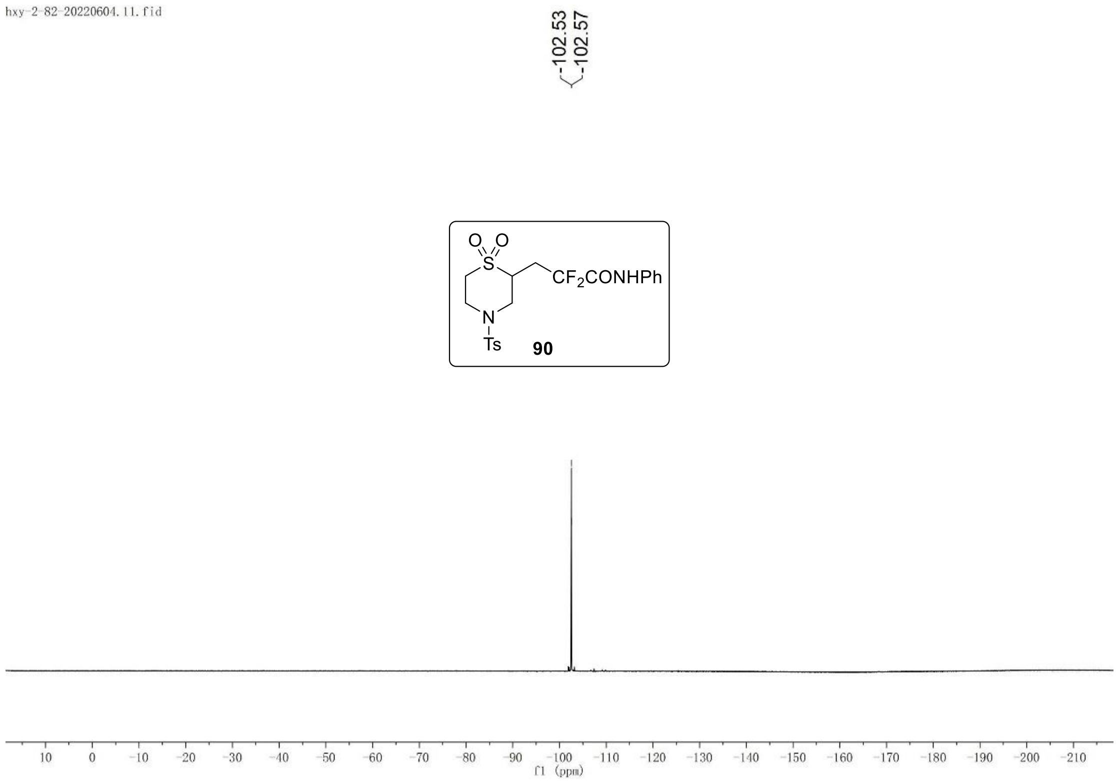

Supplementary Figure 314. ^{19}F NMR (376 MHz, DMSO-d_6) spectra of **90**

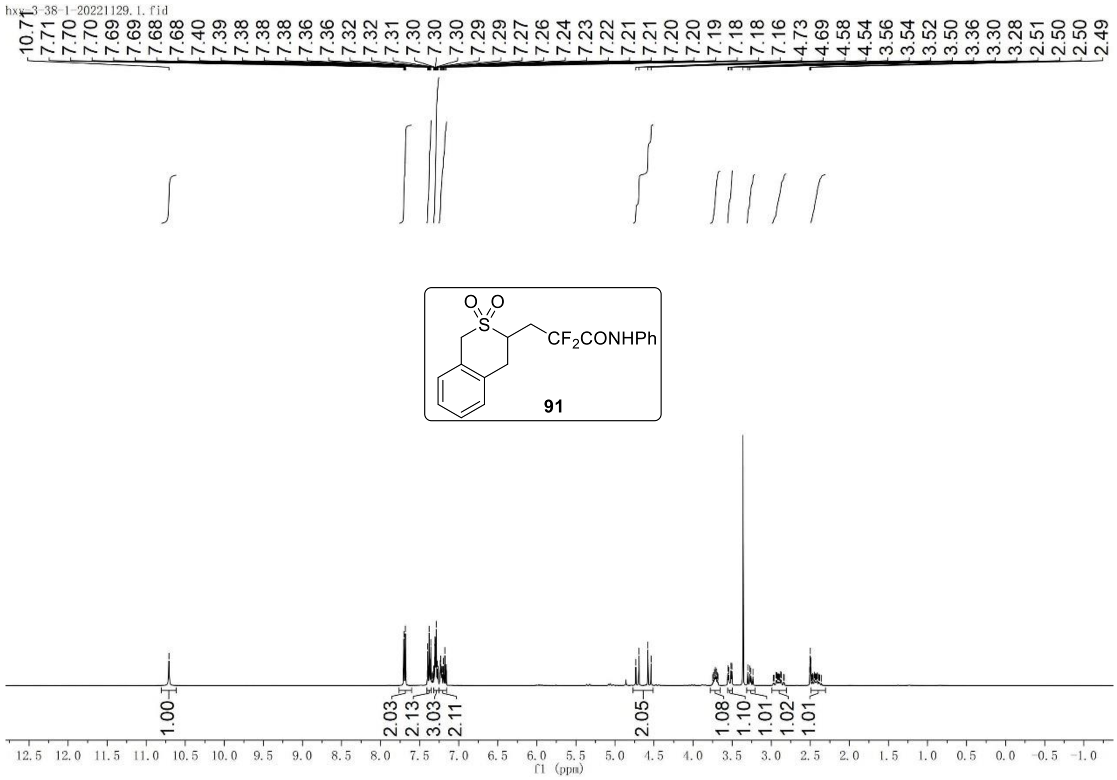

Supplementary Figure 315. ^{1}H NMR (400 MHz, DMSO-d_6) spectra of **91**

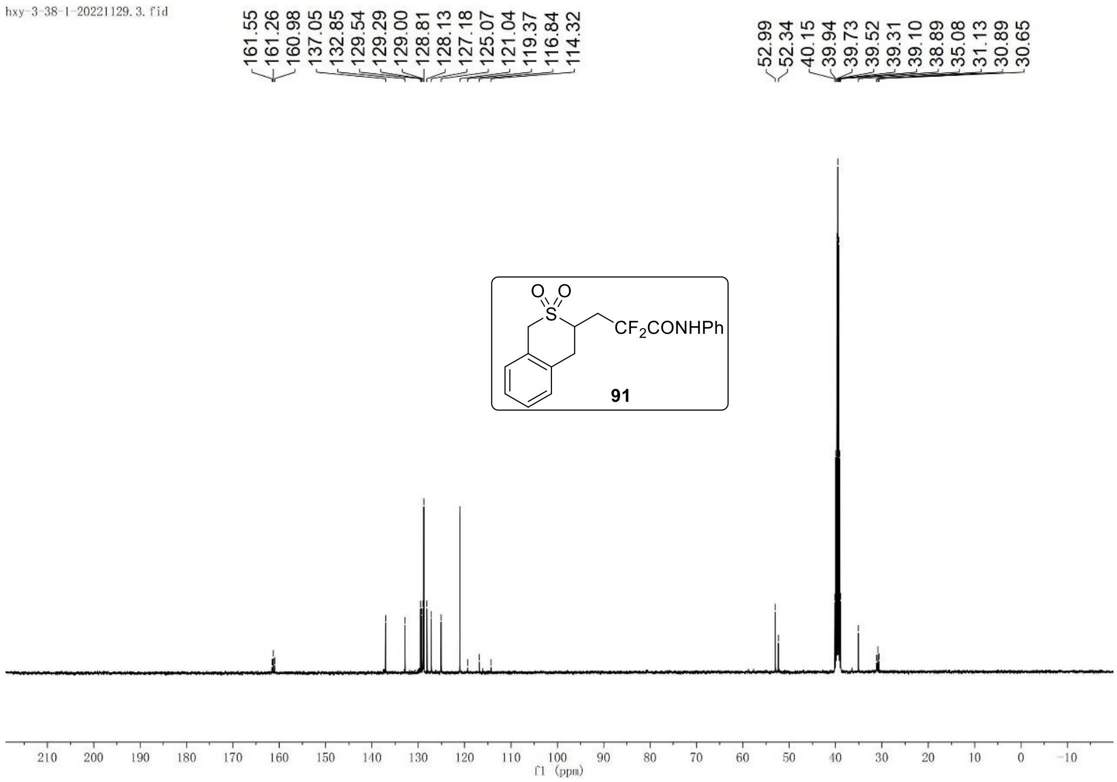

Supplementary Figure 316. ^{13}C NMR (101 MHz, DMSO-d_6) spectra of **91**

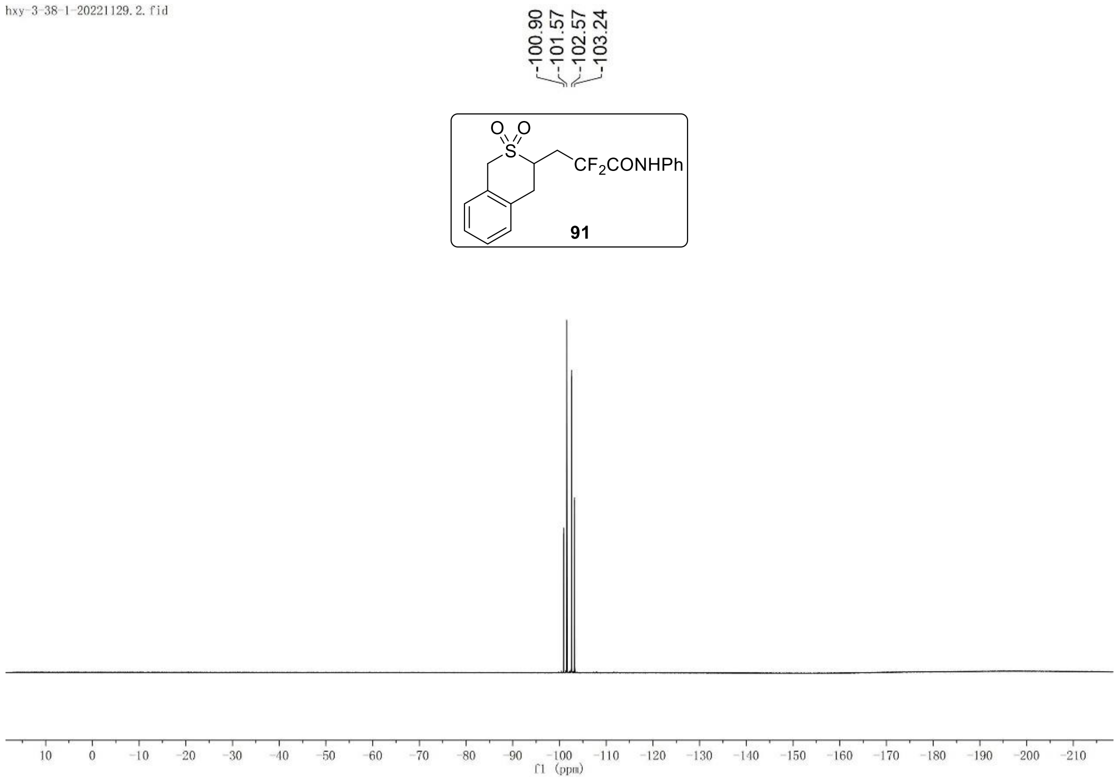

Supplementary Figure 317. ^{19}F NMR (376 MHz, DMSO-d_6) spectra of **91**

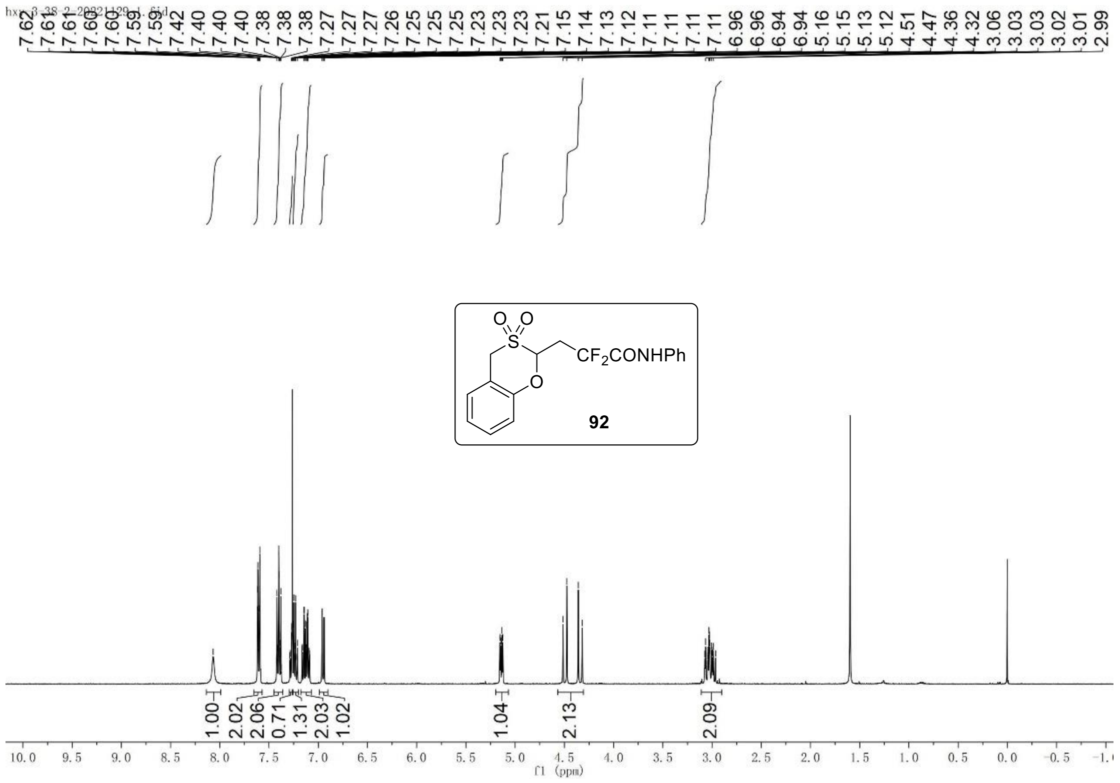

Supplementary Figure 318. ^{1}H NMR (400 MHz, $CDCl_3$) spectra of **92**

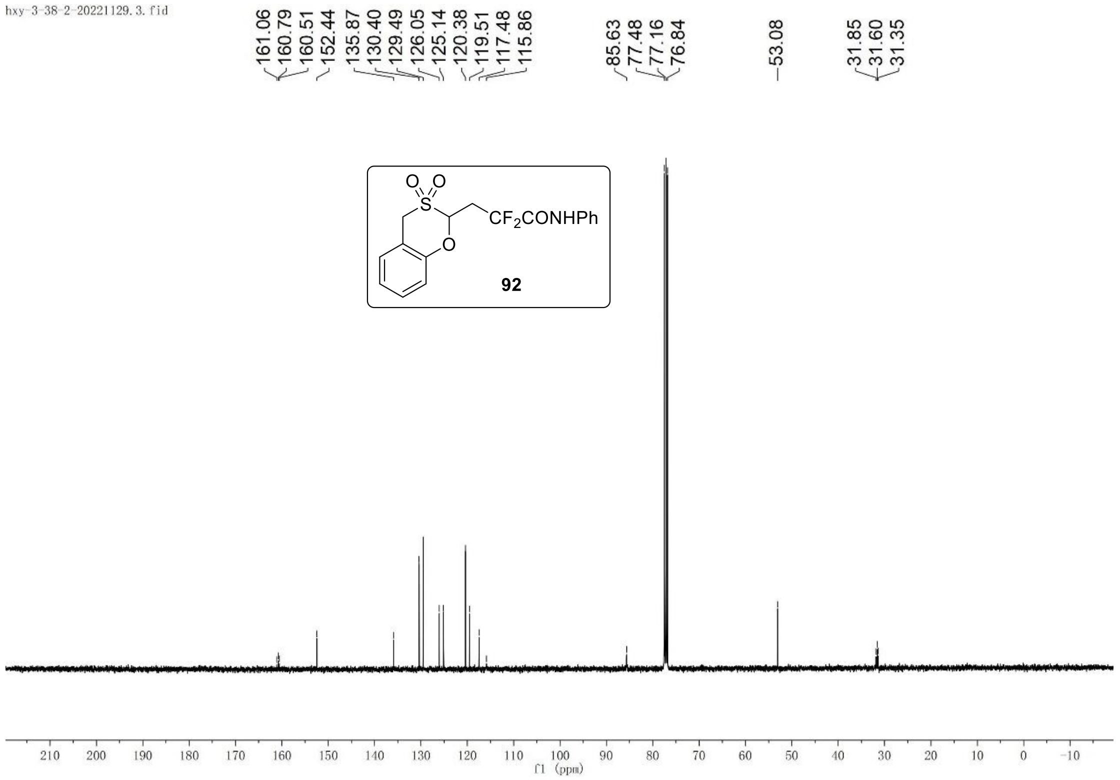

Supplementary Figure 319. ^{13}C NMR (101 MHz, $CDCl_3$) spectra of **92**

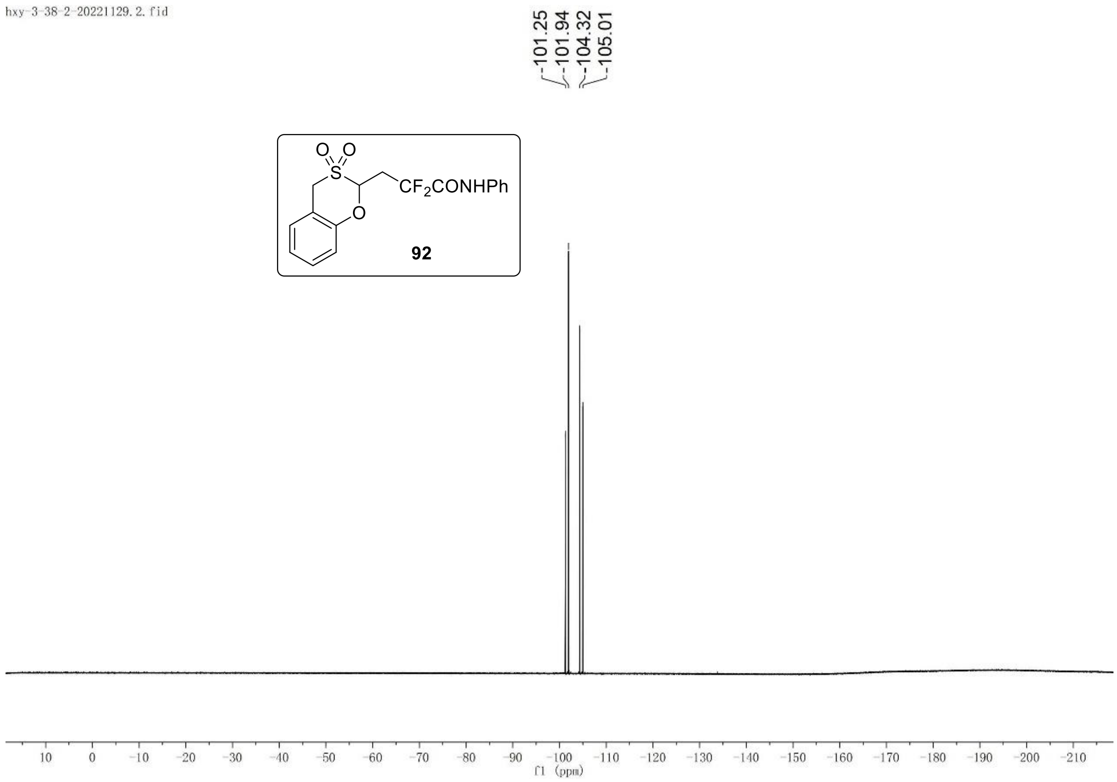

Supplementary Figure 320. ^{19}F NMR (376 MHz, $CDCl_3$) spectra of **92**

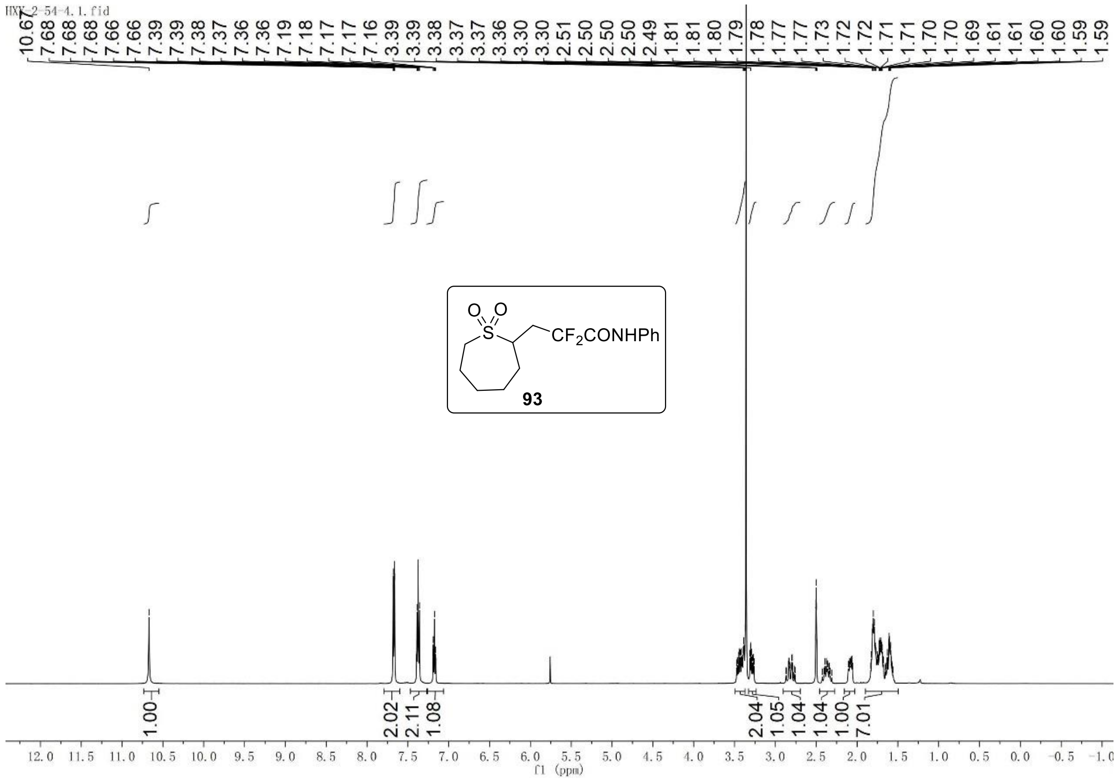

Supplementary Figure 321. ^{1}H NMR (500 MHz, DMSO-d_6) spectra of **93**

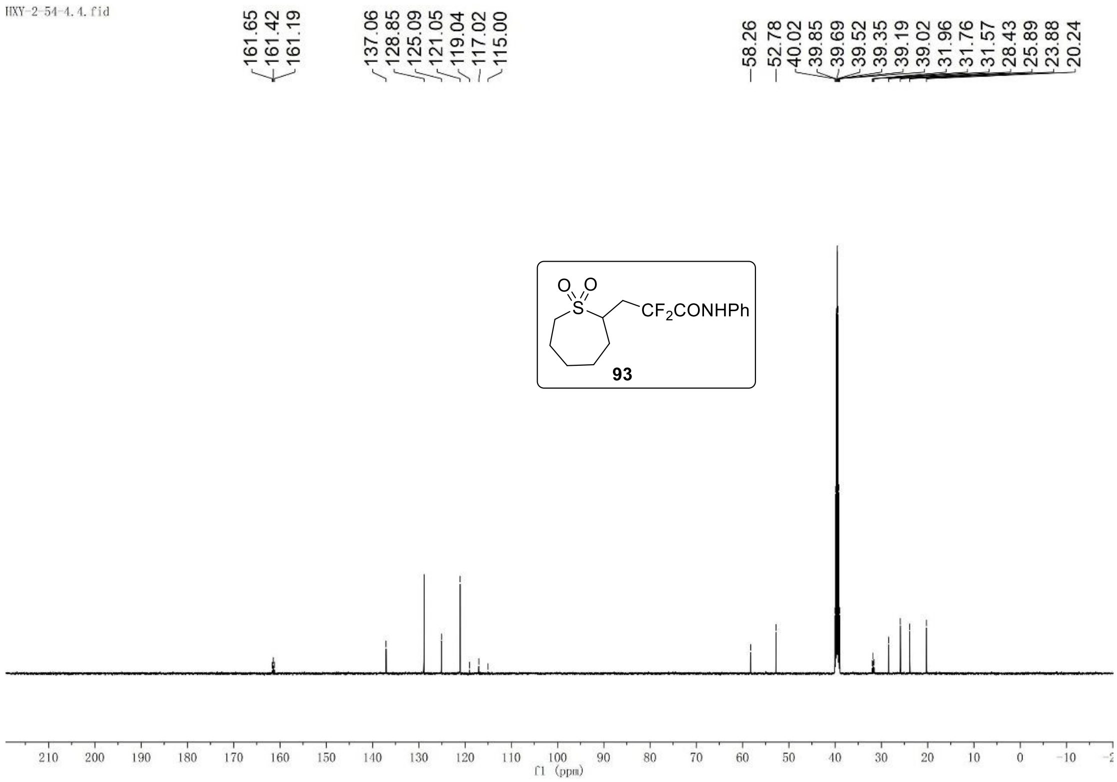

Supplementary Figure 322. ^{13}C NMR (126 MHz, DMSO-d_6) spectra of **93**

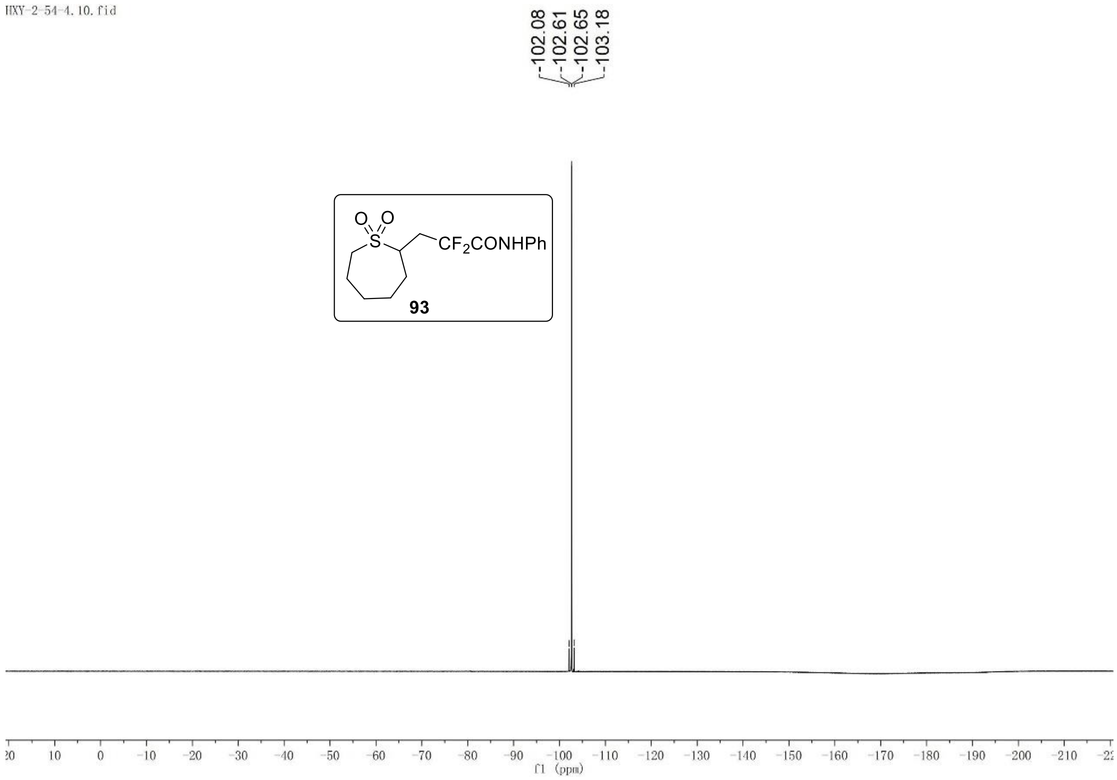

Supplementary Figure 323. ^{19}F NMR (471 MHz, DMSO-d_6) spectra of **93**

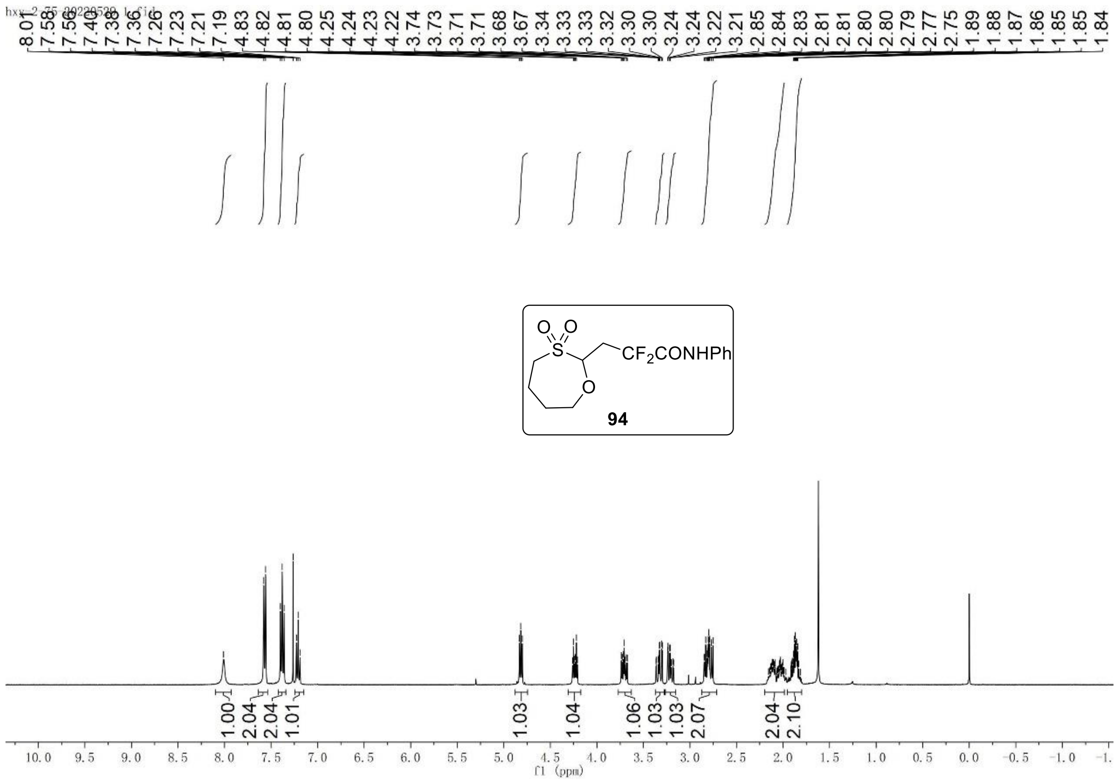

Supplementary Figure 324. ^{1}H NMR (400 MHz, $CDCl_3$) spectra of **94**

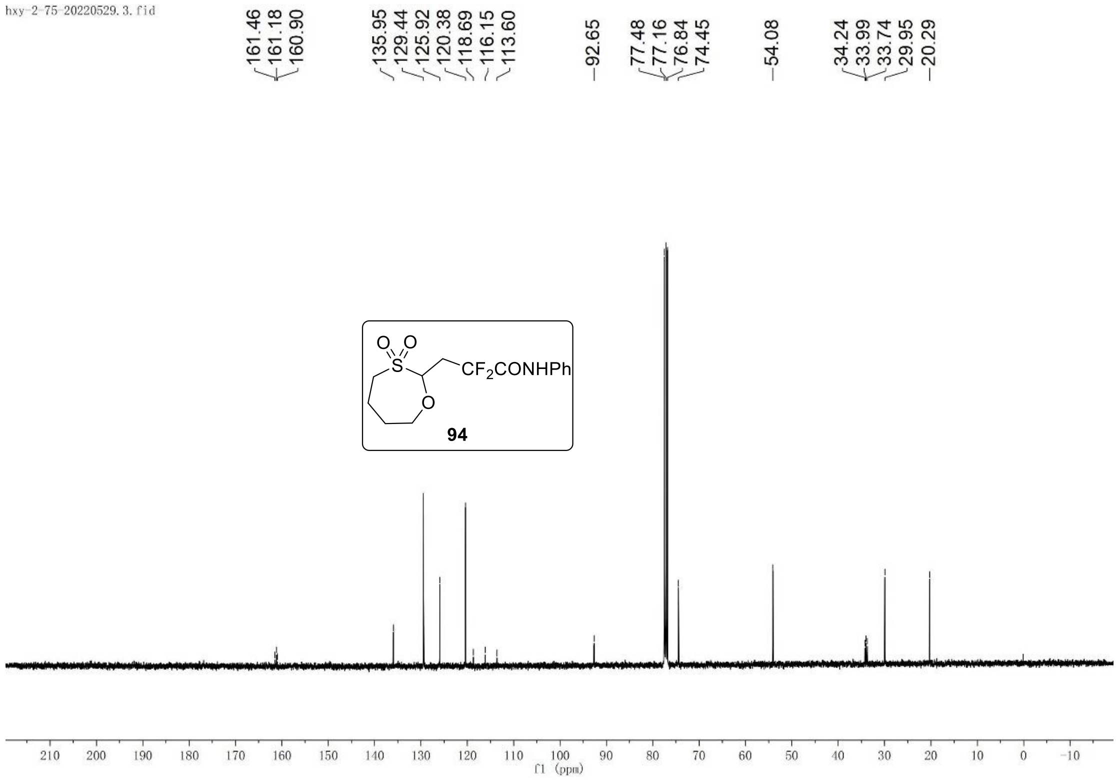

Supplementary Figure 325. ^{13}C NMR (101 MHz, $CDCl_3$) spectra of **94**

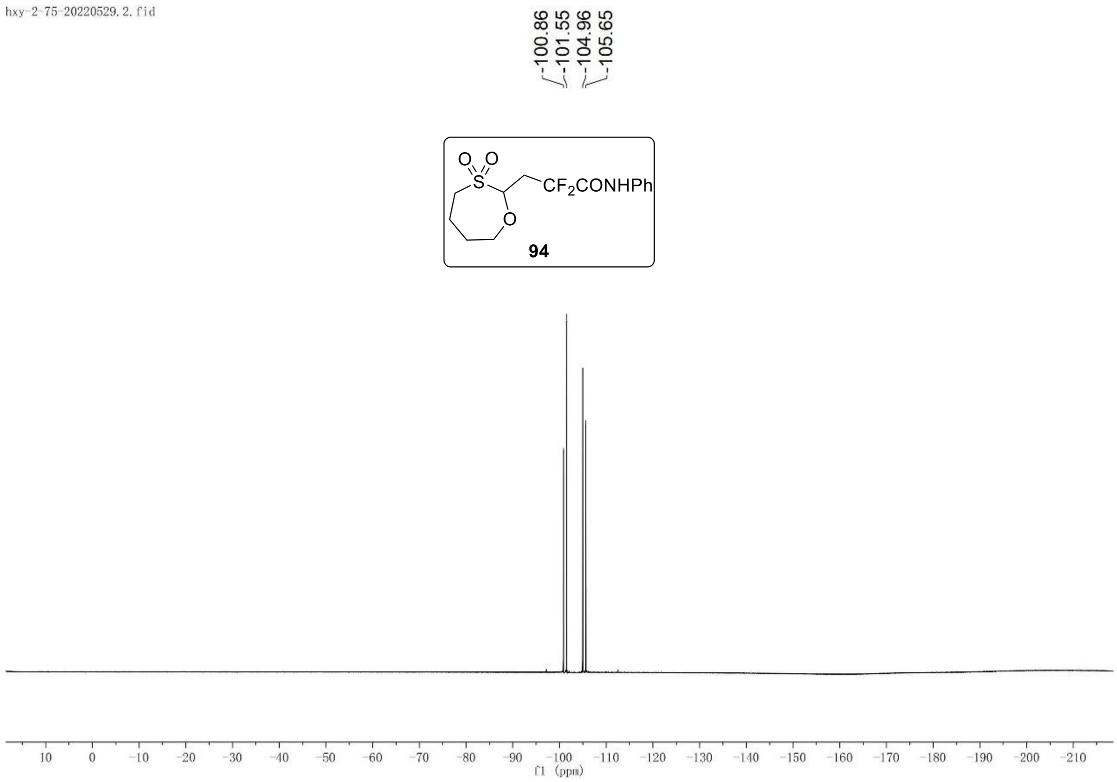

Supplementary Figure 326. ^{19}F NMR (376 MHz, $CDCl_3$) spectra of **94**

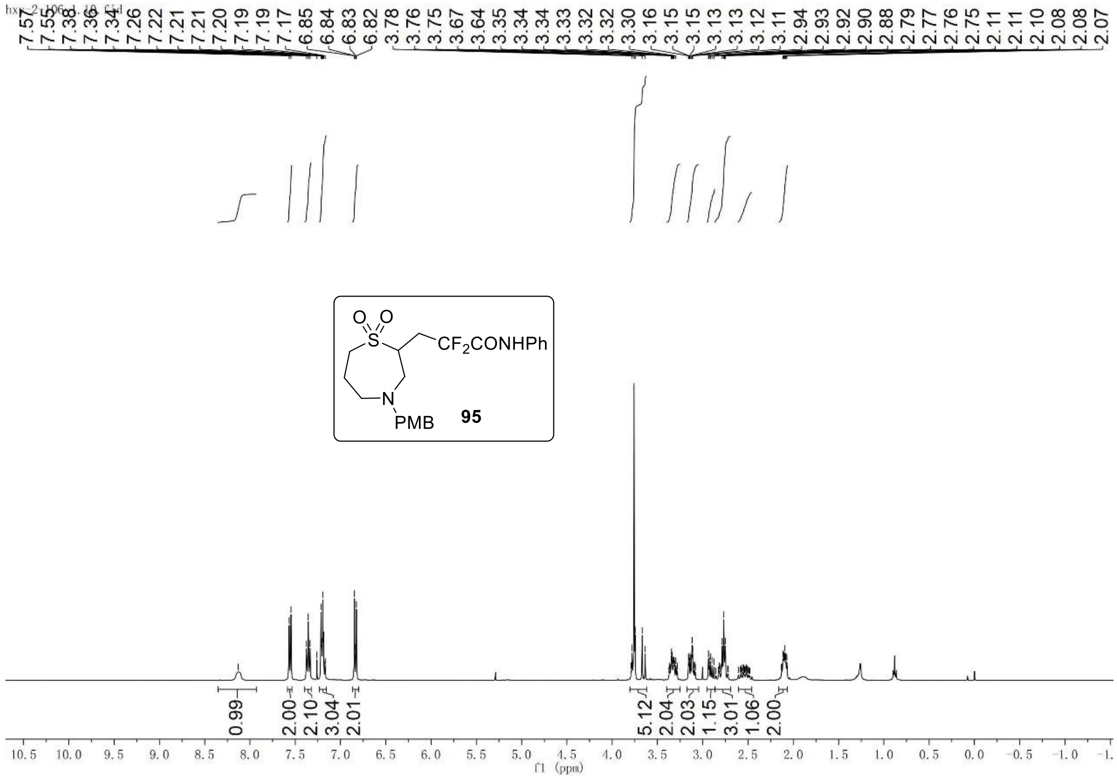

Supplementary Figure 327. ^{1}H NMR (400 MHz, $CDCl_3$) spectra of **95**

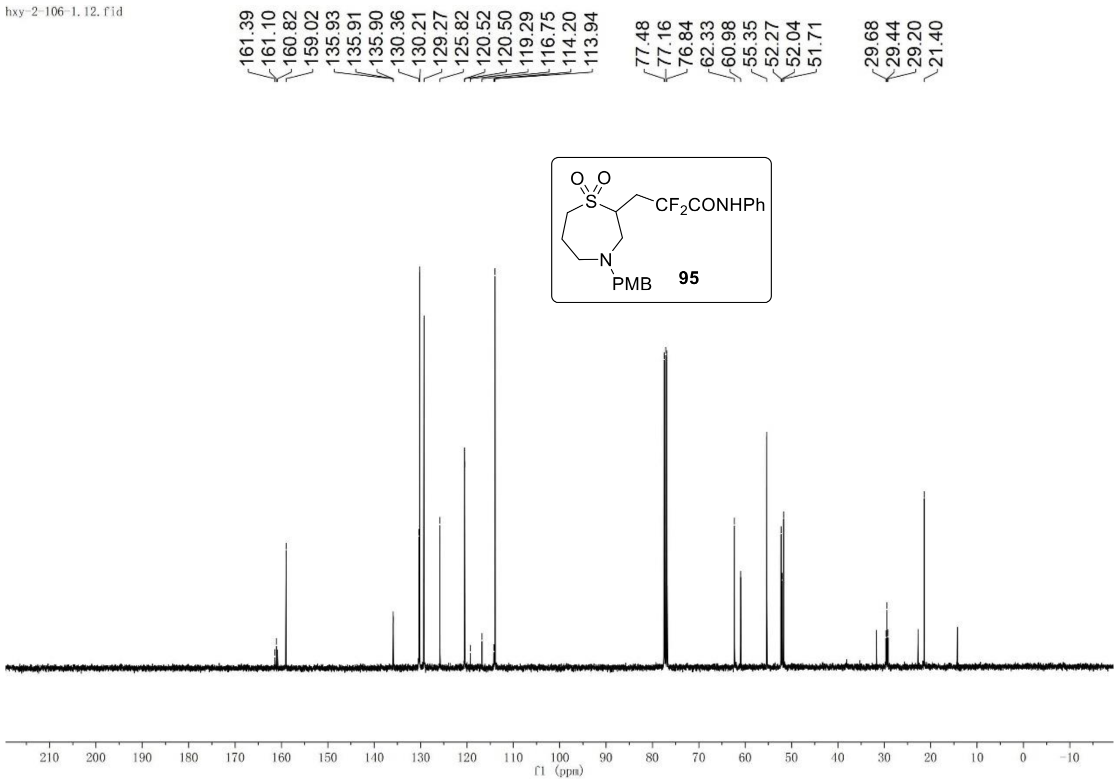

Supplementary Figure 328. ^{13}C NMR (101 MHz, $CDCl_3$) spectra of **95**

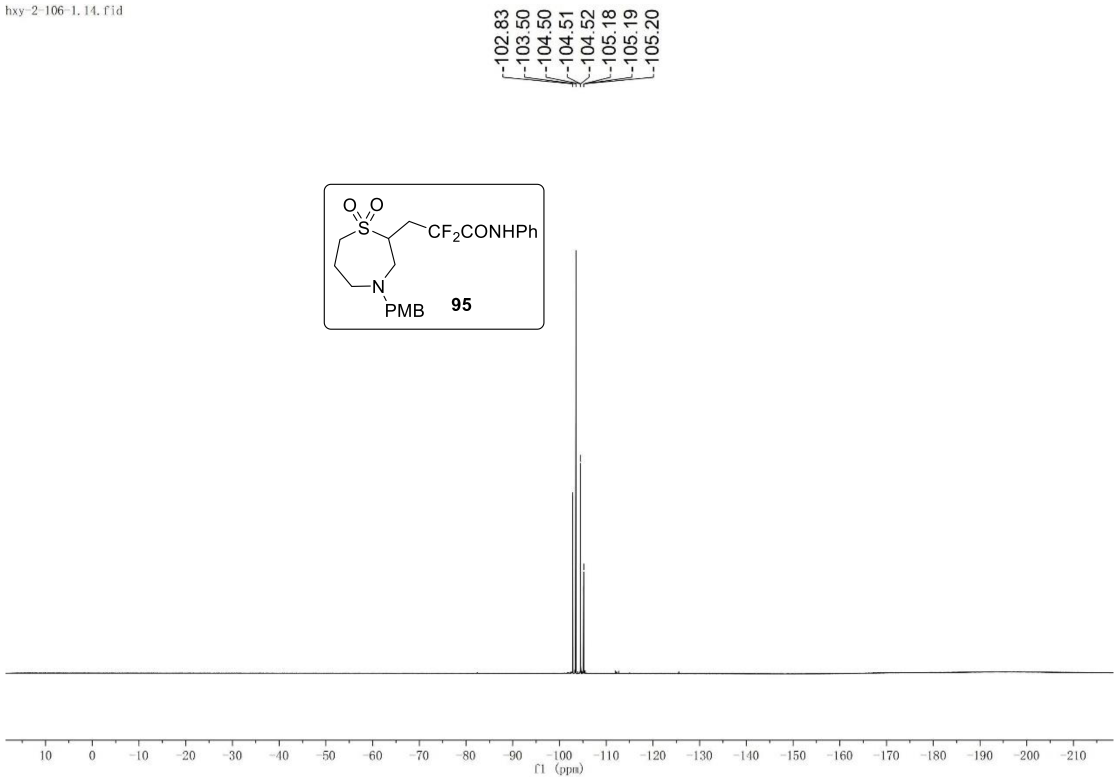

Supplementary Figure 329. ^{19}F NMR (376 MHz, $CDCl_3$) spectra of **95**

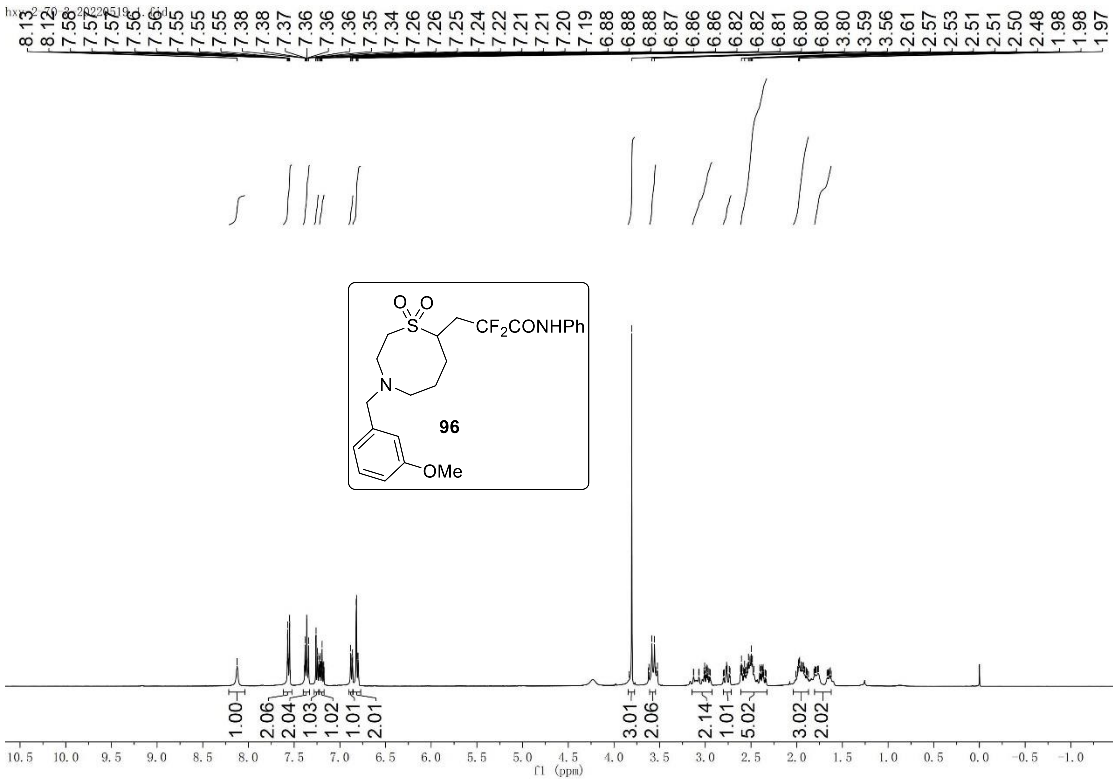

Supplementary Figure 330. ^{1}H NMR (400 MHz, $CDCl_3$) spectra of **96**

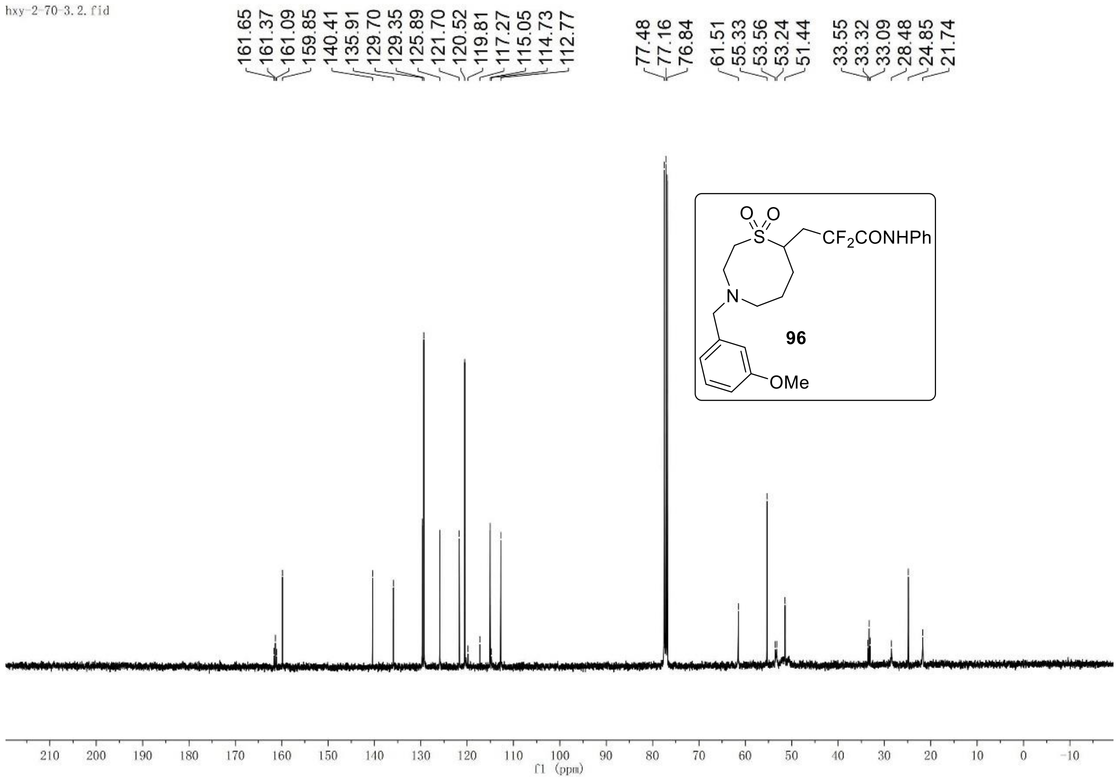

Supplementary Figure 331. ^{13}C NMR (101 MHz, $CDCl_3$) spectra of **96**

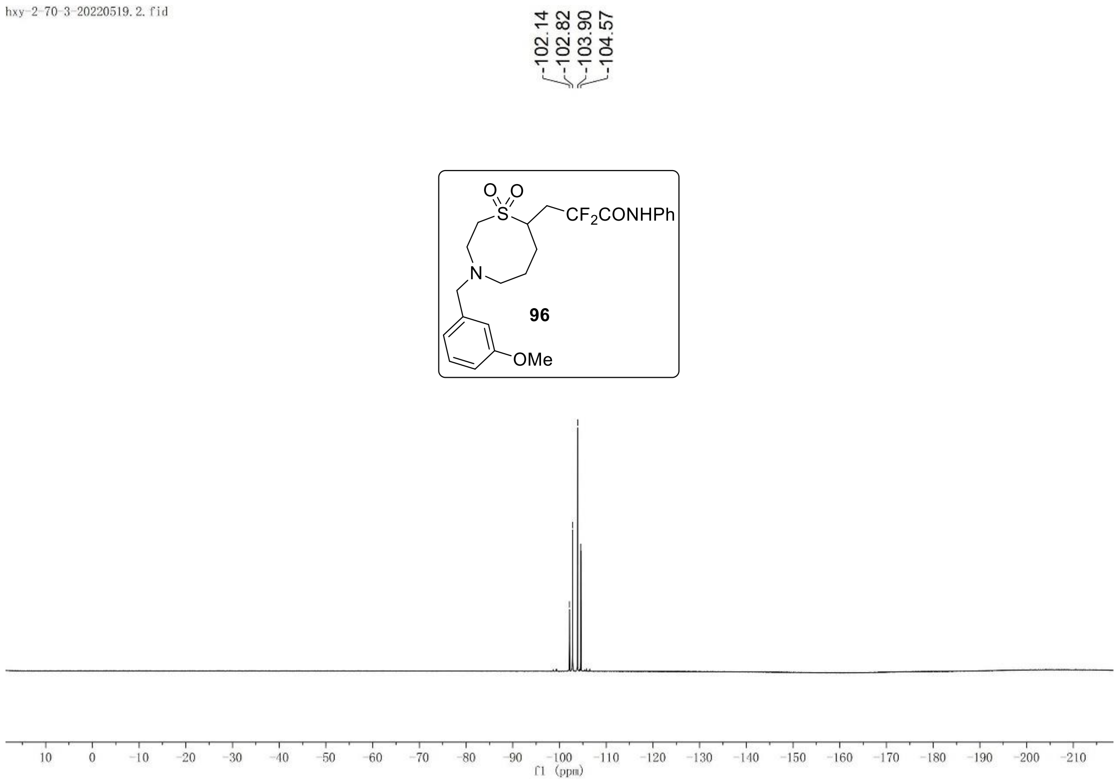

Supplementary Figure 332. ^{19}F NMR (376 MHz, $CDCl_3$) spectra of **96**

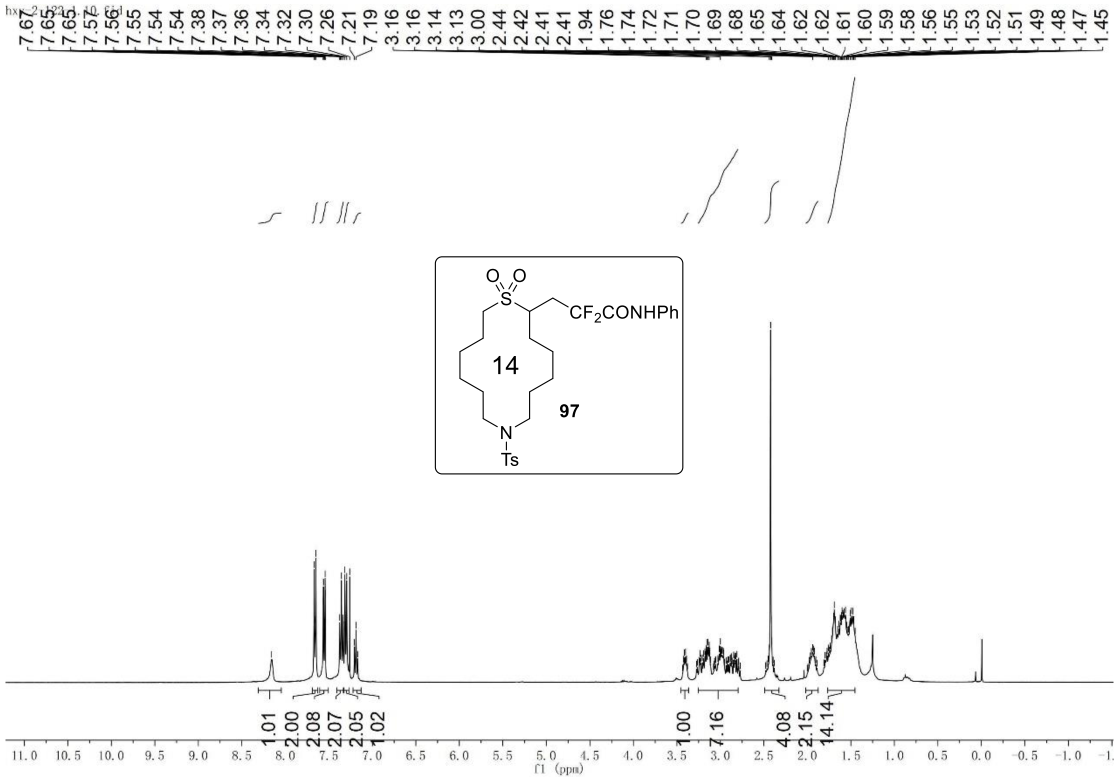

Supplementary Figure 333. ^{1}H NMR (400 MHz, $CDCl_3$) spectra of **97**

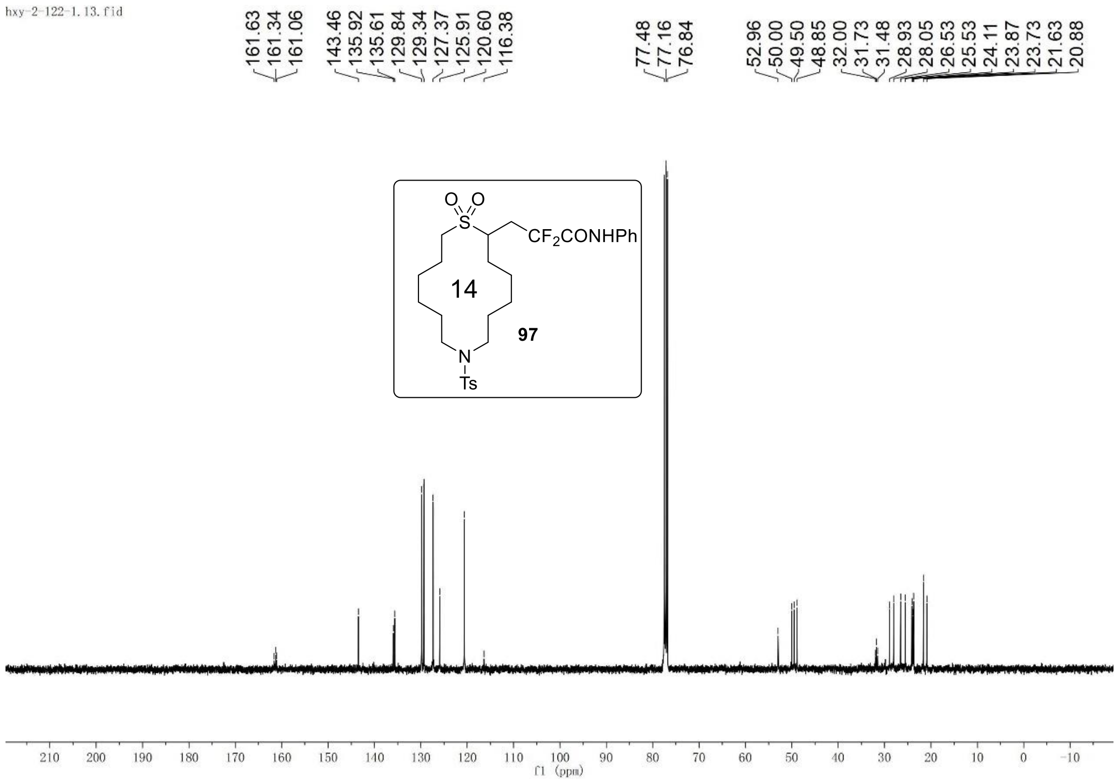

Supplementary Figure 334. ^{13}C NMR (101 MHz, $CDCl_3$) spectra of **97**

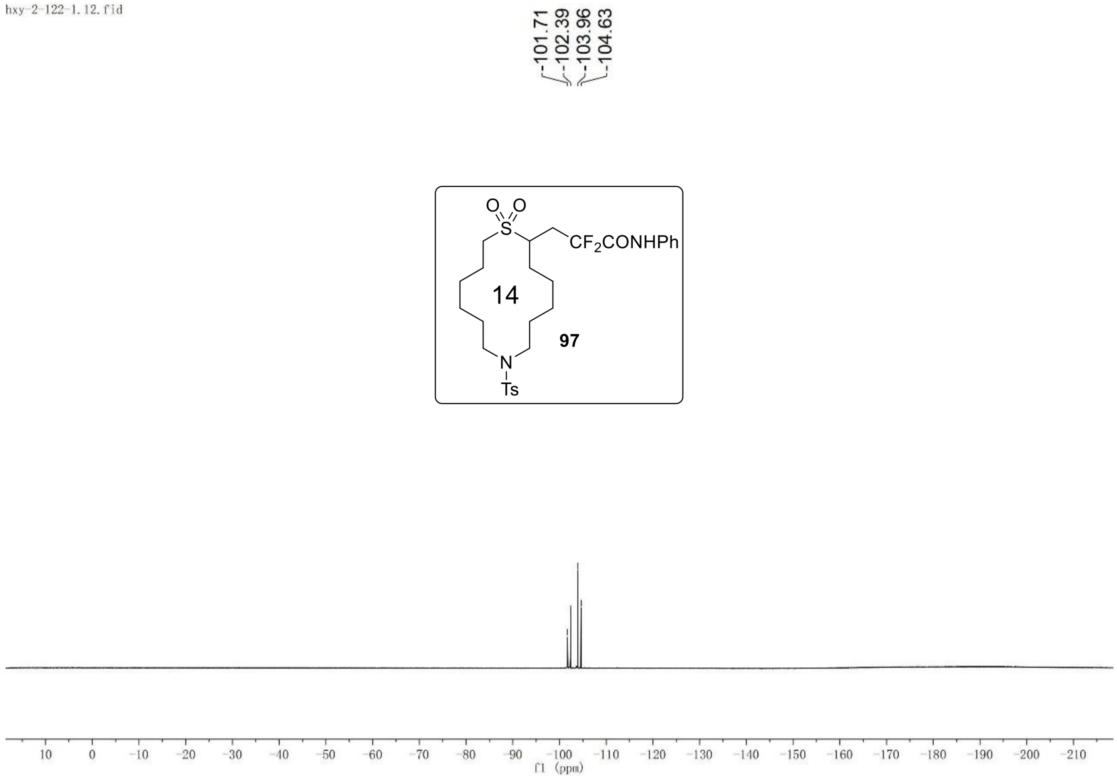

Supplementary Figure 335. ^{19}F NMR (376 MHz, $CDCl_3$) spectra of **97**

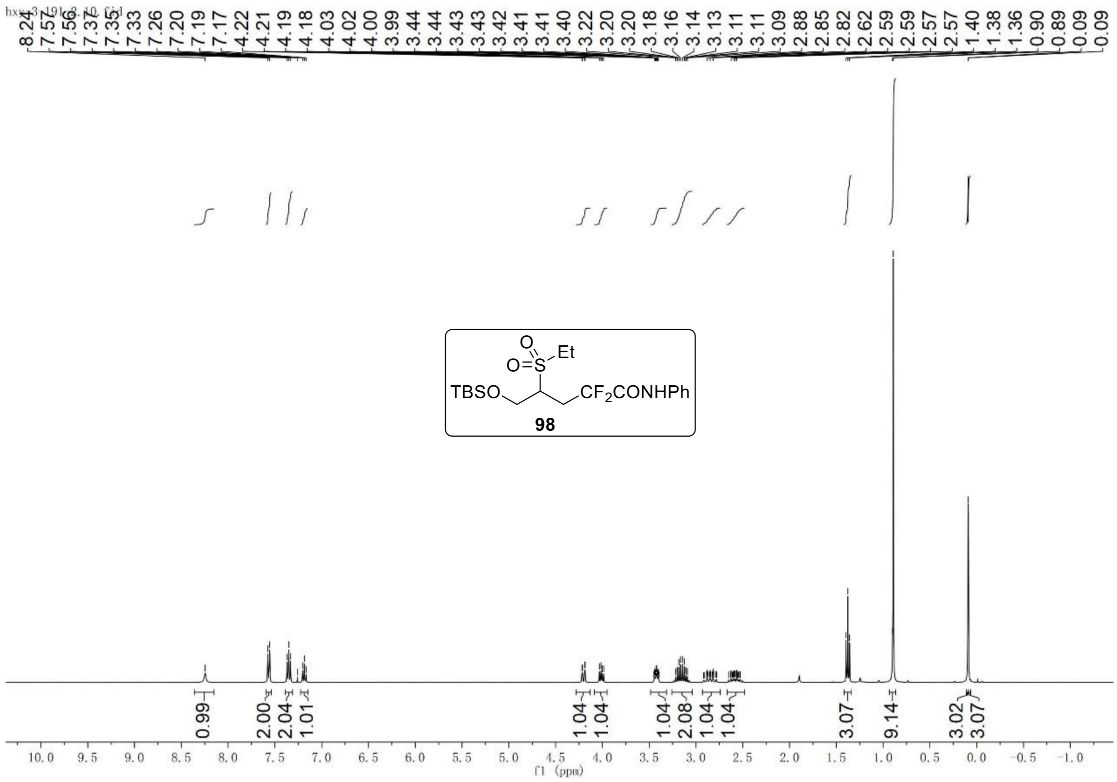

Supplementary Figure 336. ^{1}H NMR (400 MHz, $CDCl_3$) spectra of **98**

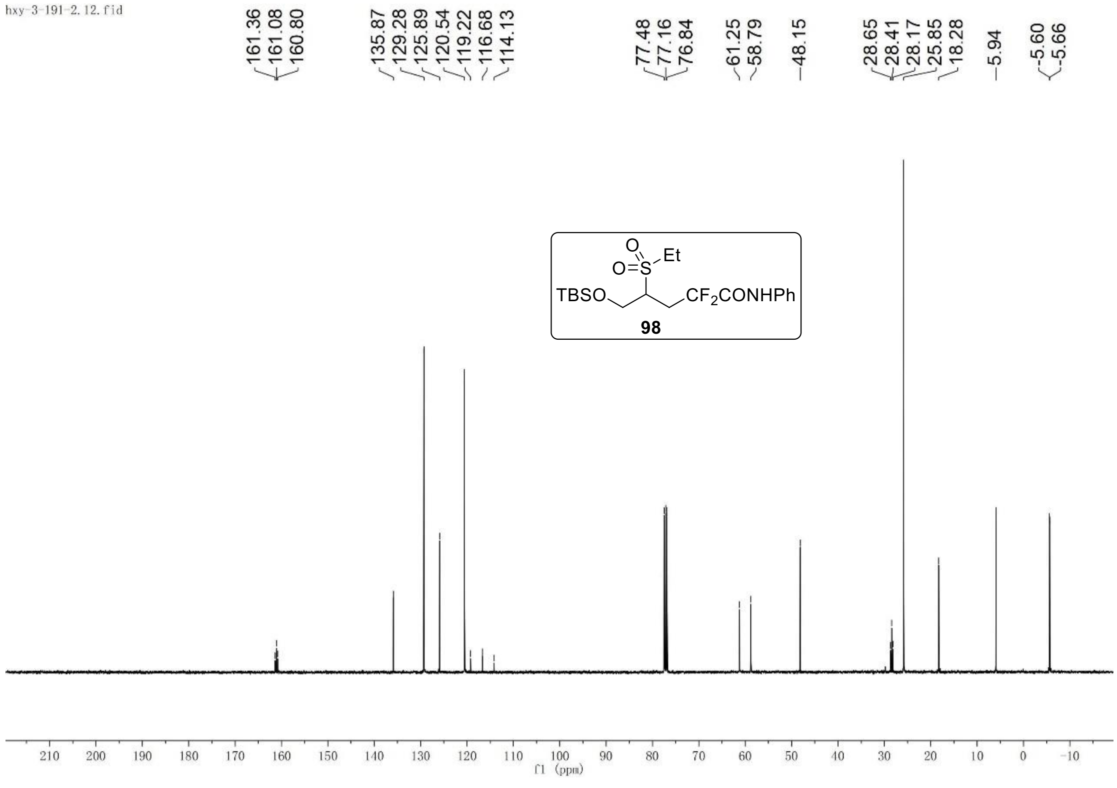

Supplementary Figure 337. ^{13}C NMR (101 MHz, $CDCl_3$) spectra of **98**

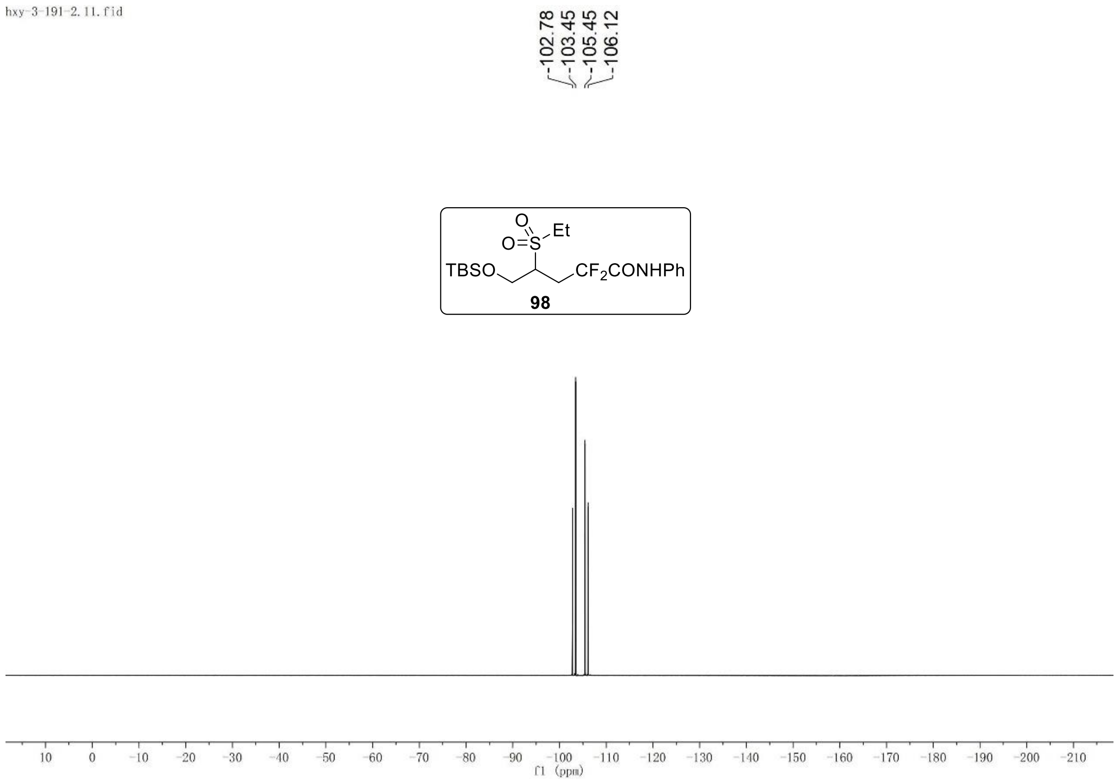

Supplementary Figure 338. ^{19}F NMR (376 MHz, $CDCl_3$) spectra of **98**

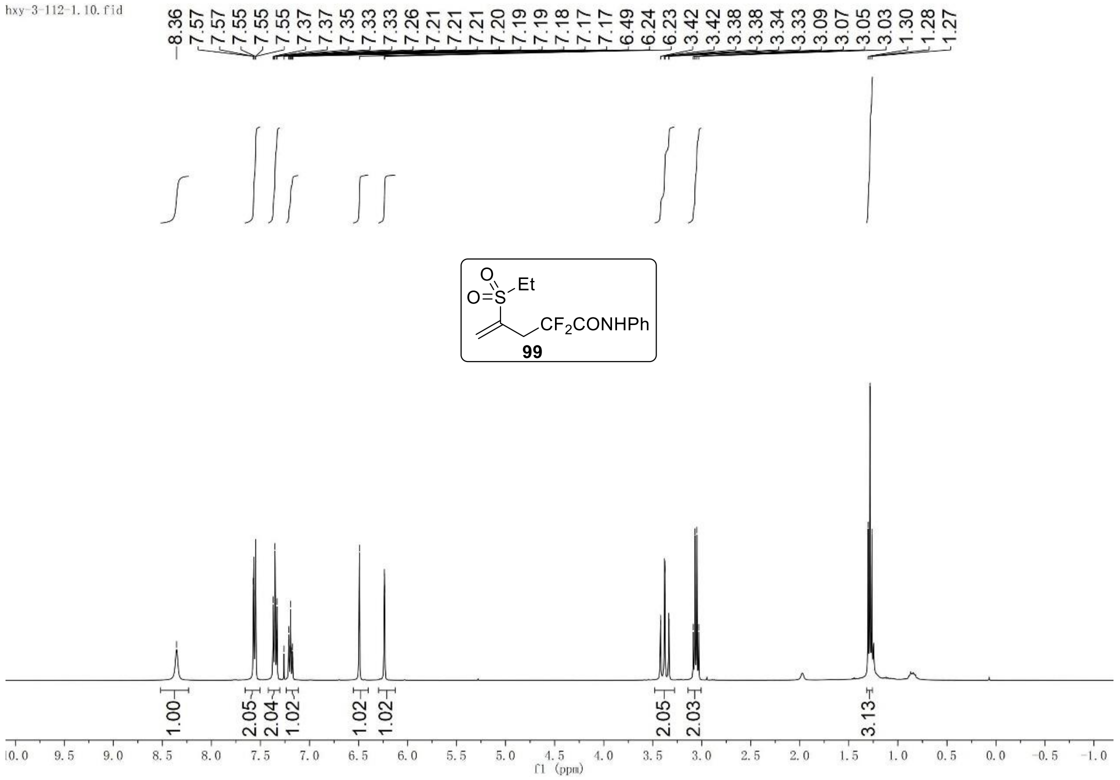

Supplementary Figure 339. ^{1}H NMR (400 MHz, $CDCl_3$) spectra of **99**

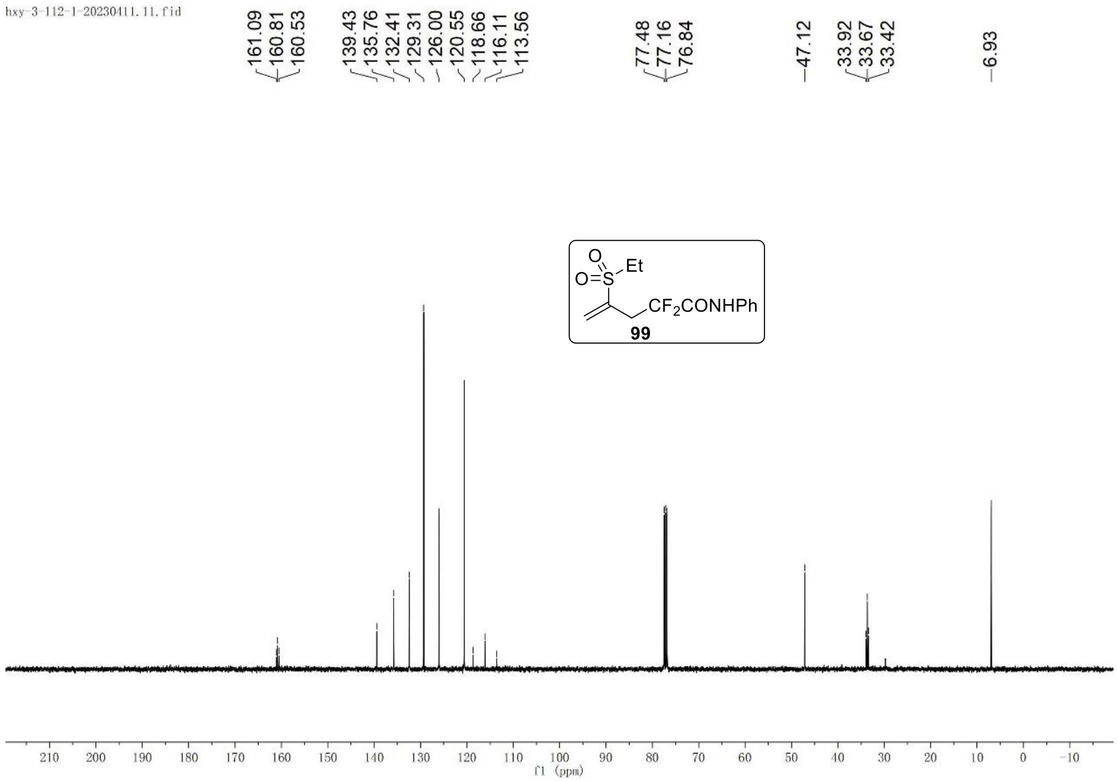

Supplementary Figure 340. ^{13}C NMR (101 MHz, $CDCl_3$) spectra of **99**

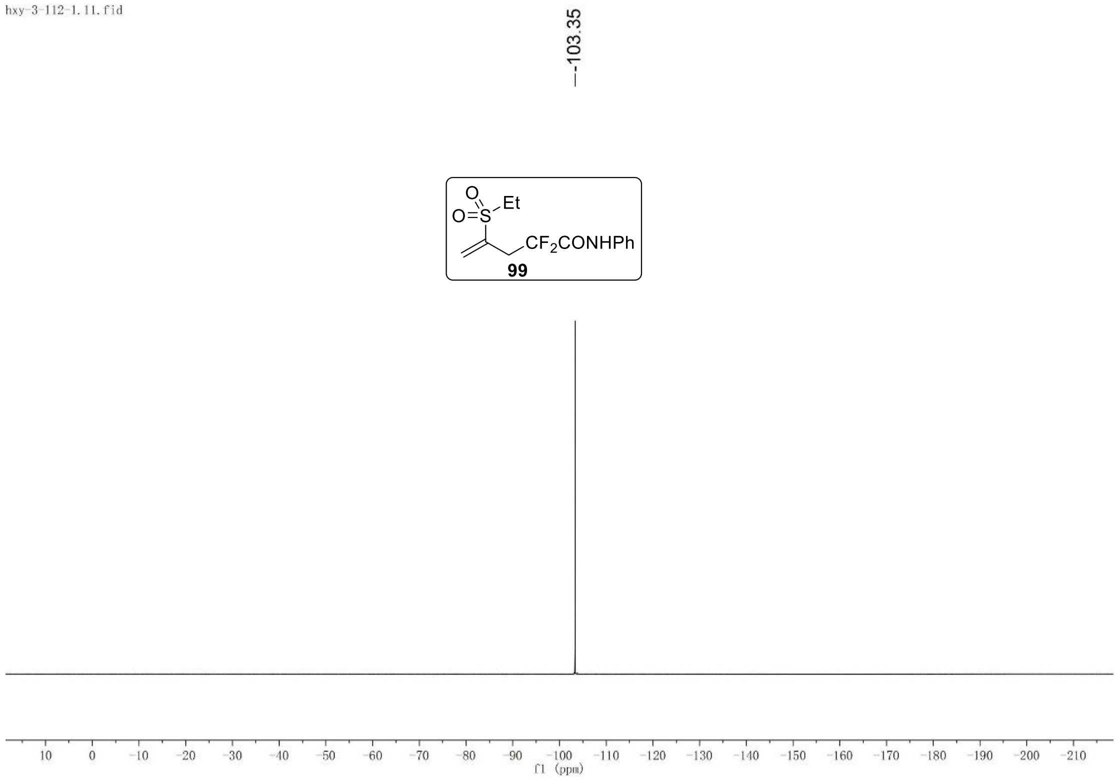

Supplementary Figure 341. ^{19}F NMR (376 MHz, $CDCl_3$) spectra of **99**

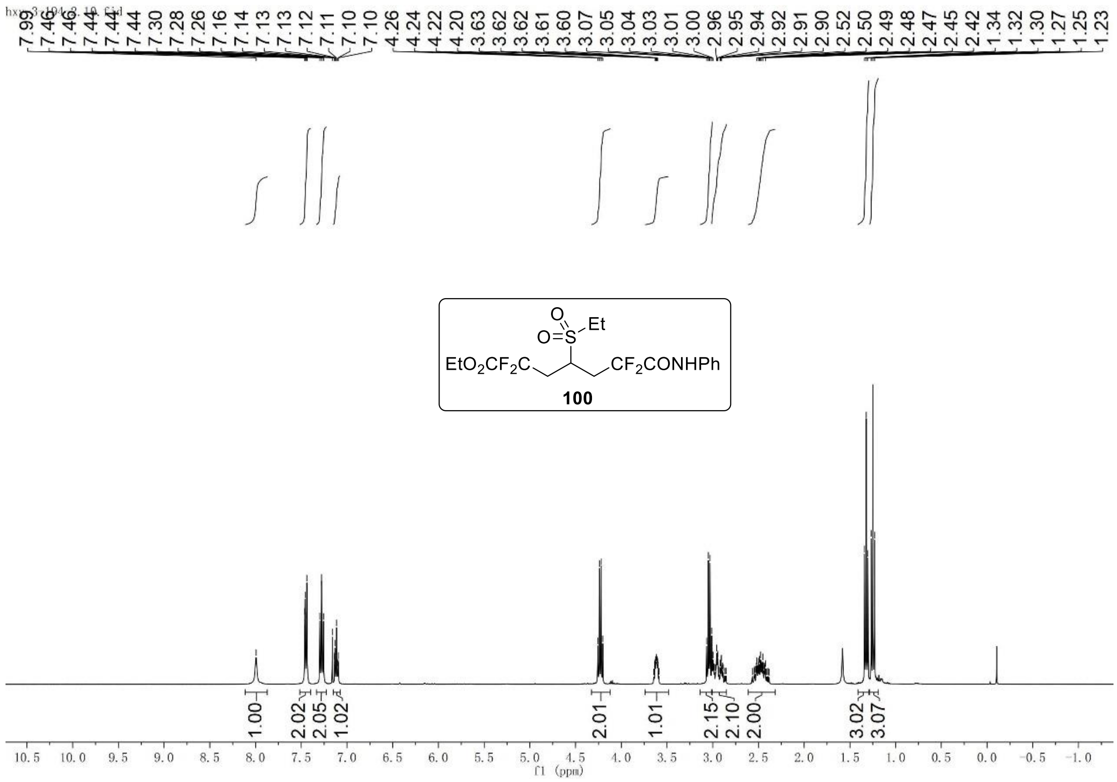

Supplementary Figure 342. ^{1}H NMR (400 MHz, $CDCl_3$) spectra of **100**

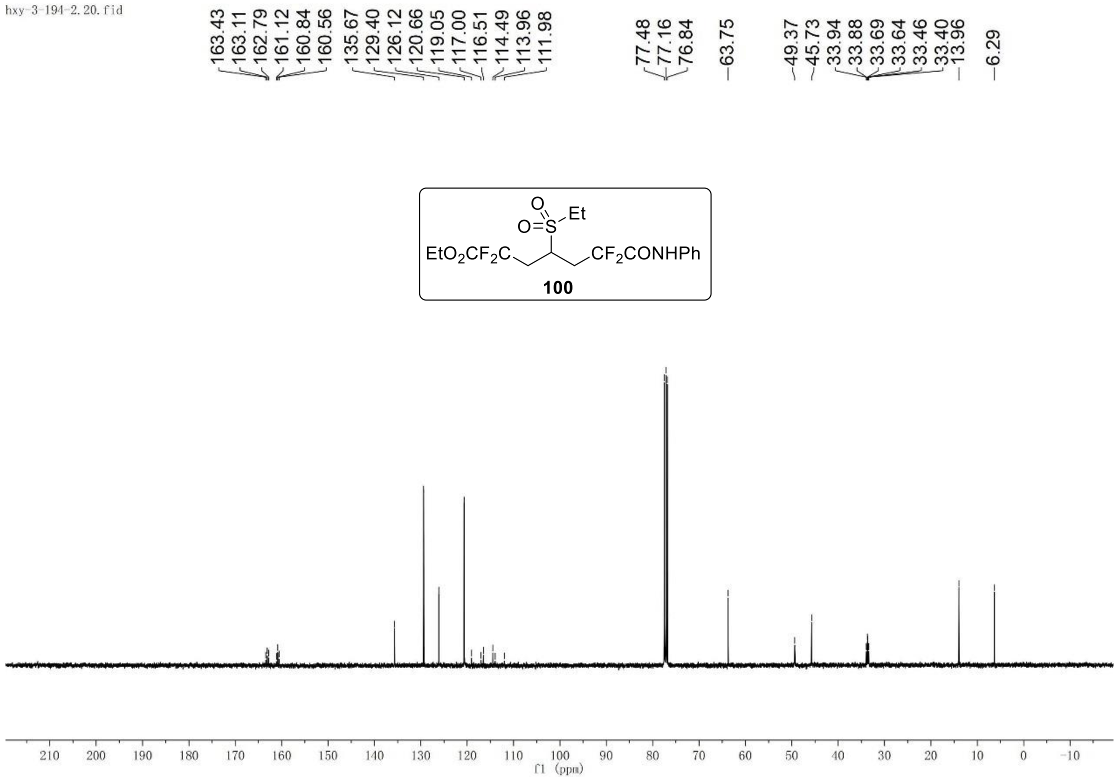

Supplementary Figure 343. ^{13}C NMR (101 MHz, $CDCl_3$) spectra of **100**

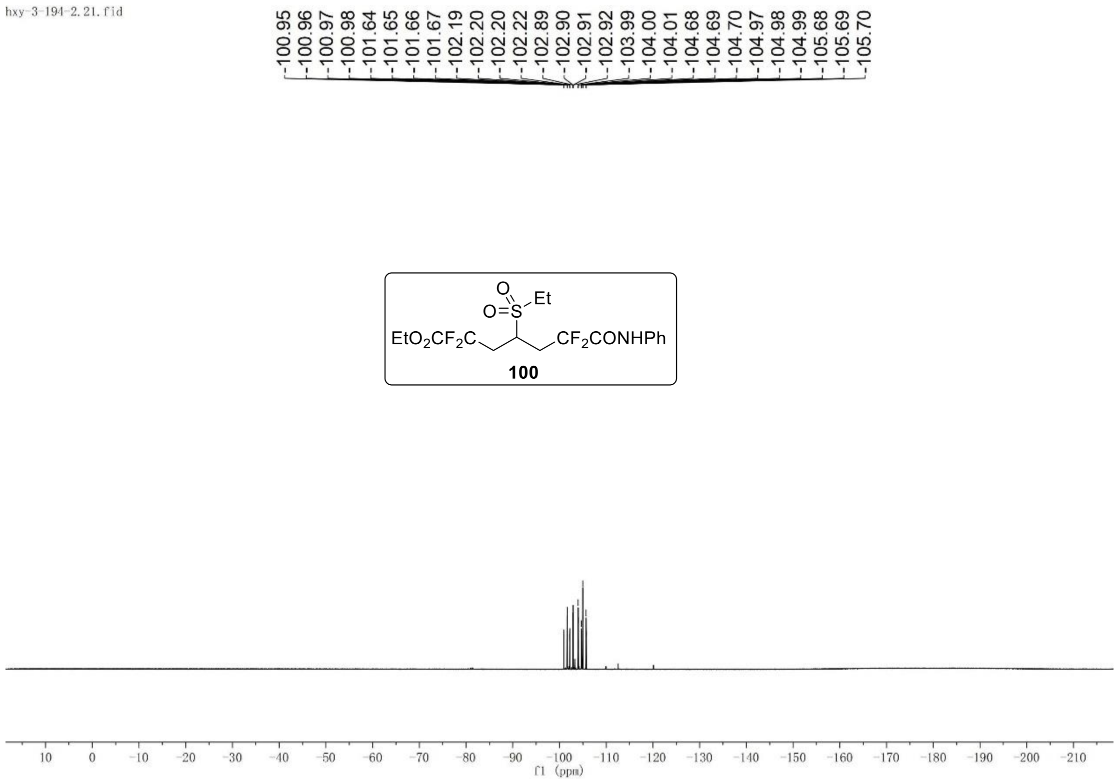

Supplementary Figure 344. ^{19}F NMR (376 MHz, $CDCl_3$) spectra of **100**

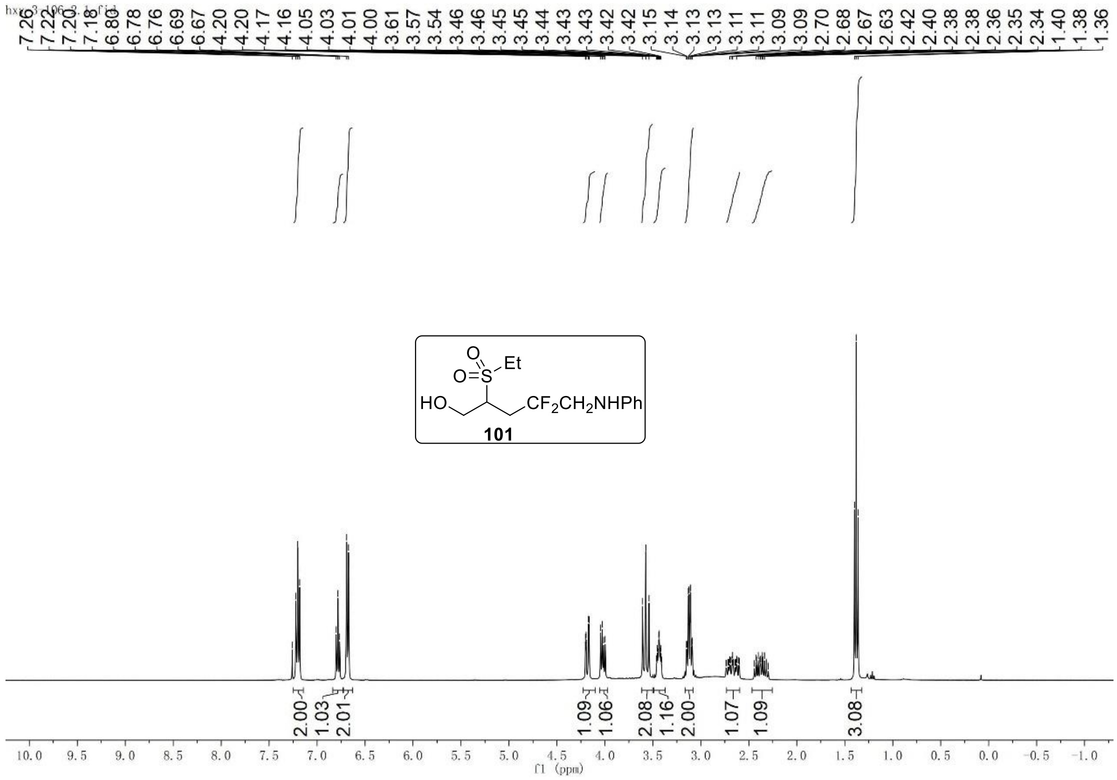

Supplementary Figure 345. ^{1}H NMR (400 MHz, $CDCl_3$) spectra of **101**

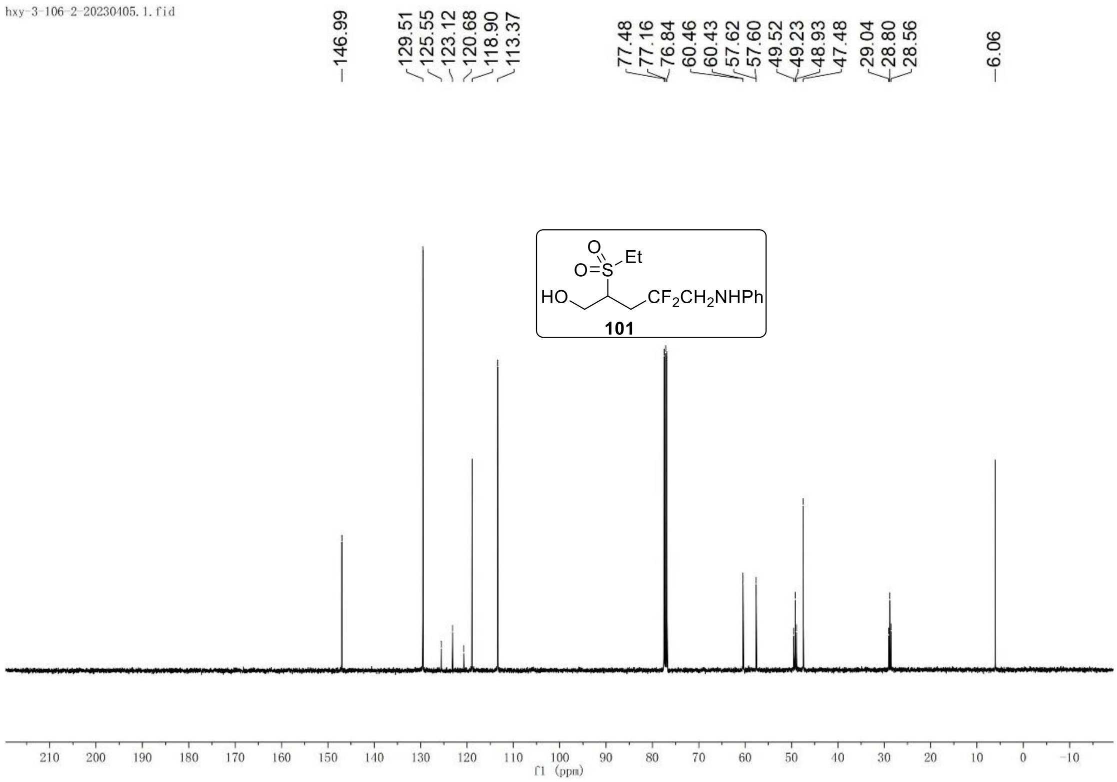

Supplementary Figure 346. ^{13}C NMR (101 MHz, $CDCl_3$) spectra of **101**

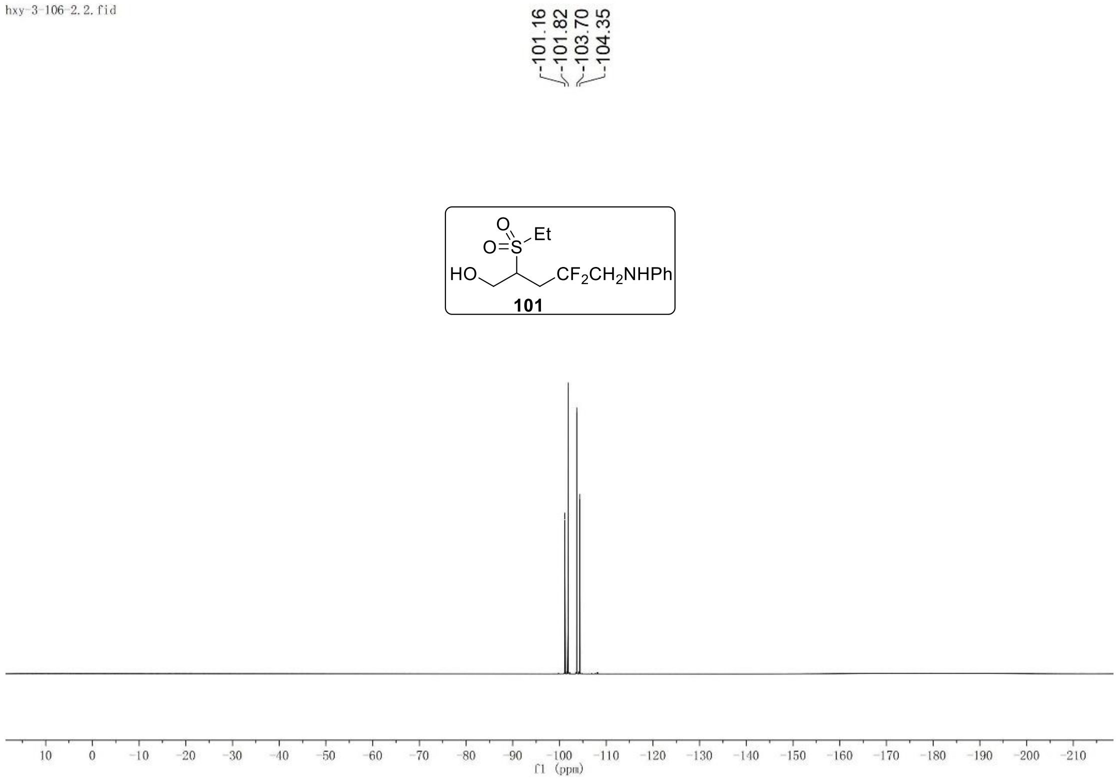

Supplementary Figure 347. ^{19}F NMR (376 MHz, $CDCl_3$) spectra of **101**

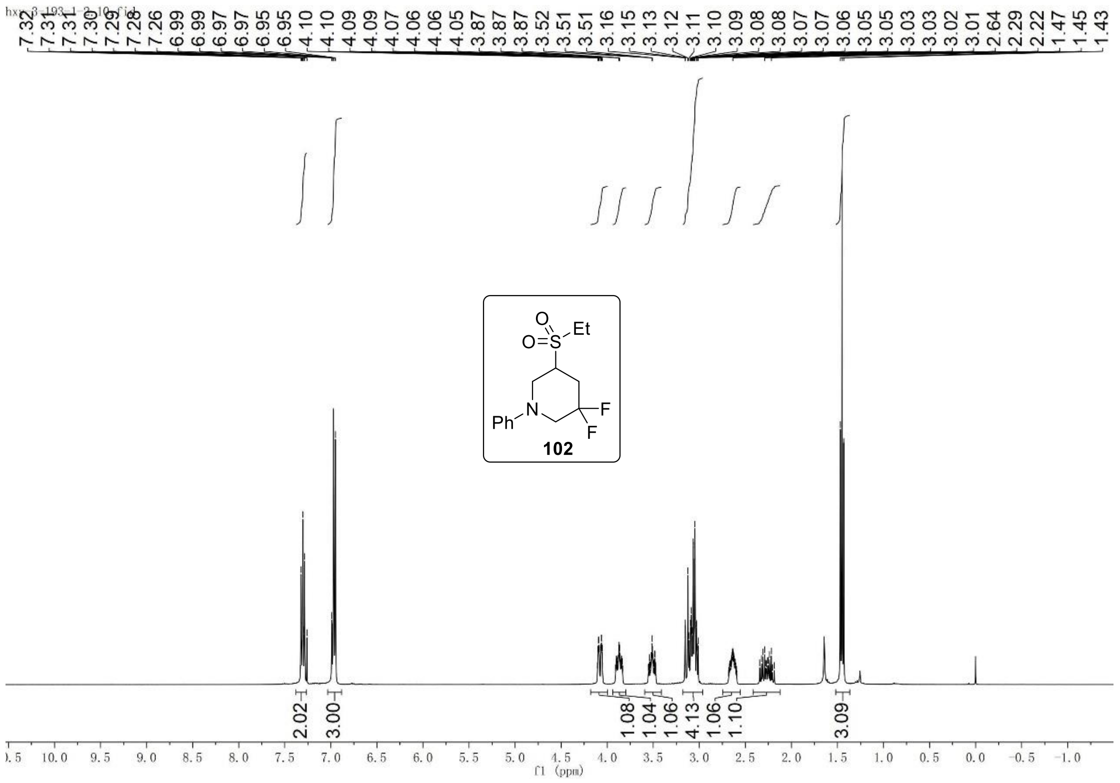

Supplementary Figure 348. ^{1}H NMR (400 MHz, $CDCl_3$) spectra of **102**

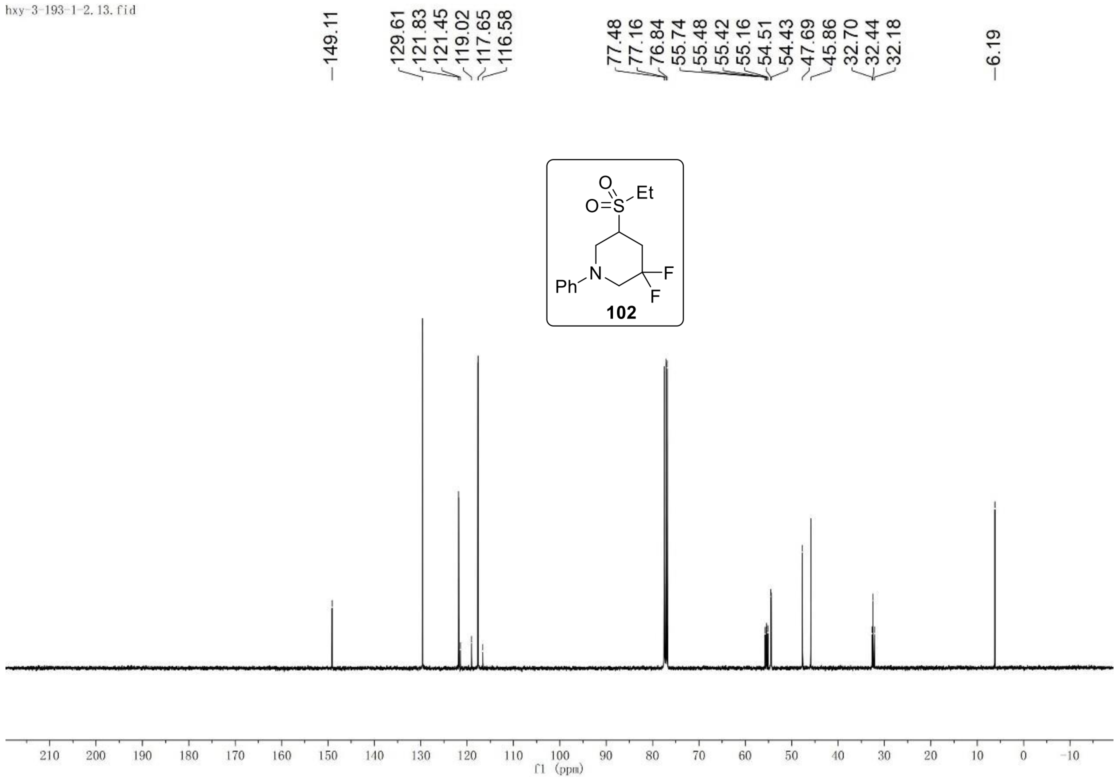

Supplementary Figure 349. ^{13}C NMR (101 MHz, $CDCl_3$) spectra of **102**

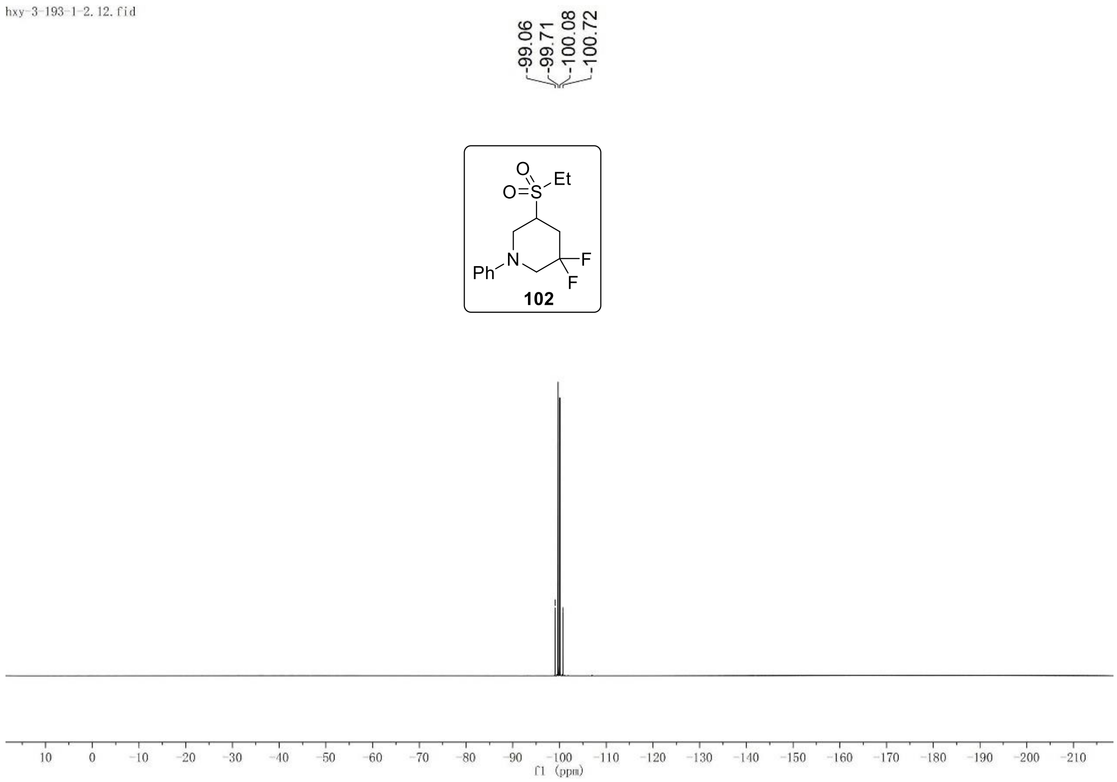

Supplementary Figure 350. ^{19}F NMR (376 MHz, $CDCl_3$) spectra of **102**

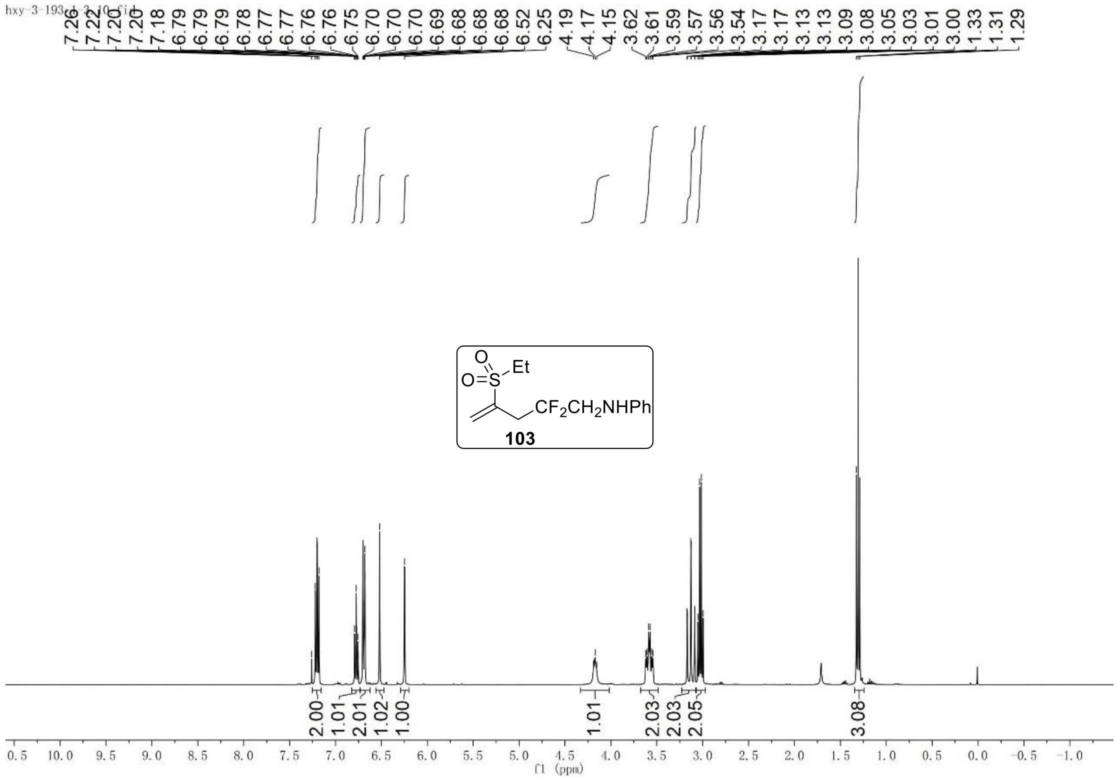

Supplementary Figure 351. ^{1}H NMR (400 MHz, $CDCl_3$) spectra of **103**

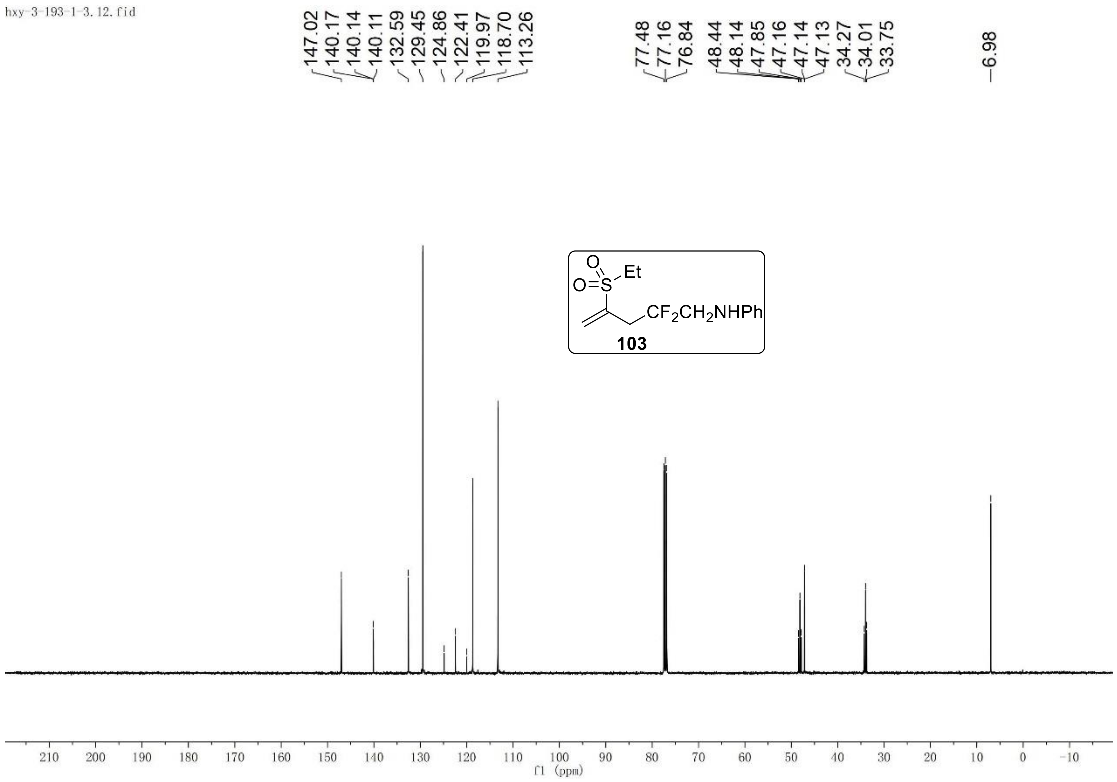

Supplementary Figure 352. ^{13}C NMR (101 MHz, $CDCl_3$) spectra of **103**

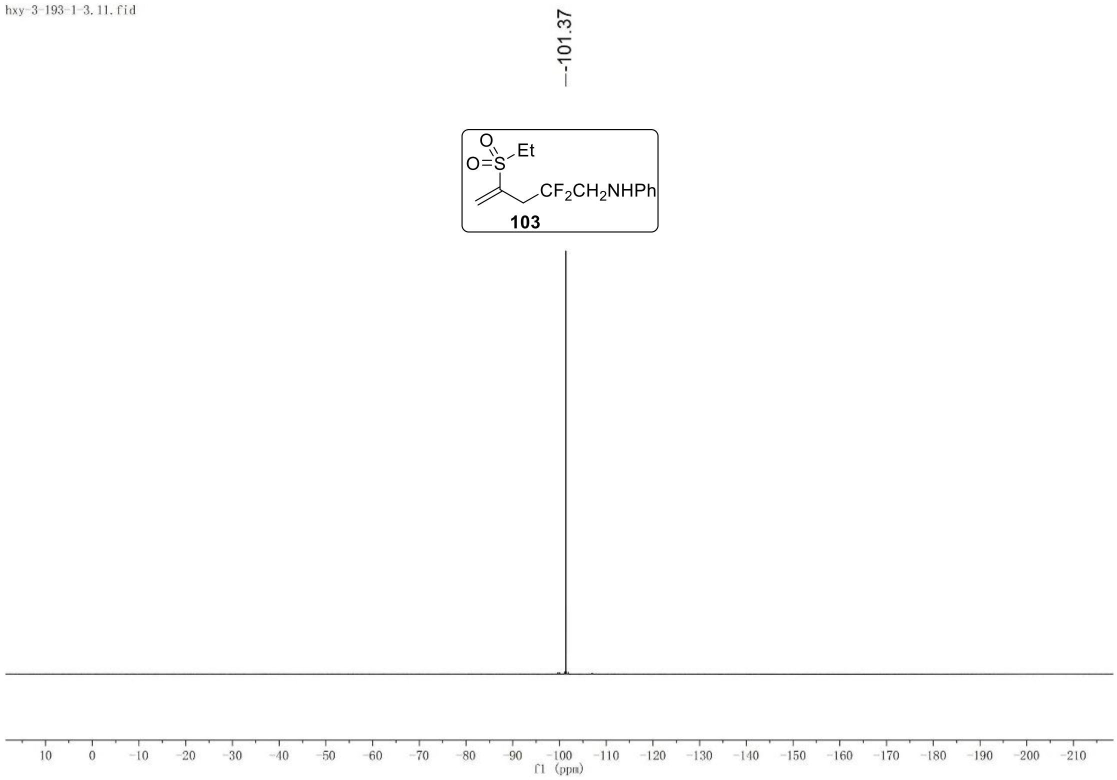

Supplementary Figure 353. ^{19}F NMR (376 MHz, $CDCl_3$) spectra of **103**

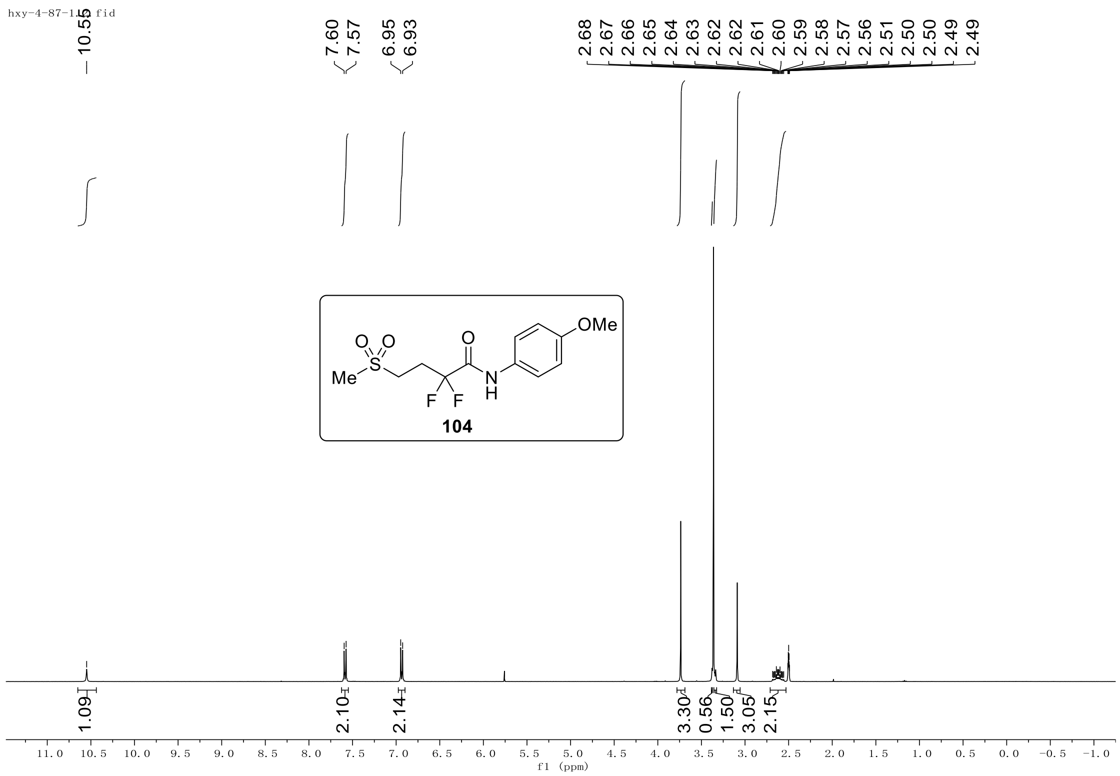

Supplementary Figure 354. ^{1}H NMR (400 MHz, DMSO-d_6) spectra of **104**

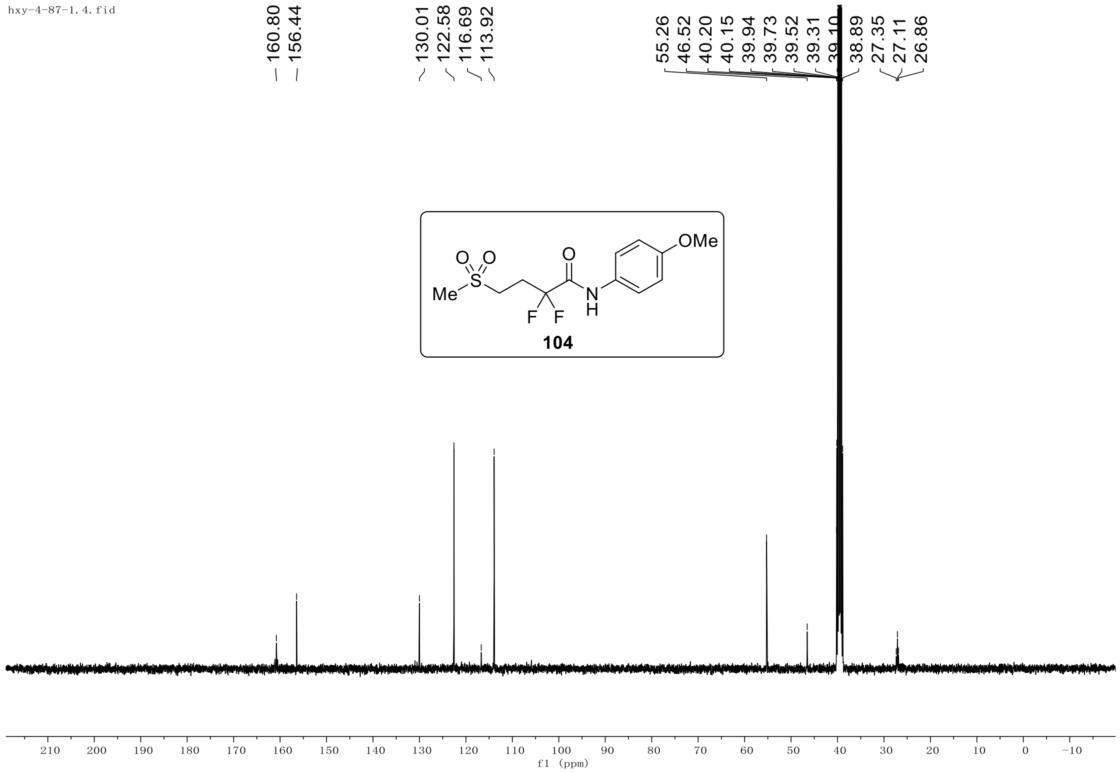

Supplementary Figure 355. ^{13}C NMR (101 MHz, DMSO-d_6) spectra of **104**

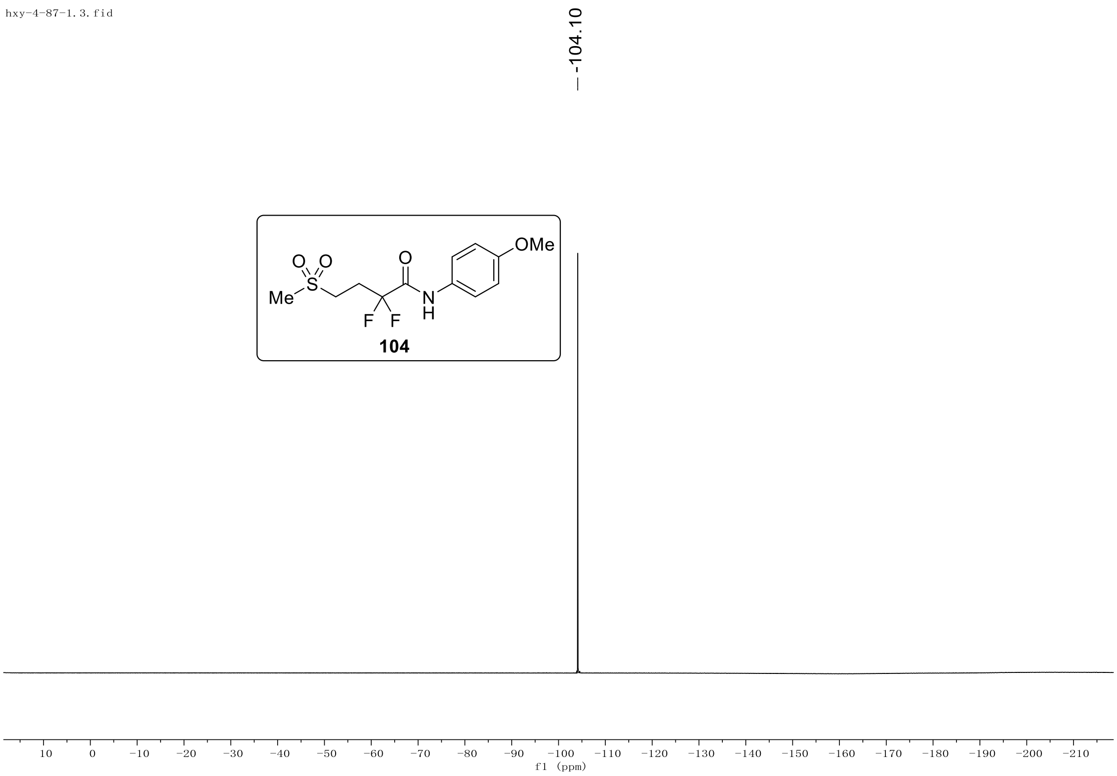

Supplementary Figure 356. ^{19}F NMR (376 MHz, DMSO-d_6) spectra of **104**

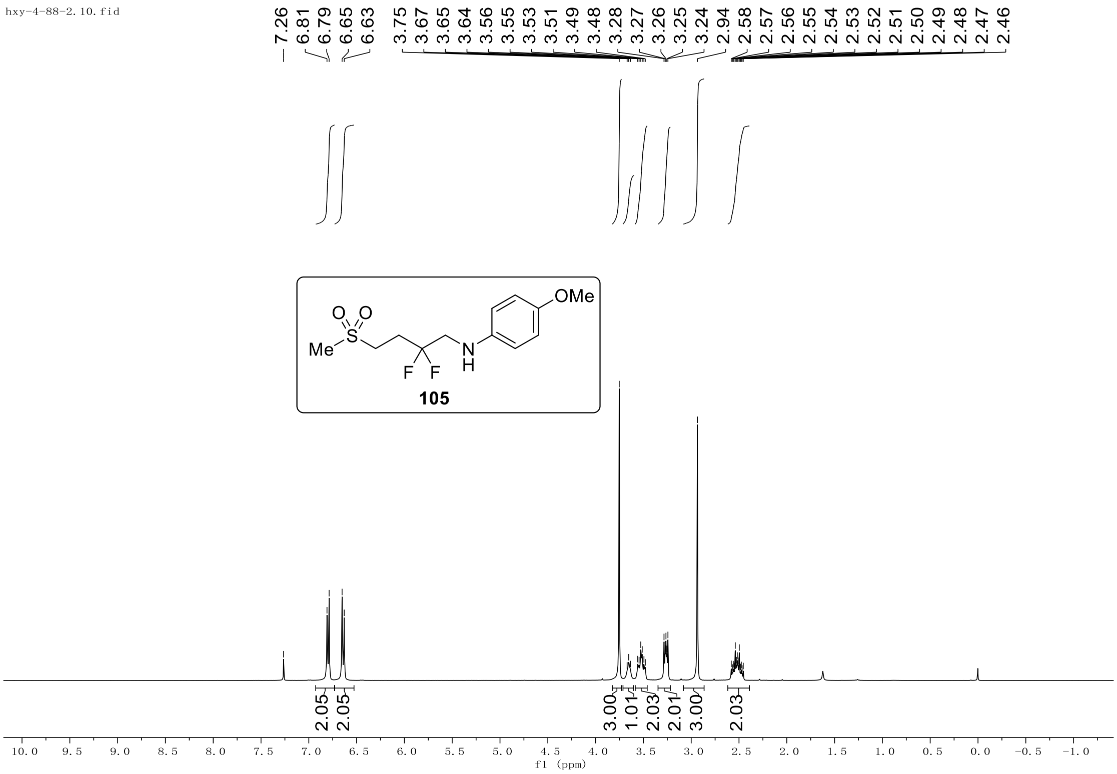

Supplementary Figure 357. ^{1}H NMR (400 MHz, $CDCl_3$) spectra of **105**

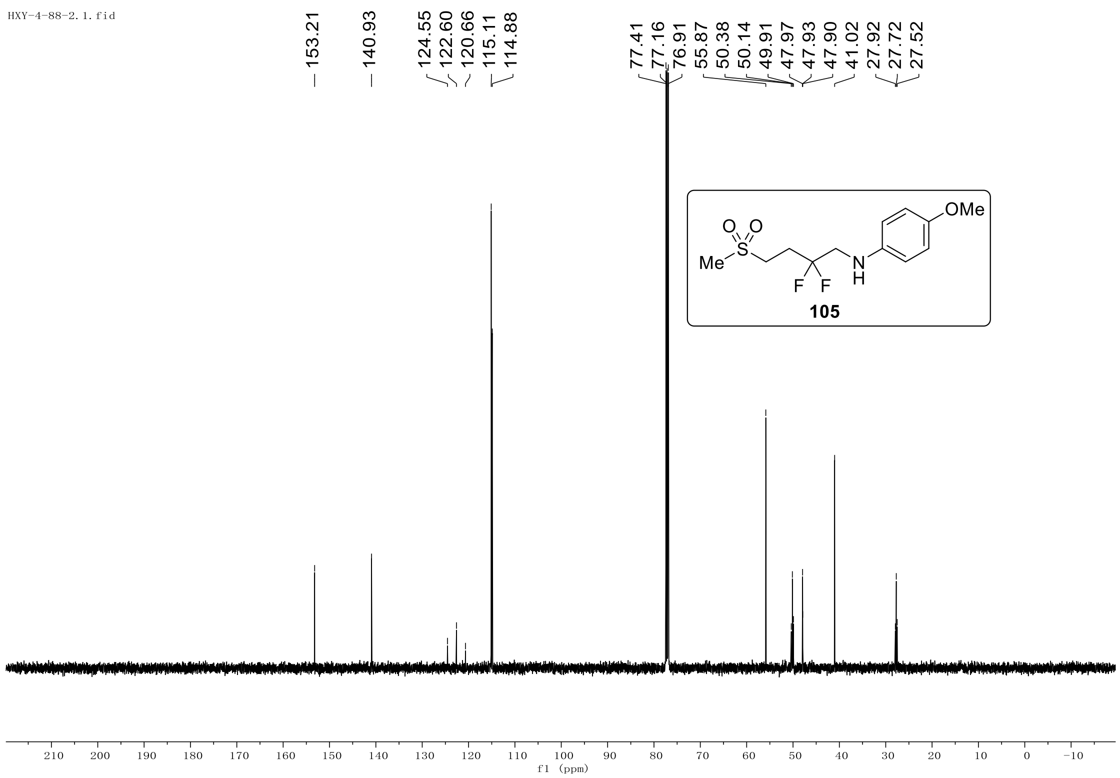

Supplementary Figure 358. ^{13}C NMR (126 MHz, $CDCl_3$) spectra of **105**

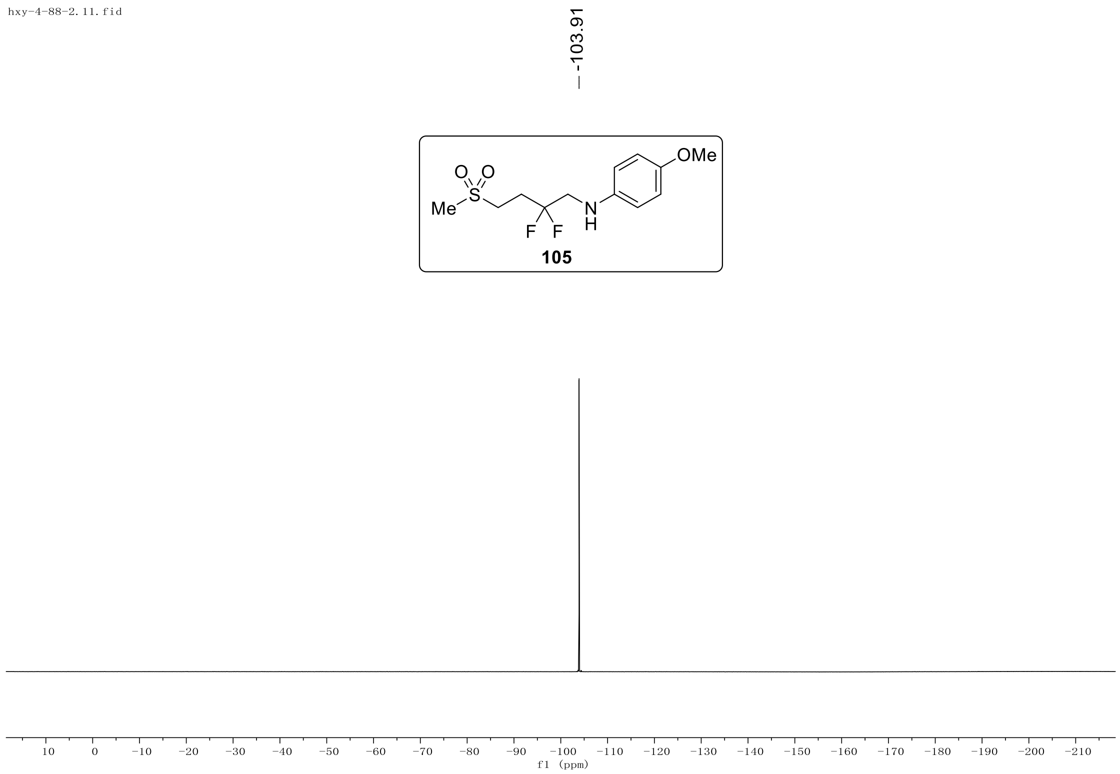

Supplementary Figure 359. ^{19}F NMR (376 MHz, $CDCl_3$) spectra of **105**

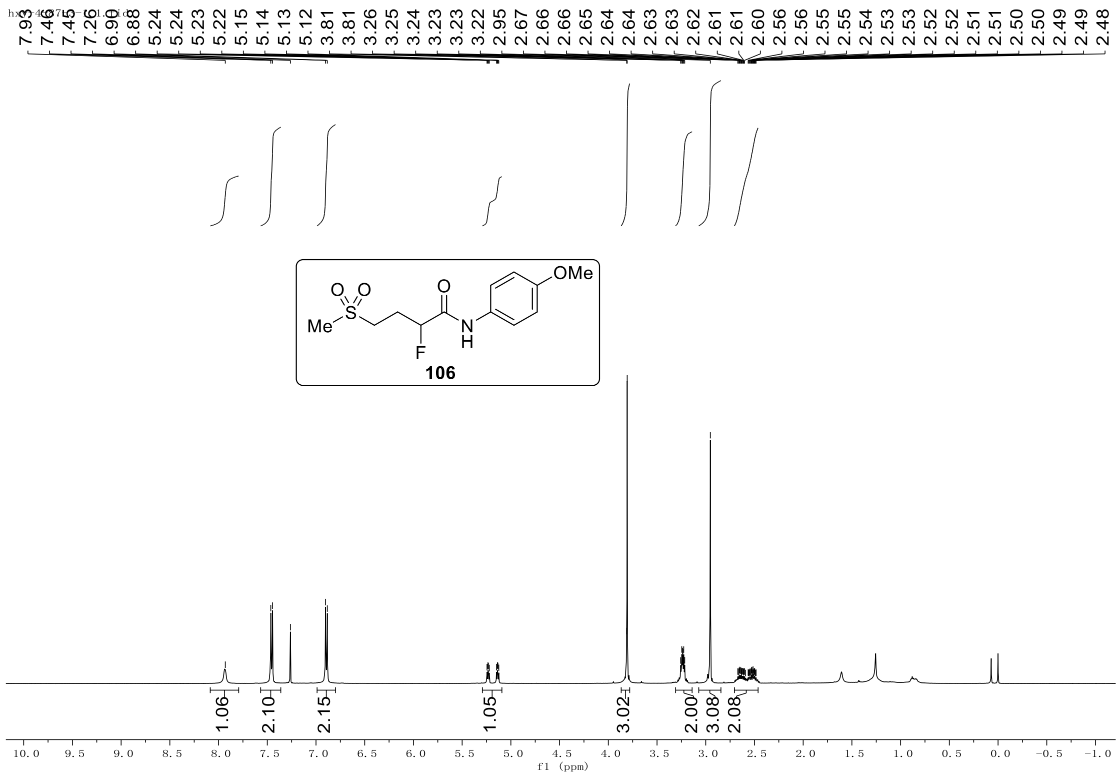

Supplementary Figure 360. ^{1}H NMR (500 MHz, $CDCl_3$) spectra of **106**

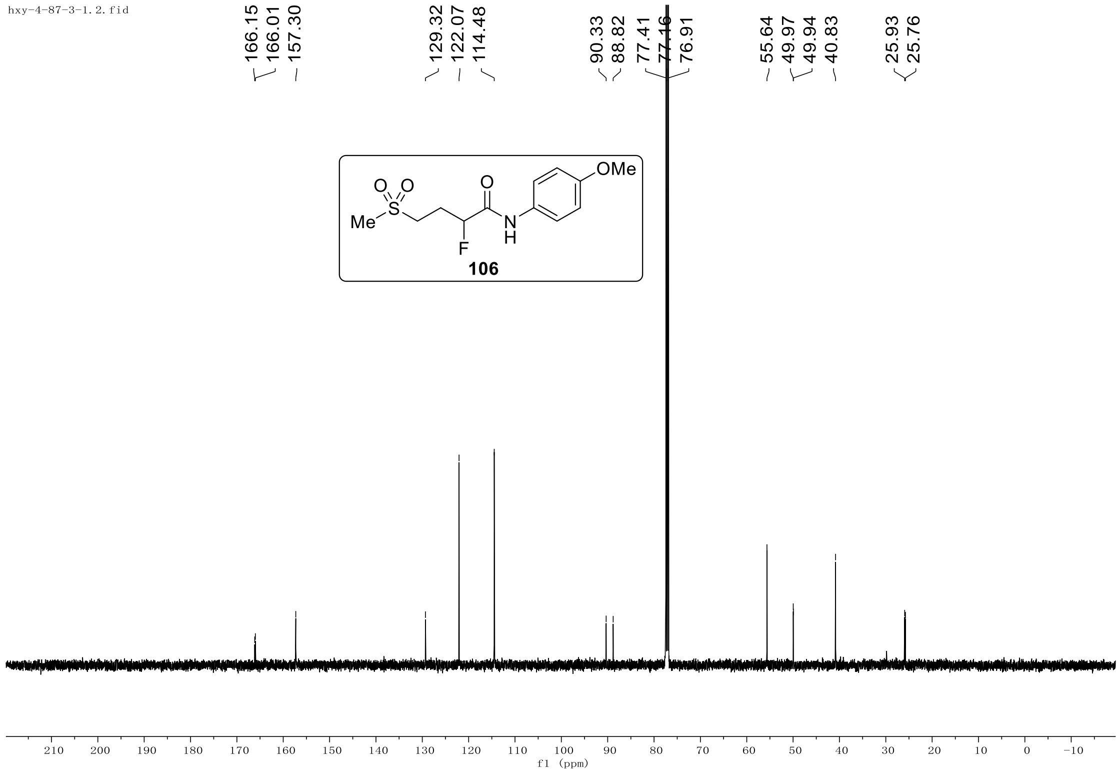

Supplementary Figure 361. ^{13}C NMR (126 MHz, $CDCl_3$) spectra of **106**

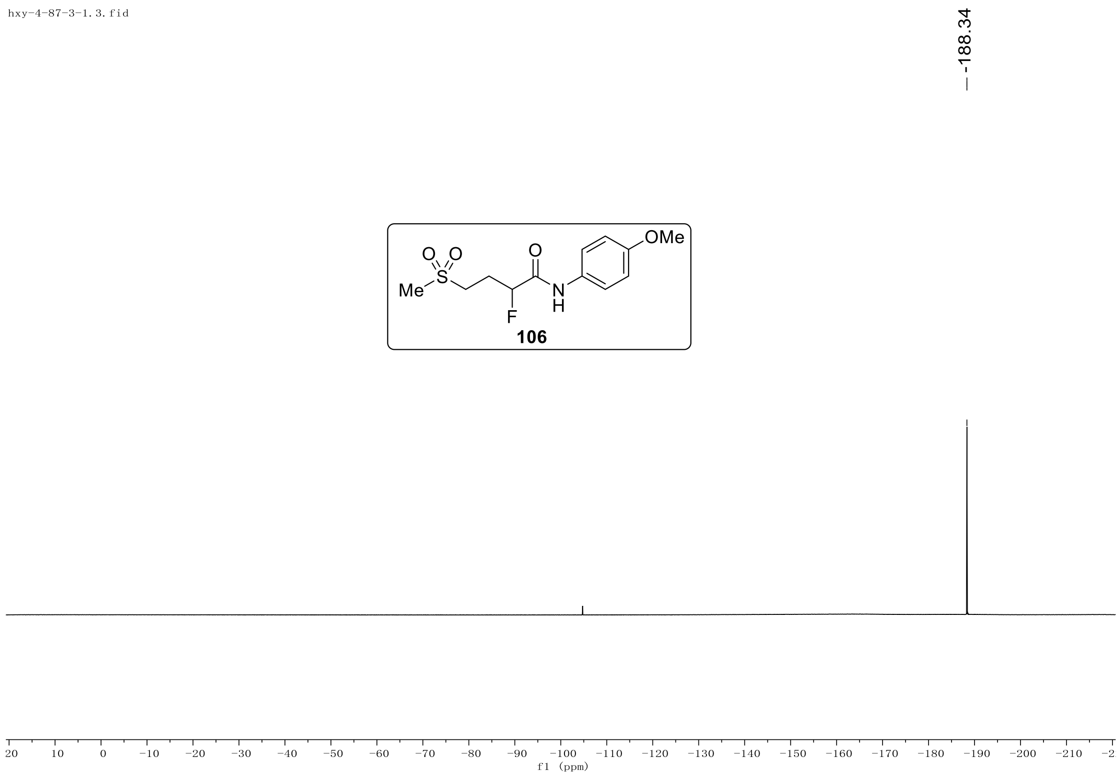

Supplementary Figure 362. ^{19}F NMR (471 MHz, $CDCl_3$) spectra of **106**

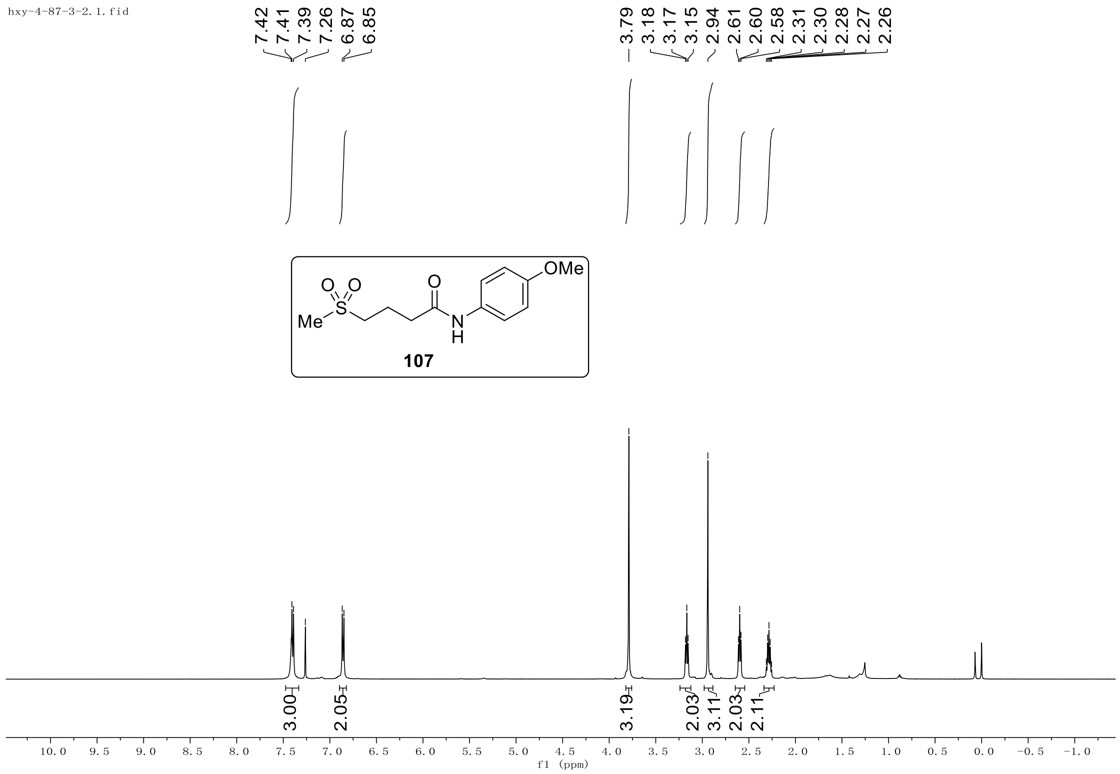

Supplementary Figure 363. ^{1}H NMR (500 MHz, $CDCl_3$) spectra of **107**

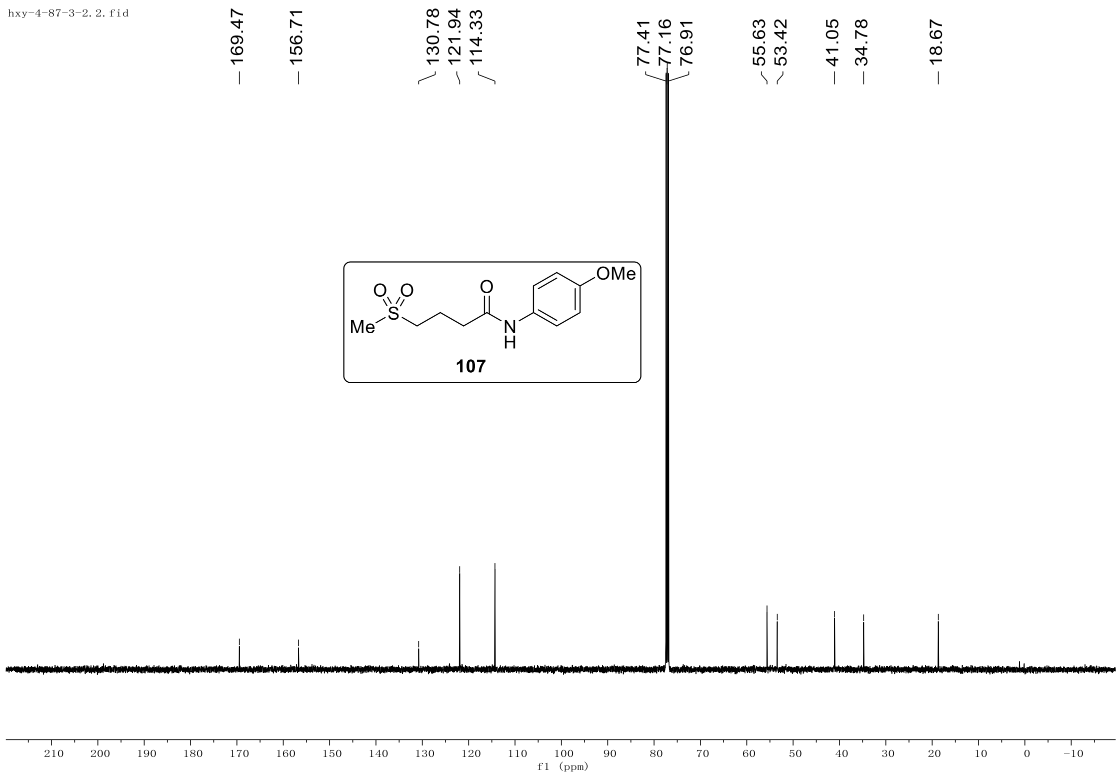

Supplementary Figure 364. ^{13}C NMR (126 MHz, $CDCl_3$) spectra of **107**

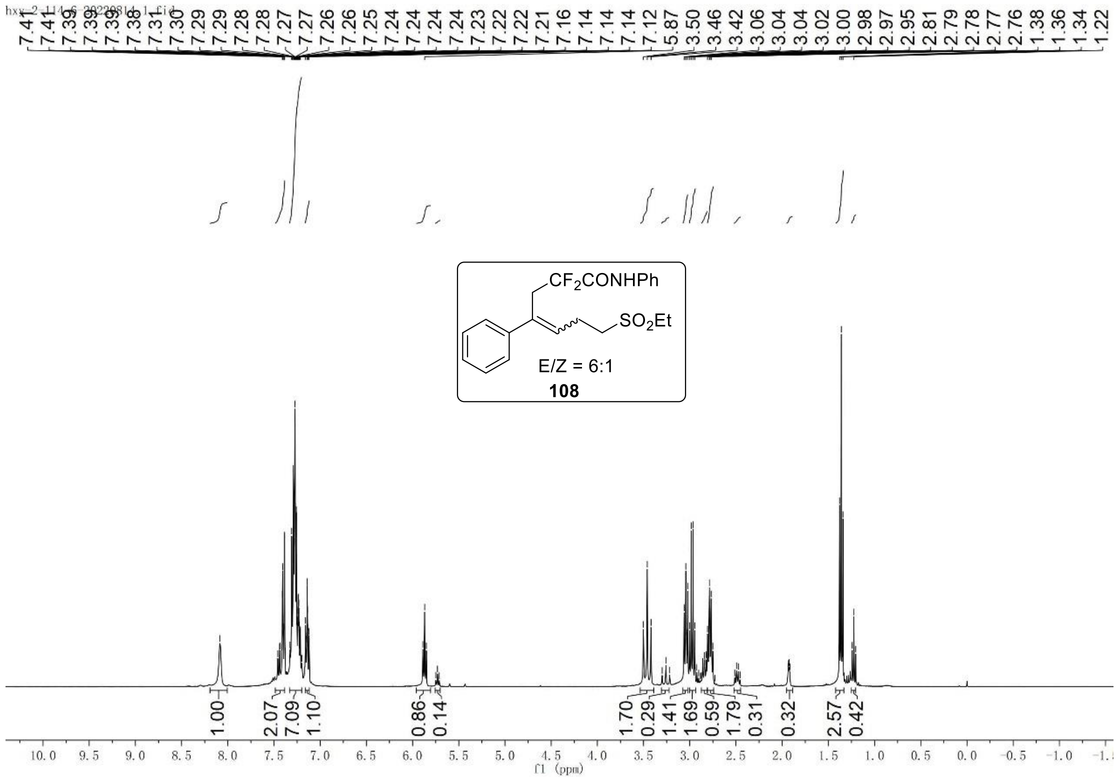

Supplementary Figure 365. ^{1}H NMR (400 MHz, $CDCl_3$) spectra of **108**

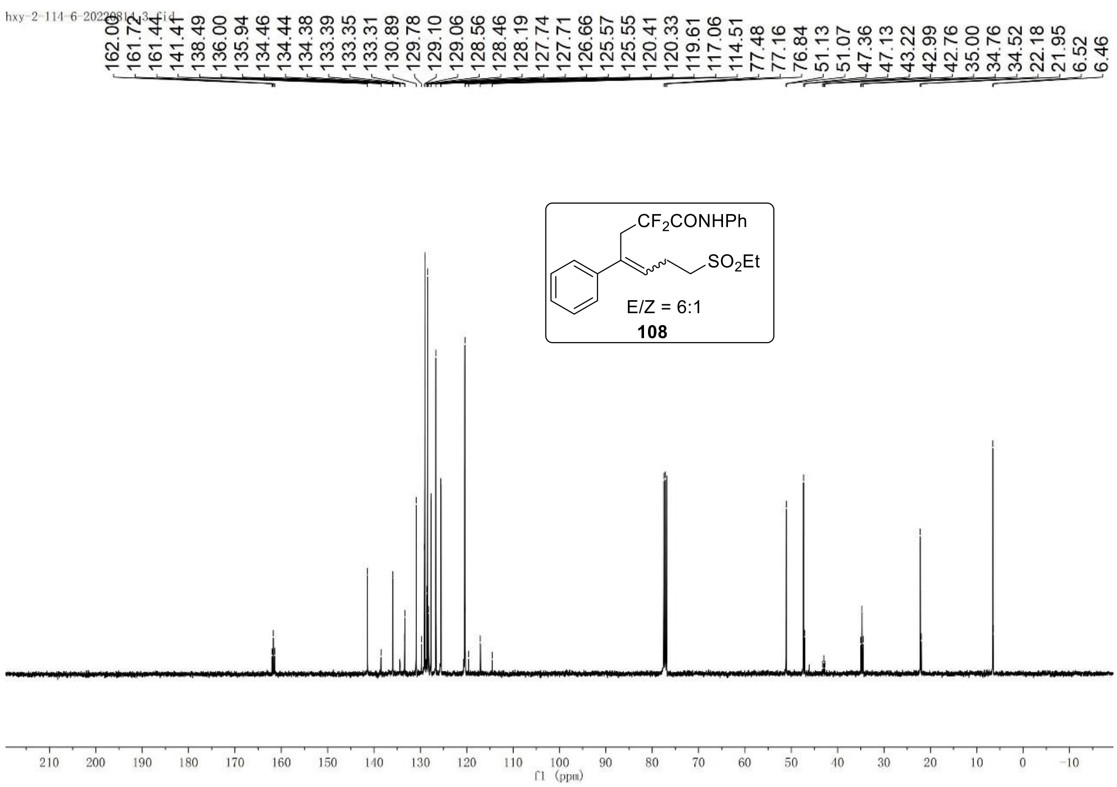

Supplementary Figure 366. ^{13}C NMR (101 MHz, $CDCl_3$) spectra of **108**

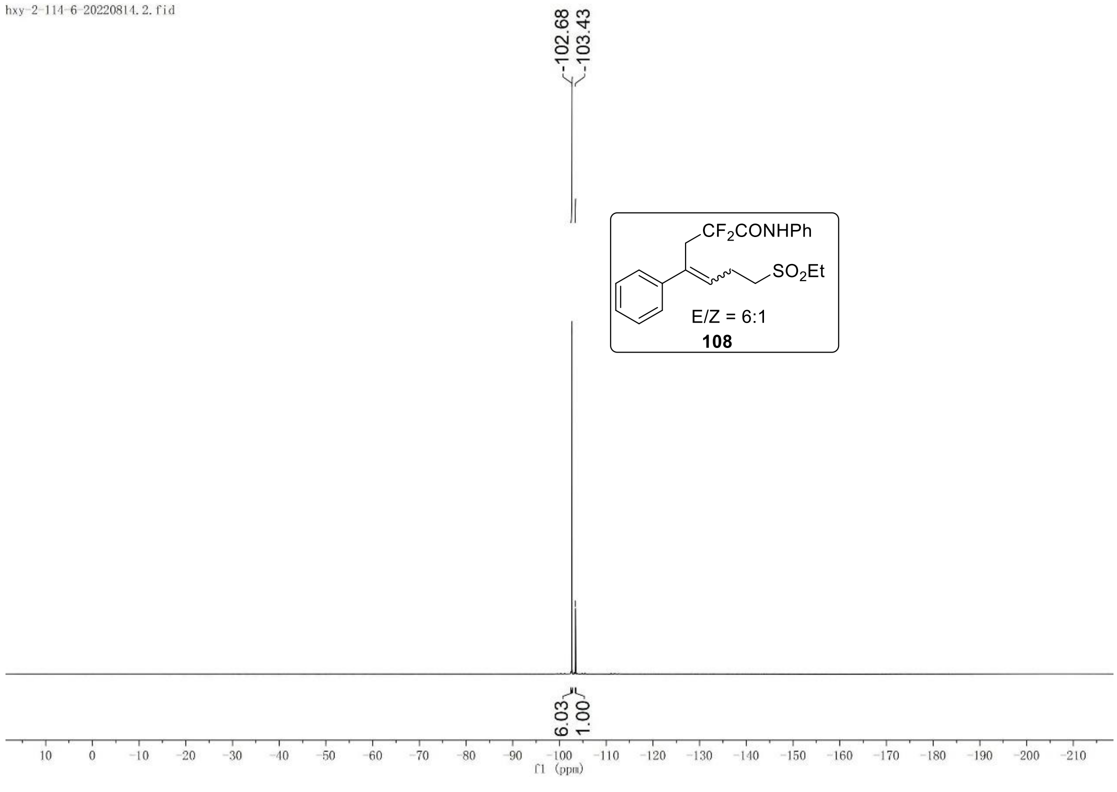

Supplementary Figure 367. ^{19}F NMR (376 MHz, $CDCl_3$) spectra of **108**

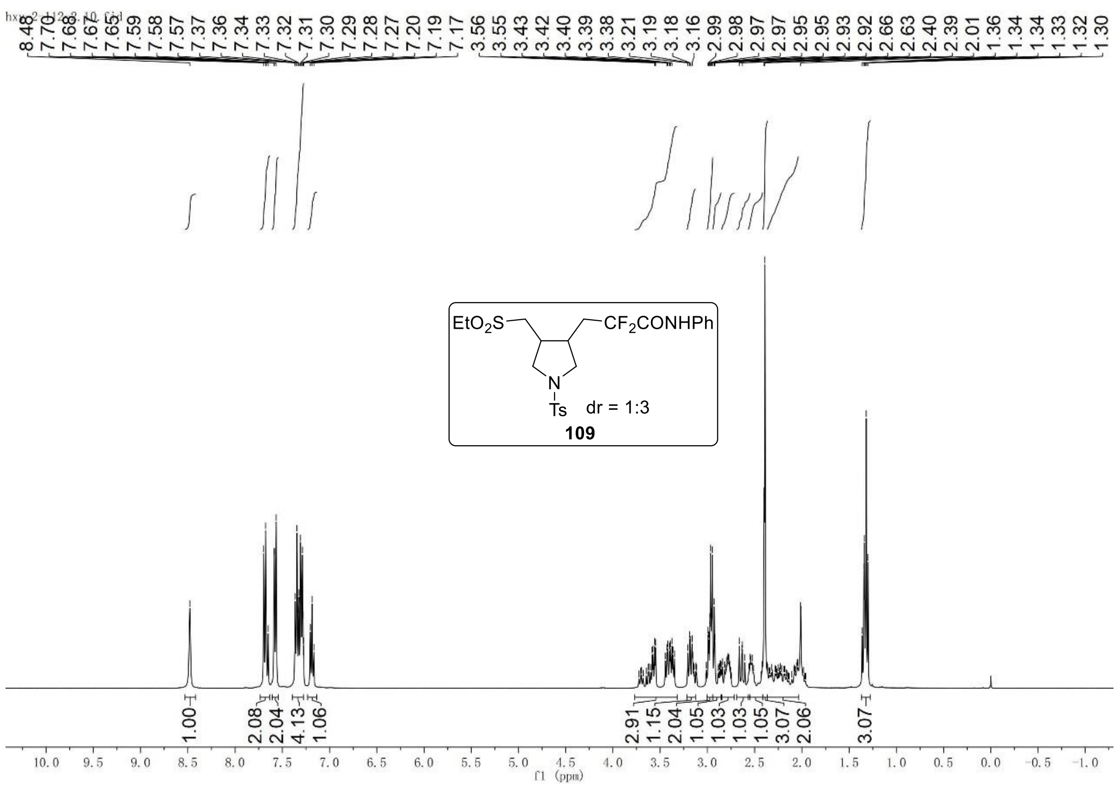

Supplementary Figure 368. ^{1}H NMR (400 MHz, $CDCl_3$) spectra of **109**

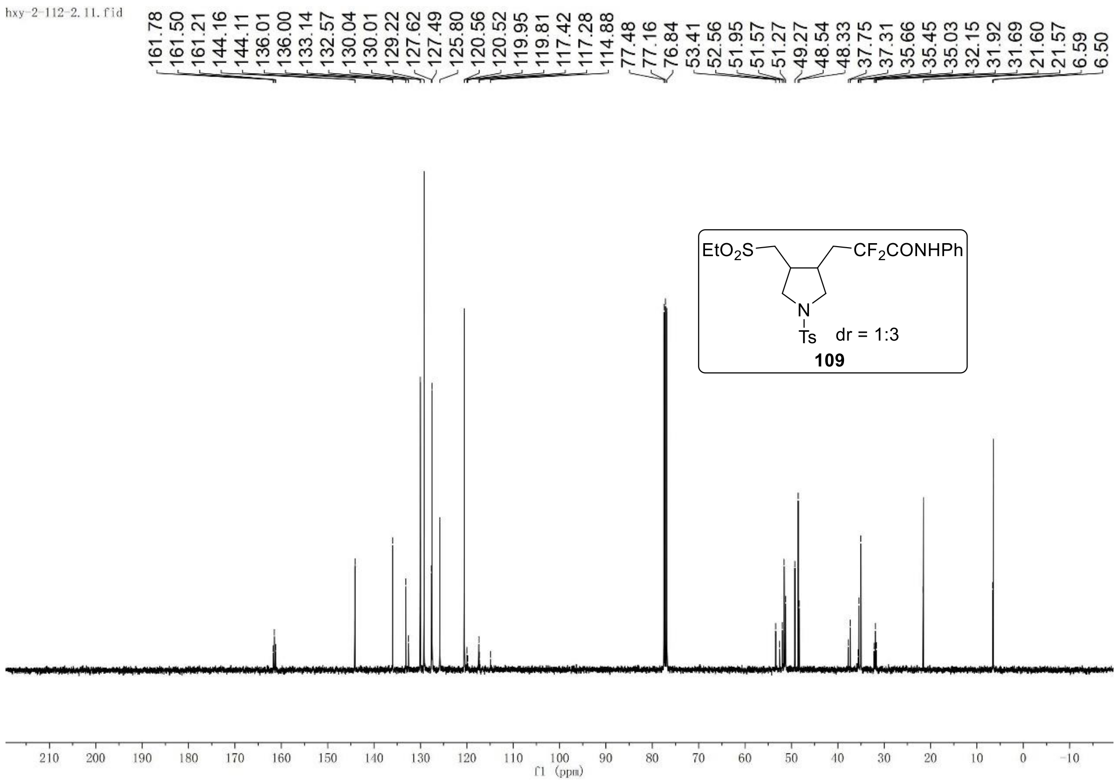

Supplementary Figure 369. ^{13}C NMR (101 MHz, $CDCl_3$) spectra of **109**

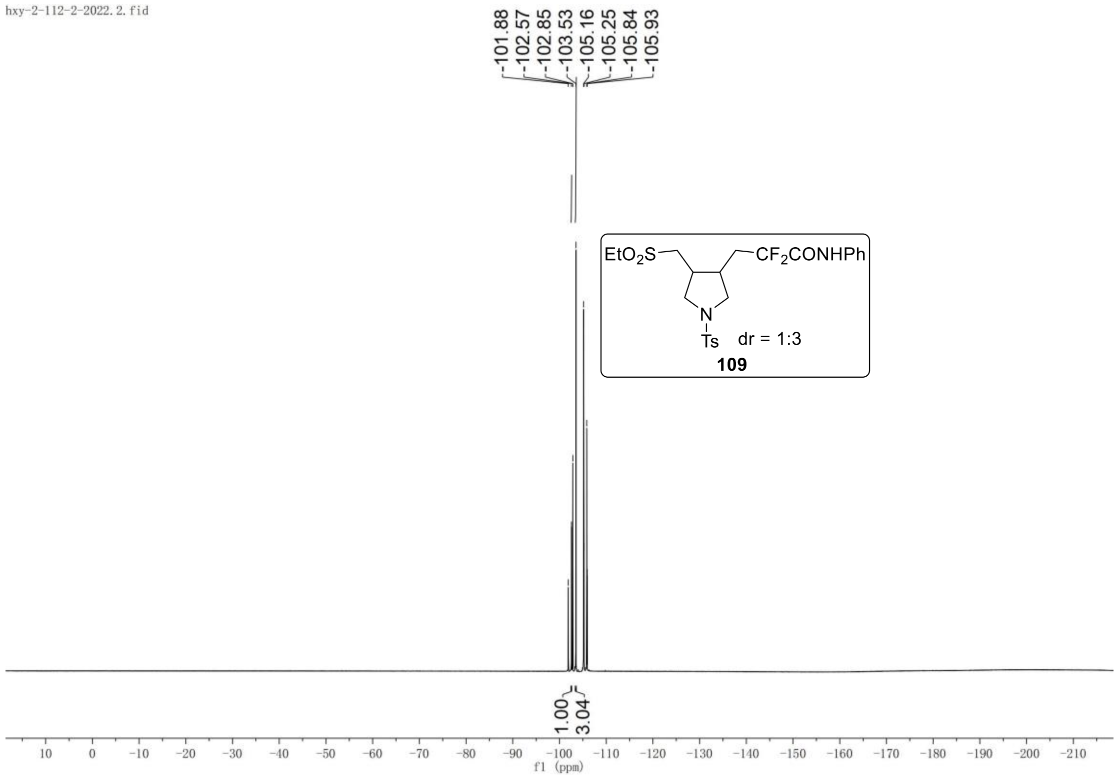

Supplementary Figure 370. ^{19}F NMR (376 MHz, $CDCl_3$) spectra of **109**

10. Supplementary References

1. Ye, J. -H., Bellotti, P., Heusel, C. & Glorius, F. Photoredox-catalyzed defluorinative functionalizations of polyfluorinated aliphatic amides and esters. *Angew. Chem. Int. Ed.* **61**, e202115456 (2022).

2. Wu, Z., Meng, J., Liu, H., Li, Y., Zhang, X. & Zhang, W. Multi-site programmable functionalization of alkenes via controllable alkene isomerization. *Nat. Chem.* **15**, 988-997 (2023).

3. Schwarz, L., Schäfers, F., Tlahuext-Aca, A., Lückemeier, L. & Glorius, F. Diastereoselective allylation of aldehydes by dual photoredox and chromium catalysis. *J. Am. Chem. Soc.* **140**, 12705-12709 (2018).

4. Ren, J., Du, F.-H., Jia, M.-C., Hu, Z.-N., Chen, Z. & Zhang, C. Ring expansion fluorination of unactivated cyclopropanes mediated by a new monofluoroiodane(III) reagent. *Angew. Chem. Int. Ed.* **60**, 24171-24178 (2021).

5. Soulard, V., Villa, G., Vollmar, D. P. & Renaud, P. Radical deuteration with D_2O: catalysis and mechanistic insights. *J. Am. Chem. Soc.* **140**, 155-158 (2018).

6. Kim, M., Shin, S., Koo, Y., Jung, S. & Hong, S. Regiodivergent conversion of alkenes to branched or linear alkylpyridines. *Org. Lett.* **24**, 708-713 (2022).

7. Li, X., He, S. & Song, Q. Diethylzinc-mediated radical 1,2-addition of alkenes and alkynes. *Org. Lett.* **23**, 2994-2999 (2021).

8. Maity, S., Kancherla, R., Dhawa, U., Hoque, E., Pimparkar, S. & Maiti, D. Switch to allylic selectivity in cobalt-catalyzed dehydrogenative heck reactions with unbiased aliphatic olefins. *ACS Catal.* **6**, 5493-5499 (2016).

9. Zidan, M., McCallum, T., Swann, R. & Barriault, L. Formal bromine atom transfer radical addition of nonactivated bromoalkanes using photoredox gold catalysis. *Org. Lett.* **22**, 8401-8406 (2020).

10. Azizi, M. S., Edder, Y., Karim, A. & Sauthier, M. Nickel(0)-catalyzed *N*-allylation of amides and *p*-toluenesulfonamide with allylic alcohols under neat and neutral conditions. *Eur. J. Org. Chem.* **22**, 3796-3803 (2016).

11. Yu, C., Ke, F., Su, J., Ma, X., Li, X. & Song, Q. Cu-Catalyzed three-component cascade synthesis of 1,3-benzothiazines from *ortho*-aminohydrazones and bromodifluoroacetamides. *Org. Lett.* **24**, 7861-7865 (2022).

12. Li, Y., Liu, J., Zhao, S., Du, X., Guo, M., Zhao, W., Tang, X. & Wang, G. Copper-catalyzed fluoroolefination of silyl enol ethers and ketones toward the synthesis of *β*-fluoroenones. *Org. Lett.* **20**, 917-920 (2018).

13. Mai, W.-P., Wang, F., Zhang, X.-F., Wang, S.-M., Duan, Q.-P. & Lu, K. Nickel-catalyzed radical tandem cyclization/arylation: practical synthesis of 4-benzyl-3,3-difluoro-γ-lactams. *Org. Biomol.*

Chem. **16**, 6491-6498 (2018).
14. Zhou, M., Zhao, H.-Y., Zhang, S., Zhang, Y. & Zhang, X. Nickel-catalyzed four-component carbocarbonylation of alkenes under 1 atm of CO. *J. Am. Chem. Soc.* **142**, 18191-18199 (2020).
15. Surapanich, N., Kuhakarn, C., Pohmakotr, M. & Reutrakul, V. Palladium-mediated Heck-type reactions of [(bromodifluoromethyl)sulfonyl]benzene: synthesis of α-alkenyl- and α-heteroaryl-substituted α,α-difluoromethyl phenyl sulfones. *Eur. J. Org. Chem.* **30**, 5943-5952 (2012).
